# Mind the Queue: A Case Study in Visualizing Heterogeneous Behavioral Patterns in Livestock Sensor Data Using Unsupervised Machine Learning Techniques

**DOI:** 10.3389/fvets.2020.00523

**Published:** 2020-08-13

**Authors:** Catherine McVey, Fushing Hsieh, Diego Manriquez, Pablo Pinedo, Kristina Horback

**Affiliations:** ^1^Department of Animal Science, University of California, Davis, Davis, CA, United States; ^2^Department of Statistics, University of California, Davis, Davis, CA, United States; ^3^Department of Animal Science, Colorado State University, Fort Collins, CO, United States

**Keywords:** milking order, exploratory data analysis, unsupervised machine learning, data mechanics, entropy, manifold learning, precision livestock

## Abstract

Sensor technologies allow ethologists to continuously monitor the behaviors of large numbers of animals over extended periods of time. This creates new opportunities to study livestock behavior in commercial settings, but also new methodological challenges. Densely sampled behavioral data from large heterogeneous groups can contain a range of complex patterns and stochastic structures that may be difficult to visualize using conventional exploratory data analysis techniques. The goal of this research was to assess the efficacy of unsupervised machine learning tools in recovering complex behavioral patterns from such datasets to better inform subsequent statistical modeling. This methodological case study was carried out using records on milking order, or the sequence in which cows arrange themselves as they enter the milking parlor. Data was collected over a 6-month period from a closed group of 200 mixed-parity Holstein cattle on an organic dairy. Cows at the front and rear of the queue proved more consistent in their entry position than animals at the center of the queue, a systematic pattern of heterogeneity more clearly visualized using entropy estimates, a scale and distribution-free alternative to variance robust to outliers. Dimension reduction techniques were then used to visualize relationships between cows. No evidence of social cohesion was recovered, but Diffusion Map embeddings proved more adept than PCA at revealing the underlying linear geometry of this data. Median parlor entry positions from the pre- and post-pasture subperiods were highly correlated (*R* = 0.91), suggesting a surprising degree of temporal stationarity. Data Mechanics visualizations, however, revealed heterogeneous non-stationary among subgroups of animals in the center of the group and herd-level temporal outliers. A repeated measures model recovered inconsistent evidence of a relationships between entry position and cow attributes. Mutual conditional entropy tests, a permutation-based approach to assessing bivariate correlations robust to non-independence, confirmed a significant but non-linear association with peak milk yield, but revealed the age effect to be potentially confounded by health status. Finally, queueing records were related back to behaviors recorded via ear tag accelerometers using linear models and mutual conditional entropy tests. Both approaches recovered consistent evidence of differences in home pen behaviors across subsections of the queue.

## Introduction

For much of its history, ethological research in livestock has relied on human observers to encode behaviors of interest (1). While developing a detailed ethogram and observer training protocols constitute no simple task, there are several inherent advantages to this approach for subsequent statistical analyses. Continuous involvement of a human in the incoming data stream allows many erroneous data points to be identified and excluded from downstream analyses that they might otherwise destabilize. Extensive involvement of research personnel in the data collection phase also nurtures a deeper familiarity with the system under study. This not only aids in the specification of an appropriate statistical model and interpretation of results, but is often critical in identifying unexpected behavioral patterns that can inspire novel hypotheses.

Unfortunately, the inherent quality of such data imposes practical limitations on the quantity that can be produced. This can restrict both the number of animals utilized in a study and the period of time over which they are observed. The later limitation can overlook important dynamic features of the behavioral patterns under consideration. Restrictions on the number of animals that can be studied, on the other hand, can fundamentally alter the behavioral mechanisms at play in a herd. For example, the linearity of dominance hierarchies are known to change with group size (2). As commercial herds and flocks become ever larger, this only serves to broaden the gap between experimental findings and the welfare challenges they are meant to inform. Subsampling of animals or observations windows may be employed to reduce the number of observations collected without restricting the size of the study system. If the pre-existing base of scientific literature does not provide clear guidance on the selection of target animals or focal periods, however, such strategies may risk overlooking finer-grain behavioral patterns and skewing inferences about the collective behavior of the group (3, 4).

In recent years, livestock sensor technologies have become a popular alternative to visual observation (5–8). While the behaviors recorded are neither as complex or as detailed as those quantified via an observational ethogram, such devices have the capacity to continuously monitor hundreds or even thousands of animals for extended periods of time. Such a substantial expansion in the bandwidth capacity of ethological studies creates many new opportunities to better understand the behavior of livestock, particularly in large-scale commercial settings, but also raises new methodological challenges. Replacing nuanced human intuition with basic computer logic may increase the risk of erroneous data points, an issue that is only further compounded by the scale of data produced by such technologies, which renders many conventional visualizations techniques ineffective in identifying outliers. Observations recorded over extended time periods with high sampling frequency from large heterogeneous social groups may also contain a range of complex stochastic features—autocorrelation, temporal non-stationary, heterogeneous variance structures, non-independence between experimental units, etc.—that can lead to spurious inferences when not appropriately specified in a conventional liner model. The hands-off and somewhat black-boxed nature of many sensor platforms, however, do not nurture the intuition needed to identify many of these model structures *a priori*. Such insights must instead be drawn directly from the data itself, but here again, standard visualization tools may not scale to such large datasets.

Unsupervised machine learning (UML) tools offer a distinct empirical approach to knowledge discovery that are purpose built for large and complex datasets (9). Whereas, conventional linear models excel at providing answers to targeted experimental hypotheses, UML algorithms strive to identify and characterize the non-random patterns hiding beneath the stochastic surface of a dataset using model-free iterative techniques that impose few structural assumptions. This open-ended and highly flexible approach to data exploration may offer an empirical means by which to recover much of the familiarity with a study system that is lost with the shift from direct observation to sensor platforms. The purpose of this research was contrast the behavioral insights gleaned from UML algorithms with those recovered using conventional exploratory data analysis (EDA) techniques, and to then explore how such information could be best integrated into standard linear analysis pipelines.

Milking order, or the sequence in which cows enter the parlor to be milked, is recorded in all RFID (Radio Frequency Identification) equipped milking systems, making such records one of the most universal automated data streams to be found on modern dairies. Despite their ubiquity, such records are seldom used to inform individual or herd-level management strategies. This lack of utility, however, has not been for lack of study. Milking order has been the subject of scientific study since 1950's (10), with early investigators speculating that such records might contain pertinent information about individual cow productivity (11, 12), health (13), and social status (12, 14, 15). The modest base of scientific literature that has since been compiled on this topic, however, has struggled to recover repeatable evidence of such associations (16–19). While such inconsistency may simply reflect non-uniformity in the behavioral strategies driving queueing patterns across different herds and farm environments, misspecification of the linear models used to describe this system could also contribute to volatility in these statistical inferences. The objective of this methodological case study will be visualize the various stochastic aspects of such records using UML tools in an effort to identify erroneous data points and heterogeneous variance structures that may not be recovered using conventional EDA techniques.

## Methods

### Study Animal Management

Data for this case study was repurposed from a feed trial assessing the effect of an organic fat supplement on cow health and productivity through the first 150 days of lactation (20). All animal handling and experimental protocols were approved by the Colorado State University Institution of Animal Care and Use Committee (Protocol ID: 16-6704AA). The study ran from January to July of 2017 on a certified organic dairy in Northern Colorado. A total of 200 mixed-parity Holstein cows were enrolled over a 1.5 month period as study-eligible animals calved. Cows were maintained in a closed herd for the duration of the study, with sick animals temporarily removed to a hospital pen when necessary. The study pen was an open-sided free stall barn, stocked at just above half capacity with respect to bunk space and beds, with free access to an adjacent outdoor dry lot. At roughly the midpoint of the trial, cows were moved overnight to a grass pasture that conformed with organic grazing requirements [for more details on pen setup see (21)]. Cows had access to total mixed ration (TMR) ration following each milking. Animals were temporarily split into two subsections of the pen following the morning milking to facilitate administration of control and treatment diets. Cows remained locked for roughly 45 min following this division so that farm and research staff could collect health and fertility data. Additionally, all animals were fitted with CowManager® ear tag accelerometers (Agis Automatisering BV, Harmelen, Netherlands). This commercial sensor platform, while designed and optimized for disease and heat detection, also provided hourly time budget estimates for total time (min) engaged in a range of behaviors—eating, rumination, non-activity, activity, and high activity—as well as average skin temperature.

### Data Wrangling

Raw milk logs were exported from the rotary parlor following each morning milking (ALPRO, DeLaval, Tumba, Sweden), and were processed using data wrangling tools available in R version 3.5.1 (22). To account for missing records due to illnesses and RFID reader errors, ordinal entry positions were normalized by the total number of cows recorded in a given milking (18). Transforming the data to an entry quantile served to make the domain restriction uniform across days. Additionally, “dividing out” daily variations in herd size served to prevent this uncontrolled experimental factor from artificially increasing individual variability in entry position. For example, if a cow were always the last animal to enter the parlor, her ordinal entry position might vary widely with herd size, but her entry quantile would always be 1.

The first 55 days of records were excluded from analyses to allow all animals to enter the herd over the rolling enrollment period and become established in their parlor entry position (16). To avoid irregularities in cow movements, several observation days surrounding management changes were also dropped, including: the 2 days preceding transition to pasture, the 4 days following pasture access, and the final 7 days on trial. Any days where <75% of the herd was successfully recorded in the parlor were also dropped. This left a total of 80 days of milk order observations−26 recorded while cows remained overnight in their pen, and 54 after the transition to overnight pasture. Finally, cows that were not present in at least 50% of the remaining milkings were excluded from further analysis. Of the 177 cows with sufficient records, 114 had no recorded health events.

### Quantifying Degree of Randomness

The first step in understanding this system was to determine if there was any evidence of non-random patterns in queue formation. Had this data been collected observationally, researchers might have simply noted if the same cows consistently entered the parlor in a given section of the queue. Standard summary statistics do not readily lend themselves to making an equivalent empirical determination, however, as cow identity is a discrete variable with no inherent nominal value. This issue is encountered fairly regularly in ethological studies, where many qualitative behaviors have no natural ordering, such as: locations occupied in a pen at discrete time points, conspecifics an individual interacts with, feedstuffs or enrichment items engaged with, etc. Here we use entropy to quantify the stochasticity of cow-membership within each fixed quantile range (H_q_). To compute these values, the queue was divided into 20 equally-sized segments (*q*_0−0.05_, ..., *q*_0.95−1_). For each queue segment, counts were generated to determine how frequently each individual cow had been observed in that range of entry quantiles. Shannon's entropy was then calculated conditional of queue segment (q) using the formula in Equation 1 (23).

(1) $$\begin{array}{r} H_{q}=-\underset{c\;=\;cow}{\sum }\frac{n_{c|q}}{N_{q}}{\;}^{*}\log_{2}\left(\frac{n_{c|q}}{N_{q}}\right) \end{array}$$

$$\begin{array}{l} n_{c|q}=total\;times\;a\;given\;cow\;\left(c\right)\;is\;observed\;in\;quantile\\ \;position\;\left(q\right)\\ N_{q}\;=\;total\;animals\;observed\;in\;queue\;segment\;\left(q\right)\;across\\ \;observed\;milkings \end{array}$$

With this metric, the more consistently a smaller set of cows are observed in a given segment of the queue, the smaller the entropy values becomes to reflect less stochasticity in the system. In standard statistical models, the nominal value of estimators such as loglikelihood and AIC scale with the size of the data set, and must be interpreted relative the value of equivalent terms assessed against a null model. Analogously, the nominal value of the entropy estimates scales with the number of discrete categories used. The maximum theoretical value occurs when no underlying deterministic structures are present and all categories are equally likely to occur, which algebraically simplifies to the log of the number of discrete categories used (23). Here the maximum theoretical entropy value would be log_2_(114) = 6.83. To visually contrast differences in stochasticity across the queue, the observed entropy values were plotted against the median entry quantile of the corresponding queue segment using the *ggplot2* package, with maximum theoretical entropy added as a horizontal reference line (24).

Non-random patterns in queue formation could also be explored by tracking the entry position of individual cows over time. As entry quantile has a numerical value, we can now also use variance to quantify and contrast stochasticity between animals. As with all analytical approaches reviewed in this paper, there are both strengths and shortcomings to either approach (Table 1). In this system there are two potential drawbacks to this conventional summary statistic. The first is that variance estimates are quite sensitive to outliers, making it difficult to empirically distinguish between cows that occupy a wider range of queue positions and animals who typically occupy a narrower range but might have gotten jostled far from their normal position on one or several occasions. The second drawback is that, because variance quantifies dispersion about a central value, it cannot distinguish between cows that demonstrate little consistency in entry position and multimodal queuing patterns. For example, if a cow always entered the parlor either first or last, we would intuitively determine that this pattern is non-random, but the corresponding variance estimate would be the largest in the herd. These issues are circumvented, however, by discretizing entry quantile values and again using entropy to quantify stochasticity. To evaluate an individual cow's variability in quantile range-memberships (H_c_), count data was used to recalculate Shannon's entropy conditional on cow ID (c) using Equation 2 (23).

(2) $$\begin{array}{r} H_{c}=-\underset{q\;=\;Queue\;Segment\;}{\sum }\frac{n_{q|c}}{N_{c}}{\;}^{*}\log_{2}\left(\frac{n_{q|c}}{N_{c}}\right) \end{array}$$

$$\begin{array}{l} \;n_{q|c}=total\;times\;a\;given\;cow\;\left(c\right)\;is\;observed\;in\;quantile\;position\;\left(q\right)\\ N_{c}\;=\;total\;number\;of\;days\;a\;given\;cow\;\left(c\right)\;was\;observed\;in\;the\;queue \end{array}$$

Here the maximum possible entropy value, signifying a cow is equally likely to occupy any queue segment, would be log_2_(20) = 4.32. Observed entropy and variance values were visually compared using the *ggplot2* package (24). To test if an individual cow demonstrated less stochasticity in entry positions than would be expected with a purely random queueing process, entry quantile values were again permuted within each observation day, and both variance and entropy recalculated. This process was repeated over 5,000 iterations to generate empirical cumulative density functions (CDFs) for both stochasticity estimators under the null, which were then used to estimate *p*-values for the corresponding observed values.

**Table 1:** Summary of analytical approaches compared in this manuscript, and a comparison of their relative strengths and shortcomings.

**Analytical Goal**	**Approach**	**Strengths**	**Shortcomings**
Quantifying randomness	Variance	• Uses continuous measures	• Cannot use categorical data • Assumes unimodality • Sensitive to outliers
	Entropy	• Uses categorical data • Permits Multimodality • Robust to Outliers	• Continuous measures must be discretized, which can result in loss of information
Visualizing inter-animal relationships	PCA	• No metaparameters • Assess embedding via loadings	• Assumes latent structures are linear (additive)
	Diffusion map	• No linearity assumption	• Employs metaparameters • Embedding qualitatively assessed using visualizations
Visualizing temporal non-stationarity	Residual plots of stational repeated measures model	• Considers all data points simultaneously	• Non-homogeneous temporal trends may be overlooked
	Time × Response scatter plots for each cow	• Easy to create and visually assess for non-stationarity	• Difficult to contextualize trends across entire herd
	Data mechanics plot	• Simultaneously visualize social and temporal structure • Non-homogeneous temporal trends visually enhanced	• Row and column cluster granularity must be determined visually
Association between queue position and cow attributes	Linear mixed effect model	• Targeted hypotheses • Simultaneous estimation of multiple covariates	• Non-independence between animals inflates rate of Type I Errors
	Mutual conditional entropy permutation test	• Robust to between-animal non-independence • Detects non-linear patterns	• Cannot adjust for influence of other variables (confounders)
Association between queue position and accelerometer logs of home pen behavior	Linear mixed effect model	• Targeted hypotheses	• Convergence issues with two large sets of repeated measures
	Mutual conditional entropy permutation test	• Generalized pattern detection • Easily extended to large data	• Cannot adjust for confounding variables

### Visualization of Inter-animal Relationships

Having recovered evidence of non-random patterns, the next step was to begin characterizing the behavioral mechanisms driving this heterogeneity. The most fundamental question that need be answered to inform further analysis was the degree to which queueing patterns were driven by individual or collective behaviors. Because cows jockey for position with one another in the crowd pen, where they are pushed up to enter the parlor, we know intuitively that entry quantile records cannot be considered truly independent observations. If cows move through this melee as independent agents, such that their position within the queue is determined by individual attributes—preferences, dominance, etc. —then a linear model may still provide a reasonable approximation of the underlying system. Early observational work on milking order, however, has suggested that cows may form consistent associations when entering the milking parlor, particularly when heifers are reared together (13, 25). If cows move into the parlor in cohesive units, such that queue position is more determined by clique-level than individual attributes, then network analyses may be a more appropriate.

Principal Component Analysis (PCA) is commonly employed to visualize relationships between observational units in high dimensional datasets. In this approach, redundancy between variables, here each milking record, is captured using either covariance or correlation assessed across all data points, here all animals. An eigenvector decomposition is then used to linearly compress the information contained in the data via rotation of the orthogonal axes. New axes (loadings) are added iteratively such that each new dimension is pointed in the direction of greatest remaining variability until only noise remains (26). Each data point is then projected into the resulting low-dimension linear space (27). PCA was here performed only on animals with no recorded health in order to prevent any anomalous queuing behaviors recorded from acutely or chronically ill animals from obscuring the queuing patterns of the broader herd. The correlation matrix was constructed using all pairwise complete observations, and a scree plot was used to determine the dimensionality of the resulting space (see Supplemental Materials). The *plotly* package (28) was then used to visualize the final embedding.

While PCA provides a computationally expedient means of visualizing high dimensional data, the underlying assumption of linearity is not always appropriate (26, 27). In some data sets complex geometric constraints, such as those commonly found with images or raw accelerometer data, and other latent deterministic features may project data points onto high dimensional geometric surfaces collectively called manifolds (29, 30). When these topologies are non-linear (cones, spheres, donuts, etc.), the spatial relationships between data points cannot always be reliably maintained when projected directly into a linear (Euclidean) space, which can lead to incorrect inferences (27, 31). Imagine, for example, you had a round globe of the world and wanted instead a flat map. Applying PCA to this task would be analogous to smooshing the globe flat on a table. Some of the original geographic relationships would be discernable, but some locations would appear erroneously close, and some landscapes would be entirely obscured. Modern manifold learning algorithms strive to more reliably project the complex geometric relationships between observational units into a standard Euclidean space by approximating the surface of a non-linear manifold with a series of interconnected flat surfaces that can then be “unwrapped” onto a linear space (32). Returning to the previous pedagogical metaphor, this would be analogous to taking pictures of the globe centered around a number of key geographic locations, and then attempting to arrange the overlapping images onto a flat table. Some geographic features will still be lost, particularly over sparsely sampled regions like the oceans, but the spatial relationships between landmarks would collectively prove more representative of the original topography.

To further explore the underlying structure of this data absent assumptions of linearity, and thereby potentially accommodate any complex geometric constraints imposed on milk order records by latent social structures within the herd, a diffusion map algorithm was implemented using functions provided in base R (22). This was done here by first calculating the Euclidean distance between temporally aligned vectors of parlor entry quantiles for each pairwise combination of cows, scaled to adjust for missing records, and then inverting these values to create a similarity matrix. From this similarity matrix a weighted network was created by progressively adding links for the k = 10 nearest neighbors surrounding each data point. A spectral value decomposition was then performed on the corresponding graph Laplacian matrix (27, 33). The resulting eigenvalues were used to select the appropriate number of dimensions, and the corresponding eigenvectors visualized using the 3D scatter tools from the *plotly* package (28). Finally, as a means of comparing geometric structures identified in the observed dataset with those of a completely randomized queuing process, the permutated dataset generated in the previous section was also embedded and visualized using *plotly* graphics (28).

### Characterization of Temporal Dynamics

Having determined from the previous visualizations that a linear model might be a reasonable representation of the underlying deterministic structures of this system, the next step was to explore the temporal dynamics of this dataset. In a standard repeated measures model, multiple observations from the same animal are assumed to be identically and independently sampled, implying that sampling order should not affect the observed value. If the observation period is sufficiently long to allow the underlying process to shift or evolve over time, however, stationarity cannot be assumed. Failure to statistically accommodate a temporal trend can not only lead to spurious inferences due to incorrect estimation of error variance, but also risks overlooking dynamic features of the behaviors under consideration (34). In practice temporal trends are often assessed by first fitting a stationary model and analyzing the resulting residuals. This may suffice when the temporal trend is uniform across animals, but risks overlooking more complex non-homogeneous temporal affects. This could occur if only a subset of the larger group displays a non-stationary pattern, a risk that is likely heightened in large socially heterogeneous groups. In this physically constrained system, where we know that every cow moving forwards in the queue must force other cows backwards, compensatory trends could also be easily overlooked in collective assessment of residuals.

We first assessed temporal trend using two conventional EDA techniques. First, the *ggplot2* package (24) was used to generate scatter plots of entry quantile values against the corresponding observation date for each individual cow, with pasture access annotated with a vertical line. Plots were visually inspected for non-stationary, and are provided in Supplemental Materials. Next, to further explore the impact of the shift from pen to overnight pasture access on morning queueing patterns, median queue positions from the two subperiods were plotted against using the *ggplot2* package (24), and Pearson correlation (R) and Kendall Tau (τ) were computed using the *stats* package (22). While these preliminary visualizations were easy to both generate and interpret, both treat cows as independent and somewhat isolated units. With such a large number of animals to consider, the capacity for human pattern detection is quickly overwhelmed, making it difficult to contextualize trends within the broader herd. Further, this approach fails to leverage non-independence between animals entering the parlor, and thus risks overlooking subtler collective responses.

Data mechanics visualizations were implemented to simultaneously explore systematic heterogeneity in milk entry quantiles both between animals and across the temporal axis. This was done by first using entry quantile values to compute two Euclidean distance matrices: one quantifying the similarity between pairwise combinations of cows, the second quantifying similarity between pairwise combinations of daily milking sequences. These distance matrices were then used to generate two independent hierarchical clustering trees using the Ward D2 method (22, 26). By cutting both trees at a fixed number of clusters, observation days and cows were both partitioned into empirically defined categories, and a contingency table was then formed with cow clusters as the row variable and day clusters as the column variable. The original distance matrices were then updated, using the clustering structure between cows to create a weighted distance matrix between days and vice versa, thereby allowing mutual information to be shared between the temporal and social axes of the dataset (see Supplemental Materials for details). After several iterations of this algorithm, clusterings converged toward a contingency table with minimal entropy, wherein the entry quantile values within each cell were as homogenous as possible. When the entry quantile values were subsequently visualized using a heat map, this highly generalizable entropy minimization technique served to visually enhance heterogeneity within the data driven by non-random patterns along either axis. Further, by facilitating the transfer of information between axes, interaction effects between the social and temporal dimensions of this system were magnified, which here provided a means to explore non-homogeneous temporal non-stationary between subgroups within the herd (35–37).

The data mechanics pipeline was used to analyze the temporal dynamics present in both the complete milking order dataset and the subset of animals with no recorded health events. Heat map visualizations were generated using the *pheatmap* package in R (38), with observation days arranged on the column axis and Cow ID's arranged on the row axis. Fixed values for the number of clusters used to divide the row and column axes could not be determined *a priori*. Instead this algorithm was applied on a grid from 1 to 10 clusters for either axis. The resulting 100 heat maps scanned visually to determine the clustering granularity required to bring into resolution any interactions between social and temporal mechanisms. While this process may be computationally cumbersome, it is empirically analogous to systematically varying the focus of a light microscope to bring into resolution microbes of unknown size—a tedious but effective means of identifying all relevant structures within a sample (35). Finally, the *RColorBrewer* package (39) was used to add color annotations to the column margin, to clarify temporal patterns, and to the row margins, which served to visualize potential relationships between queue position, a selection of individual cow attribute variables, and the onset of recorded health complications.

### Linear Analysis of Cow Attributes

Having thoroughly characterized the stochastic structures present in this dataset, the insights gleaned from the preceding visualizations were incorporated into a linear model to evaluate the relationship between queue position and several cow attributes. The 4 days identified as outliers by the data mechanics visualizations were first removed and the dataset converted to long format to be analyzed as a repeated measures model using the *nlme* package (40). Cow was fit as a random intercept via maximum likelihood method. Guided by the results of entropy and data mechanics visualizations, VarIdent was used to estimate separate error variance terms for each cow, and the necessity of this data-hungry heterogeneous variance model confirmed via likelihood ratio test against the null model with homogenous variance (34). After centering and scaling cow attribute variables, linear fixed effects were added for cow age (days old at start of trial), calving date (approximately the date of entry into the herd), and peak milk yield (estimated via the 95th quantile of each cow's 150 day parlor lactation record). Interaction effects were created for each combination of these linear terms, and a categorical effect added for the control and treatment groups of the fat supplementation trial. Models were generated for both the complete dataset and the subset of animals with no recorded health events, which consisted of 160 and 104 cows, respectively after removing animals with incomplete attribute records. The predictive value of each fixed effect term was evaluated via a Wald's test. Where a significant association was identified at the standard α = 0.05 (Type I Error) confidence level, this pattern was visualized by plotting the cow attribute variable against the predicted queue position for each cow (fixed effect + BLUP).

While UML insights served to improve the specification of model variance structures within-animal, the validity of statistical insights made at the between-animal level is still contingent upon the correct estimation of model degrees of freedom. A fundamental assumption of frequentist tests is that observations must be independently sampled. When observations are not independent, the effective degrees of freedom present in the model may be lower than the nominal value. This causes the model to be overconfident in its estimation of error terms, increasing the risk of a false positive result. Non-independence due to repeated sampling (pseudoreplication) has here been accounted for by fitting a random effect for each cow, but non-independence between animals has not been accommodated. The results of the diffusion map and data mechanics visualizations did not recover overwhelming evidence of coordinated movements between animals through the queue, which would have signified non-independence due to social cohesion (positive interclass correlation between animals); however, we both visualized via data mechanics and know intuitively that in this physically constrained system any cow moving forward in the queue must be countered with other cows being forced backwards and vice versa. If this effect extends beyond isolated fluctuations in daily formation of the queue, then the presence of some animals in the herd might systematically dampen or even completely prevent other animals from demonstrating behavioral patterns that they would otherwise display independently or in another herd with a different social composition (negative interclass correlation between animals). This would not only serve to confound the behavioral mechanisms at play, but such cows whose behaviors are suppressed by their herd mates cannot be said to be contributing fully to the model, potentially reducing the effective sample size. This could allow sampling fluctuations to produce misleading statistical inferences, even in this large sample of animals (41–43).

UML algorithms cannot recover information about behaviors that were never expressed, and so are also not immune to the biasing effects of non-independence between animals. These tools can, however, provide model-free tests of association that may serve as a sanity check for statistical inferences when degrees of freedom may be uncertain. We explore this option here by again combining modern clustering tools with a flexible information theoretic approach to pattern detection (35). First, independent clustering tress were used to subdivide the herd based on queuing records and each of the cow attributed variable. The resulting categorical variables were then used to form contingency tables between queue subgroups and each of the candidate predictor variables. If no relationship existed between these two axes, then a cow belonging to a given row category based on queue records would be just as likely to belong to any of the column categories based on cow attribute and vice versa. If instead an underlying biological mechanism was present linking these axes, then cows within a range of cow attribute values would be spread unevenly among queue subgroups. Such heterogeneity in cell counts was quantified by calculating a weighted mutual conditional entropy (MCE) value across first the rows and then the columns of the contingency table and averaging the results, which reflected the amount of mutual information shared between the two variables. To determine if the observed MCE value was significantly smaller than would be expected from random fluctuations in the sample, row and column classifiers were randomly permuted across cows to remove any underlying bivariate relationship and MCE recalculated. This randomization procedure was repeated over 2,000 iterations, and the observed entropy value compared to the resulting empirical CDF to produce a *p*-value for the significance of the bivariate association. Mutual conditional entropy tests were performed for all significant or marginally significant linear effects for both regression models. While the number of clusters used to discretize the cow attribute and queue records may be specified *a priori* provided strong biological reasoning or empirical evidence, mutual conditional entropy tests were here preformed on a grid from two clusters up to the highest visible granularity of the corresponding clustering tree, and the optimal metaparameter values selected by minimizing the average marginal rank.

### Exploring Associations Between Sensor and Queue Records

Previous studies seeking to identify factors that predict an animal's parlor entry position have focused primarily on biological drivers of queueing behavior related to productivity, health, and traditional measures of fitness such as age and size (44). As this herd was also fitted with ear tag accelerometers, it is here also possible to explore relationships between queue position and behavioral patterns displayed between milkings. Due to the size of these datasets, however, this small step beyond the bounds of the existing literature constitutes a considerable leap in statistical complexity within a linear modeling framework. A multivariate mixed model that considers all observations from either dataset would exceed the capacity of many solvers (45). A simpler approach to exploring this relationship might therefore be to compress the information available in parlor entry records into a grouping variable and then attempt to identify differences in the various home pen behaviors across the resulting subsections of the herd.

We implement this strategy here by using the *nlme* package to fit linear mixed models, with cow fit as a random intercept, against each of the five behaviors recorded by the CowManager platform (non-activity, activity, high activity, rumination, eating) and also average body temperature (40). To avoid the risk of anomalous behaviors that might skew model inferences, only cows with no recorded health events were used. Hour of the day was fit as a categorical variable to capture cyclical patterns. Days on trial was also fit as a categorical fixed effect to allow for non-smooth longitudinal changes in behaviors due weather and also the shift to pasture. Finally, queue groups were determined by arbitrarily dividing the herd into quartiles based on median entry position. The resulting categorical variable was then fit as both a main effect and an interaction effect against both cyclic and longitudinal time effects. Due to the size of the model, temporal correlation and heterogeneous variance models both exceeded the capacity of this package to converge. Comparisons of the cyclic and longitudinal trends in behavioral patterns between queue groups were made using the plotting utility available in the *emmeans* package (46), with the complete results provided in the Supplemental Materials.

While linear models provide an expedient means to statistically evaluate targeted experimental hypotheses, the more open-ended approach to knowledge discovery provided by UML algorithms may offer an advantage in exploratory data analysis problems such as this. We explore the utility of this alternative strategy here by again employing a mutual conditional entropy (MCE) test to identify significant associations between these two behavioral axes (35). The flexibility of hierarchical clustering tools allows this technique to be directly extended from the previous section, which compared repeated measures of queue position against a univariate covariate, to accommodate both high dimensional datasets. For each parameter recorded by the CowManager platform, this model free test of association was performed on the complete sensor record, on subsets of the records corresponding to each of the three lounging periods (morning, afternoon, and night), and finally on a subset of the records where observations from all three lounging periods had been aggregated. As in the previous section, the number of clusters used to discretize queue and sensor data were evaluated on a grid, here from tree depths 2–10. To characterize the divergent behavioral patterns across queue groups identified by significant tests of association, tube plots were created by plotting each within-day subgroup median on a circular grid and then stacking rings to form a tube using the 3D plotting tools in the *plotly* package (28).

## Results and Discussion

### Quantifying Degree of Randomness

**Figure 1:**
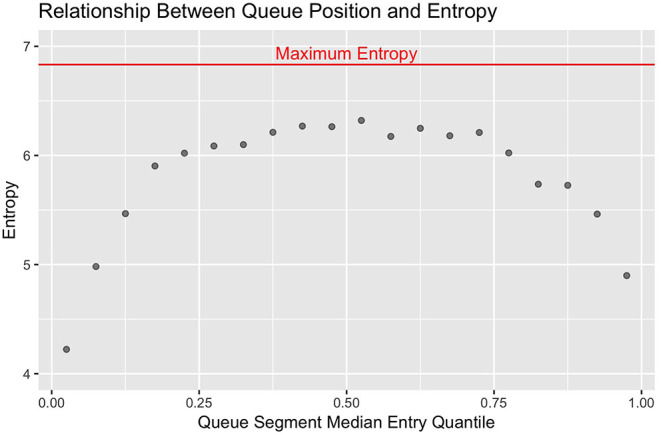
Entropy estimates from observed (red) and randomly permuted (blue) datasets are plotted against the median quantile value for the corresponding segment of the queue. The front and end of the queue are the least stochastic, but all sections of the queue demonstrate lower entropy than with purely random queue formation.

**Figure 2:**
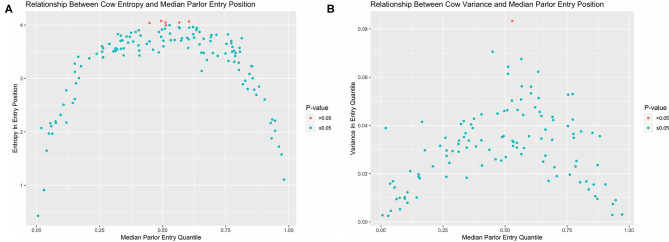
Observed entropy **(A)** and variance **(B)** values for each cow plotted against their corresponding median entry position. Cows with the greatest consistency in parlor entry position are found at the front and end of the queue. With both estimators, nearly all cows in the herd demonstrate less stochasticity in parlor entry position than completely random queue formation as determined by permutation test.

Looking first at the entropy calculations for each segment of the queue visualized in Figure 1, it is clear that all parlor entry positions are not stochastically equivalent. The same animals are seen consistently at the very front and back of the queue, such that the resulting entropy values are far lower than would be seen with a purely random queueing process. Moving toward the middle of the queue, however, there is progressively less consistency in the animals present across milkings, such that the observed entropy values approach a random process. Looking next at the stochasticity demonstrated by each individual cow in Figure 2, we see there is again a clear gradient. Cows with median entry quantiles at the front and rear of the herd again show far greater consistency in their entry positions. As their median quantile position moves toward the center of the herd they become more variable in their entry positions over the observation window. This gradient is seen using both entropy and variance as estimators of stochasticity, but is more visually distinct using entropy estimates. While discretizing an intrinsically continuous parameter results in a loss of information, we see here that this sacrifice has excluded extraneous noise in the system to bring the underlying stochastic pattern into clearer resolution. This data thus highlights the potential upside of amending entropy estimates to the traditional cadre of summary statistics, particularly when working with outcome variables that are prone to extreme or anomalous values.

In examining the results of the permutation tests, nearly all animals demonstrated significantly less stochasticity in their entry positions at the standard α = 0.05 significance level as compared with a completely randomized queueing process. Only 3 cows out of 114 overlapped with the empirical distribution of entropy estimates under a randomized queueing pattern, and only 1 cow overlapped when variance was used as the estimator of stochasticity. This suggests that nearly all animals in the herd might contribute some information about the underlying non-random patterns in queue formation to subsequent analyses; however, the amount of information they contribute may not be equal, as there is considerable heterogeneity between cows. Of greater concern, this heterogeneity is systematic, as there are no cows showing high consistency in entry quantile in the center of the queue. If this pattern is not driven by variability in the underlying predictors of queue position, but instead reflects either an underlying behavioral mechanism or something even more fundamental to this system such as the inherent domain constraint (18), this could lead to inaccurate statistical inferences. To avoid such risks, these simple visualizations provide clear evidence that a non-trivial variance model should be incorporated into the model specification phase to accommodate the heterogeneous variance structures in this dataset.

Finally, the insights gleaned from these entropy-based visualization techniques agree well with the prior literature. Previous studies have repeatedly determined milk order records to be significantly more consistent than would be expected from a random queuing process using an array of correlation and regression-based approaches (10, 12, 16–18, 47). Fewer papers, however, have explored differences in the consistency of entry positions between animals. Gadbury (13) observed that only a subset of his herd seemed to demonstrate clear preferences for parlor entry positions. Such preferences do not appear to have been constrained to the front or back of the queue, however, as Gadbury (13) also reported animals with a preference for the middle of the queue. In a more recent analysis with large commercial herds, however, Beggs et al. (18) reported a nearly identical parabolic relationship between mean entry quantile and variance. With clear and consistent evidence of non-random patterns having been recovered from this dataset, further investigation of the behavioral mechanisms that might give rise to such heterogeneity in milk order records was clearly warranted.

### Visualization of Inter-animal Relationships

**Figure 3:**
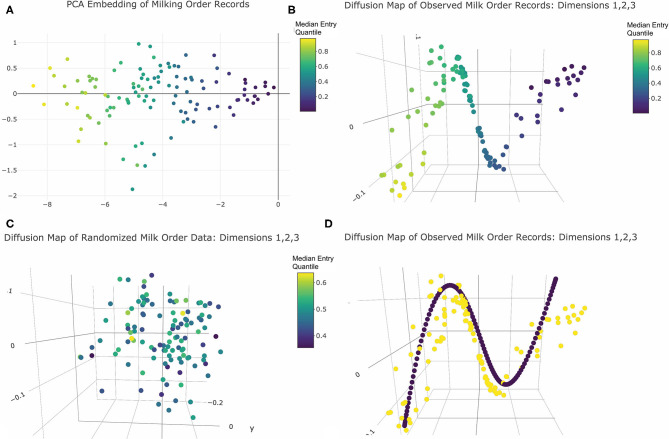
Dimension reduction of milk order records colored by median entry quantile value: **(A)** PCA embedding returns one principal axis reflecting each cows central moment of entry position. **(B)** The first three significant axes of diffusion map embeddings reveals a clearly linear underlying geometry reflecting central moment. **(C)** Diffusion map embeddding of data from the simulation of purely random queue formation recovers no distinctive geometry. **(D)** Harmonic series (purple points) imposed over observed cow records (yellow points) reveal curvilinear geometry is a embeding artifact. Note: Interactive versions of 3D plots are availble in Supplemental Materials.

Visual inspection of the scree plot produced from PCA analysis revealed only one significant dimension was recovered from the original 80-dimensional dataset. To visualize the resulting projections, the first two principal components were plotted (Figure 3A). Cows appeared evenly spaced along the first principal axis with no clear gaps between observations. In two dimensions points also appeared randomly scattered with no clear clustering. Thus, the PCA results revealed no compelling visual evidence of social cohesion. The color encoding further revealed that the first principal component conveyed information about the center of each cow's entry quantile observations. As this was the only significant dimension, this may suggest that a linear model to predict variations in central moment would be a reasonable representation of this dataset. This feature of the dataset was not, however, self-evident in the geometric relationships between data points revealed by the PCA projection, and thus might have been overlooked without specification of color encoding by median entry quantile value *a priori*.

Evaluation of eigenvalues returned by the diffusion map embedding identified five significant dimensions. The 3D visualizations of these axes in Figure 3B and provided in Supplemental Materials revealed quite clearly the underlying linear geometry of this dataset. Color encodings showed that the relative positions of animals along this narrow geometric band were determined by median entry quantile, further reinforcing that central moment was the most defining feature of this dataset. As with the PCA results, cows appeared fairly evenly spread along this linear object, with no clear clustering to suggest social cohesion amongst large or temporally persistent subgroups. Comparing these results with the embedding of the permutated queue records (Figure 3C), no clear geometric features were recovered from data simulated from a purely random queueing strategy. This reinforced that the linearity of the observed records was not simply an artifact of the physical linearization of cows as they enter the parlor single-file, but a reflection of a consistent pattern in queue formation that might be driven by some underlying behavioral or biological mechanism.

While the diffusion map embeddings convey a clear linear geometry, there was also unexplained curvature in the band along which cows were projected. This proved not to be an inherent feature of the data itself but a harmonic artifact imposed by the spectral value decomposition of the graph Laplacian used to deduce the shape of the underlying network between cows (48). Such a mathematical operation has several physical interpretations. One is that an singular value decomposition (SVD) of the Laplacian is akin to walking around an object in the dark and striking with a mallet at many points across its surface so that the quality of the resulting sounds can be used to discern its shape (49). The linear geometry of this dataset forms a “rope-like” network (48). When the SVD decomposition “strikes” such a network to assess the quality of sound produced, it responds like a plucked guitar string. As a result, each axis of the subsequent embedding contains an element of the harmonic series, producing the curvature seen in these milk order visualizations. Fortunately, this artifact can be described by closed form equations (48) and imposed onto the data to aid in discerning authentic geometric features of the data (Figure 3D). Thus, while diffusion map did provide a clearer geometric representation of the inherent linearity of this dataset than PCA, this dataset al.so reinforces that modern manifold learning techniques are also not infallible in recovering the underlying geometry of high dimensional data. While such embedding techniques may provide helpful insights into the underlying structure of large datasets, a conservative approach to visual interpretation of such results is still warranted.

### Characterization of Temporal Dynamics

Independent visualization of parlor entry records from each individual cow (see Supplemental Materials) revealed that the majority of animals in this sample were surprisingly stationary in their queueing position. Animals that frequented the front and end of the queue, being more consistent in their entry position, provided clearer visual evidence for a lack of temporal trend. Cows in the middle of the queue showed far greater variability in their entry positions, making it more difficult to visually discern temporal trend from stochastic fluctuations. Only two animals were identified as having a clearly visible trend: cow 13,467, who had no recorded health events, and cow 13,826, who was diagnosed with metritis during the enrollment phase early in the trial. Both cows showed similar trajectories, starting nearer the end of the herd and moving progressively forward toward the front, but neither change in queue position coincided with the shift to overnight pasture access.

**Figure 4:**
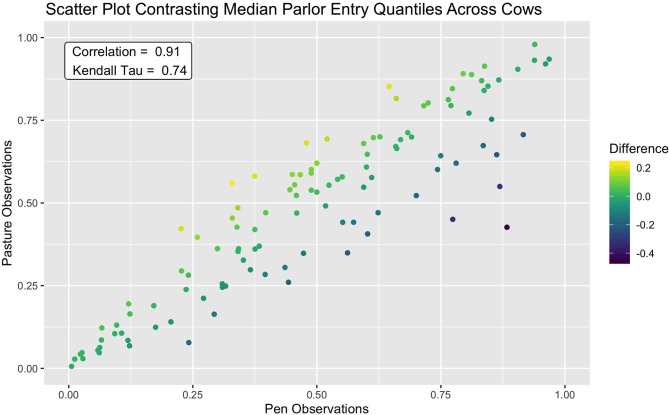
Scatter plot showing strong agreement of median parlor entry quantiles between the pen and pasture subperiods. A small portion of cows which demonstrated a larger shift in entry position moved forward in the queue after the shift to pasture from pen.

This consistency in queue position was further reflected in a clear linear association between median entry quantiles from overnight pen and pasture subperiods (see Figure 4). A slightly wider spread was discernable amongst cows occupying the middle ranks, but for the majority of animals, median entry quantile values did not change more than ±0.2. Among the handful of animals demonstrating a more extreme shift, these jumps tended to be in the forward direction toward the head of the queue. Overall, fewer extreme shifts were seen in this dataset than in a similar bivariate means plot provided in Beggs et al. (18), though this may simply be a reflection of the longer subperiods over which median entry positions were assessed. Correlations between these values were also quite high, with a Pearson correlation estimate of 0.91 (*p* < 2.2e-16) and a Kendal Tau estimate of 0.74 (*p* < 2.2e-16). These values are, as expected, higher than the estimates of consistency reported for individual milk order samples (17, 19), but on par with results using subperiod averages on similar time scales (12, 16, 18). Given the extreme shift in management routine spanning these two subperiods, however, this level of stability in parlor entry positions was an unexpected result. Such resilience to changes in overnight housing environment and the subsequent distance traversed to access the parlor could suggest that milking order is largely determined in the crowd pen, a result supported by early observations by Soffie et al. (12), who reported little correlation between the order of cows exiting the home pen and entering the parlor past the first few animals.

**Figure 5:**
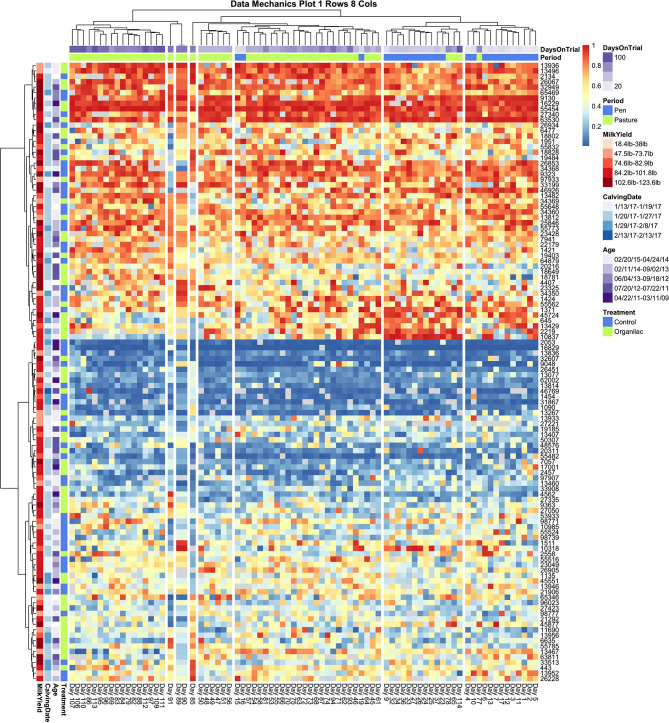
Data Mechanics visualization of cows with no recorded health events. Clustering along the temporal axis has isolated 4 days of milk order observations in middle of the pasture subperiod with anomalous queuing patterns, which can be viewed as irregularities in color values scanning from left to right. These days remain isolated in Data Mechanics mappings which also allow for social stratification along the row axis (see Supplemental Materials), suggesting that these observations likely constitute outliers. Color annotations along the column axis reveal observations from the pen and pasture subperiods remain fairly distinct. No clear patterns or gradients are seen on the row color annotations for cow attributes, even though the heat map itself clearly reflects a gradient along the column axis driven by individual differences in queue entry position. Progressive clustering of the row axis did not bring any additional patterns in cow attribute variables into clear resolution.

Collective assessment of entry quantile records using data mechanics visualizations did, however, reveal additional temporal features not identified using independent visualizations of cow records or collective assessment of aggregate records. The first and perhaps must surprising insight was that, with finer granularity in number clusters applied to the temporal (row) axis, data mechanics identified several days with anomalous queuing patterns. In Figure 5, a total of 8 column clusters are imposed without any social stratification on the subset of cows with no health events. If these records were completely stationary with no temporal effects, we would expect days to be randomly partitioned into these eight categories. Instead 4 days are isolated from the remaining observations. Days 85 and 91 are separated into clusters of size *n* = 1, and 89 and 91 are also isolated into their own cluster of size n = 2. Looking from left to right along the heat map to identify temporal heterogeneity, it is easy to see that on these observation days animals typically occupying the extremes of the queue appear to have been pushed toward the center and animals typically found in the center of the herd were either pushed toward the extremes or inverted their tendency to stay toward the front or end of this middle section of the queue. While some of the entry quantile values encompassed by these observation days would likely be identified as outliers for individual cows, other values would likely be deemed irregular but not worthy of exclusion. These clustering results, on the other hand, suggest that either transient environmental or internal social factors have disrupted the entire herd and caused them to collectively respond with highly irregular queuing patterns. As the row axis is stratified to allow for non-homogeneous temporal responses across subsets of animals, these same days are consistently isolated from the remainder of the dataset, reinforcing that these observations constitute an outlier that should be excluded from any downstream analyses.

**Figure 6:**
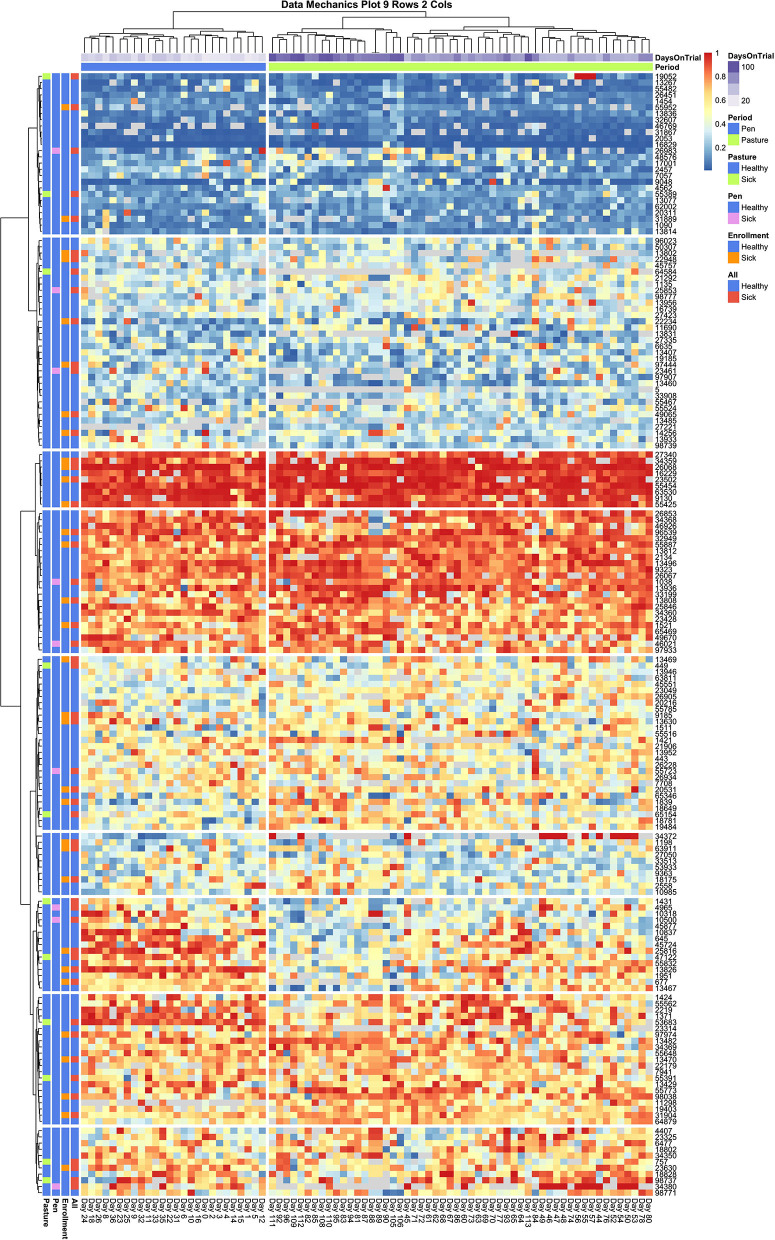
Data Mechanics visualization of all cows. Color annotations of the column axis reveal that coarser clustering along the temporal axis has revealed a perfect division of observations between pen and pasture subperiods. Scanning from left to right within the heat map, cows occupying the front and rear of the queue appear consistent in entry quantile values. Cows in the center of the herd appear to be the source of this temporal heterogeneity, as evidence by systematic changes in color along the column axis. In the cluster of animals starting with cow 1431, there is a progressive shift from the rear of the herd in the pen period toward the front of the herd in the pasture. As row color annotations reveal not all these animals have recorded health events on record, this pattern likely cannot be explained away by anomalous behaviors from acute or chronic illness.

Looking next at the coarser stratifications of the temporal axis, we also see that pen and pasture observations are not equally dispersed among the column clusters. As the animal (row) axis is more finely stratified to allow for social heterogeneity within the herd, the source of the temporal heterogeneity between these two environments comes into resolution. In Figure 6, which contains entry quantile observations on both sick and healthy animals, pen and pasture observations are perfectly stratified across only two column clusters. Looking at the subsets of animals who consistently entered at the front and rear of the herd, entry quantile values appear quite homogenous in color between the two temporal clusters. Scanning from left to right among the subgroups of animals that frequented the center of the queue, on the other hand, systematic fluctuations in daily entry quantile values can be seen even without finer temporal stratification. This pattern is clearest in the cluster which contains both cow 13,467 and cow 13,826—the two animals identified by independent inspection of cow entry quantile plots to show evidence of non-stationarity. In this subgroup, cows showed a tendency to frequent the latter half of the queue when coming to the milking parlor from the home pen, but during the pasture period showed progressively greater proclivity to enter in the front half of the queue. Where this shift is the most uniform in the latter half of the pasture period, we also see a compensatory pattern in the subgroup directly above, where cows shifted from nearer the front to the back half of the queue. Whether these results reflect the coordinated movement of relatively small social subgroups or just a common response to environmental conditions is impossible to say from this data alone. These results do make it clear, however, that not only are the cows occupying the center of the queue less consistently in their entry position, they are also less stable in their entry pattern. Further, these visualizations underscore that these divergent dynamics in the pen and pasture subperiods cannot be captured by a simple fixed effect term. The simplest option would be to drop from the analysis the animals that show the strongest non-stationary patterns. With such a large group, this would still leave ample observations to maintain statistical power, but could risk biasing the subsequent inferences. Alternatively, by specifying a heterogeneous variance model between animals, as was deemed necessary in the original entropy plots, the influence of these cows on the fitted model may be reduced sufficiently that deviations from the assumption of stationarity in this subgroup might not unduly destabilize the final model.

Finally, some preliminary insights can be gleaned from the cow attributes added to the row margins of both heat maps. In Figure 6, animals with documented health events appear fairly evenly dispersed across subsections of the queue. A slightly lower rate of illness might be attributed to animals that consistently occupied the very front of the queue, and perhaps a marginally higher rate of transition diseases was seen in the animals at the very rear of the queue, but these patterns appear subtle at best and thus likely not the only determinant of queue position. This result was somewhat surprising, as previous research has suggested that sick animals tend to populate the rear of the queue (11, 16, 17, 19). If this previously reported trend is driven by a reluctance among animals in the acute phases of a disease to move, it is possible that the daily health checks prescribed in this experimental trial succeeded in identifying and removing sick animals from the herd sufficiently early that this behavioral mechanism was not at play in this dataset. This might suggest that inclusion of these additional animals into subsequent analyses might not unduly bias subsequent behavioral inferences. Of perhaps greater concern to subsequent modeling is the lack of clear color gradients among cows attribute values across the queue, which could indicate that underlying associations may either be weak or that there are complex interaction effects creating a non-uniform trend.

### Linear Analysis of Cow Attributes

For both the full dataset and the subset of healthy animals, likelihood ratio tests revealed the heterogeneous variance model allowing for differing degrees of variability in queue position across cows to be a costly but necessary model component (*p* < 0.0001). With the model fit to cows with no recorded health events, significant linear associations were recovered for two fixed effects. Cows with higher peak milk yields demonstrated a tendency to enter nearer the rear of the queue ($\hat{\text{B }}=0.14,\;F_{1,96}=9.58,\;p=0.003$). A significant interaction term revealed this trend was further amplified for older cows ($\hat{\text{B }}=0.07,\;F_{1,96}=6.11,\;p=0.015$). No other terms approached significance for this dataset. With the model fit to all cows that attended at least 50% of recorded milkings, no predictors were significant at the α = 0.05 cutoff. Peak yield remained marginally significant ($\hat{\text{B }}=0.06,\;F_{1,152}=2.93,\;p=0.089$), as did the interaction term between peak yield and cow age ($\hat{\text{B }}=0.04,\;F_{1,152}=2.92,\;p=0.090$). With this larger dataset, however, cow age also demonstrated a marginally significant trend, indicating older cows tended to be nearer the front of the queue ($\hat{\text{B }}=-0.07,\;F_{1,152}=3.84,\;p=\;0.052$).

In contrasting the results of these two models, the loss of significant association between entry quantile values and peak yield with the addition of sick animals is perhaps not surprising. If a disease challenge early in the trial curtailed peak lactation in these cows but did not cause chronically deficient production, then the 95th quantile value of milk yield used here to estimate peak lactation level may not adequately reflected the overall productivity of these animals across the duration of this extended trial, obscuring the underlying biological mechanism. The emergence of a nearly significant association between entry quantile and age with the addition sick animals, however, is more difficult to explain. Given that peak yield and age are highly correlated biological parameters (*r*_*all*_ = 0.66, *r*_*healthy*_ = 0.70), this sample may simply contain too few older cows with low productivity levels by which to disentangle the positive association with peak yield from the negative association with cow age. Alternatively, if a diseased state permanently alters a cow's queueing pattern and if risk of health complications in turn varies with age, then health status may be a lurking variable masquerading as an age effect. In either case, a relatively small number of animals may be unduly influencing statistical inferences.

**Figure 7:**
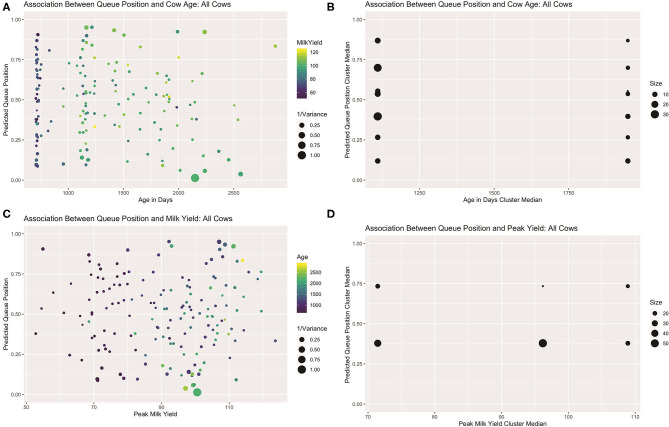
Visualizations for all cows that attended >50% of observed milkings. Scatter plots of predicted queue position against cow age **(A)** and peak yield **(B)**, scaled for heterogeneity in repeated samples of entry quantiles. For both attributes, linear trends are only readily discerned among multiparous animals. Sparsity in parity 3+ animals might skew the calculation of age effect as a continuous variable. **(B)** Visualization of contingency table results derived from independent clusterings used in MCE test of bivariate association. Age effect is lost with empirical discretization **(C)**. A non-linear trend is recovered with peak yield **(D)**.

Visual examination of predicted queue positions plotted against age and peak yield for both the full dataset (see Figure 7) and healthy subset (see Supplemental Materials) seem to confirm these misgivings. Looking first at age, the first lactation heifers, being evenly spread across the center of the queue, cannot be driving this linear effect. Among the multiparous animals, the five cows seen consistently in the front of the queue are indeed among the oldest in the herd, but if this handful of animals and their corresponding queue positions are ignored, a clear gradient is not visible among the remaining cows. Results of the mutual conditional entropy tests confirm this suspicion. For the disease free subset the MCE test confirms the insignificant association found in the linear model (*p*_2,2_ = 0.103). For the full dataset, where the linear effect is marginally significant, the MCE test does not (*p*_7,2_ = 0.305). This suggests that either that age effect is only discernable after adjusting for peak yield or that the association is not robust.

Looking next at peak yield, a clear global trend could not be discerned. Among the lower-yielding cows, a group comprised predominantly of heifers, a linear trend is difficult to discern, but amongst older cows a slight positive gradient is perhaps perceptible. Results of the mutual conditional entropy tests not only confirmed this trend among the healthy animals(*p*_3,5_ = 0.036), but also within the full dataset (*p*_2,3_ = 0.012) where the linear effect was only suggestive. Visualization of the contingency table for this later result revealed no distinctive pattern among the lowest and highest yielding clusters, but a nearly perfect division of roughly 50 moderate-yielding cows into the leading queue cluster. This result suggests that the MCE tests may also be used in mixed modeling analyses to recover non-random patterns that are not well-represented by linear trends. Such a non-linear trend here could belie more complex interaction effects between these or other unmeasured biological drivers of queue position. Alternatively, a multilevel model may be necessary to disentangle complex hierarchical relationships between the drivers of position preference and a cow's ability to assert that preference.

Contextualizing these results within the existing base of literature underscores the inconsistency in drivers of queuing behaviors. With respect to milk yield, several studies have found no significant association (12, 16), but among those that have, most have reported high yielding cows frequent the front of the queue. Differences in motivation to obtain feed might explain this result. In early studies, cows were offered concentrate in the milking parlor, which may have increased the motivation of high yielding animals with greater energy deficiencies to enter the parlor (11, 13). In more recent work, cows may have been motivated to access limited feed bunk space on commercial dairies (19) or to obtain prime pasture (50). In this study, as all animals were locked following milking to facilitate feeding treatments and health checks, cows would have had ample access silage regardless of queue position. Alternatively, Rathore (11) suggested greater intermammary pressure might motivate high yielding animals to be milked earlier. As this herd was milked three times daily, however, this biological driver may also have been attenuated. Indeed, among modern studies with herds milked thrice daily, Polikarpus et al. (16) found no significant correlation and Grasso et al. (17) also found high yielding cows frequented the rear of the queue. Ultimately, as yield is influenced by a wide range of health and management factors, any number of confounding variables might be implicated in explaining this somewhat unexpected result. In this study a significant linear association between age and entry position was not found. Recent work by Berry et al. (19), which identified a non-linear trend across parity, and by Grasso et al. (17), which highlighting significant interactions of parity with other biological drivers of queue position, suggests that a linear effect may not adequately capture the underlying biological relationship. A larger and more structured sample may be necessary to bring more complex age dynamics into clearer resolution.

### Exploring Associations Between Sensor and Queue Records

Visual inspection of means plots produced from mixed model analysis of sensor records recovered only a handful of statistically significant differences between queue quartiles when hour and day effects were assessed individually, but several global trends were still readily visible. With respect to minutes recorded as active, the 1st−3rd queue quartiles were visually indistinguishable in their cyclical behavioral patterns, but cows in the fourth queue quartile were consistently more active, particularly during the night and morning lounging period. With respect to longitudinal trends across days, fourth queue quartile animals were again more active across the observation window, whereas cows in the first queue quartile were consistently the least active. These patterns were somewhat mirrored in the longitudinal and cyclical analysis of high activity minutes, but the pattern was both less distinct and less consistent. No clear qualitative insights could be drawn for cyclical or longitudinal patterns in non-activity. Cyclical patterns in minutes spent eating were not seen overnight or in the afternoon, but first queue quantile cows may have spent slightly more time eating after the morning milking. Longitudinal analysis of eating patterns suggested cows in the fourth queue quartile spent relatively less time eating, whereas the cows in the first and second queue quartile consistently spent more time at the bunk. This contrasted with longitudinal results for minutes spent ruminating, where the cows in the second queue quartile were consistently low. No clear distinctions between groups were recovered in cyclical rumination patterns. Temperature patterns were, surprisingly, the most visually distinct of all the sensor parameters. Cows in the first queue quartile were consistently lower in body temperature in both the longitudinal and cyclical time dimensions as compared with the remainder of the herd.

While the preceding analyses revealed few statistically significant differences at individual time points, collective analysis of days and subsets of the 24 h management cycle would uundoubtedly return statistically significant differences for the broader qualitative trends visually identified via mean plots. Within a linear modeling framework, however, this constitutes no small task. For all of the above models, Wald's tests revealed Group-by-Hour interactions effects to be highly significant components of the model (*p* < 0.0001). Group-by-date interaction effects were also significant for activity, high activity, and temperature models (*p* < 0.05). This suggests that these models should not be simplified to a single cyclical or longitudinal trend, which would allow overall differences between groups to be tested via a single group intercept term. Targeted hypotheses comparing comprehensive trends between groups would instead require formulation of linear contrasts—a daunting task with so many fixed effects terms used to accommodate the high sampling frequency and extended observation period of this dataset. Further, as with the linear models with cow attributes, behavioral synchronization due to social cohesion or compensatory use of physical resources in the pen could again create non-independence between animals in such sensor records. Any such issues in estimation of model degrees of freedom, compounded with the inability to fit behaviorally and empirically compelling correlation and variance models, would only serve to further confound the estimation of appropriate *p*-values from these models.

Fortunately, the qualitative trends identified via the preceding means plots largely aligned with the significant bivariate associations identified by mutual conditional entropy tests summarized in Table 2. Activity again proved to be the most distinctive behavioral axis. Significant associations were identified for all three lounging periods when analyzed both independently and in aggregate, with the afternoon lounging periods being the most distinct. High activity also showed a significant relation to queue records, but this association may have been driven predominantly by overnight lounging period. Whereas, no clear qualitative patterns were identified for non-activity data via the means plots, a significant association with queue records was identified during the afternoon lounging period. A highly significant relationship was identified for time spent eating for the full sensor record, but given that time budgets recorded by this platform were segmented somewhat arbitrarily at the start of each hour, this result may simply reflect a lag in the arrival of cows to the feed bunk after exiting the parlor. Significant associations were not found during the lounging periods at the standard α = 0.05 cutoff, though records from the afternoon lounging period approached significance. These results were mirrored in rumination patterns, where again no significant association was recovered, but the afternoon lounging period approached significance. Finally, as with the linear modeling results, temperature proved highly distinct between queue subgroups for all subperiods.

**Table 2:** *P*-values generated from mutual conditional entropy tests comparing queue records to sensor logs.

	**All**	**Lounging**	**Morning**	**Afternoon**	**Night**
Non-activity	<0.001_7,5_	0.048_2,5_	0.432_8,7_	0.002_2,5_	0.132_2,6_
Activity	<0.001_3,8_	0.006_2,8_	0.038_2,11_	<0.001_2,3_	0.033_2,9_
High Activity	0.052_2,9_	0.028_2,2_	0.272_10,9_	0.306_3,4_	0.014_2,4_
Eating	0.004_7,6_	0.234_7,5_	0.188_6,4_	0.066_2,4_	0.212_9,2_
Rumination	0.021_2,4_	0.05_9,3_	0.325_7,2_	0.083_10,3_	0.152_8,10_
Temperature	<0.001_5,10_	0.004_5,5_	0.022_7,3_	0.006_3,5_	0.015_5,3_

*Subscripts represent the number of clusters used to discretize the row variable (queue records) and column variable (sensor records). For example, in the test for morning non-activity, cows were assigned to 8 clusters using queue records and 7 clusters using sensor logs. Activity and temperature data demonstrated the strongest association with queue records. The afternoon lounging period produced the strongest associations between queue and all sensor dimensions save for high activity, which showed the strongest distinction overnight*.

**Figure 8:**
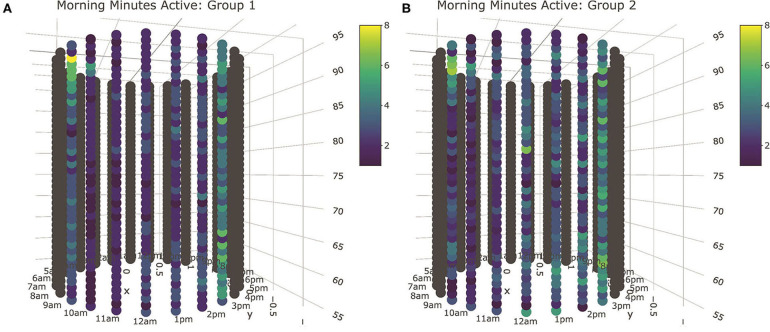
Tube plot visualizations for minutes spent active during the morning lounging period. Based on the results of the MCE tests, all behavioral axes cows were clustered into two subgroups based on queueing records, with Group 1 cows **(A)** consisting of 80 animals at the front of the queue, and Group 2 cows **(B)** constituting the 34 animals in the rear. Cyclical patterns can be seen around the diameter of the tube, and longitudinal patterns observed along its length. The median activity level of Group 2 animals, consisting of 34 animals in the rear of the queue, is heightened during the middle of the lounging period for the duration of the trial. Interactive 3D plots for all sensor output fields are provided in Supplemental Materials.

Visual inspection of tube plots produced with median queue subgroup values again yielded insights comparable to the linear modeling results (Figure 8). Based on the results of the MCE tests, all behavioral axes cows were clustered into two subgroups based on queueing records, with Group 1 cows consisting of 80 animals at the front of the queue, and Group 2 cows constituting the 34 animals in the rear. Tube plots of minutes spent active revealed Group 2 cows to be more active across all three lounging periods. This pattern was the most consistent in the morning and overnight lounging periods, though this difference was ultimately quite subtle and seldom constituted more than a few minutes. In the afternoon subperiod there was evidence of several periods with anomalously high activity levels, most of which occurred post-pasture access. The significant association recovered for minutes spent highly active in the overnight subperiod appeared to be largely driven by increased activity immediately following the evening milking, which could reflect divergent home pen behaviors, but might also have been driven by delays in milking. To complement these results for active and high active minutes, the significant association for afternoon non-activity records appears to have been driven by increased non-activity among the Group 1 cows during the 3 h immediately preceding the night milking. As anticipated, differences in time spent eating were largely restricted to the 2–3 h immediately following milking. Cows only lingered at the feed bunk during the morning lounging period, where median eating times for Group 1 cows were perhaps slightly higher. Similarly, differences in rumination also appeared restricted to time periods immediately following milking, with no clear differences seen during the lounging period with this coarse stratification of animals. Finally, as with the mean plots, body temperature values again produced surprisingly distinctive results. More finely segmented into five queue groups by the mutual conditional entropy test, the tube plots proved a slightly cumbersome means of comparing temperature records, but a clear visual distinction could still be made between the Group 2 animals and the remainder of the herd. For all three lounging periods, this relatively small cluster of 17 cows that constituted the very front of the milking queue demonstrated lower median body temperature values, a distinction seen most clearly at night.

The strong agreement between the results of these two analytical pipelines suggests that UML and conventional linear modeling approaches could be used interchangeably or in concert to glean preliminary insights from exploratory analyses of large sensor-based datasets that may inform future hypothesis-driven studies. Perhaps the most surprising result of these analyses, that cows frequenting the back of the queue are consistently more active between milkings, may indeed warrant further exploration. In much of the prior literature, health challenges that impede movement (lameness, subclinical mastitis, etc.) have been identified as the main driver of delayed entry into the parlor (13, 16, 17, 51). In fact, this mechanism is so well-established that it has even been proposed that milk order records might be incorporated into genetic evaluations to improve estimates of health traits (19). As these analyses were run on the subset of animals with no recorded health events, however, it is possible that this dataset has brought other behavioral mechanisms into focus.

One potential explanation for these results might be a dominance gradient. Previous studies have found that animals of low social status frequent the rear of the herd in voluntary movements (10, 52), and social dominance is known to impact resource access in spatially constricted conditions (53, 54) such as those found at the entrance to the milking parlor. If low dominance animals are in turn also forced to wait longer or walk farther to access resources in the home pen, this could potentially explain the increased activity levels of animals found in the rear of the queue. While the early literature has found the relationship between dominance value and milking order to be tenuous at best (11–13, 15), it is possible that such social mechanisms may have been confounded by health status, with linear analyses of limited sample size failing to disentangle these mechanisms in non-disaggregated data. Alternatively, in more recent analyses in automated milking systems, where dominance has proven highly correlated with milking order (55), greater attention has also been paid to “avoiders”—animals that seem to actively avoid social interactions and therefore occupy no clear position in the herd hierarchy (44). On this farm, where resources are not severely restricted and animals are frequently remixed, energetic investments in a dominance hierarchy may offer few returns (2). Such a behavioral strategy might also explain why it is high-yielding multiparous cows and not heifers that occupy the end of the queue. Both these hypotheses are ultimately purely speculative interpretations of these exploratory results; however, if proposals to incorporate milk order records into genetic indices are progressed, any correlations between queueing position and consistent individual differences in home pen behaviors likely warrant closer inspection to mitigate the risk of unintended and potentially deleterious selection pressures.

## Conclusions

As with previous studies of milk order records, these analyses perhaps raise more questions than answers. As dairy record management systems grow to accommodate an ever wider range data streams, perhaps future work considering more herds from a wider range of management strategies will succeed in further untangling the complex web of explanatory variables at the individual, herd, and farm levels that drive variation in queueing patterns. This dataset has, none the less, demonstrated the utility of unsupervised machine learning tools in ethological studies using sensor platforms to study larger groups of animals over extended periods of time. While these analyses recovered no evidence of social cohesion amongst large or temporally consistent subgroups, information theoretic approaches succeeded in clarifying the underlying pattern of heterogeneity in error variance between animals and also demonstrated an advantage in recovering evidence of non-uniform patterns in temporal non-stationary over basic EDA tools. After incorporating these insights into the structure of subsequent linear models, these model-free tools then showed some capacity to confirm inferential results where probabilistic assumptions were not strictly met, as well as an aptitude for recovering significant associations not captured by a simple linear effect. This flexible clustering-based approach to identifying significant bivariate associations was then easily extended to accommodate two high dimensional behavioral axes, providing equivalent insights to more computationally taxing mixed effect models. While UML approaches are by no means infallible, as seen here with artifacts produced by the spectral embeddings, these analyses have demonstrated that such tools can add value at every stage of the standard hypothesis-driven linear analysis pipeline, and may even offer an advantages over model-based approaches in early-stage exploratory projects. While many new methodological developments are doubtless on the ethological horizon, we hope this algorithmic toolset will provide a meaningful step forwards to meet the challenges of a future defined by ever larger and more complex data.

## Data Availability Statement

The datasets generated for this study are available on request to the corresponding author.

## Ethics Statement

The animal study was reviewed and approved by Colorado State University IACUC (protocol ID: 16-6704AA). Written informed consent was obtained from the owners for the participation of their animals in this study.

## Author Contributions

DM and PP contributed to experimental design and data collection. CM and FH contributed to data analysis. CM contributed to manuscript preparation. All authors contributed to manuscript writing and revisions.

## Conflict of Interest

The authors declare that the research was conducted in the absence of any commercial or financial relationships that could be construed as a potential conflict of interest.
