# Supplementary figures and images for: Mind the Queue: A Case Study in Visualizing Heterogeneous Behavioral Patterns in Livestock Sensor Data Using Unsupervised Machine Learning Techniques (part 2 of 4)
Source: Front Vet Sci. 2020 Aug 13;7:523. doi: 10.3389/fvets.2020.00523 (PMC7518149; doi:10.3389/fvets.2020.00523)

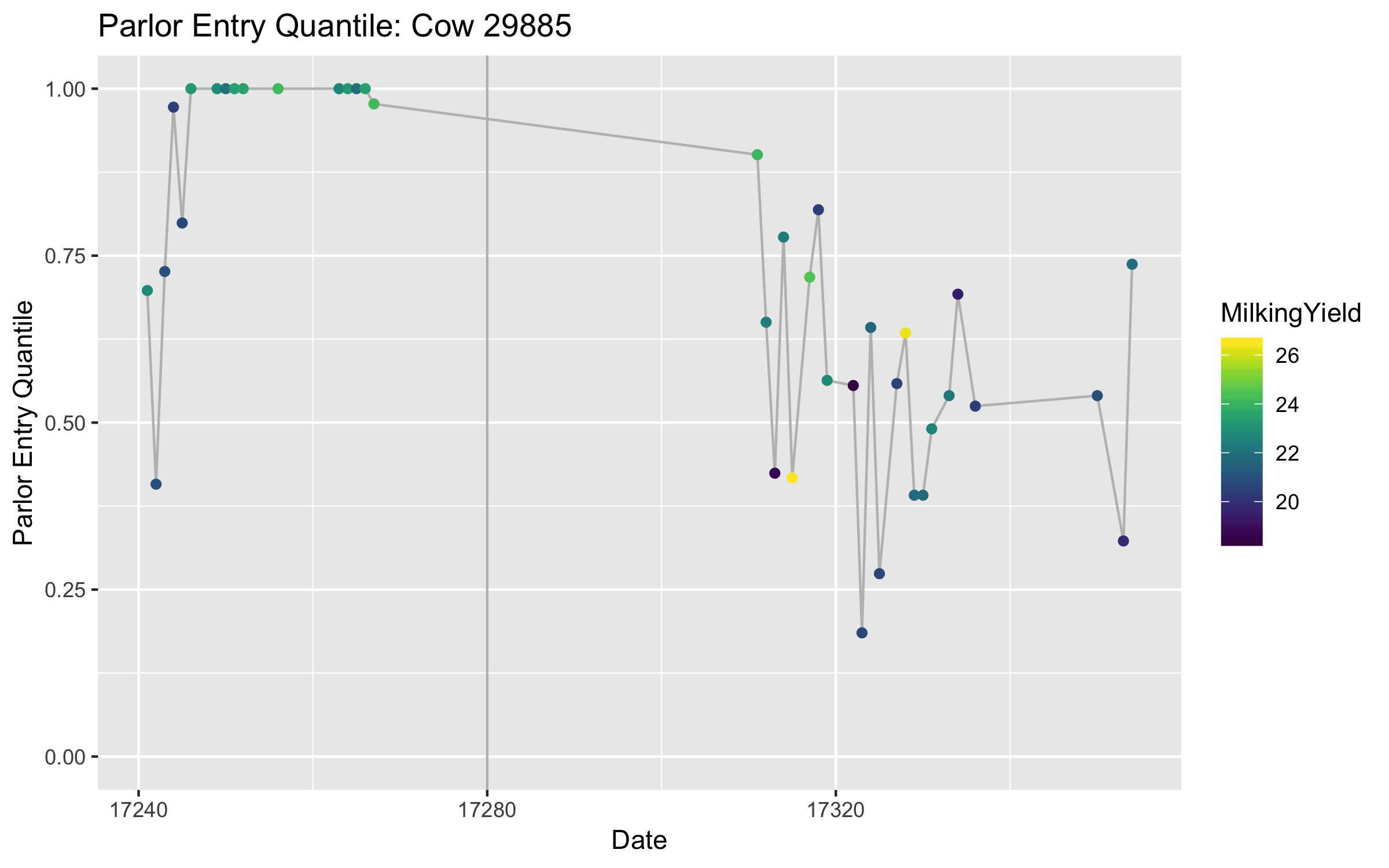

Supplement: Supplementary file 2 [file Data_Sheet_2.ZIP › Milking Yield/Cow_29885.jpg]

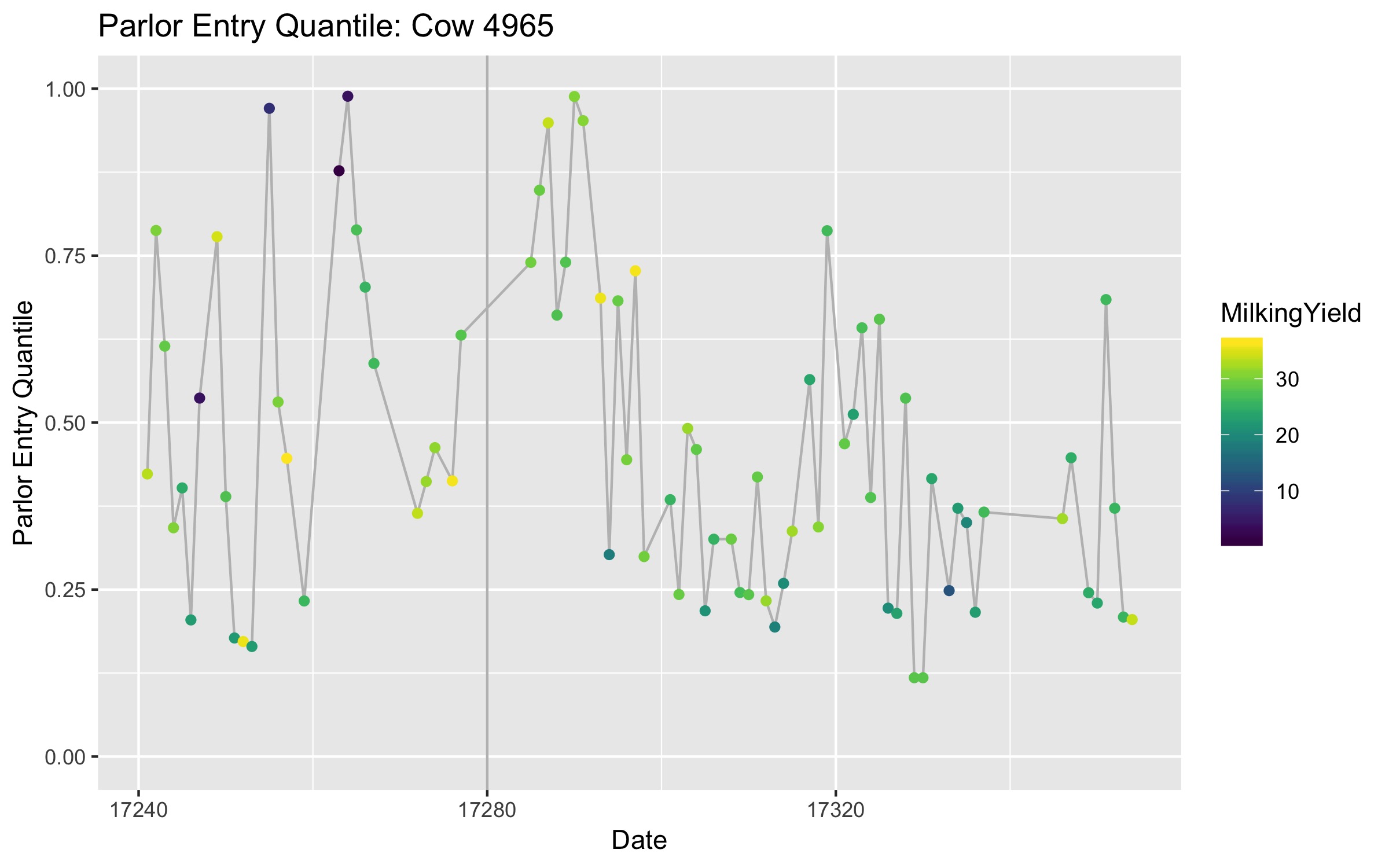

Supplement: Supplementary file 2 [file Data_Sheet_2.ZIP › Milking Yield/Cow_4965.jpg]

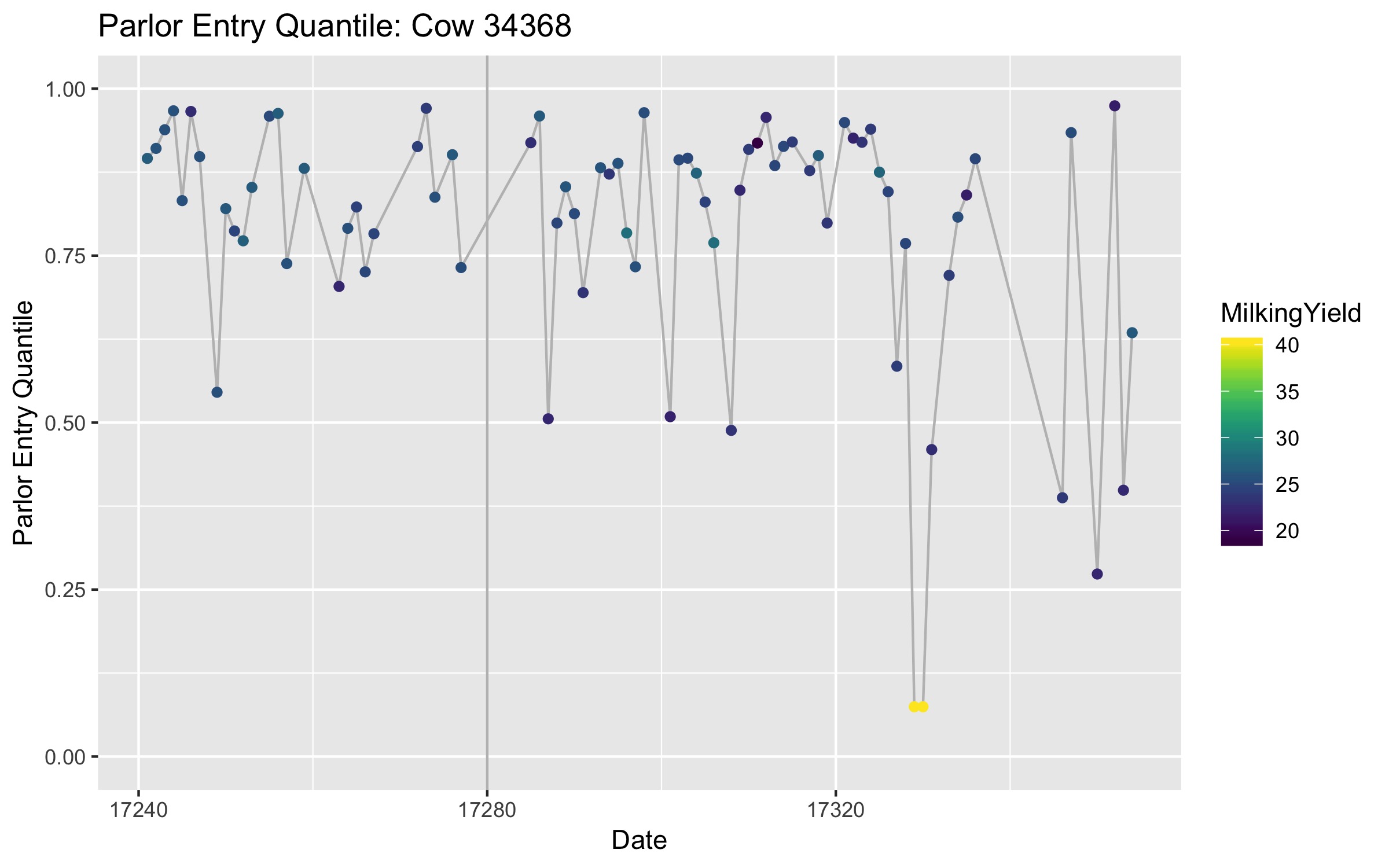

Supplement: Supplementary file 2 [file Data_Sheet_2.ZIP › Milking Yield/Cow_34368.jpg]

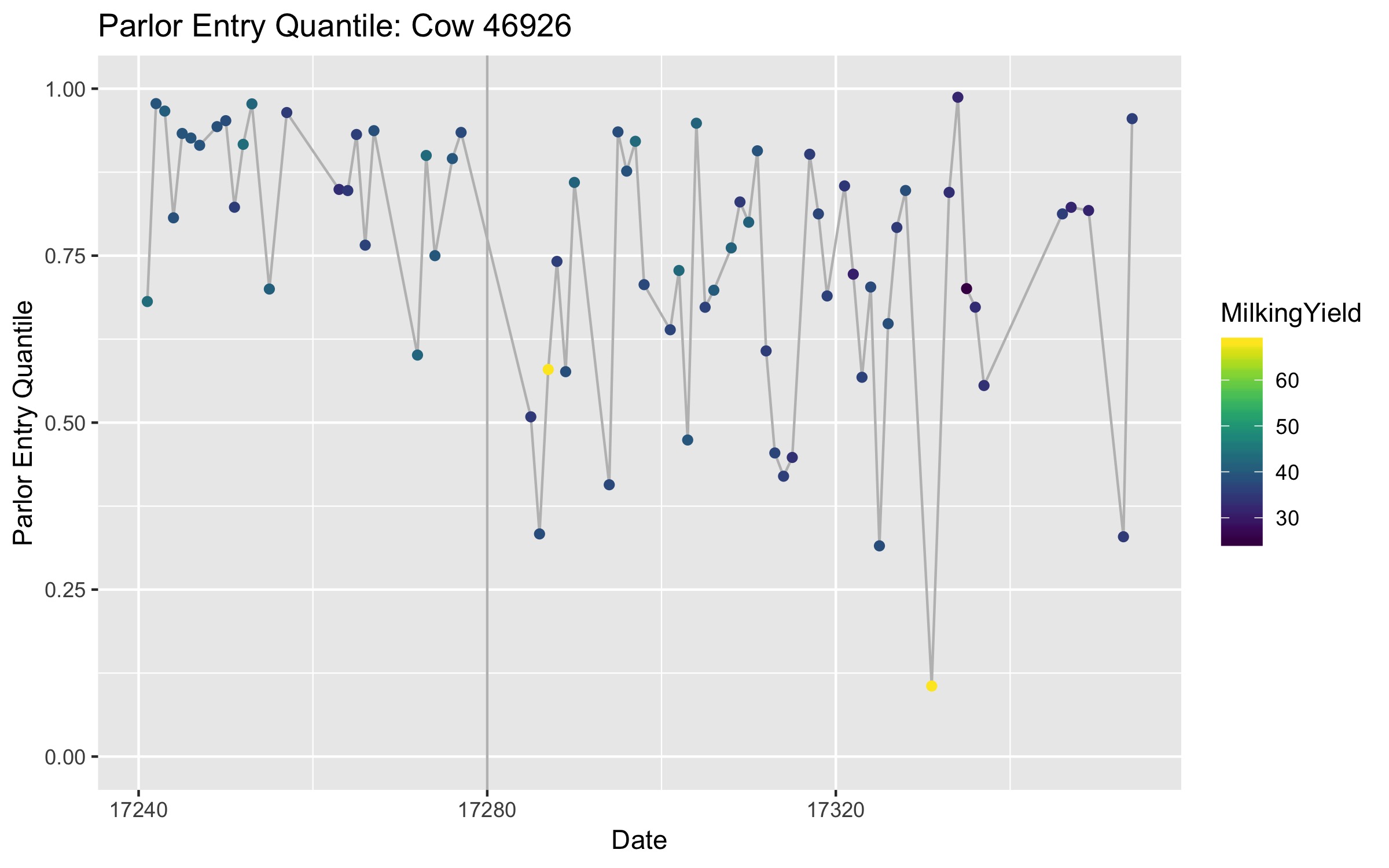

Supplement: Supplementary file 2 [file Data_Sheet_2.ZIP › Milking Yield/Cow_46926.jpg]

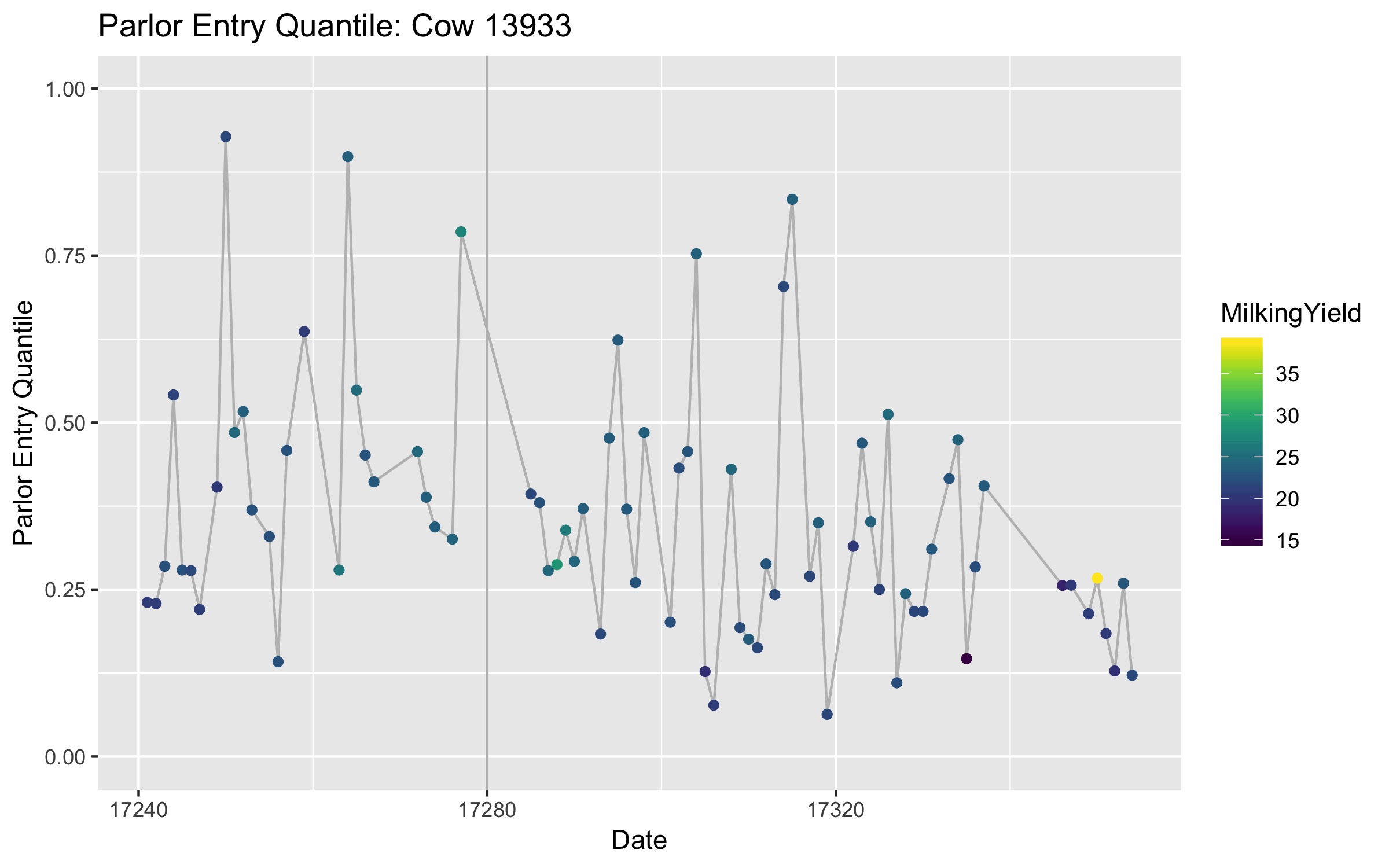

Supplement: Supplementary file 2 [file Data_Sheet_2.ZIP › Milking Yield/Cow_13933.jpg]

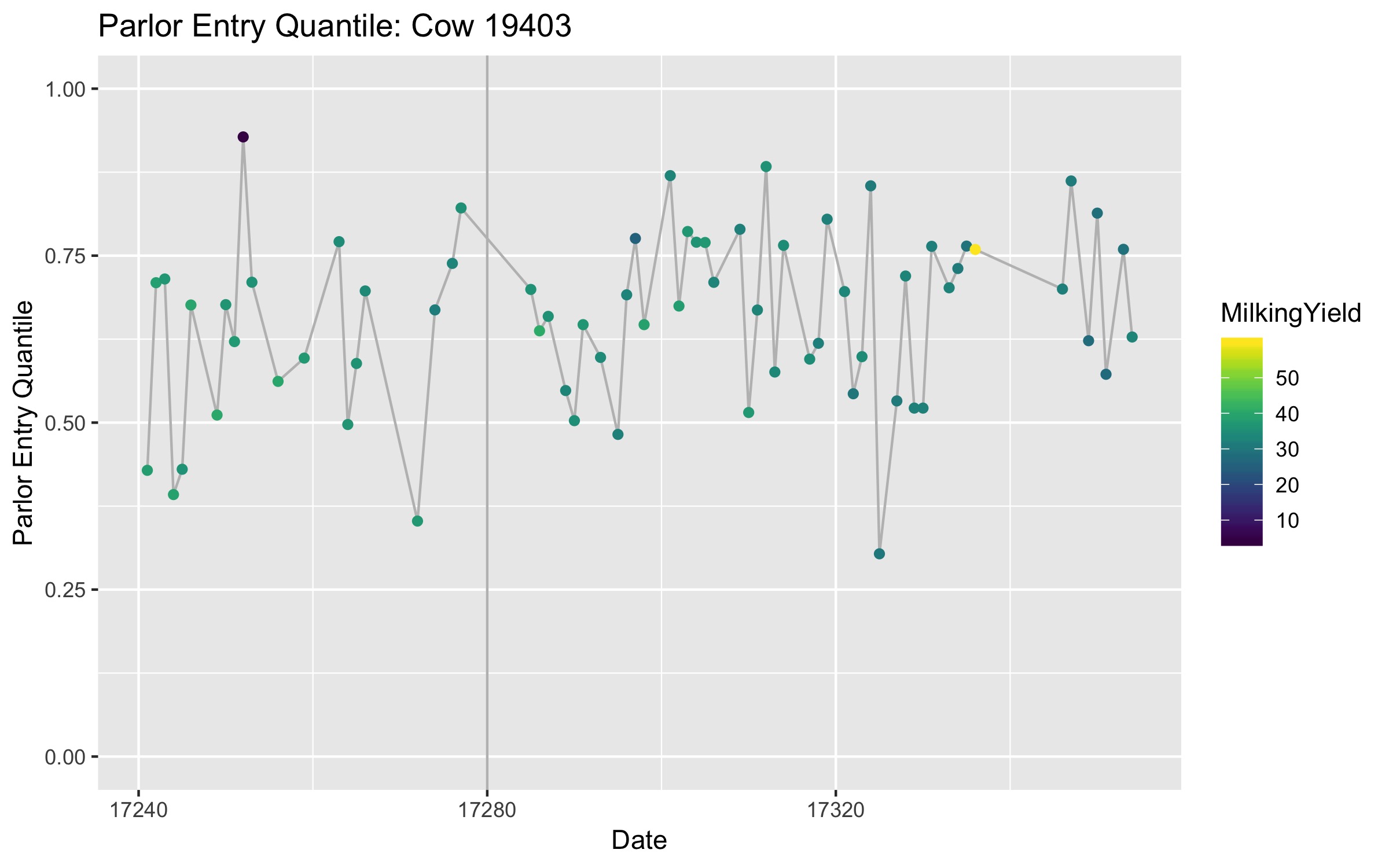

Supplement: Supplementary file 2 [file Data_Sheet_2.ZIP › Milking Yield/Cow_19403.jpg]

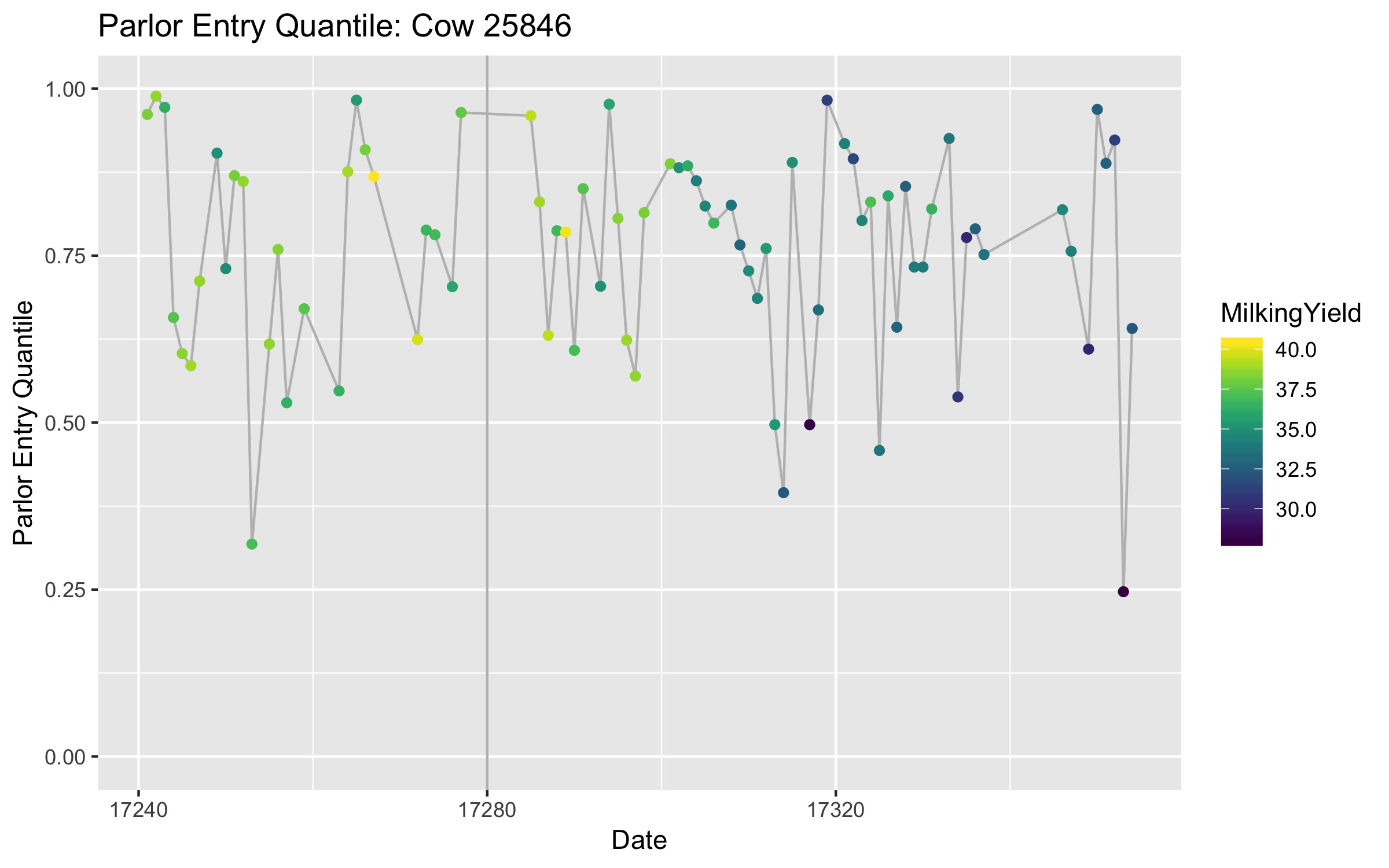

Supplement: Supplementary file 2 [file Data_Sheet_2.ZIP › Milking Yield/Cow_25846.jpg]

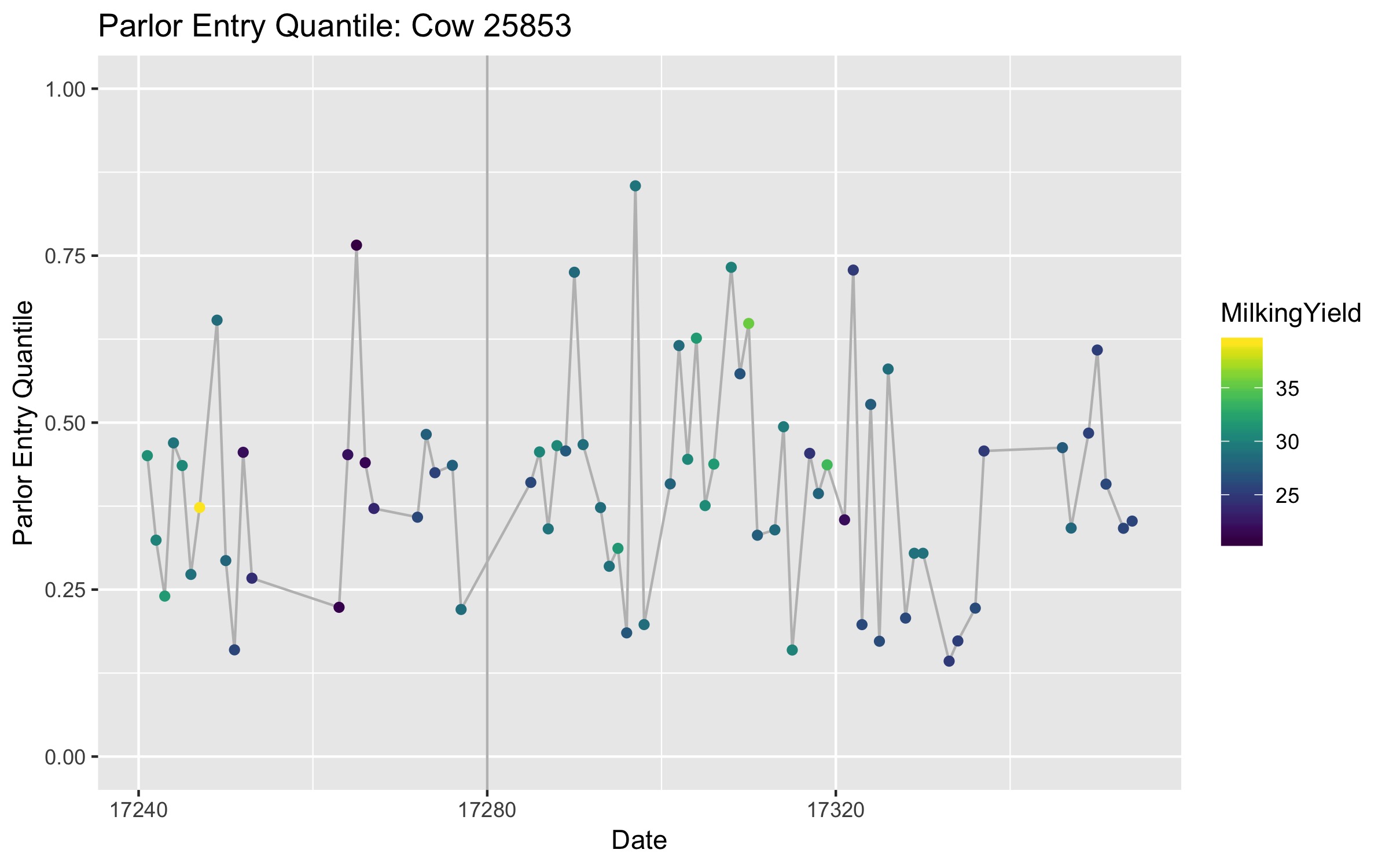

Supplement: Supplementary file 2 [file Data_Sheet_2.ZIP › Milking Yield/Cow_25853.jpg]

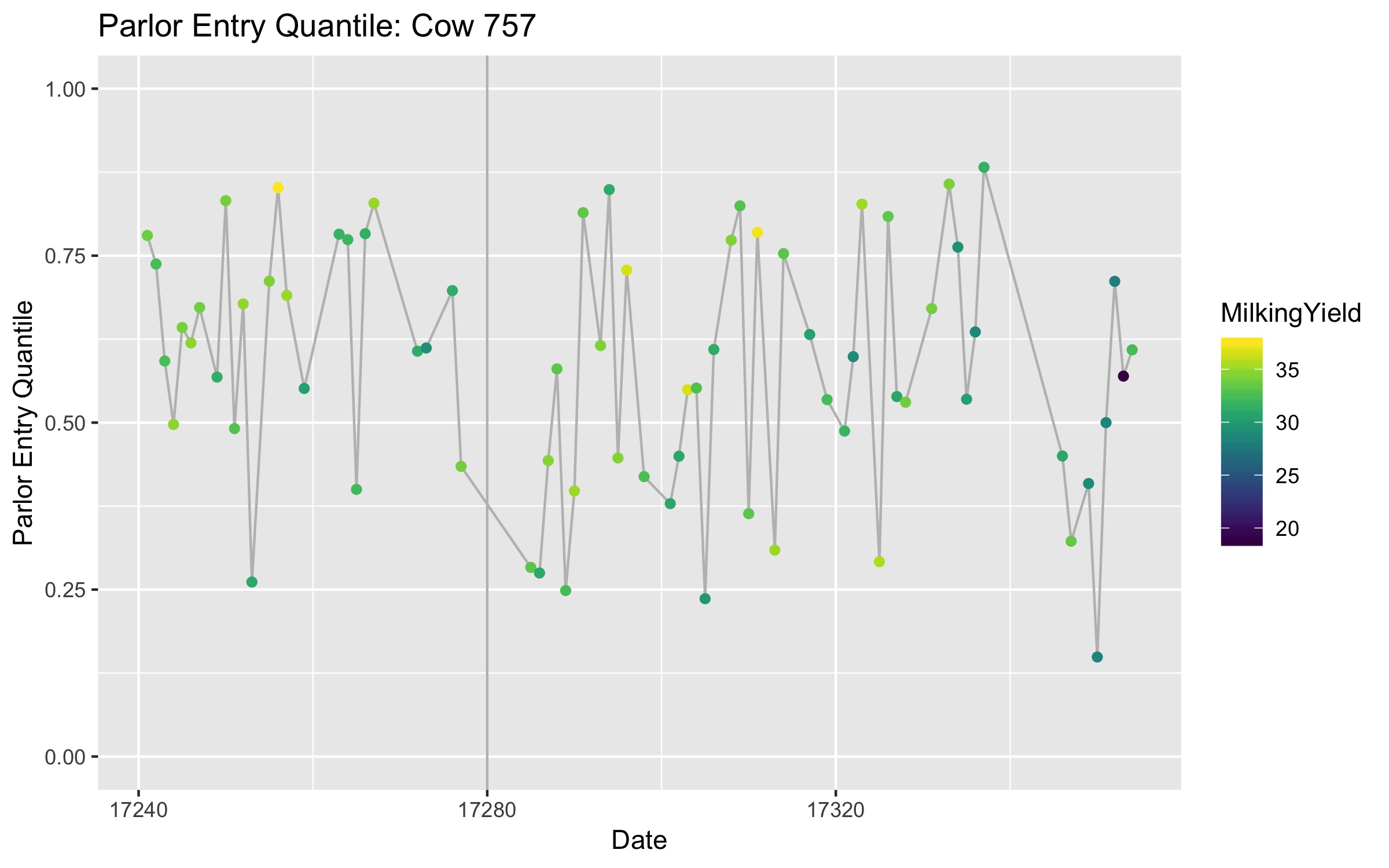

Supplement: Supplementary file 2 [file Data_Sheet_2.ZIP › Milking Yield/Cow_757.jpg]

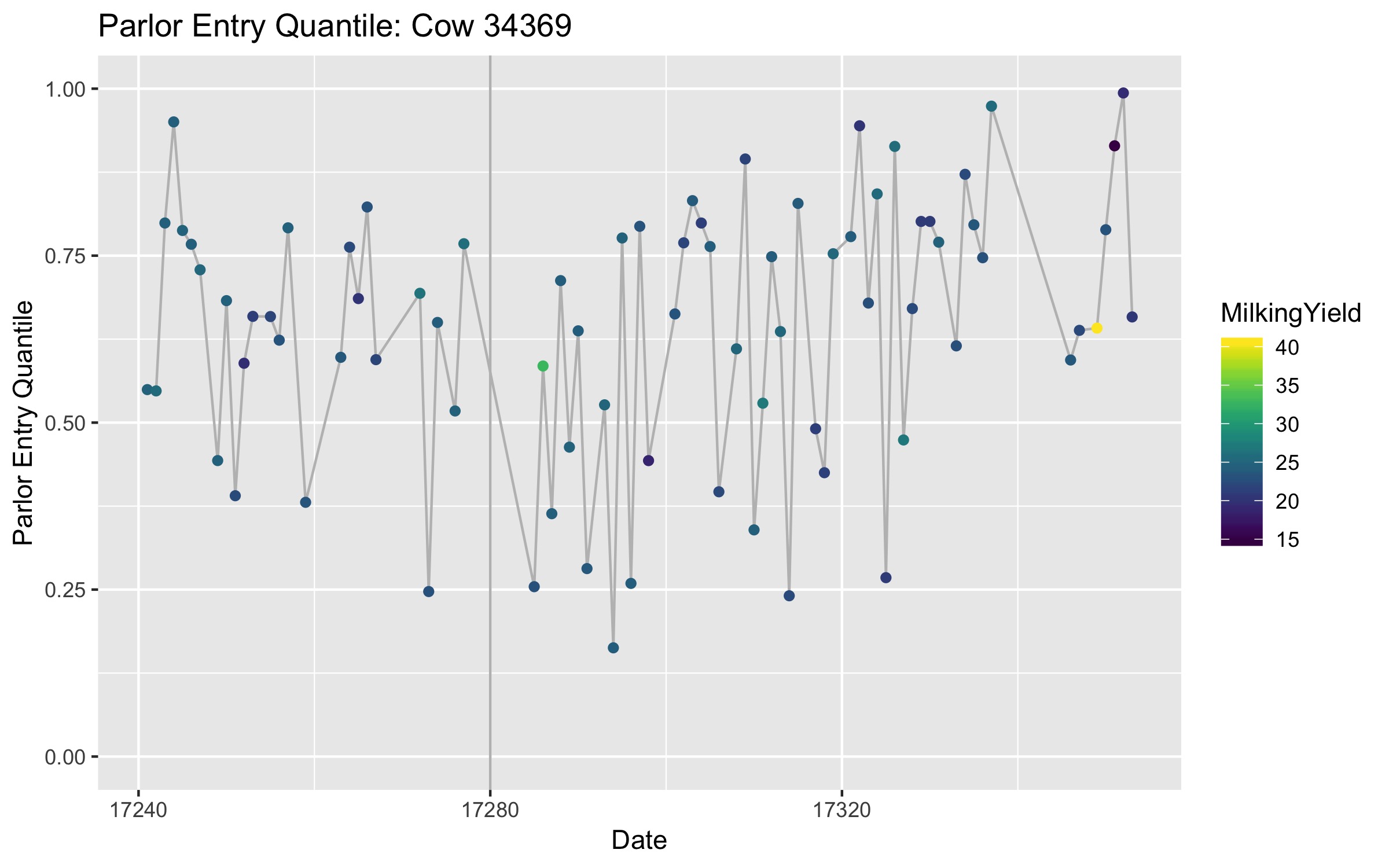

Supplement: Supplementary file 2 [file Data_Sheet_2.ZIP › Milking Yield/Cow_34369.jpg]

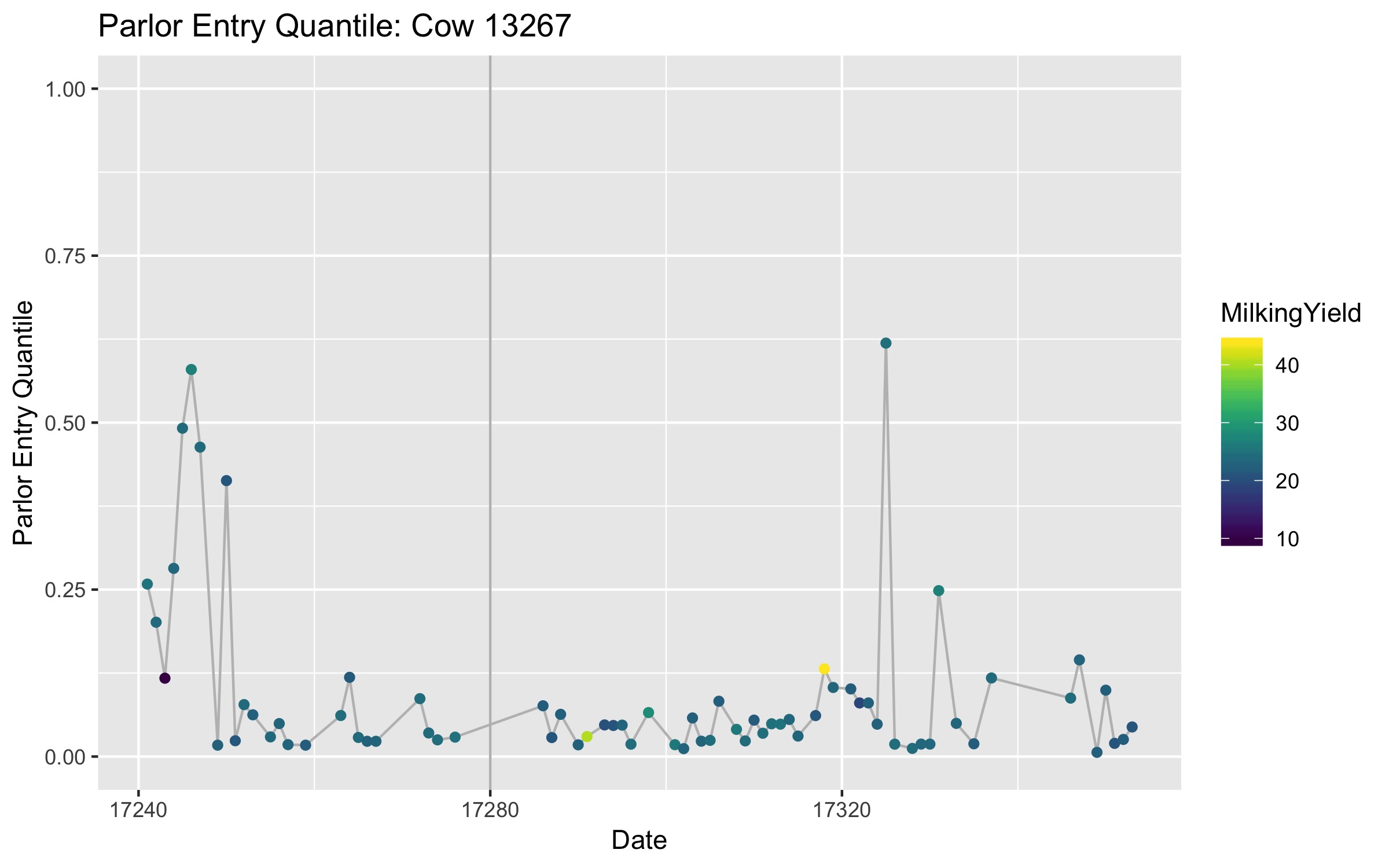

Supplement: Supplementary file 2 [file Data_Sheet_2.ZIP › Milking Yield/Cow_13267.jpg]

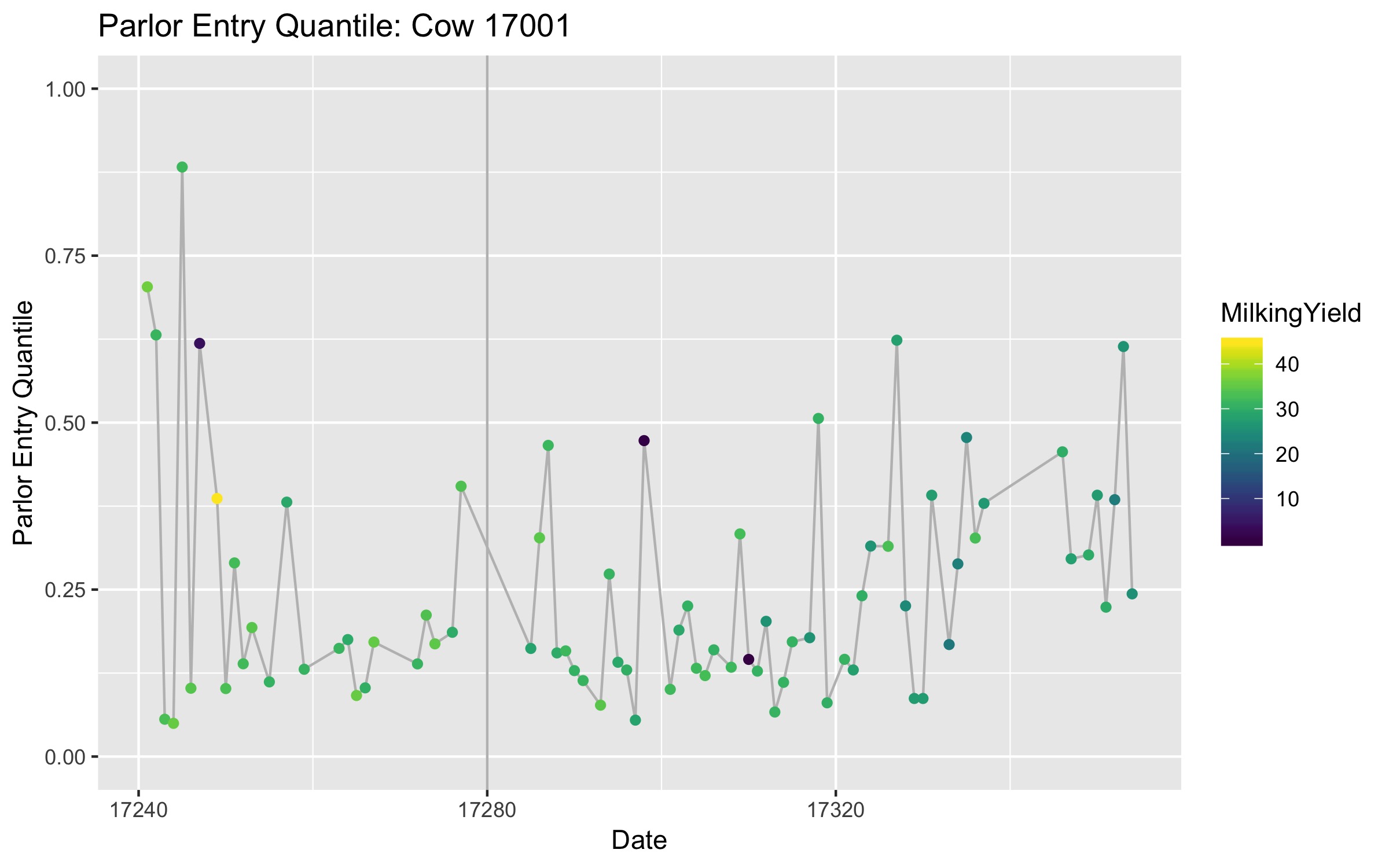

Supplement: Supplementary file 2 [file Data_Sheet_2.ZIP › Milking Yield/Cow_17001.jpg]

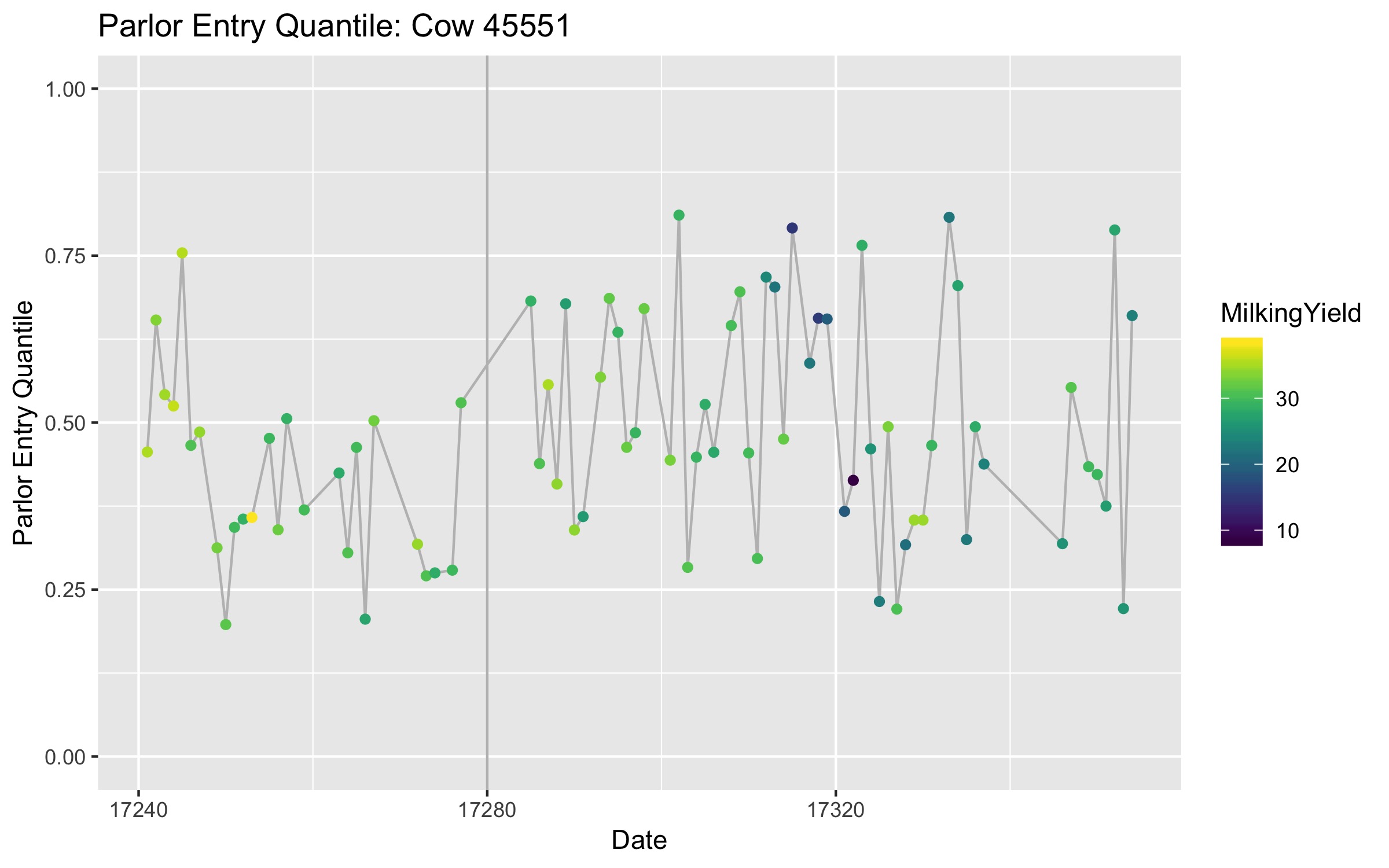

Supplement: Supplementary file 2 [file Data_Sheet_2.ZIP › Milking Yield/Cow_45551.jpg]

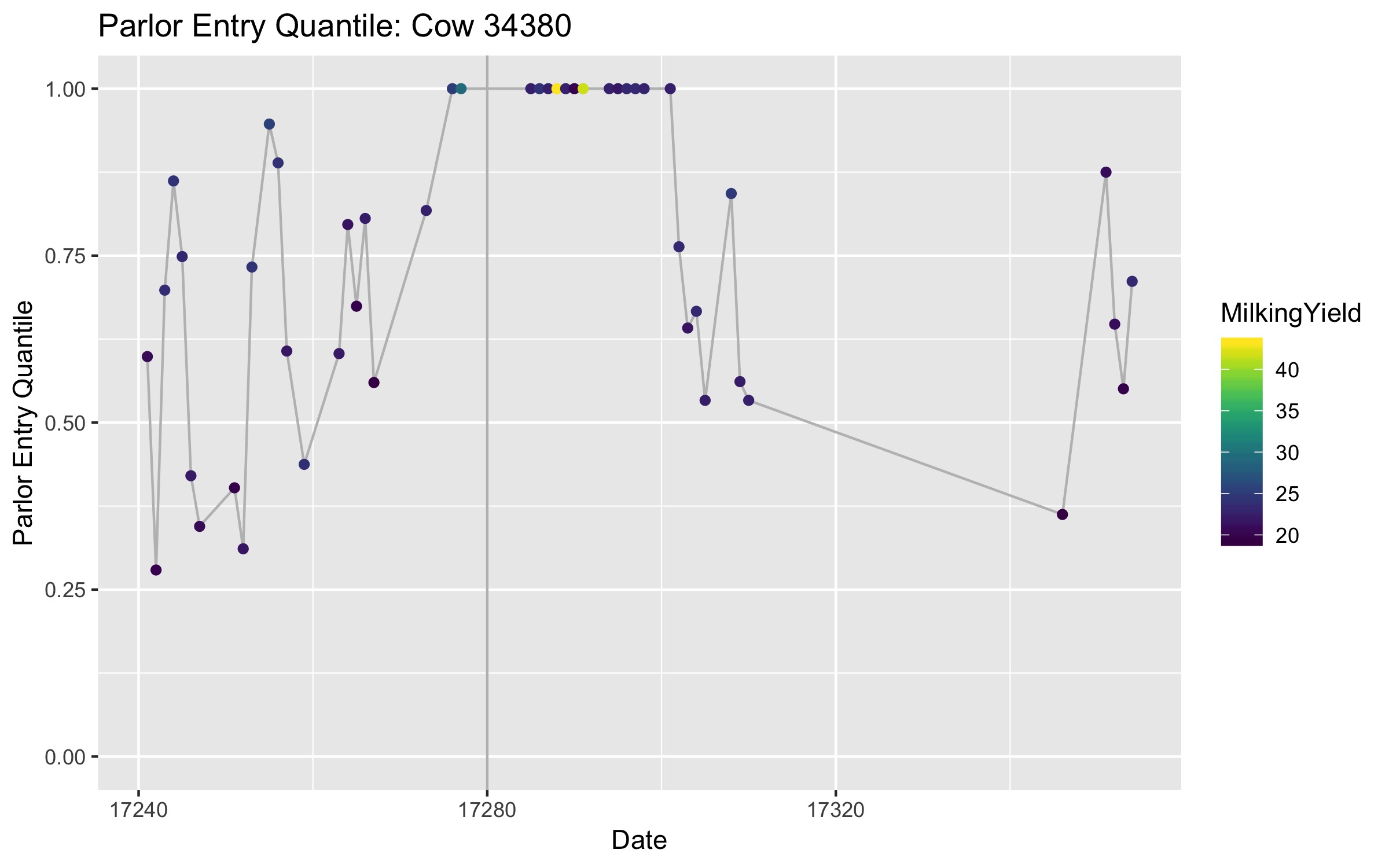

Supplement: Supplementary file 2 [file Data_Sheet_2.ZIP › Milking Yield/Cow_34380.jpg]

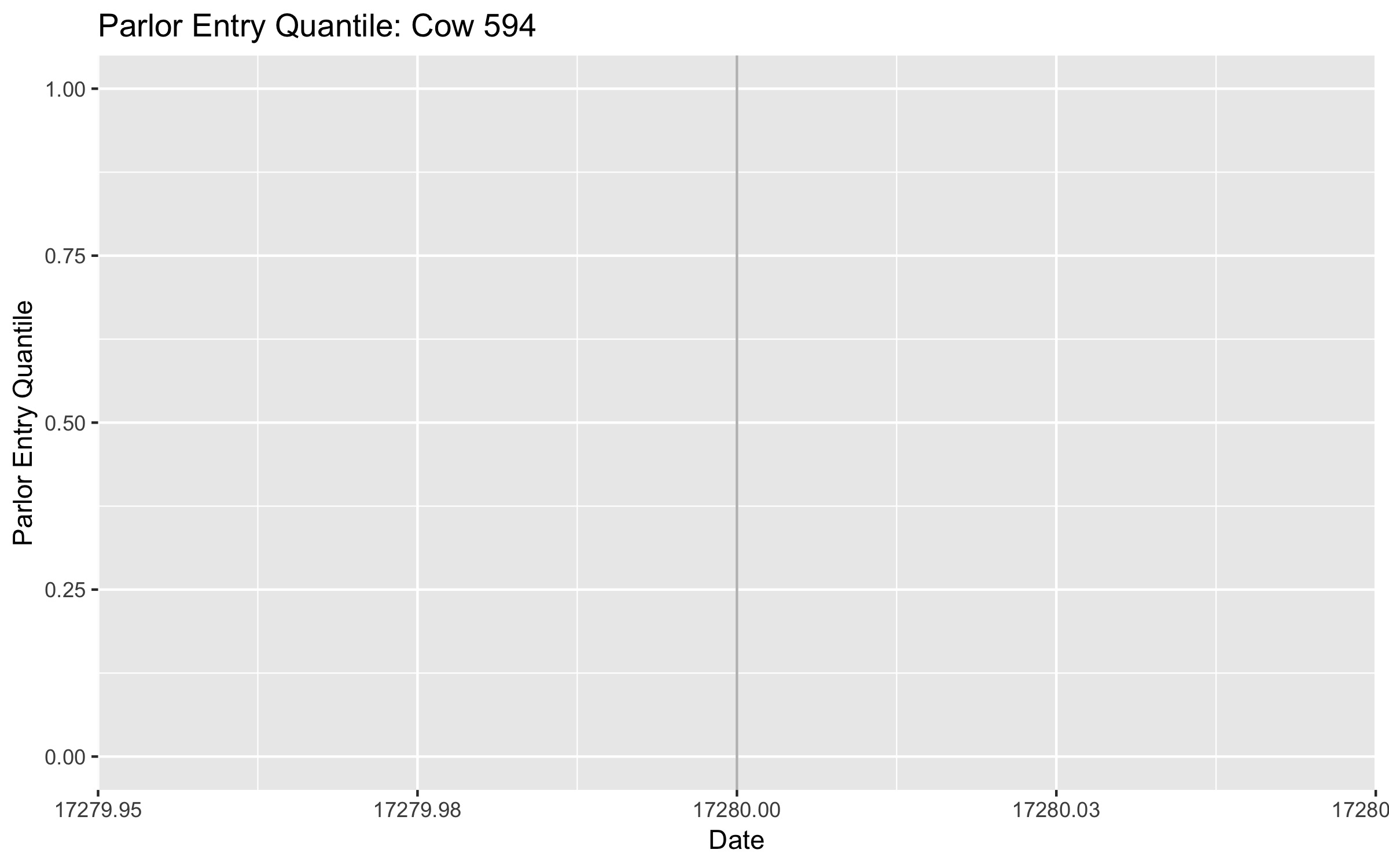

Supplement: Supplementary file 2 [file Data_Sheet_2.ZIP › Milking Yield/Cow_594.jpg]

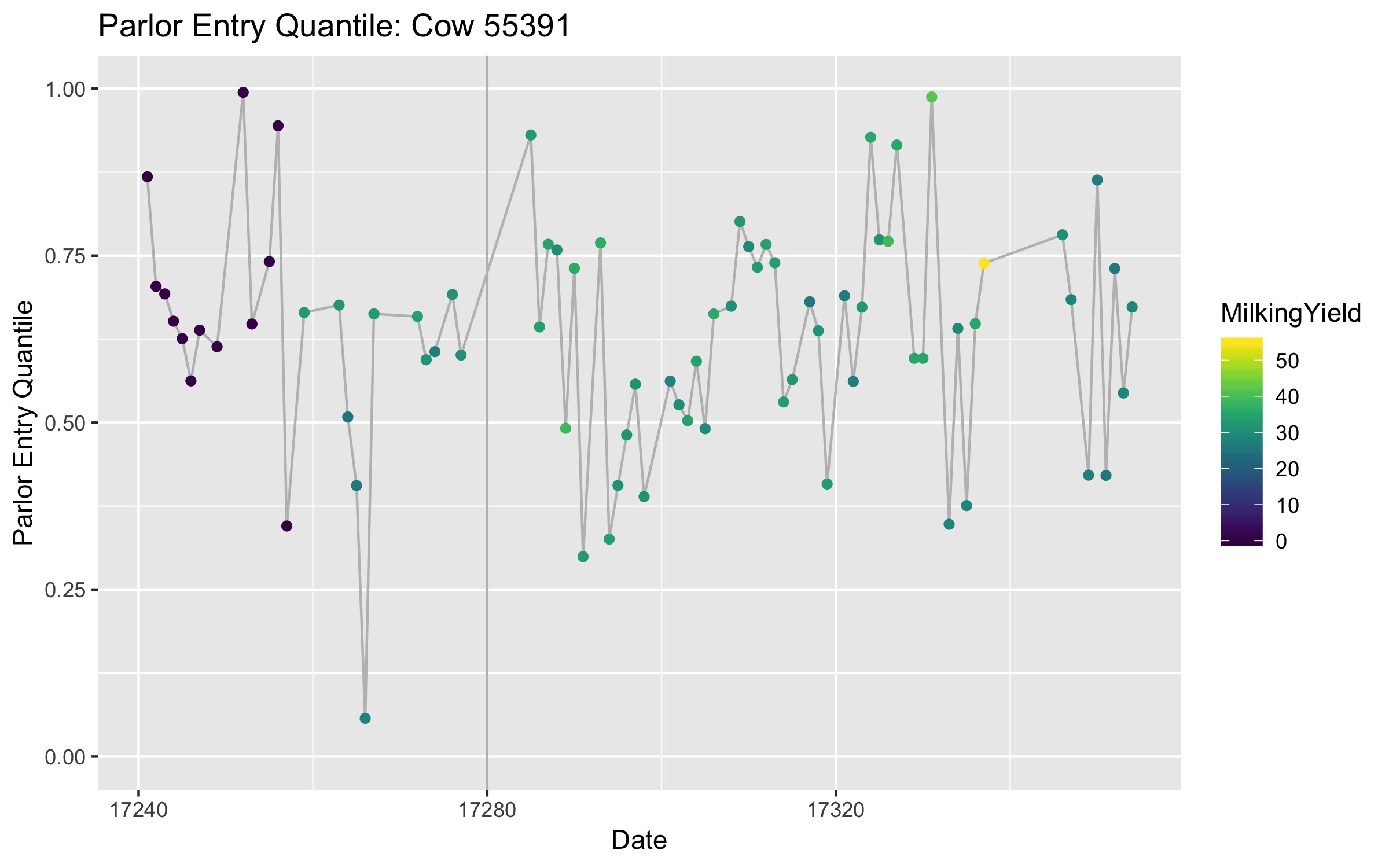

Supplement: Supplementary file 2 [file Data_Sheet_2.ZIP › Milking Yield/Cow_55391.jpg]

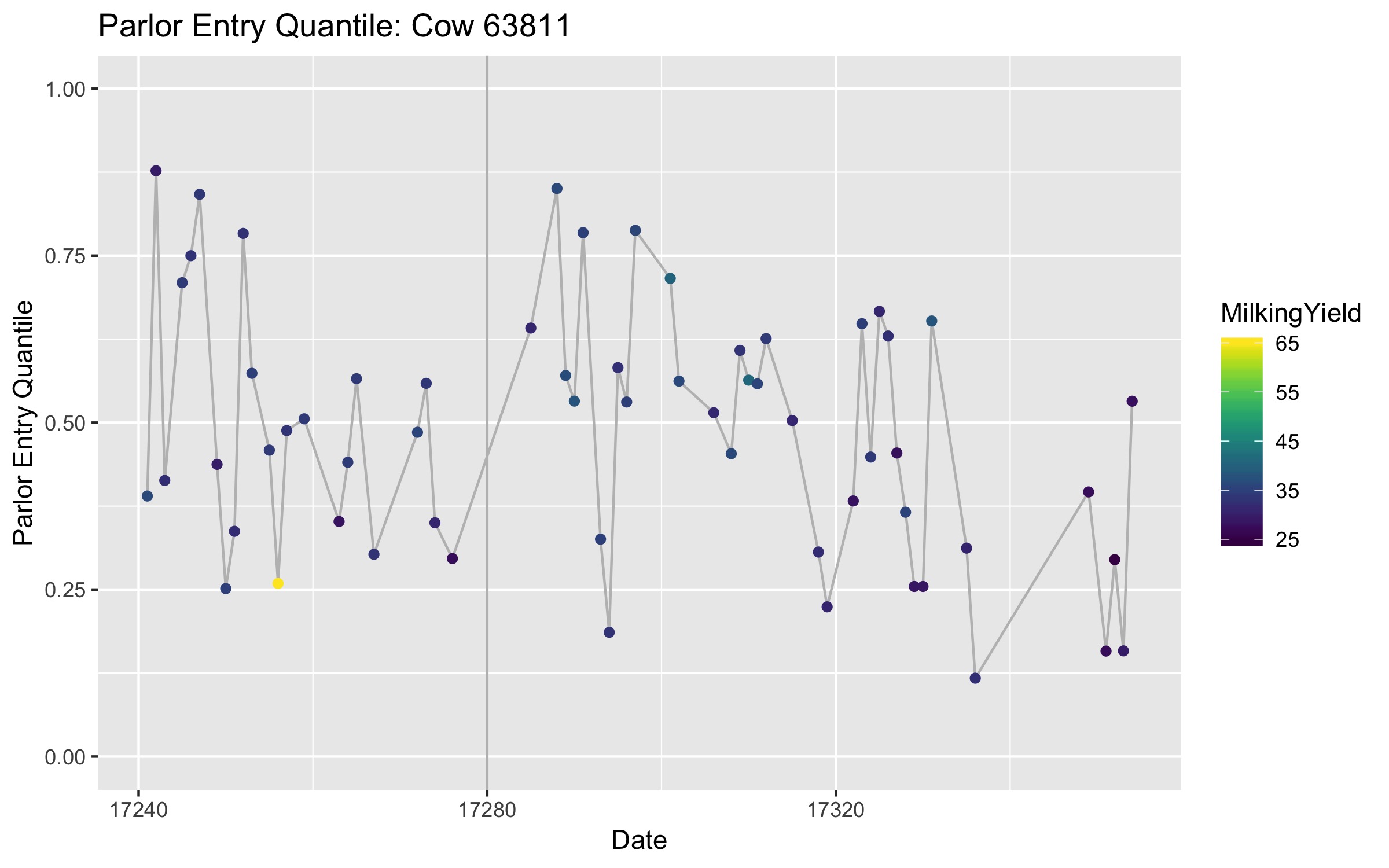

Supplement: Supplementary file 2 [file Data_Sheet_2.ZIP › Milking Yield/Cow_63811.jpg]

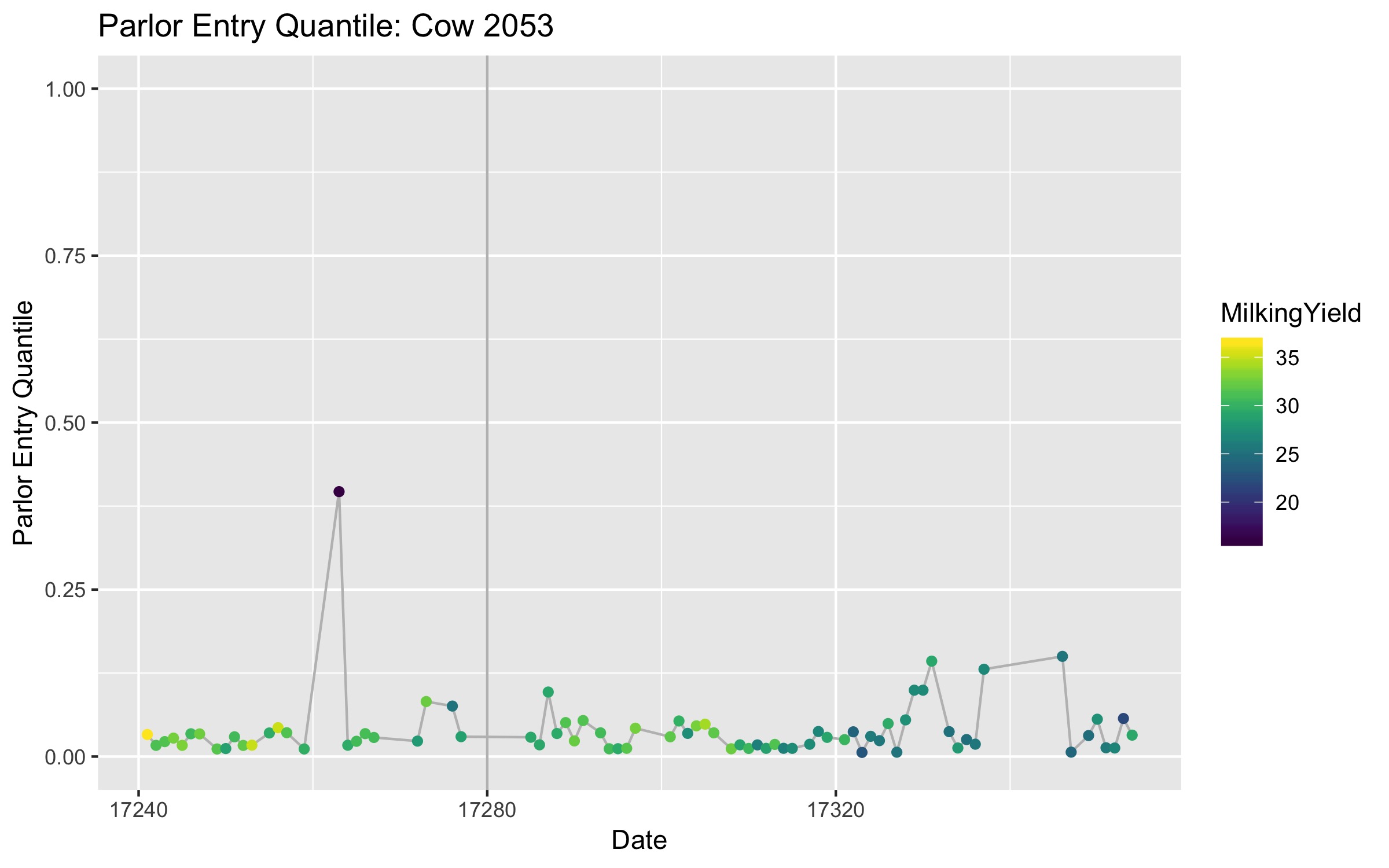

Supplement: Supplementary file 2 [file Data_Sheet_2.ZIP › Milking Yield/Cow_2053.jpg]

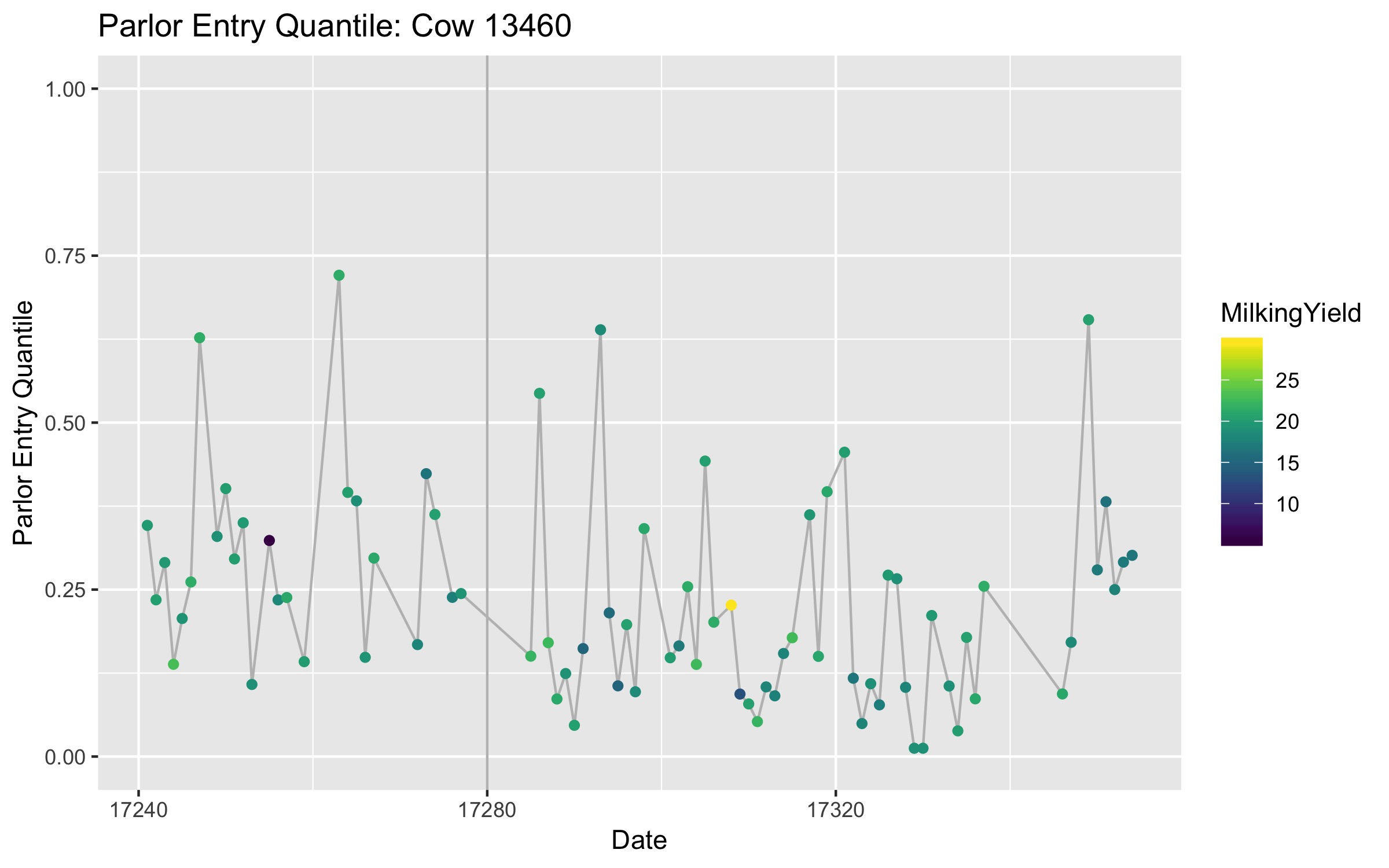

Supplement: Supplementary file 2 [file Data_Sheet_2.ZIP › Milking Yield/Cow_13460.jpg]

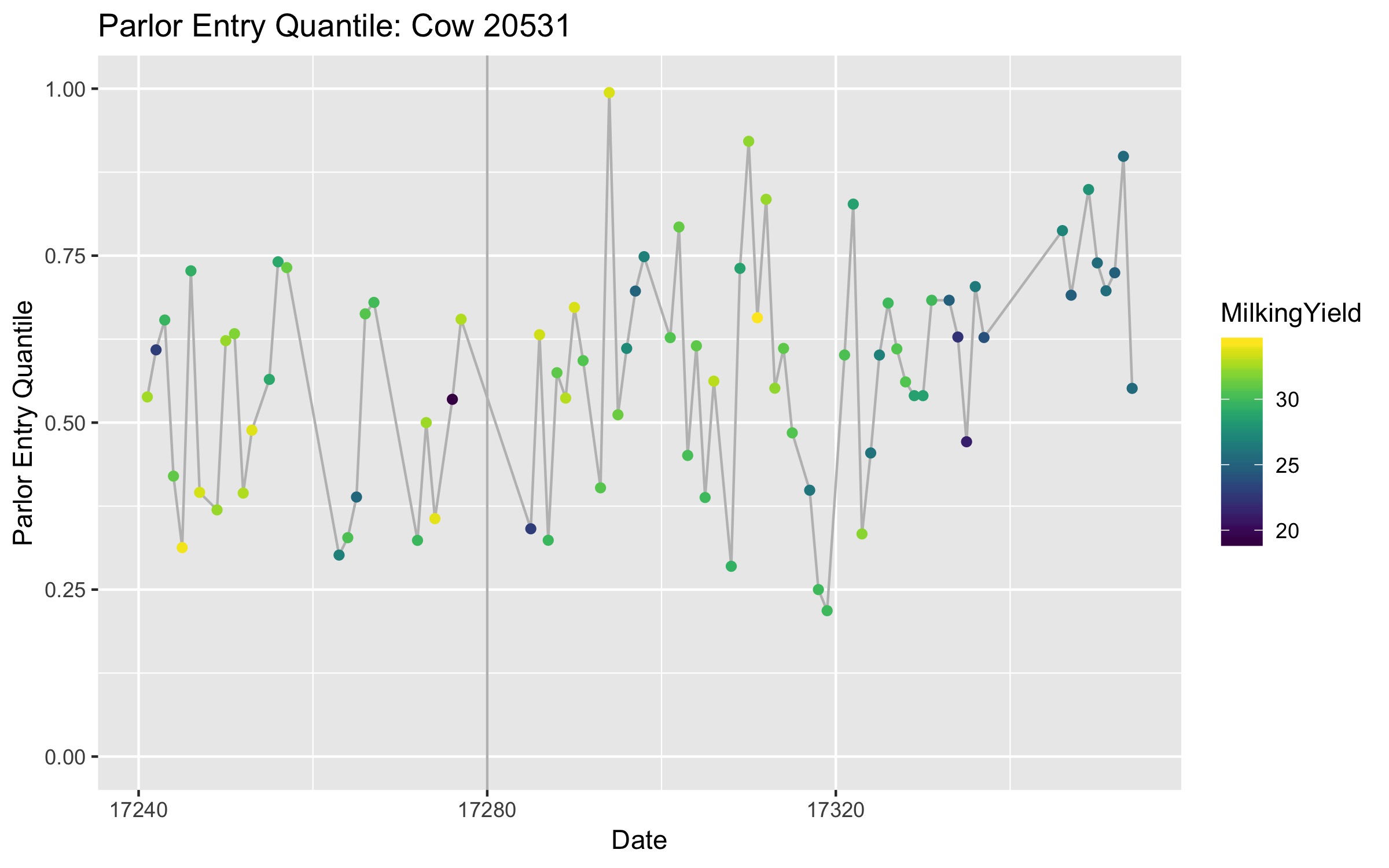

Supplement: Supplementary file 2 [file Data_Sheet_2.ZIP › Milking Yield/Cow_20531.jpg]

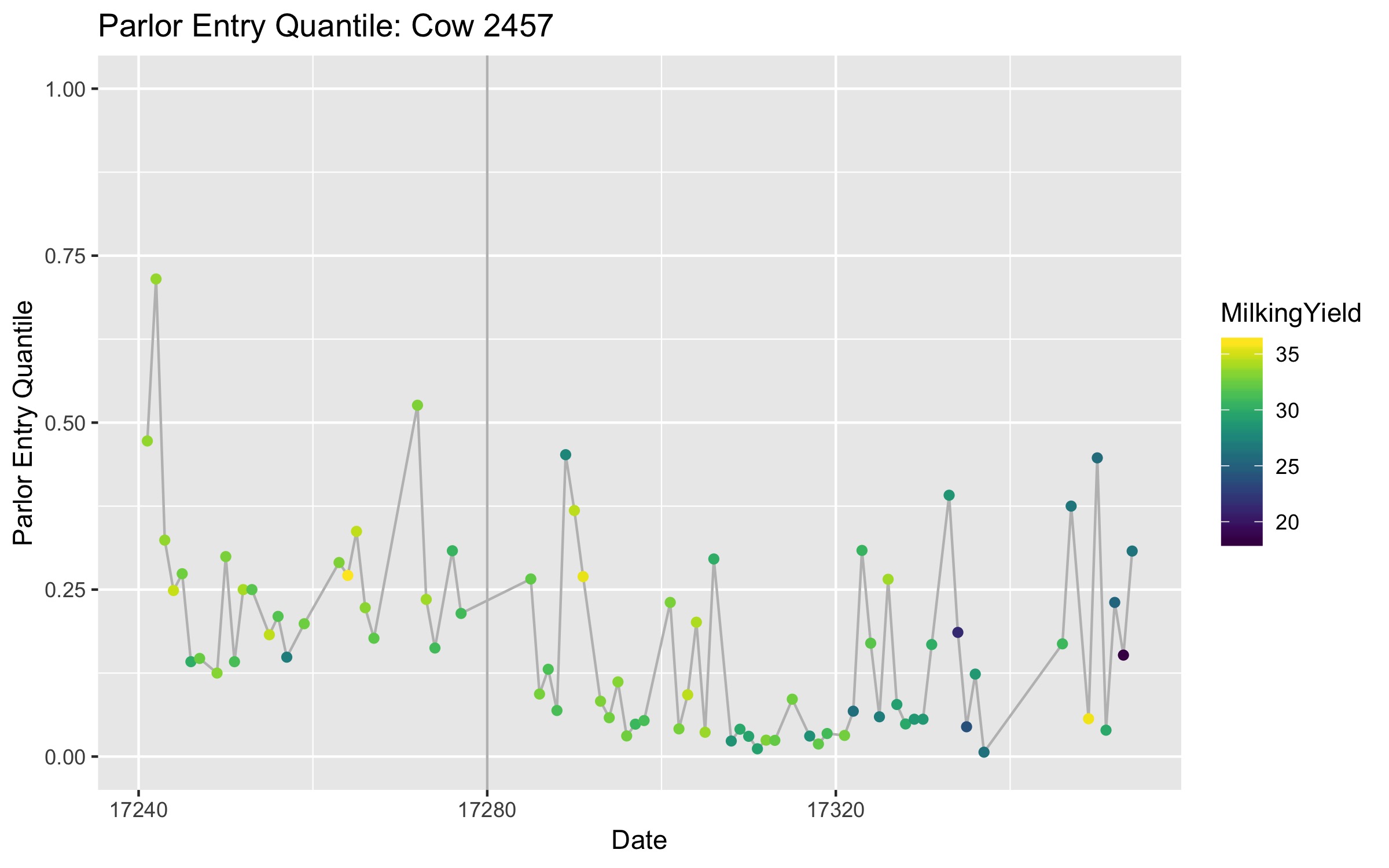

Supplement: Supplementary file 2 [file Data_Sheet_2.ZIP › Milking Yield/Cow_2457.jpg]

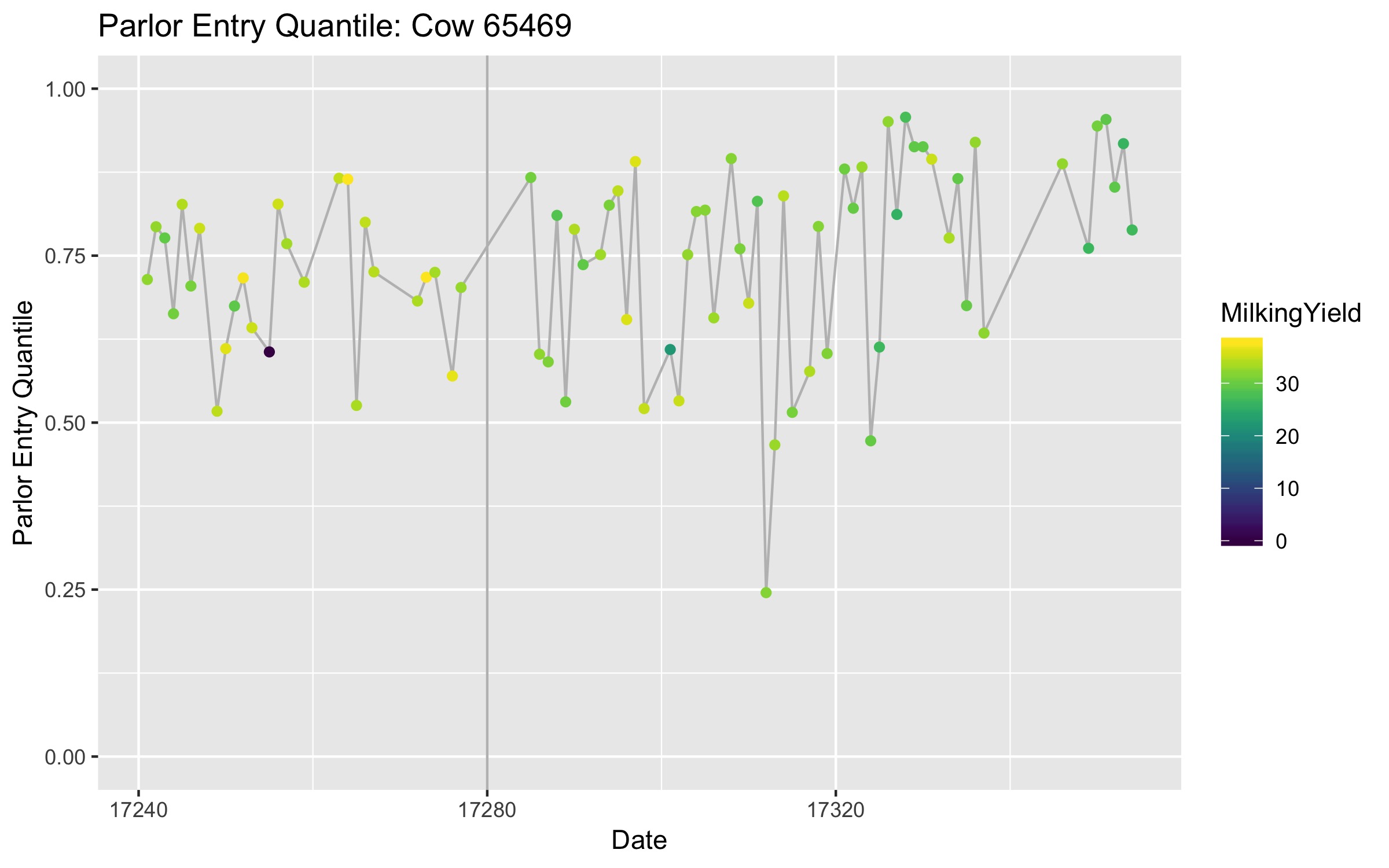

Supplement: Supplementary file 2 [file Data_Sheet_2.ZIP › Milking Yield/Cow_65469.jpg]

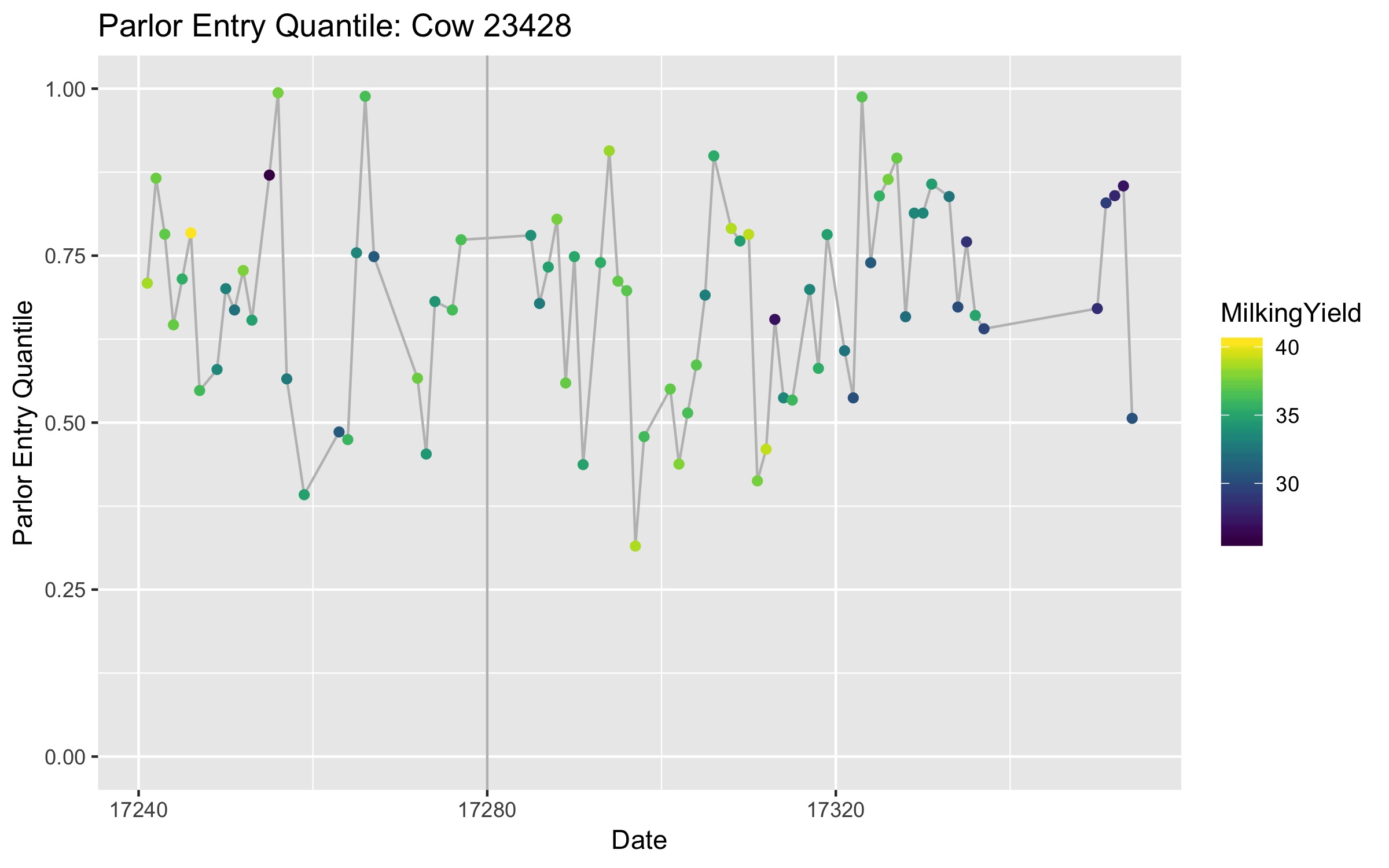

Supplement: Supplementary file 2 [file Data_Sheet_2.ZIP › Milking Yield/Cow_23428.jpg]

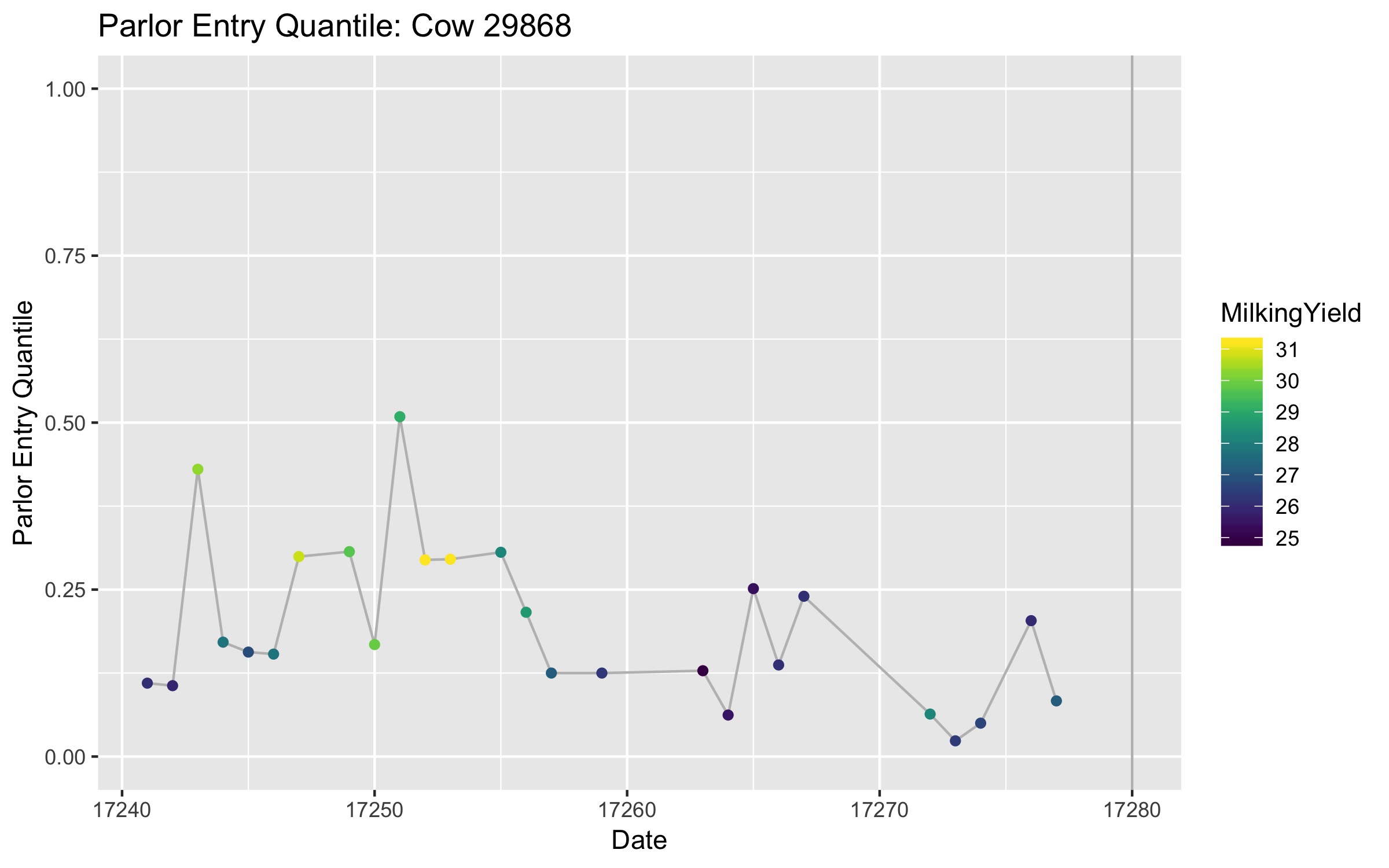

Supplement: Supplementary file 2 [file Data_Sheet_2.ZIP › Milking Yield/Cow_29868.jpg]

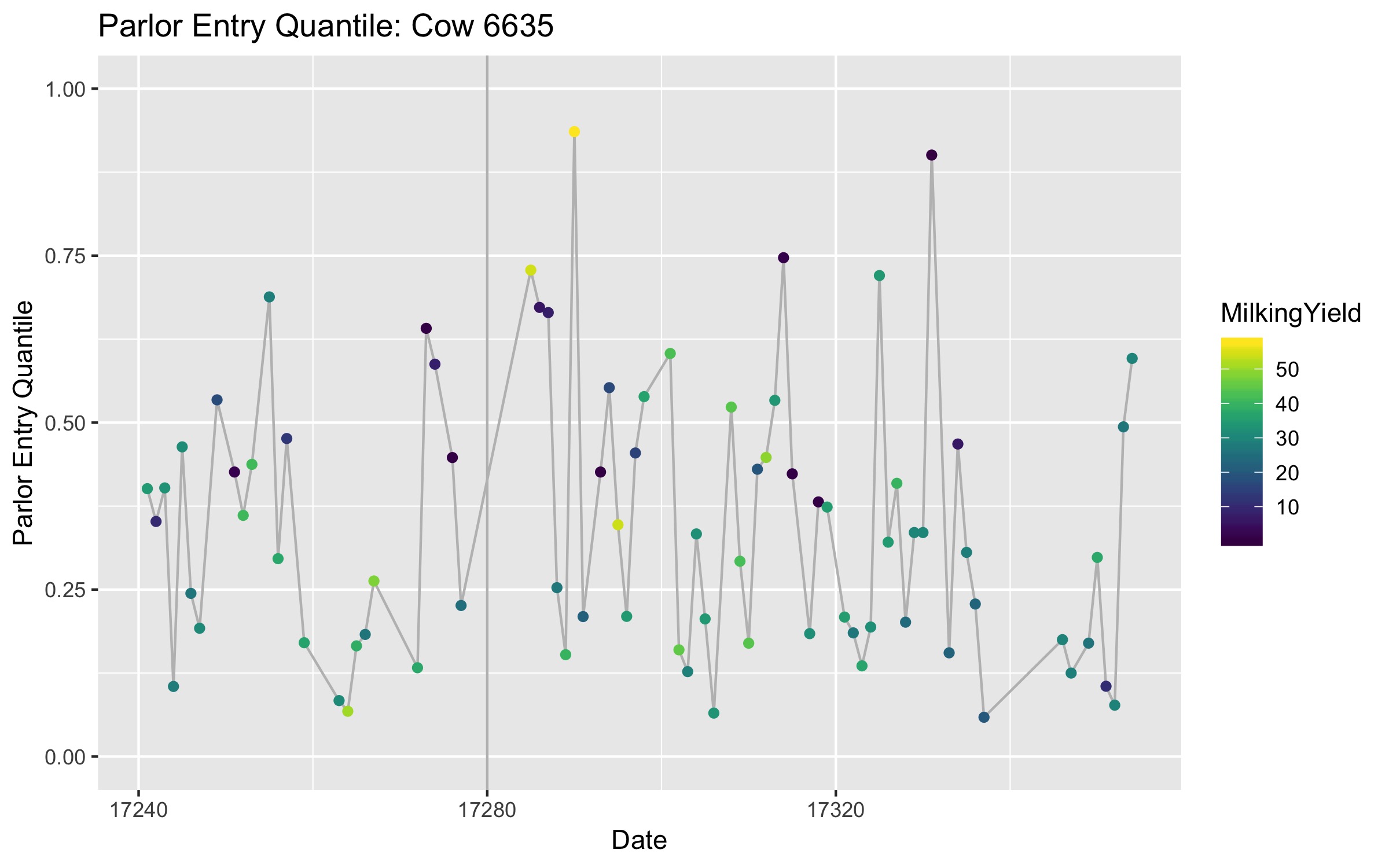

Supplement: Supplementary file 2 [file Data_Sheet_2.ZIP › Milking Yield/Cow_6635.jpg]

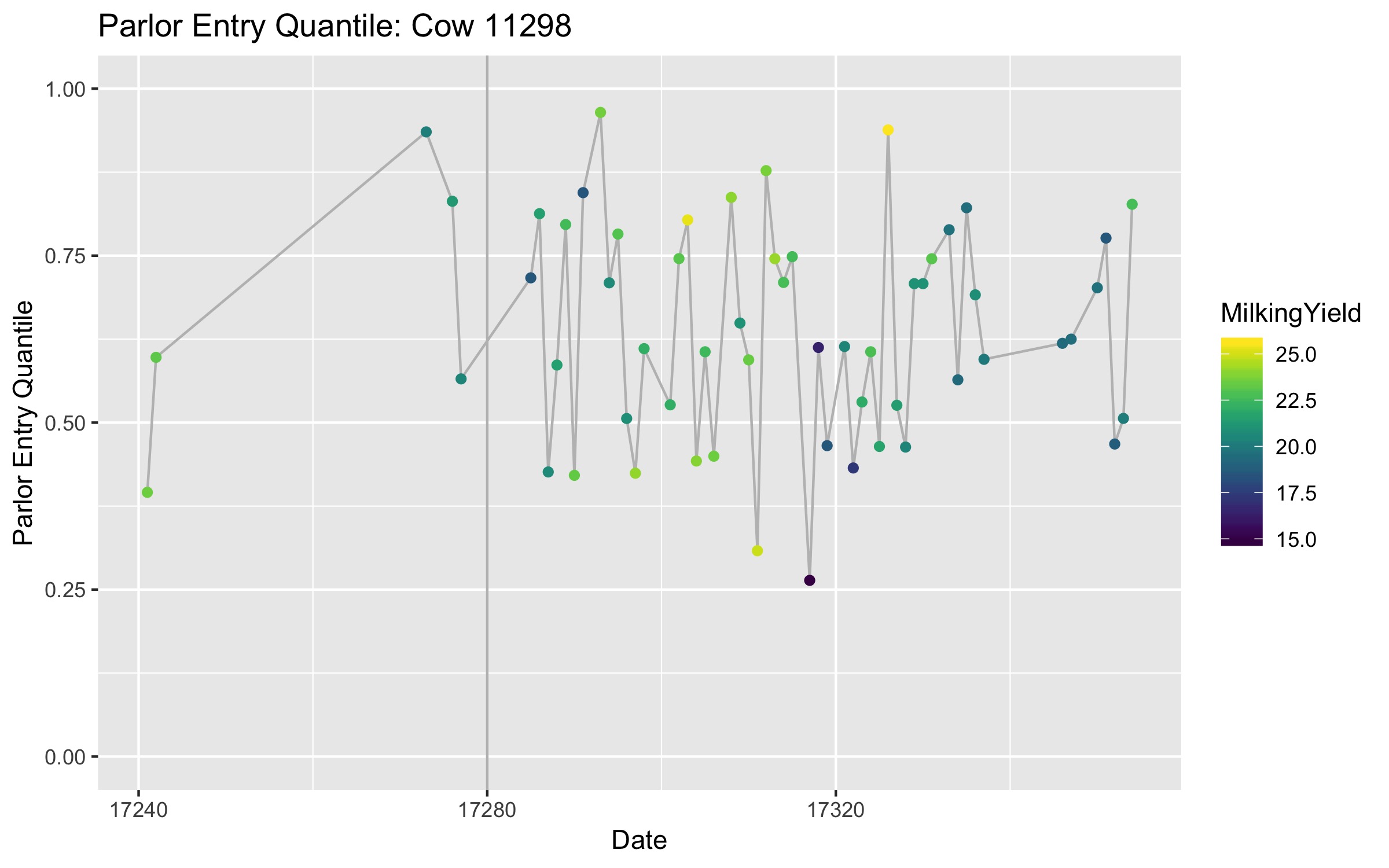

Supplement: Supplementary file 2 [file Data_Sheet_2.ZIP › Milking Yield/Cow_11298.jpg]

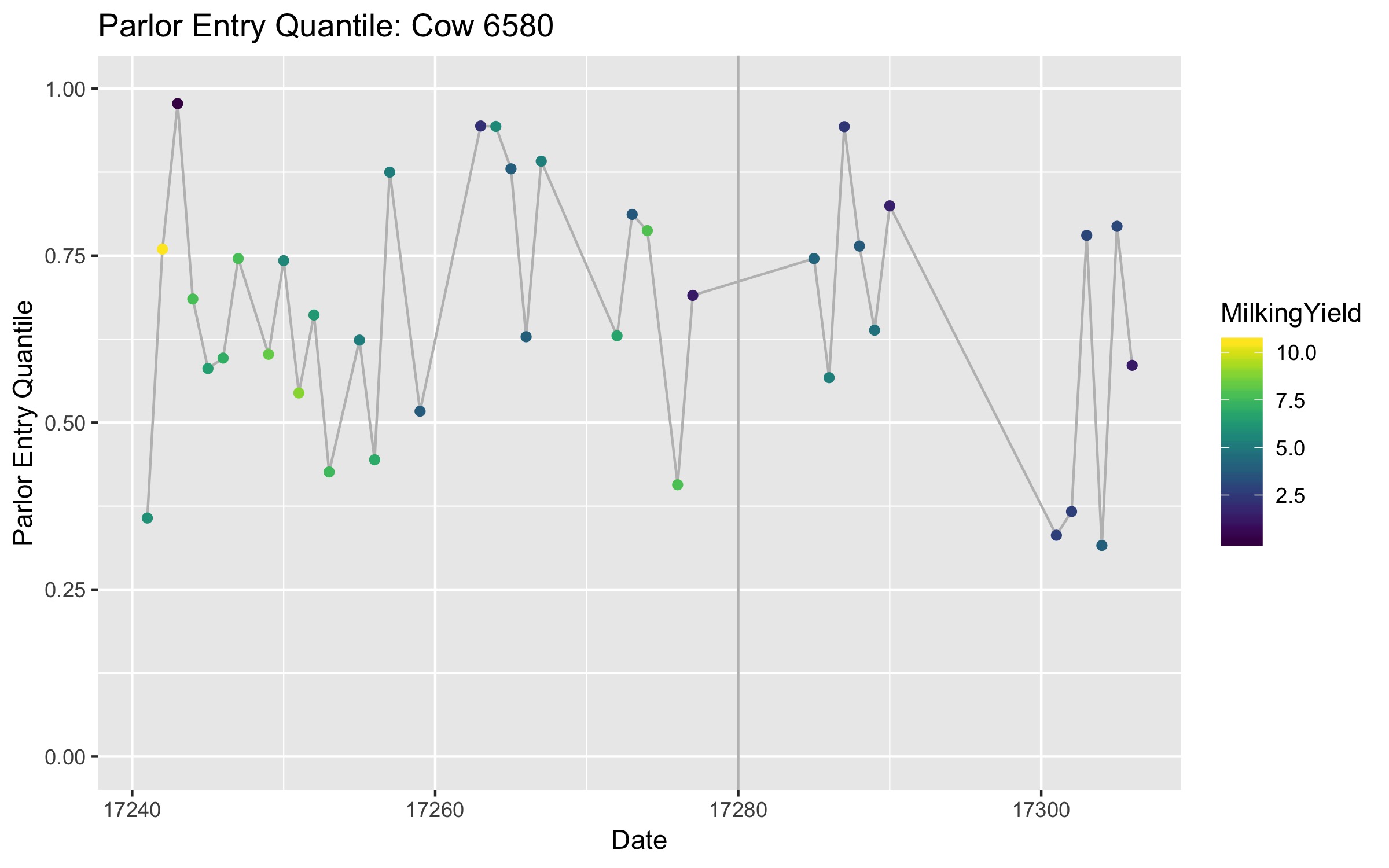

Supplement: Supplementary file 2 [file Data_Sheet_2.ZIP › Milking Yield/Cow_6580.jpg]

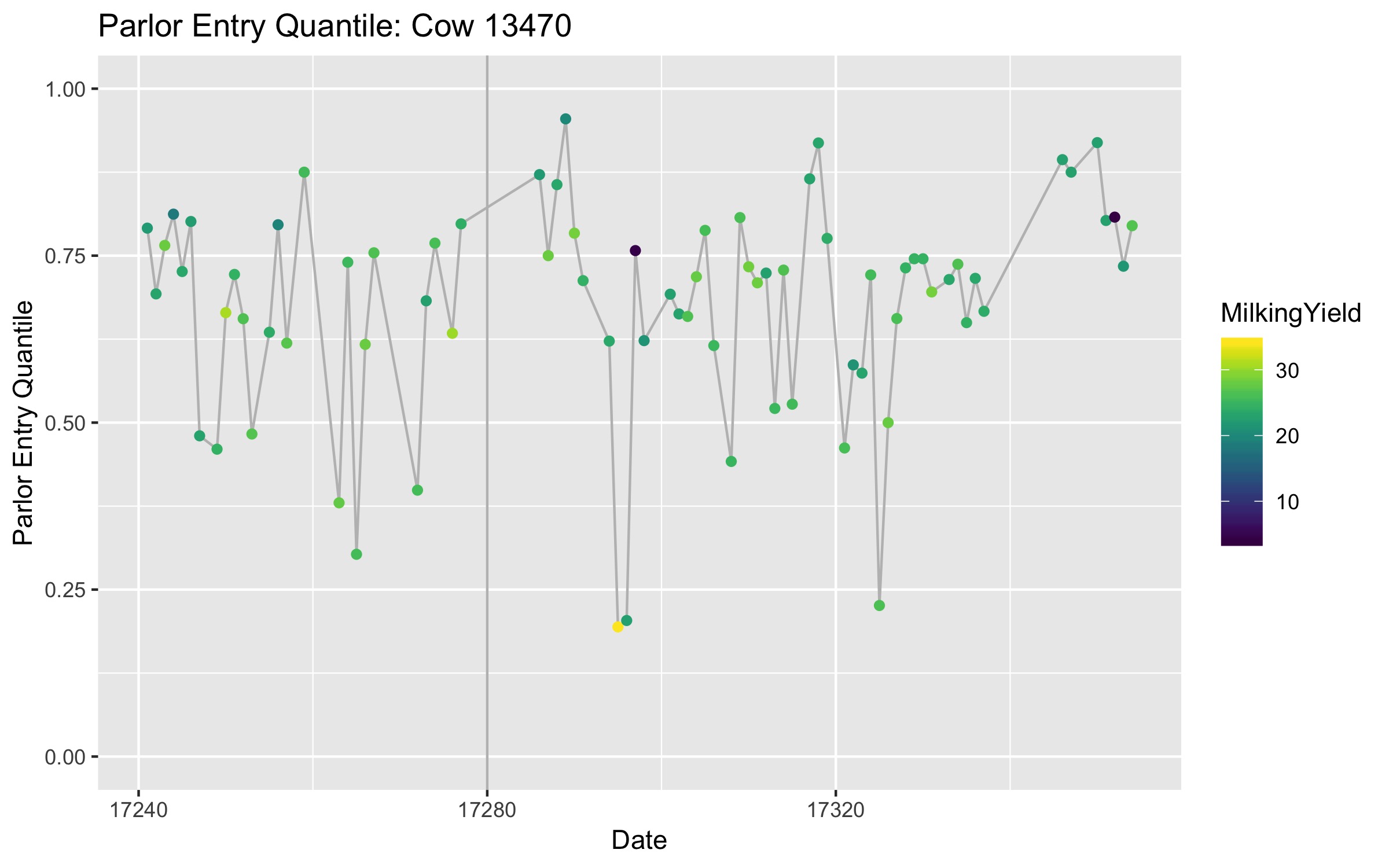

Supplement: Supplementary file 2 [file Data_Sheet_2.ZIP › Milking Yield/Cow_13470.jpg]

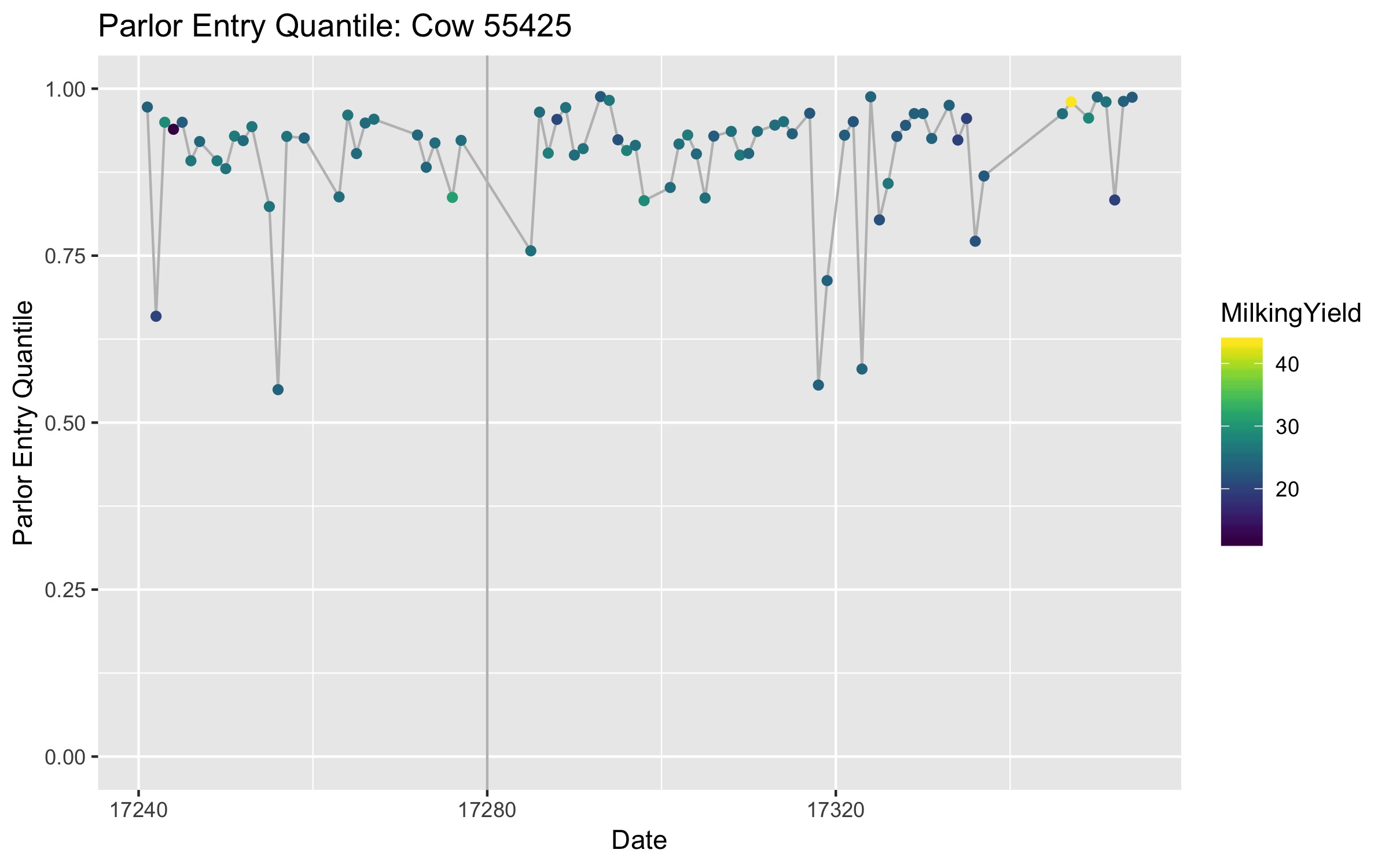

Supplement: Supplementary file 2 [file Data_Sheet_2.ZIP › Milking Yield/Cow_55425.jpg]

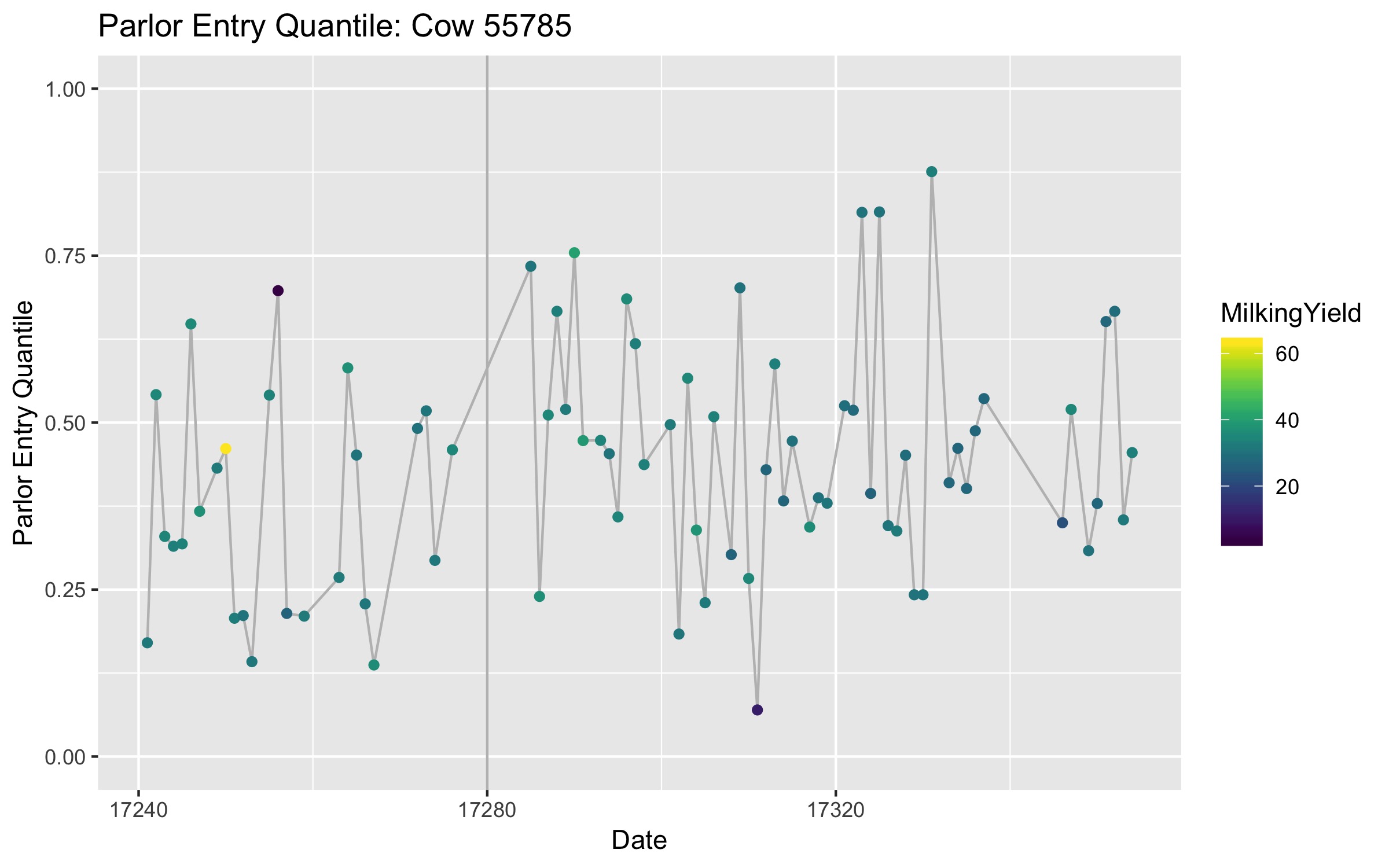

Supplement: Supplementary file 2 [file Data_Sheet_2.ZIP › Milking Yield/Cow_55785.jpg]

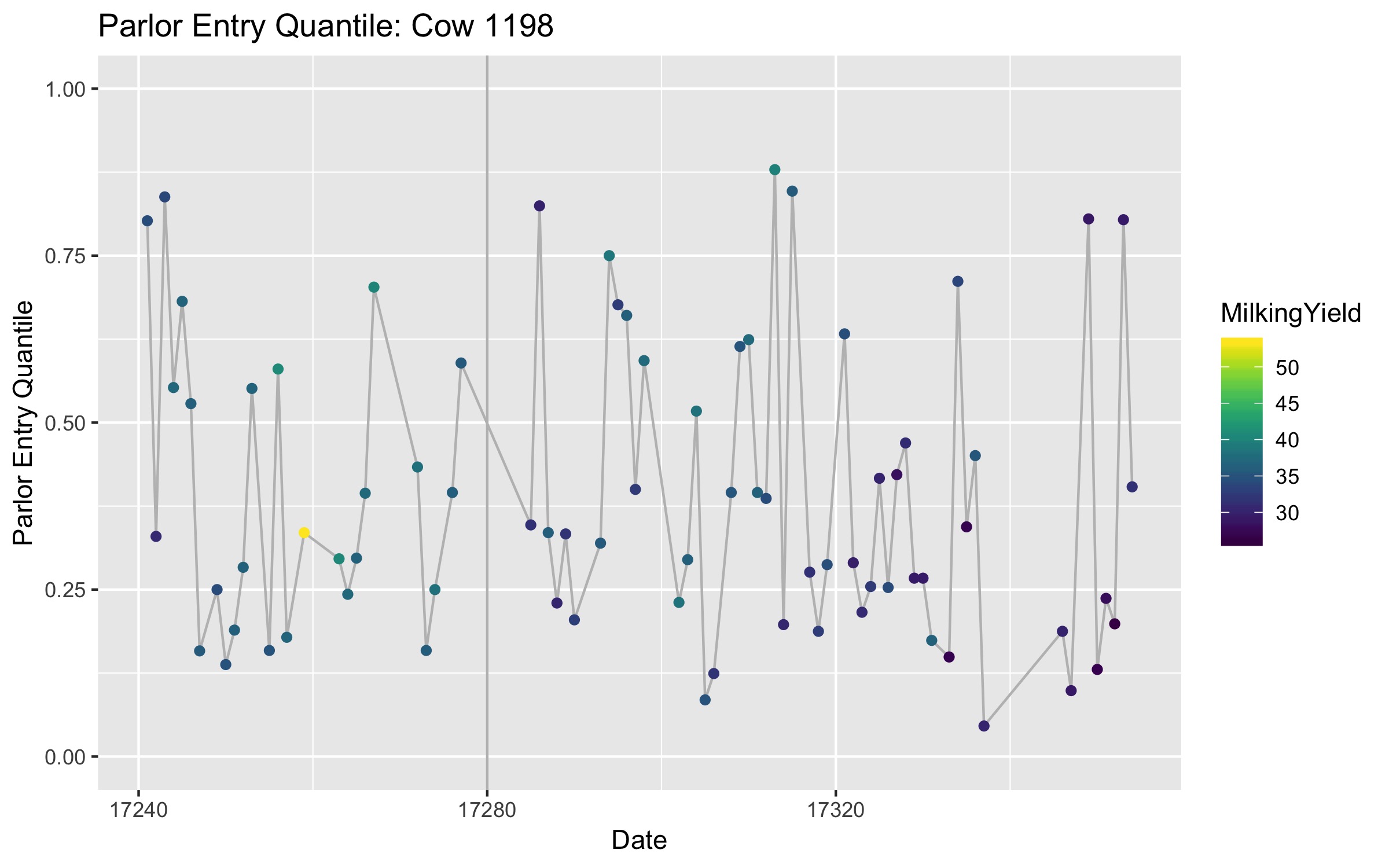

Supplement: Supplementary file 2 [file Data_Sheet_2.ZIP › Milking Yield/Cow_1198.jpg]

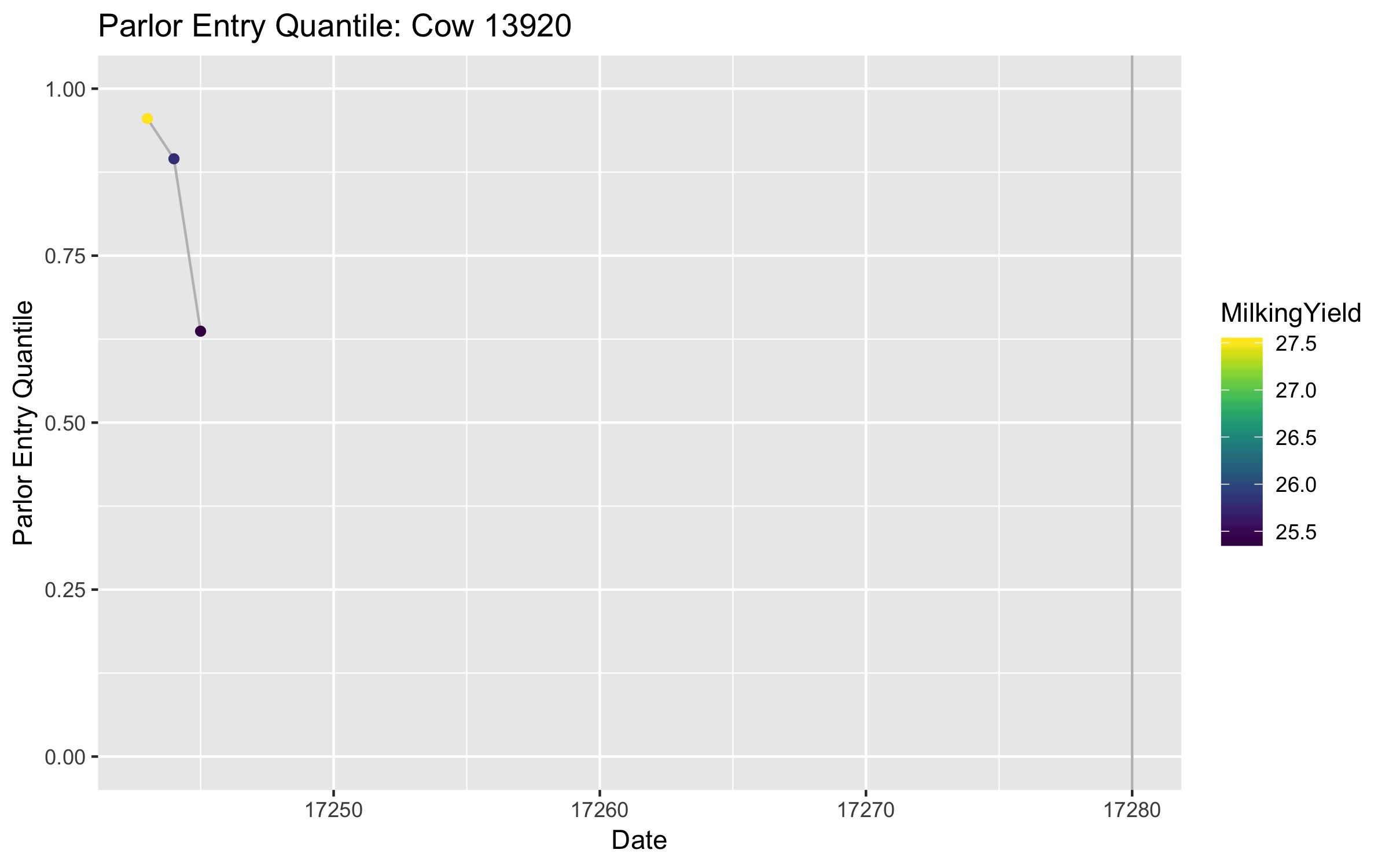

Supplement: Supplementary file 2 [file Data_Sheet_2.ZIP › Milking Yield/Cow_13920.jpg]

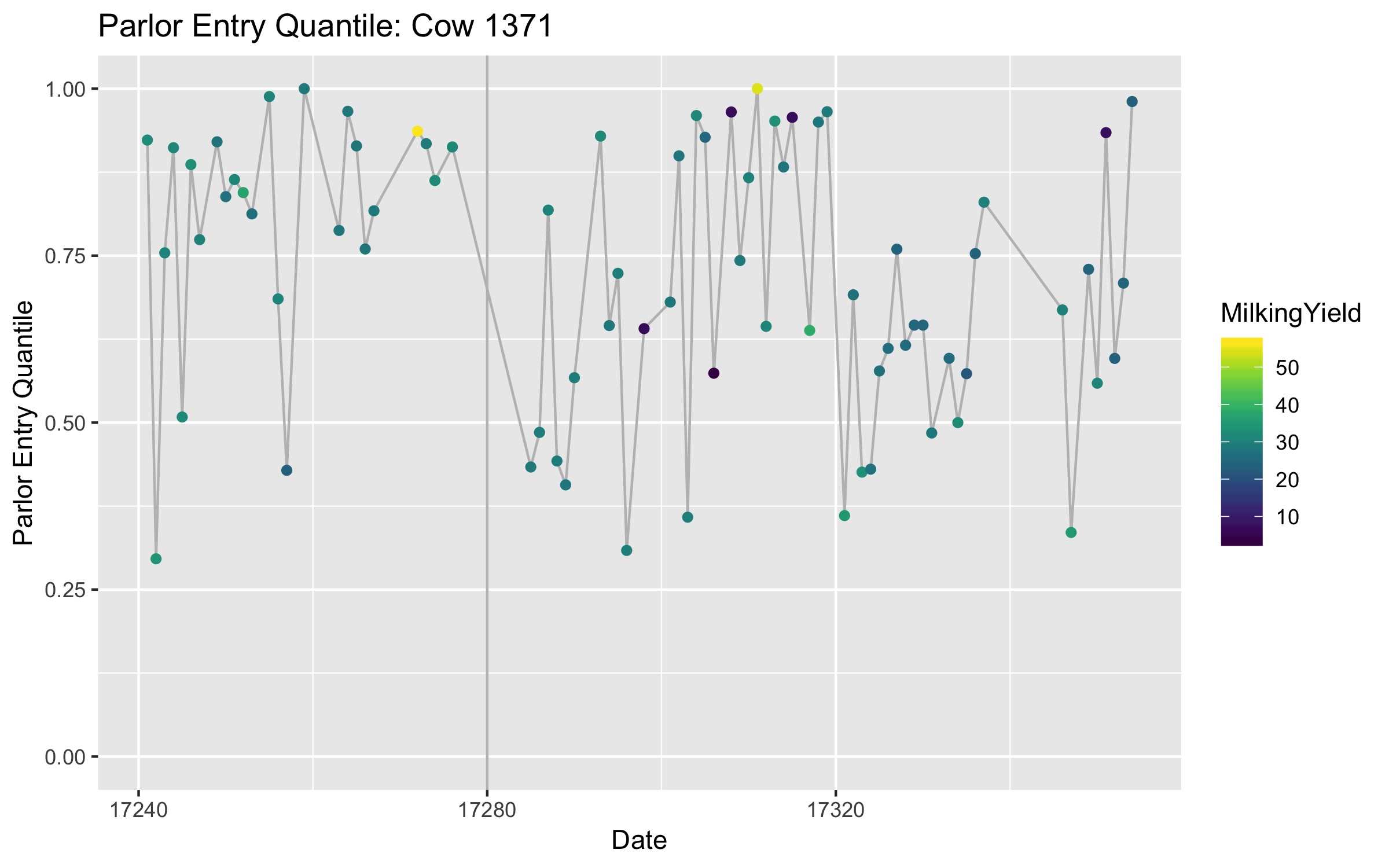

Supplement: Supplementary file 2 [file Data_Sheet_2.ZIP › Milking Yield/Cow_1371.jpg]

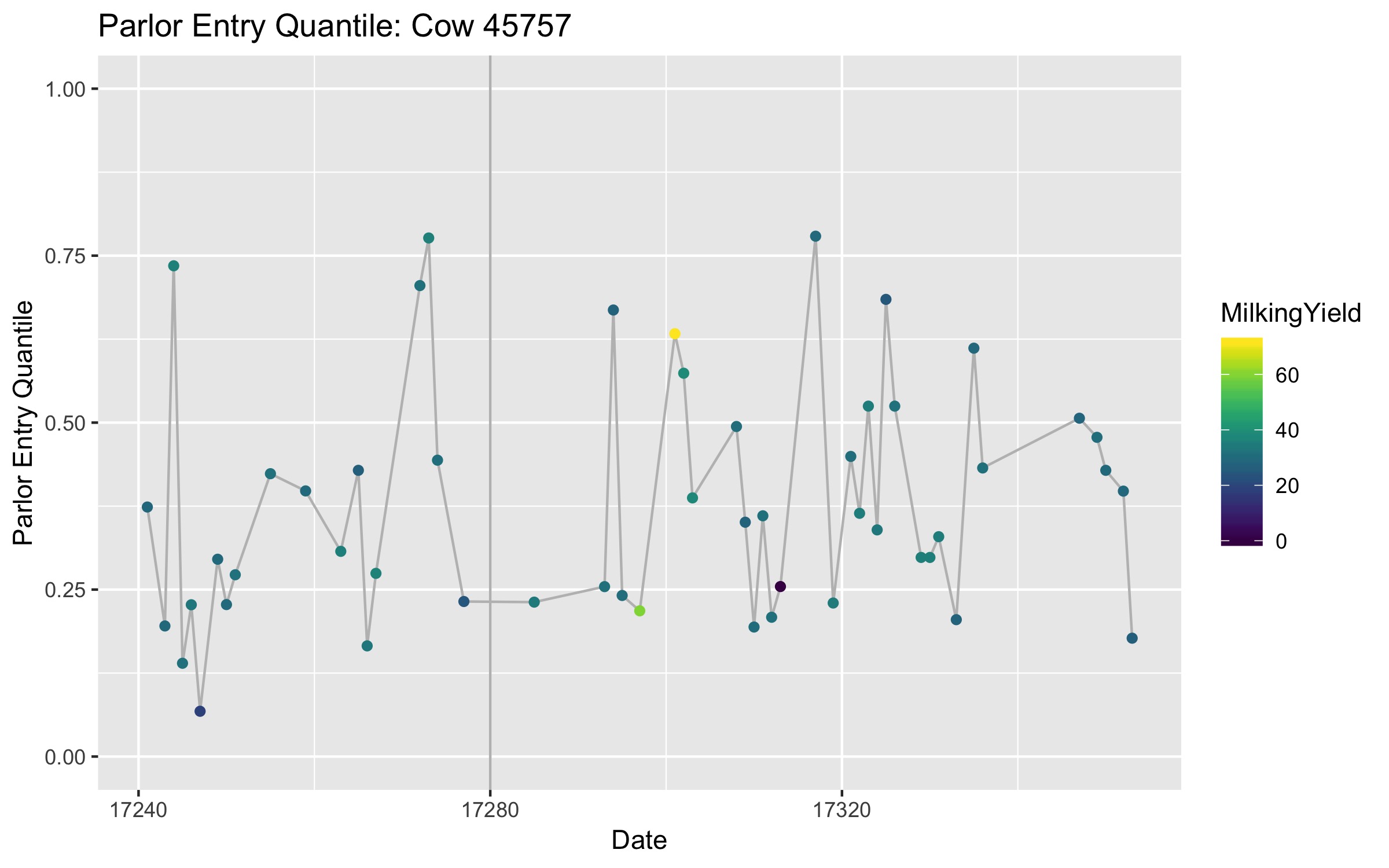

Supplement: Supplementary file 2 [file Data_Sheet_2.ZIP › Milking Yield/Cow_45757.jpg]

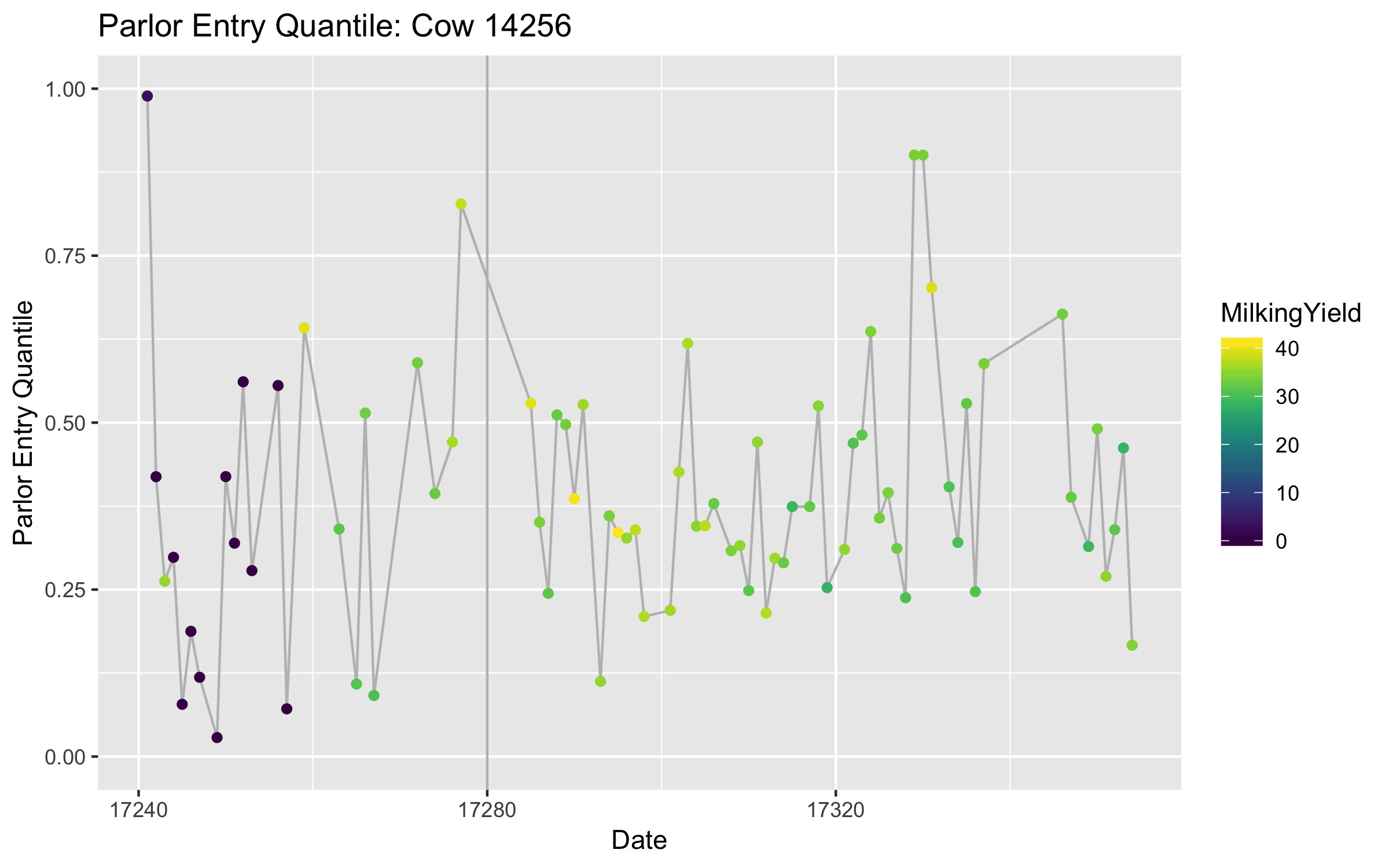

Supplement: Supplementary file 2 [file Data_Sheet_2.ZIP › Milking Yield/Cow_14256.jpg]

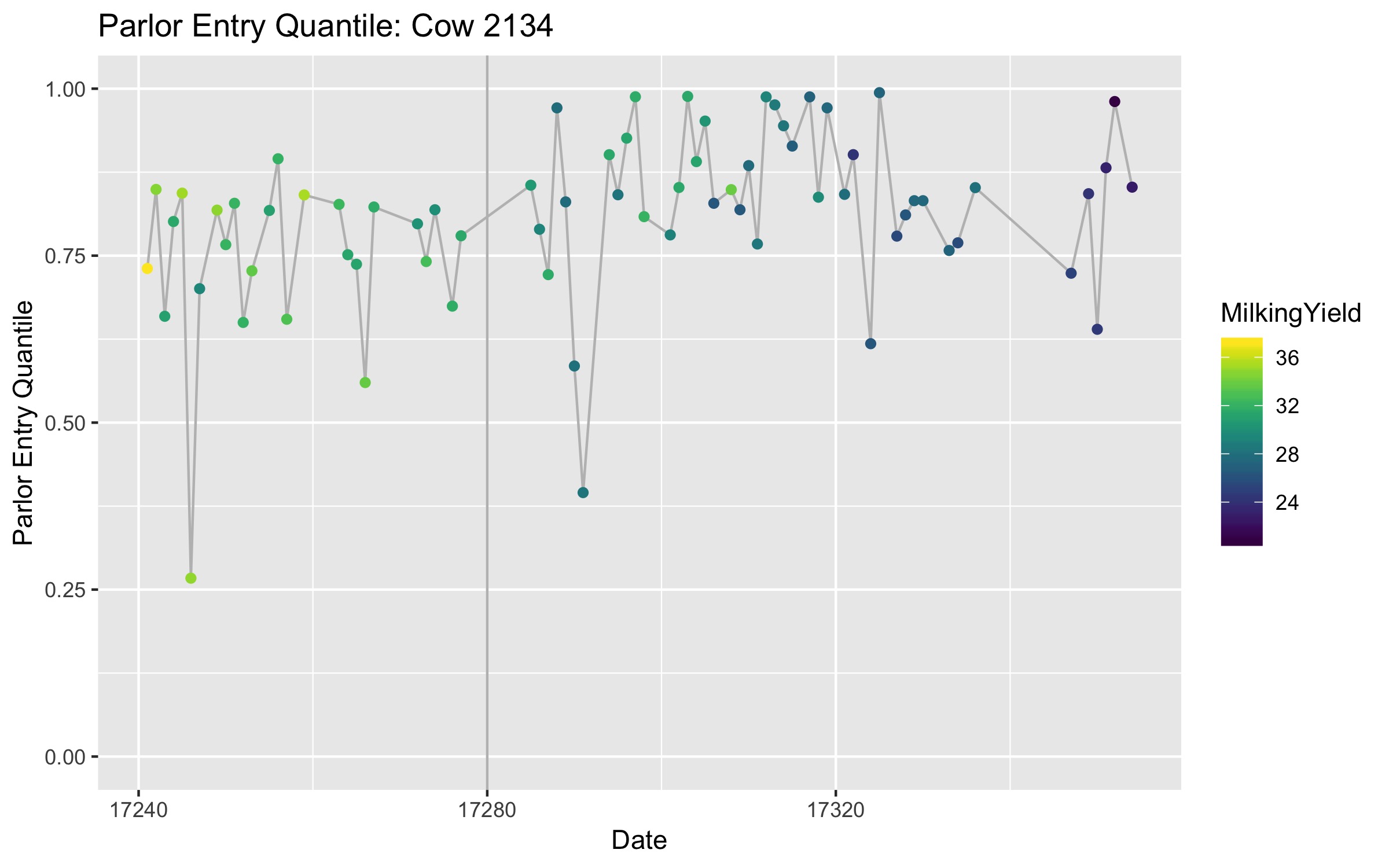

Supplement: Supplementary file 2 [file Data_Sheet_2.ZIP › Milking Yield/Cow_2134.jpg]

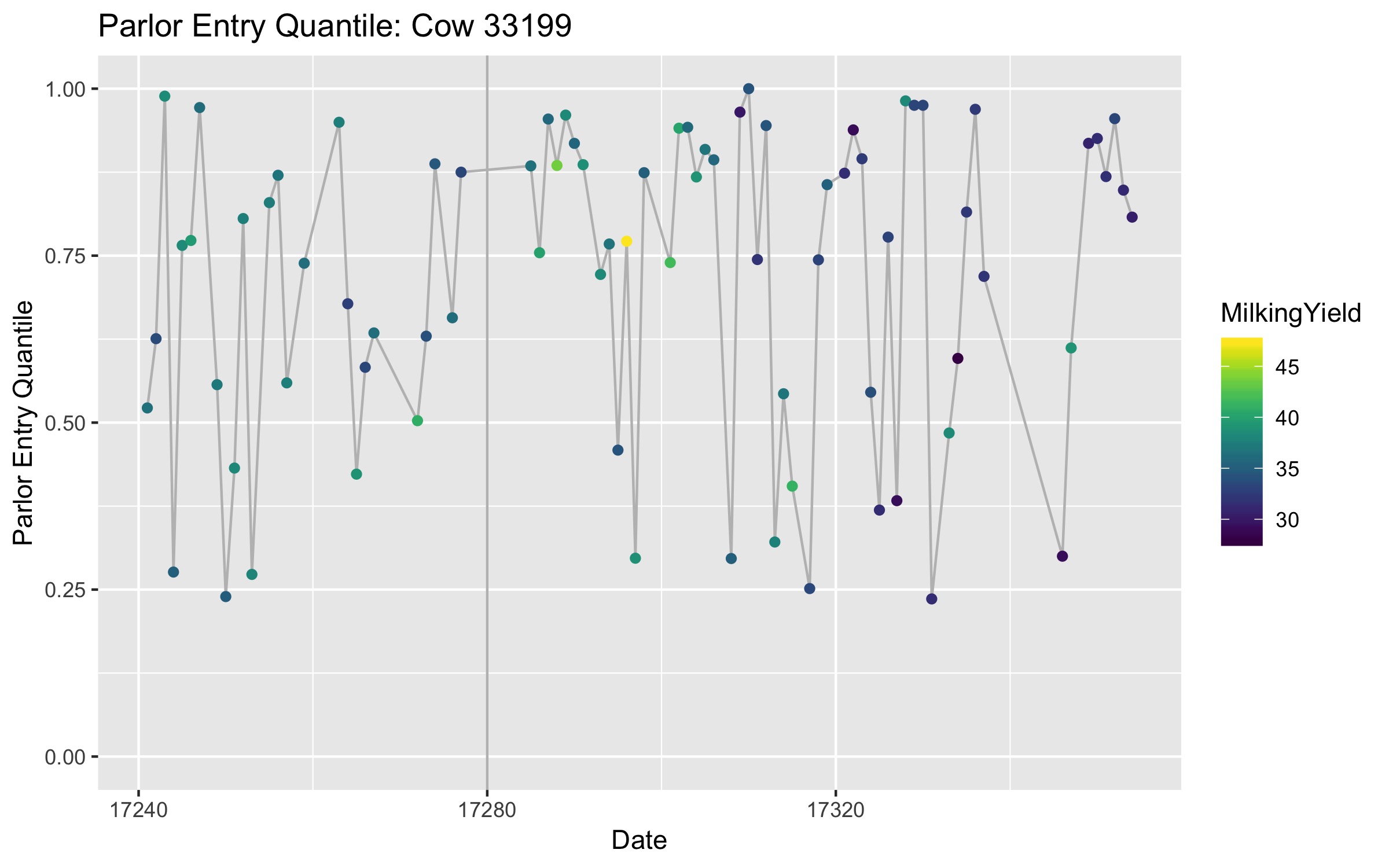

Supplement: Supplementary file 2 [file Data_Sheet_2.ZIP › Milking Yield/Cow_33199.jpg]

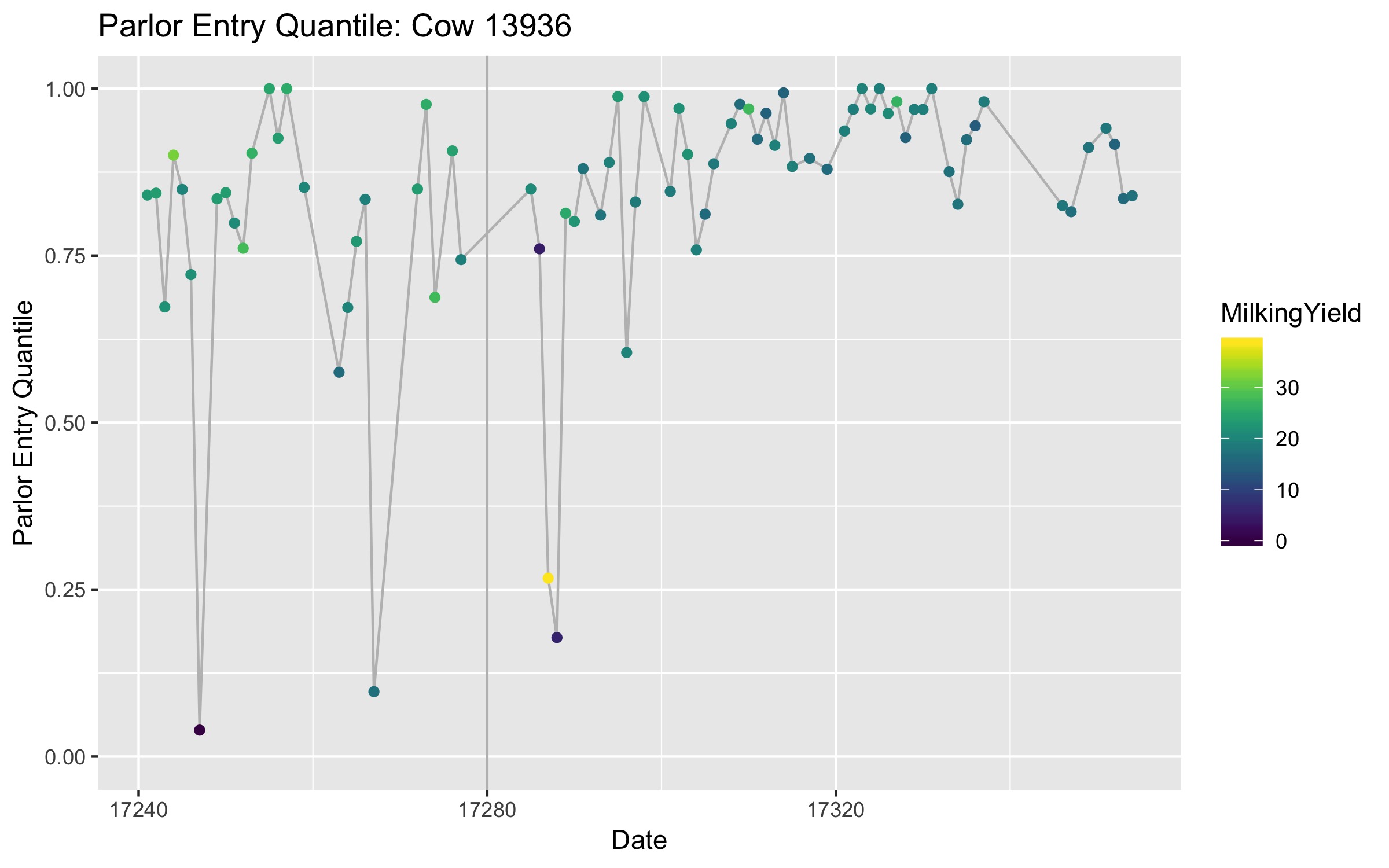

Supplement: Supplementary file 2 [file Data_Sheet_2.ZIP › Milking Yield/Cow_13936.jpg]

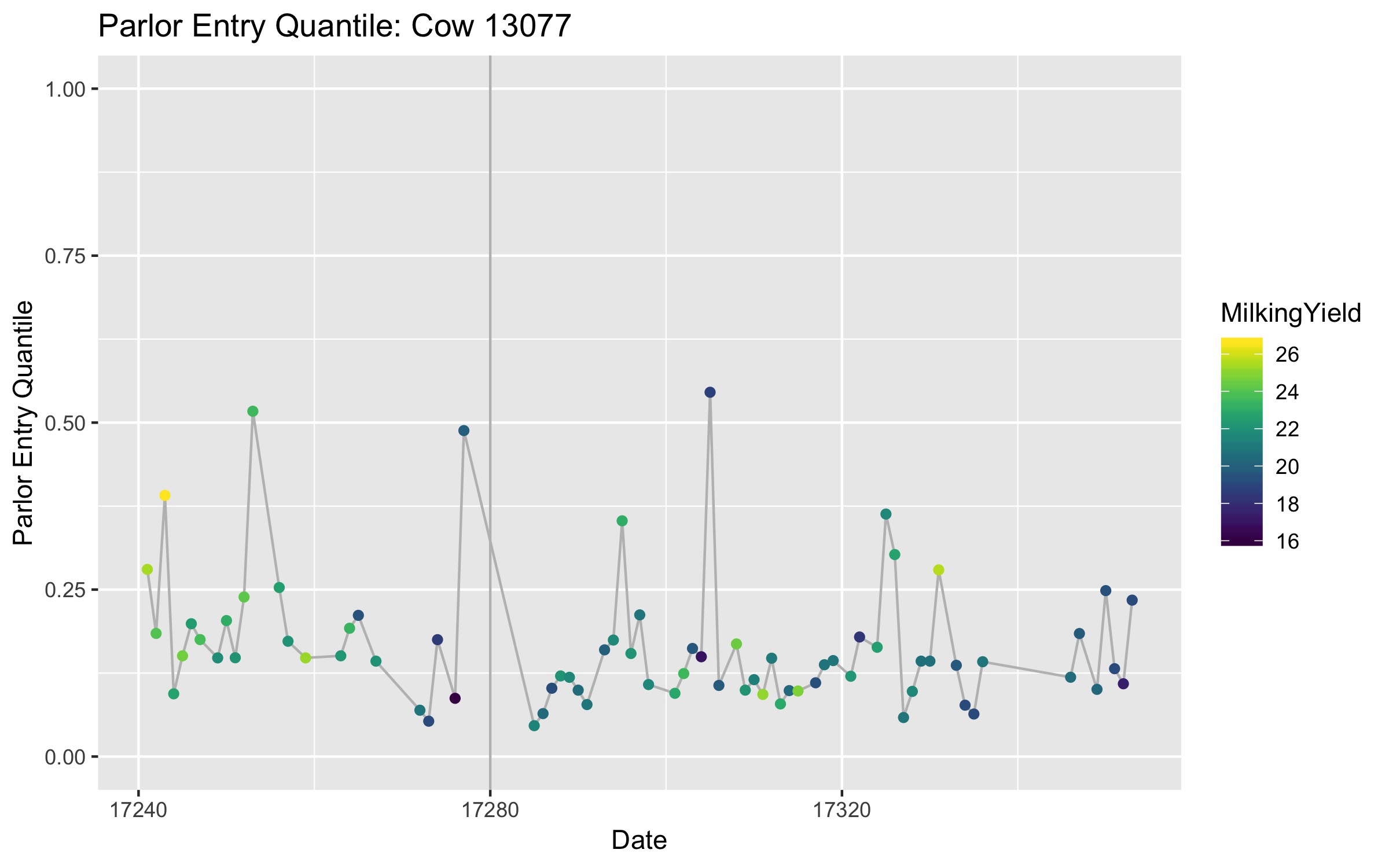

Supplement: Supplementary file 2 [file Data_Sheet_2.ZIP › Milking Yield/Cow_13077.jpg]

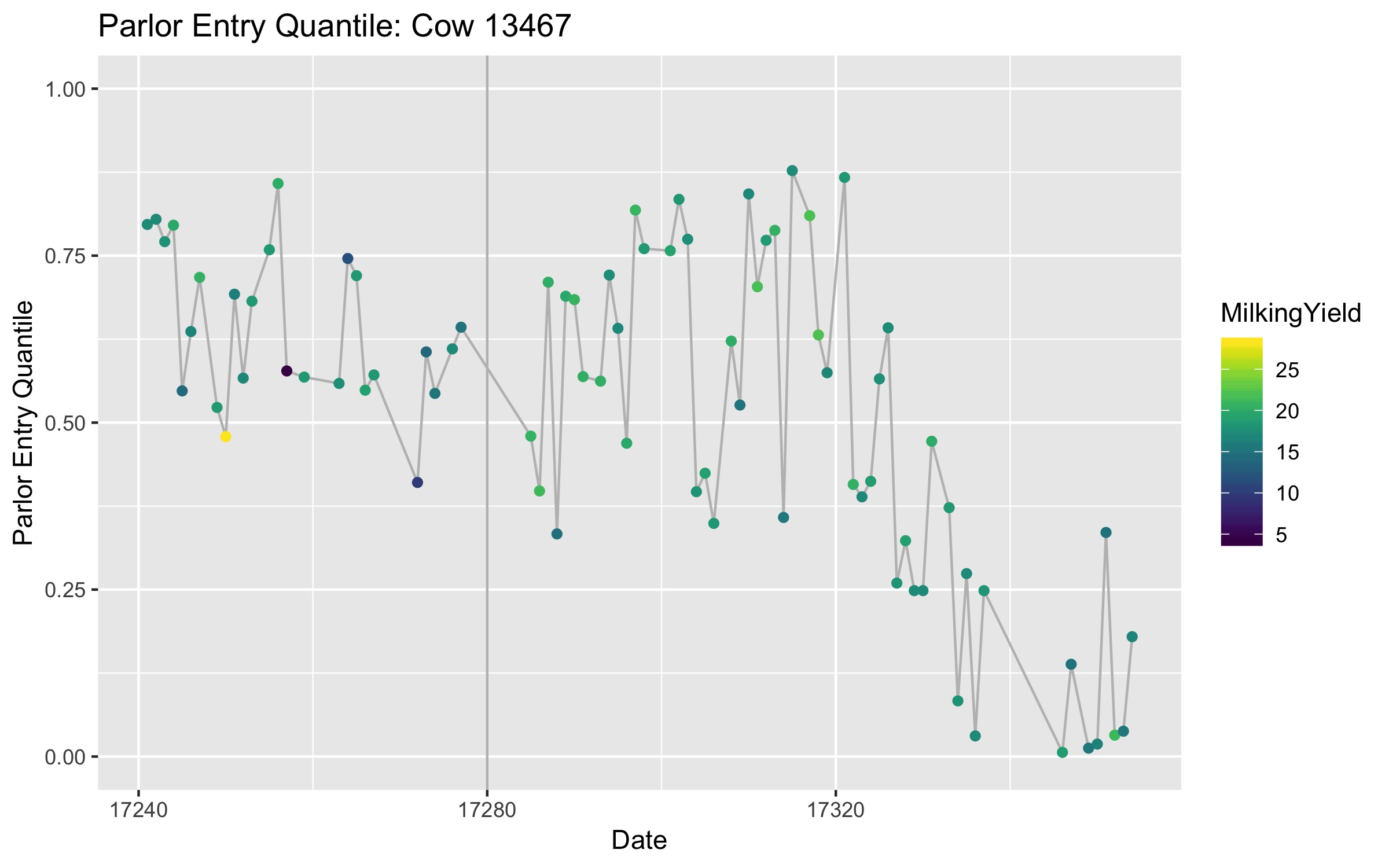

Supplement: Supplementary file 2 [file Data_Sheet_2.ZIP › Milking Yield/Cow_13467.jpg]

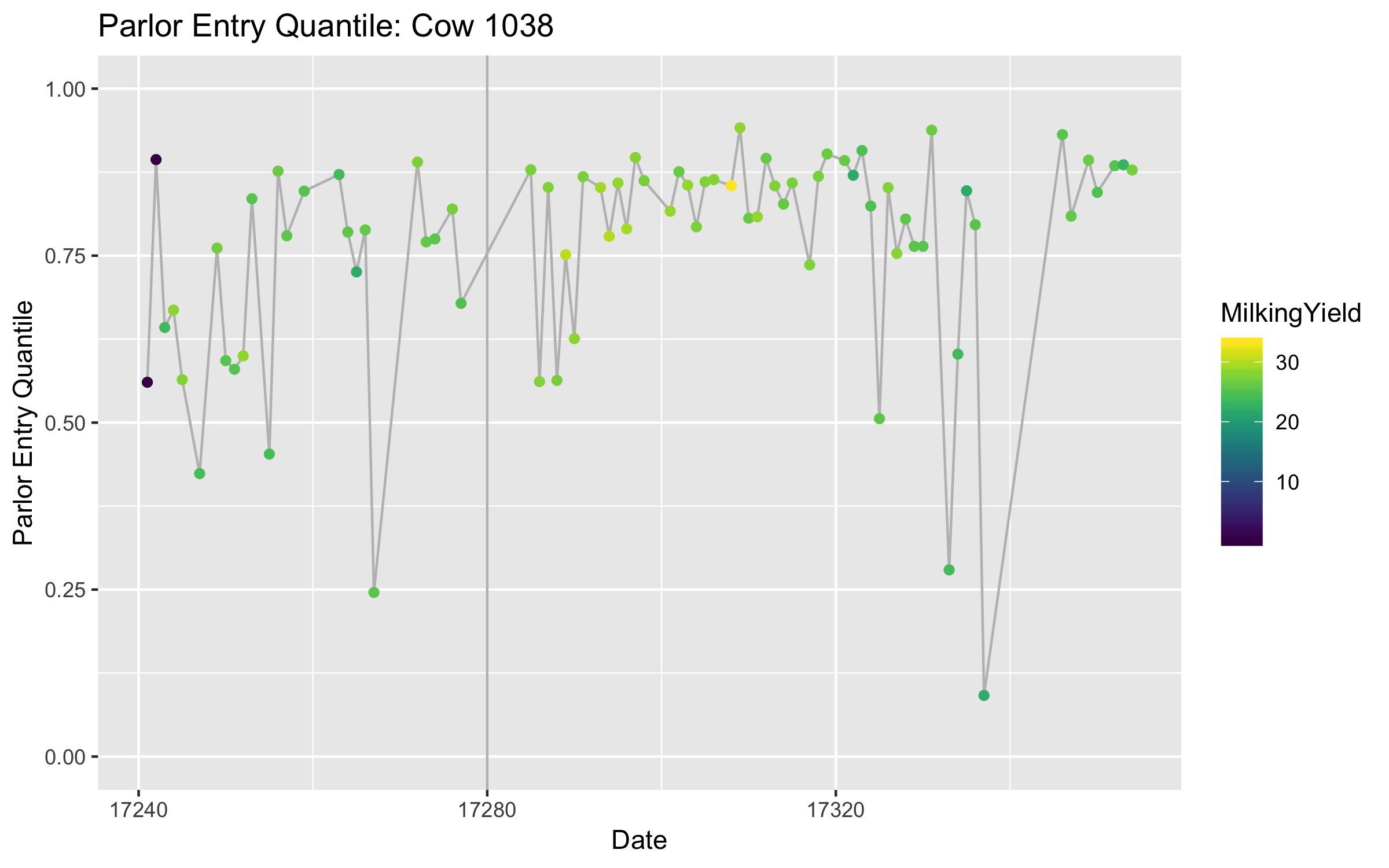

Supplement: Supplementary file 2 [file Data_Sheet_2.ZIP › Milking Yield/Cow_1038.jpg]

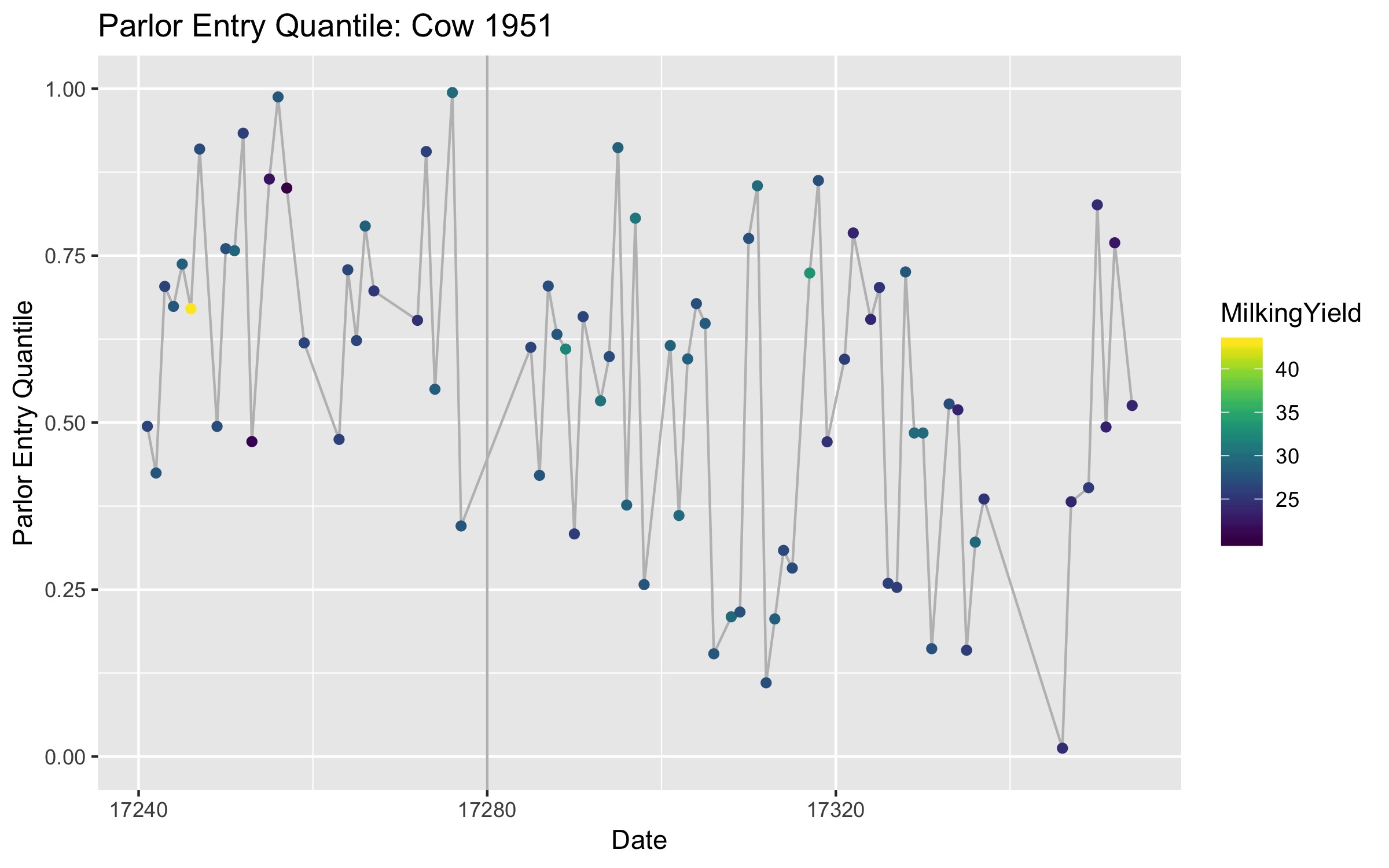

Supplement: Supplementary file 2 [file Data_Sheet_2.ZIP › Milking Yield/Cow_1951.jpg]

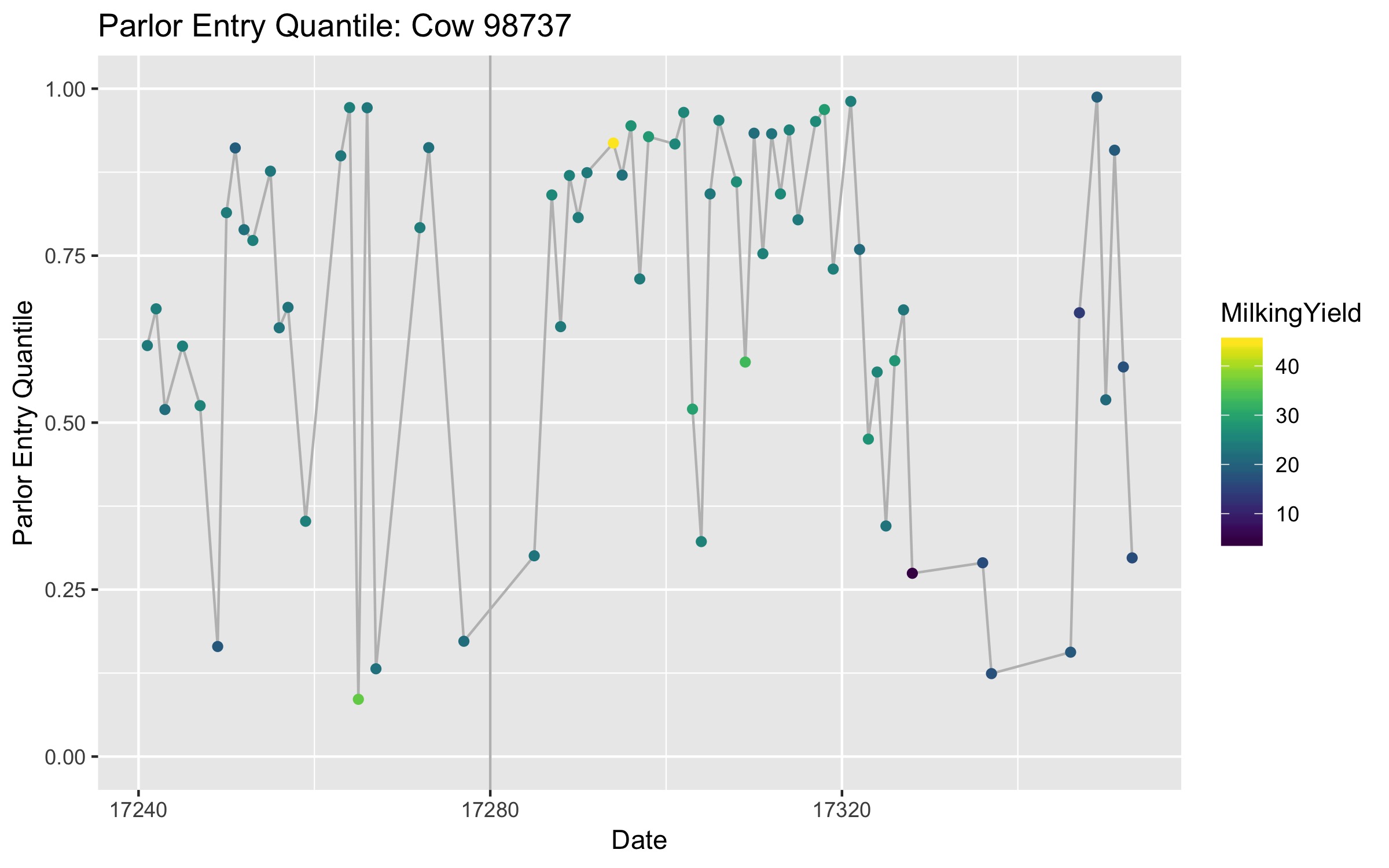

Supplement: Supplementary file 2 [file Data_Sheet_2.ZIP › Milking Yield/Cow_98737.jpg]

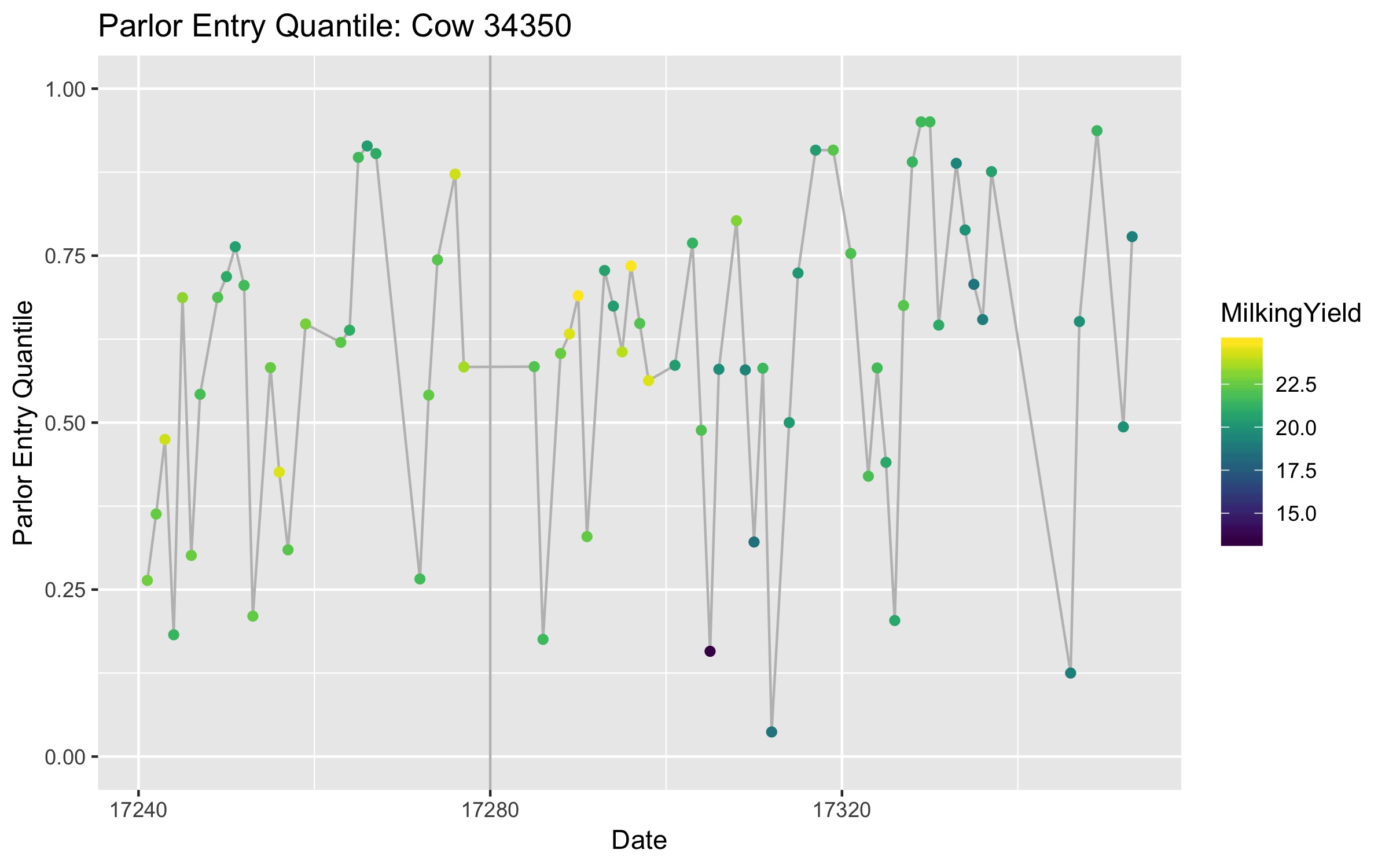

Supplement: Supplementary file 2 [file Data_Sheet_2.ZIP › Milking Yield/Cow_34350.jpg]

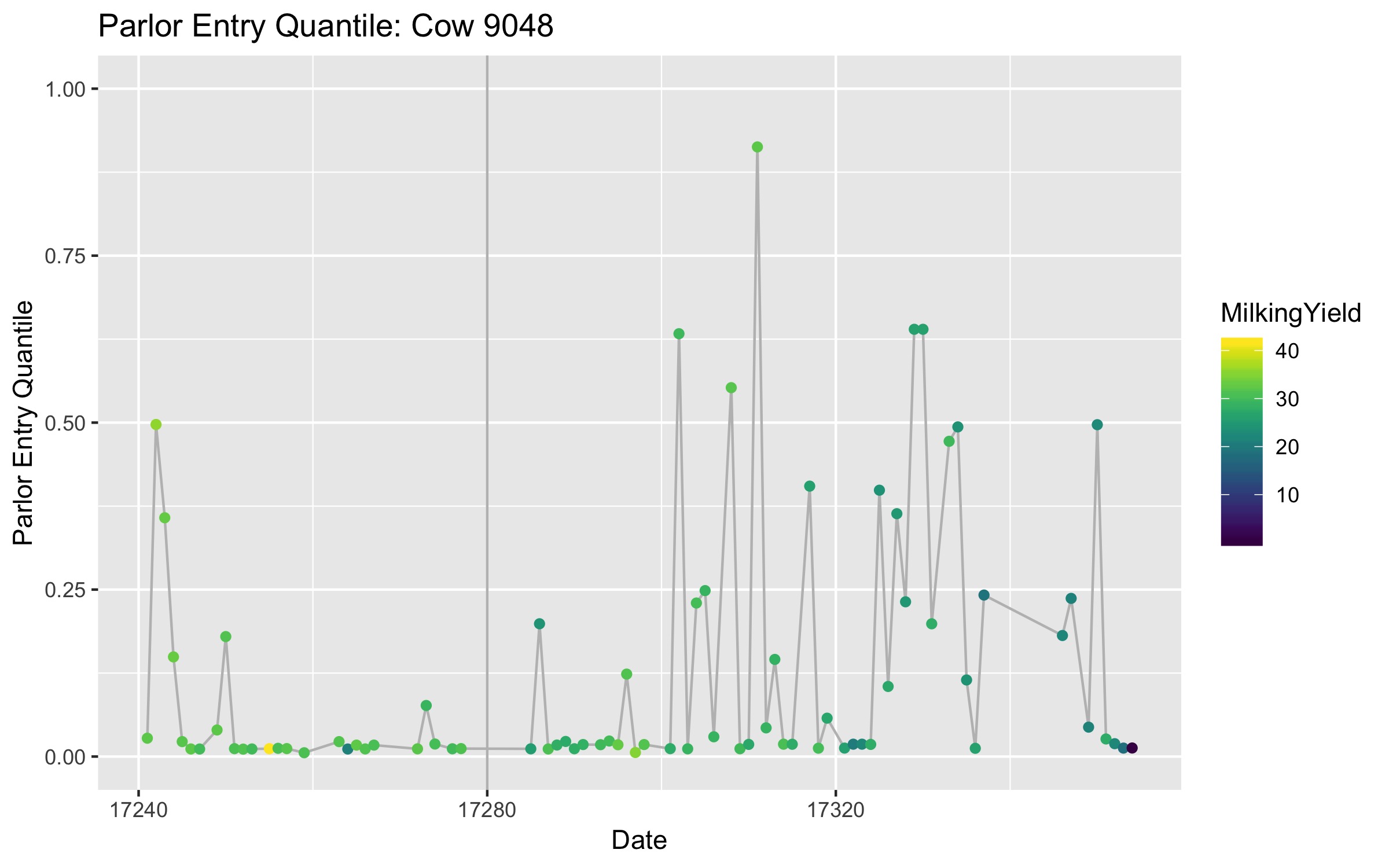

Supplement: Supplementary file 2 [file Data_Sheet_2.ZIP › Milking Yield/Cow_9048.jpg]

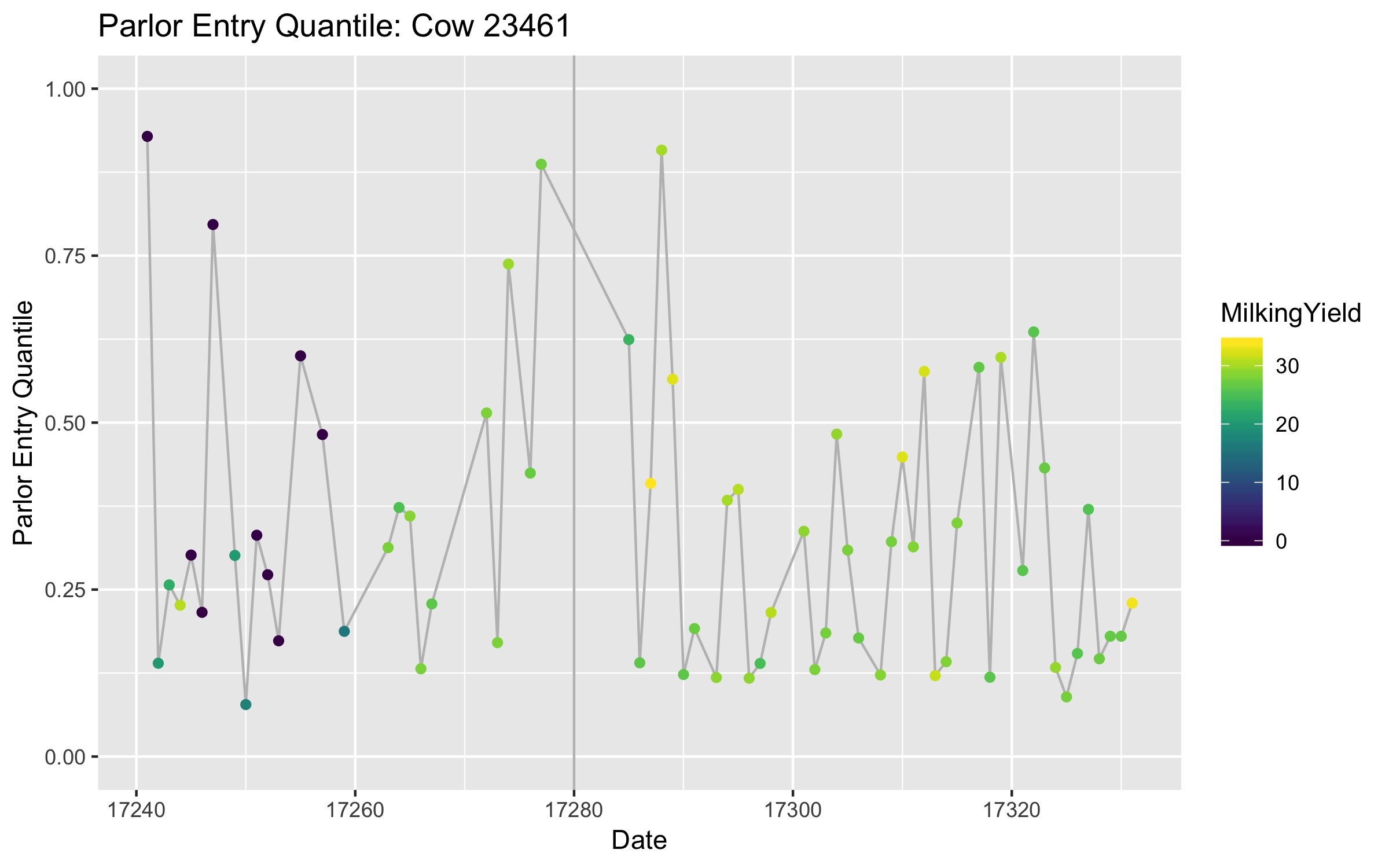

Supplement: Supplementary file 2 [file Data_Sheet_2.ZIP › Milking Yield/Cow_23461.jpg]

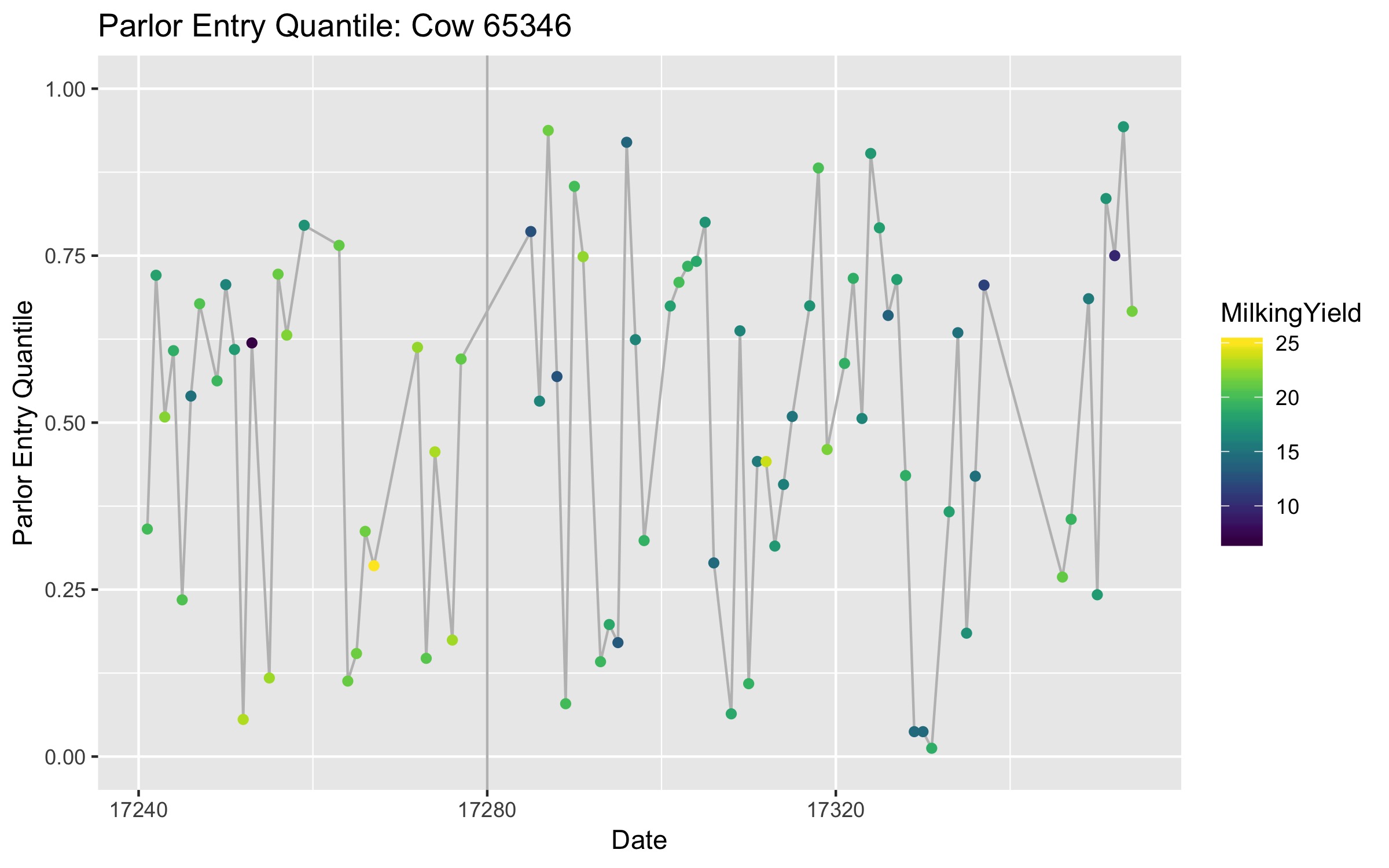

Supplement: Supplementary file 2 [file Data_Sheet_2.ZIP › Milking Yield/Cow_65346.jpg]

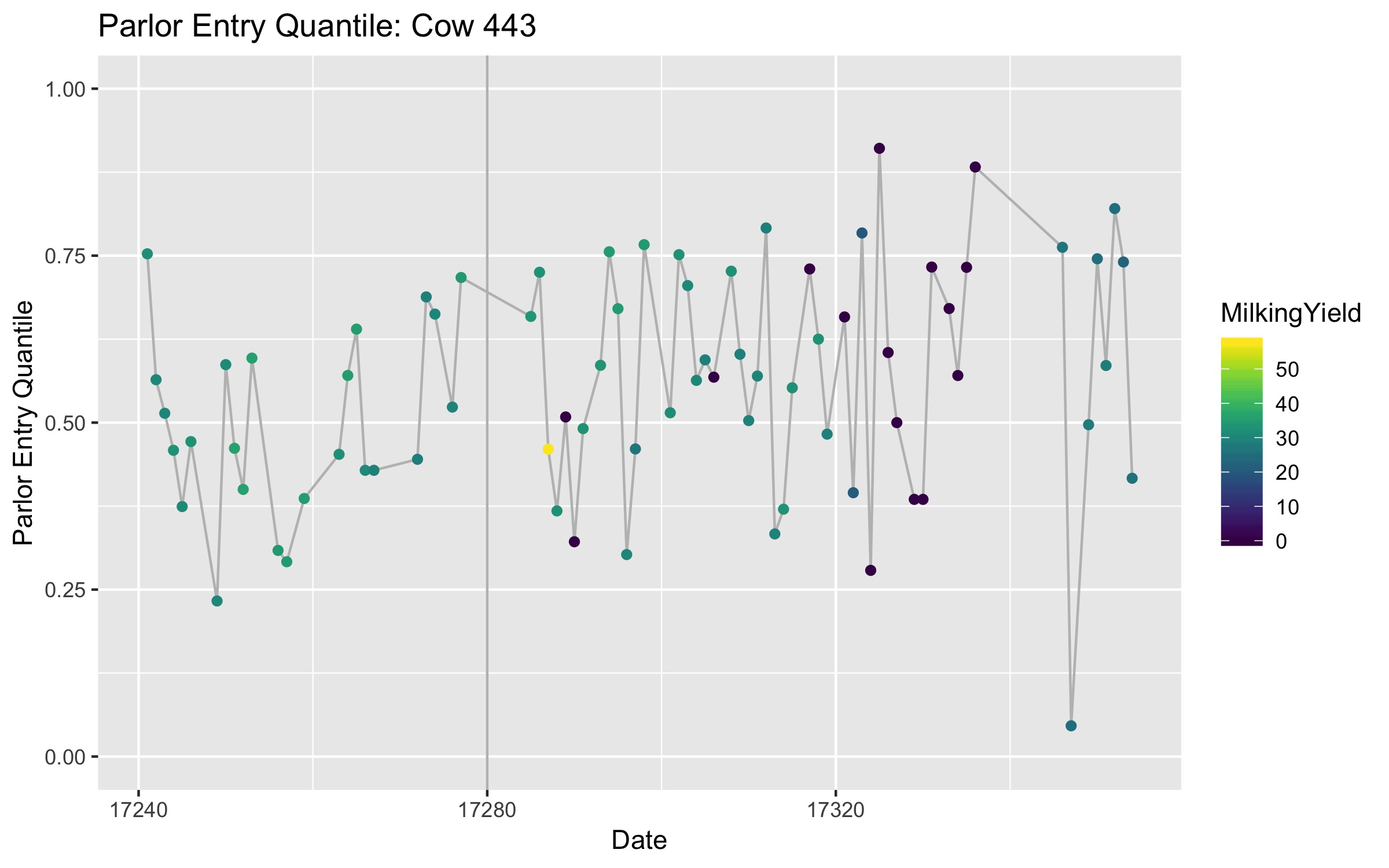

Supplement: Supplementary file 2 [file Data_Sheet_2.ZIP › Milking Yield/Cow_443.jpg]

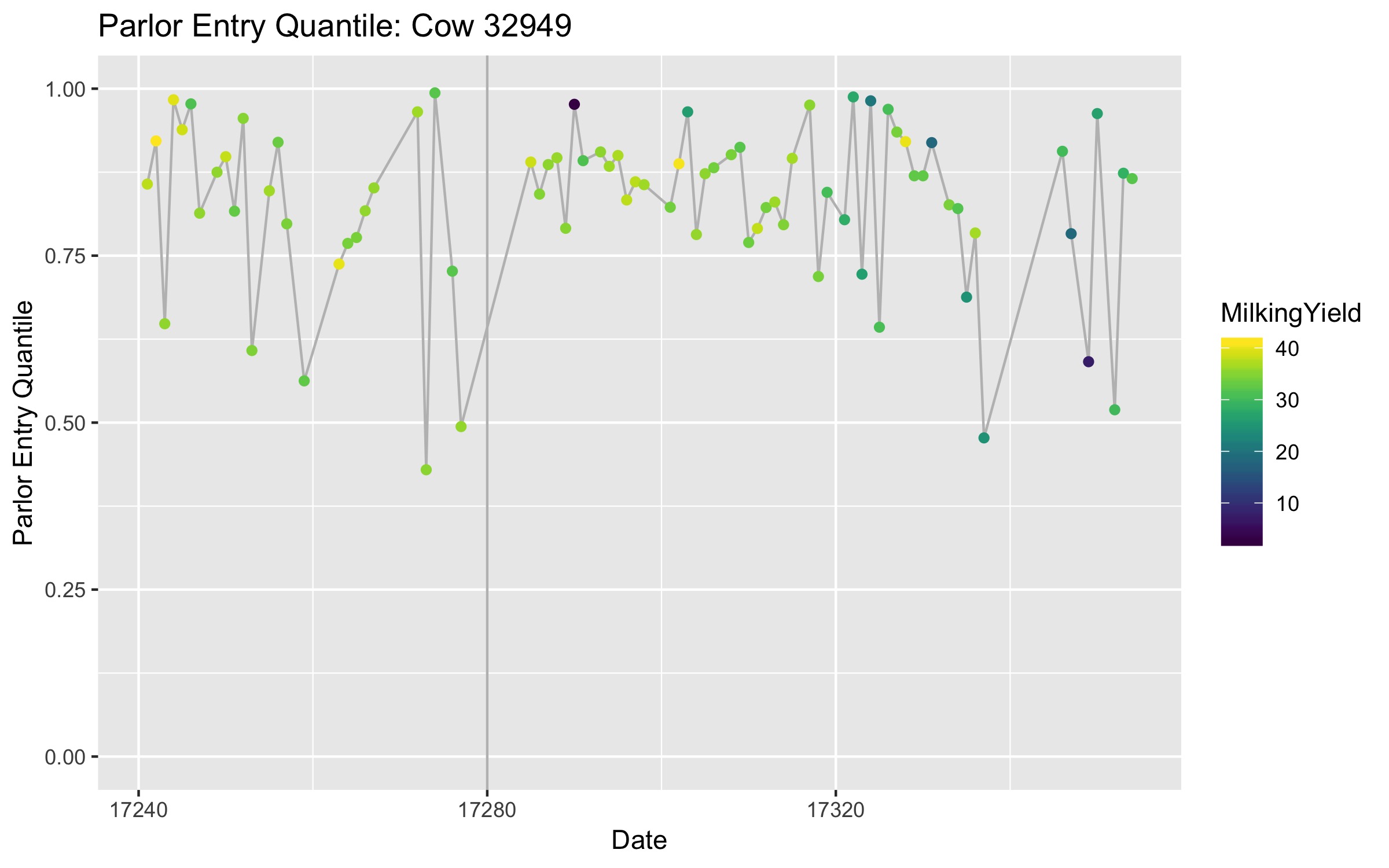

Supplement: Supplementary file 2 [file Data_Sheet_2.ZIP › Milking Yield/Cow_32949.jpg]

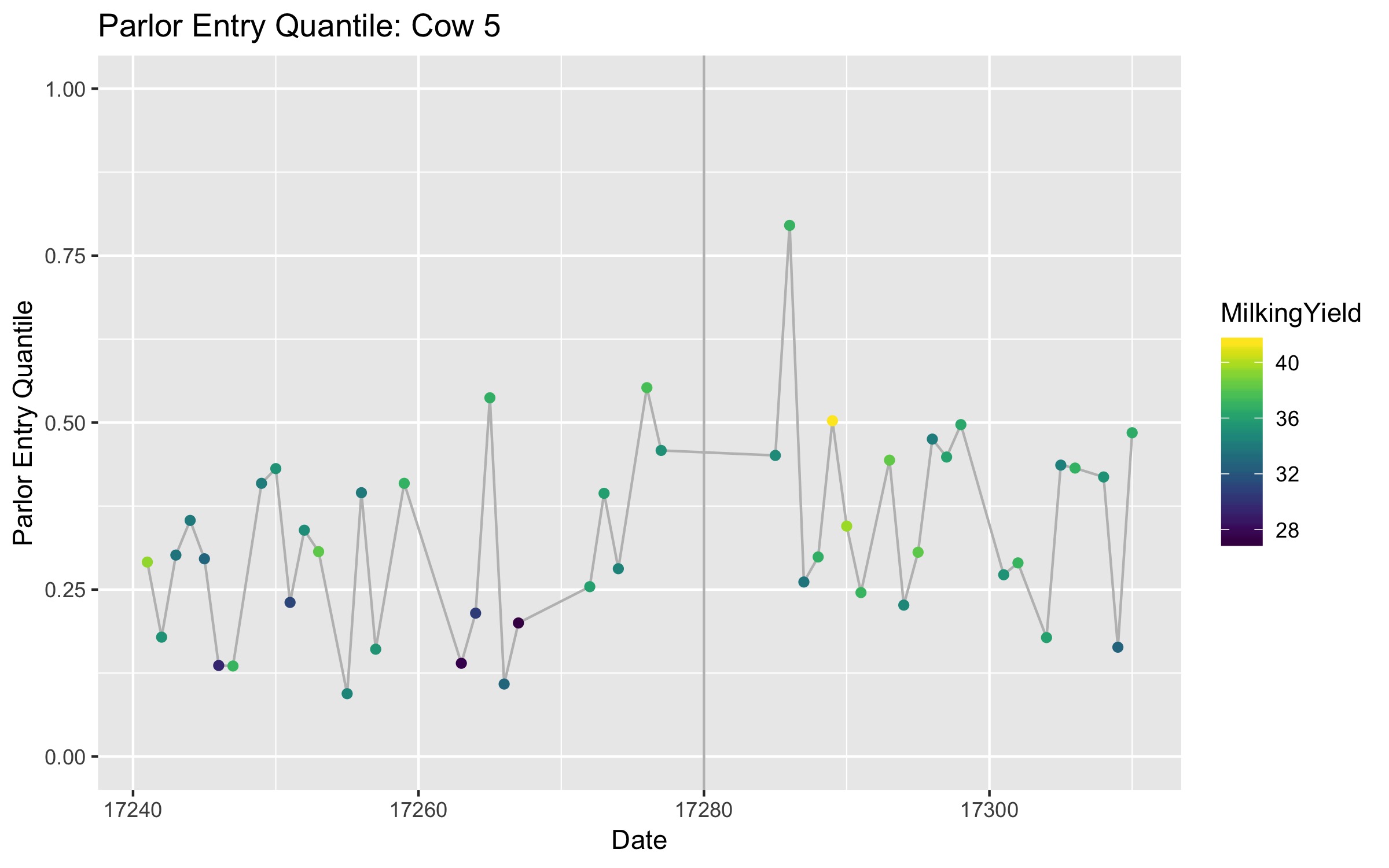

Supplement: Supplementary file 2 [file Data_Sheet_2.ZIP › Milking Yield/Cow_5.jpg]

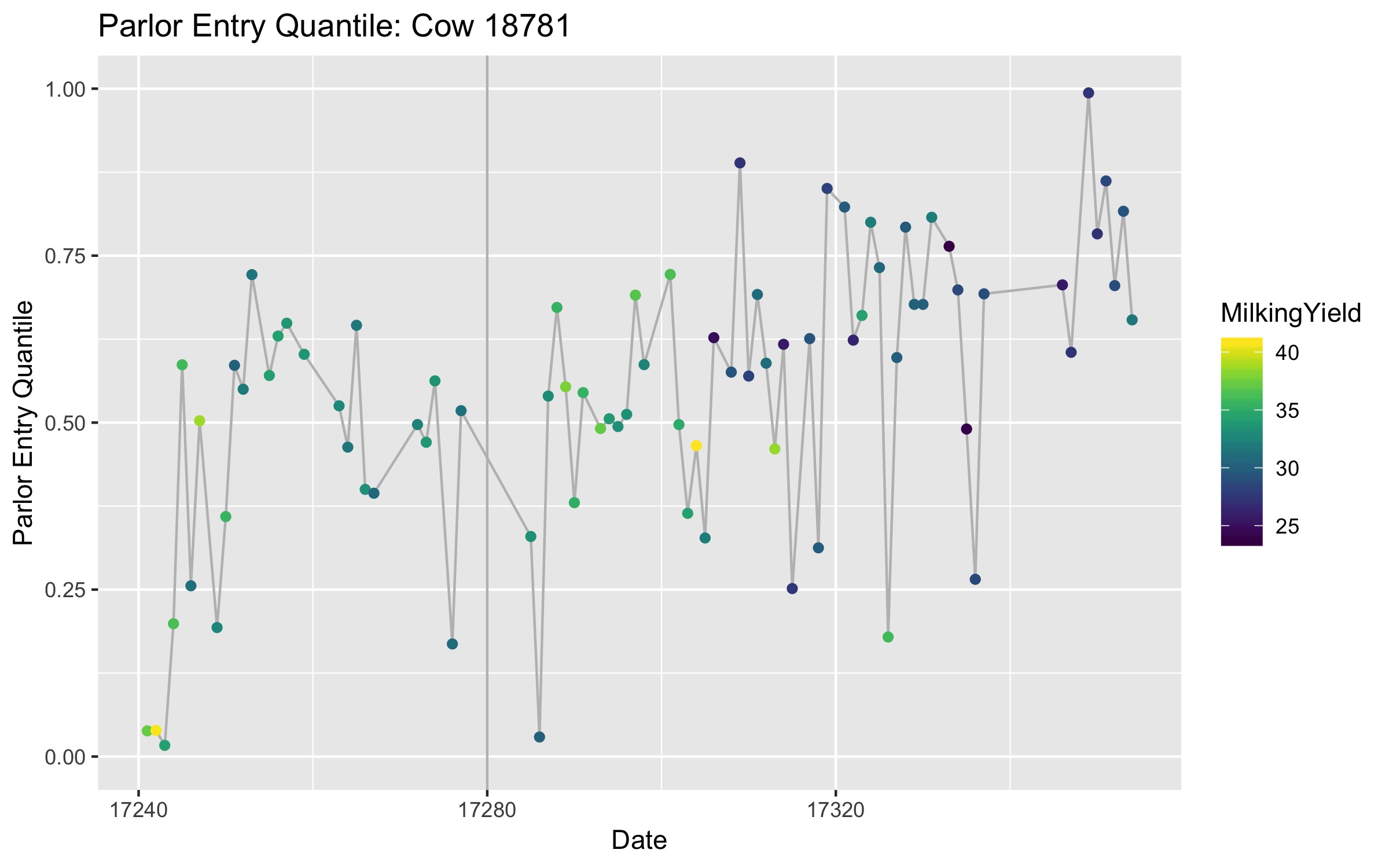

Supplement: Supplementary file 2 [file Data_Sheet_2.ZIP › Milking Yield/Cow_18781.jpg]

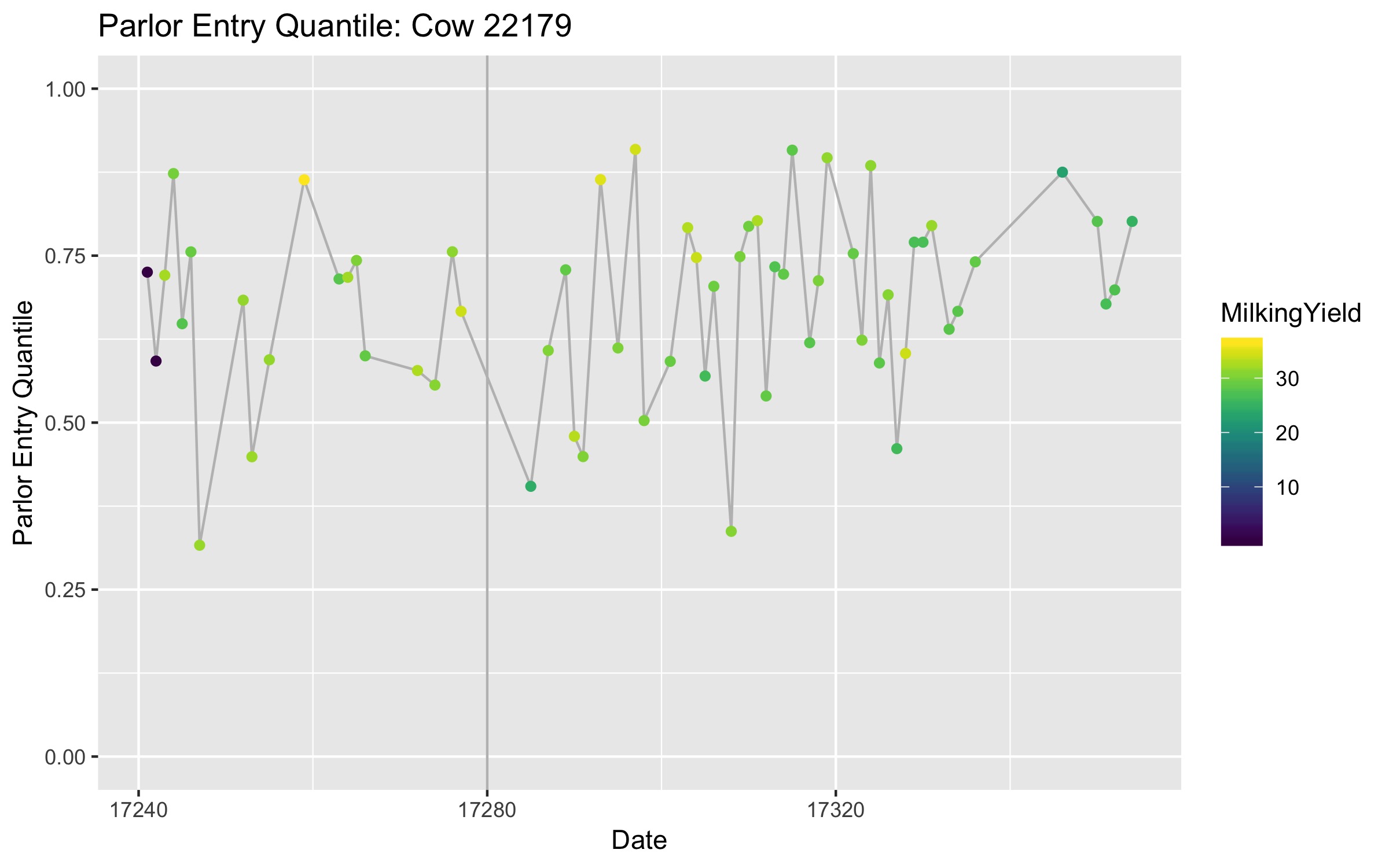

Supplement: Supplementary file 2 [file Data_Sheet_2.ZIP › Milking Yield/Cow_22179.jpg]

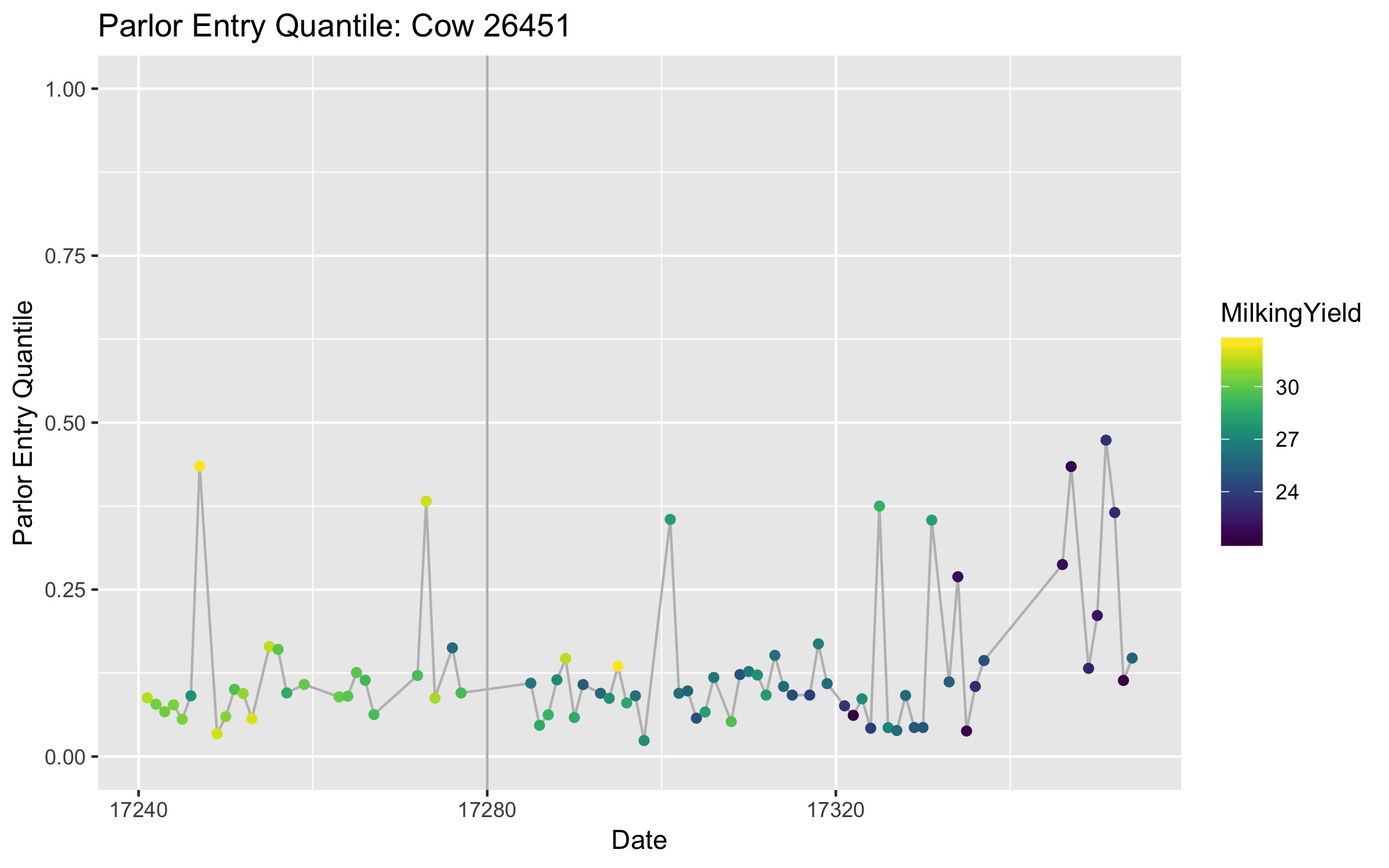

Supplement: Supplementary file 2 [file Data_Sheet_2.ZIP › Milking Yield/Cow_26451.jpg]

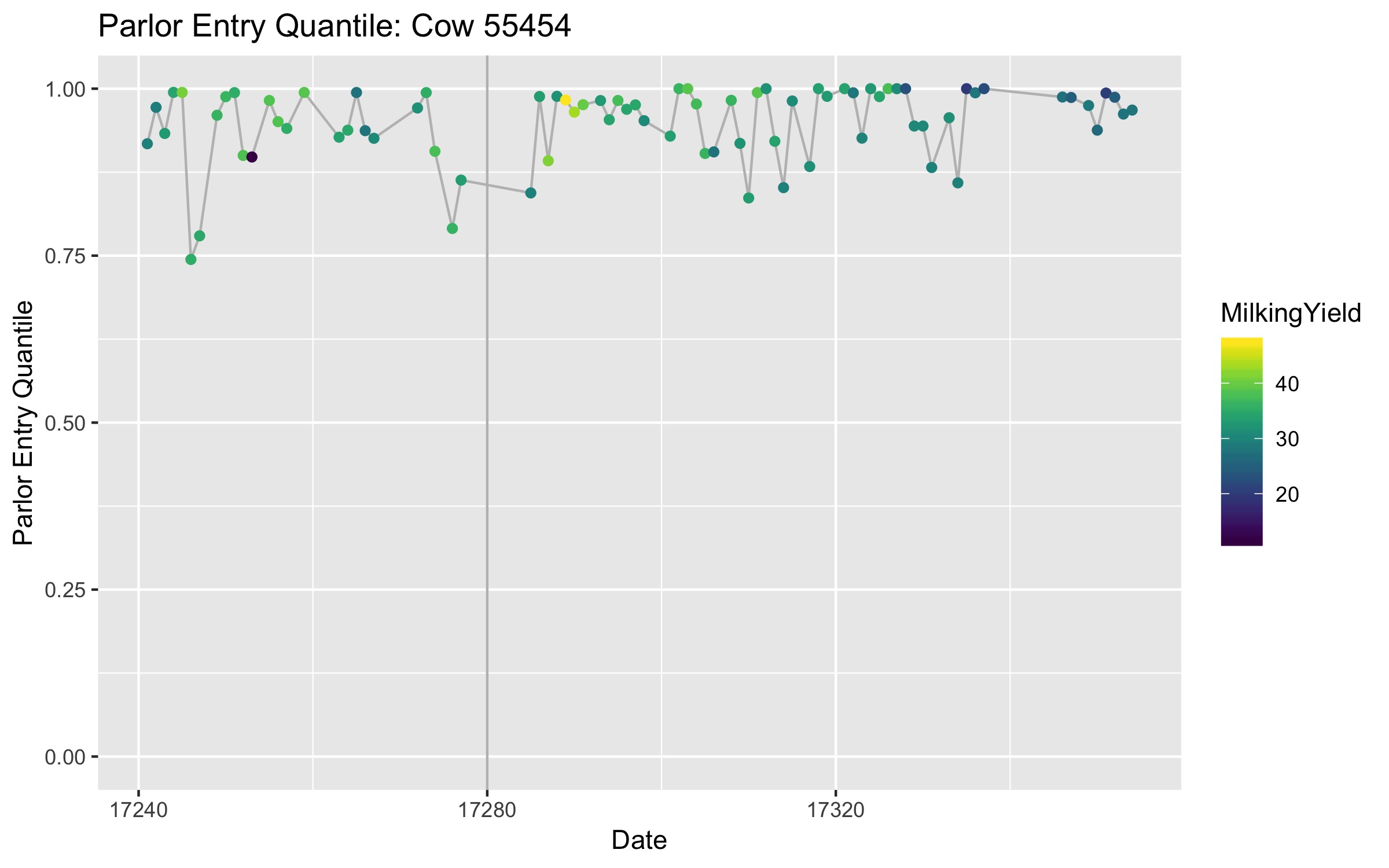

Supplement: Supplementary file 2 [file Data_Sheet_2.ZIP › Milking Yield/Cow_55454.jpg]

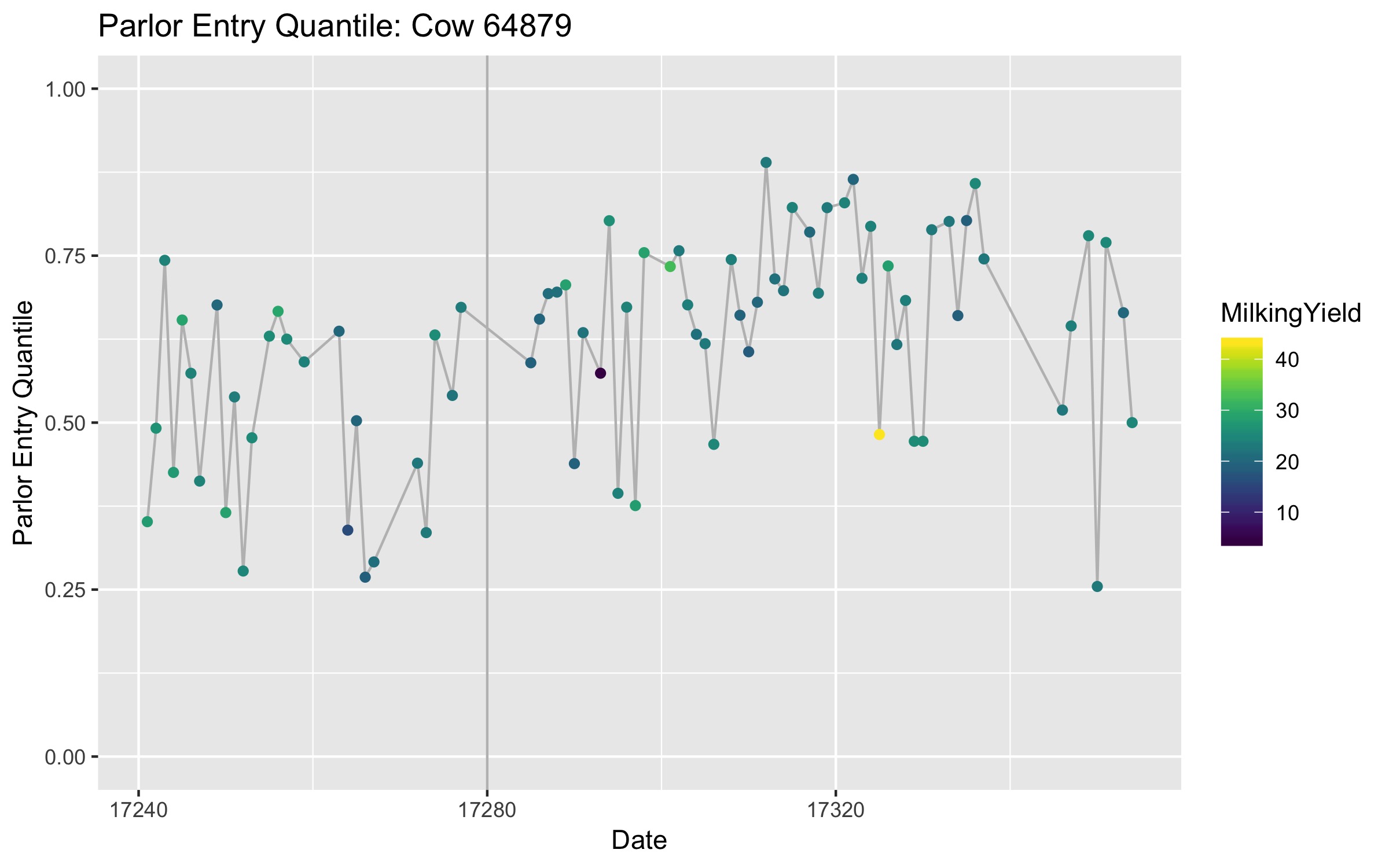

Supplement: Supplementary file 2 [file Data_Sheet_2.ZIP › Milking Yield/Cow_64879.jpg]

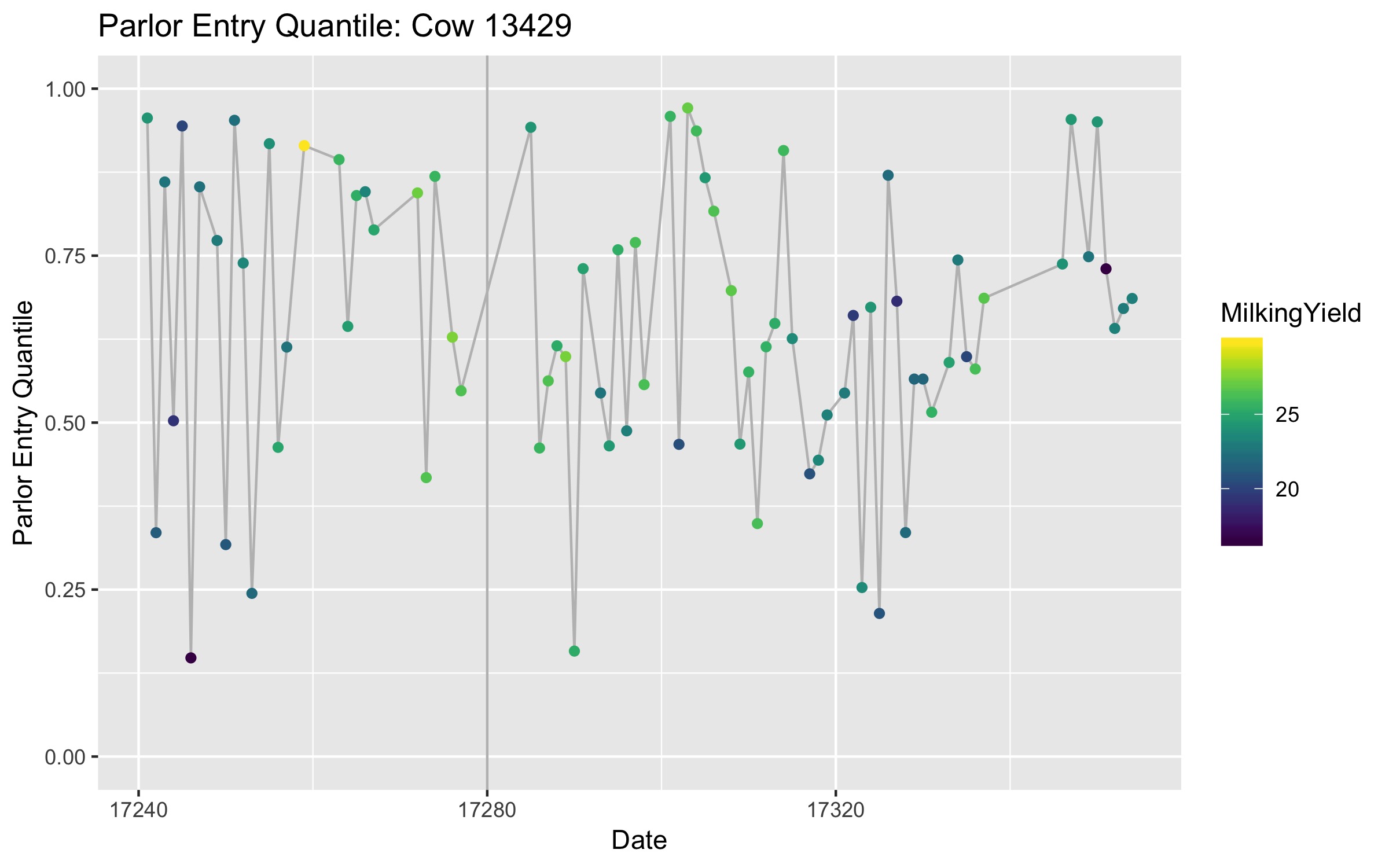

Supplement: Supplementary file 2 [file Data_Sheet_2.ZIP › Milking Yield/Cow_13429.jpg]

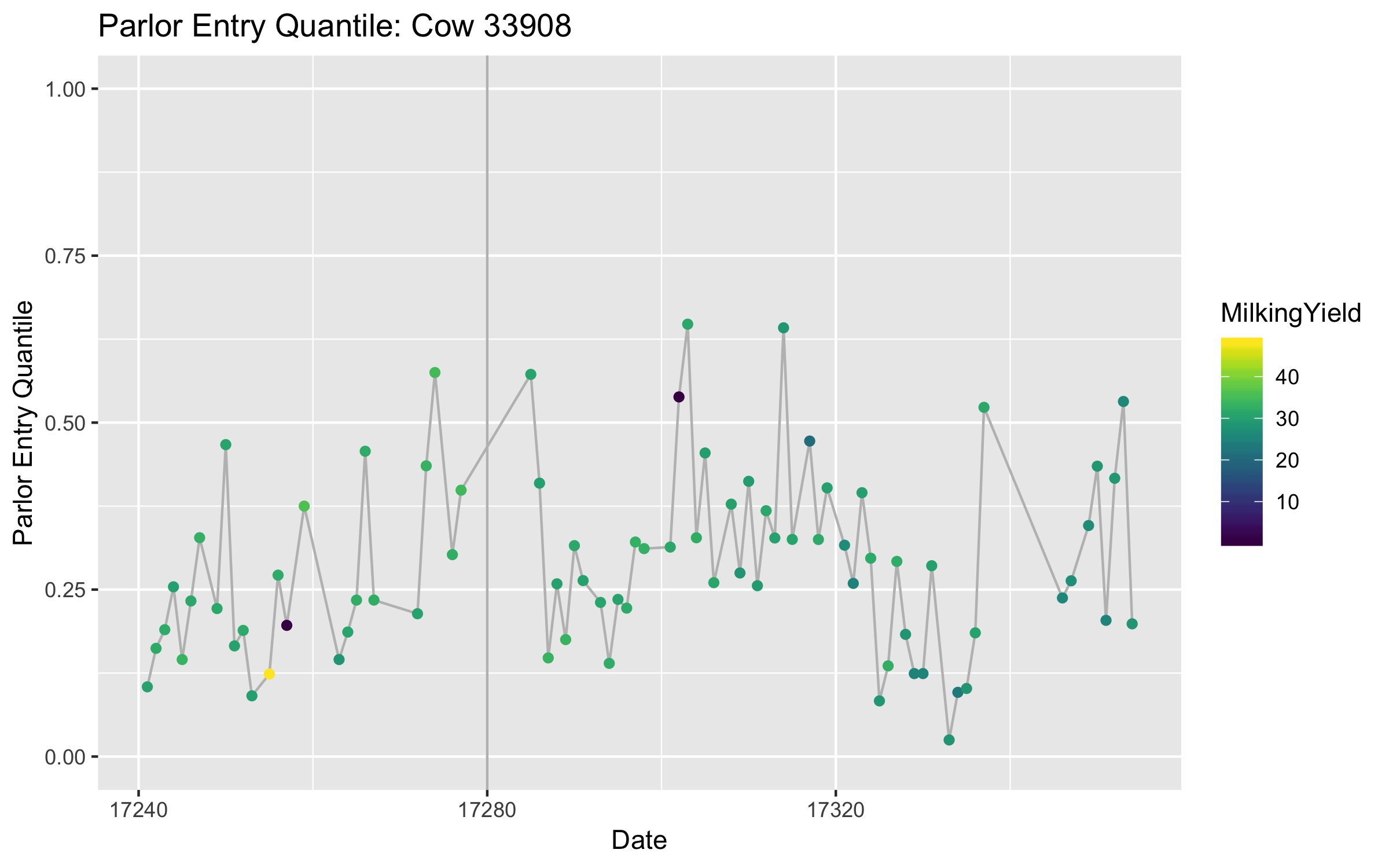

Supplement: Supplementary file 2 [file Data_Sheet_2.ZIP › Milking Yield/Cow_33908.jpg]

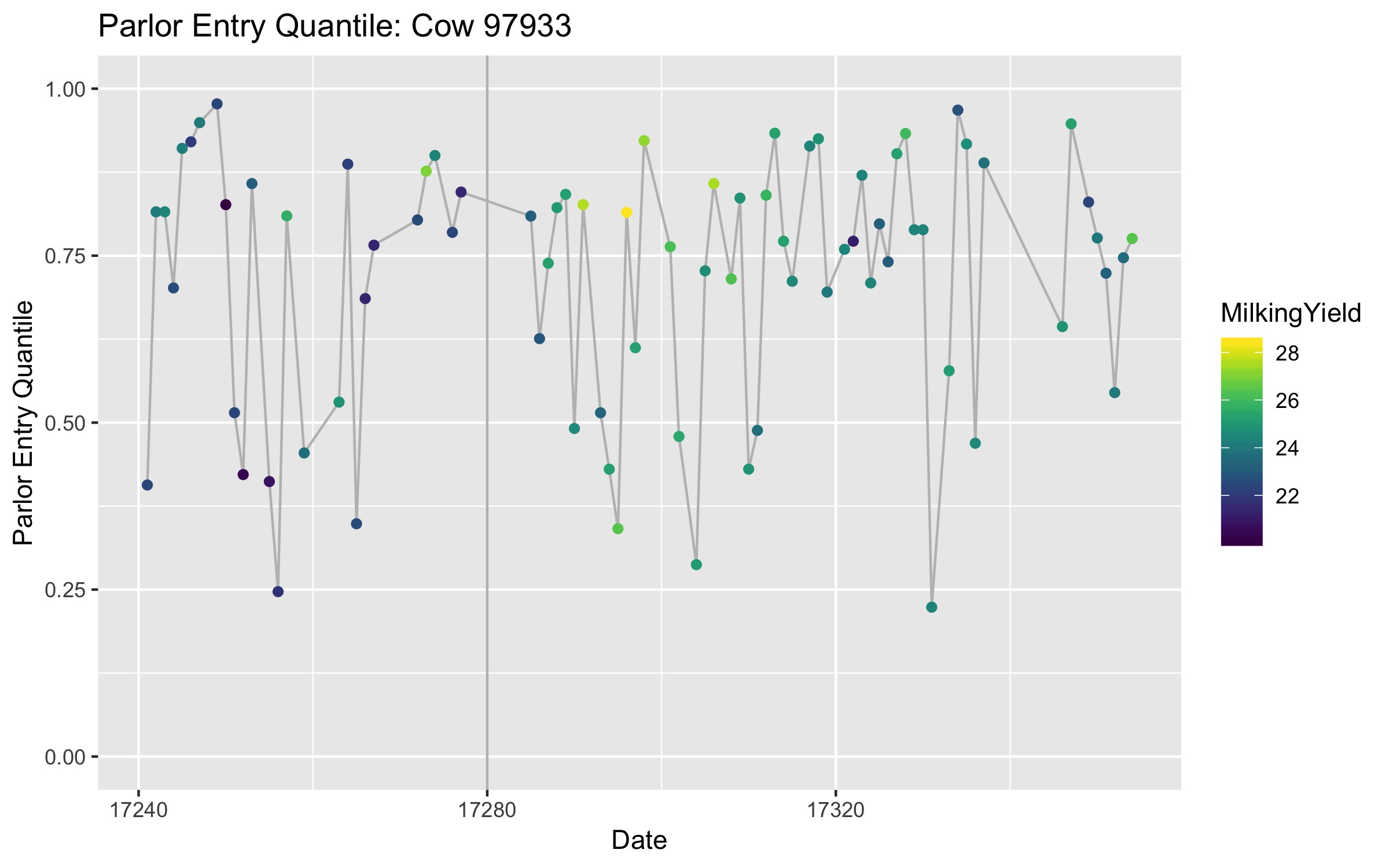

Supplement: Supplementary file 2 [file Data_Sheet_2.ZIP › Milking Yield/Cow_97933.jpg]

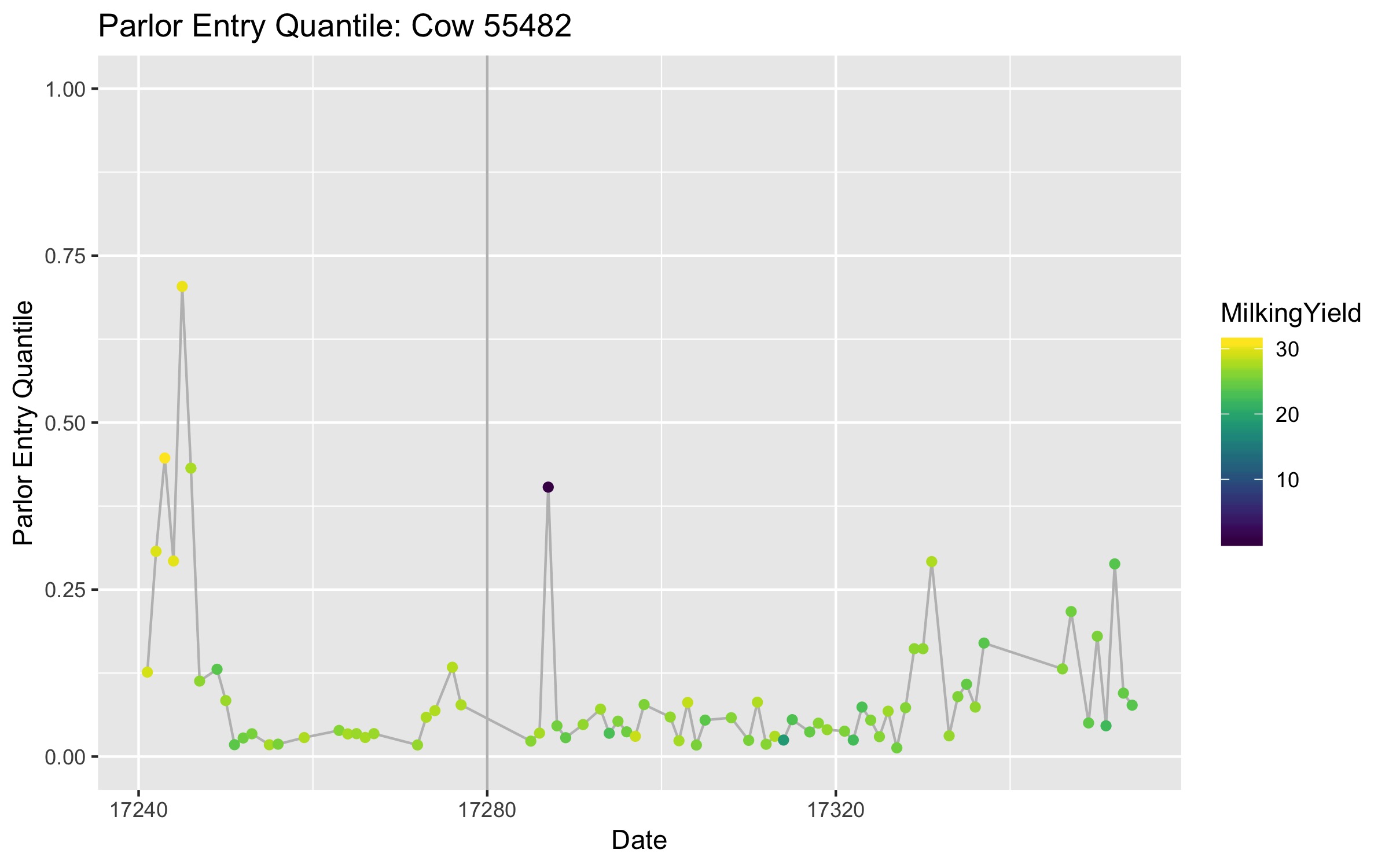

Supplement: Supplementary file 2 [file Data_Sheet_2.ZIP › Milking Yield/Cow_55482.jpg]

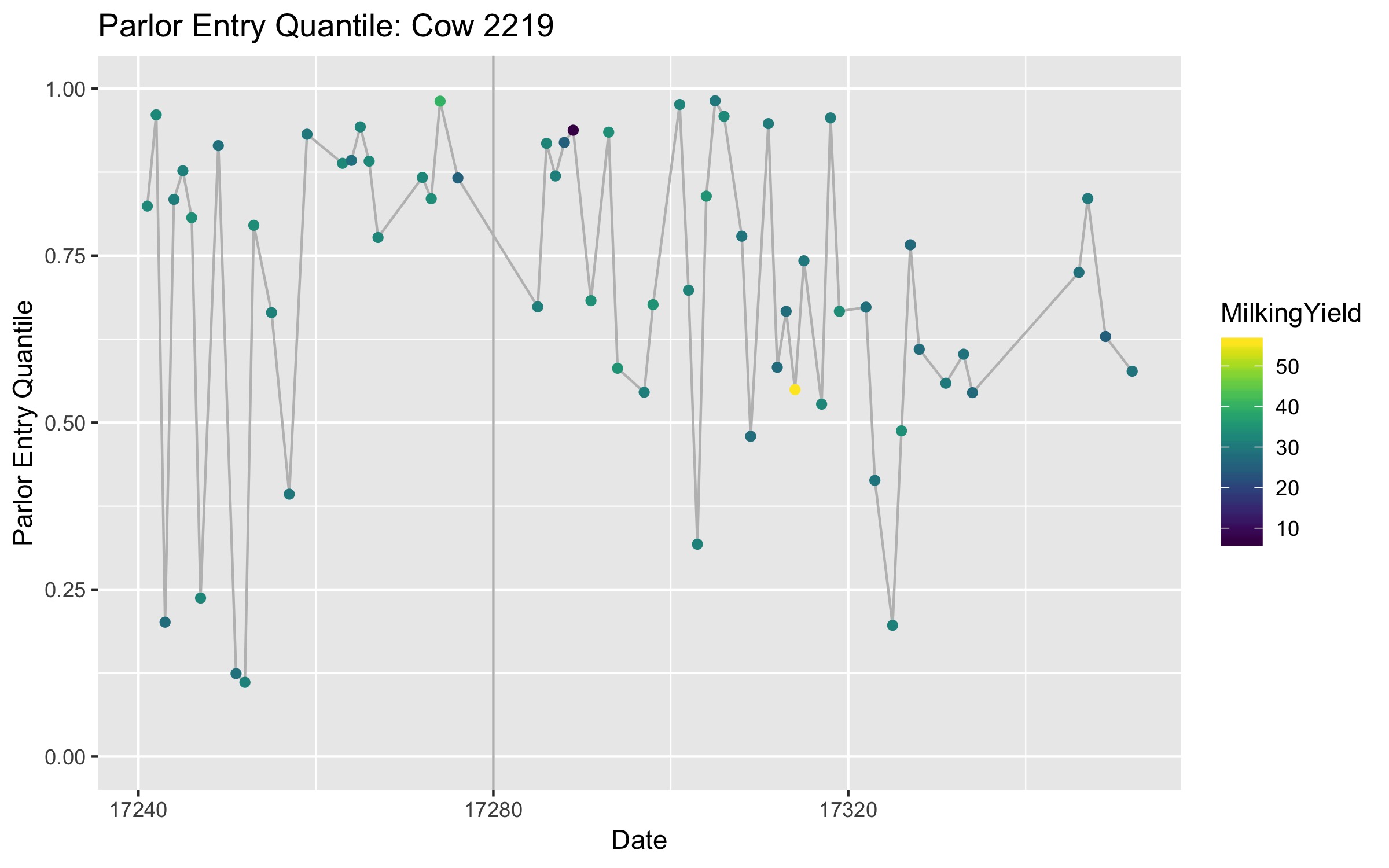

Supplement: Supplementary file 2 [file Data_Sheet_2.ZIP › Milking Yield/Cow_2219.jpg]

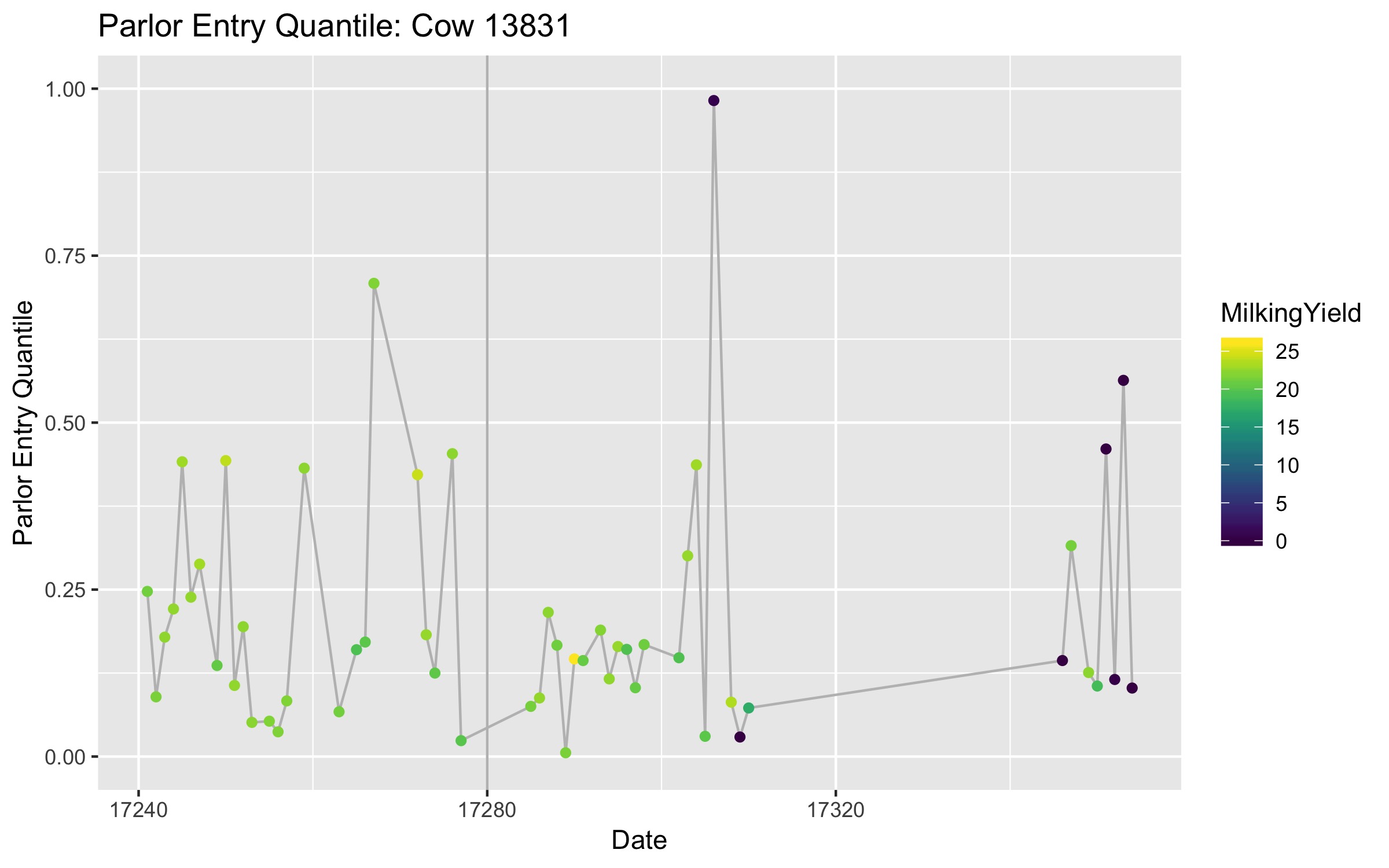

Supplement: Supplementary file 2 [file Data_Sheet_2.ZIP › Milking Yield/Cow_13831.jpg]

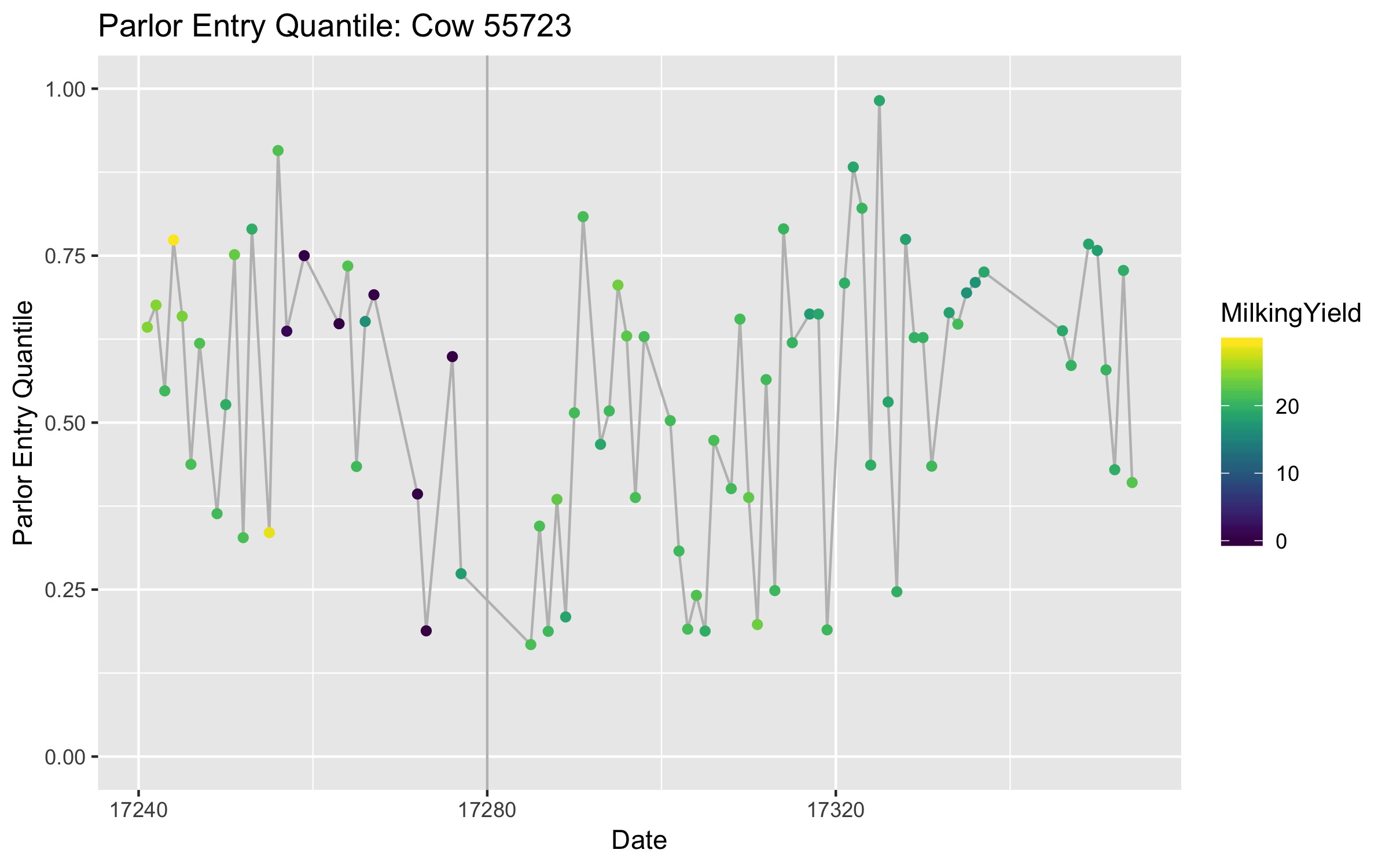

Supplement: Supplementary file 2 [file Data_Sheet_2.ZIP › Milking Yield/Cow_55723.jpg]

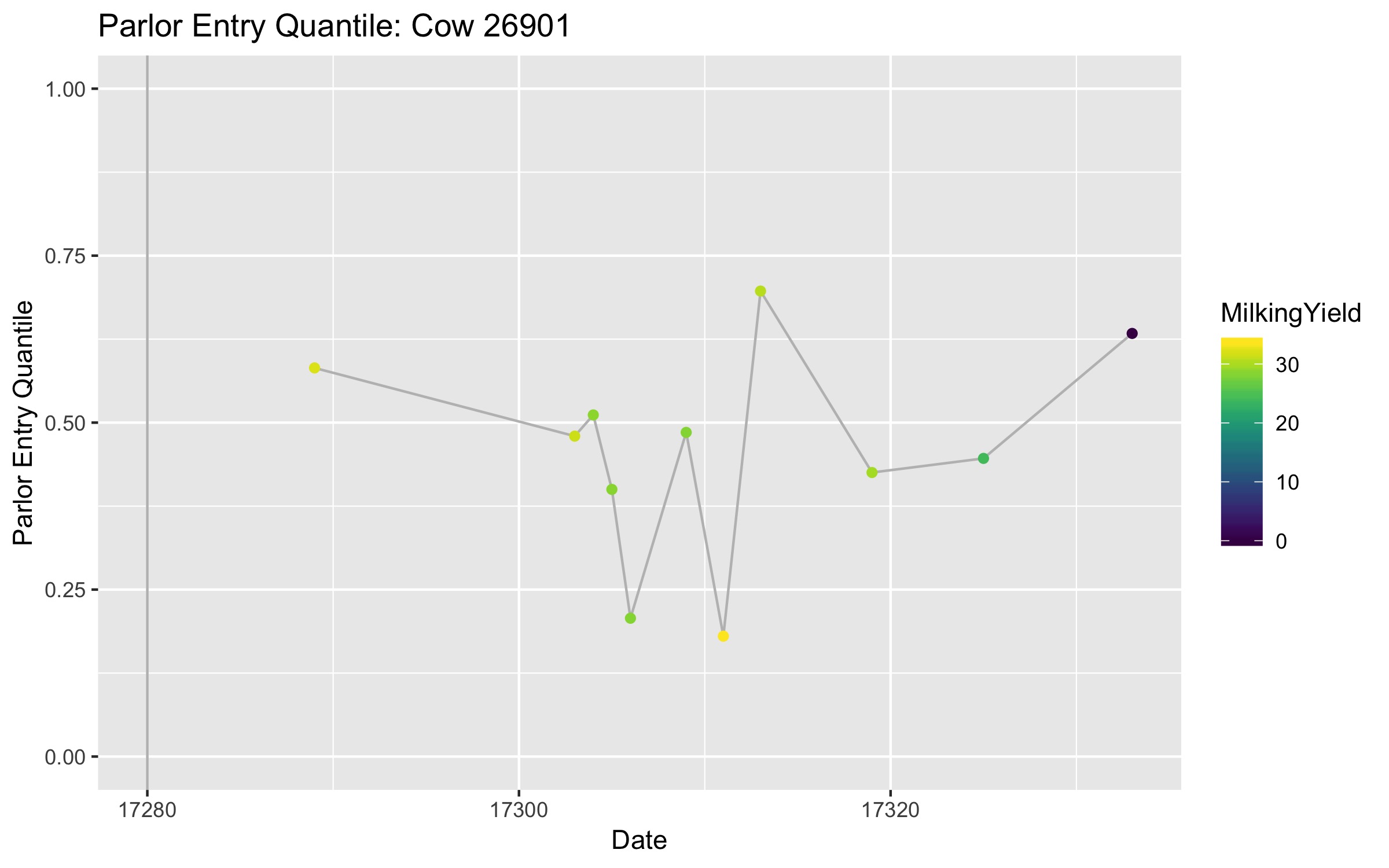

Supplement: Supplementary file 2 [file Data_Sheet_2.ZIP › Milking Yield/Cow_26901.jpg]

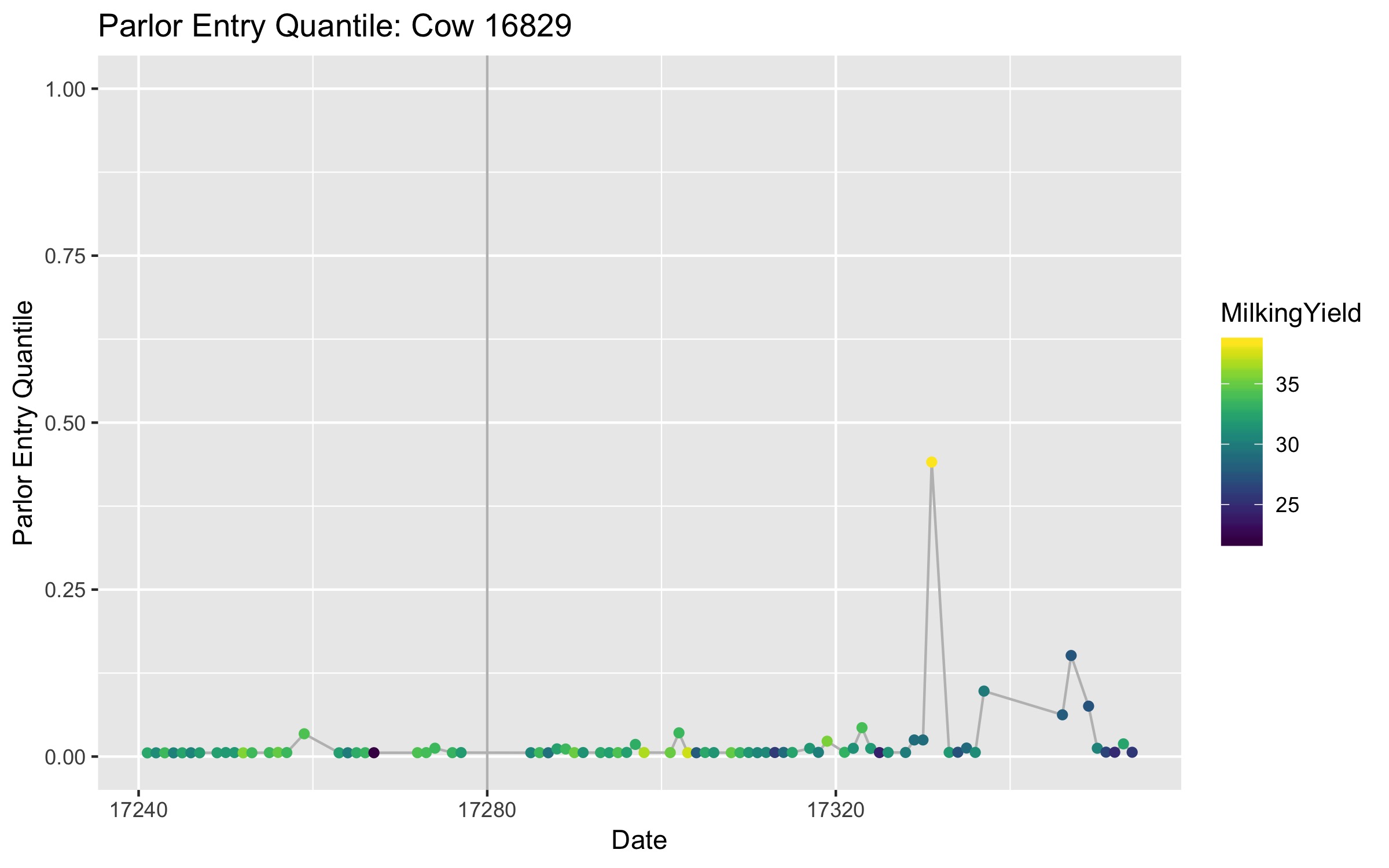

Supplement: Supplementary file 2 [file Data_Sheet_2.ZIP › Milking Yield/Cow_16829.jpg]

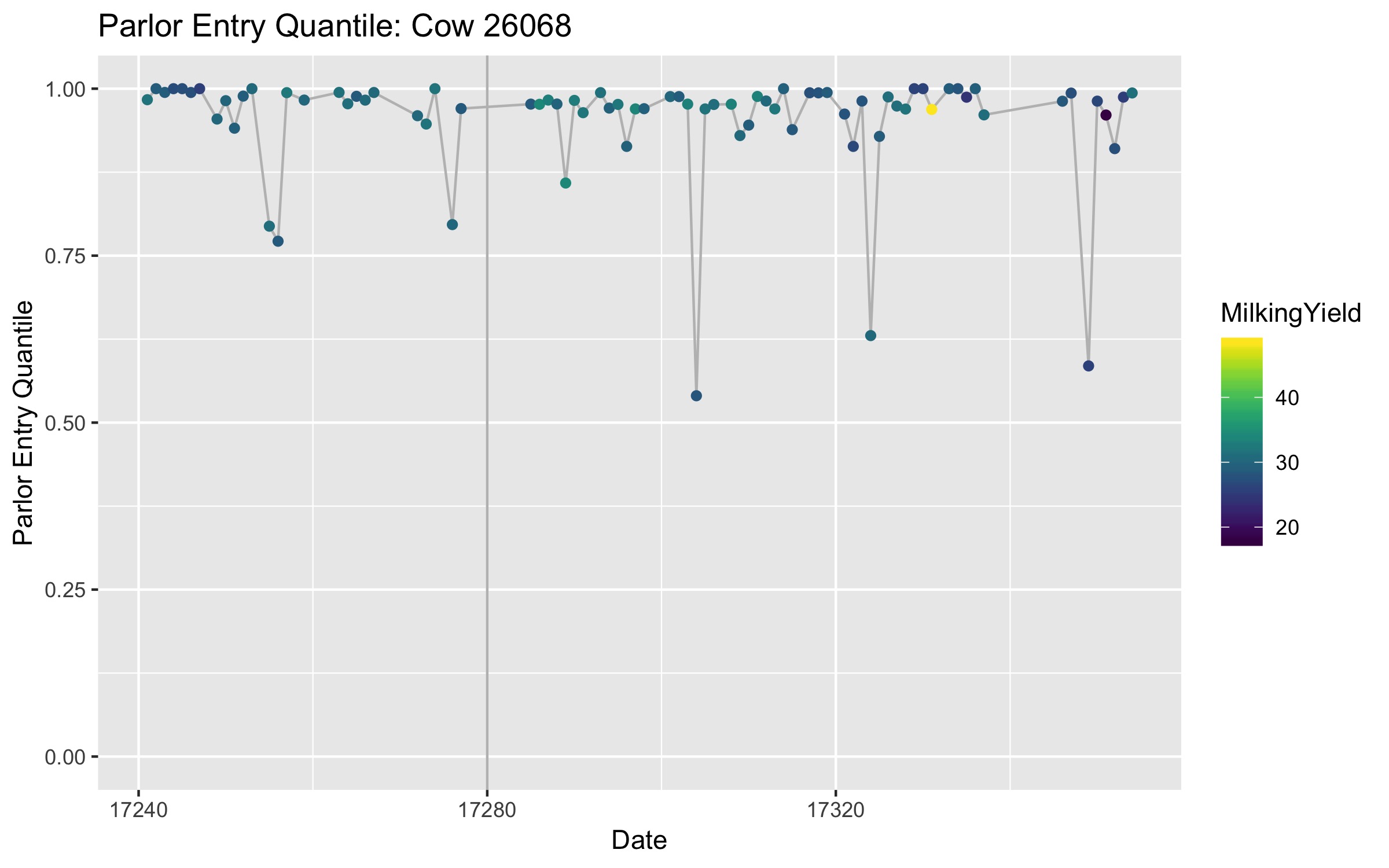

Supplement: Supplementary file 2 [file Data_Sheet_2.ZIP › Milking Yield/Cow_26068.jpg]

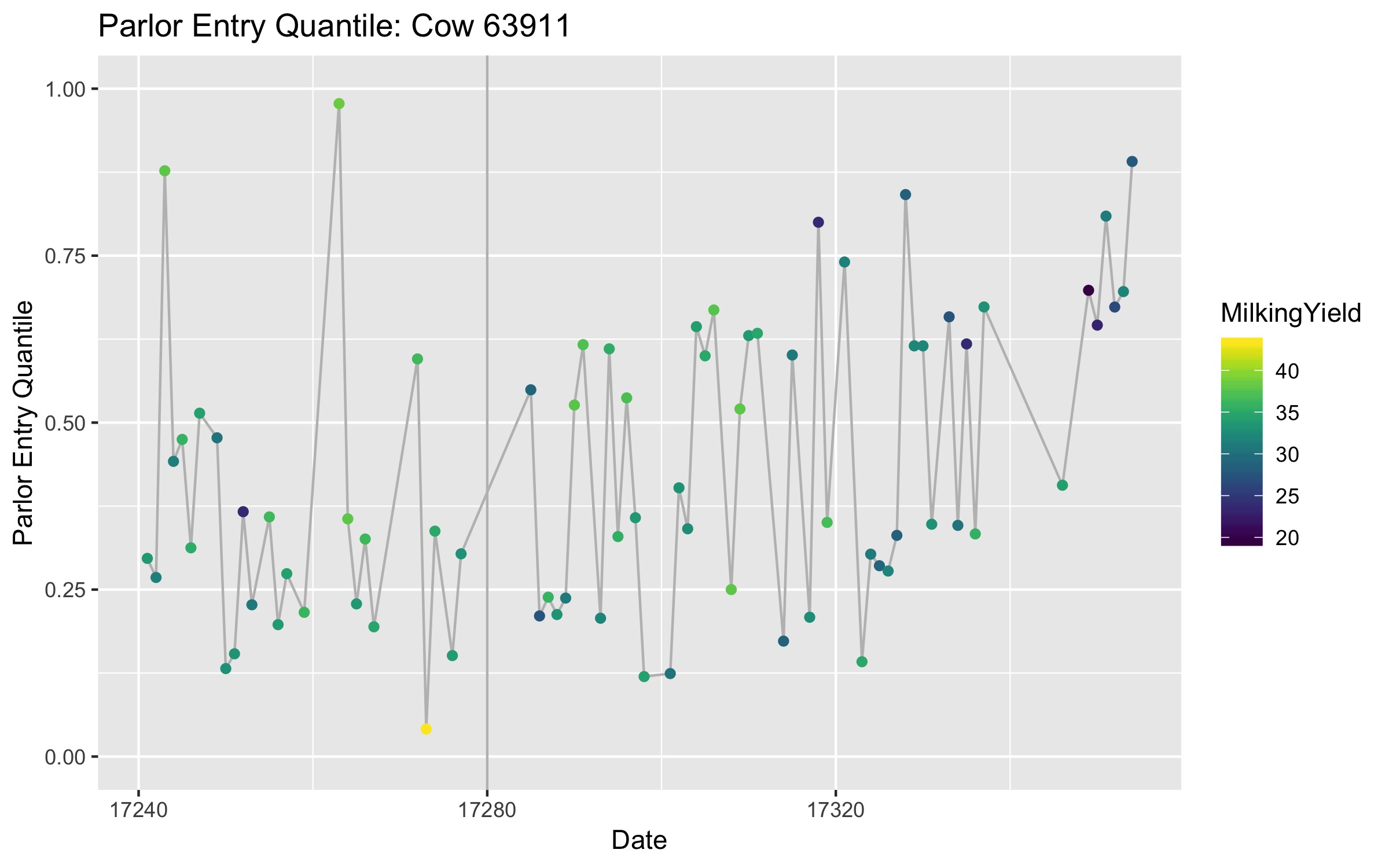

Supplement: Supplementary file 2 [file Data_Sheet_2.ZIP › Milking Yield/Cow_63911.jpg]

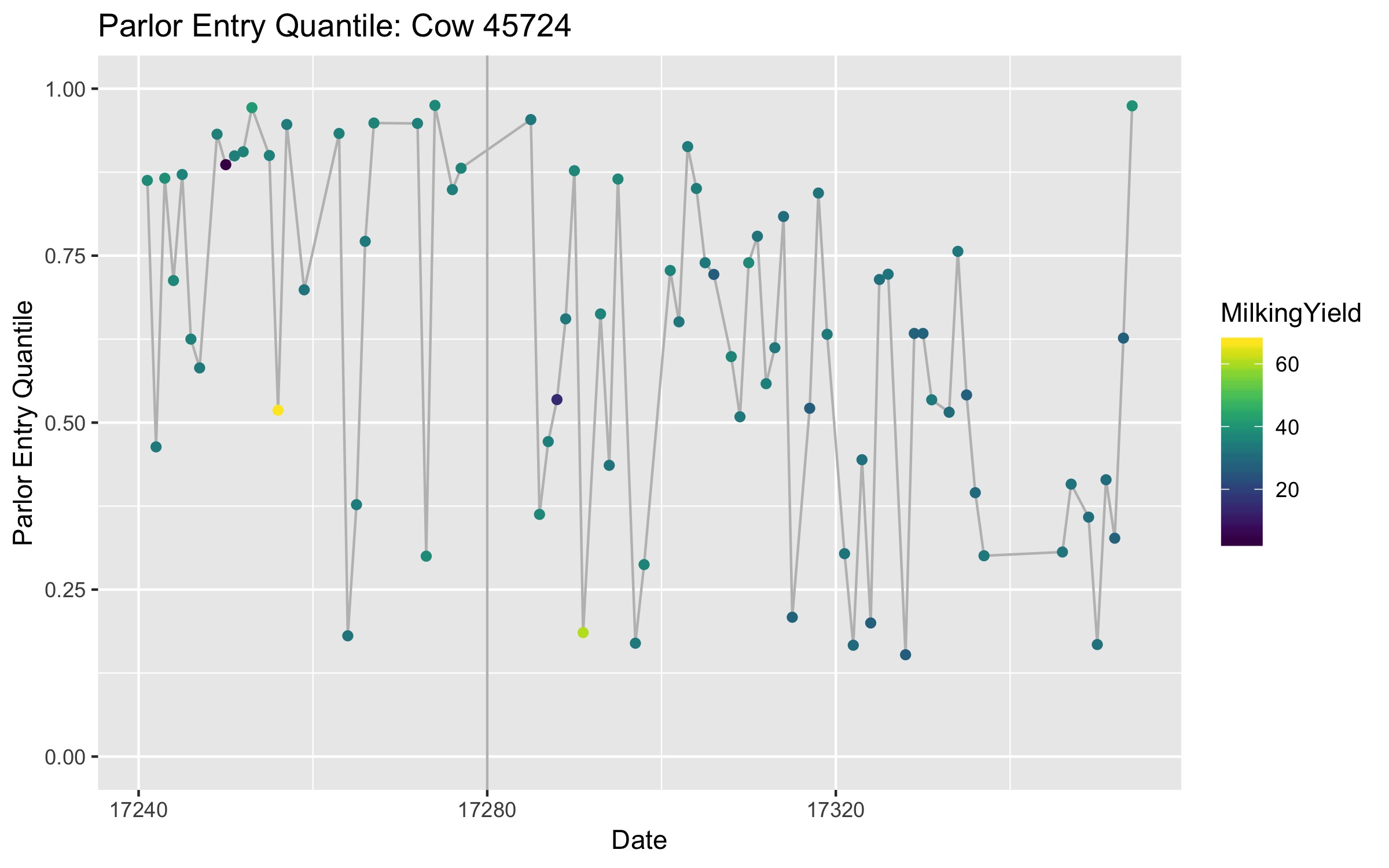

Supplement: Supplementary file 2 [file Data_Sheet_2.ZIP › Milking Yield/Cow_45724.jpg]

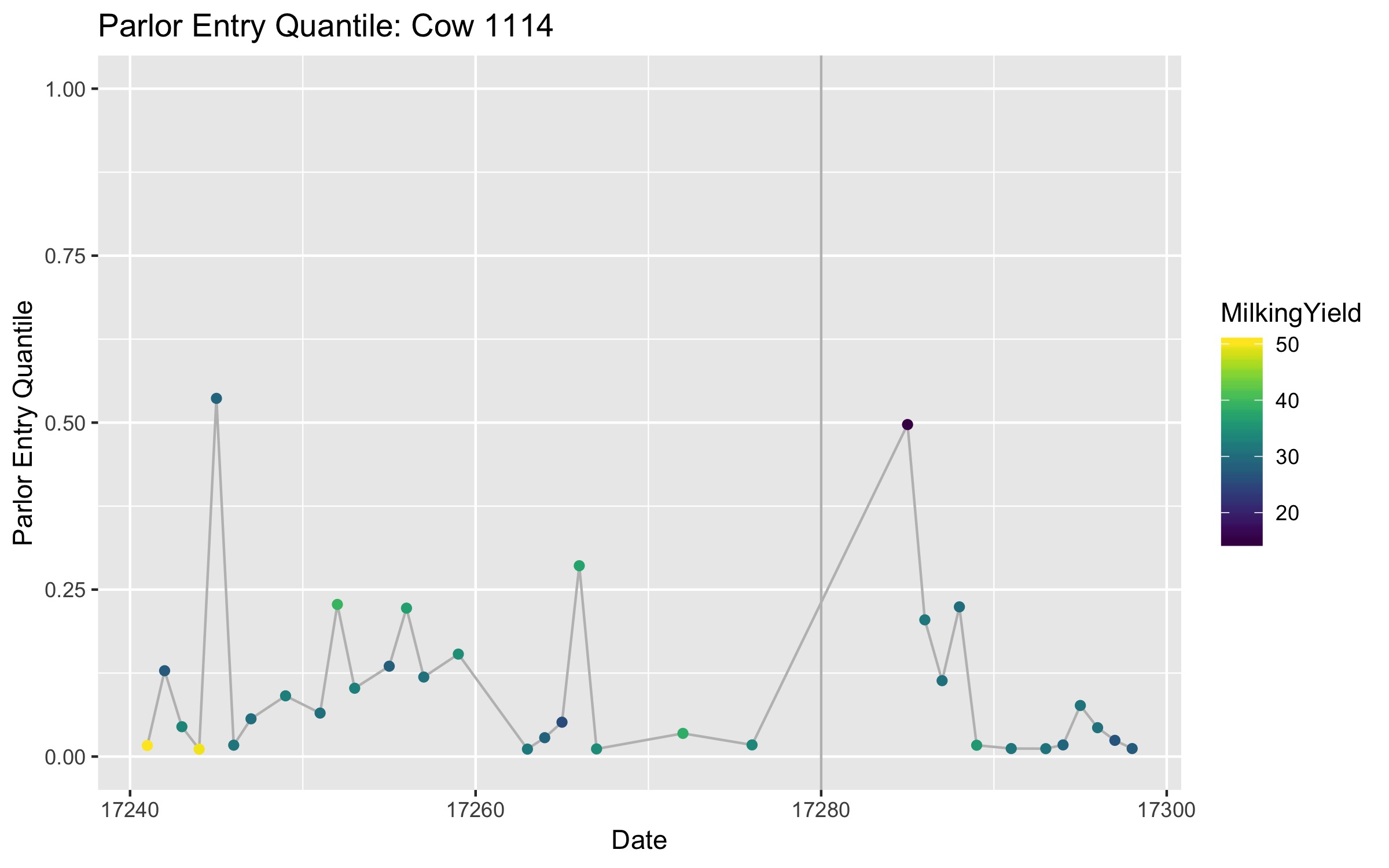

Supplement: Supplementary file 2 [file Data_Sheet_2.ZIP › Milking Yield/Cow_1114.jpg]

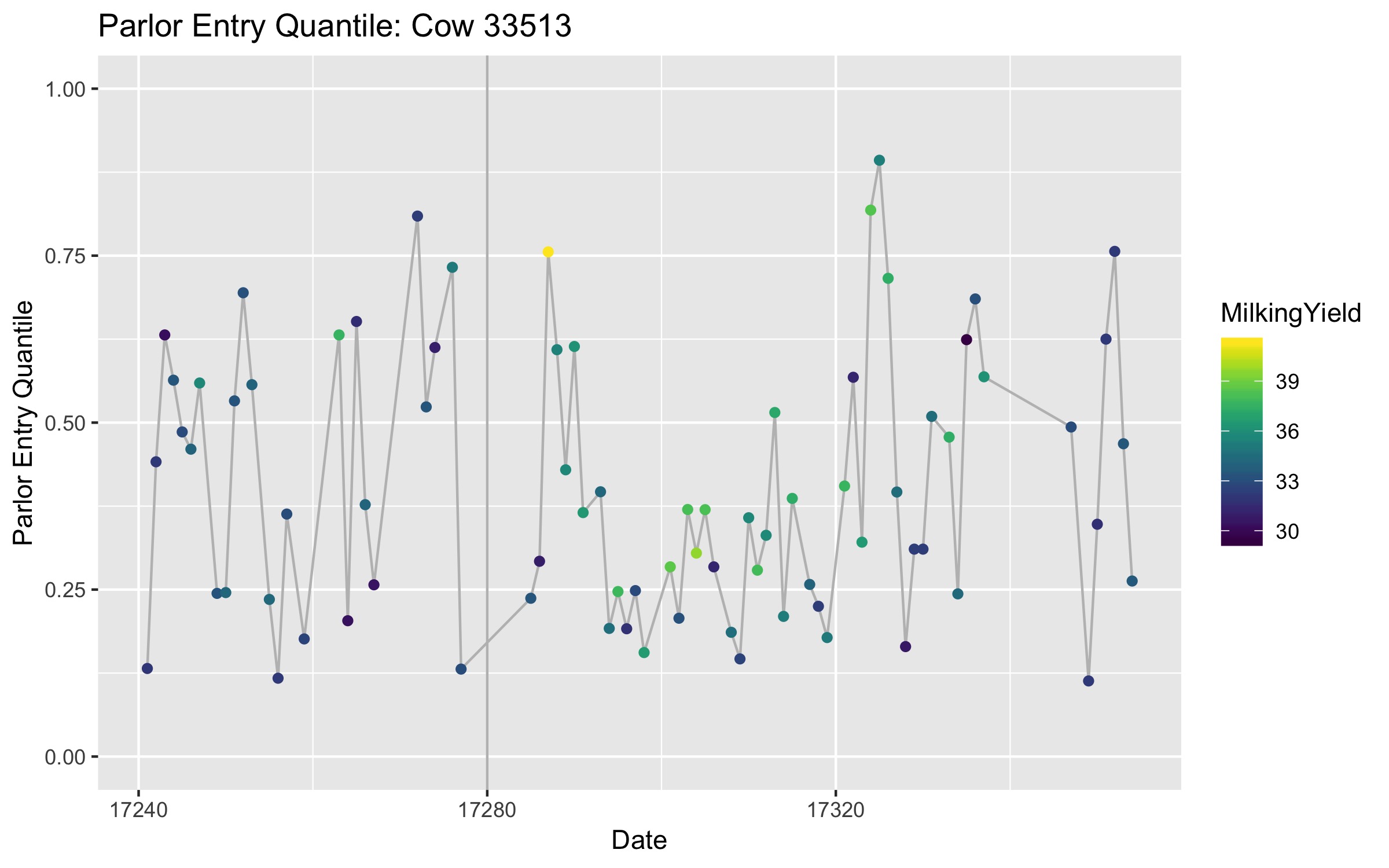

Supplement: Supplementary file 2 [file Data_Sheet_2.ZIP › Milking Yield/Cow_33513.jpg]

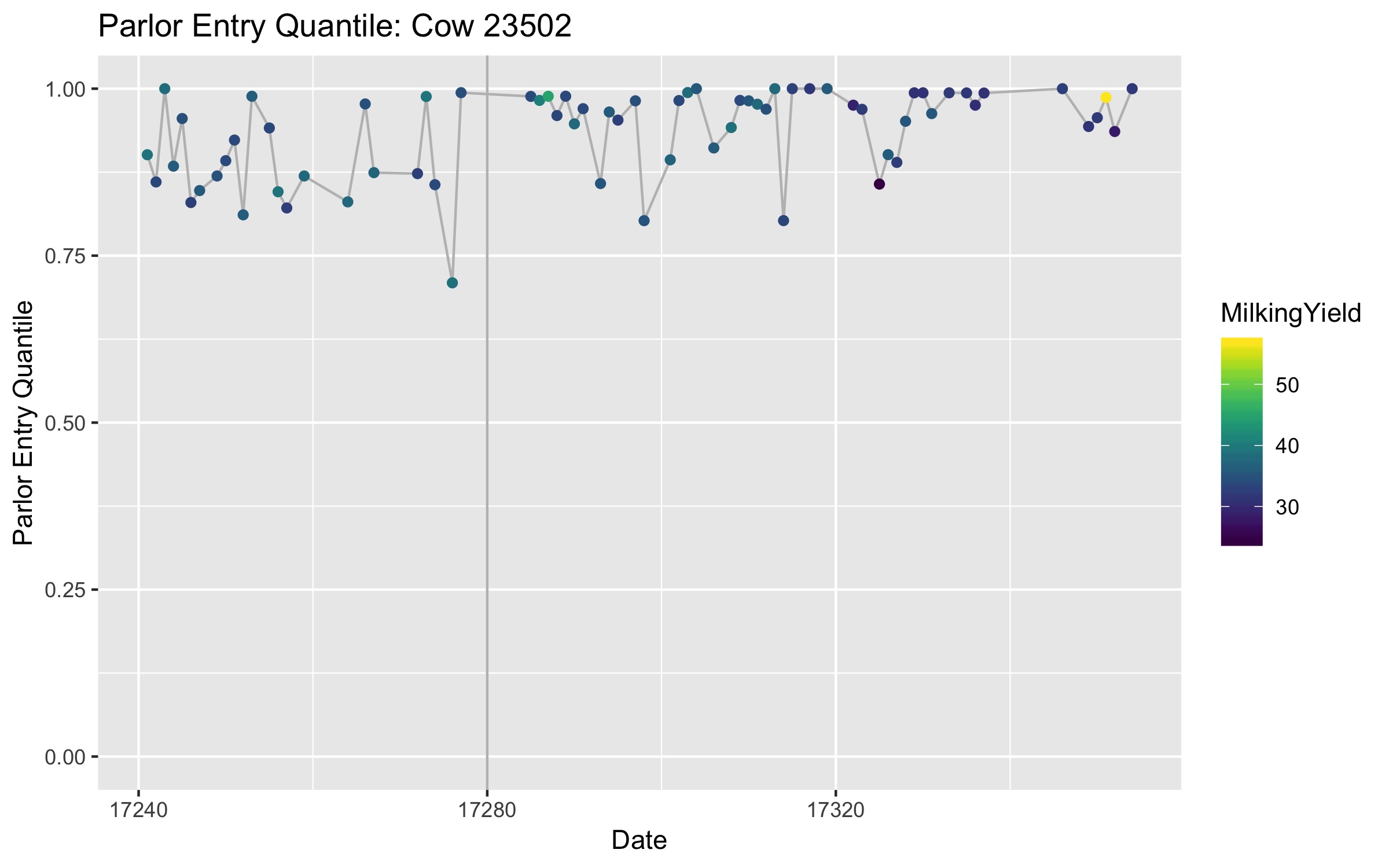

Supplement: Supplementary file 2 [file Data_Sheet_2.ZIP › Milking Yield/Cow_23502.jpg]

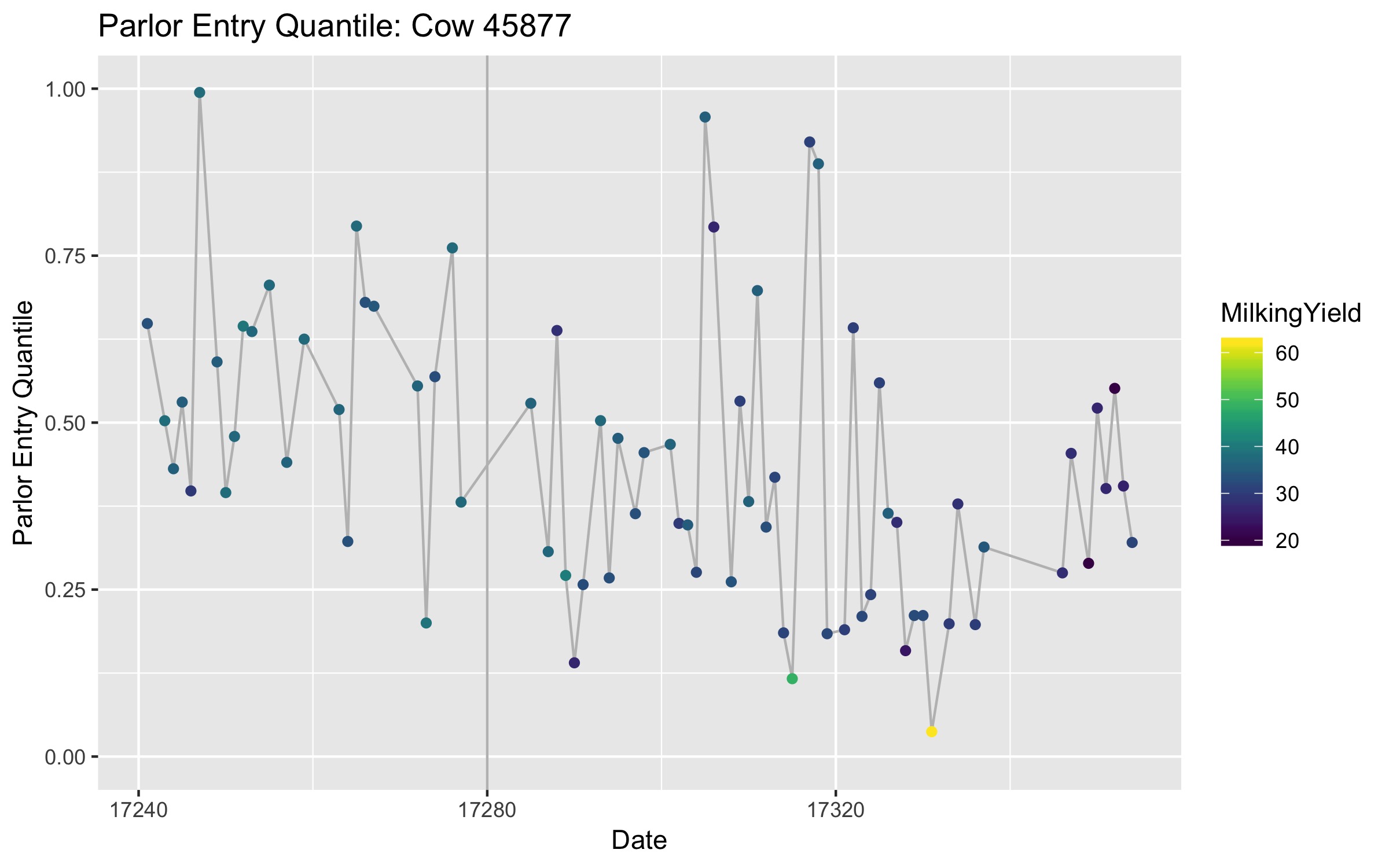

Supplement: Supplementary file 2 [file Data_Sheet_2.ZIP › Milking Yield/Cow_45877.jpg]

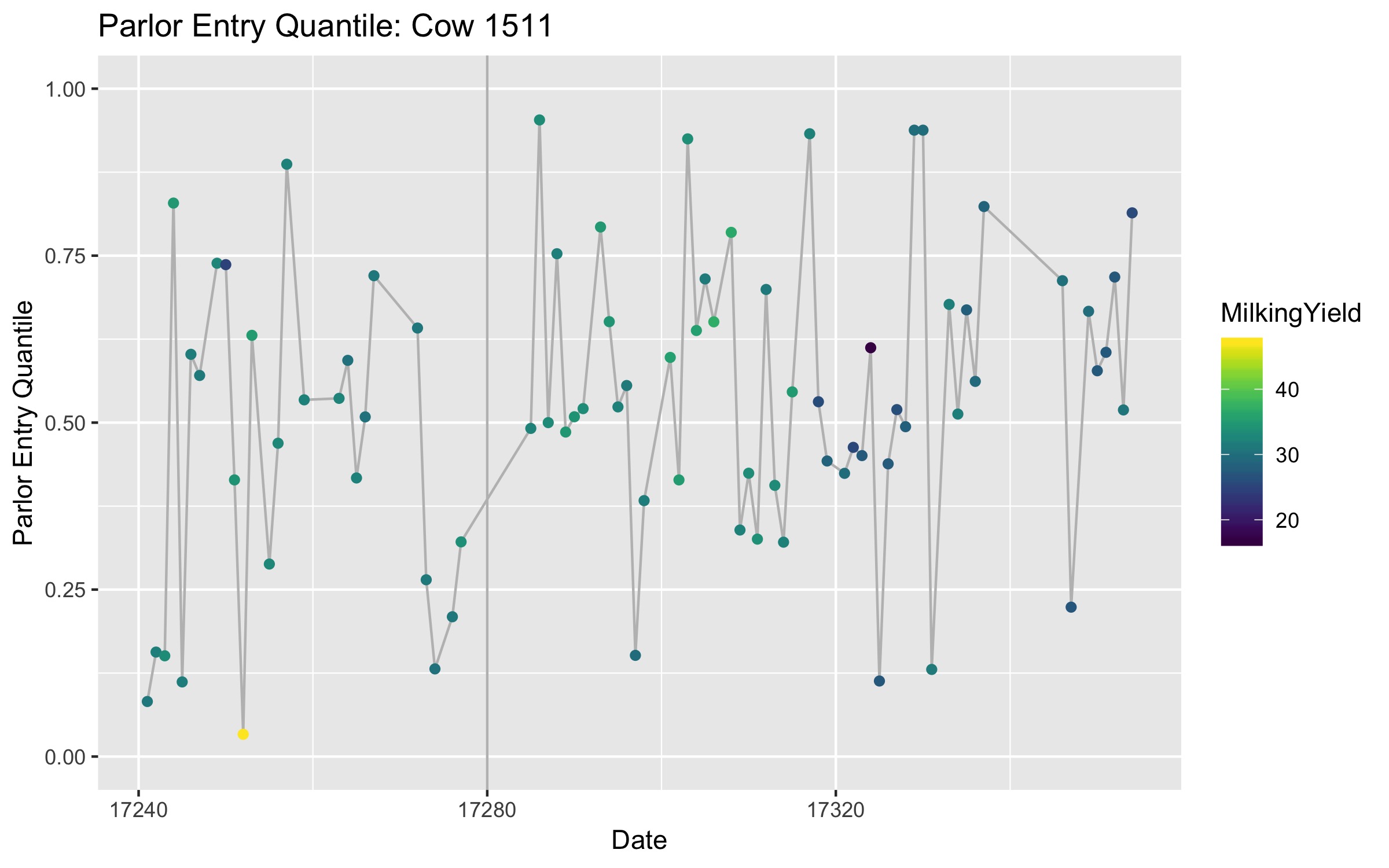

Supplement: Supplementary file 2 [file Data_Sheet_2.ZIP › Milking Yield/Cow_1511.jpg]

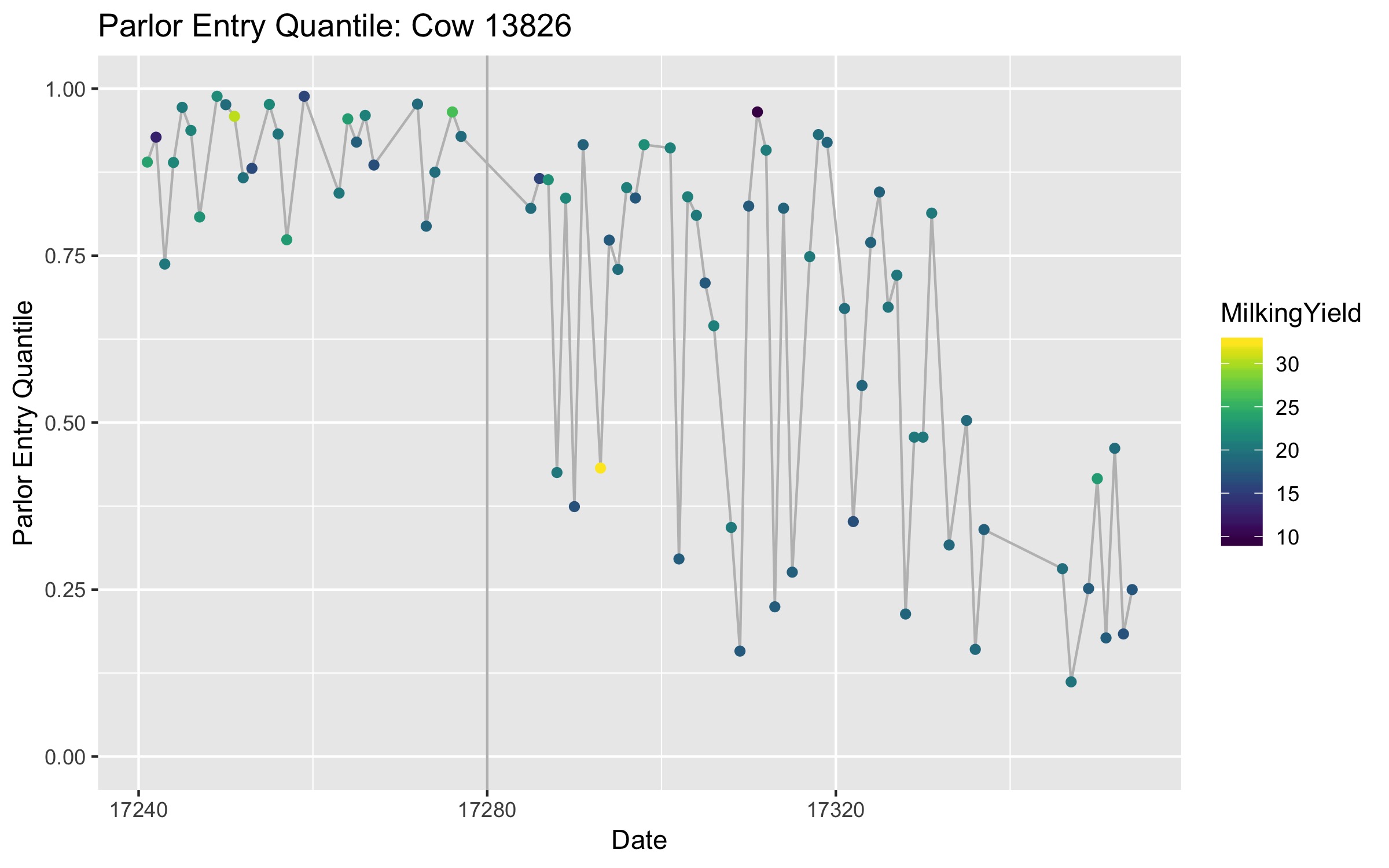

Supplement: Supplementary file 2 [file Data_Sheet_2.ZIP › Milking Yield/Cow_13826.jpg]

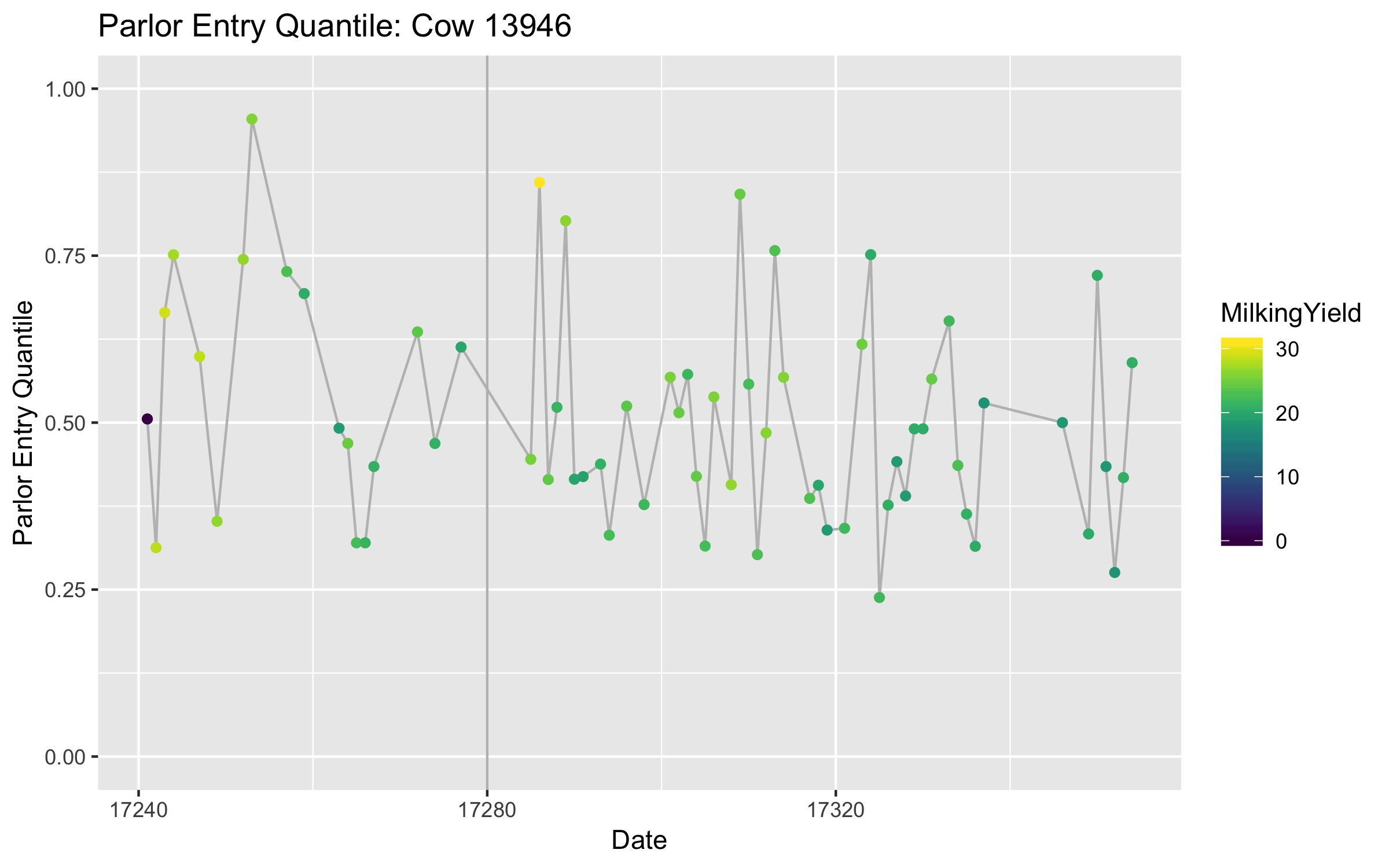

Supplement: Supplementary file 2 [file Data_Sheet_2.ZIP › Milking Yield/Cow_13946.jpg]

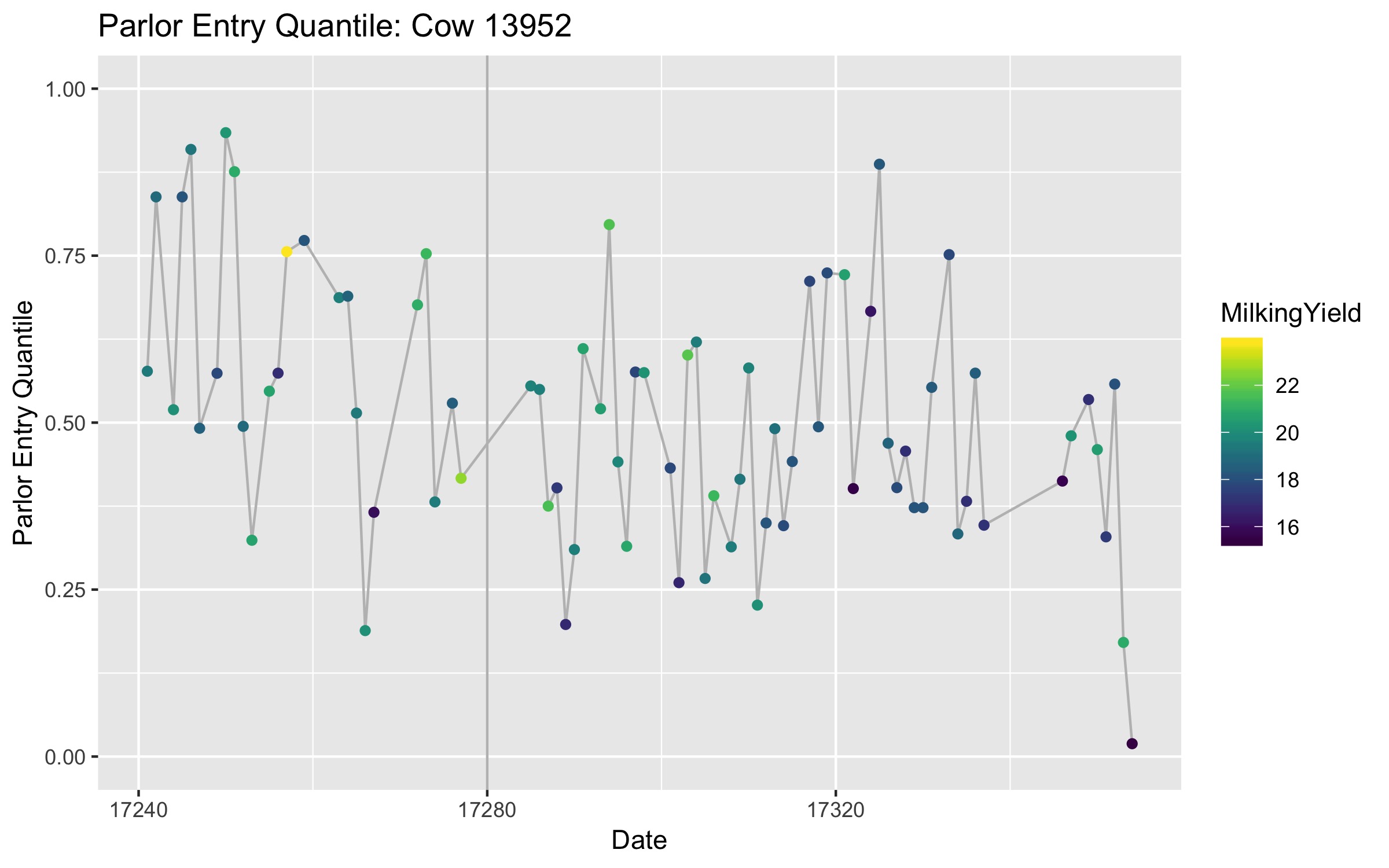

Supplement: Supplementary file 2 [file Data_Sheet_2.ZIP › Milking Yield/Cow_13952.jpg]

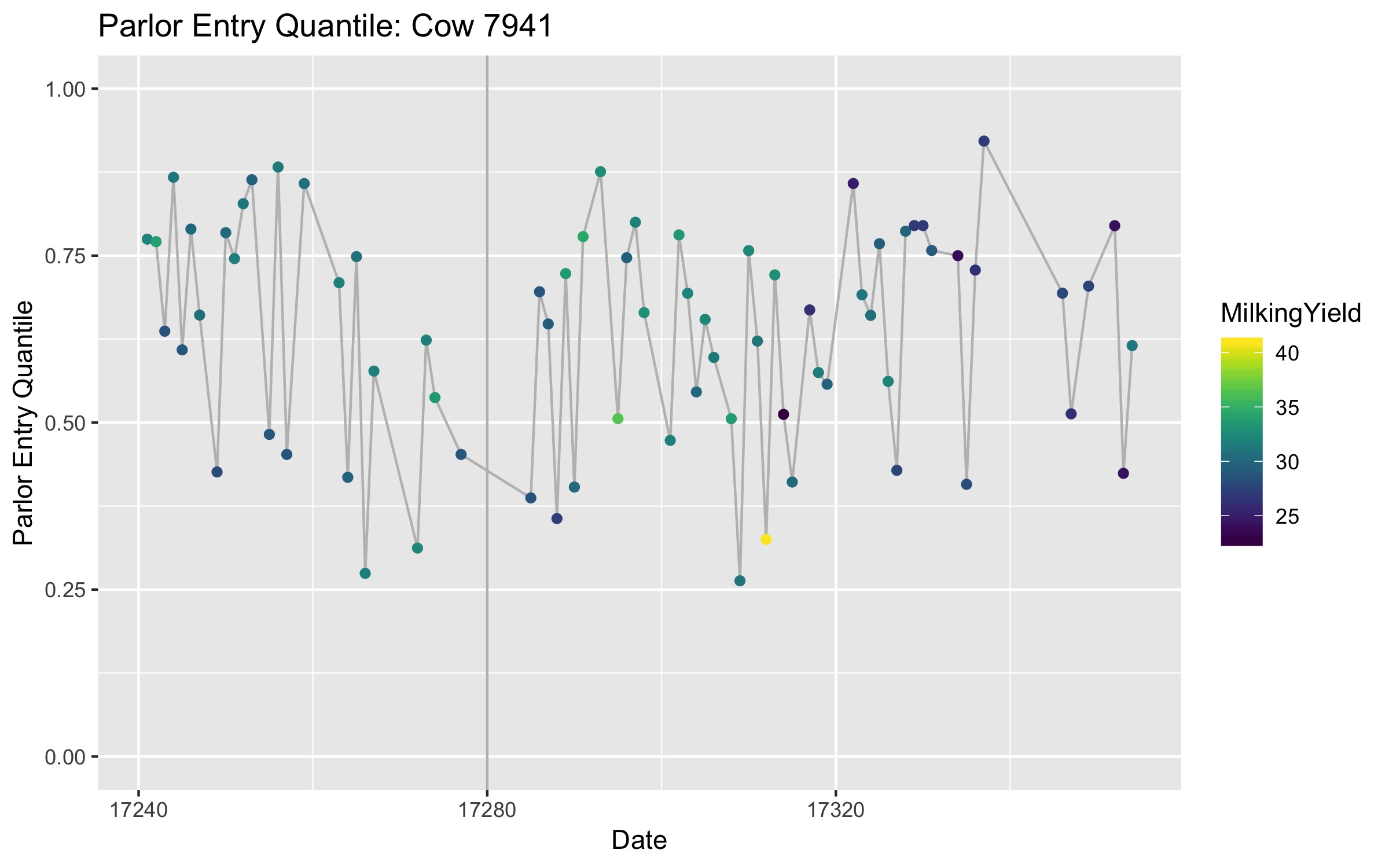

Supplement: Supplementary file 2 [file Data_Sheet_2.ZIP › Milking Yield/Cow_7941.jpg]

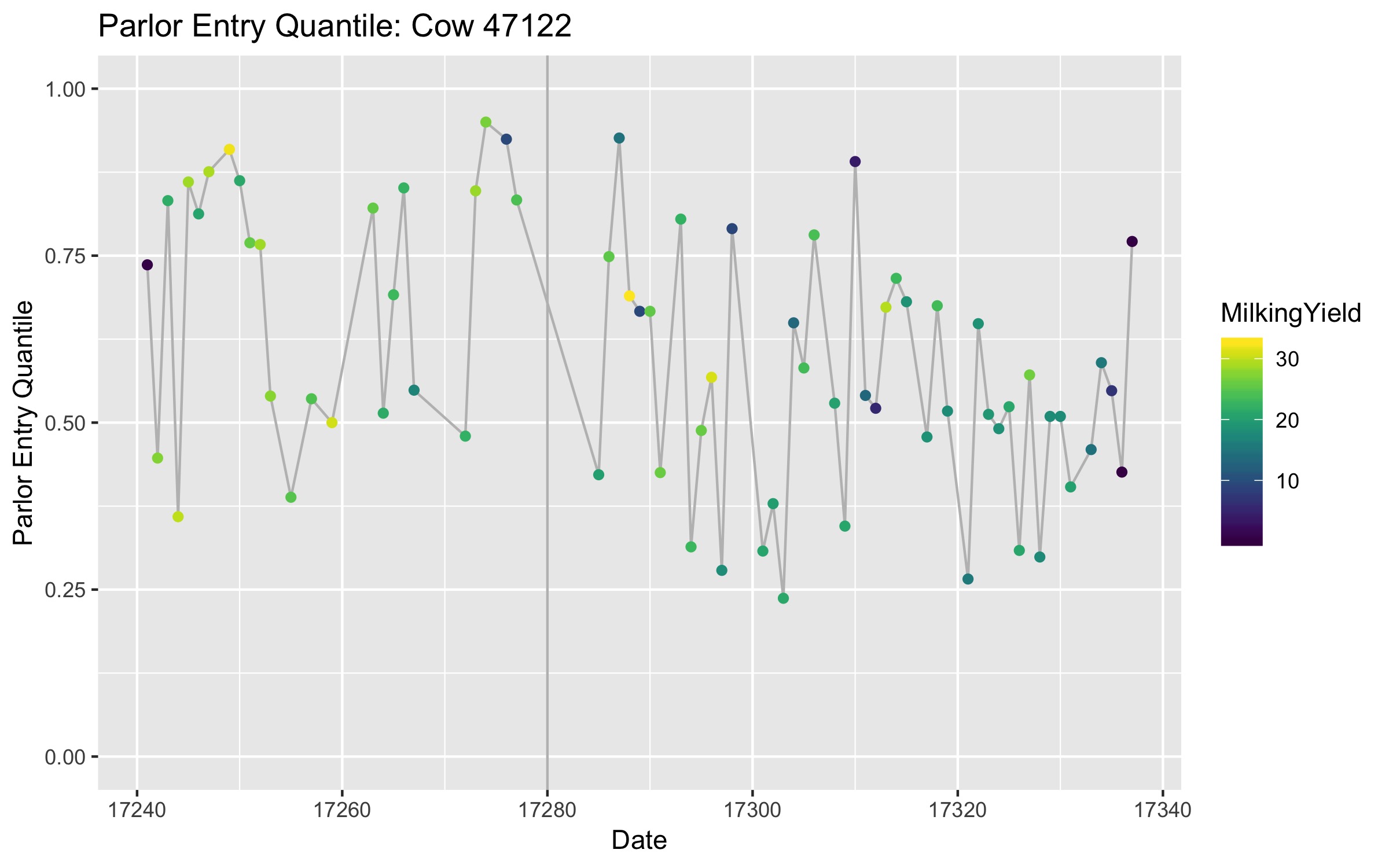

Supplement: Supplementary file 2 [file Data_Sheet_2.ZIP › Milking Yield/Cow_47122.jpg]

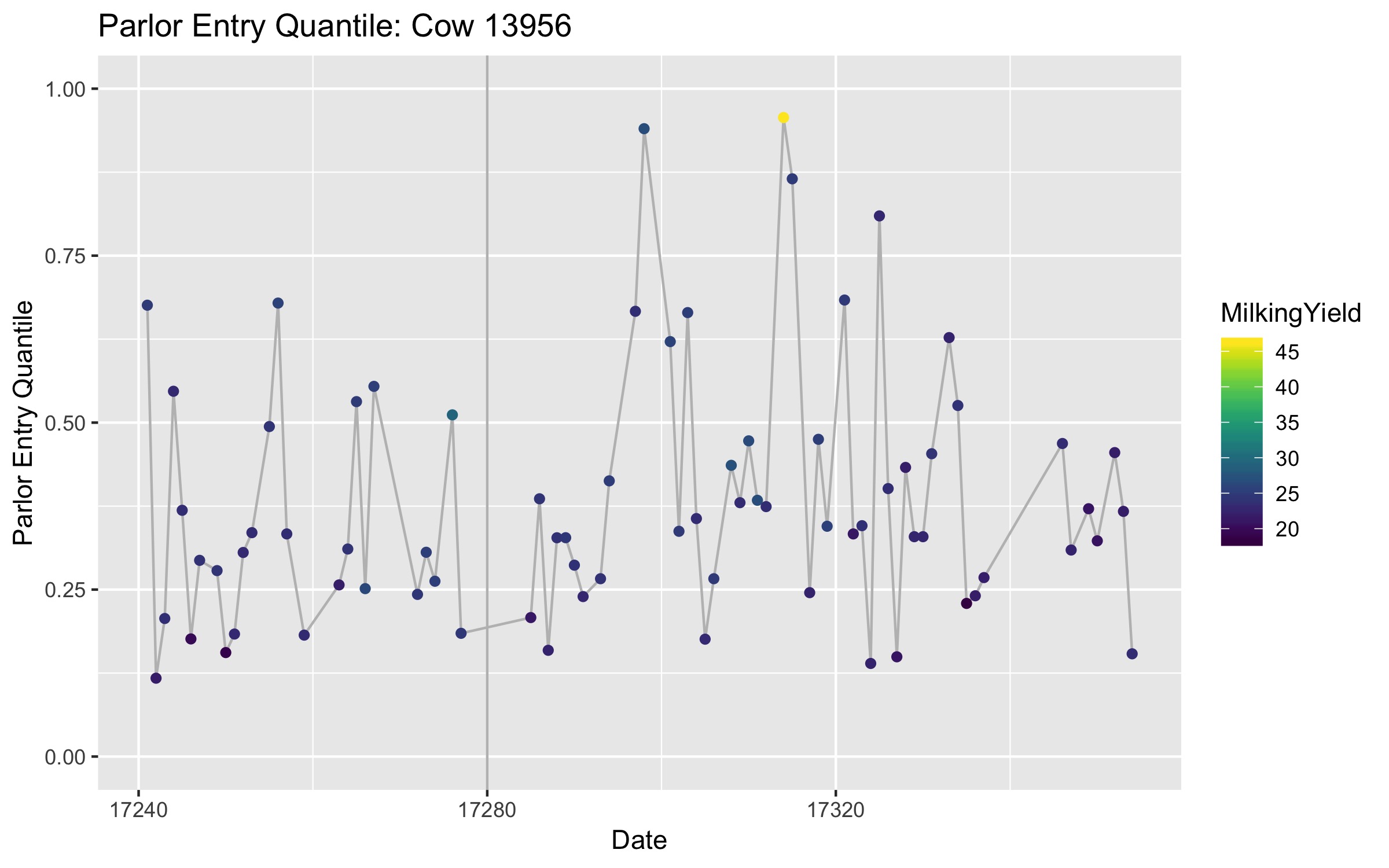

Supplement: Supplementary file 2 [file Data_Sheet_2.ZIP › Milking Yield/Cow_13956.jpg]

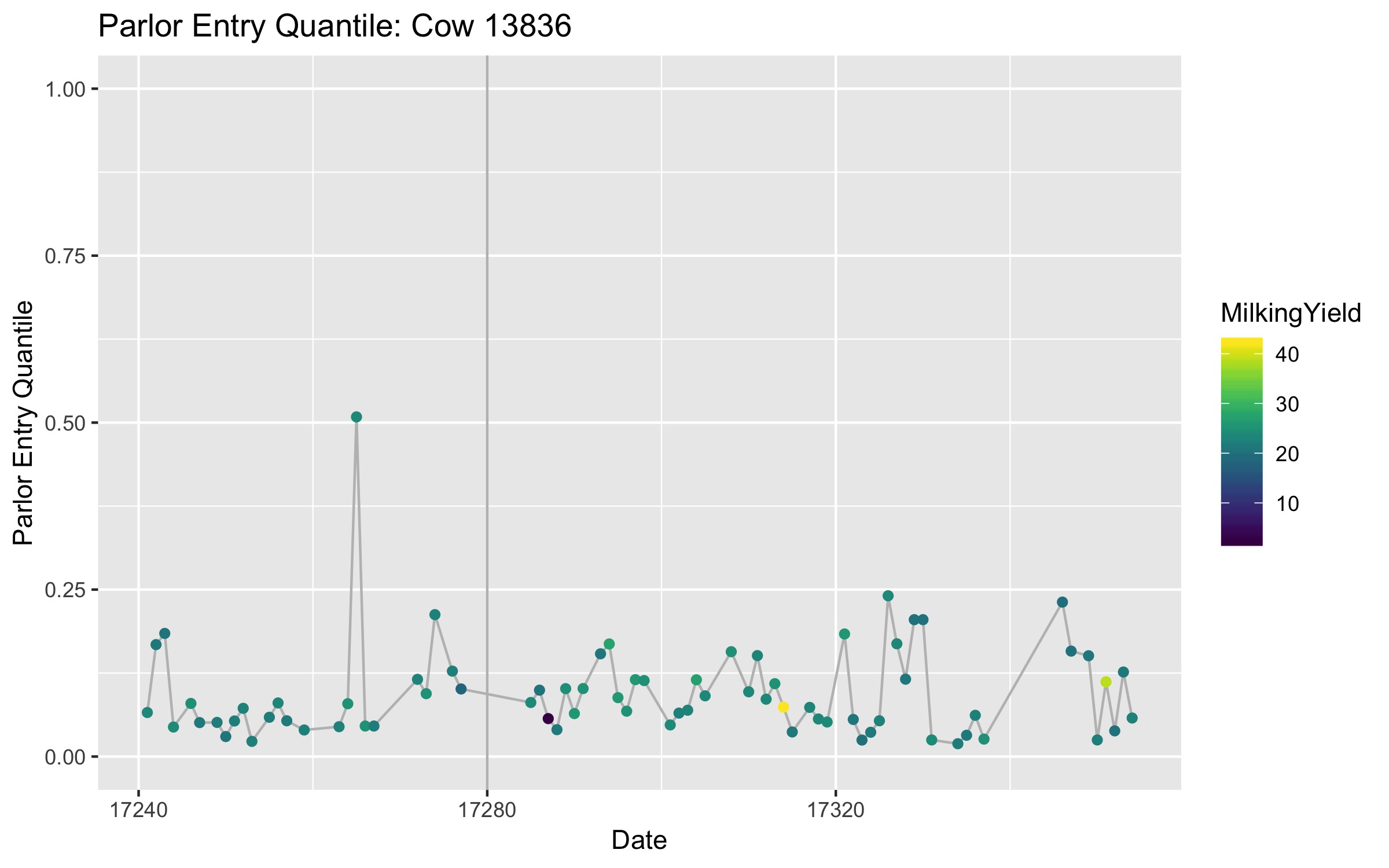

Supplement: Supplementary file 2 [file Data_Sheet_2.ZIP › Milking Yield/Cow_13836.jpg]

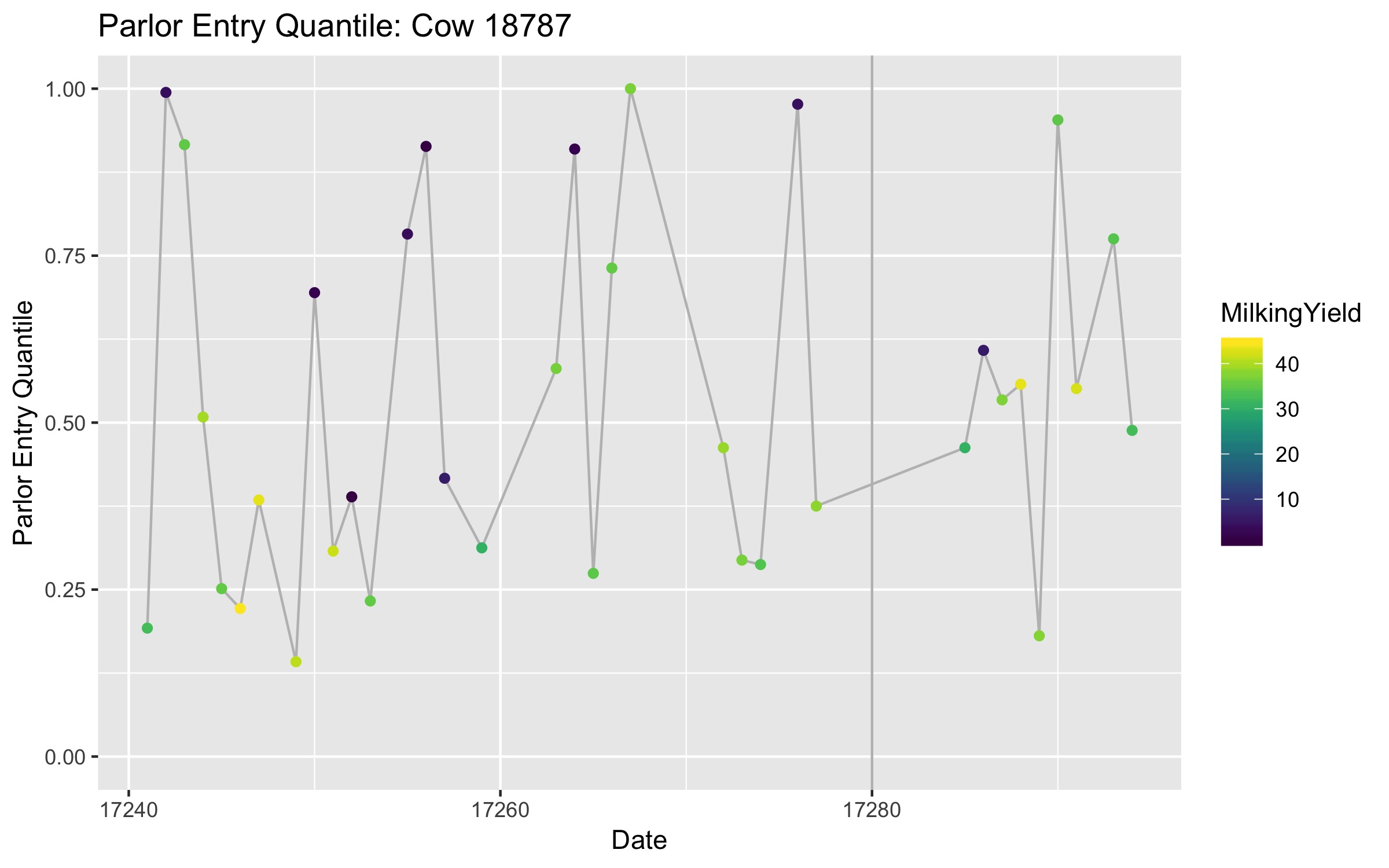

Supplement: Supplementary file 2 [file Data_Sheet_2.ZIP › Milking Yield/Cow_18787.jpg]

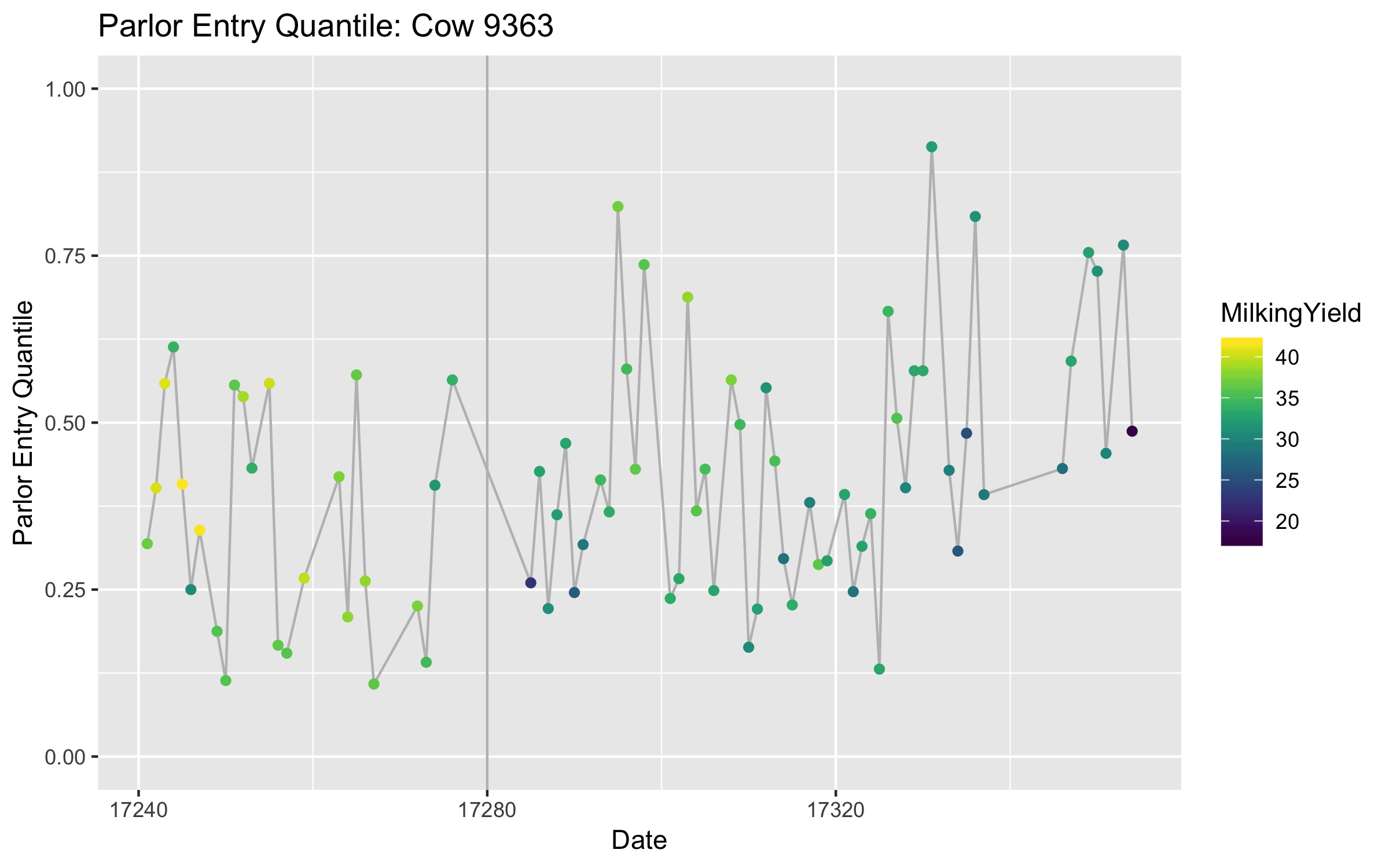

Supplement: Supplementary file 2 [file Data_Sheet_2.ZIP › Milking Yield/Cow_9363.jpg]

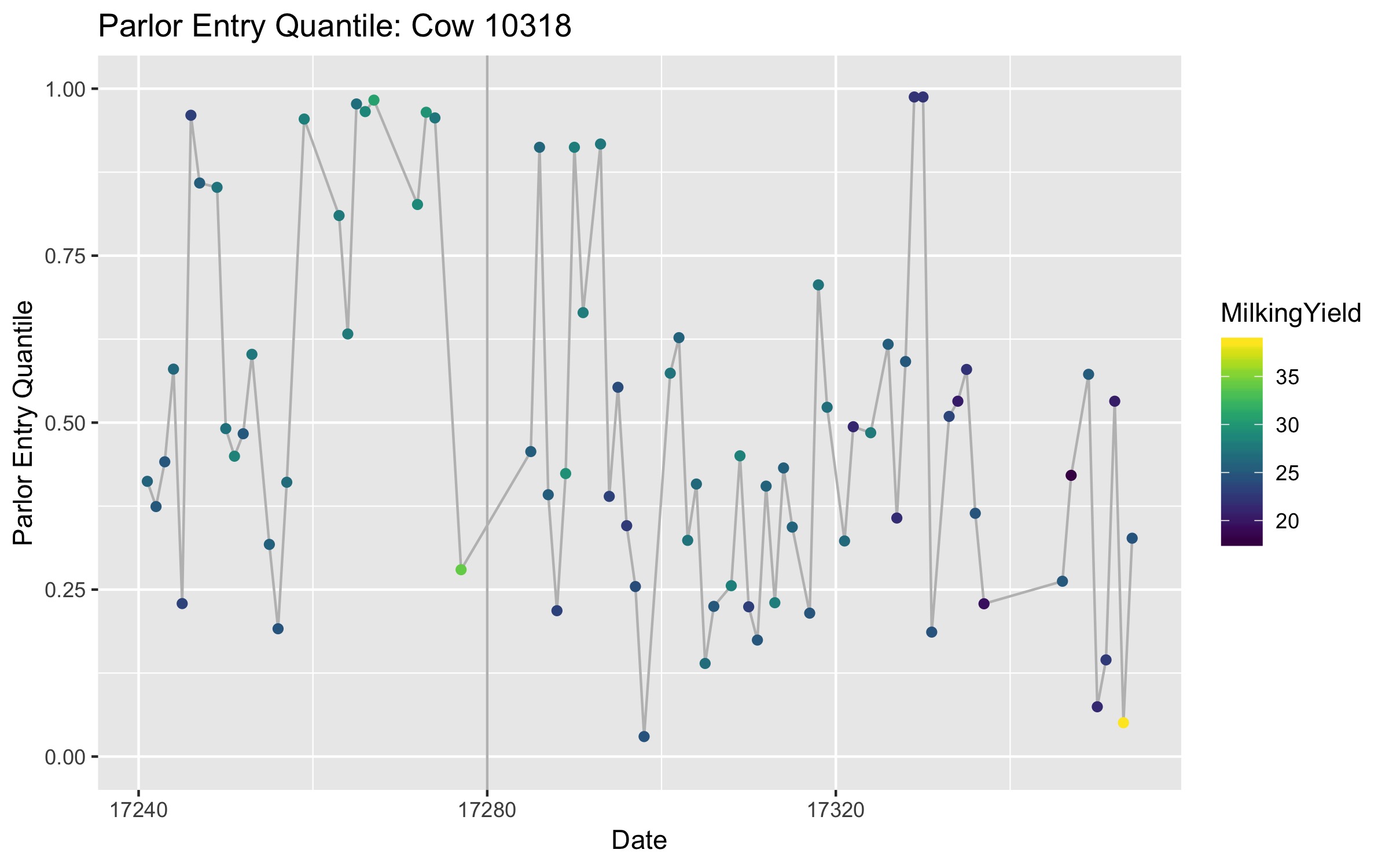

Supplement: Supplementary file 2 [file Data_Sheet_2.ZIP › Milking Yield/Cow_10318.jpg]

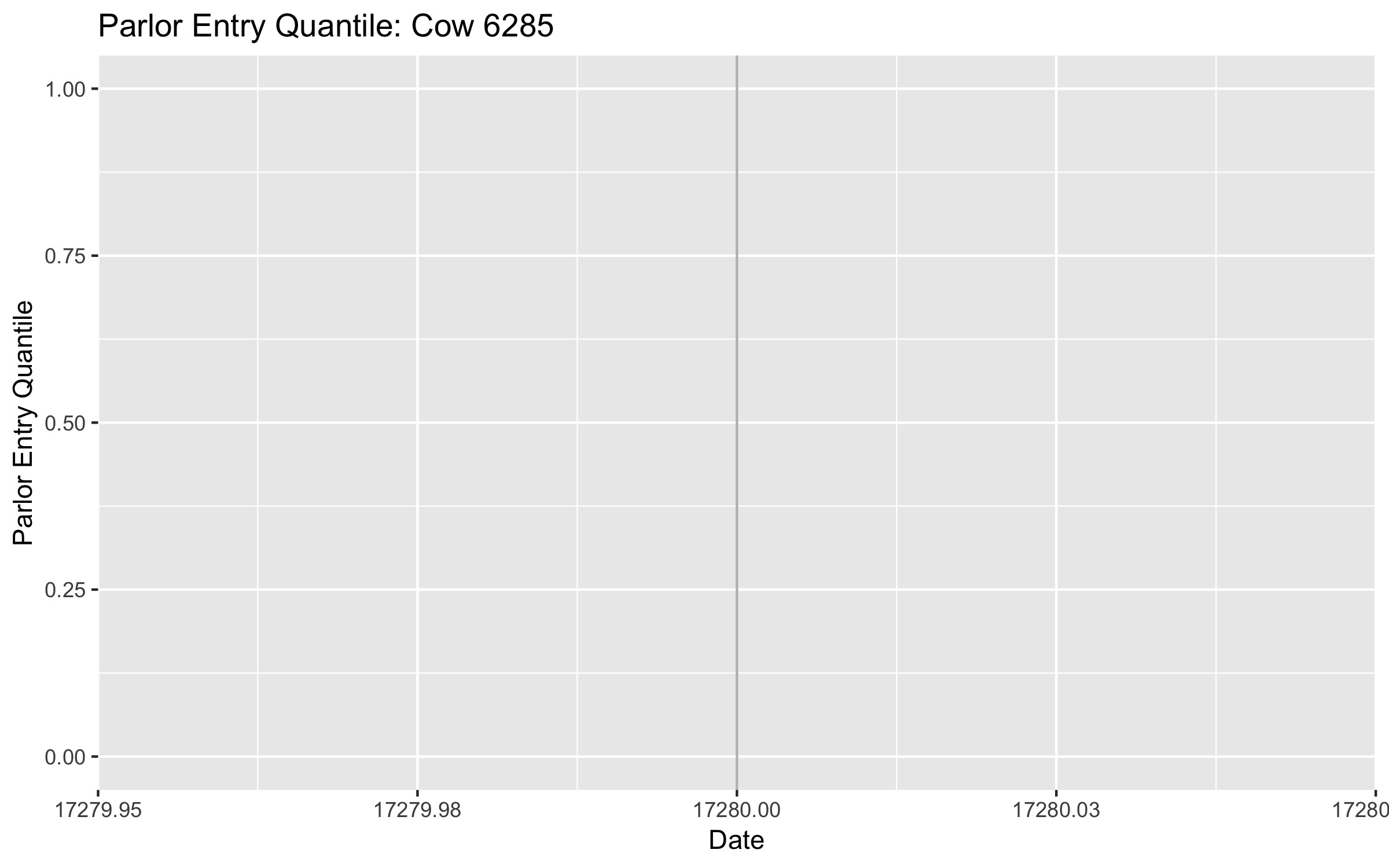

Supplement: Supplementary file 2 [file Data_Sheet_2.ZIP › Milking Yield/Cow_6285.jpg]

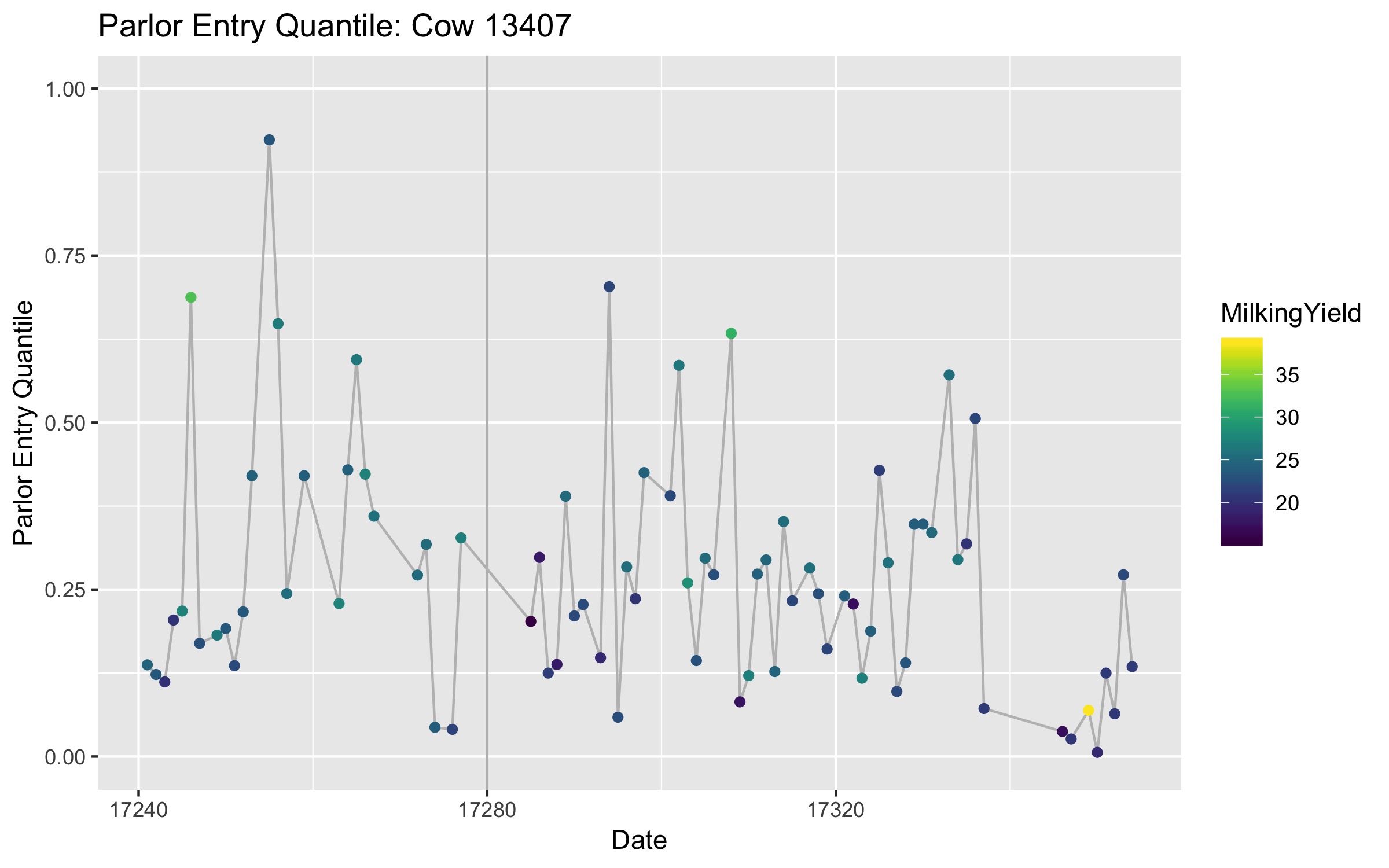

Supplement: Supplementary file 2 [file Data_Sheet_2.ZIP › Milking Yield/Cow_13407.jpg]

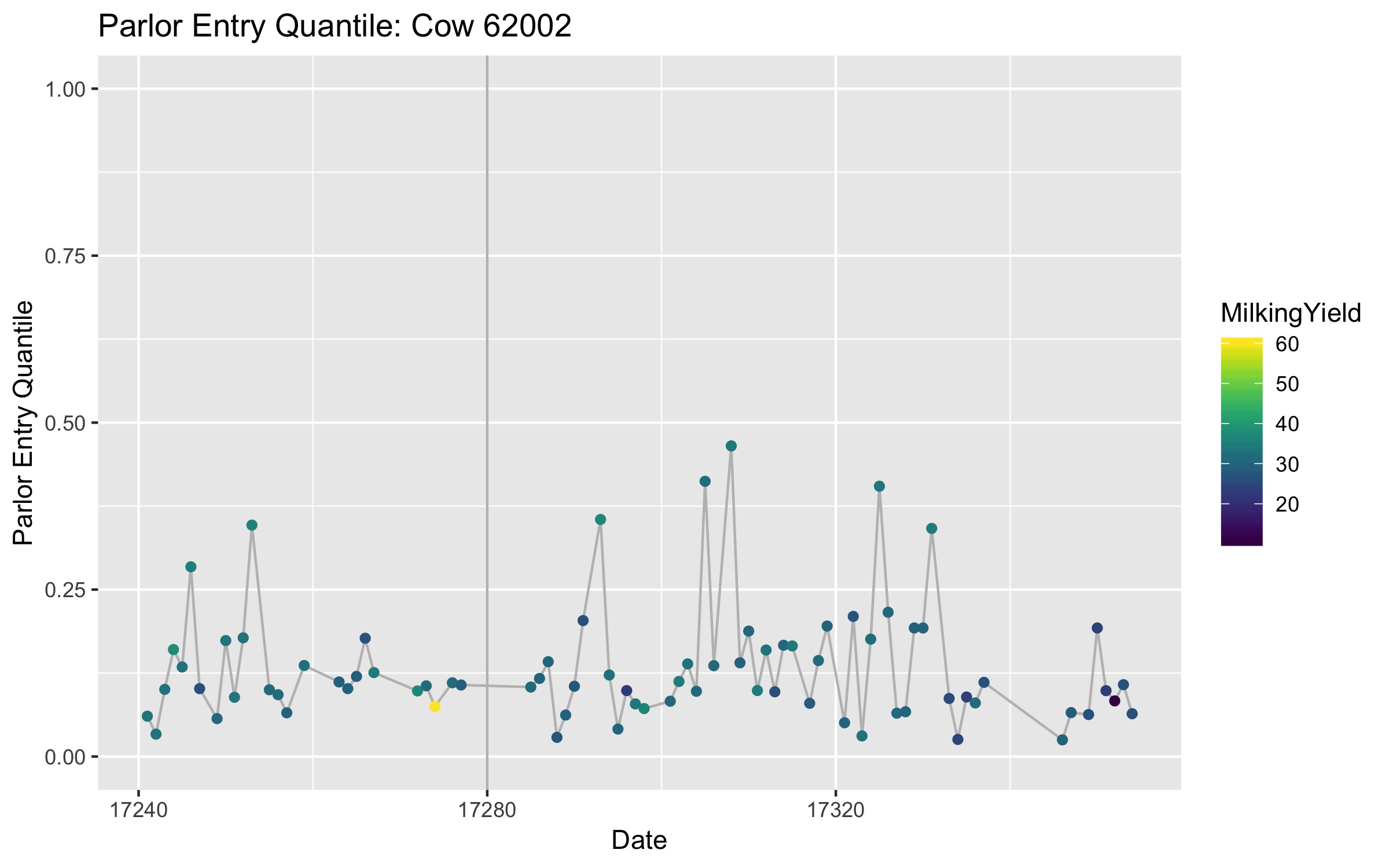

Supplement: Supplementary file 2 [file Data_Sheet_2.ZIP › Milking Yield/Cow_62002.jpg]

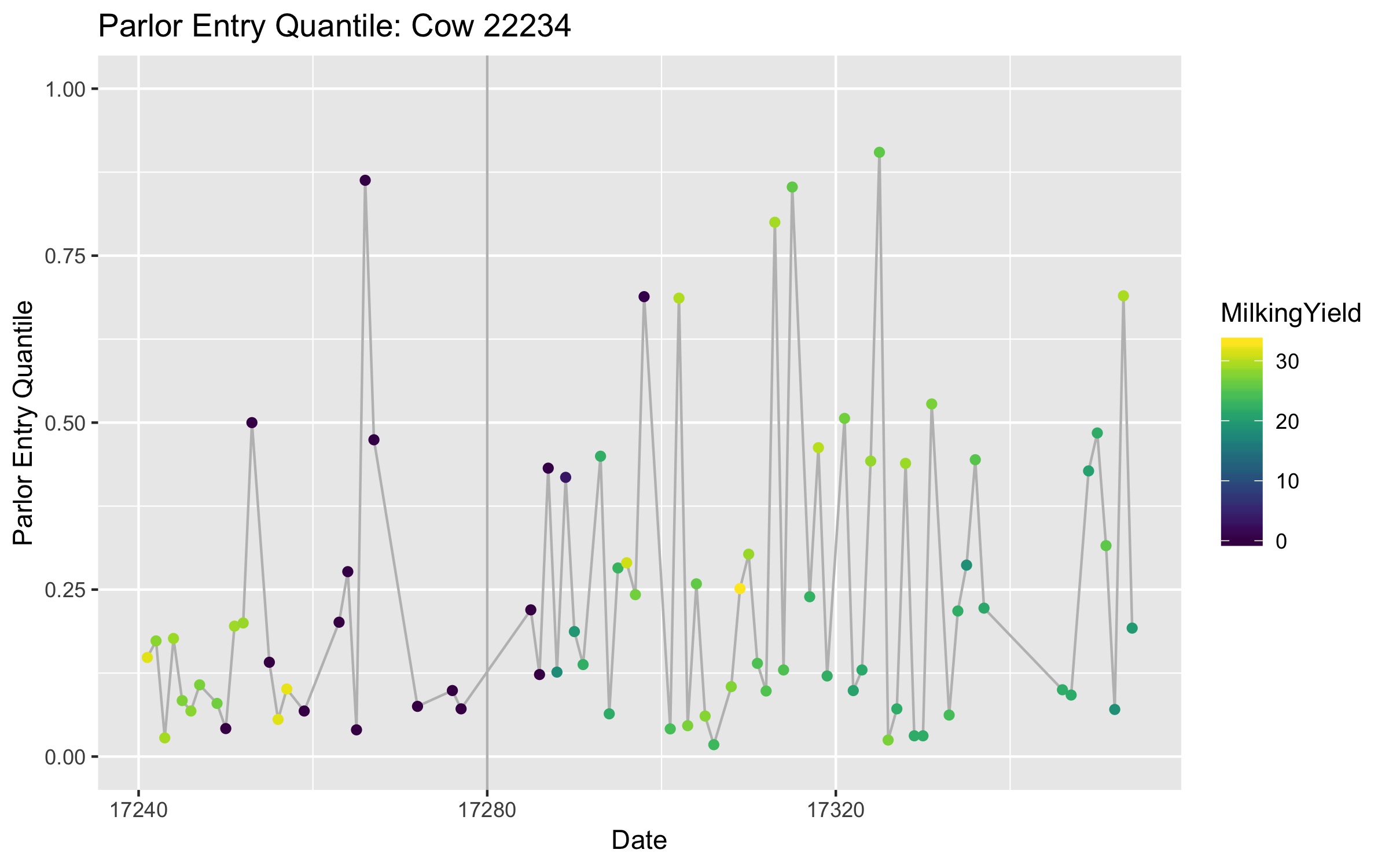

Supplement: Supplementary file 2 [file Data_Sheet_2.ZIP › Milking Yield/Cow_22234.jpg]

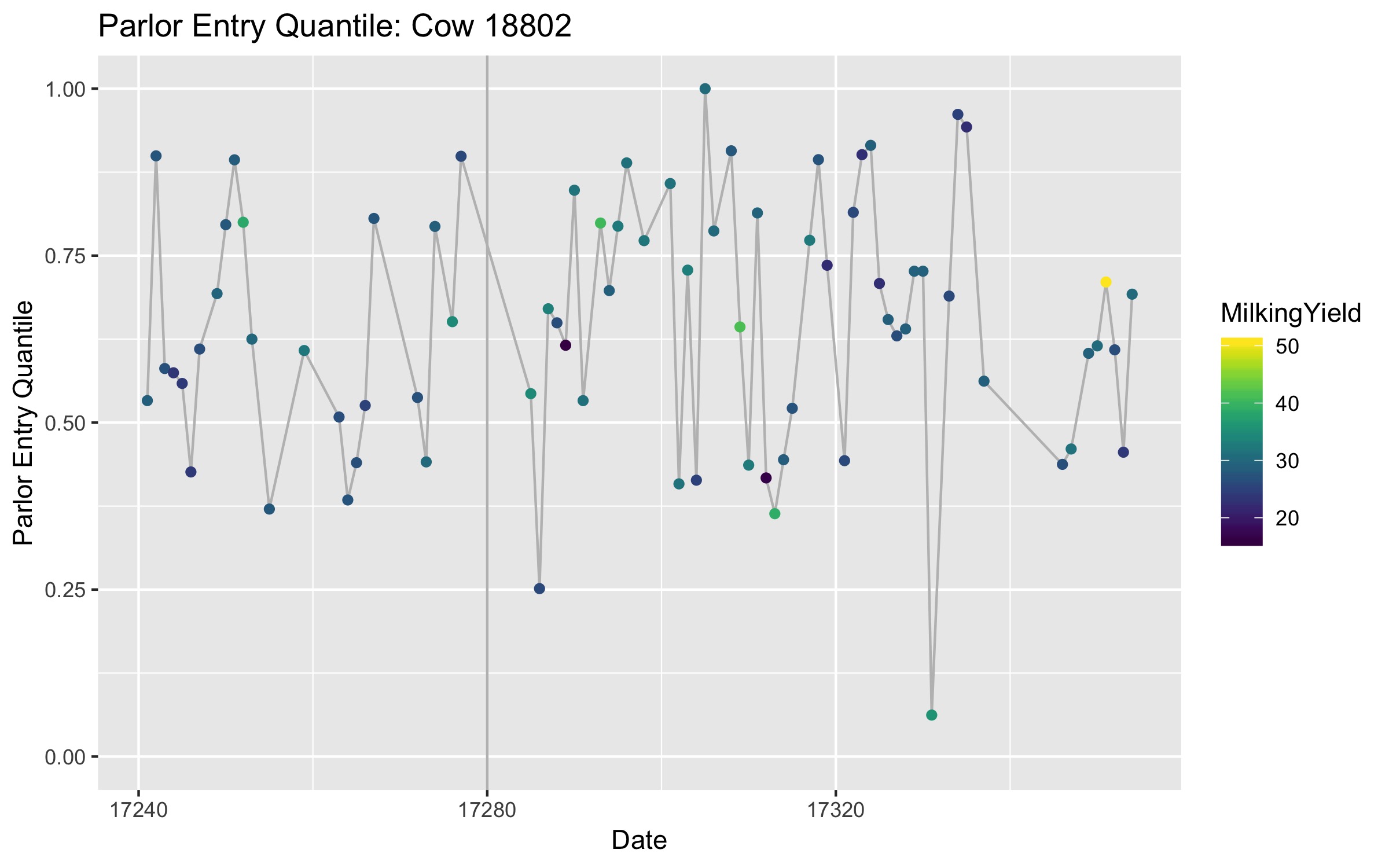

Supplement: Supplementary file 2 [file Data_Sheet_2.ZIP › Milking Yield/Cow_18802.jpg]

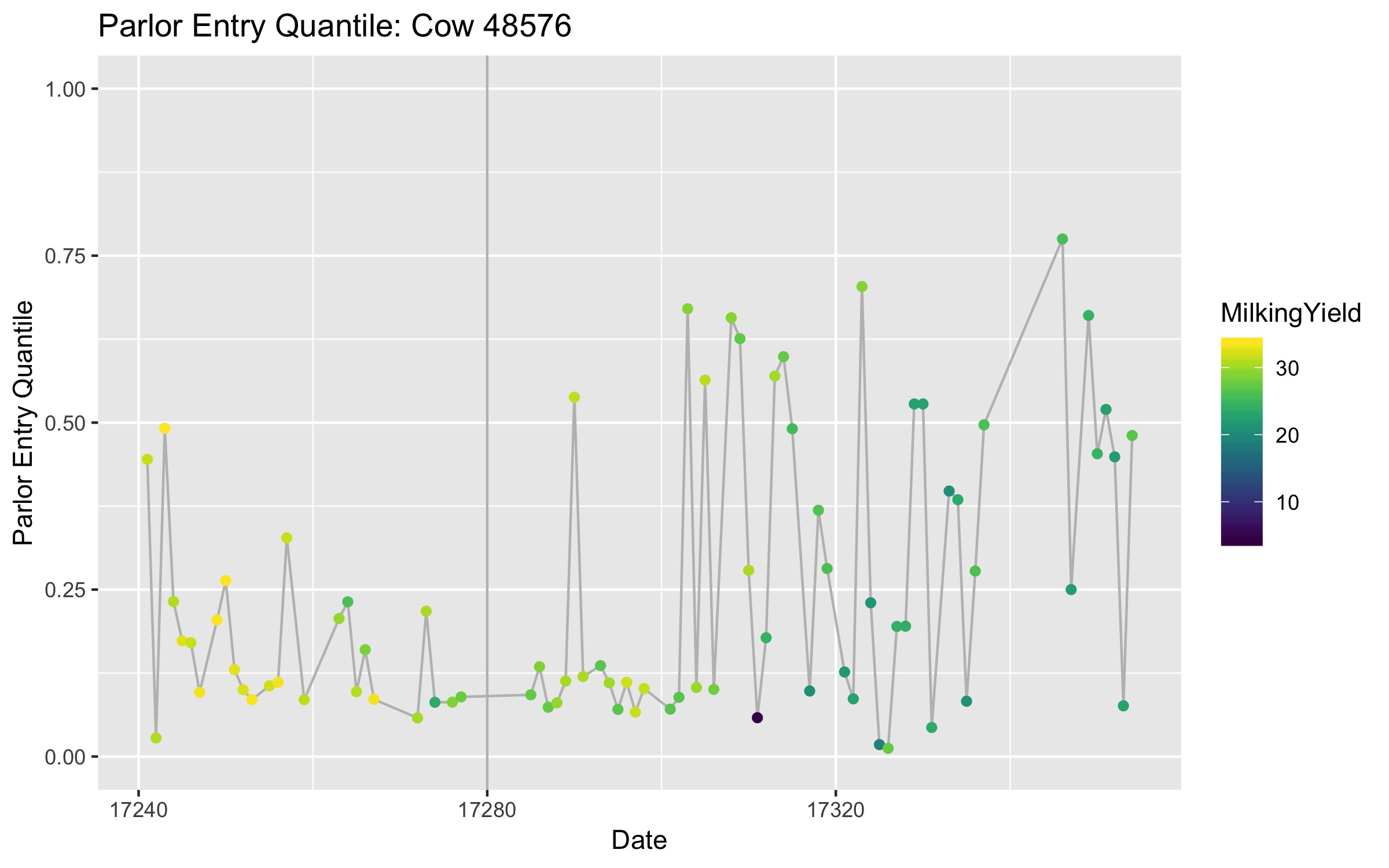

Supplement: Supplementary file 2 [file Data_Sheet_2.ZIP › Milking Yield/Cow_48576.jpg]

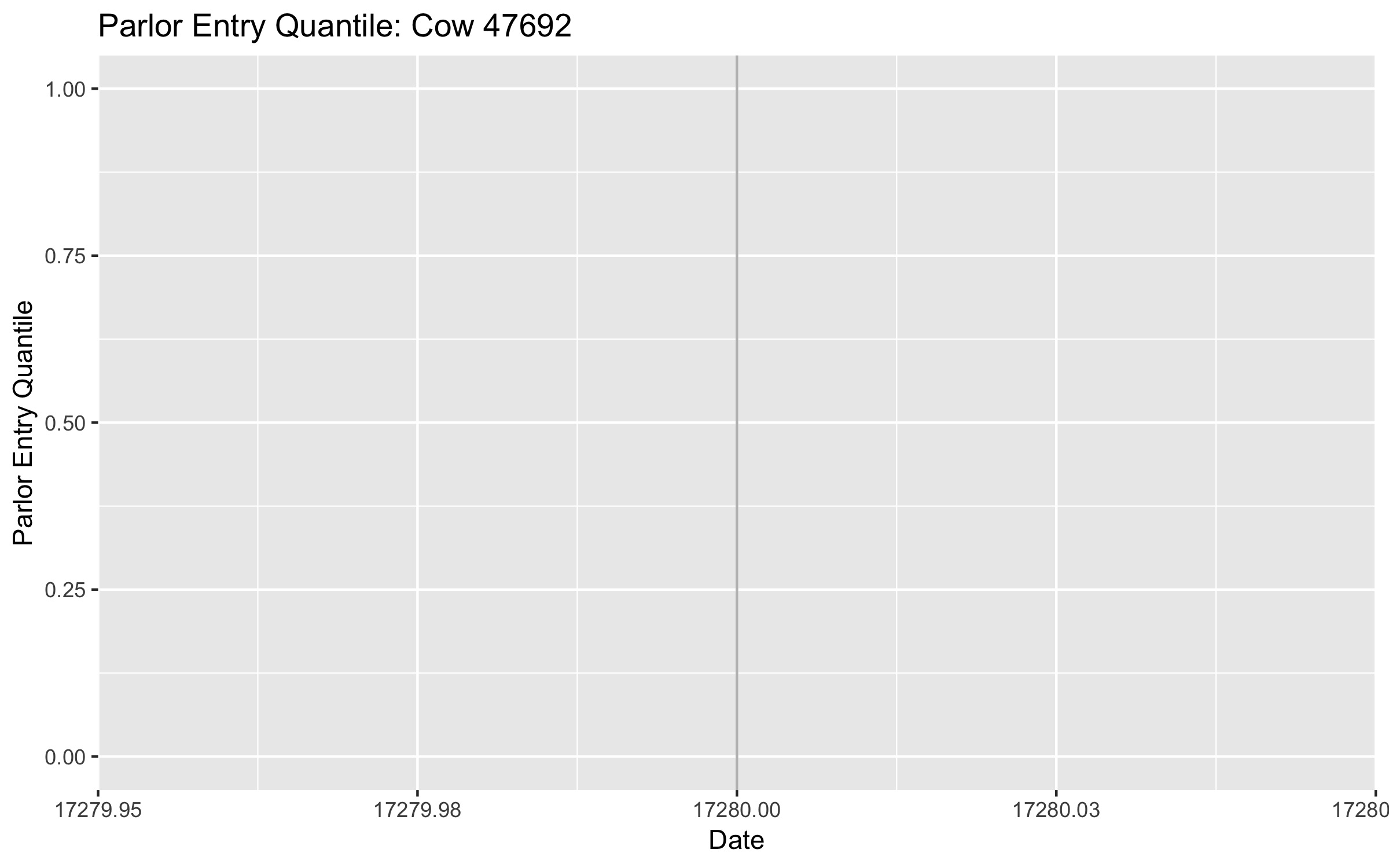

Supplement: Supplementary file 2 [file Data_Sheet_2.ZIP › Milking Yield/Cow_47692.jpg]

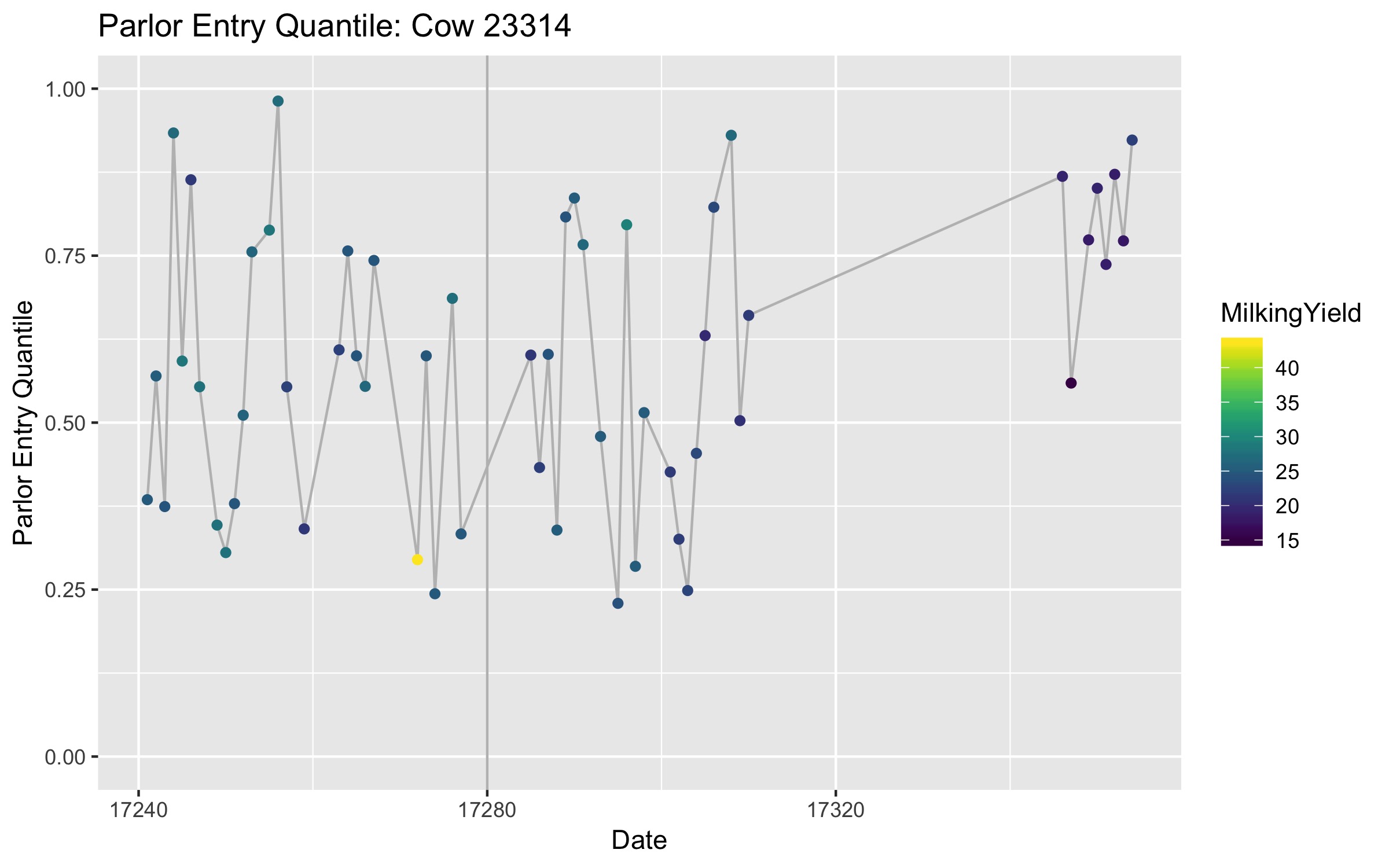

Supplement: Supplementary file 2 [file Data_Sheet_2.ZIP › Milking Yield/Cow_23314.jpg]

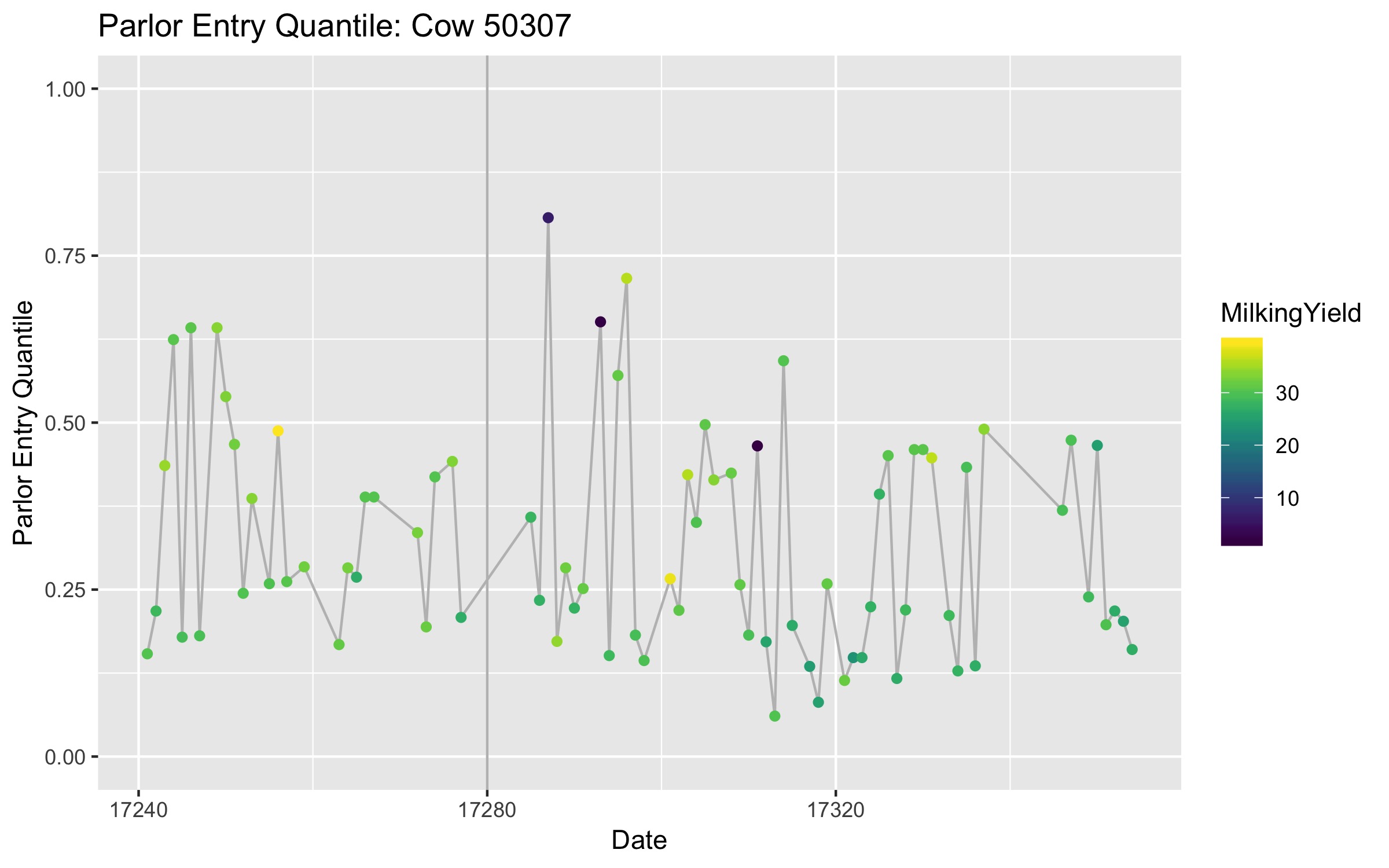

Supplement: Supplementary file 2 [file Data_Sheet_2.ZIP › Milking Yield/Cow_50307.jpg]

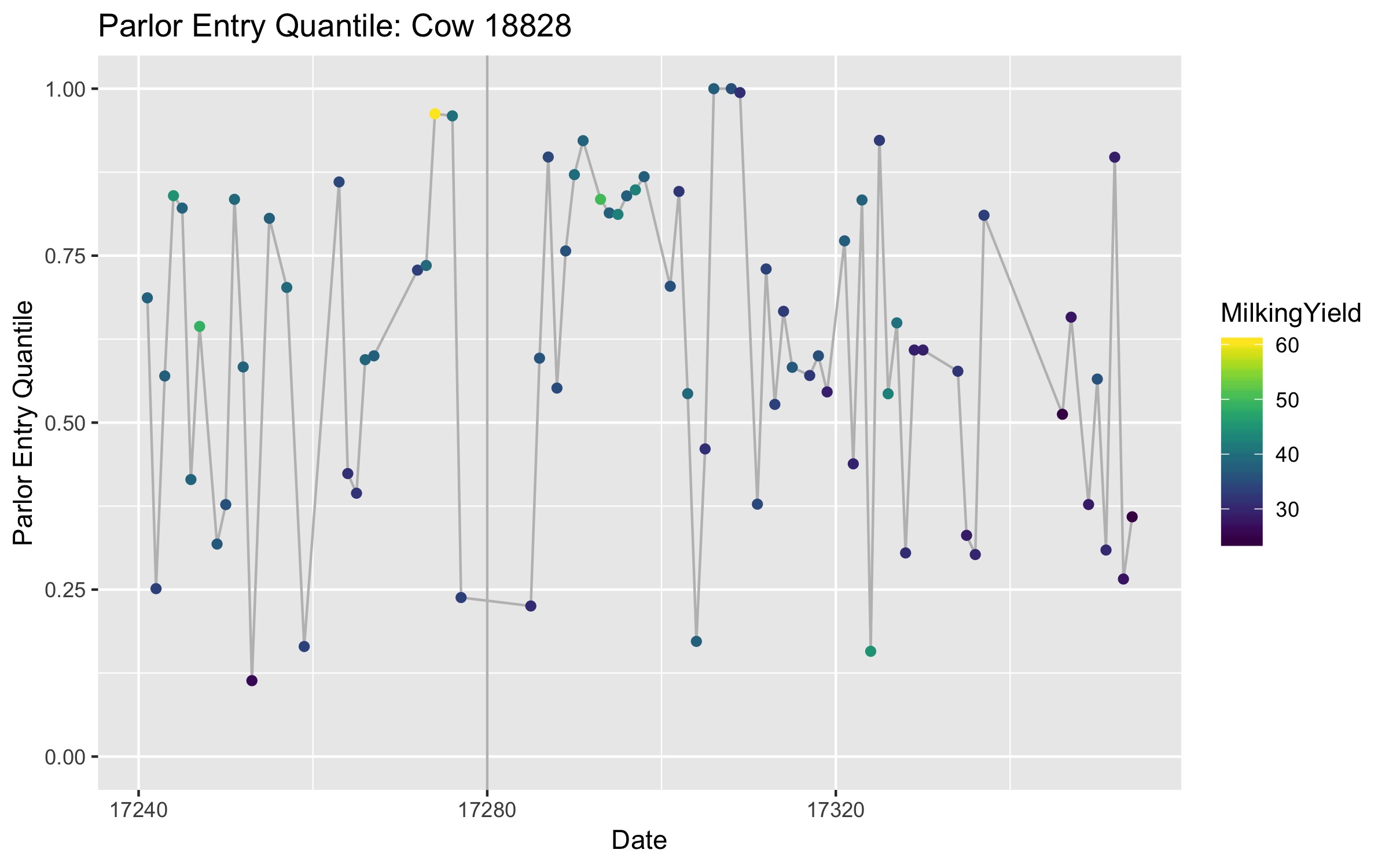

Supplement: Supplementary file 2 [file Data_Sheet_2.ZIP › Milking Yield/Cow_18828.jpg]

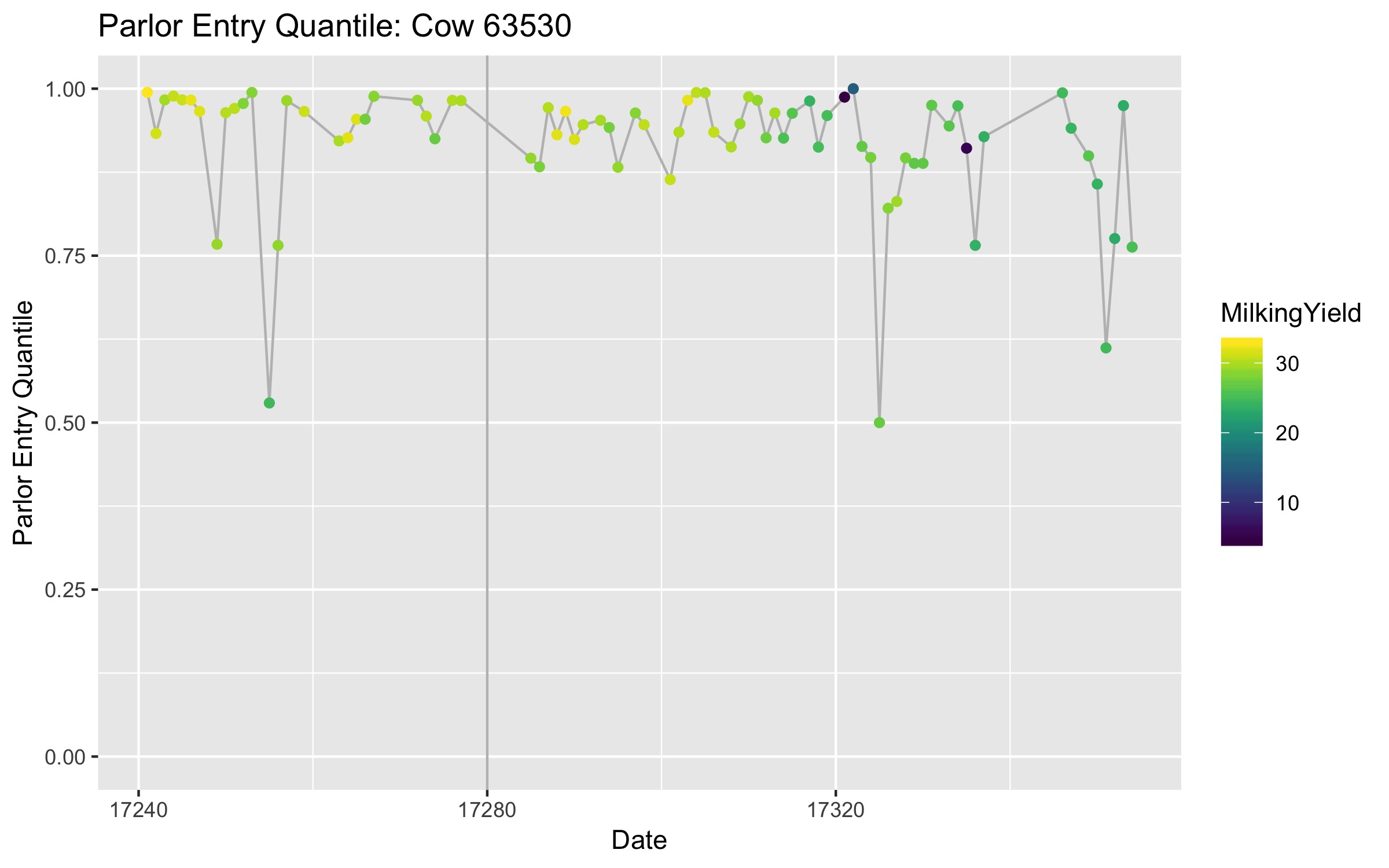

Supplement: Supplementary file 2 [file Data_Sheet_2.ZIP › Milking Yield/Cow_63530.jpg]

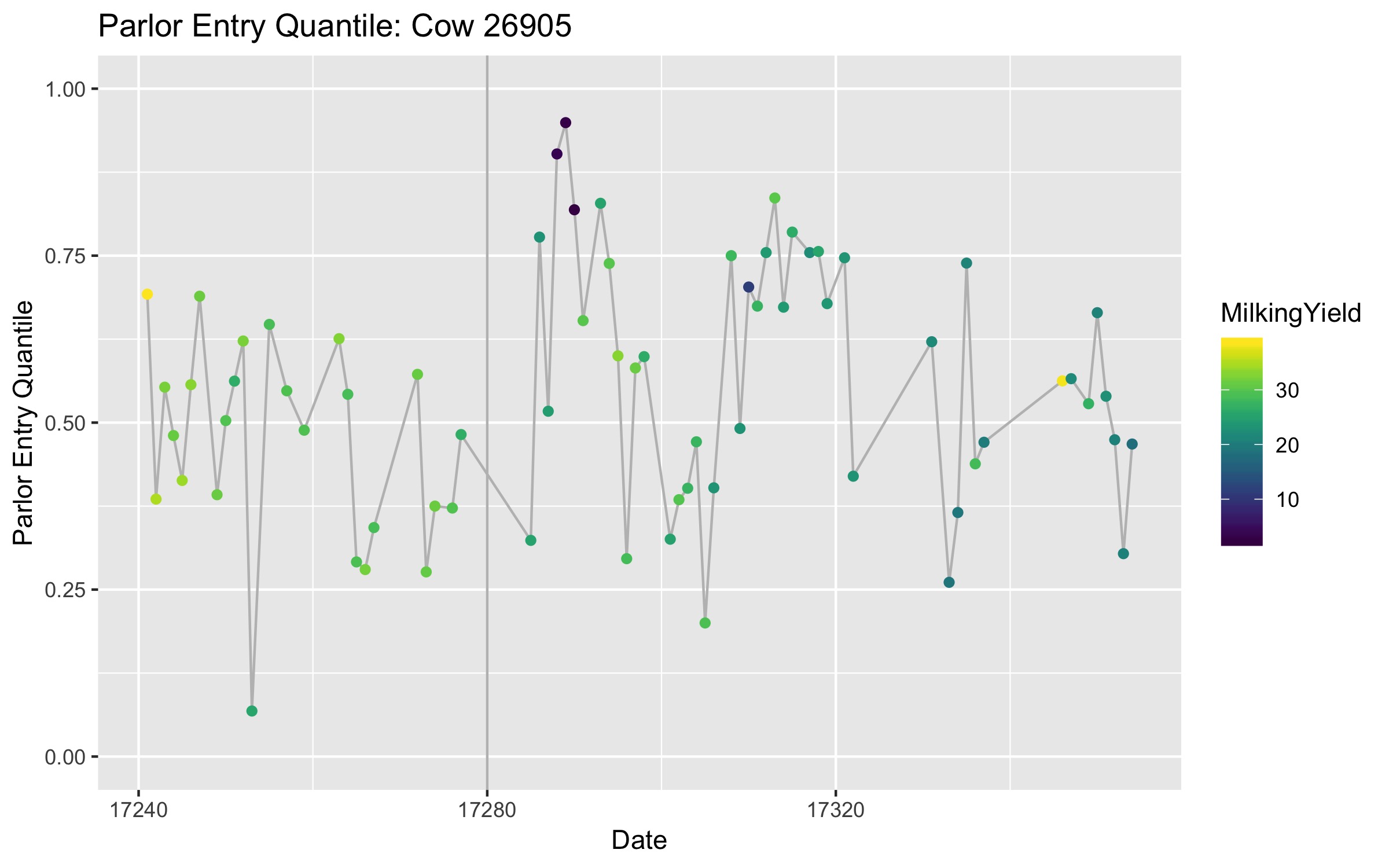

Supplement: Supplementary file 2 [file Data_Sheet_2.ZIP › Milking Yield/Cow_26905.jpg]

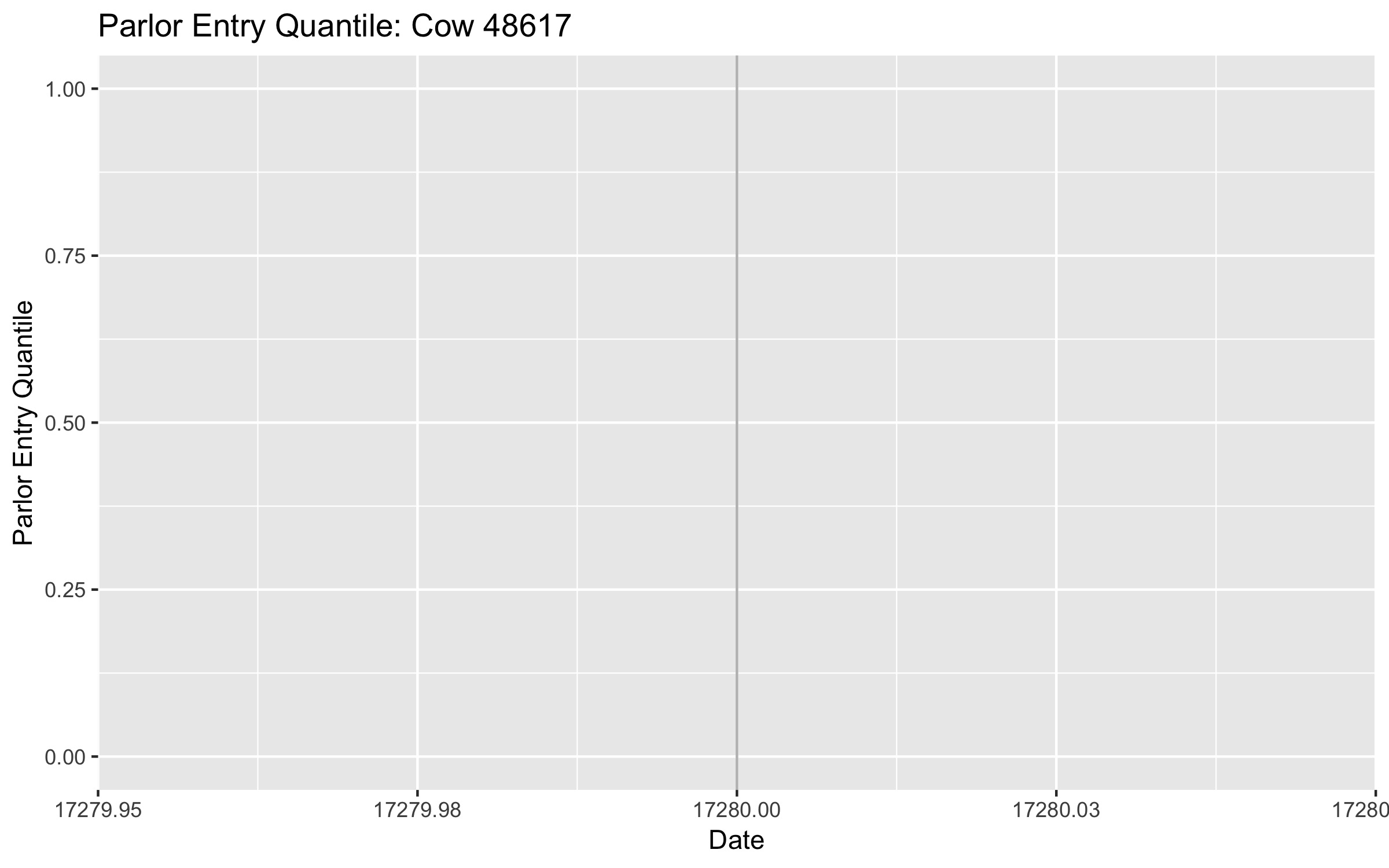

Supplement: Supplementary file 2 [file Data_Sheet_2.ZIP › Milking Yield/Cow_48617.jpg]

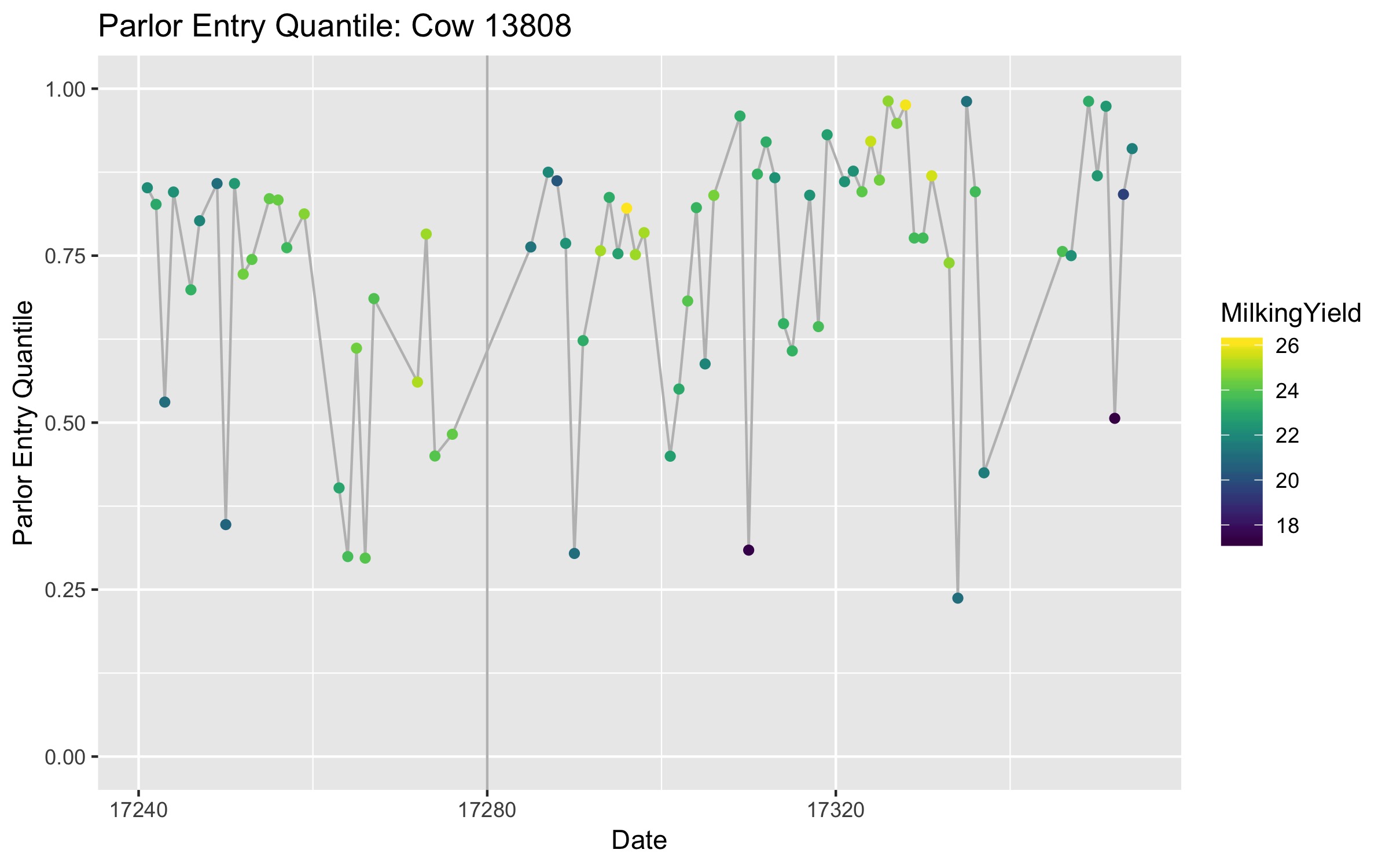

Supplement: Supplementary file 2 [file Data_Sheet_2.ZIP › Milking Yield/Cow_13808.jpg]

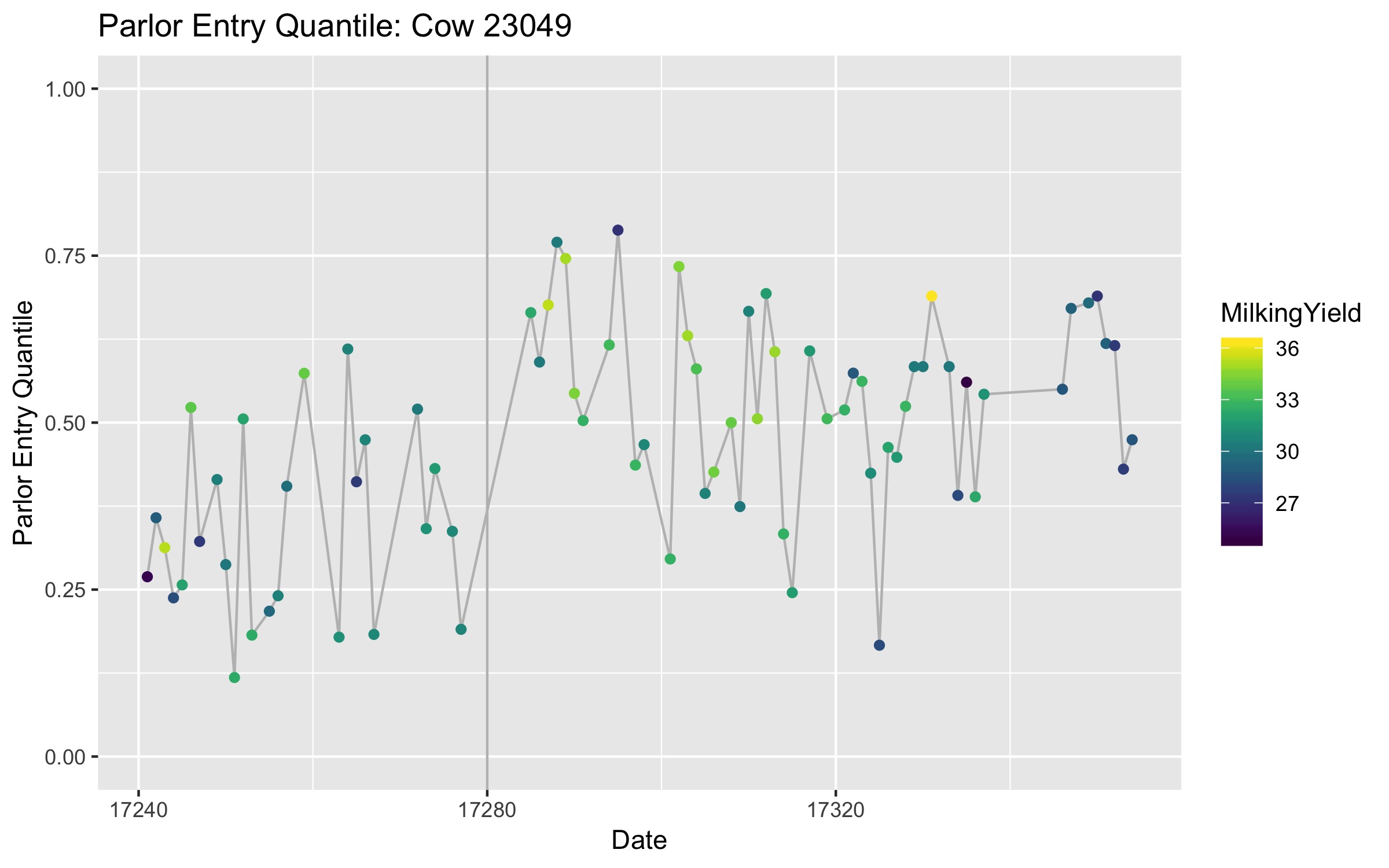

Supplement: Supplementary file 2 [file Data_Sheet_2.ZIP › Milking Yield/Cow_23049.jpg]

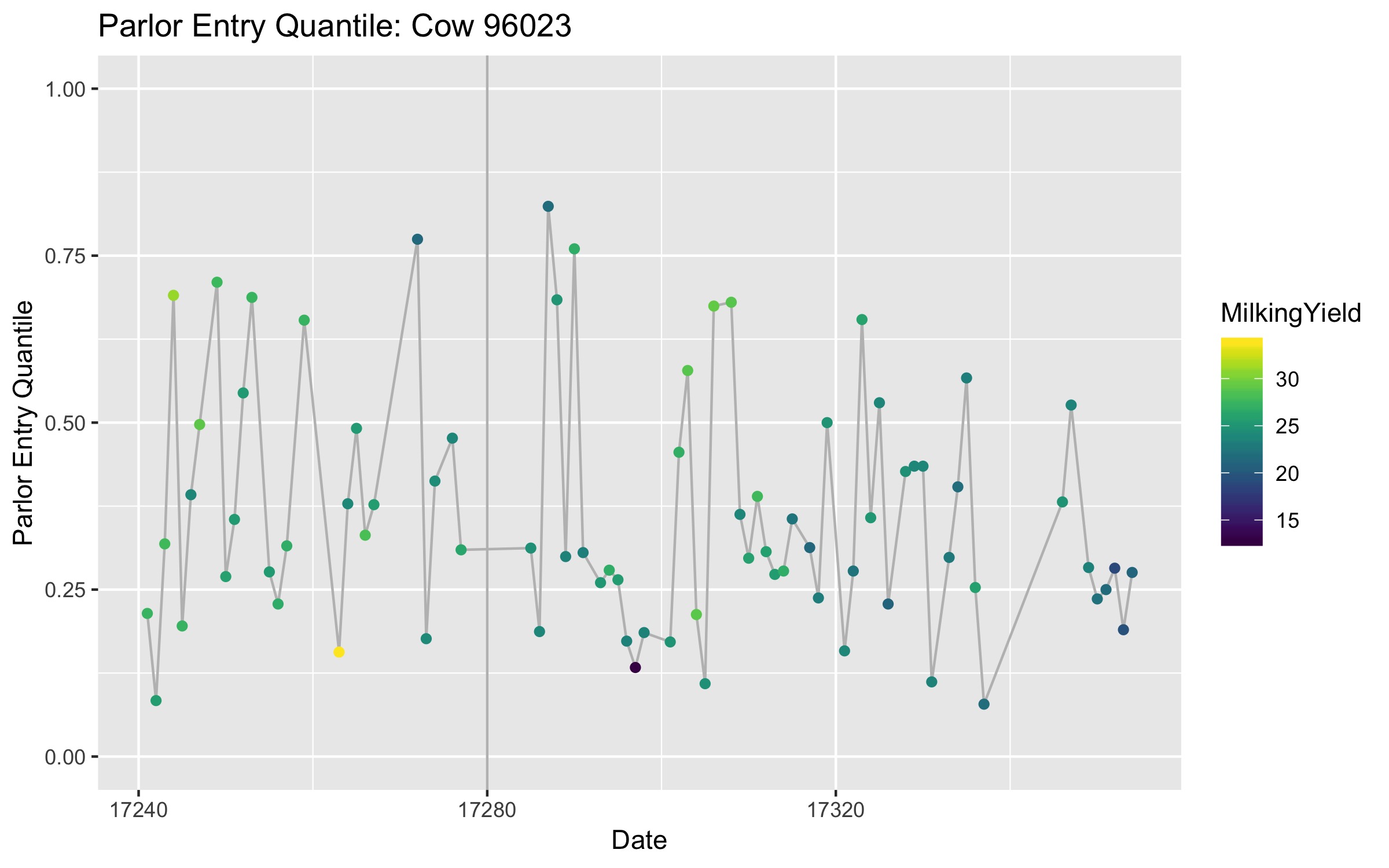

Supplement: Supplementary file 2 [file Data_Sheet_2.ZIP › Milking Yield/Cow_96023.jpg]

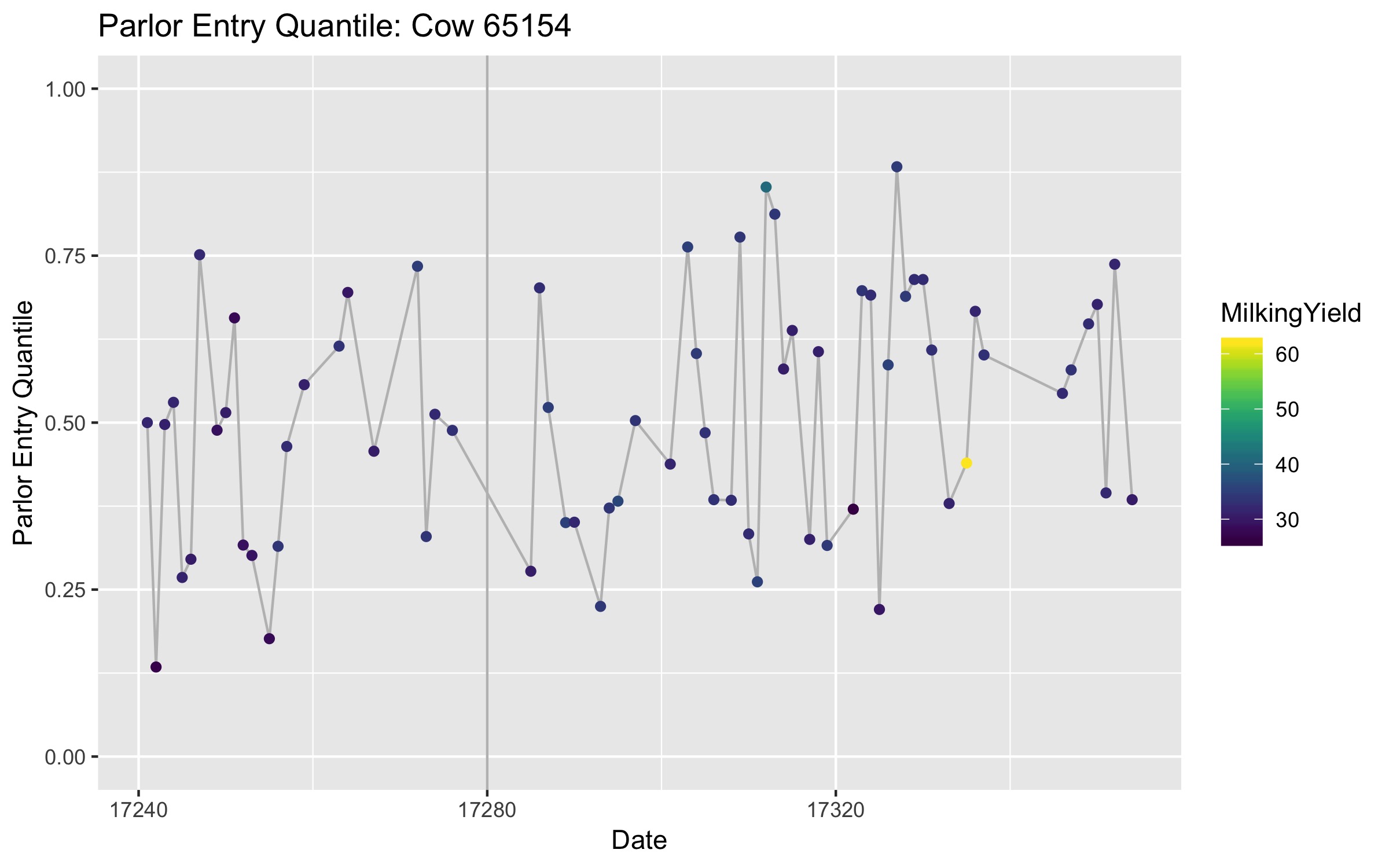

Supplement: Supplementary file 2 [file Data_Sheet_2.ZIP › Milking Yield/Cow_65154.jpg]

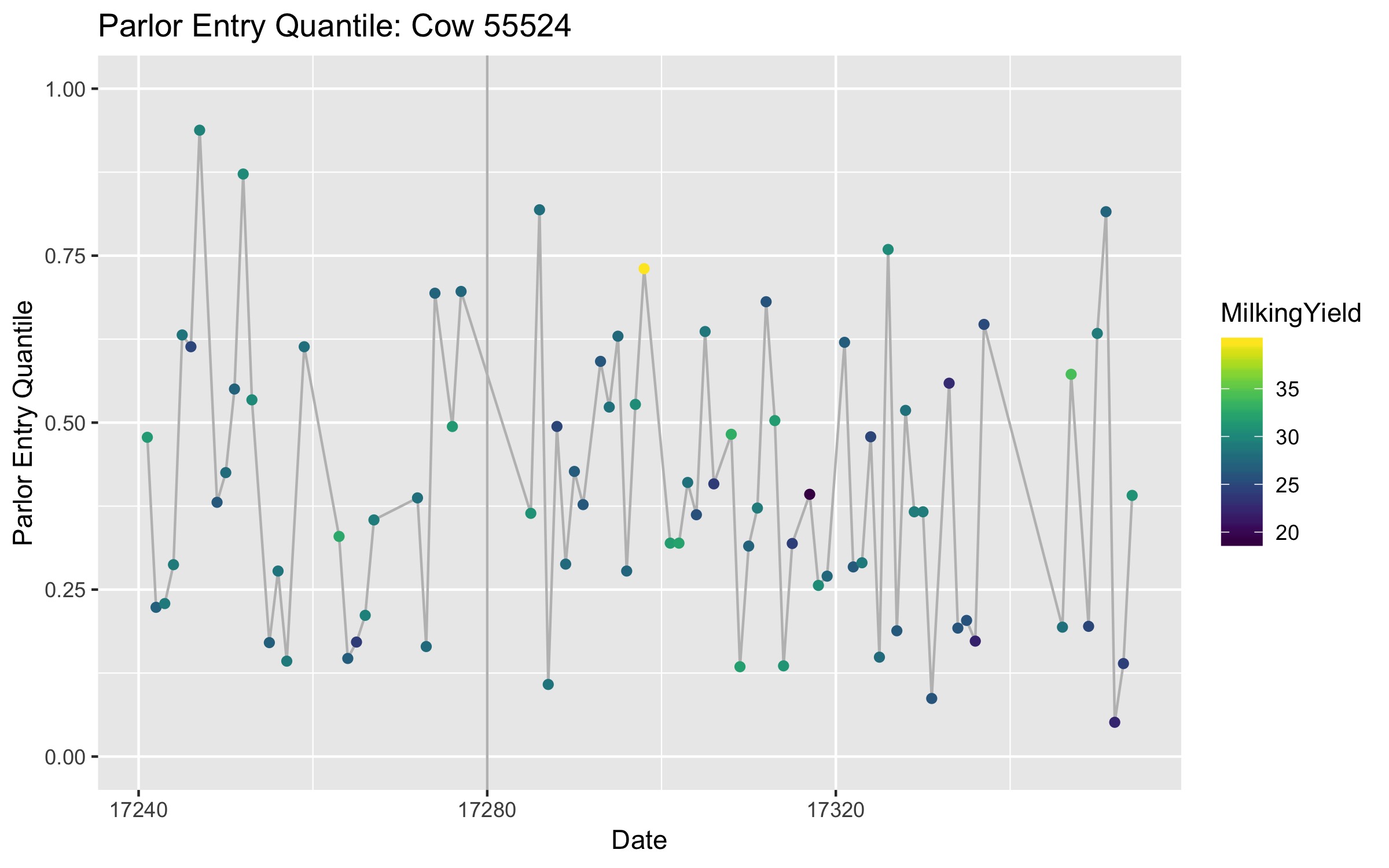

Supplement: Supplementary file 2 [file Data_Sheet_2.ZIP › Milking Yield/Cow_55524.jpg]
