# Supplementary figures and images for: Mind the Queue: A Case Study in Visualizing Heterogeneous Behavioral Patterns in Livestock Sensor Data Using Unsupervised Machine Learning Techniques (part 1 of 4)
Source: Front Vet Sci. 2020 Aug 13;7:523. doi: 10.3389/fvets.2020.00523 (PMC7518149; doi:10.3389/fvets.2020.00523)

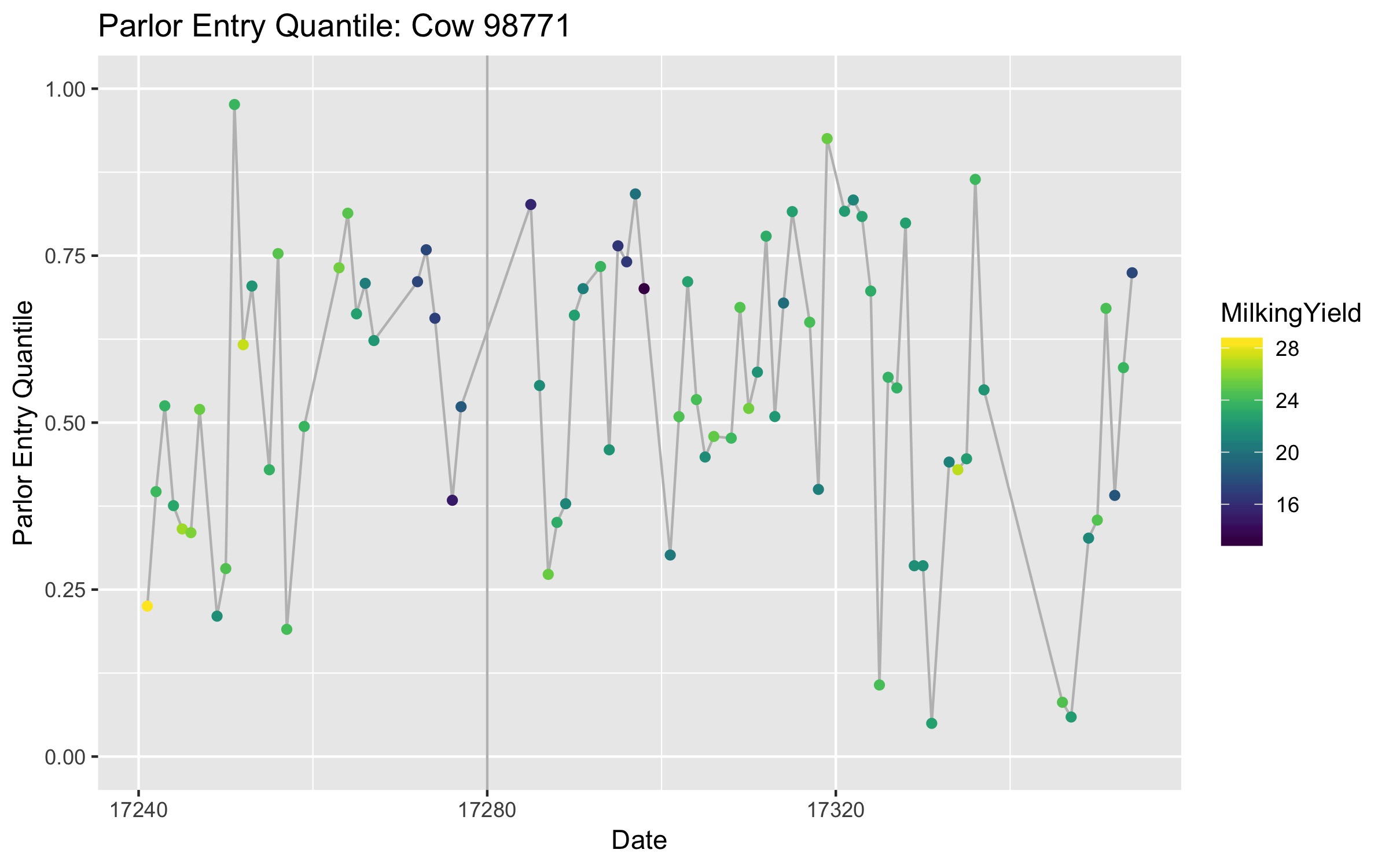

Supplement: Supplementary file 2 [file Data_Sheet_2.ZIP › Milking Yield/Cow_98771.jpg]

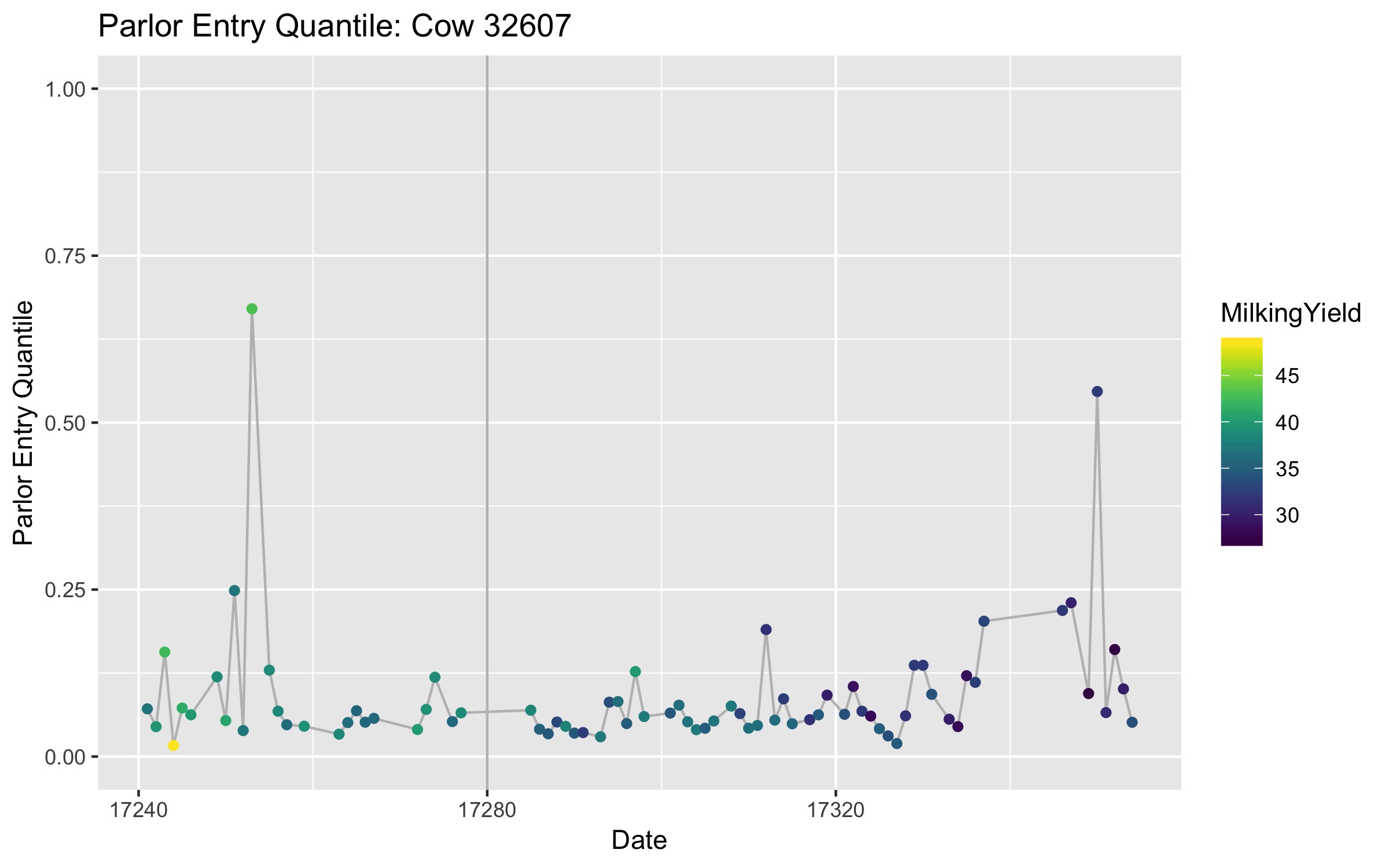

Supplement: Supplementary file 2 [file Data_Sheet_2.ZIP › Milking Yield/Cow_32607.jpg]

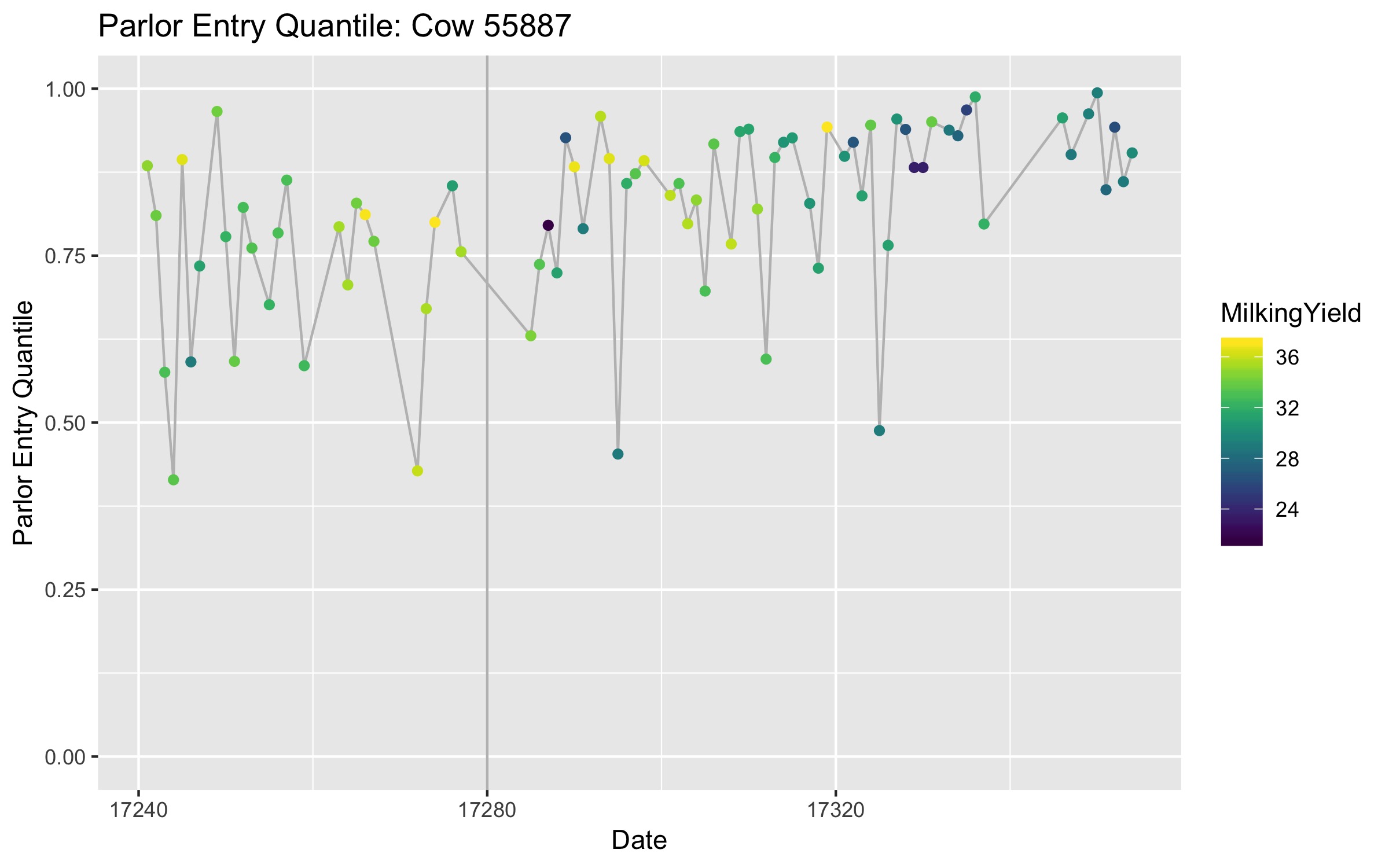

Supplement: Supplementary file 2 [file Data_Sheet_2.ZIP › Milking Yield/Cow_55887.jpg]

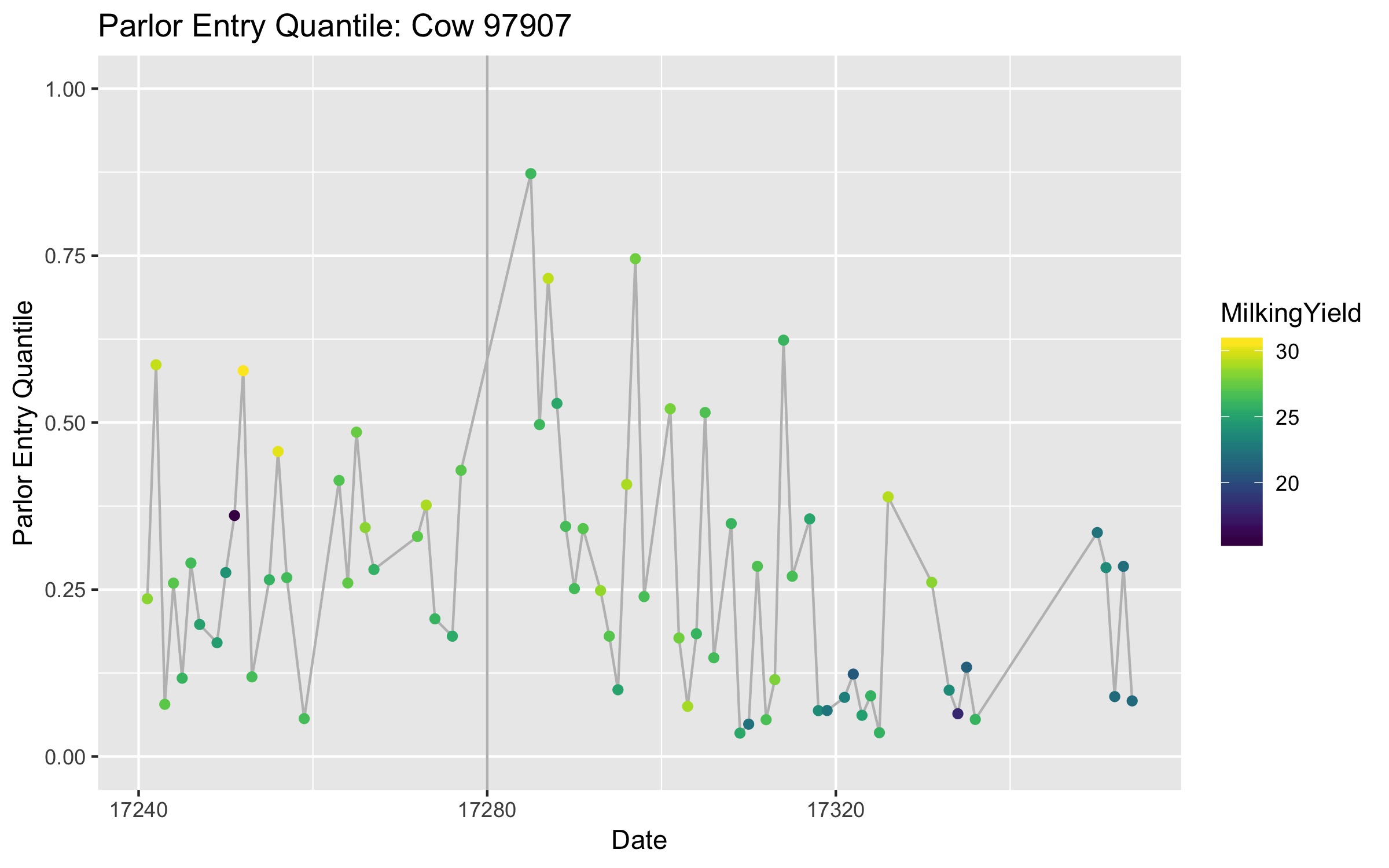

Supplement: Supplementary file 2 [file Data_Sheet_2.ZIP › Milking Yield/Cow_97907.jpg]

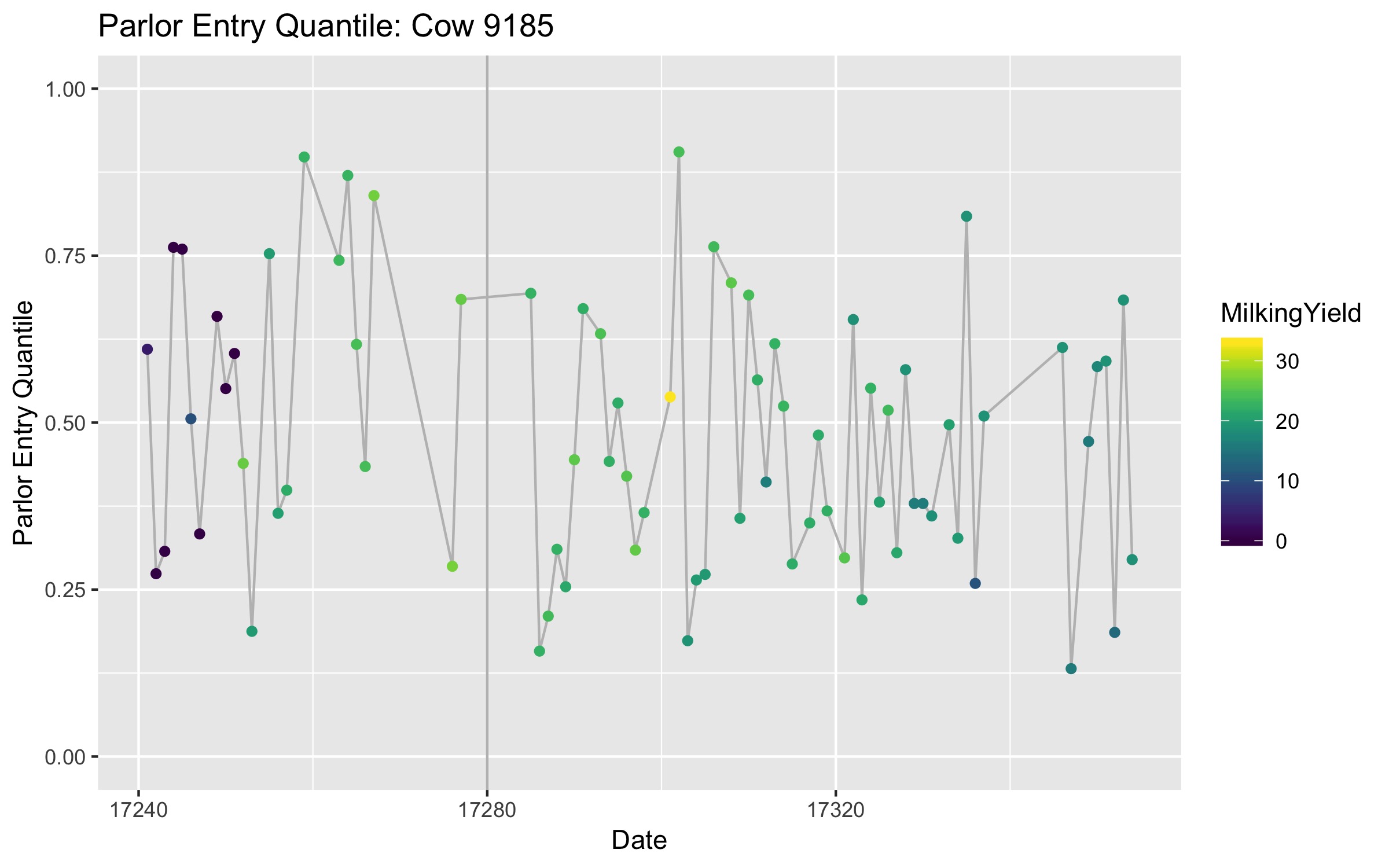

Supplement: Supplementary file 2 [file Data_Sheet_2.ZIP › Milking Yield/Cow_9185.jpg]

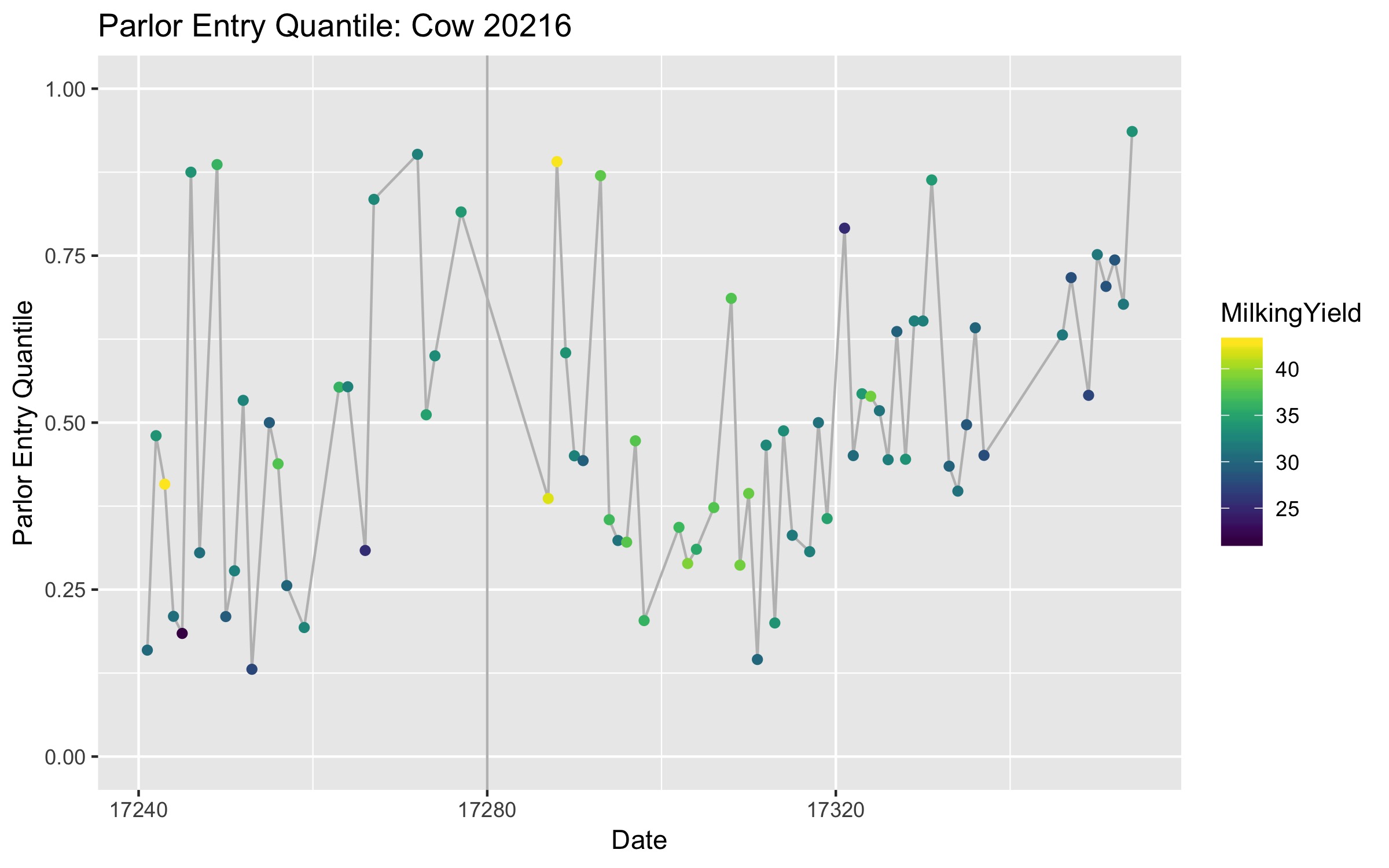

Supplement: Supplementary file 2 [file Data_Sheet_2.ZIP › Milking Yield/Cow_20216.jpg]

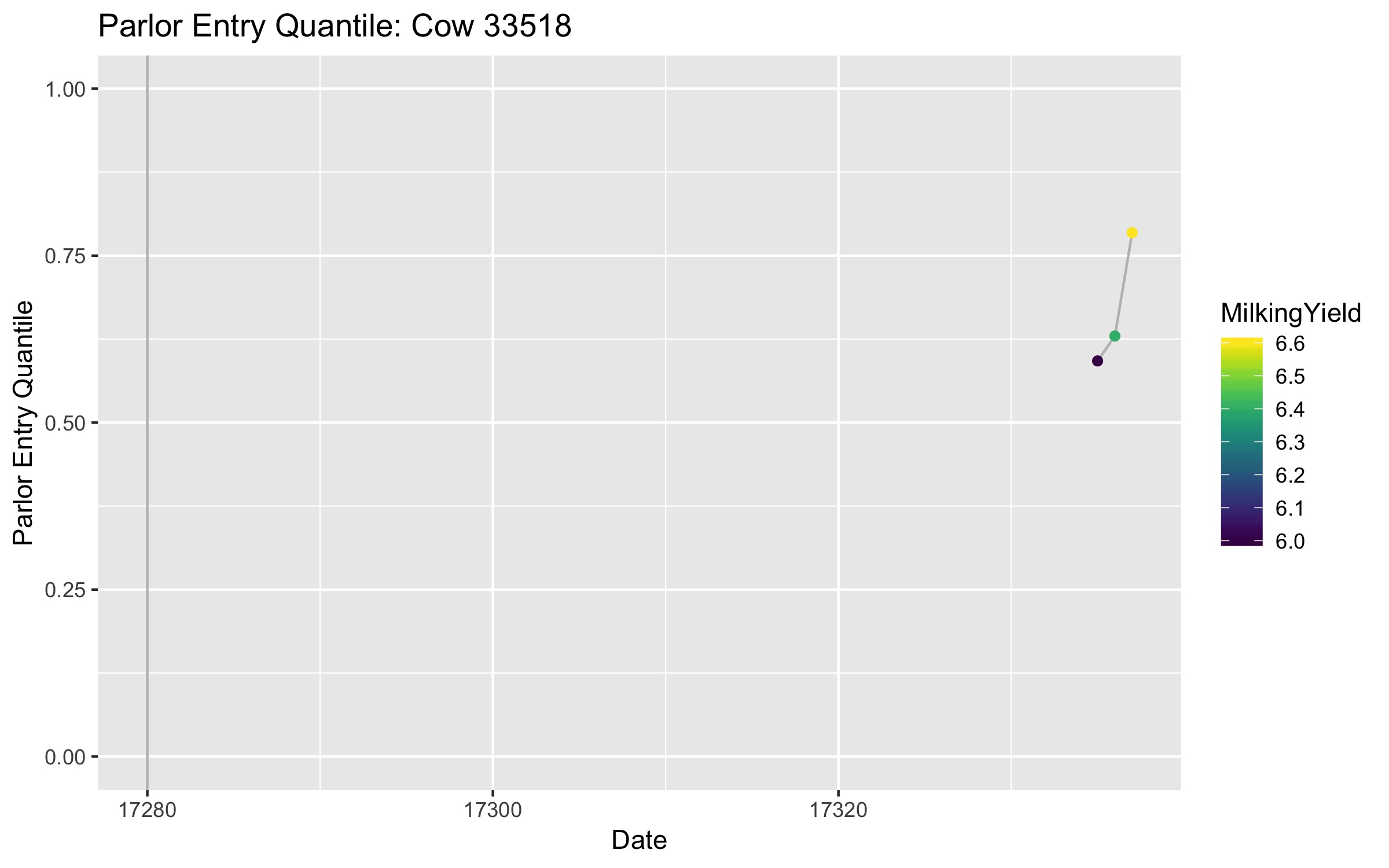

Supplement: Supplementary file 2 [file Data_Sheet_2.ZIP › Milking Yield/Cow_33518.jpg]

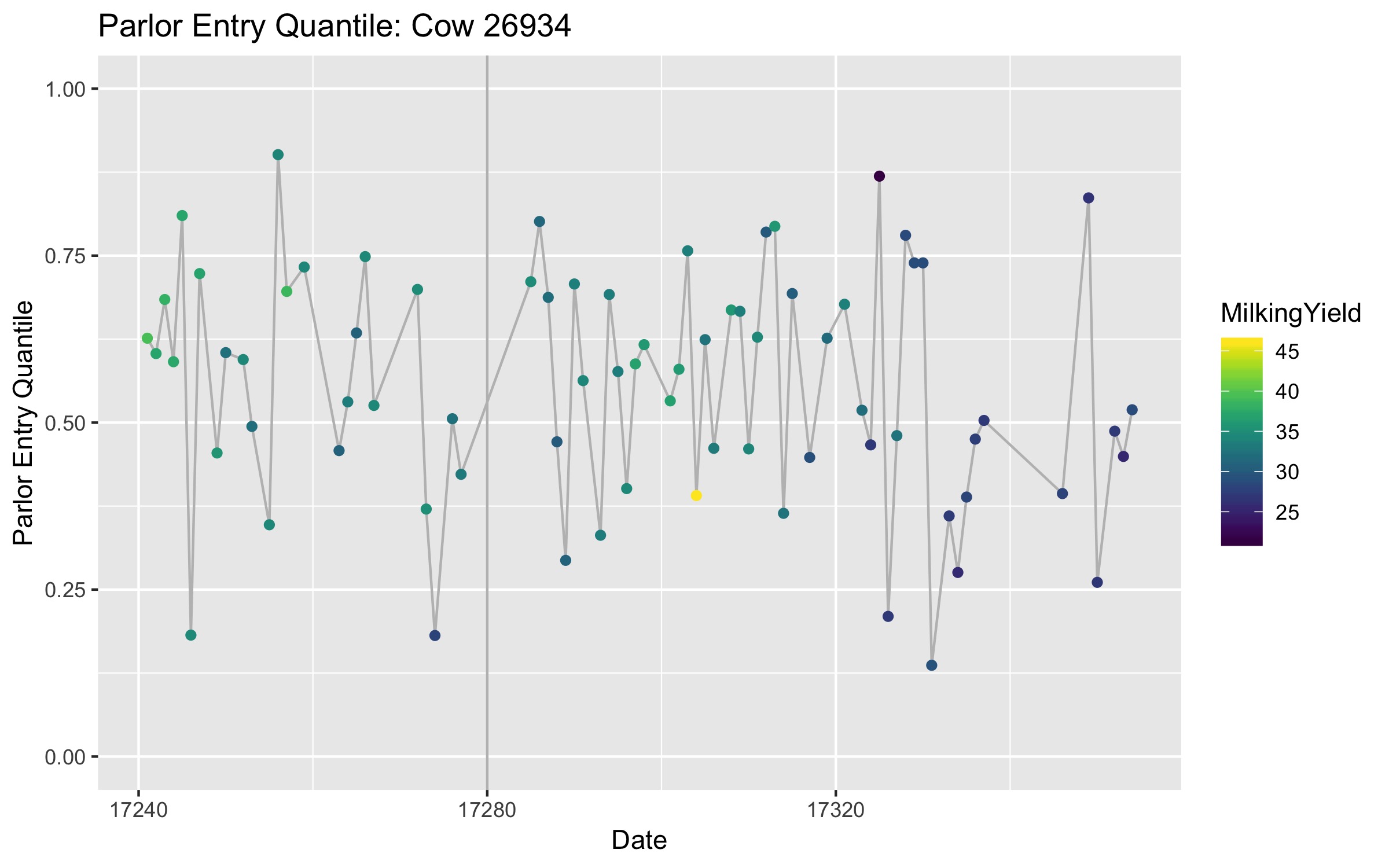

Supplement: Supplementary file 2 [file Data_Sheet_2.ZIP › Milking Yield/Cow_26934.jpg]

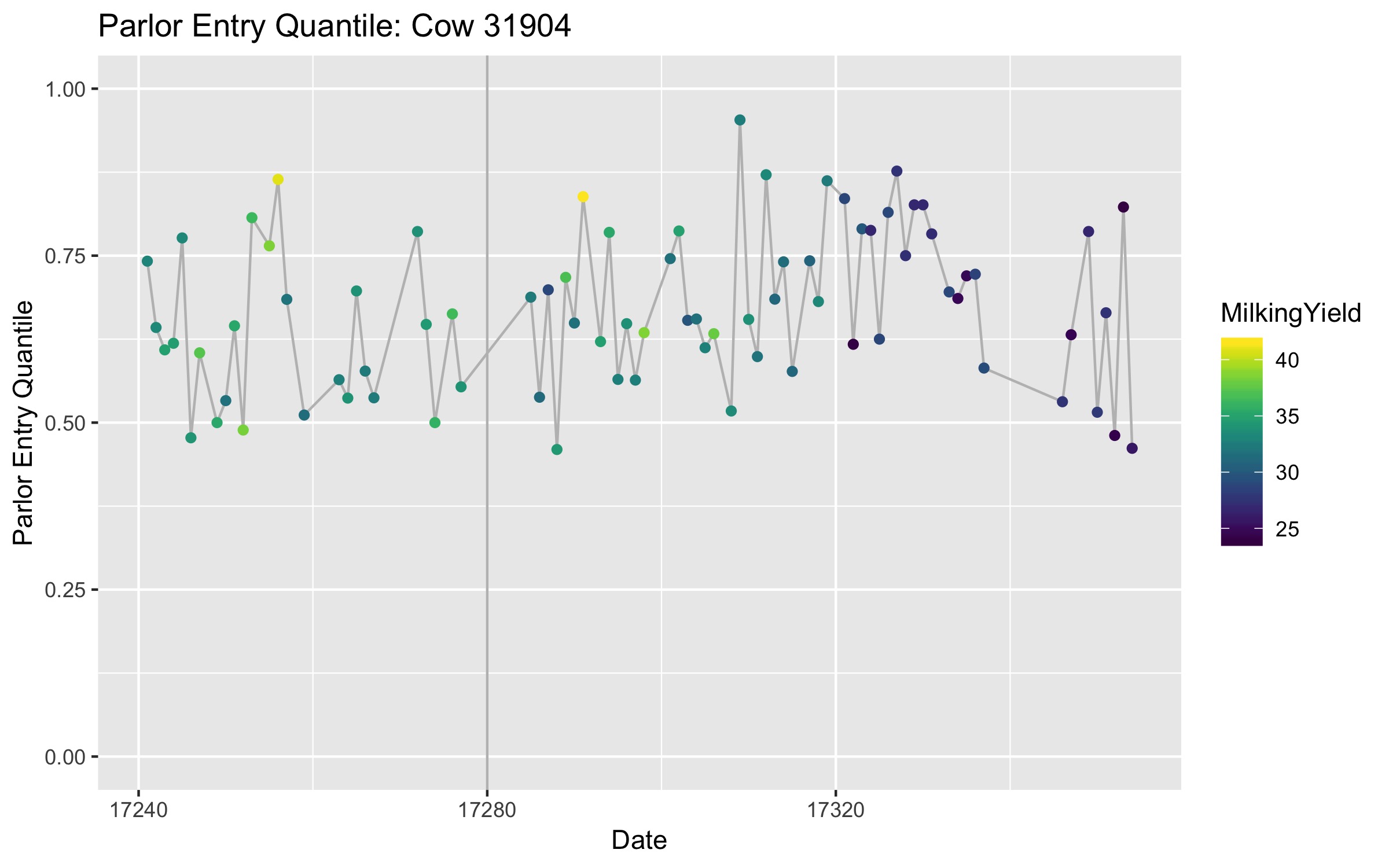

Supplement: Supplementary file 2 [file Data_Sheet_2.ZIP › Milking Yield/Cow_31904.jpg]

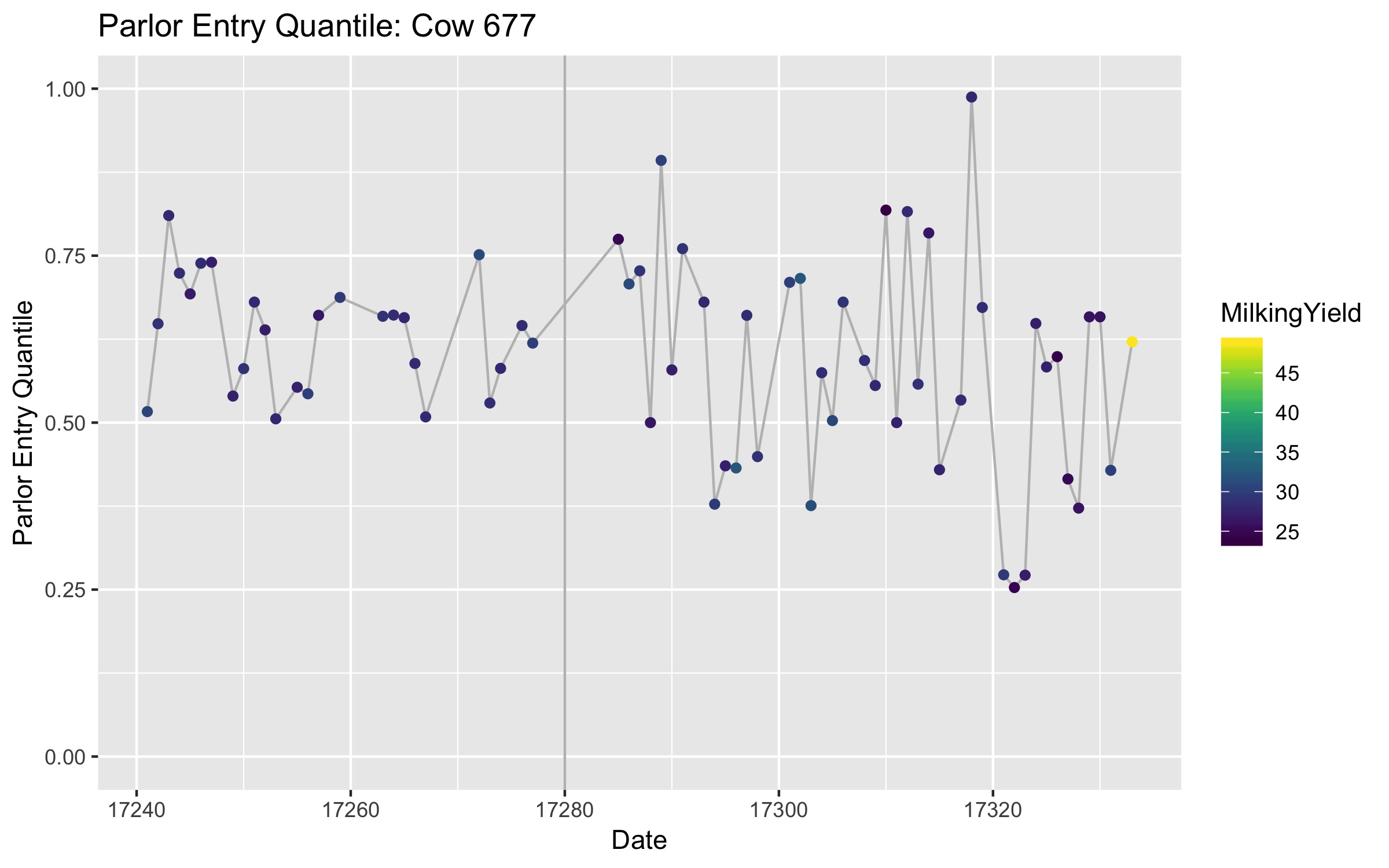

Supplement: Supplementary file 2 [file Data_Sheet_2.ZIP › Milking Yield/Cow_677.jpg]

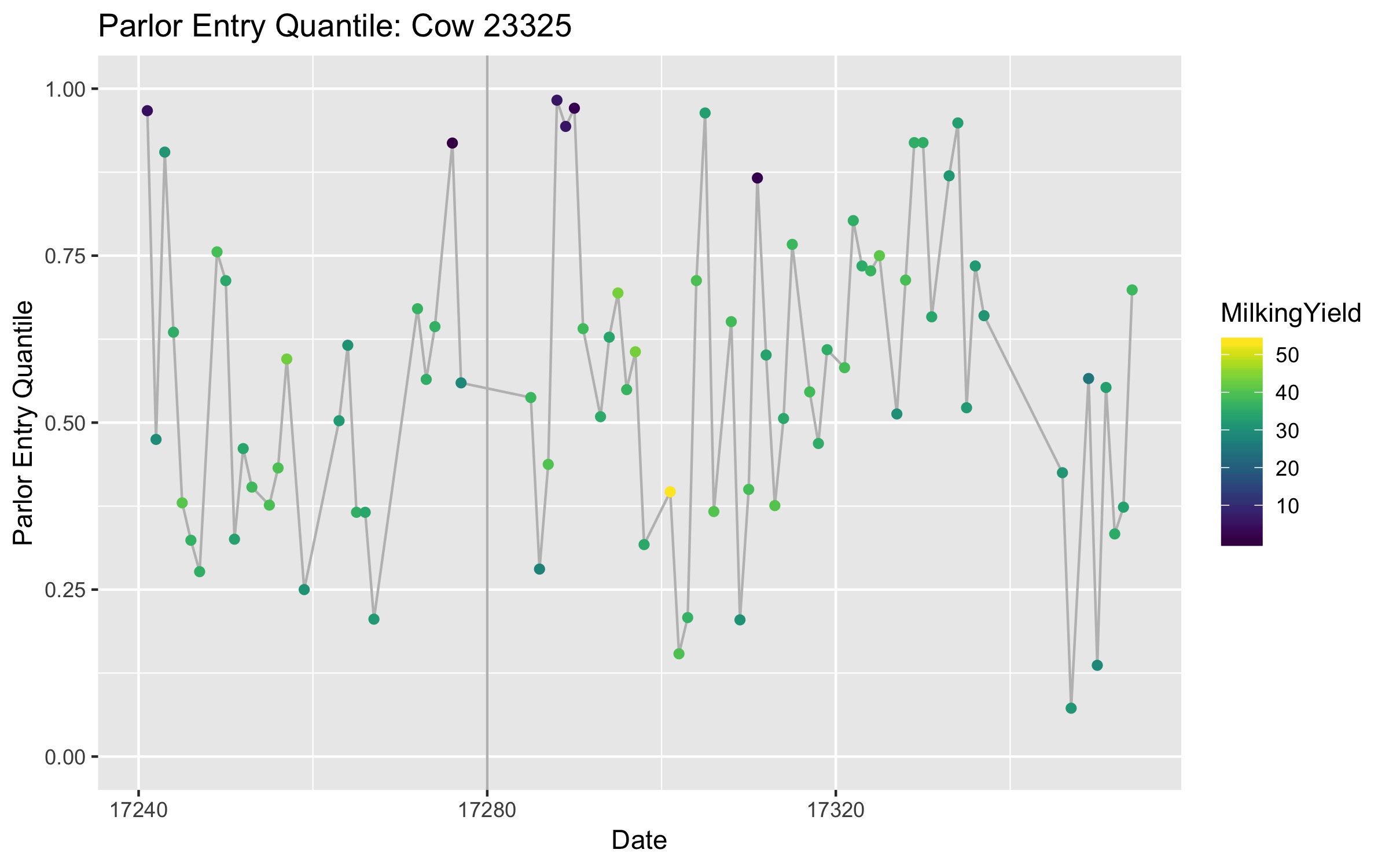

Supplement: Supplementary file 2 [file Data_Sheet_2.ZIP › Milking Yield/Cow_23325.jpg]

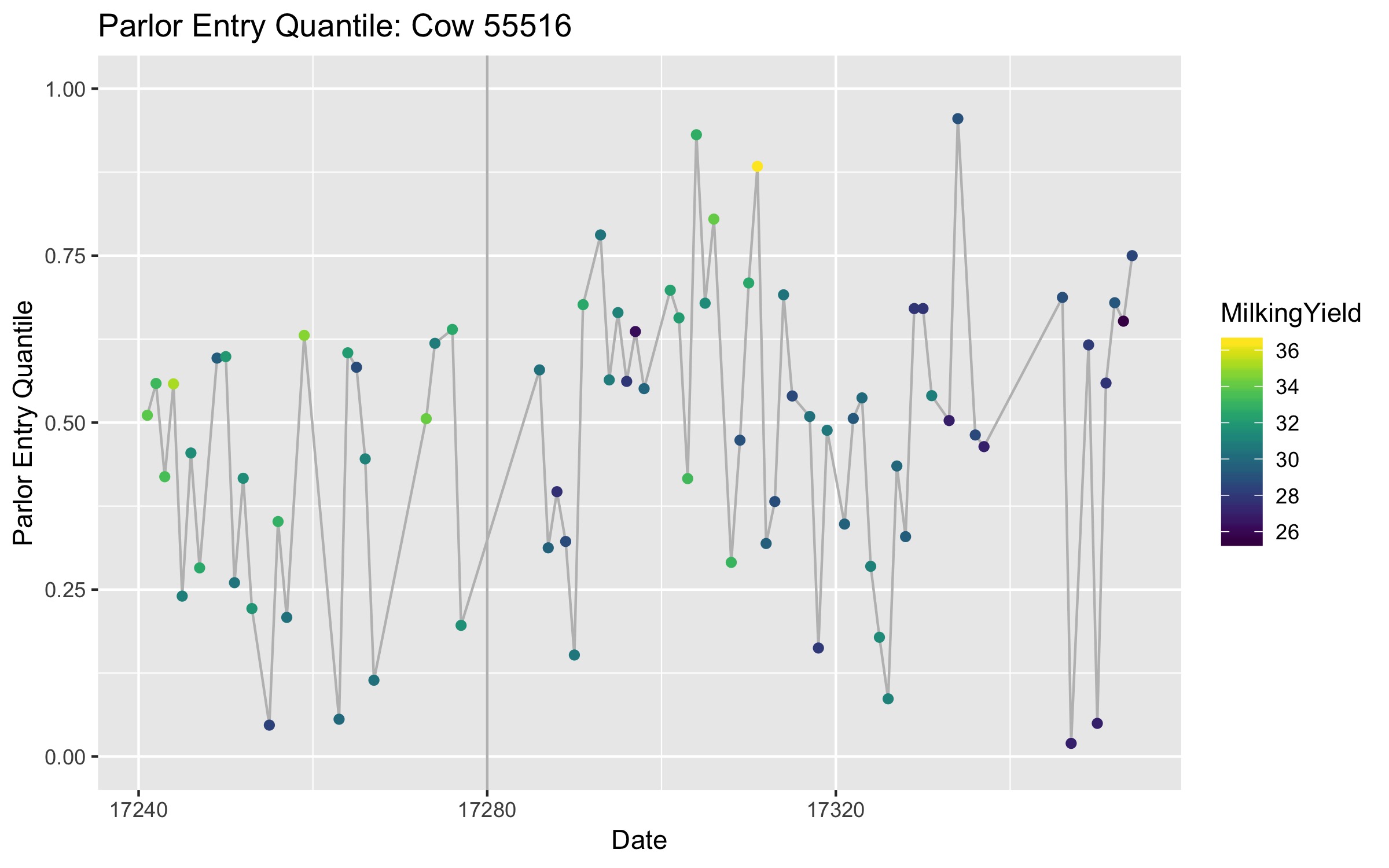

Supplement: Supplementary file 2 [file Data_Sheet_2.ZIP › Milking Yield/Cow_55516.jpg]

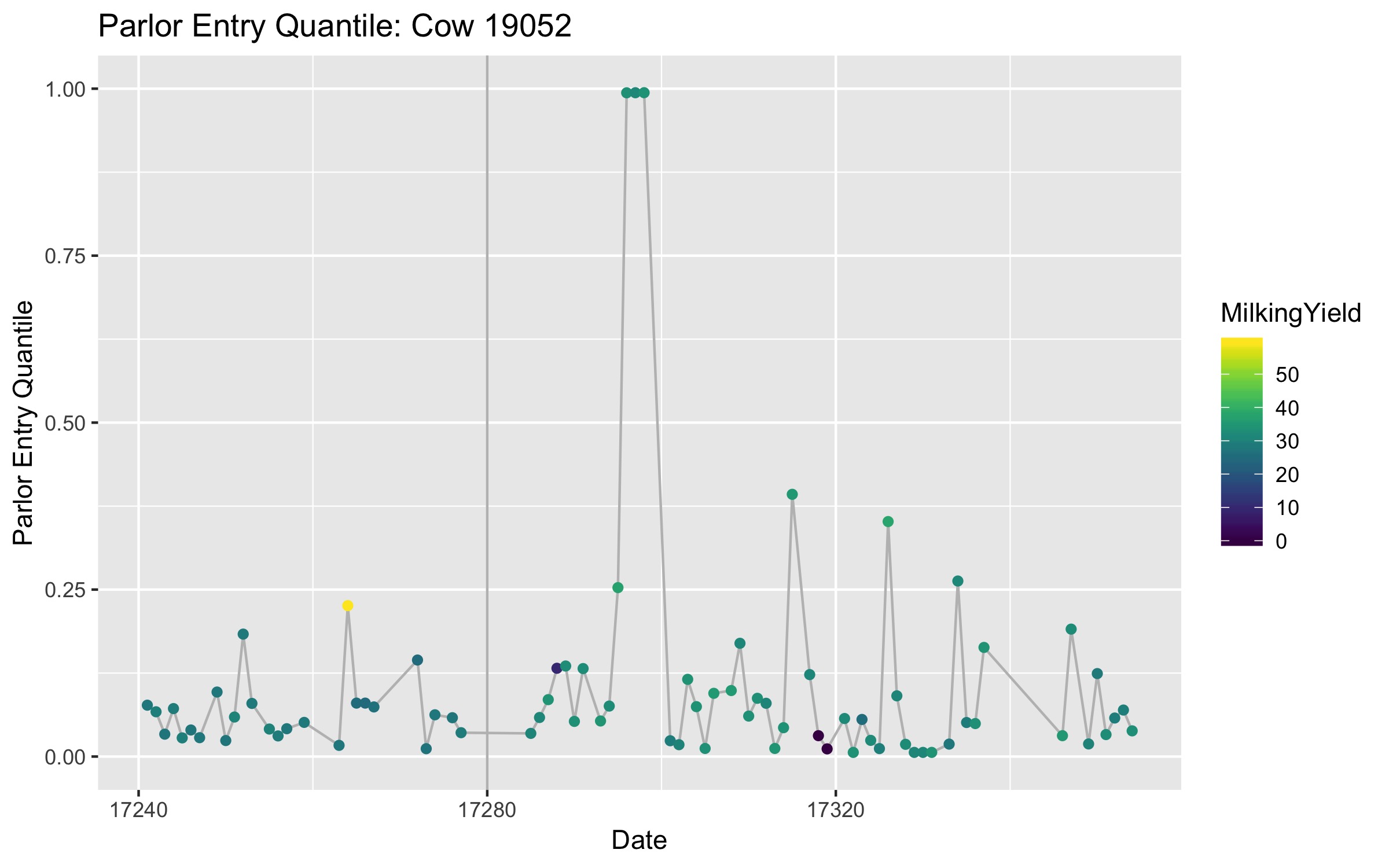

Supplement: Supplementary file 2 [file Data_Sheet_2.ZIP › Milking Yield/Cow_19052.jpg]

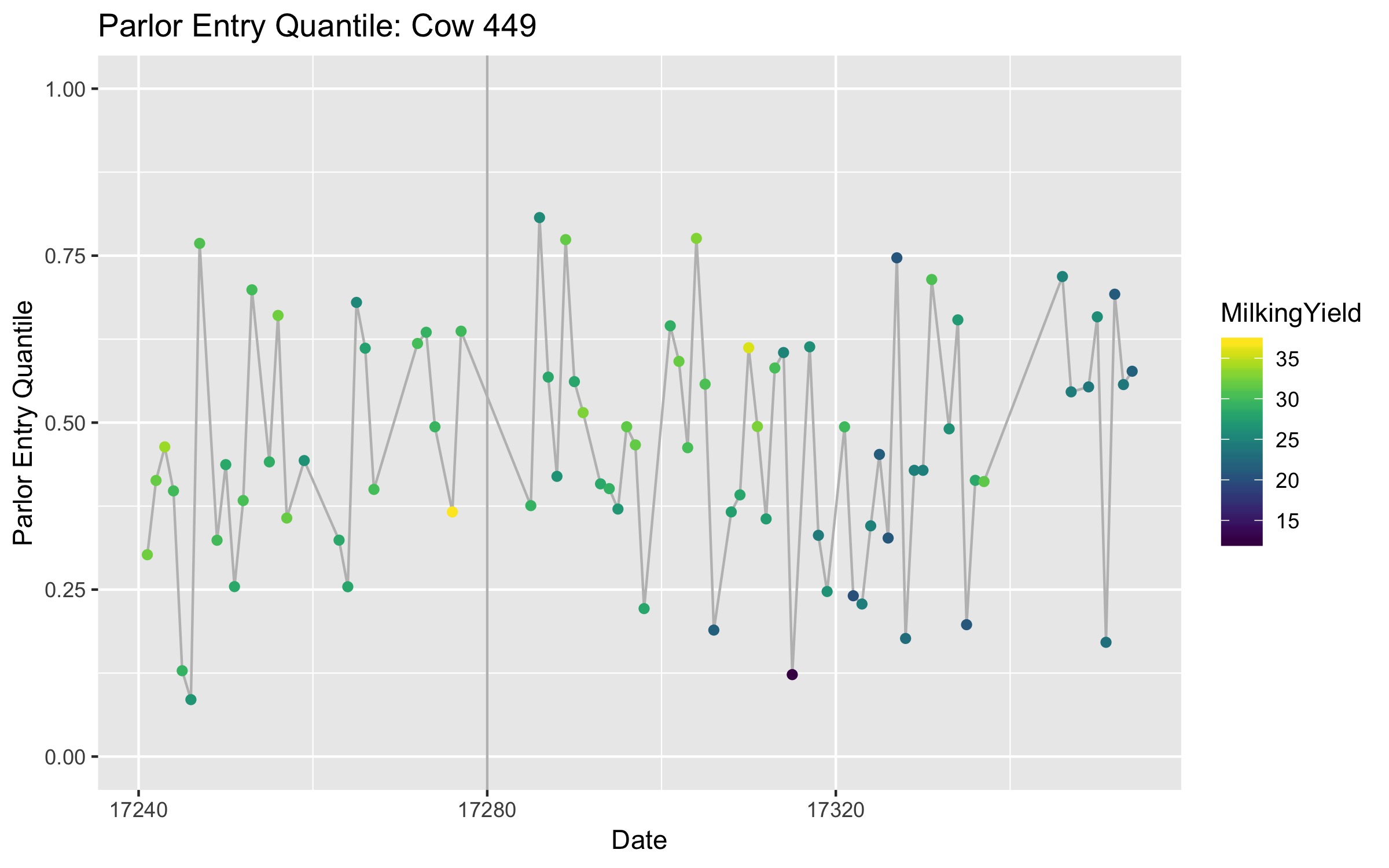

Supplement: Supplementary file 2 [file Data_Sheet_2.ZIP › Milking Yield/Cow_449.jpg]

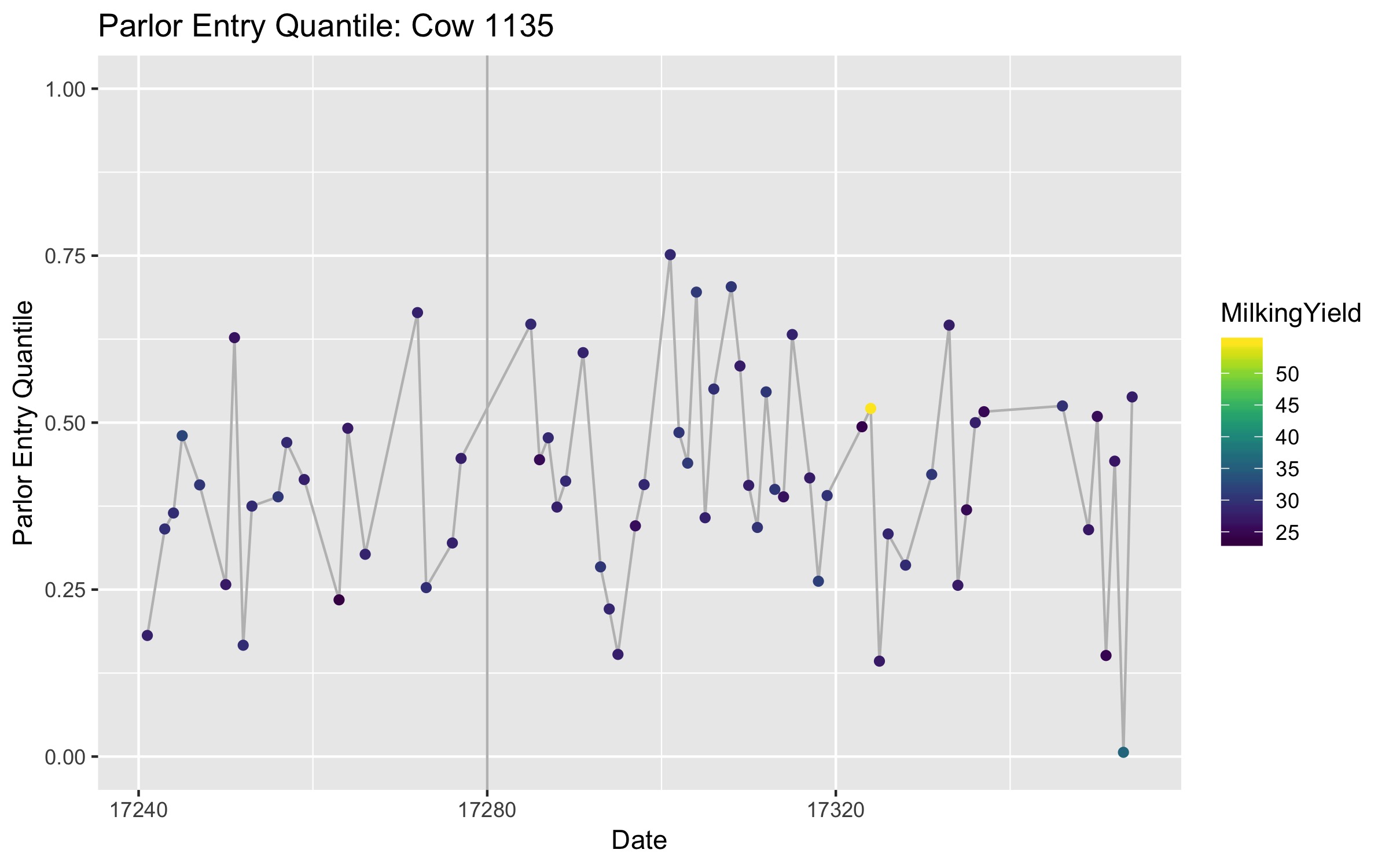

Supplement: Supplementary file 2 [file Data_Sheet_2.ZIP › Milking Yield/Cow_1135.jpg]

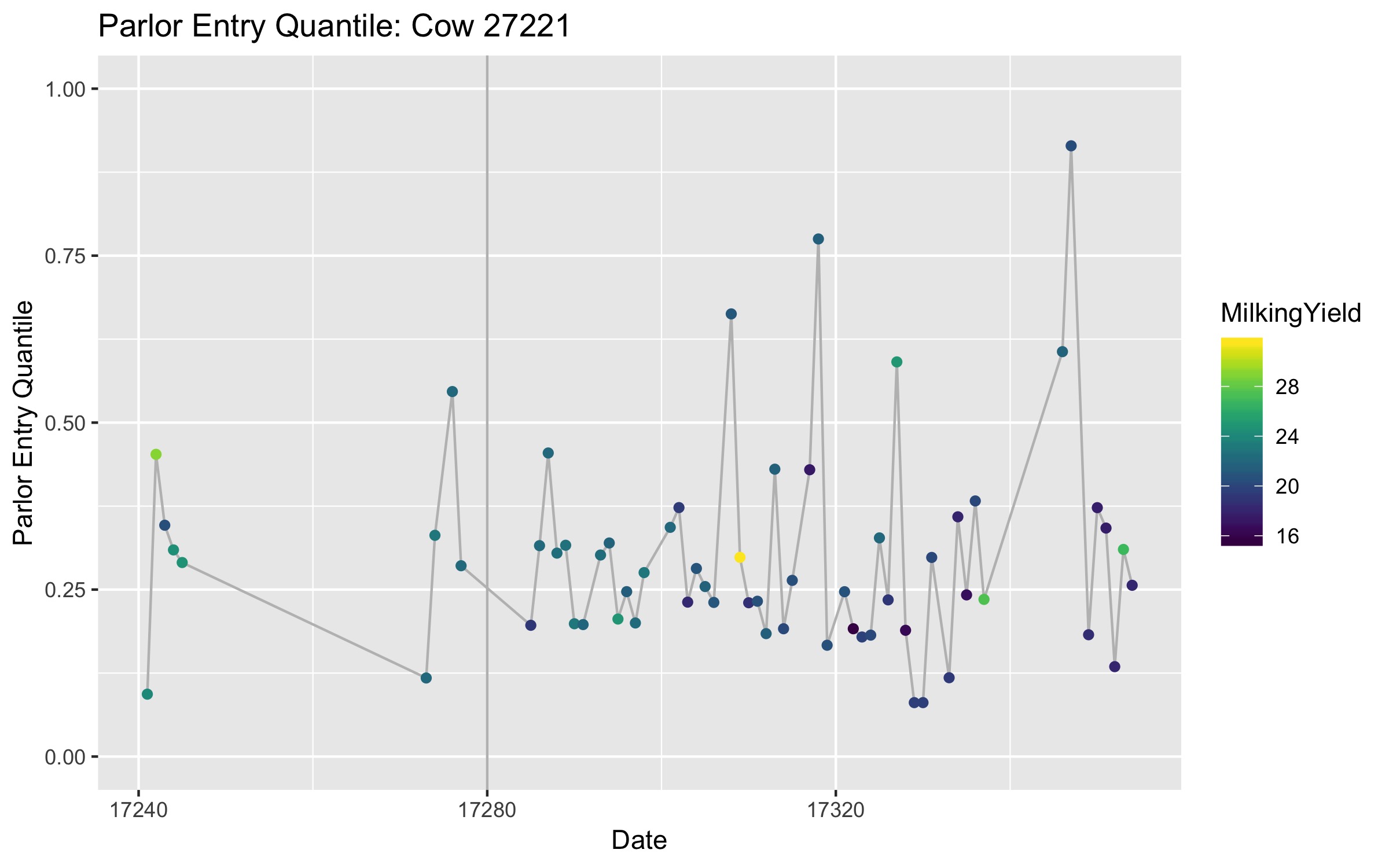

Supplement: Supplementary file 2 [file Data_Sheet_2.ZIP › Milking Yield/Cow_27221.jpg]

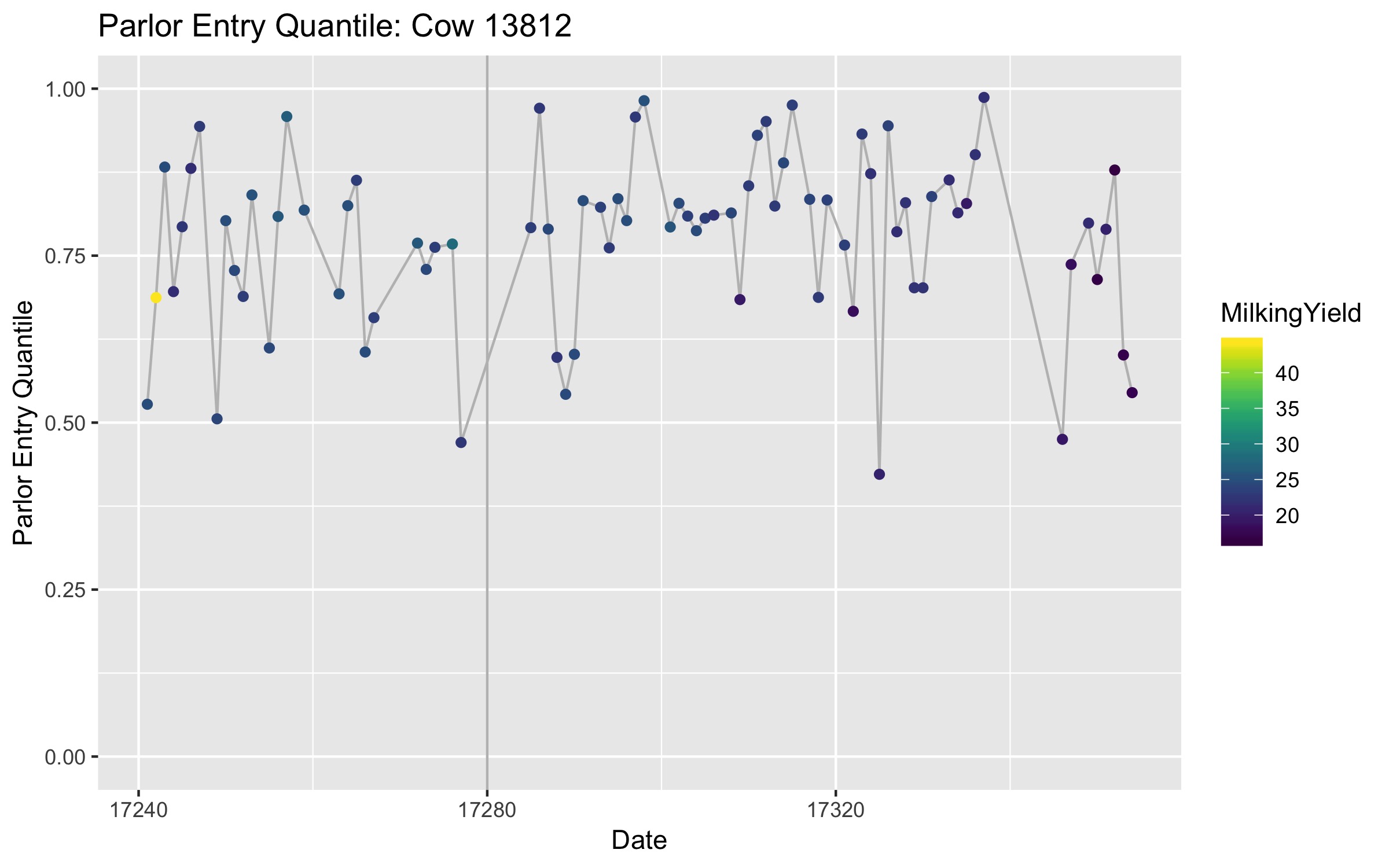

Supplement: Supplementary file 2 [file Data_Sheet_2.ZIP › Milking Yield/Cow_13812.jpg]

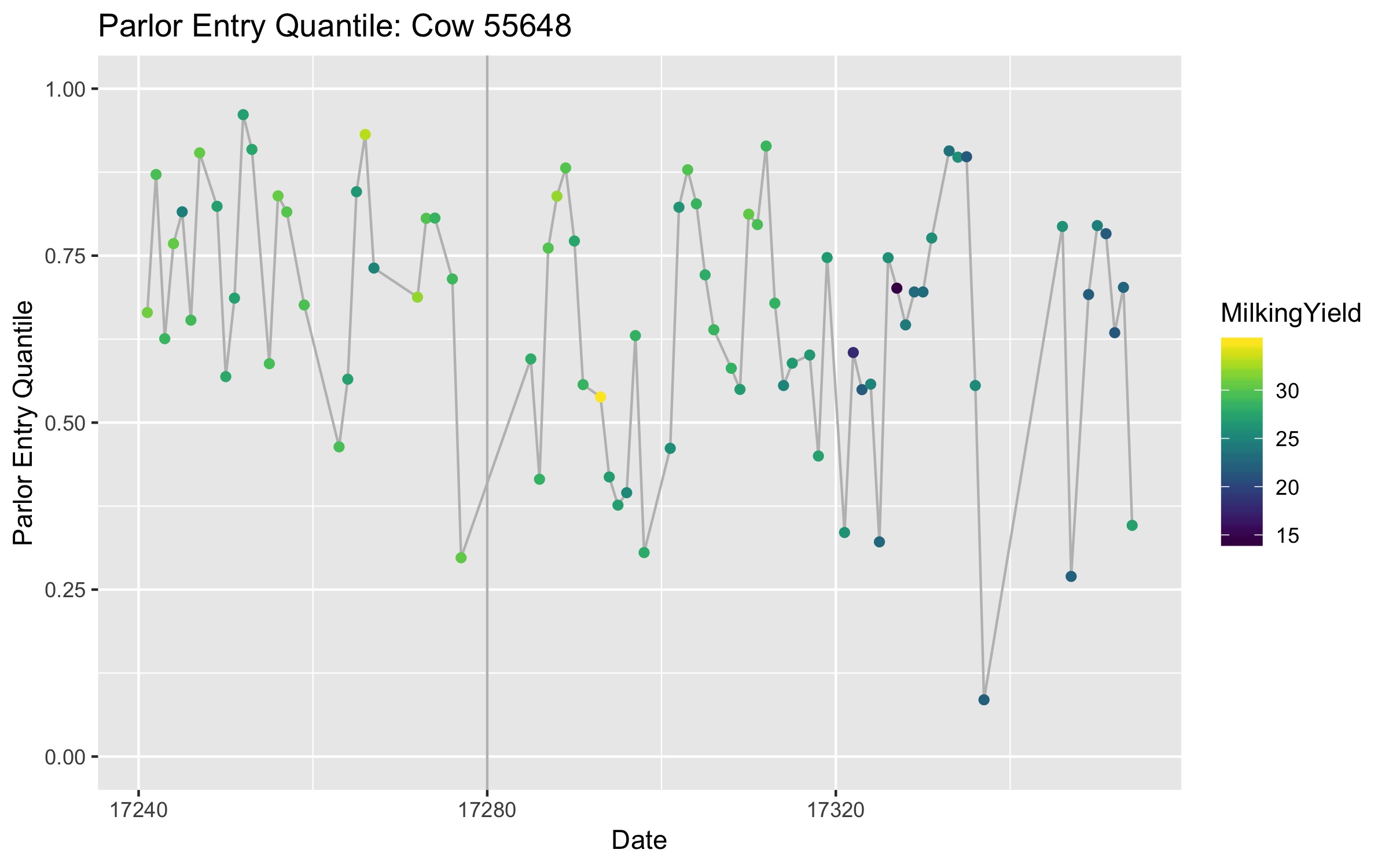

Supplement: Supplementary file 2 [file Data_Sheet_2.ZIP › Milking Yield/Cow_55648.jpg]

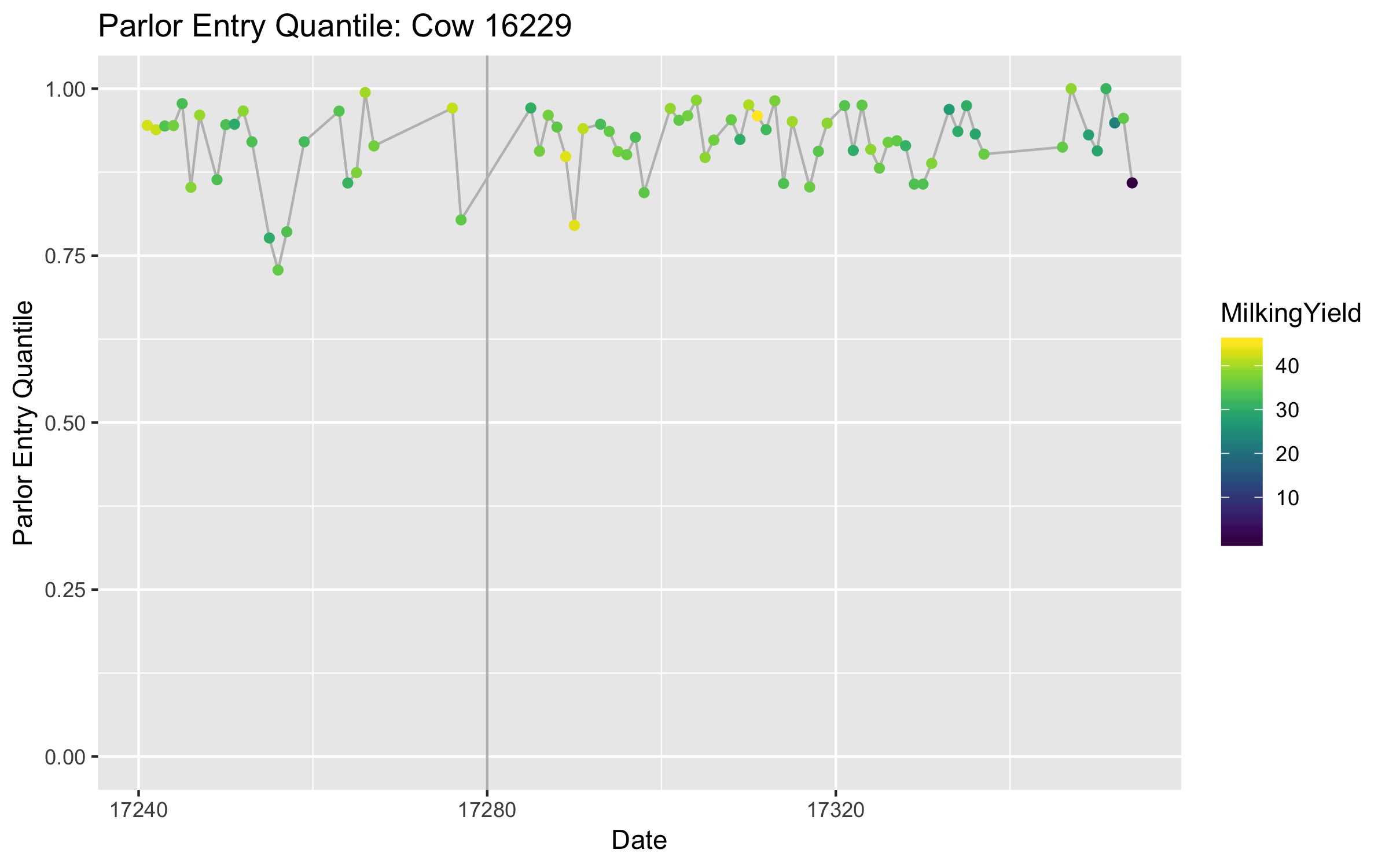

Supplement: Supplementary file 2 [file Data_Sheet_2.ZIP › Milking Yield/Cow_16229.jpg]

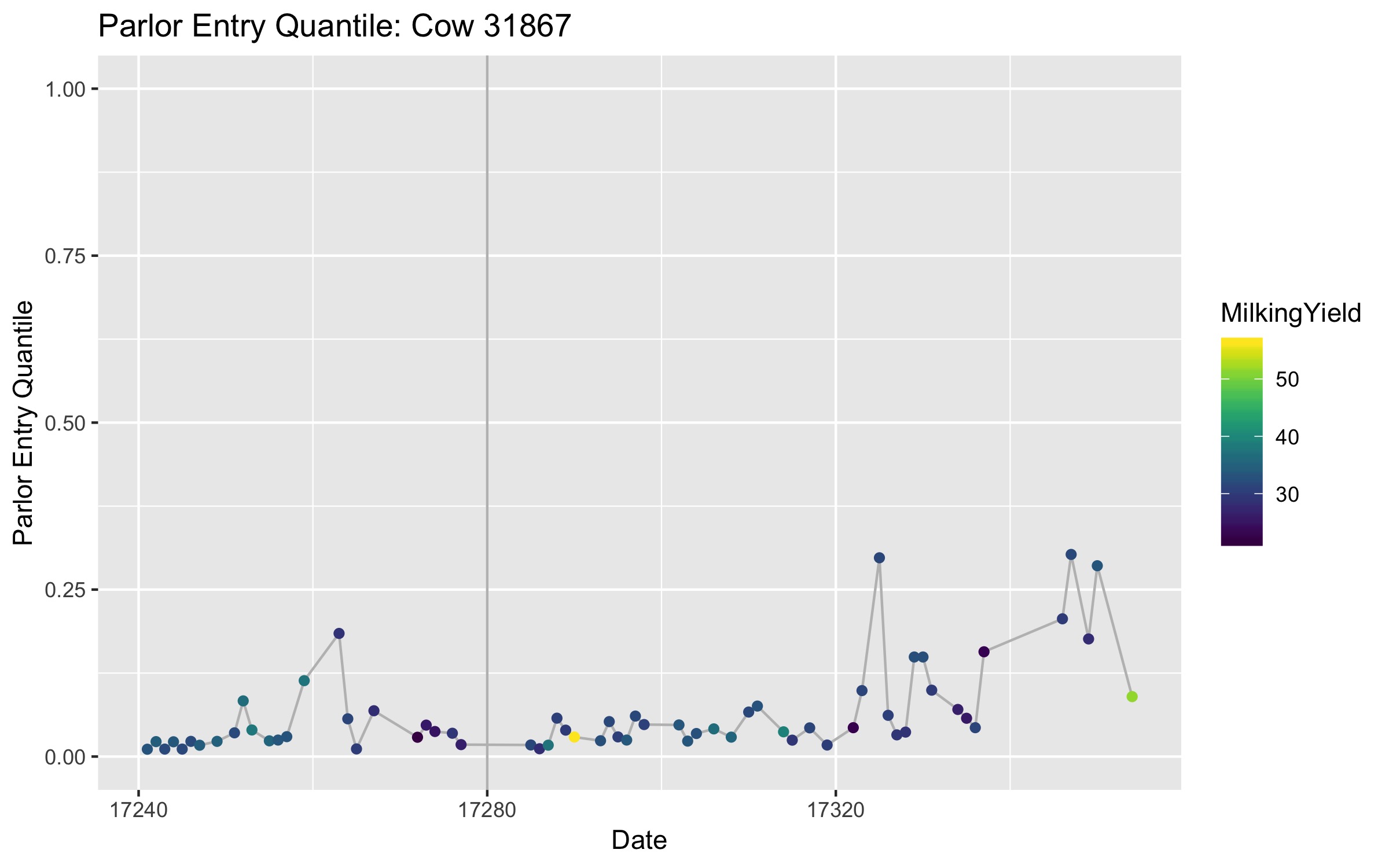

Supplement: Supplementary file 2 [file Data_Sheet_2.ZIP › Milking Yield/Cow_31867.jpg]

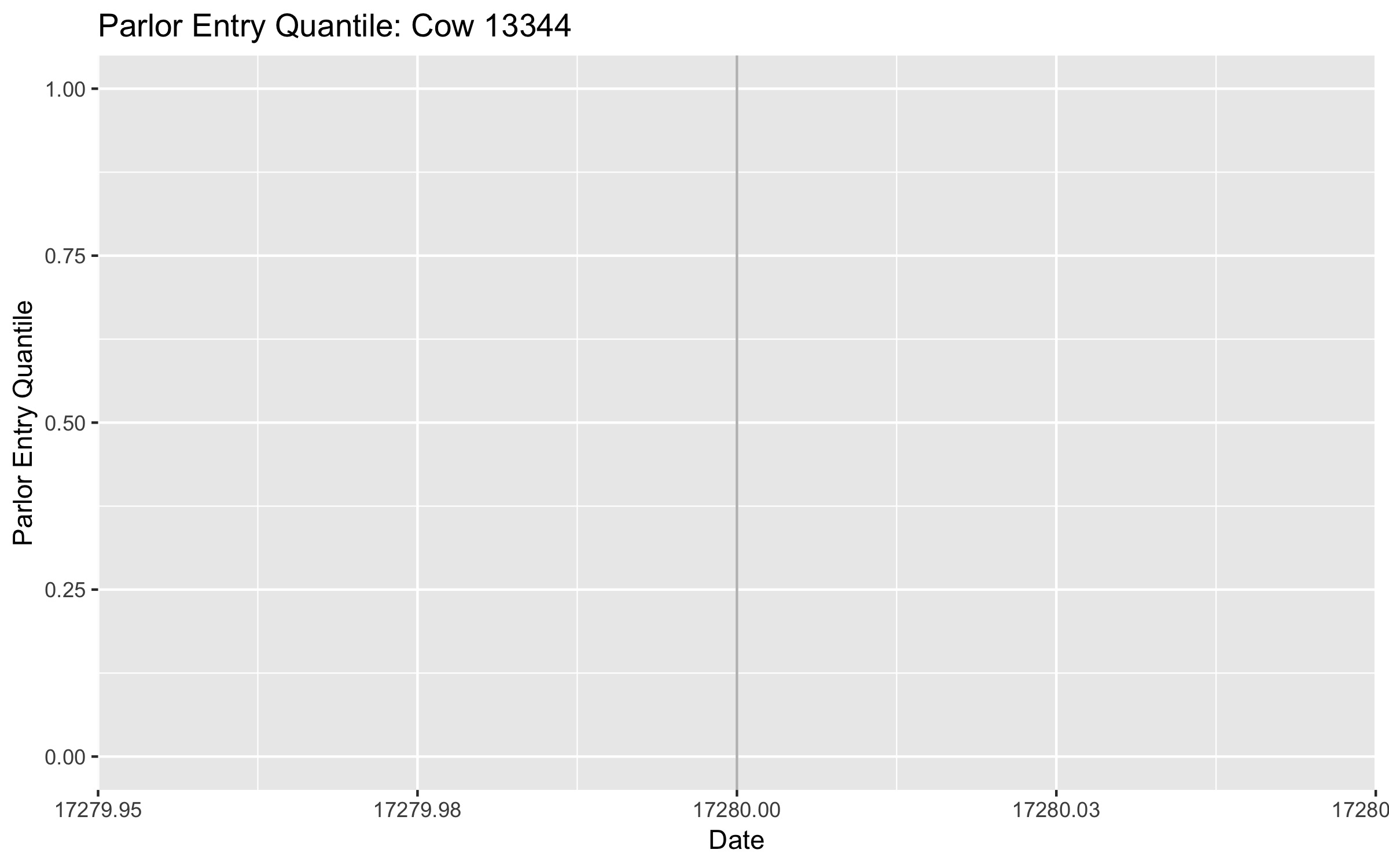

Supplement: Supplementary file 2 [file Data_Sheet_2.ZIP › Milking Yield/Cow_13344.jpg]

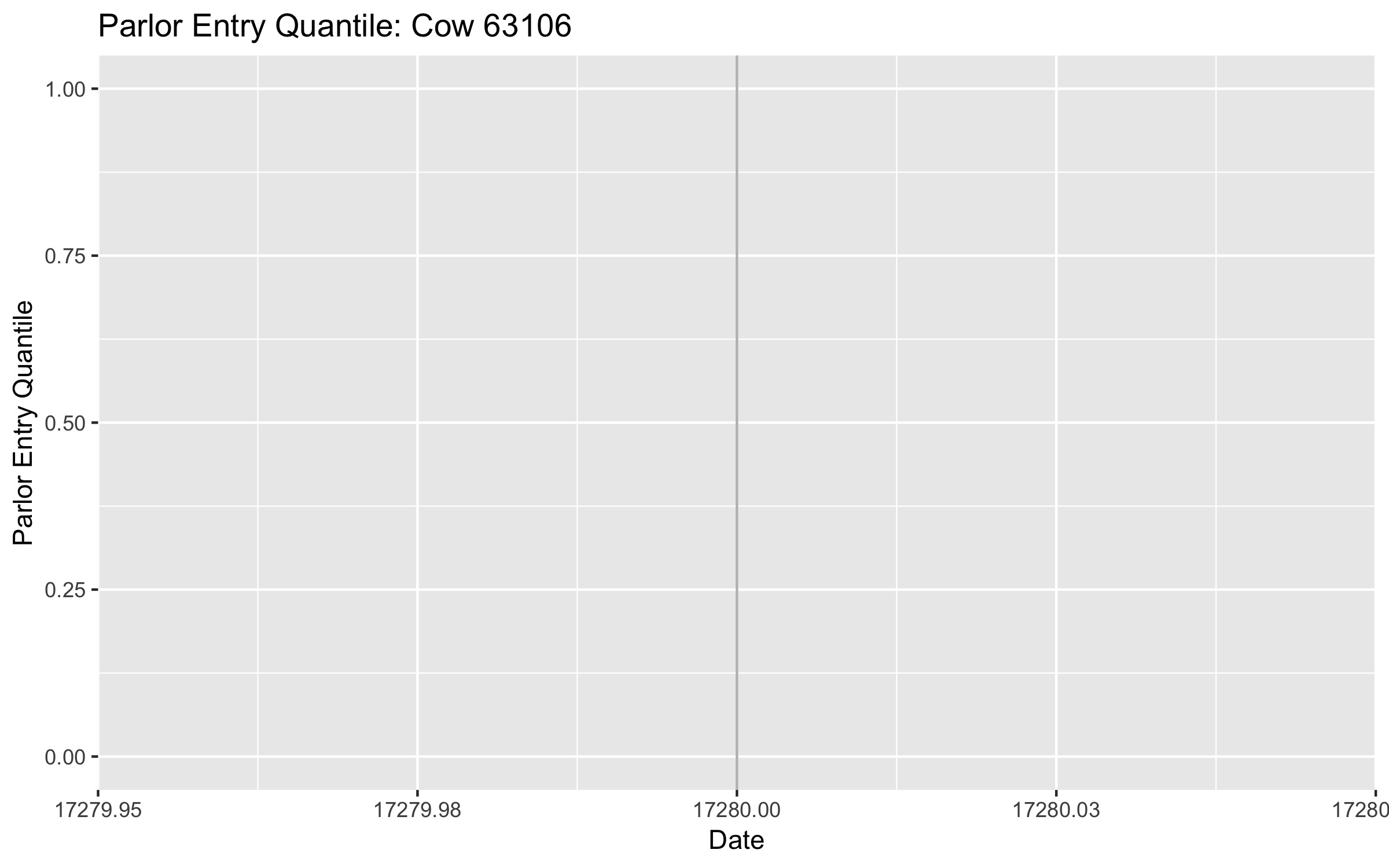

Supplement: Supplementary file 2 [file Data_Sheet_2.ZIP › Milking Yield/Cow_63106.jpg]

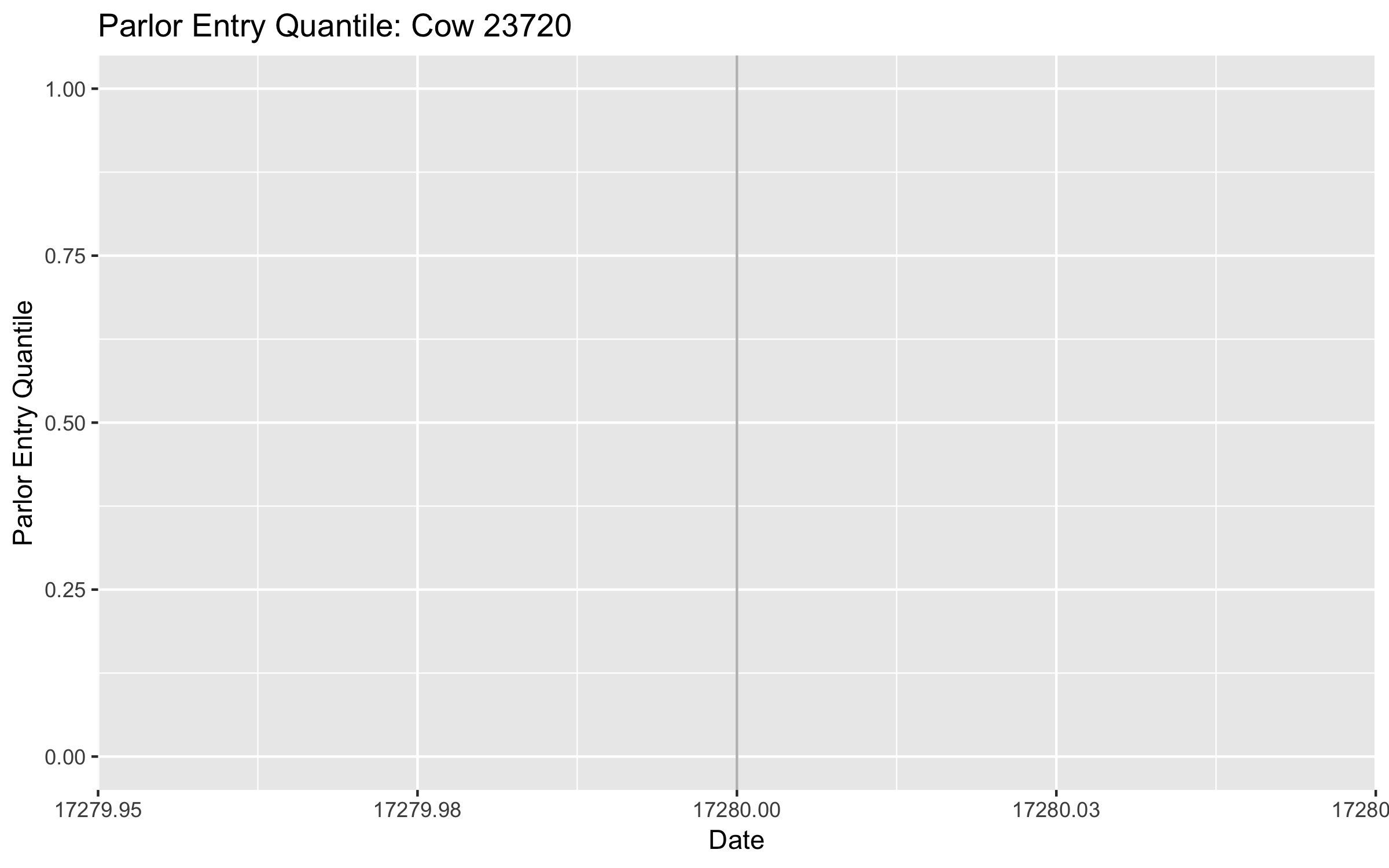

Supplement: Supplementary file 2 [file Data_Sheet_2.ZIP › Milking Yield/Cow_23720.jpg]

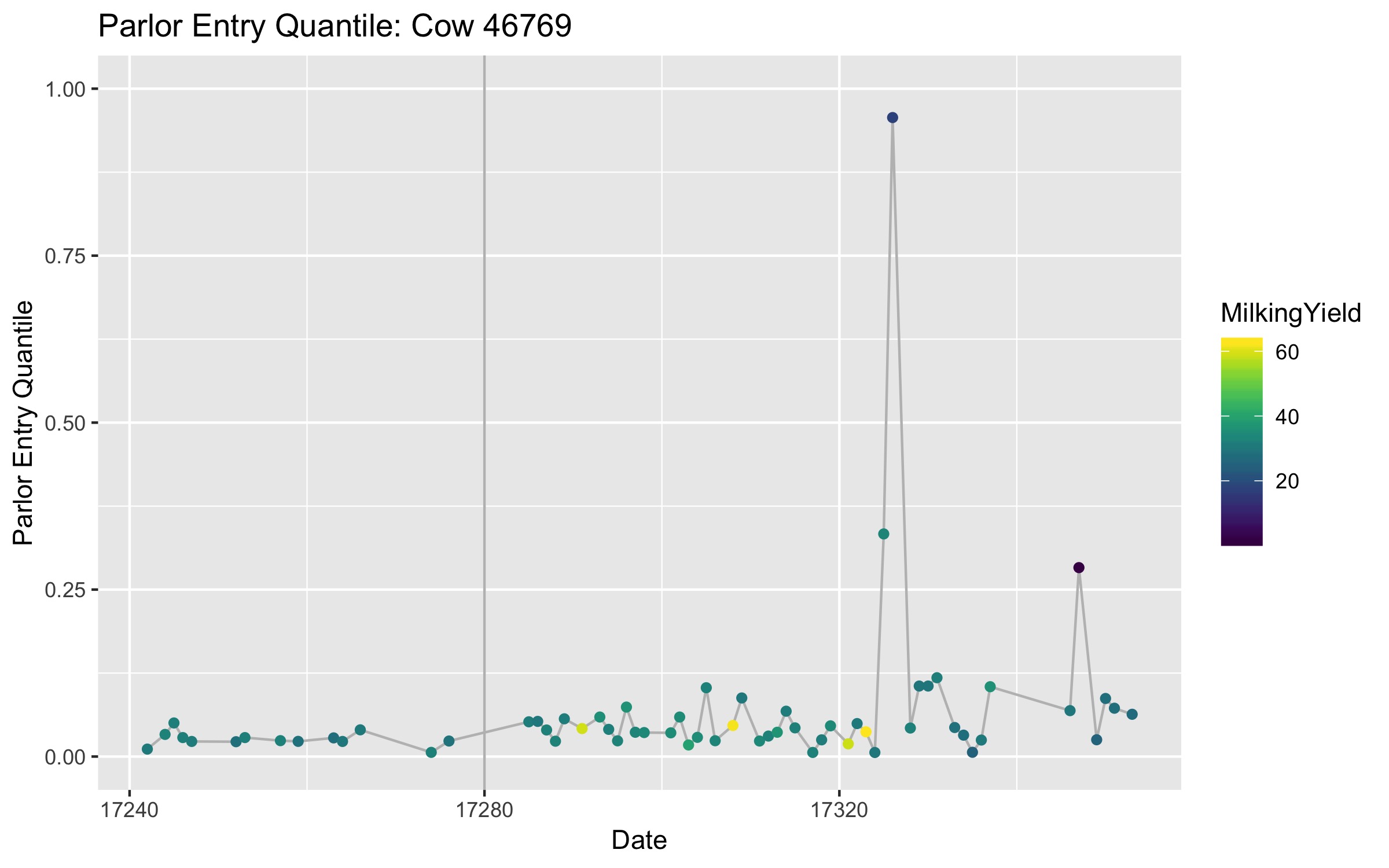

Supplement: Supplementary file 2 [file Data_Sheet_2.ZIP › Milking Yield/Cow_46769.jpg]

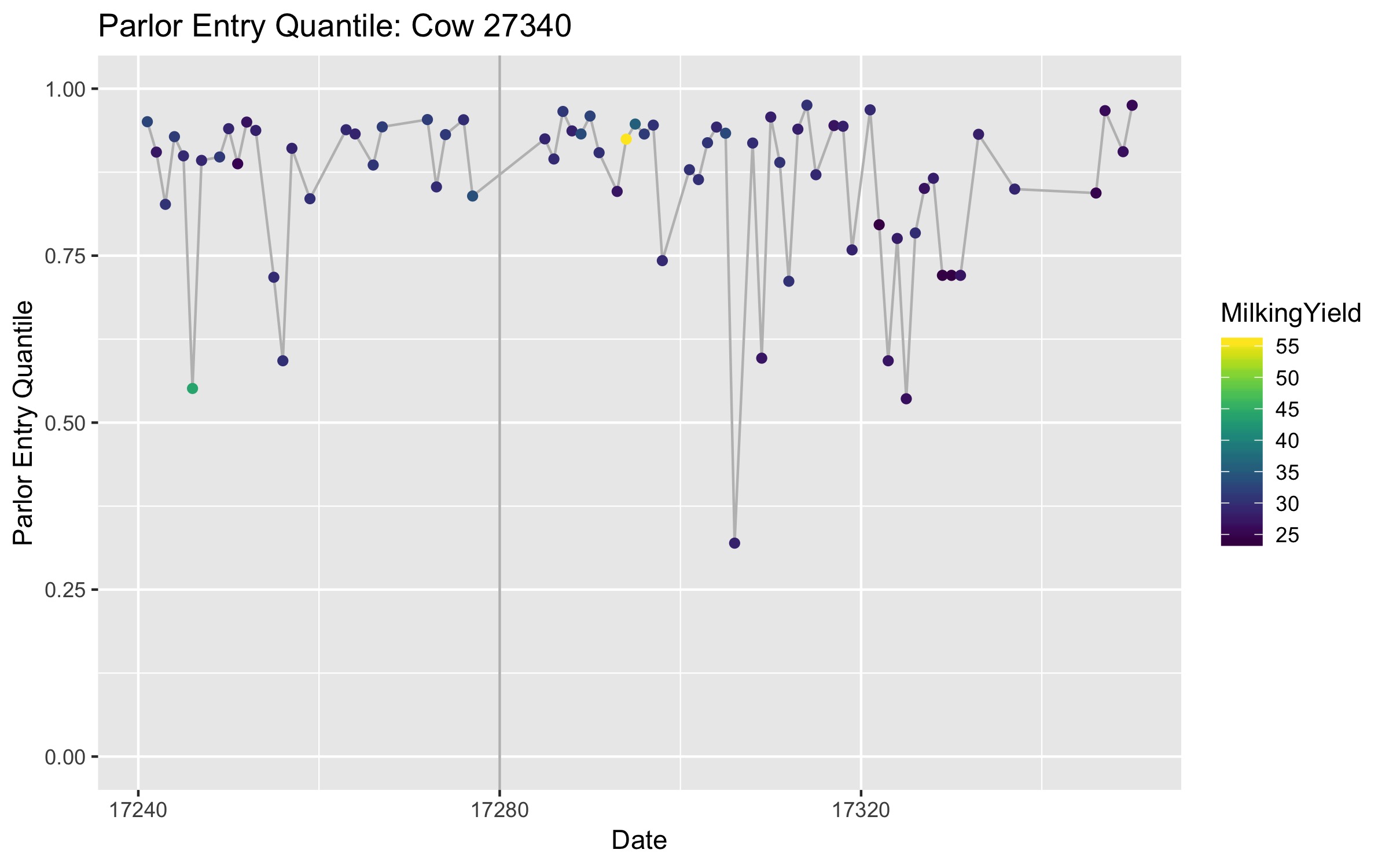

Supplement: Supplementary file 2 [file Data_Sheet_2.ZIP › Milking Yield/Cow_27340.jpg]

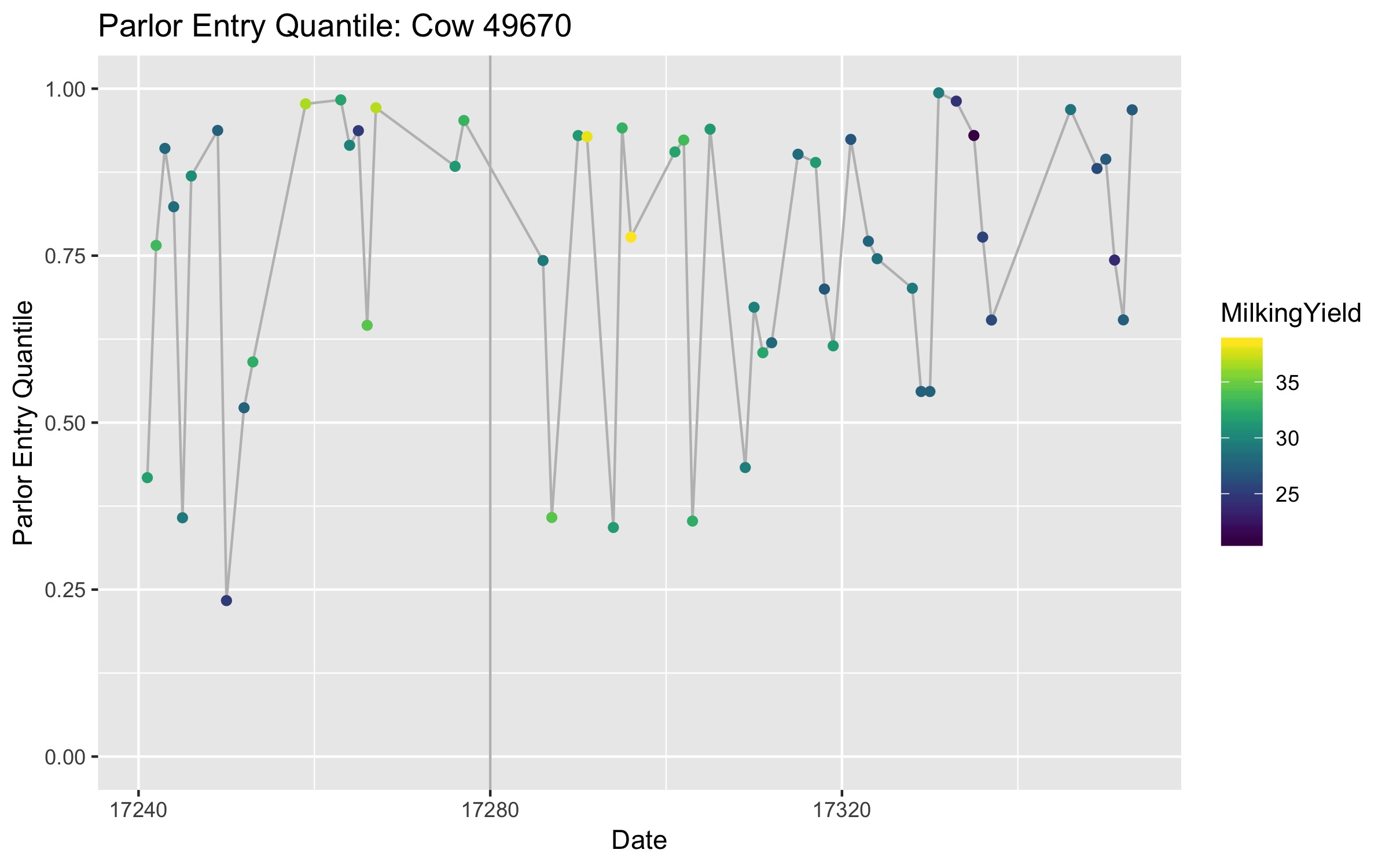

Supplement: Supplementary file 2 [file Data_Sheet_2.ZIP › Milking Yield/Cow_49670.jpg]

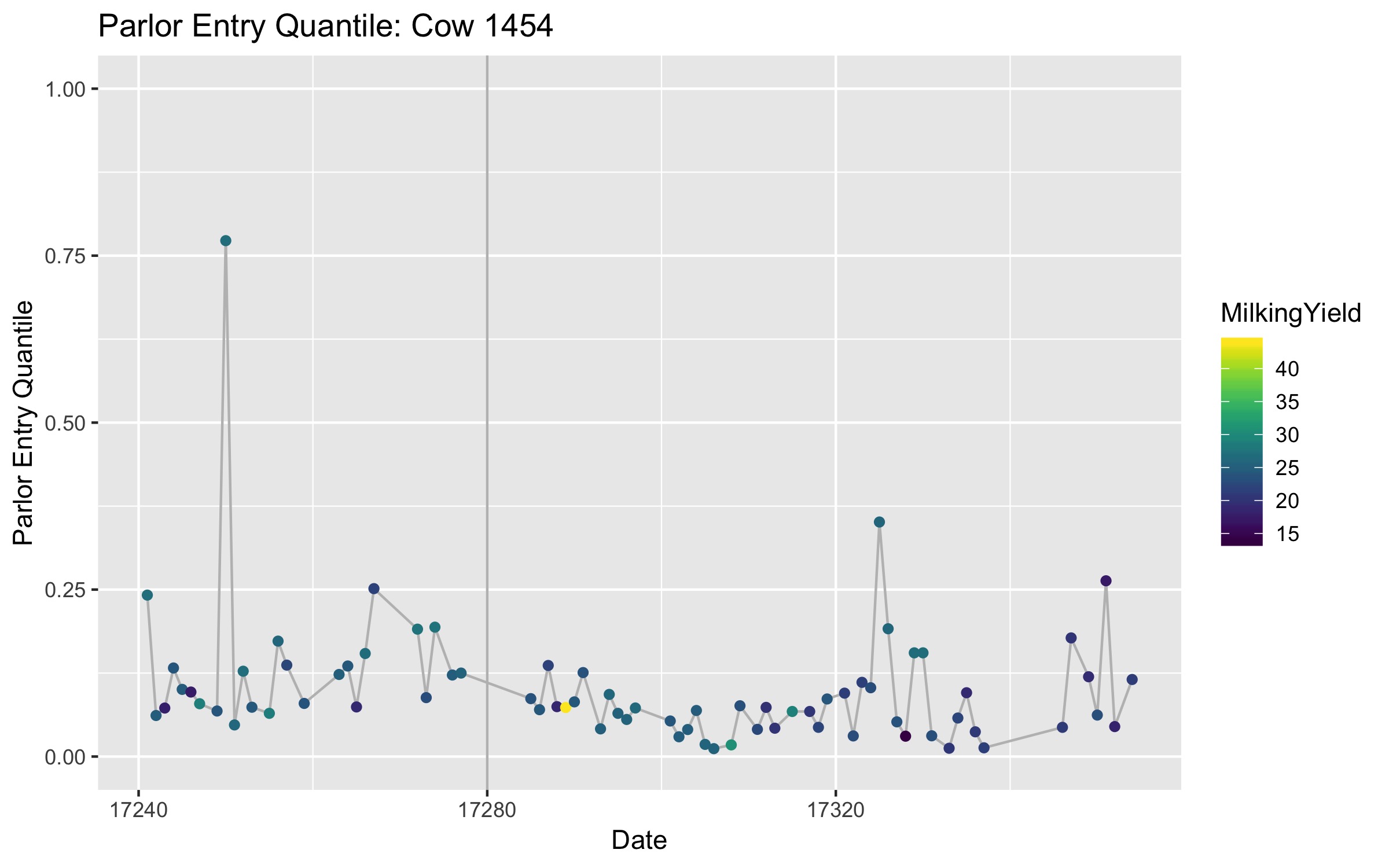

Supplement: Supplementary file 2 [file Data_Sheet_2.ZIP › Milking Yield/Cow_1454.jpg]

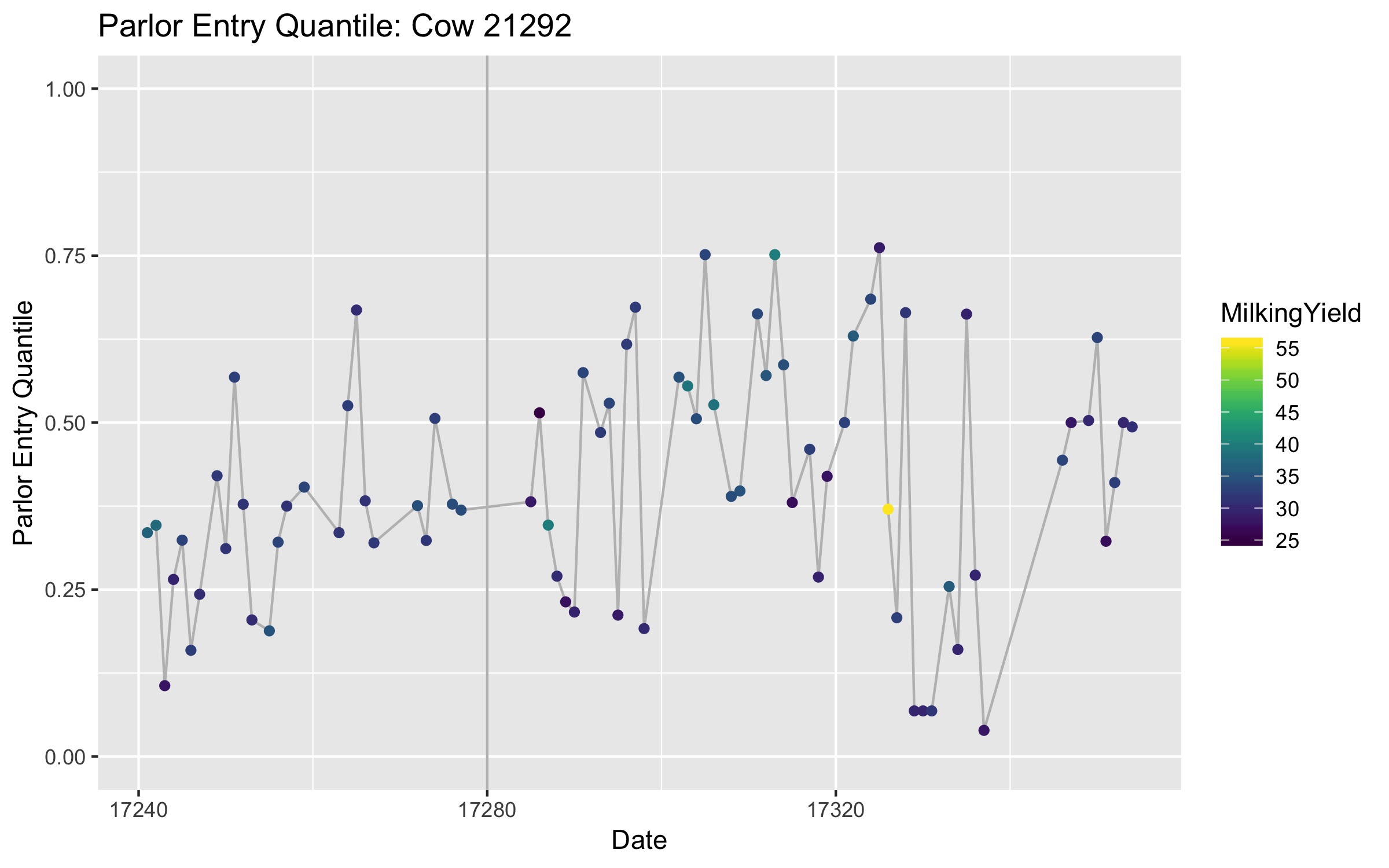

Supplement: Supplementary file 2 [file Data_Sheet_2.ZIP › Milking Yield/Cow_21292.jpg]

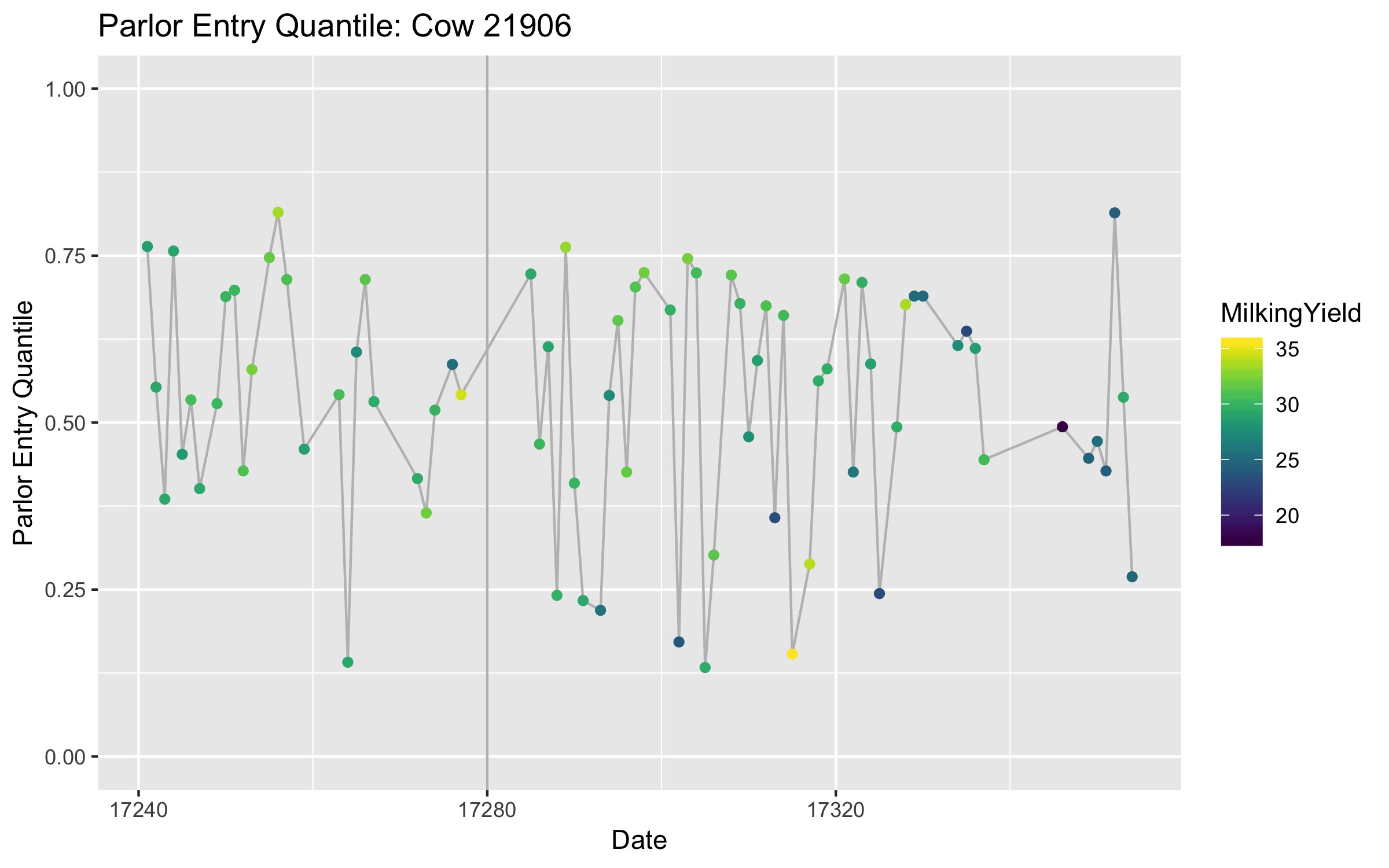

Supplement: Supplementary file 2 [file Data_Sheet_2.ZIP › Milking Yield/Cow_21906.jpg]

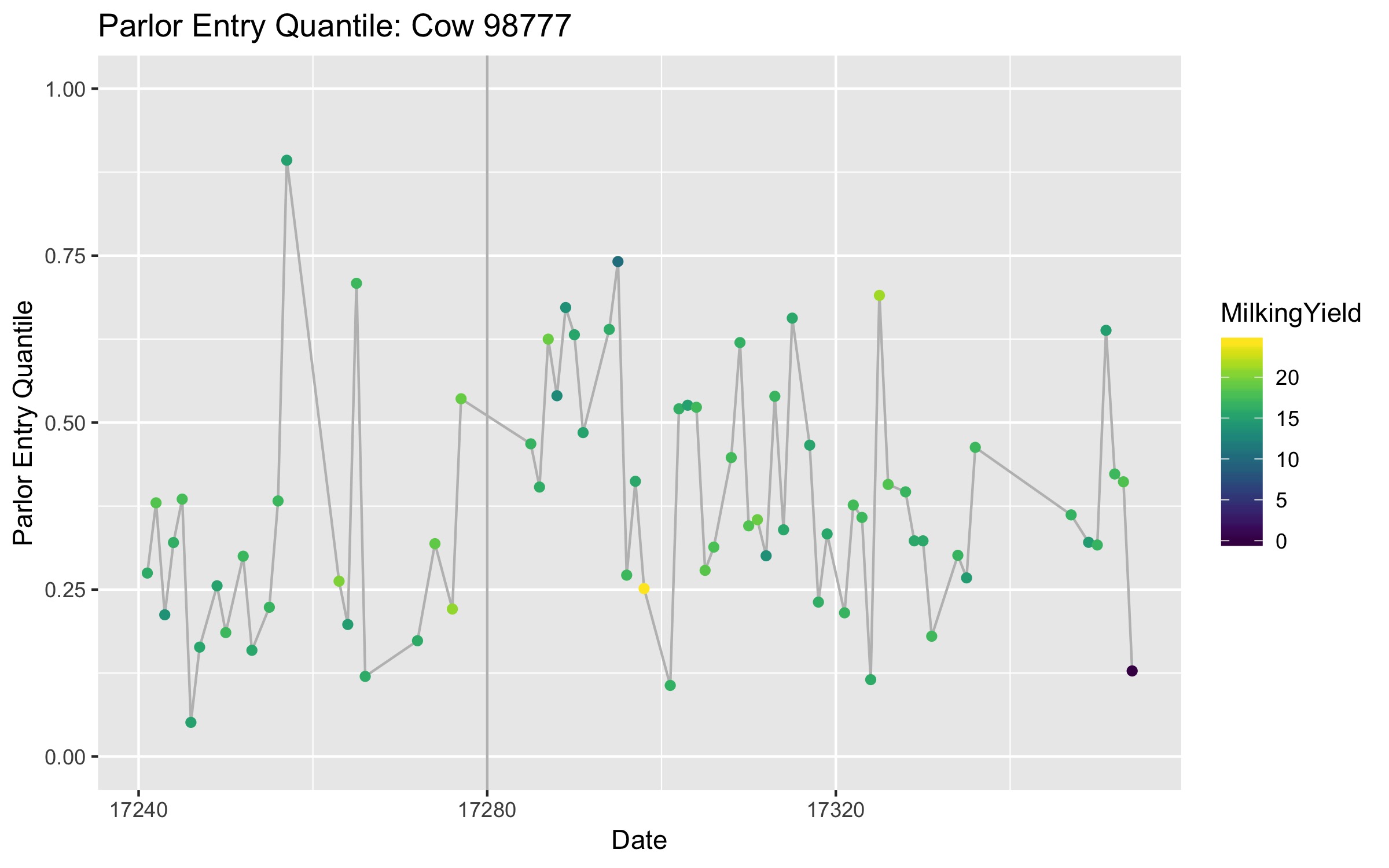

Supplement: Supplementary file 2 [file Data_Sheet_2.ZIP › Milking Yield/Cow_98777.jpg]

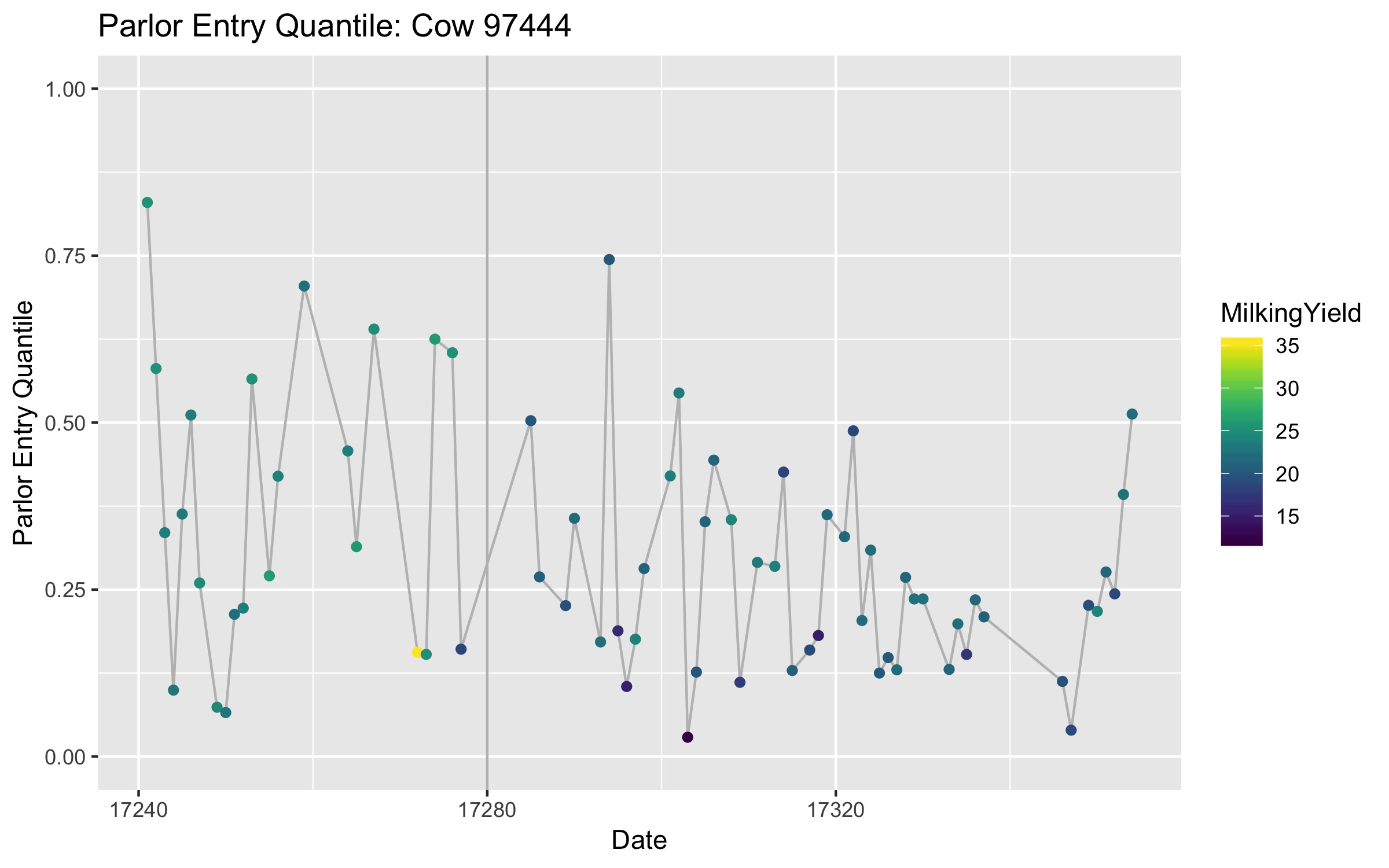

Supplement: Supplementary file 2 [file Data_Sheet_2.ZIP › Milking Yield/Cow_97444.jpg]

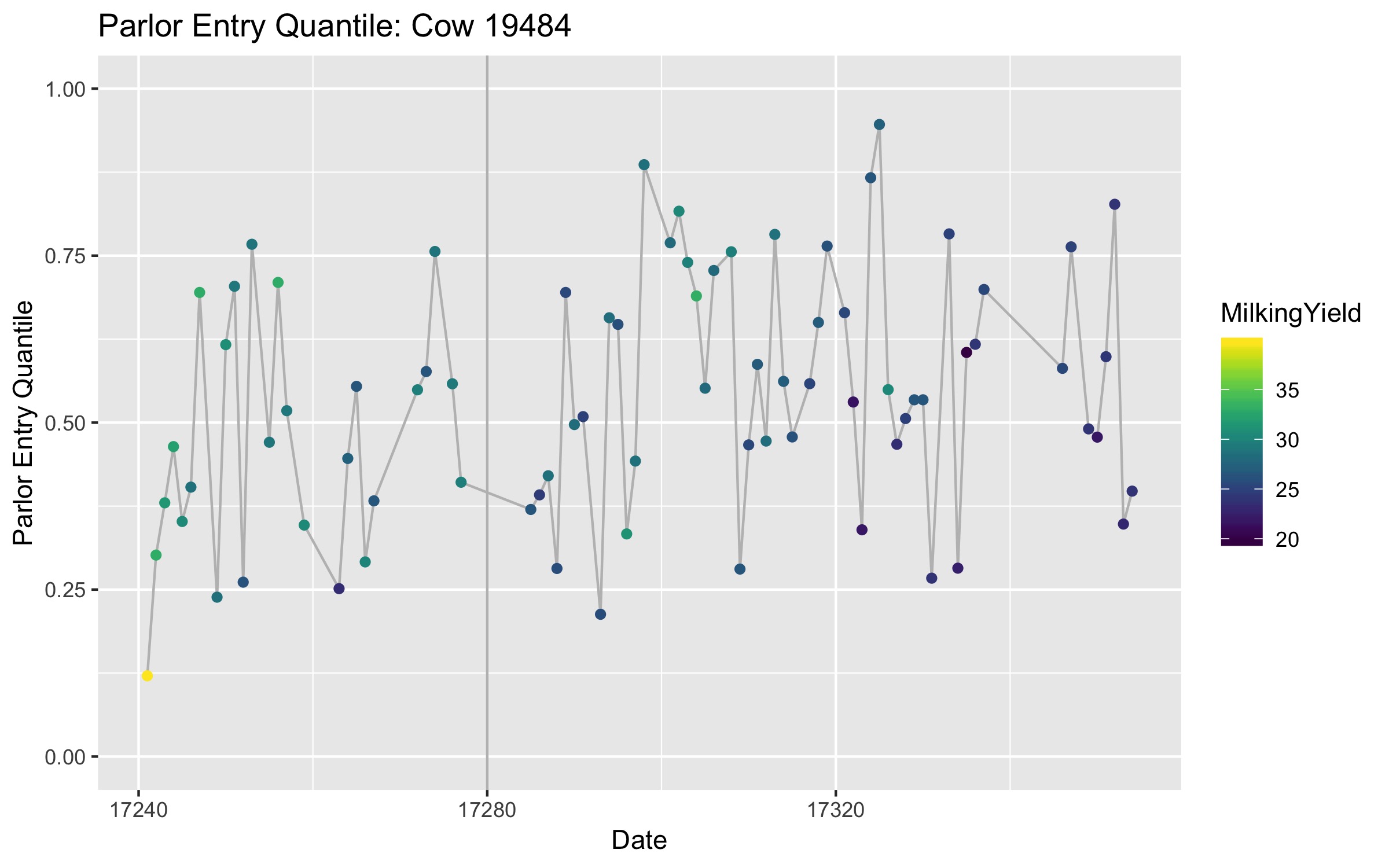

Supplement: Supplementary file 2 [file Data_Sheet_2.ZIP › Milking Yield/Cow_19484.jpg]

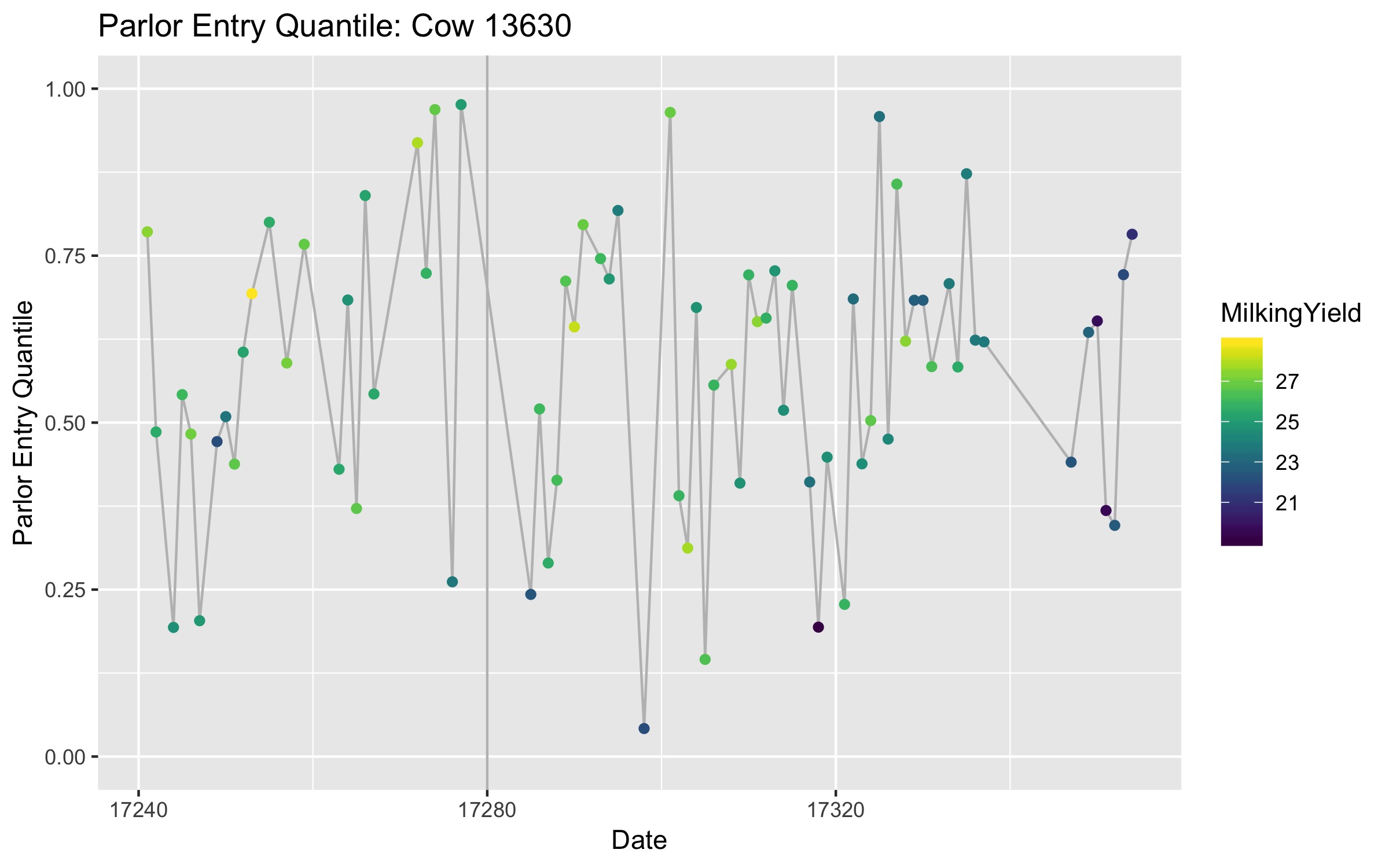

Supplement: Supplementary file 2 [file Data_Sheet_2.ZIP › Milking Yield/Cow_13630.jpg]

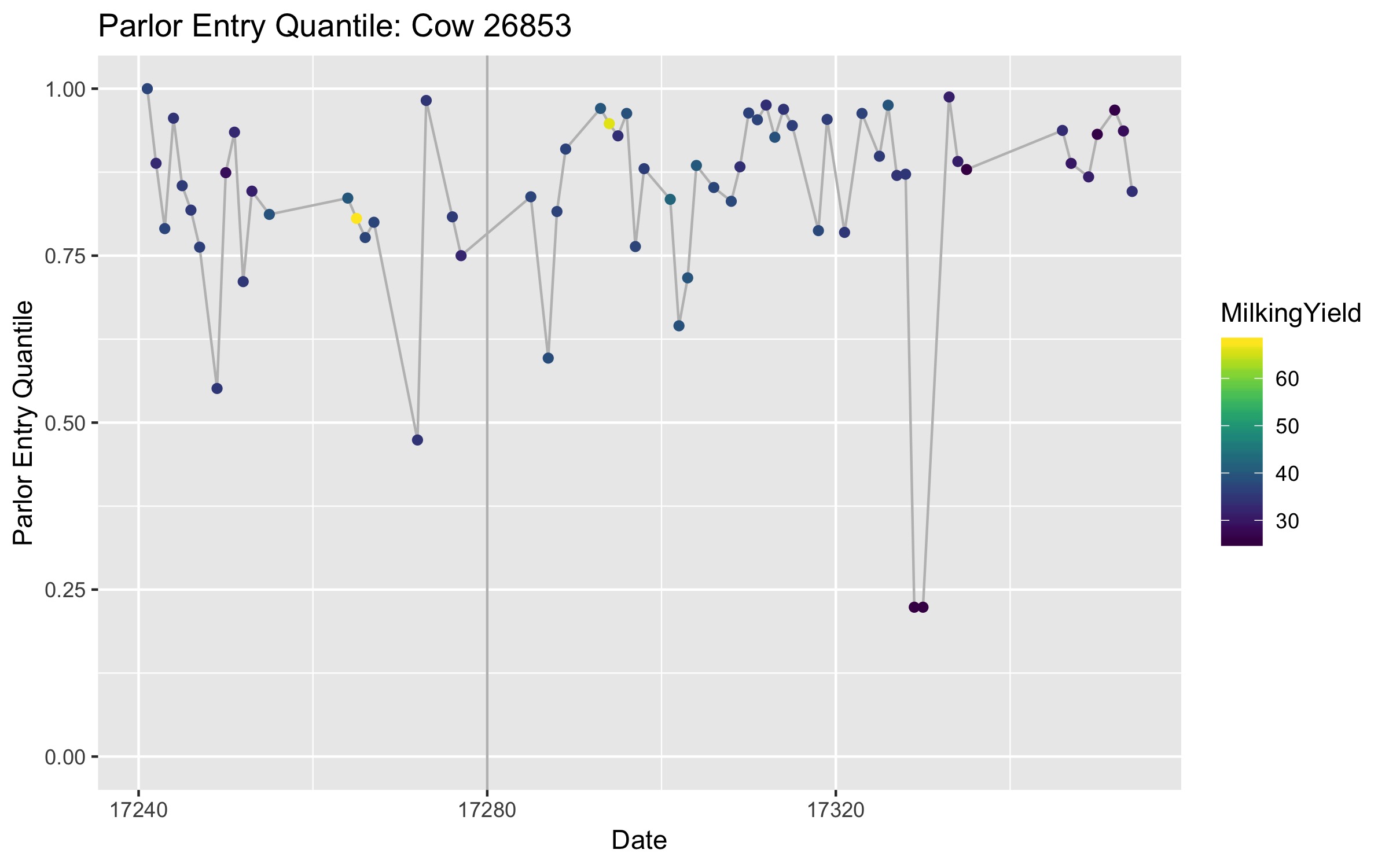

Supplement: Supplementary file 2 [file Data_Sheet_2.ZIP › Milking Yield/Cow_26853.jpg]

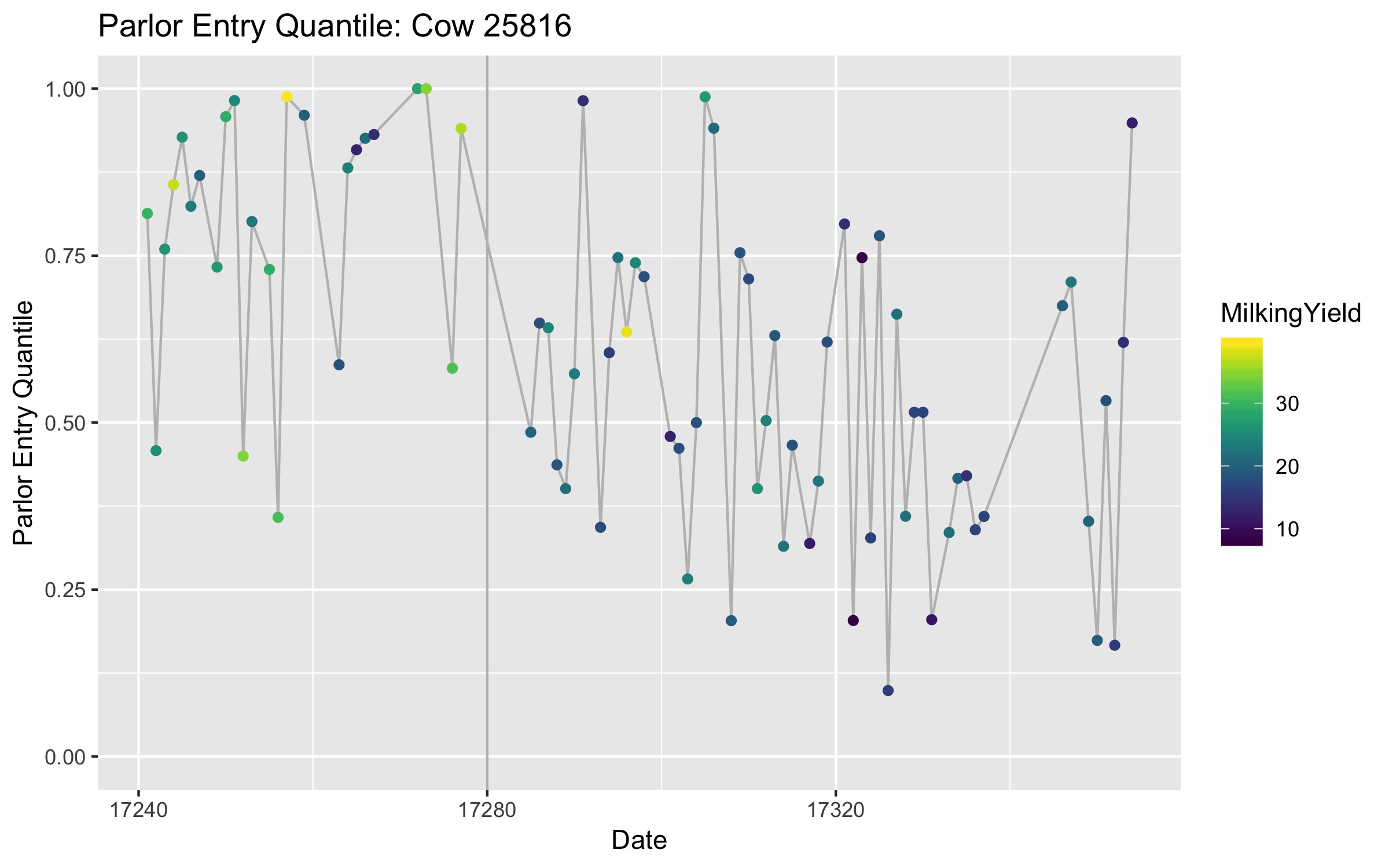

Supplement: Supplementary file 2 [file Data_Sheet_2.ZIP › Milking Yield/Cow_25816.jpg]

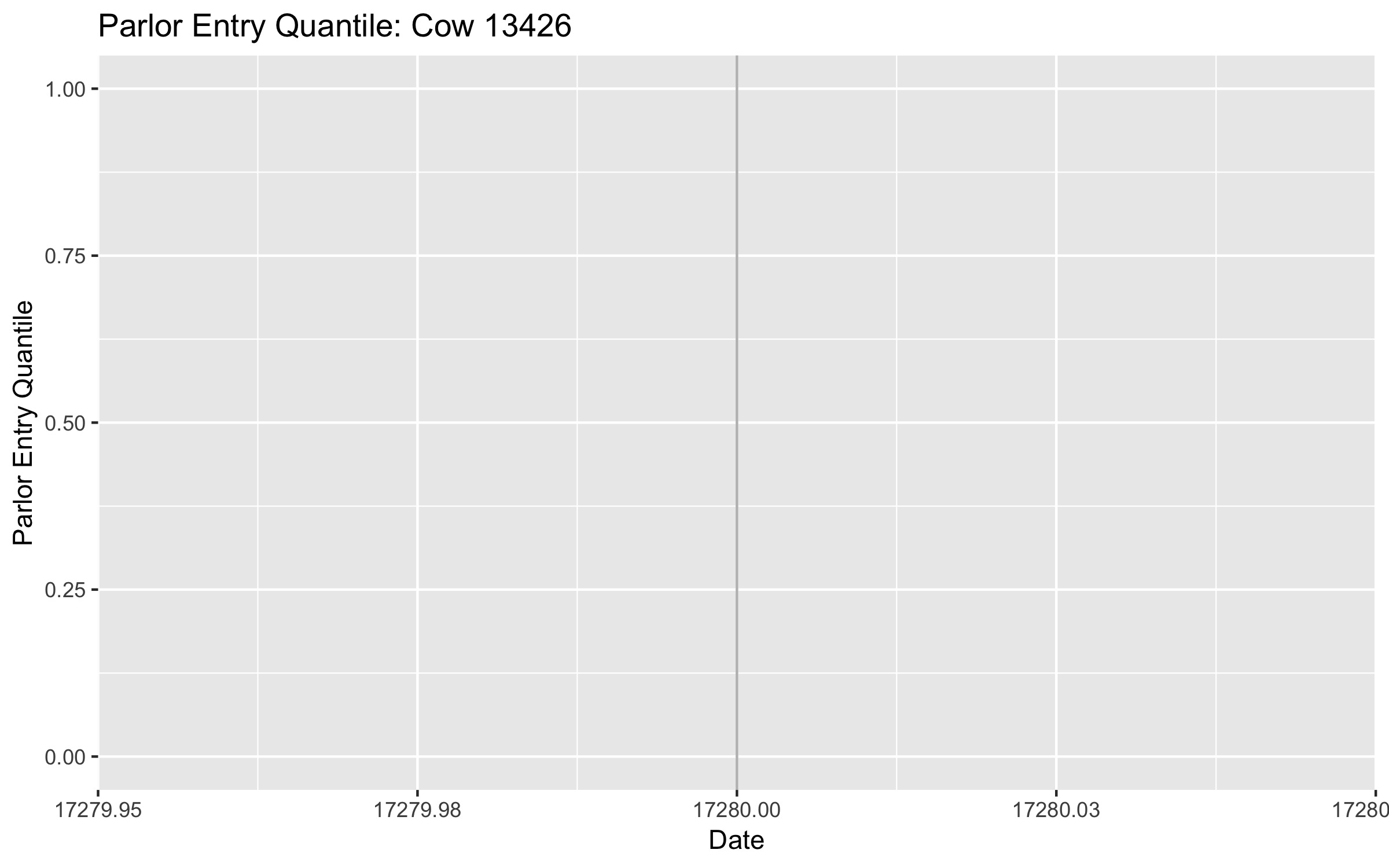

Supplement: Supplementary file 2 [file Data_Sheet_2.ZIP › Milking Yield/Cow_13426.jpg]

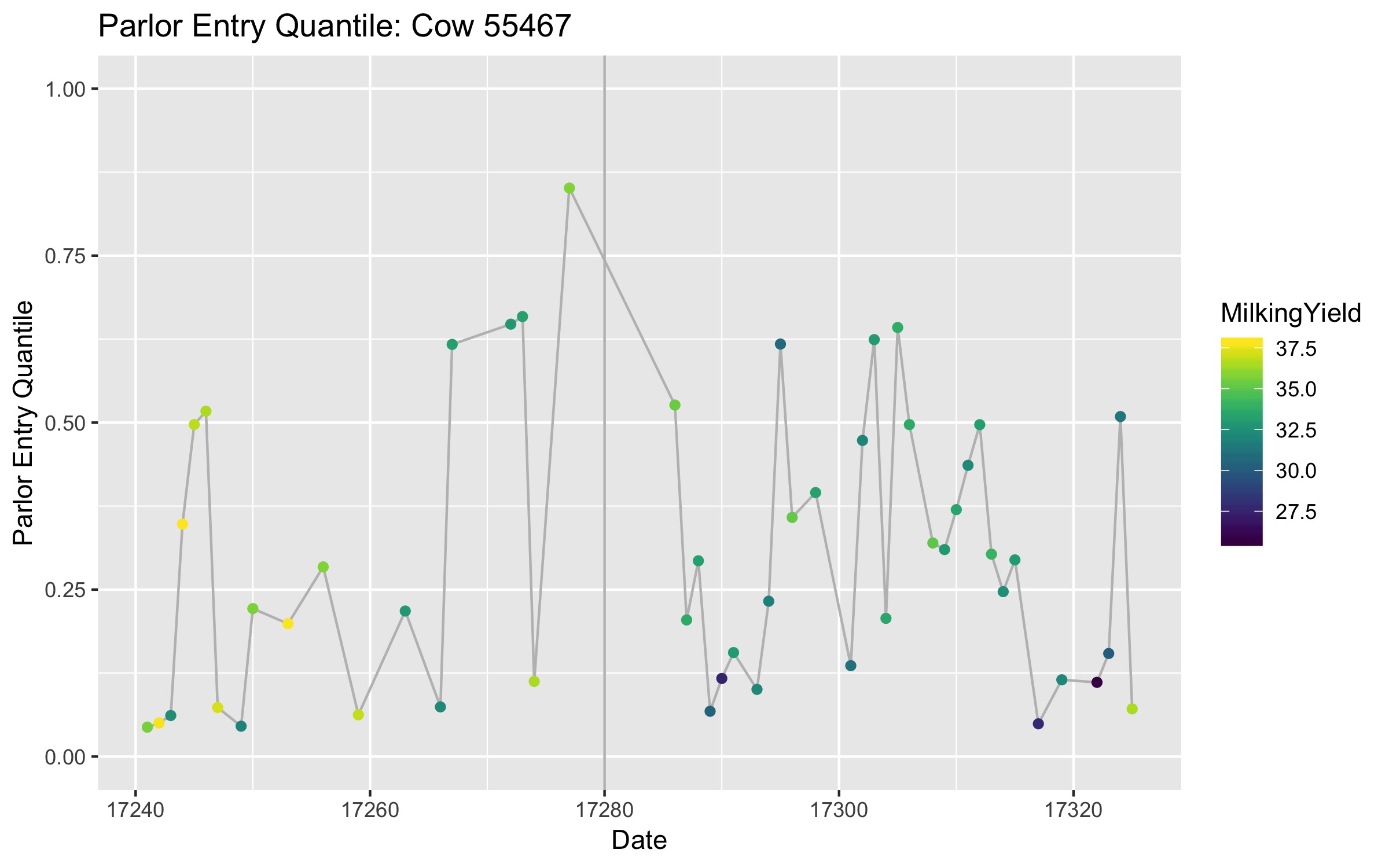

Supplement: Supplementary file 2 [file Data_Sheet_2.ZIP › Milking Yield/Cow_55467.jpg]

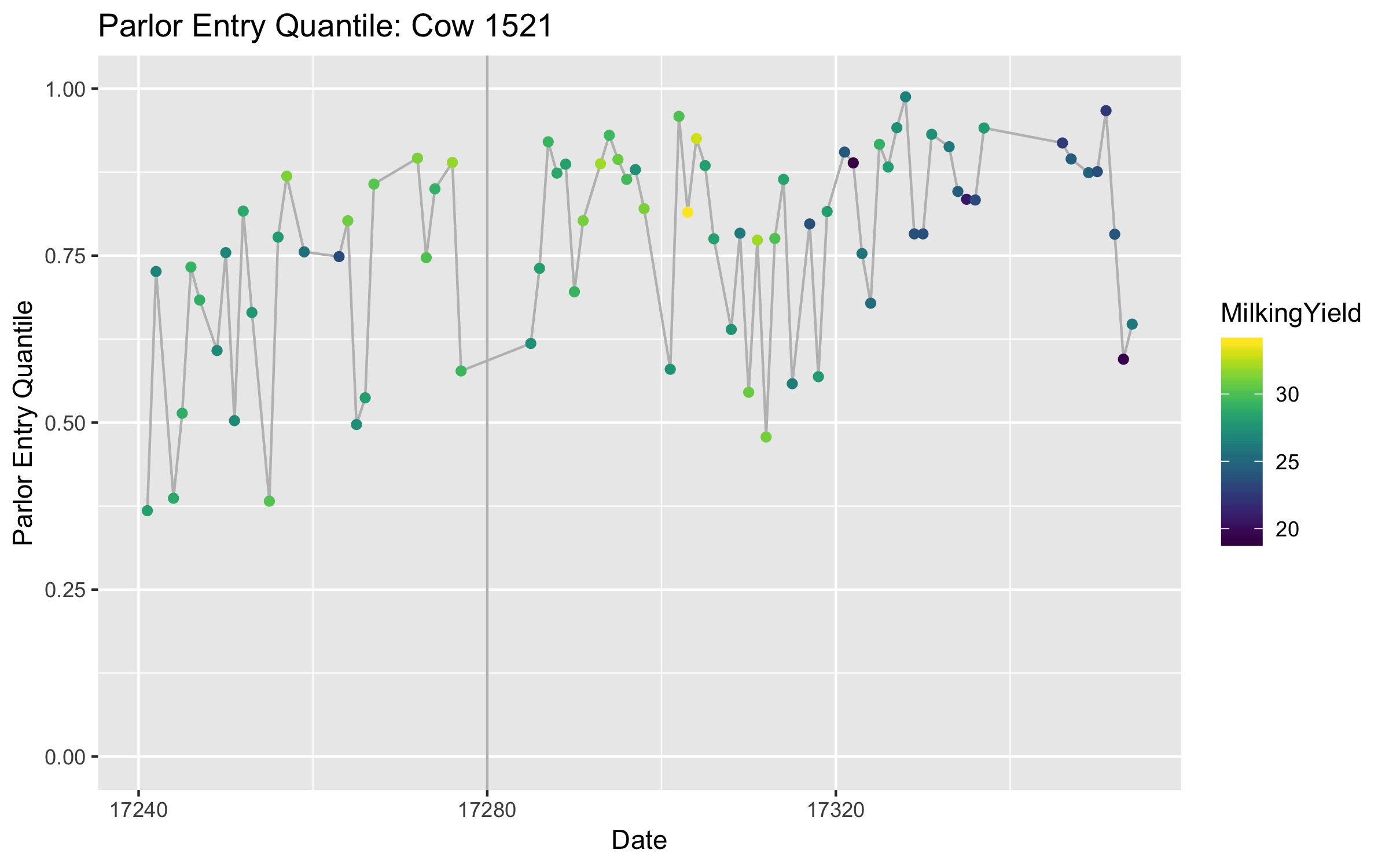

Supplement: Supplementary file 2 [file Data_Sheet_2.ZIP › Milking Yield/Cow_1521.jpg]

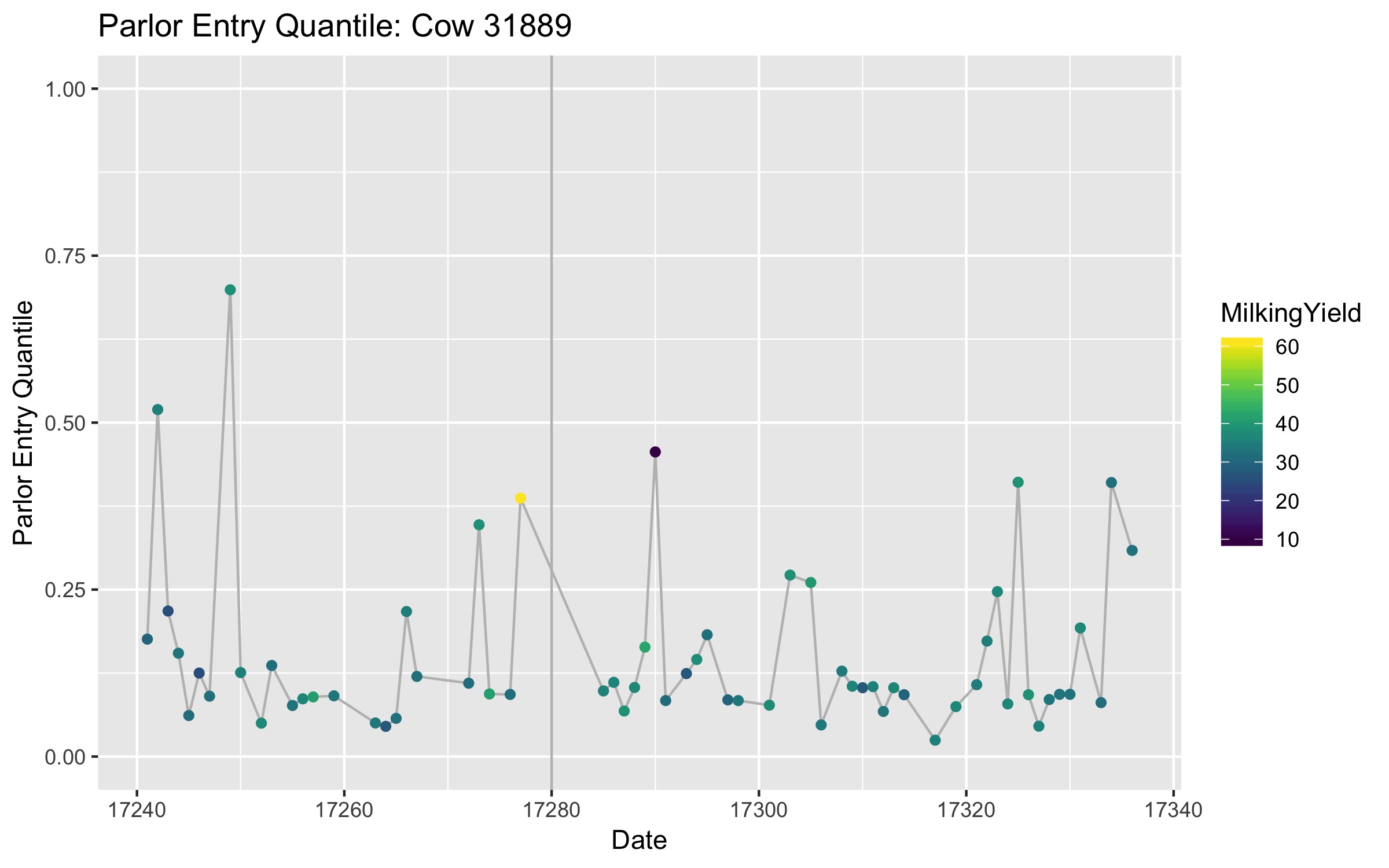

Supplement: Supplementary file 2 [file Data_Sheet_2.ZIP › Milking Yield/Cow_31889.jpg]

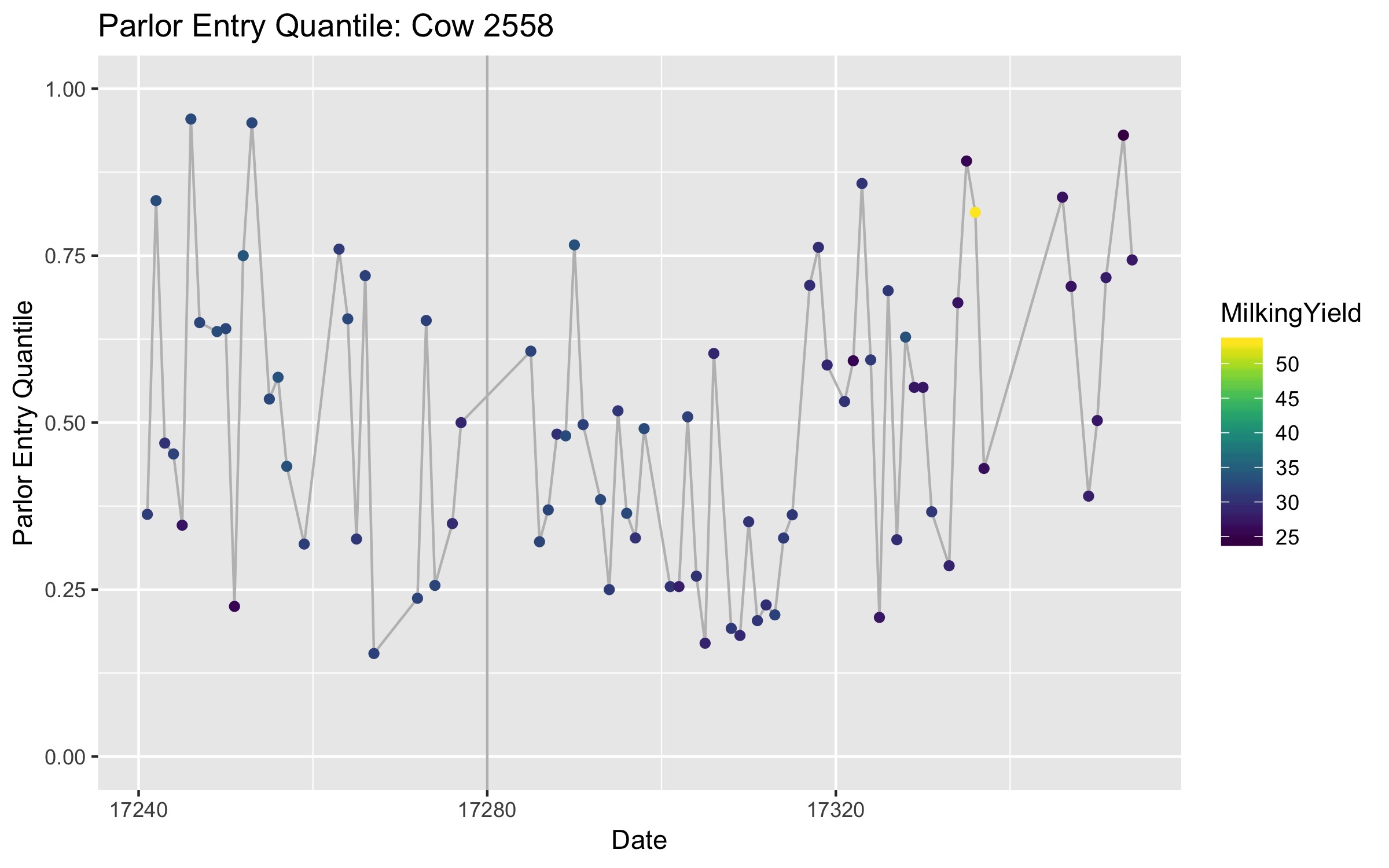

Supplement: Supplementary file 2 [file Data_Sheet_2.ZIP › Milking Yield/Cow_2558.jpg]

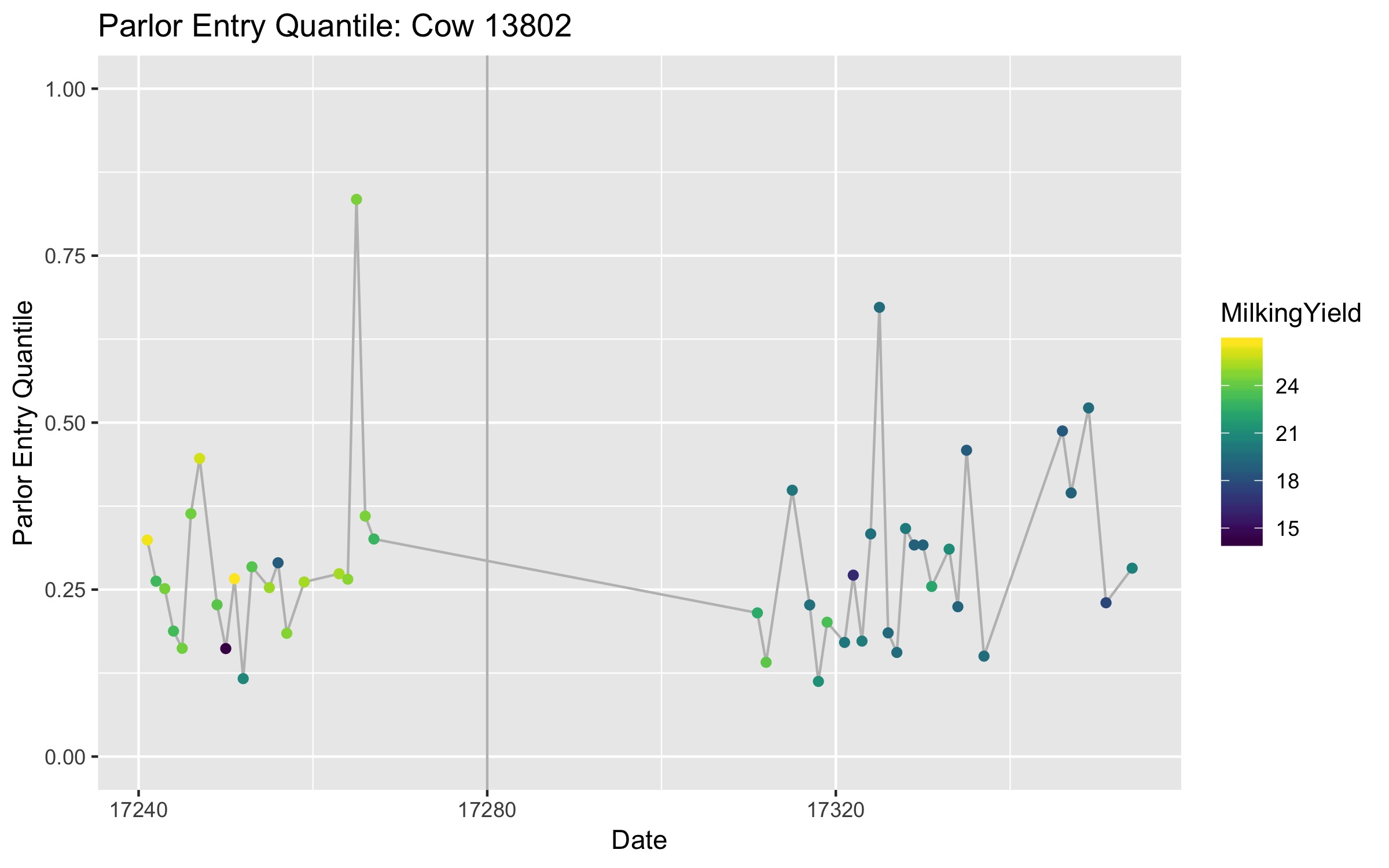

Supplement: Supplementary file 2 [file Data_Sheet_2.ZIP › Milking Yield/Cow_13802.jpg]

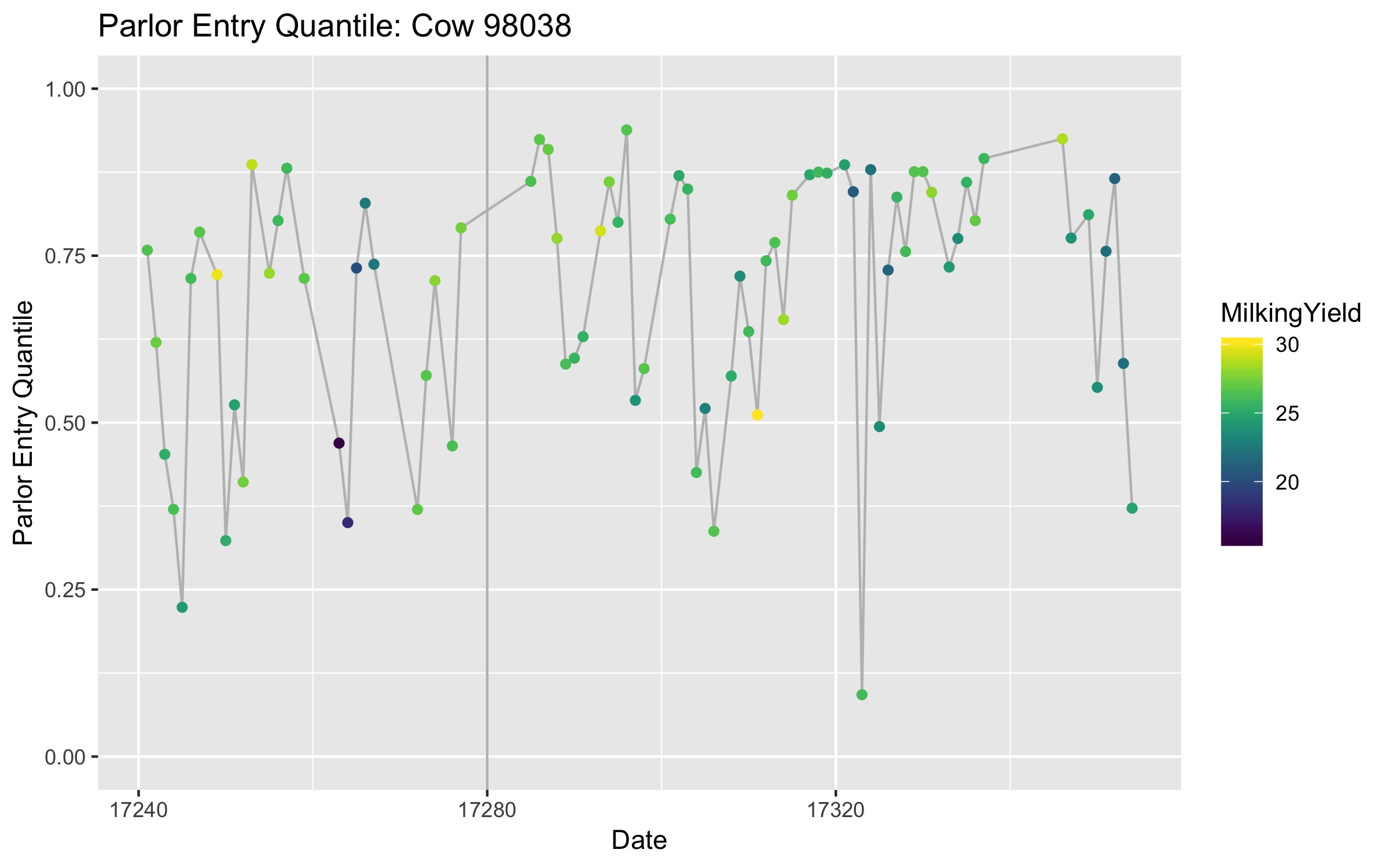

Supplement: Supplementary file 2 [file Data_Sheet_2.ZIP › Milking Yield/Cow_98038.jpg]

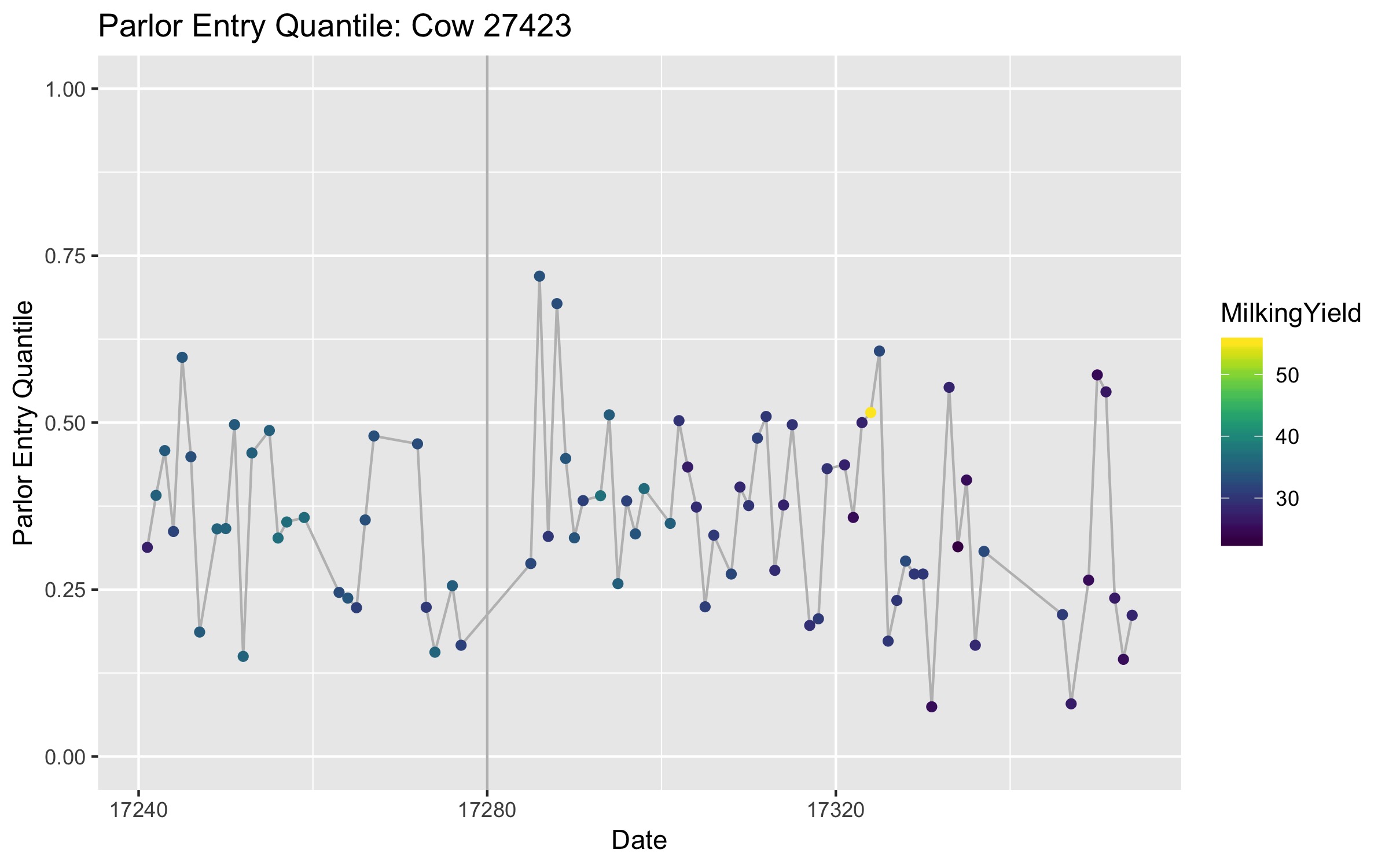

Supplement: Supplementary file 2 [file Data_Sheet_2.ZIP › Milking Yield/Cow_27423.jpg]

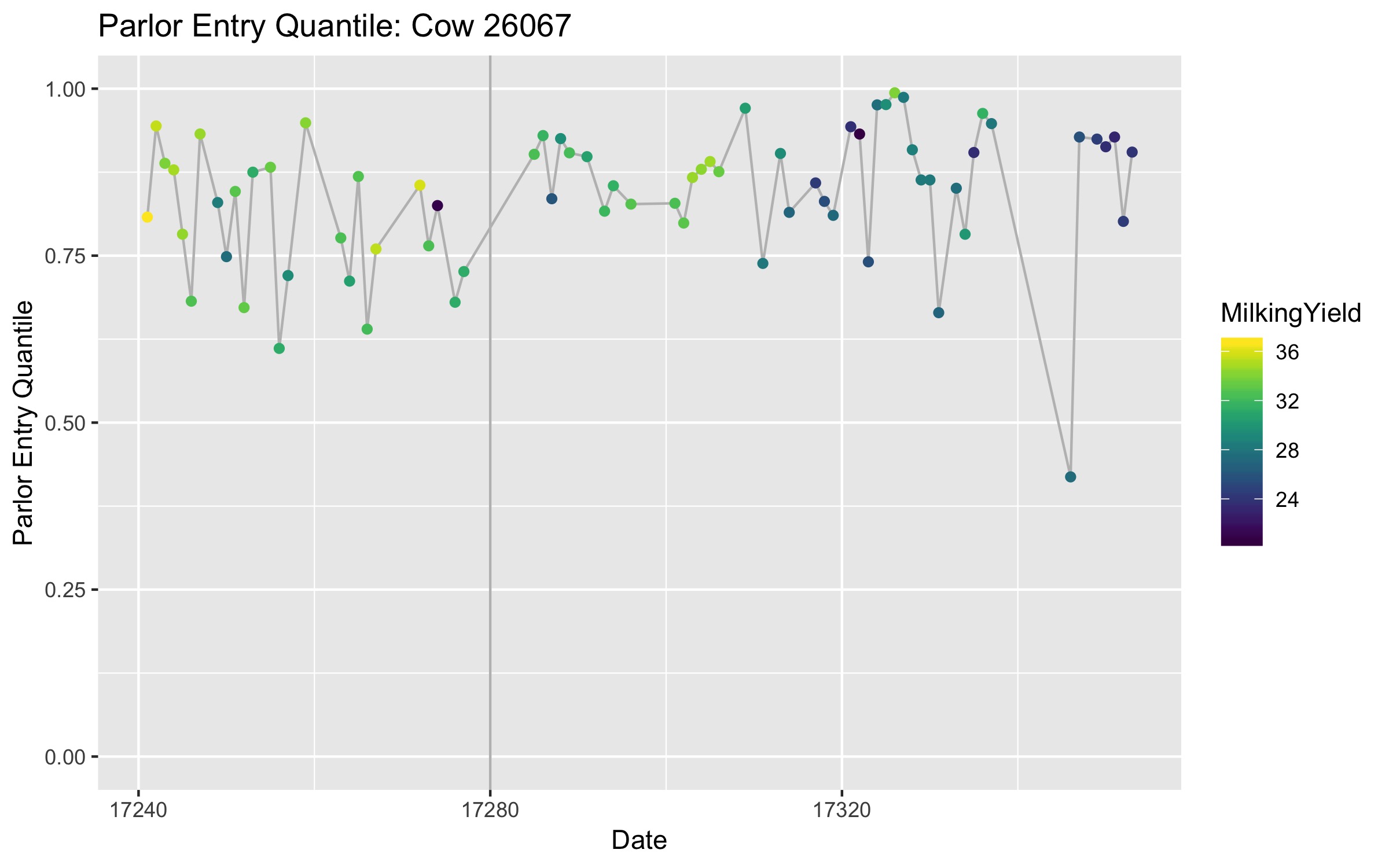

Supplement: Supplementary file 2 [file Data_Sheet_2.ZIP › Milking Yield/Cow_26067.jpg]

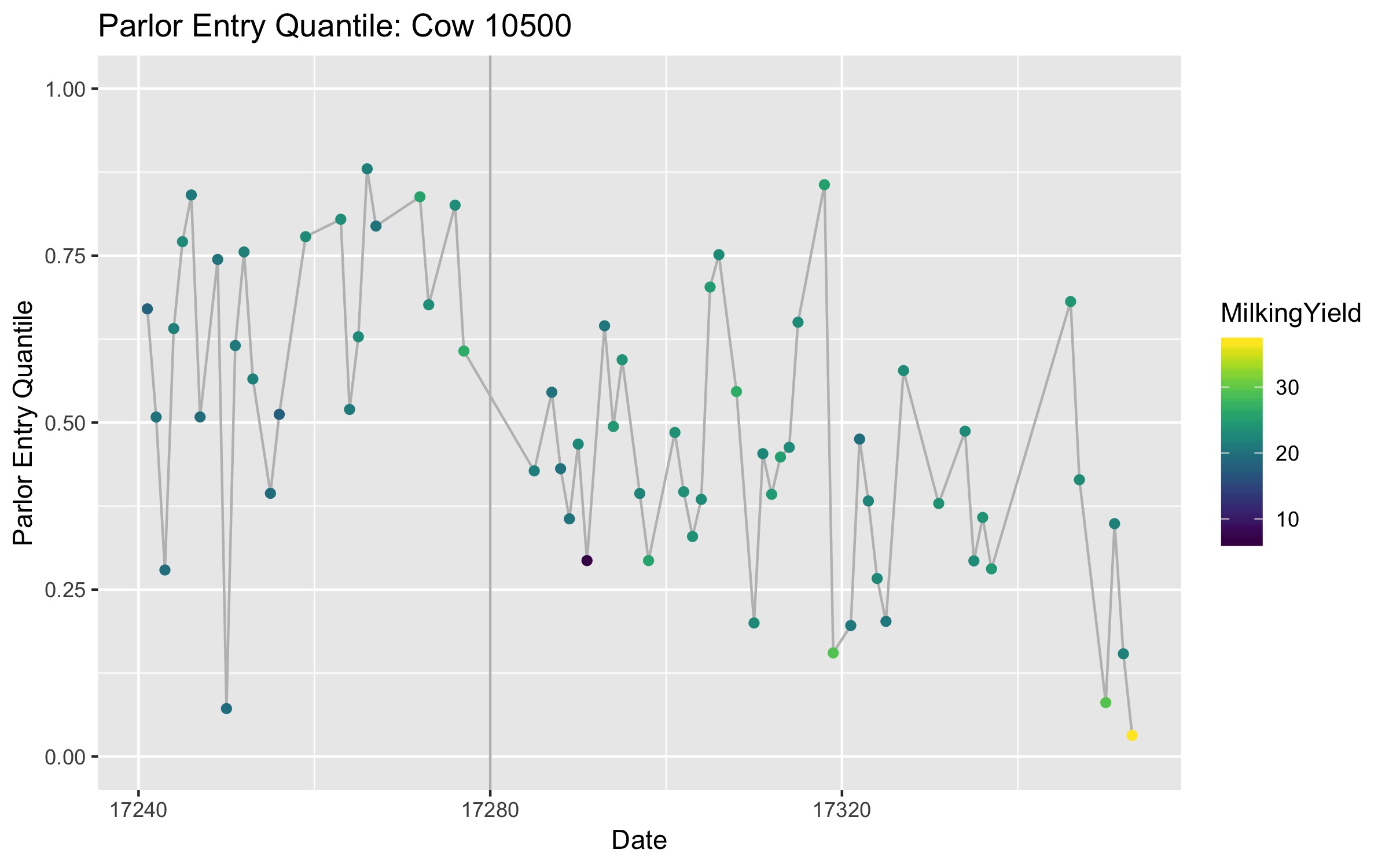

Supplement: Supplementary file 2 [file Data_Sheet_2.ZIP › Milking Yield/Cow_10500.jpg]

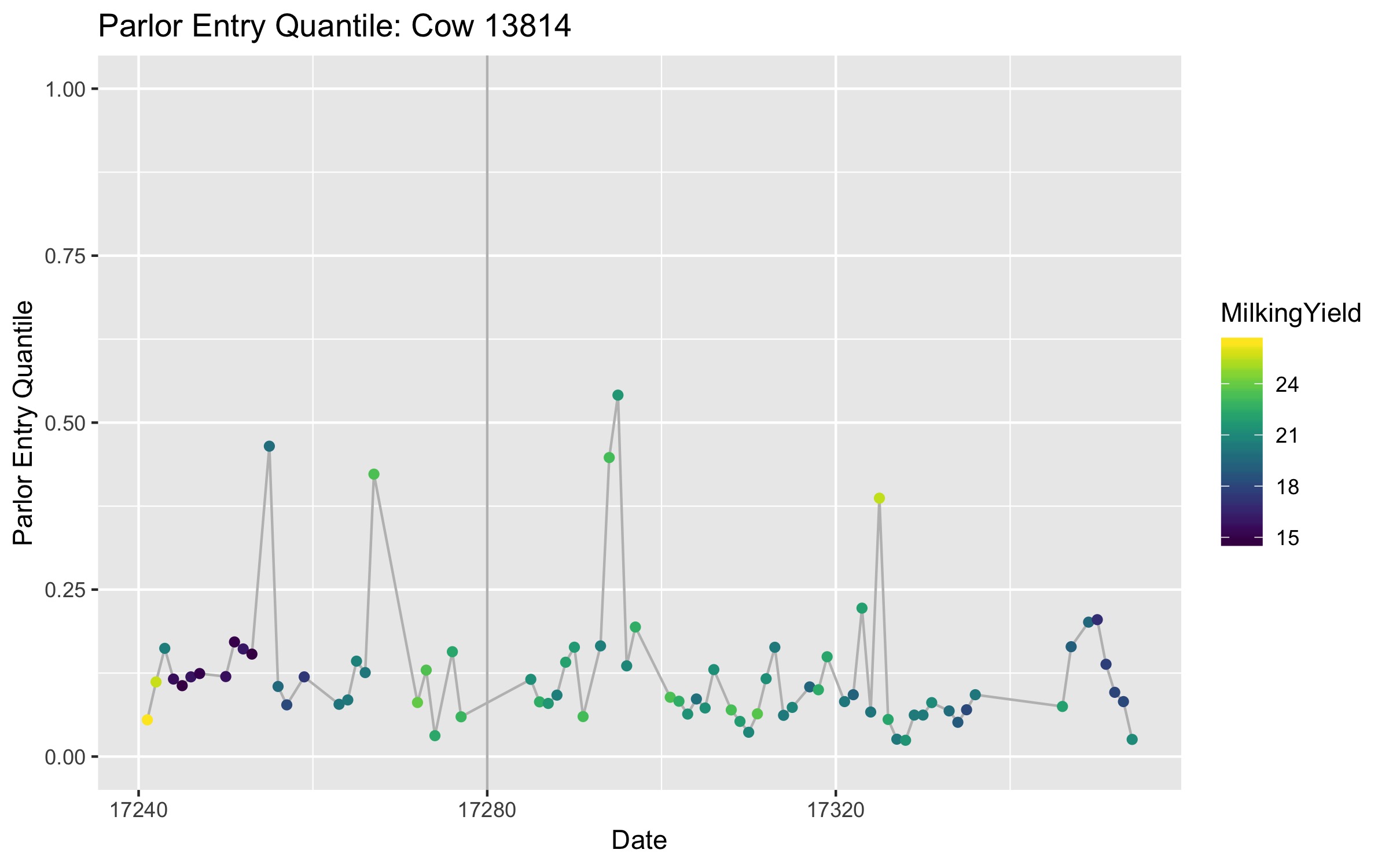

Supplement: Supplementary file 2 [file Data_Sheet_2.ZIP › Milking Yield/Cow_13814.jpg]

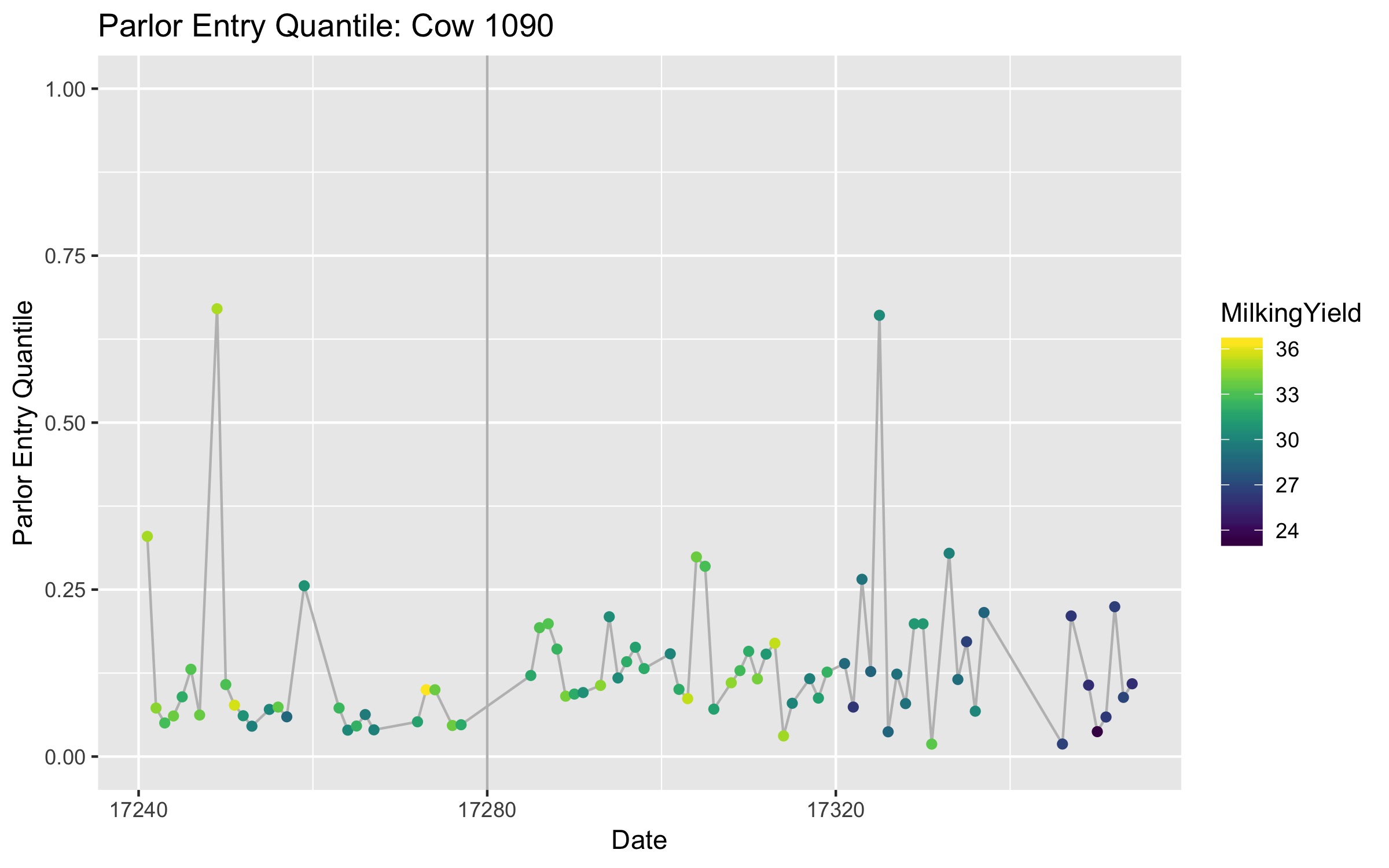

Supplement: Supplementary file 2 [file Data_Sheet_2.ZIP › Milking Yield/Cow_1090.jpg]

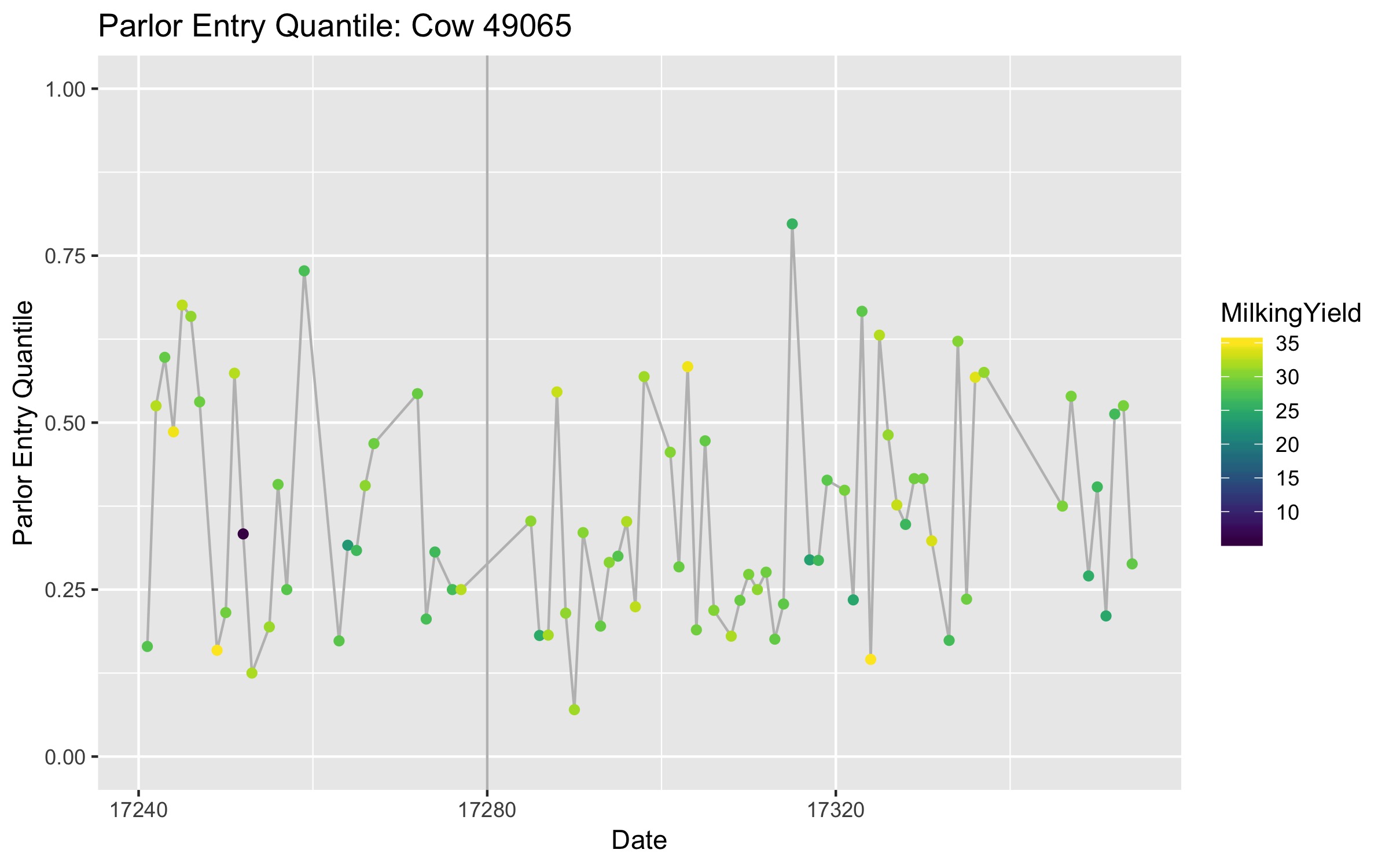

Supplement: Supplementary file 2 [file Data_Sheet_2.ZIP › Milking Yield/Cow_49065.jpg]

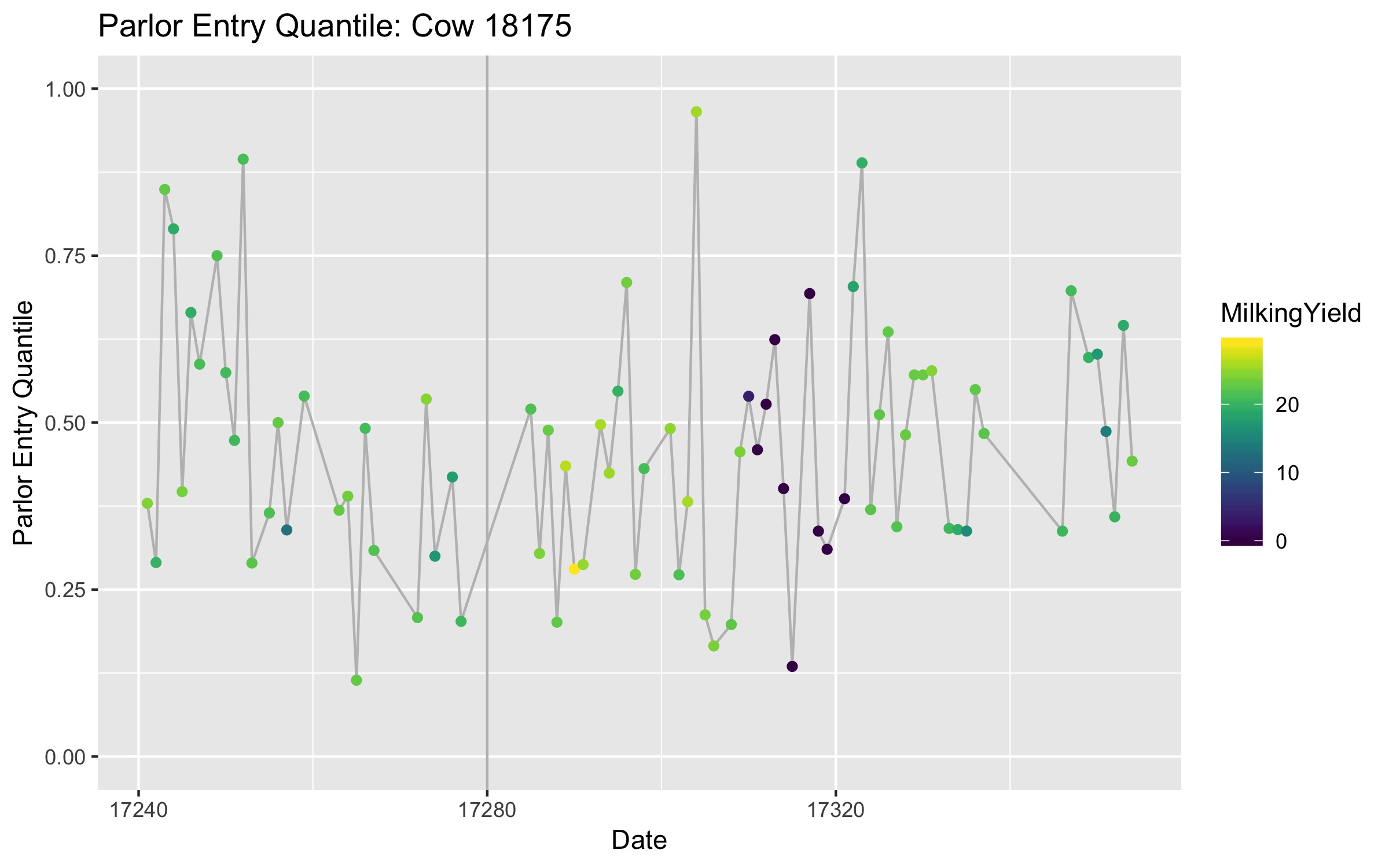

Supplement: Supplementary file 2 [file Data_Sheet_2.ZIP › Milking Yield/Cow_18175.jpg]

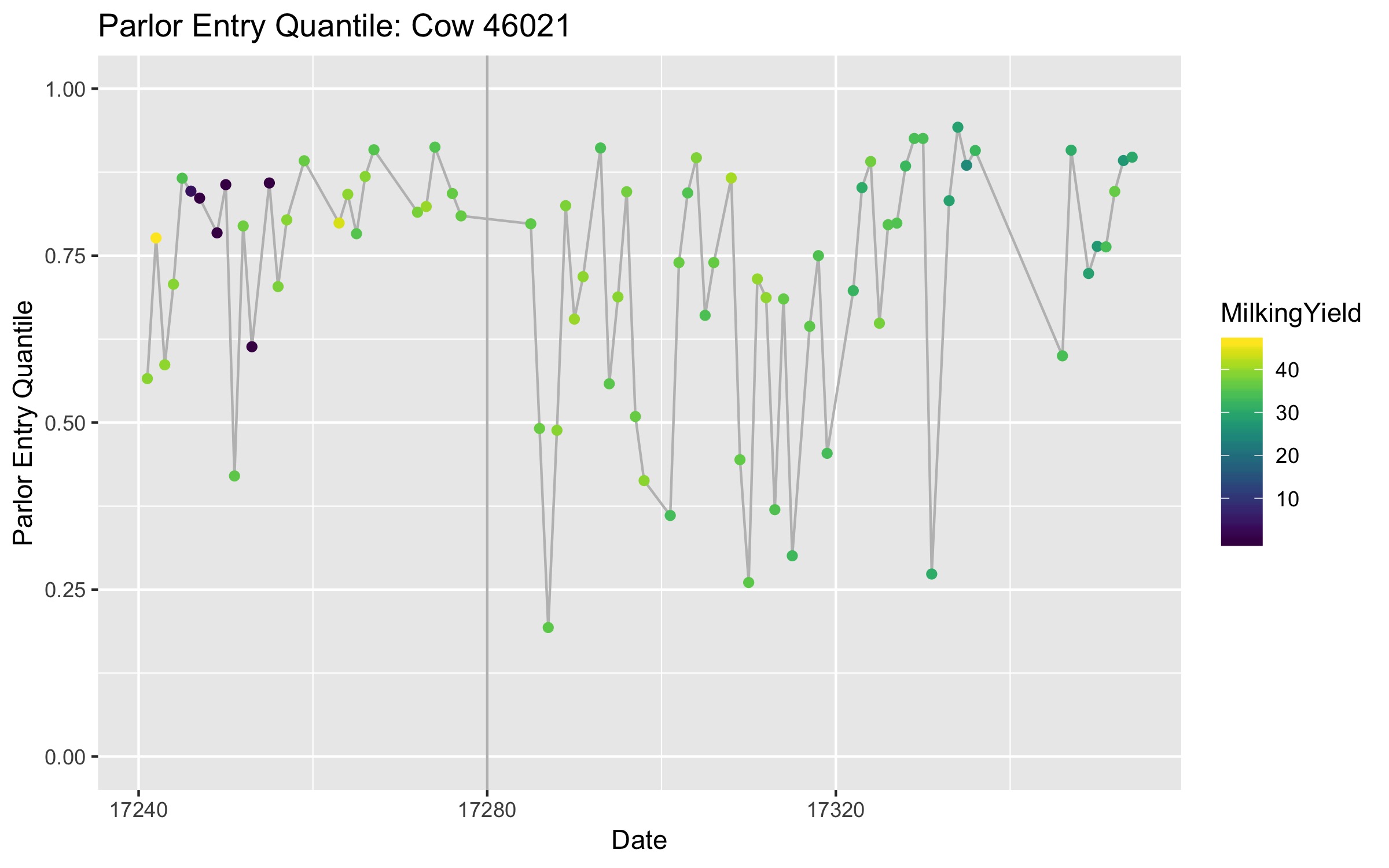

Supplement: Supplementary file 2 [file Data_Sheet_2.ZIP › Milking Yield/Cow_46021.jpg]

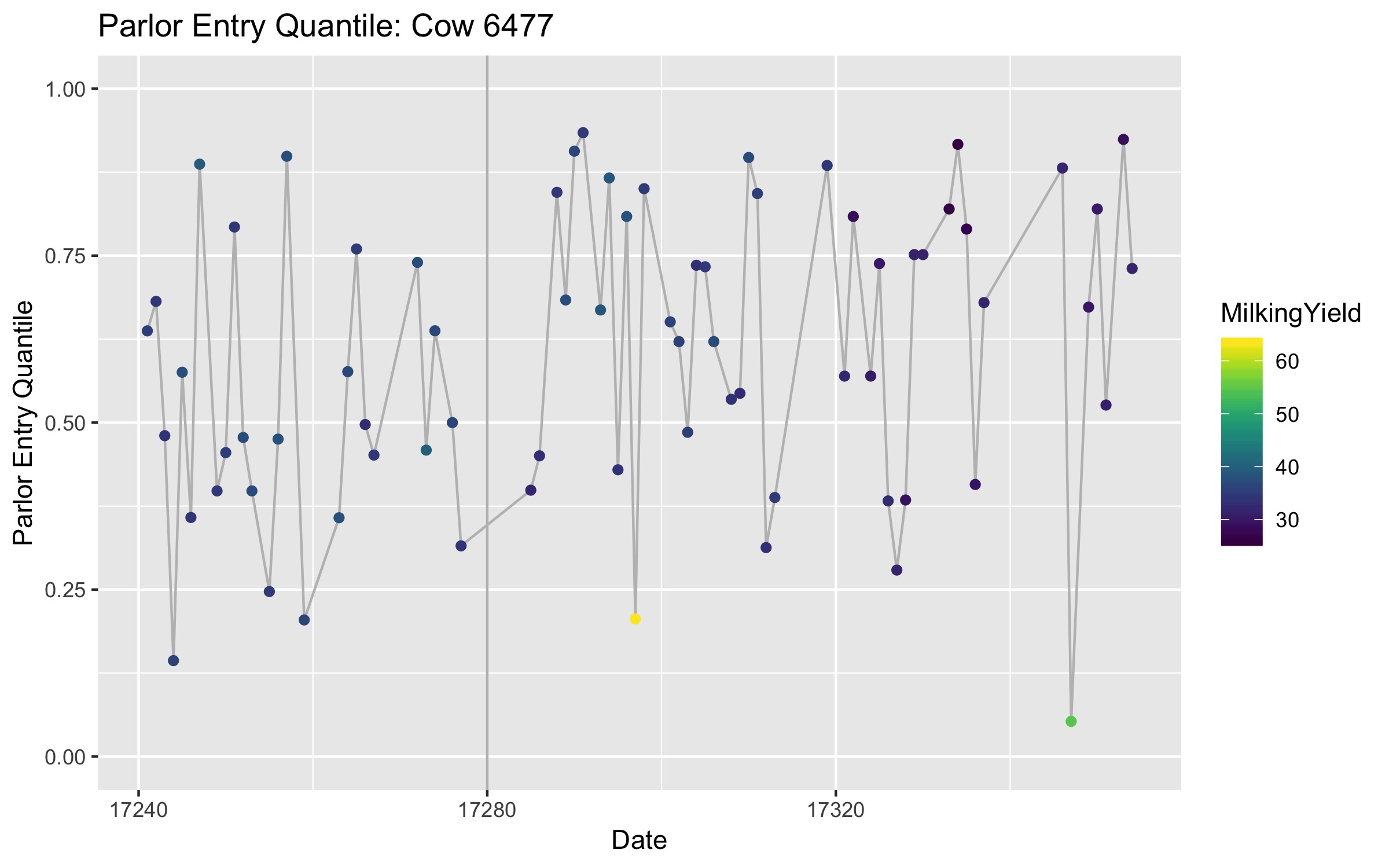

Supplement: Supplementary file 2 [file Data_Sheet_2.ZIP › Milking Yield/Cow_6477.jpg]

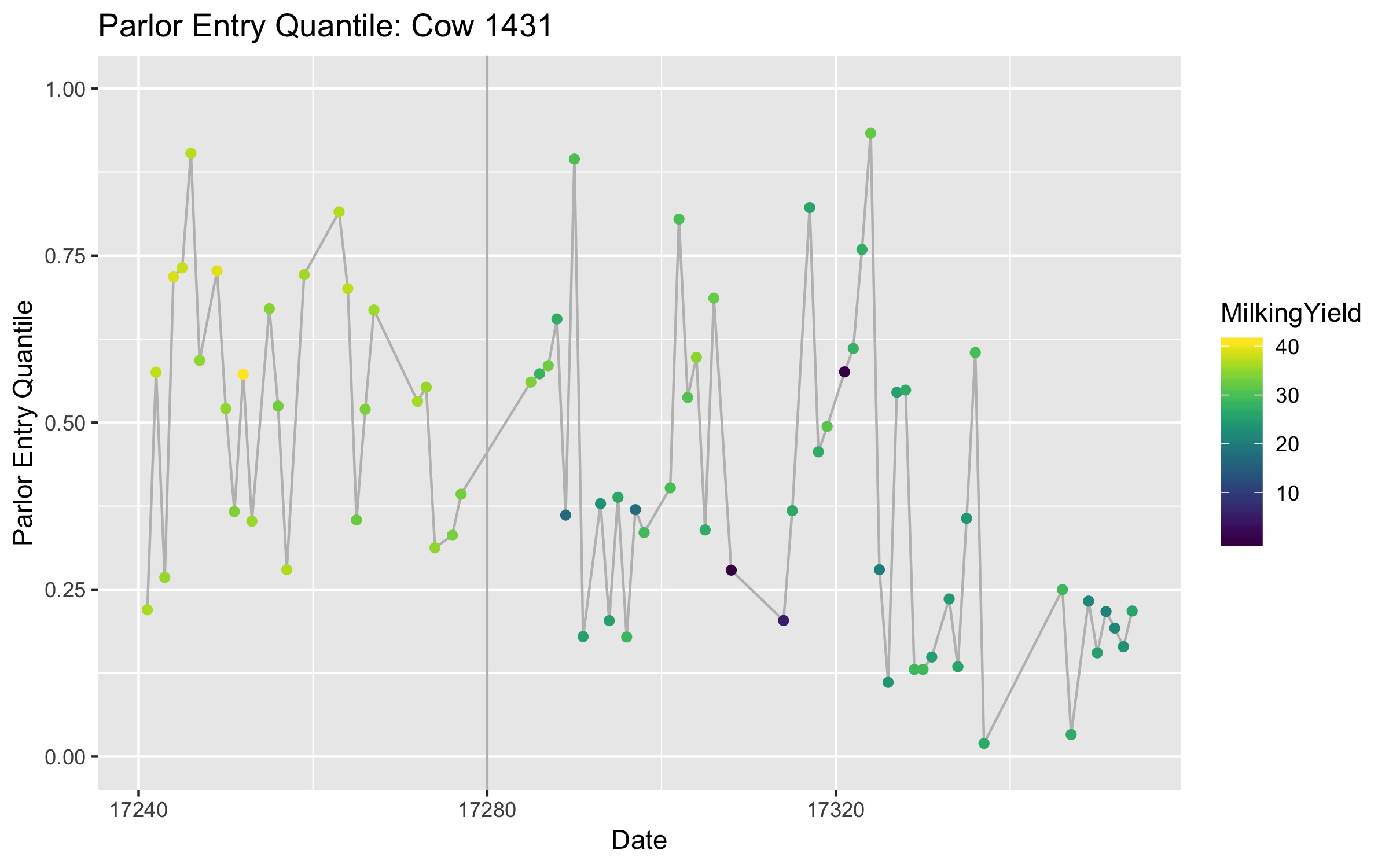

Supplement: Supplementary file 2 [file Data_Sheet_2.ZIP › Milking Yield/Cow_1431.jpg]

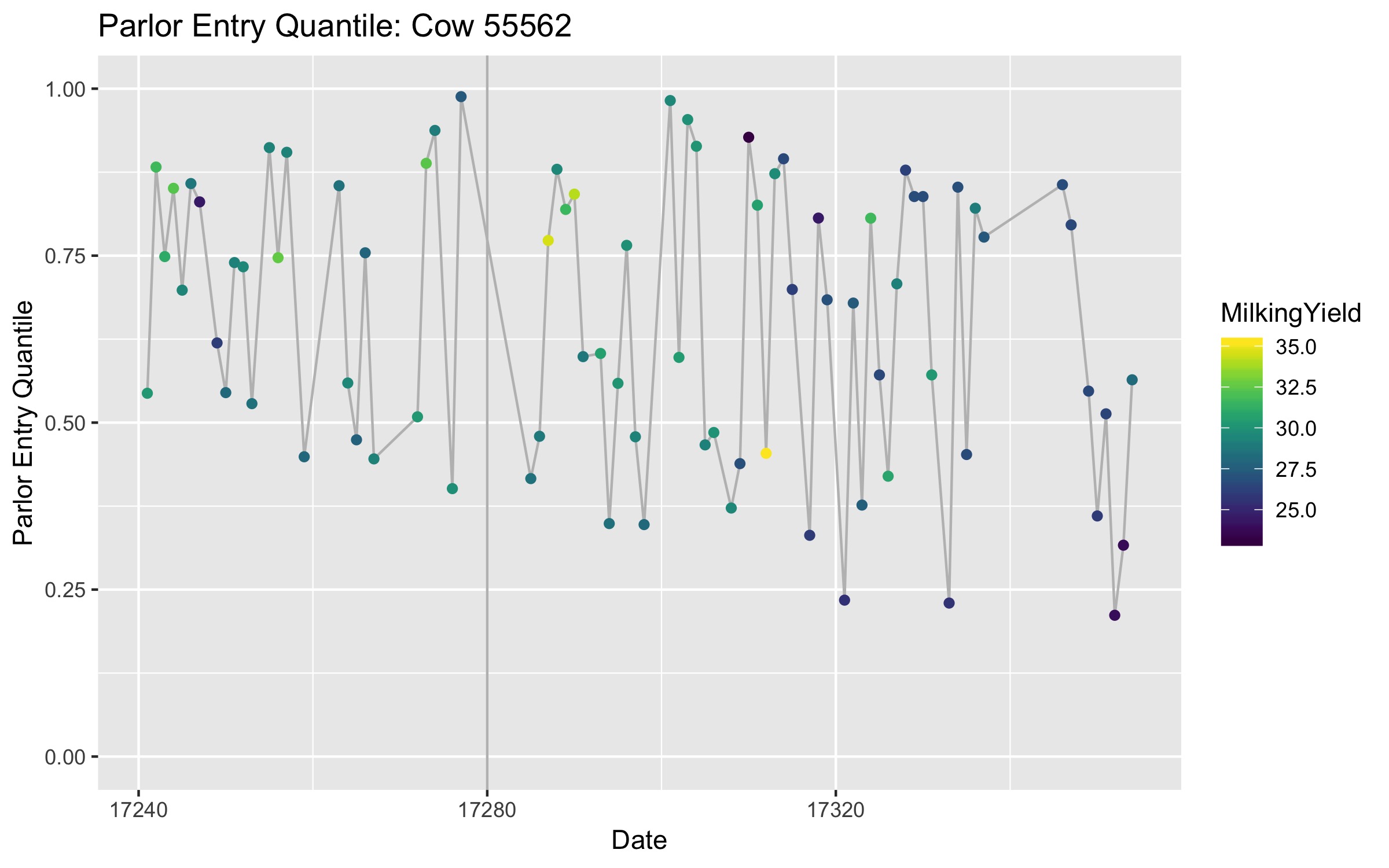

Supplement: Supplementary file 2 [file Data_Sheet_2.ZIP › Milking Yield/Cow_55562.jpg]

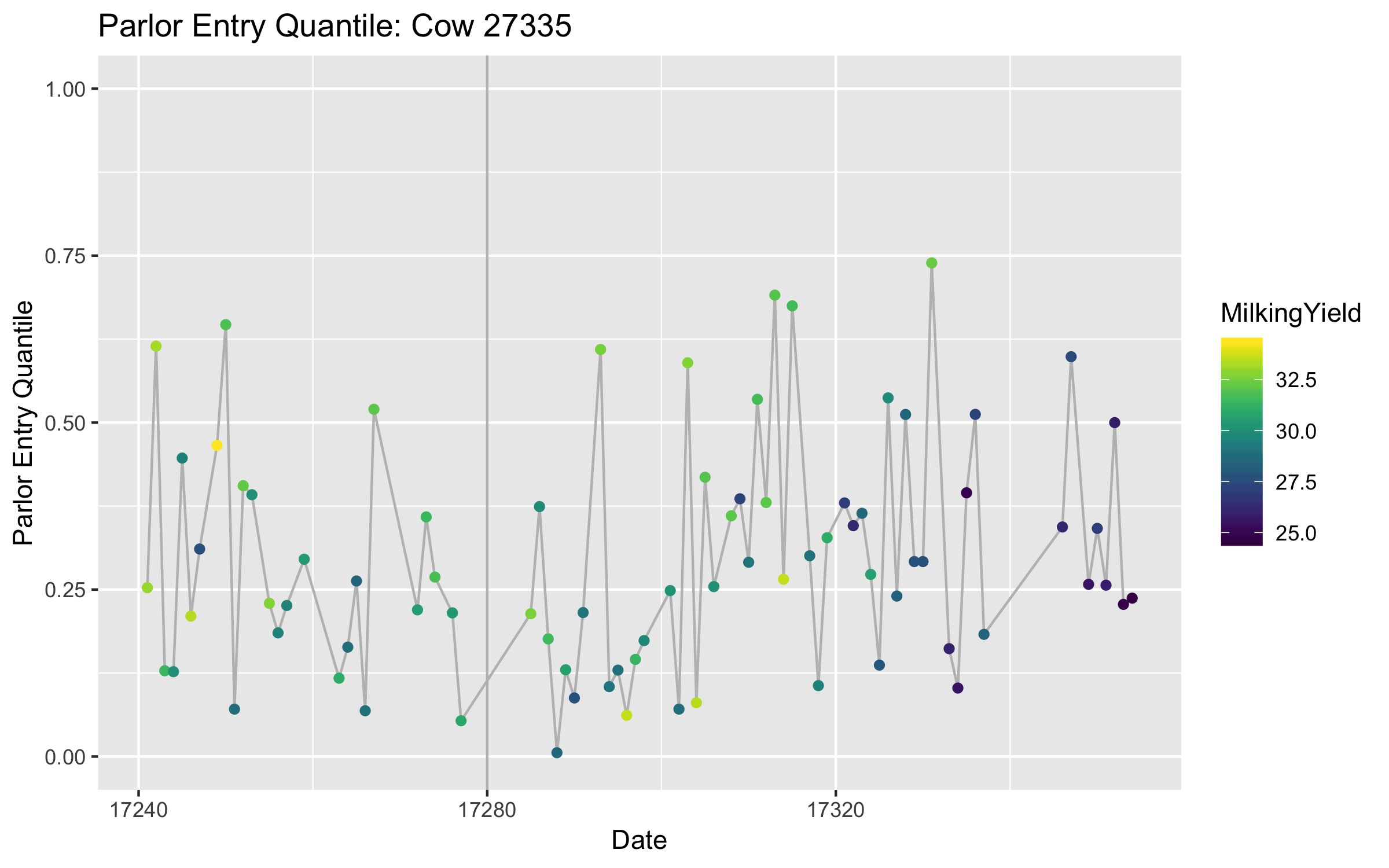

Supplement: Supplementary file 2 [file Data_Sheet_2.ZIP › Milking Yield/Cow_27335.jpg]

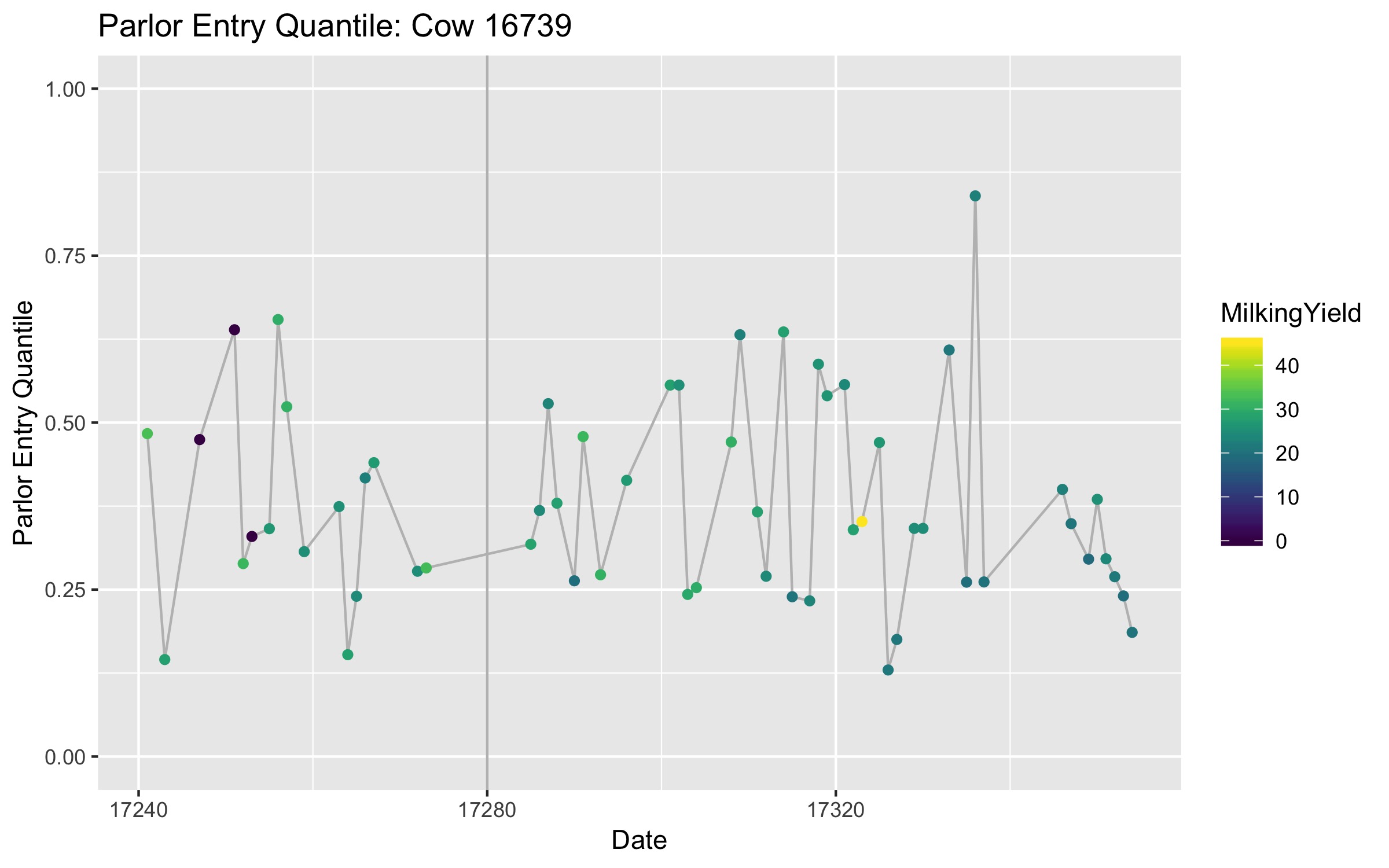

Supplement: Supplementary file 2 [file Data_Sheet_2.ZIP › Milking Yield/Cow_16739.jpg]

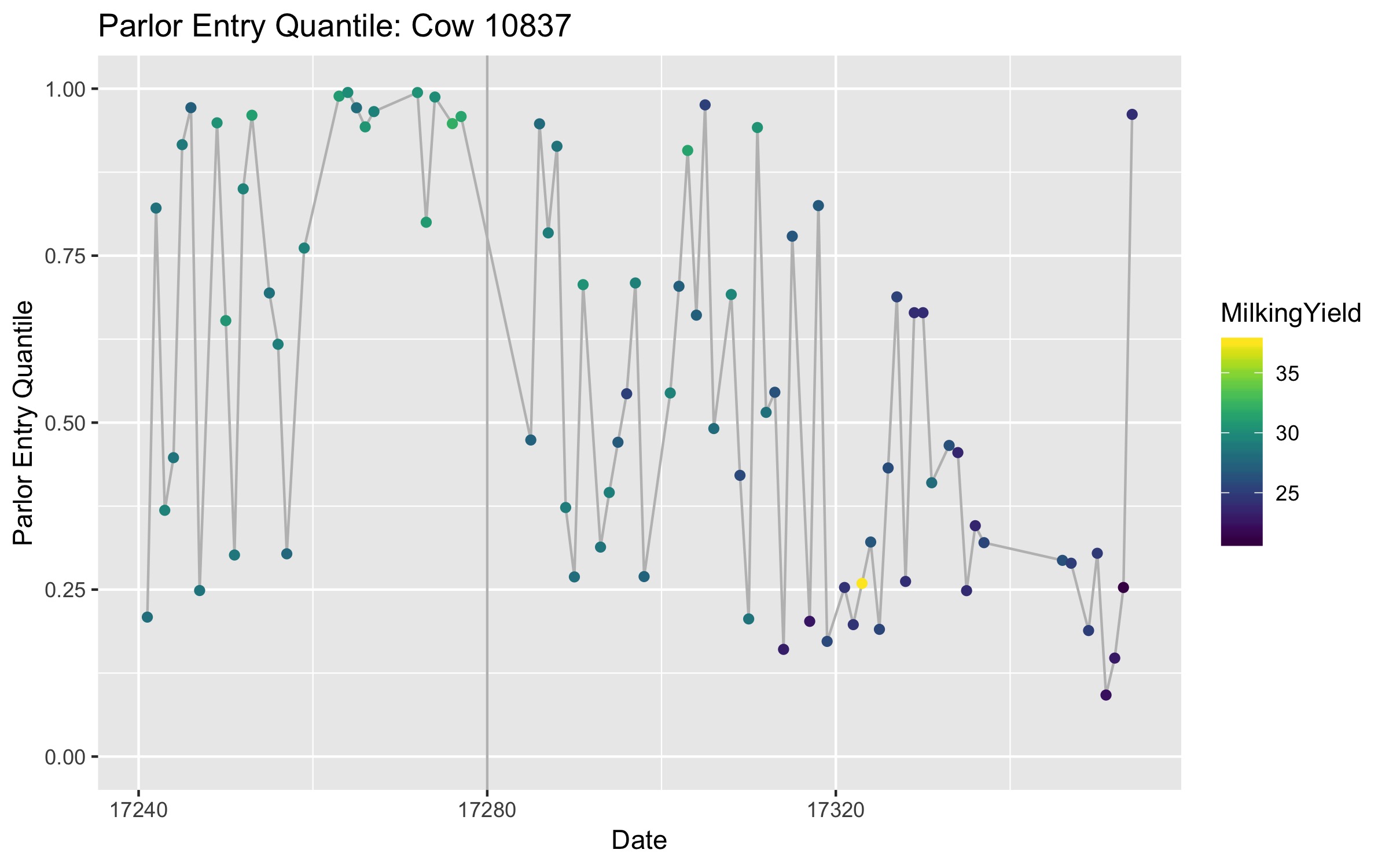

Supplement: Supplementary file 2 [file Data_Sheet_2.ZIP › Milking Yield/Cow_10837.jpg]

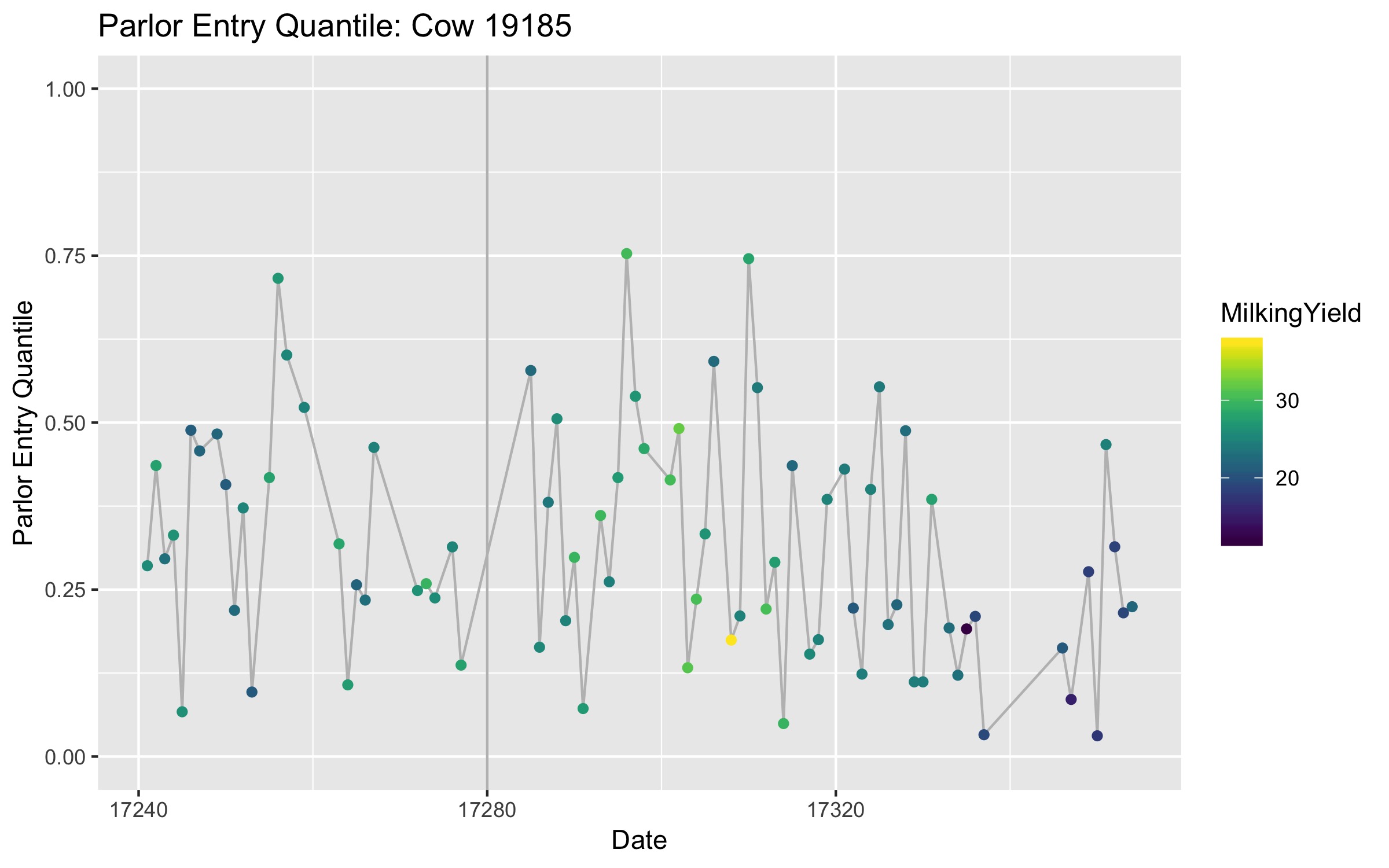

Supplement: Supplementary file 2 [file Data_Sheet_2.ZIP › Milking Yield/Cow_19185.jpg]

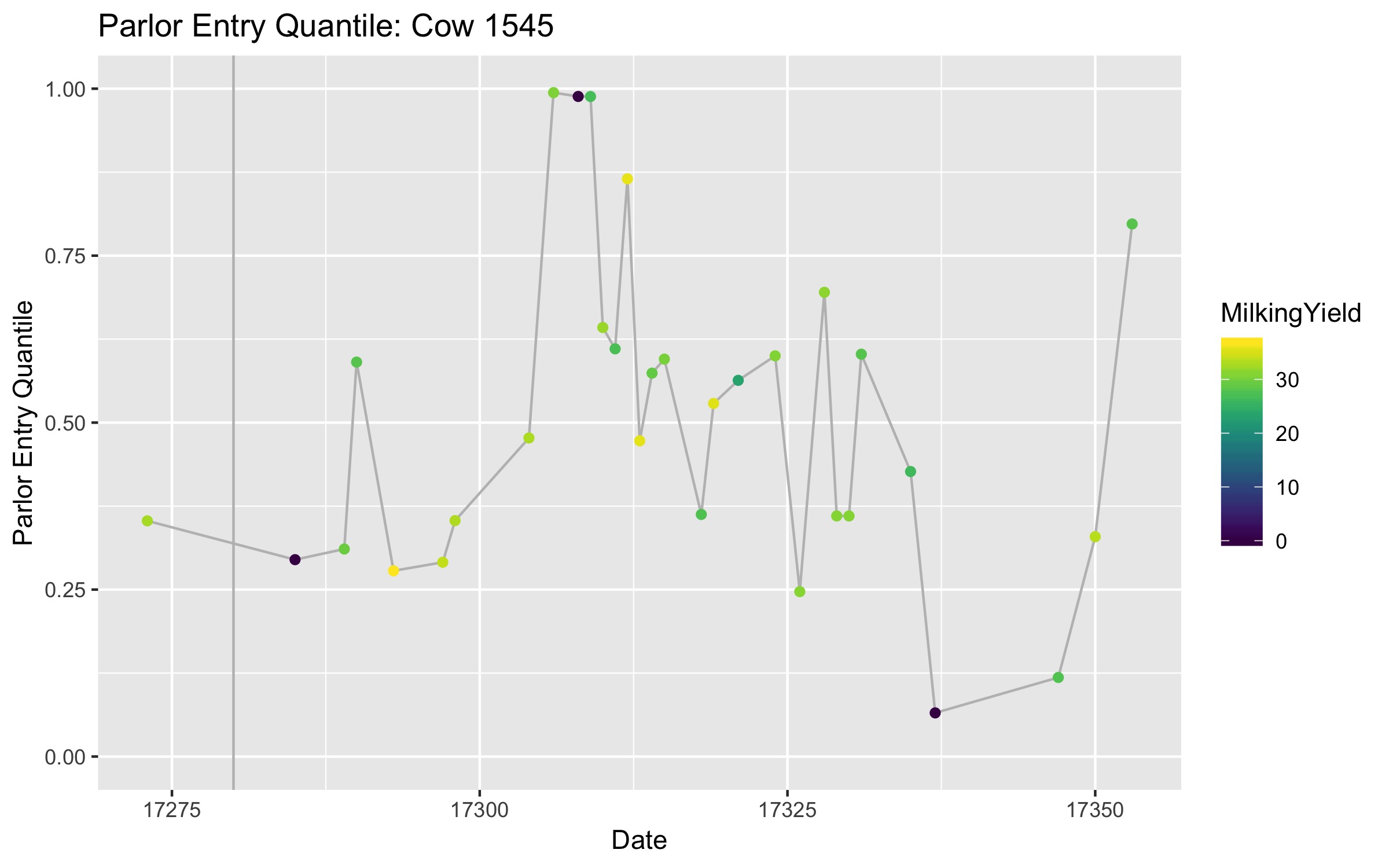

Supplement: Supplementary file 2 [file Data_Sheet_2.ZIP › Milking Yield/Cow_1545.jpg]

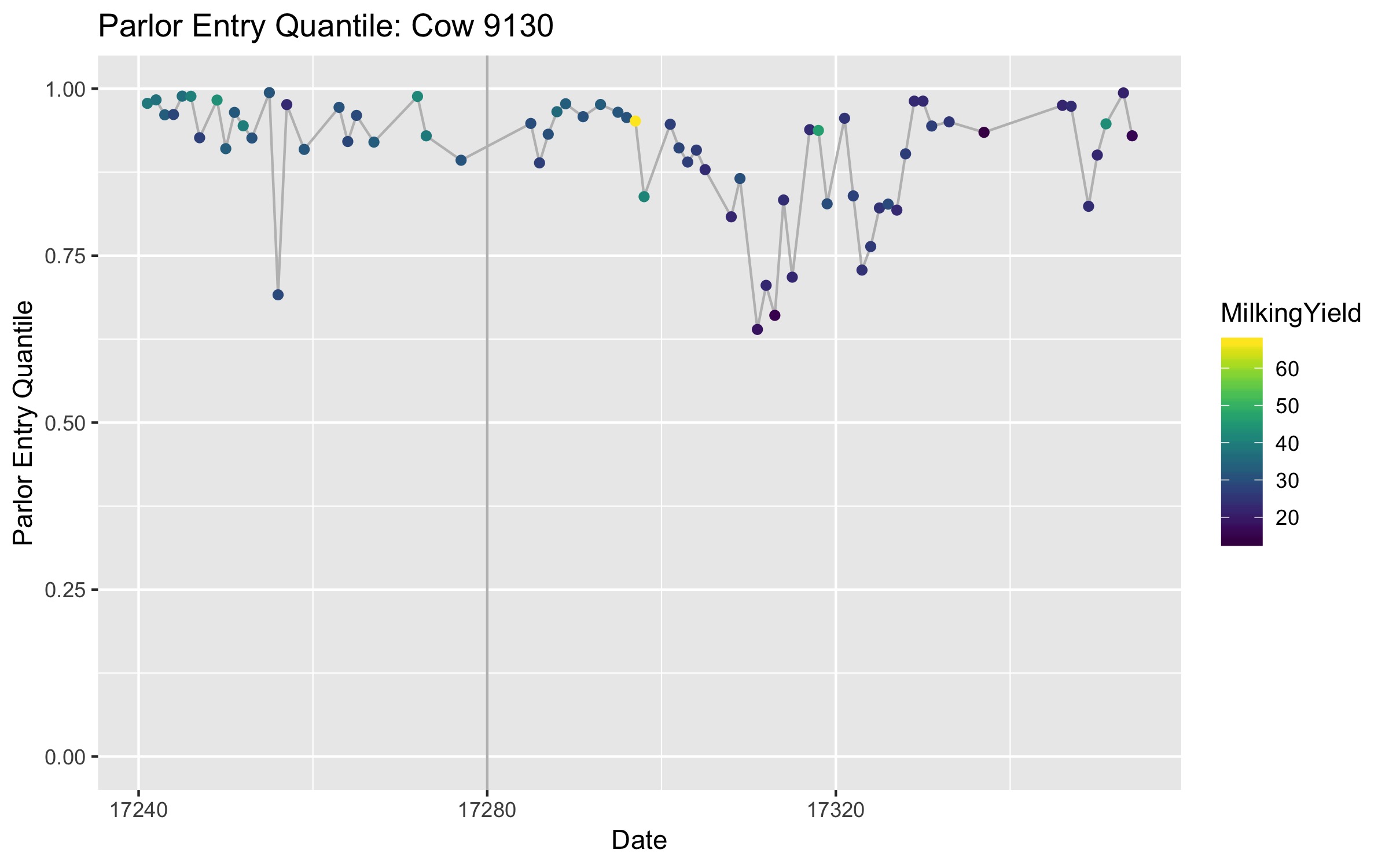

Supplement: Supplementary file 2 [file Data_Sheet_2.ZIP › Milking Yield/Cow_9130.jpg]

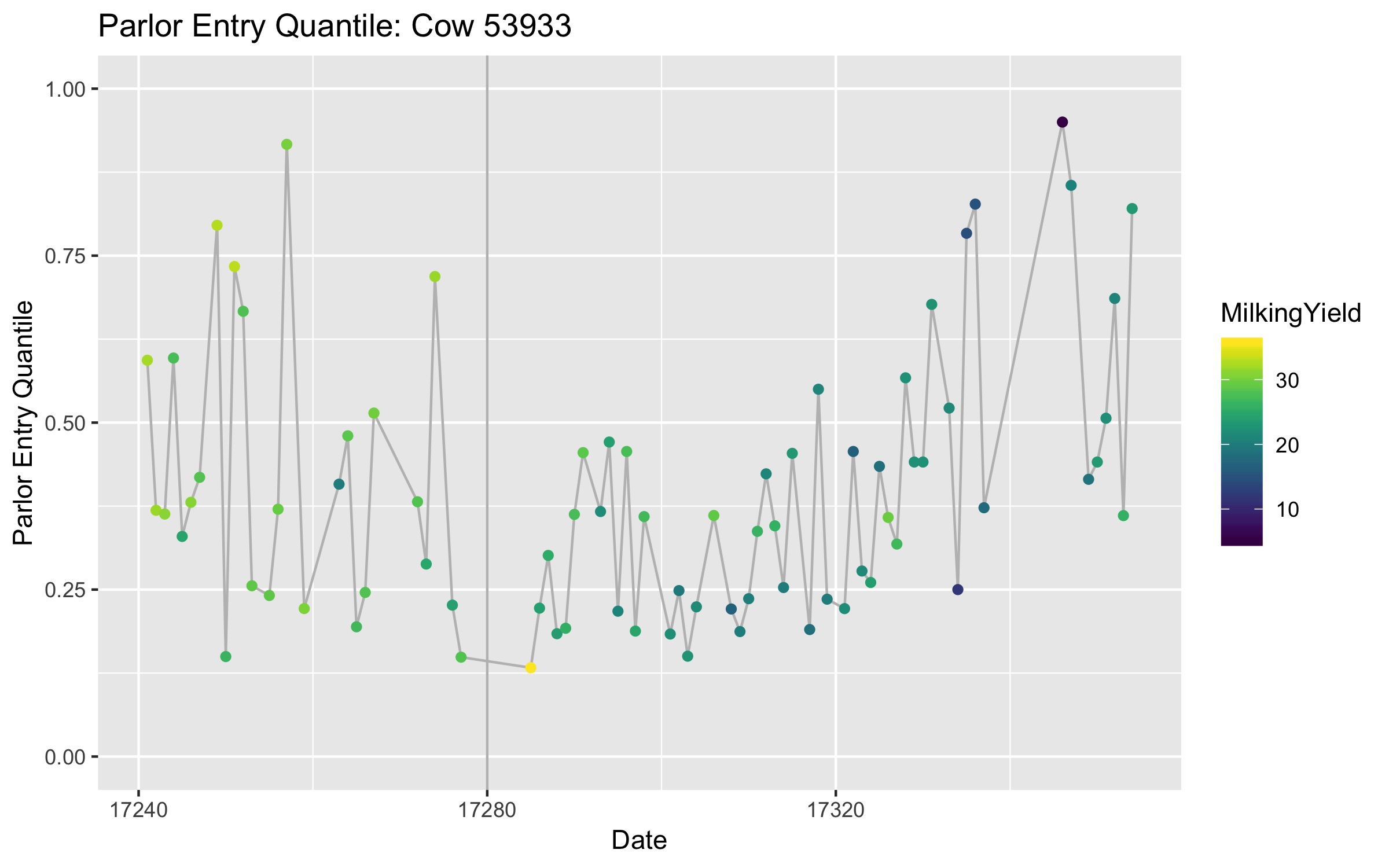

Supplement: Supplementary file 2 [file Data_Sheet_2.ZIP › Milking Yield/Cow_53933.jpg]

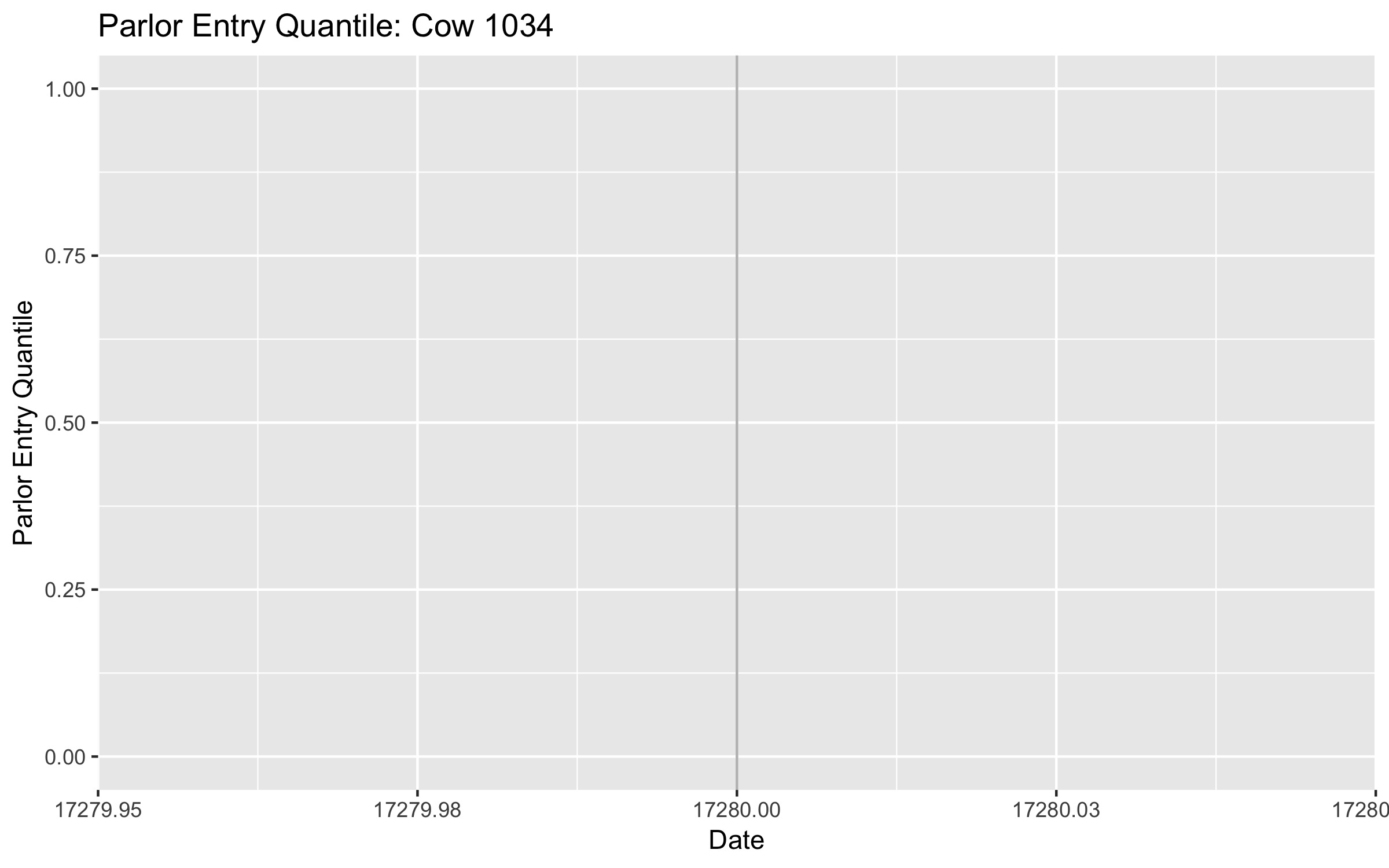

Supplement: Supplementary file 2 [file Data_Sheet_2.ZIP › Milking Yield/Cow_1034.jpg]

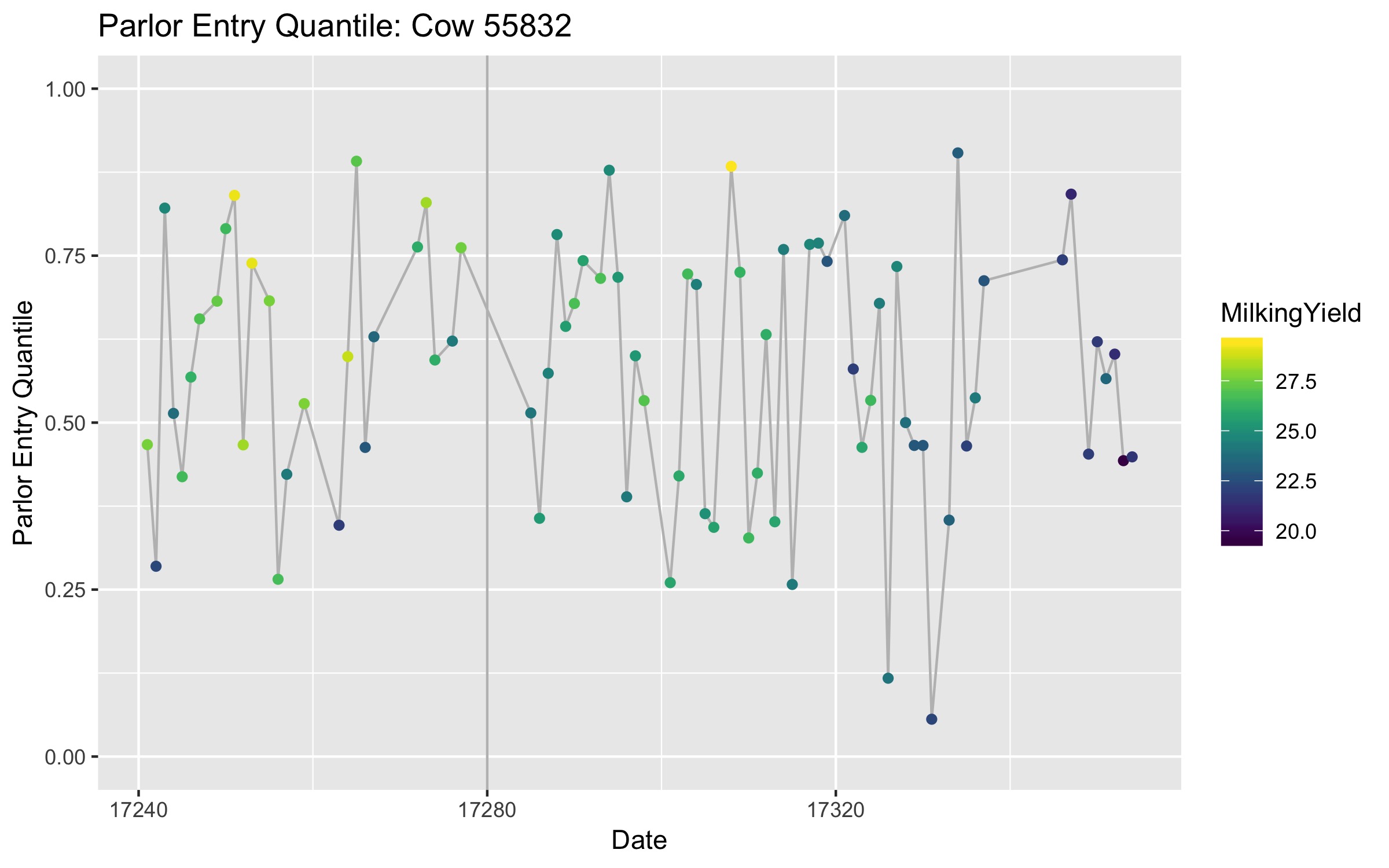

Supplement: Supplementary file 2 [file Data_Sheet_2.ZIP › Milking Yield/Cow_55832.jpg]

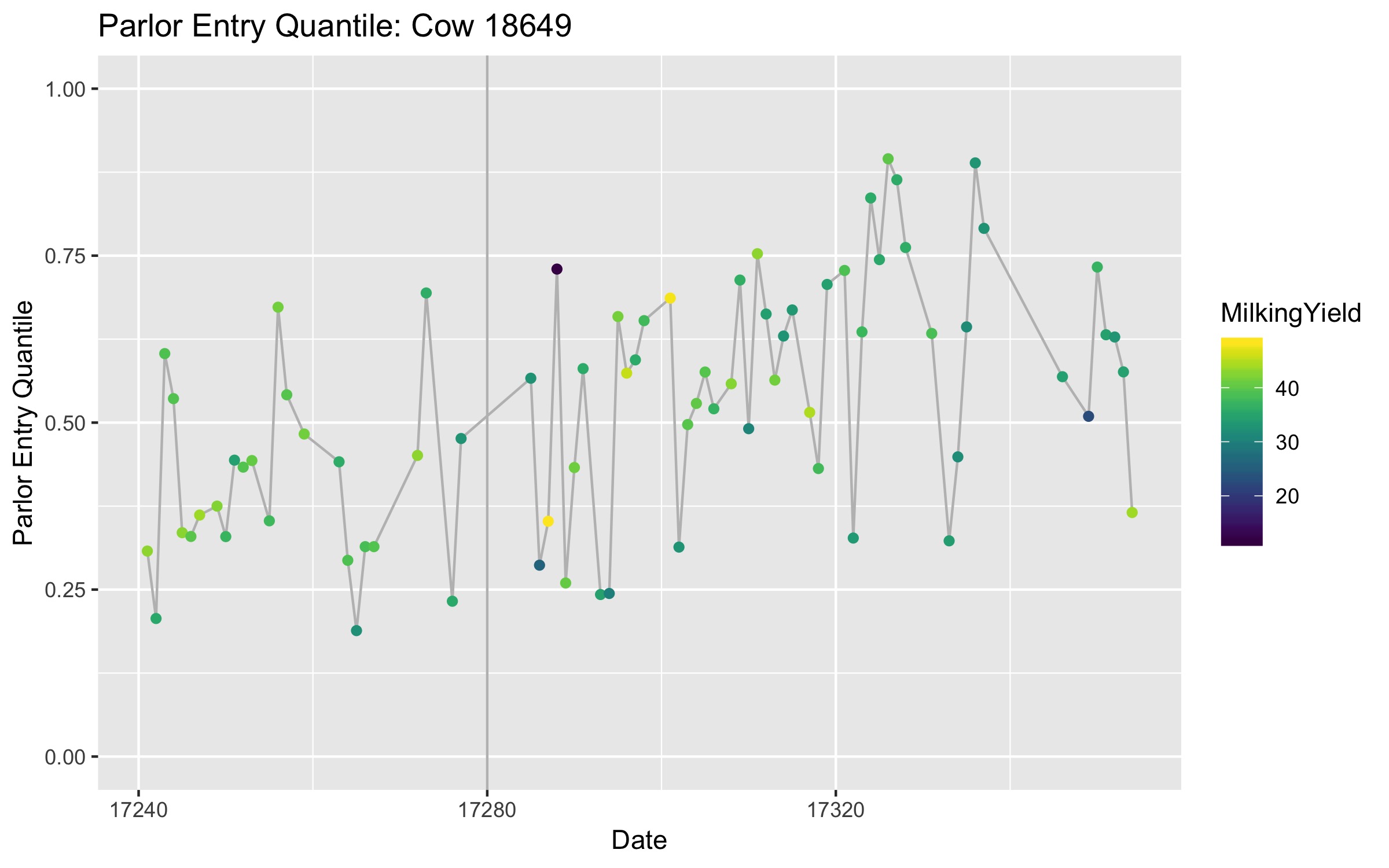

Supplement: Supplementary file 2 [file Data_Sheet_2.ZIP › Milking Yield/Cow_18649.jpg]

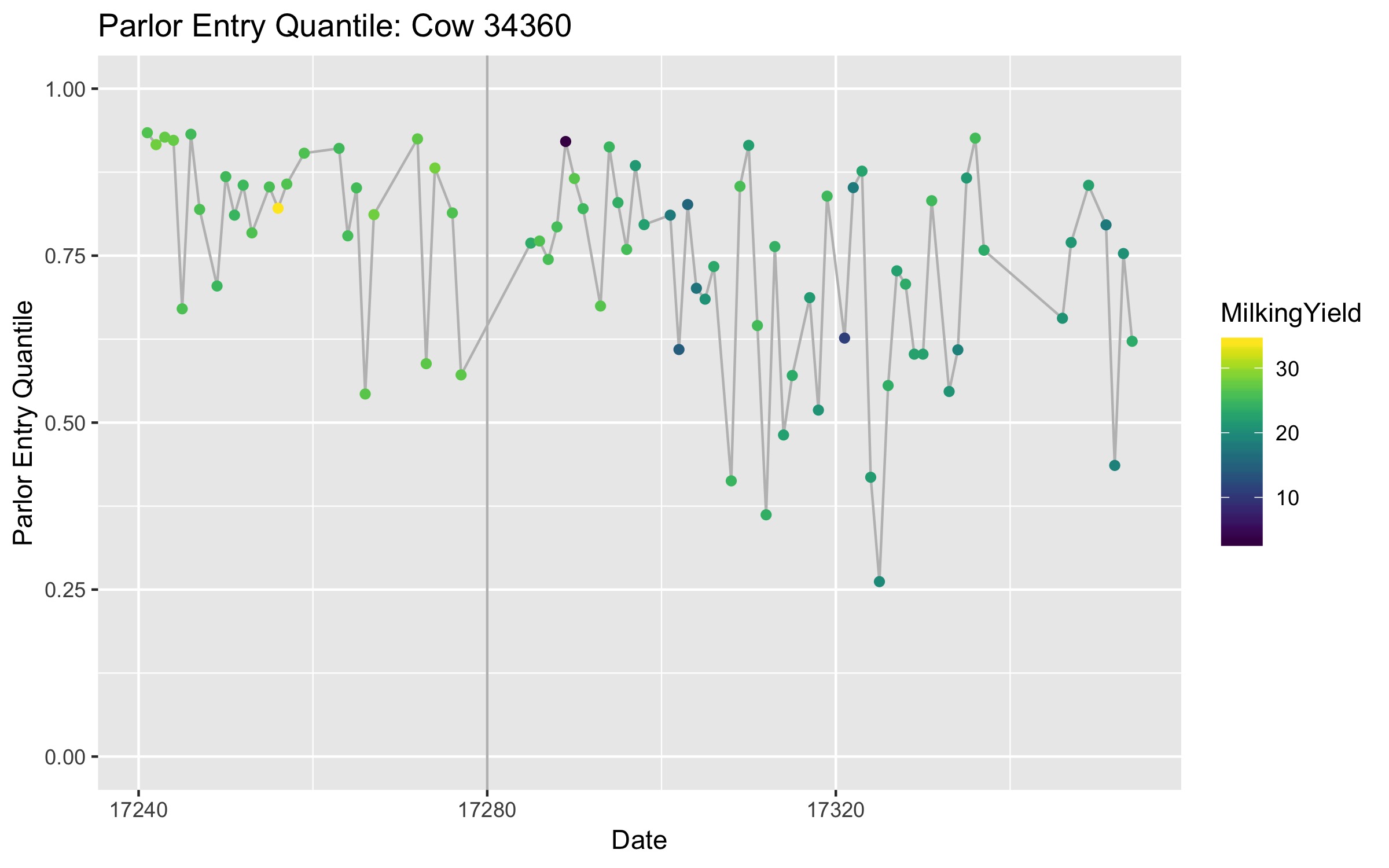

Supplement: Supplementary file 2 [file Data_Sheet_2.ZIP › Milking Yield/Cow_34360.jpg]

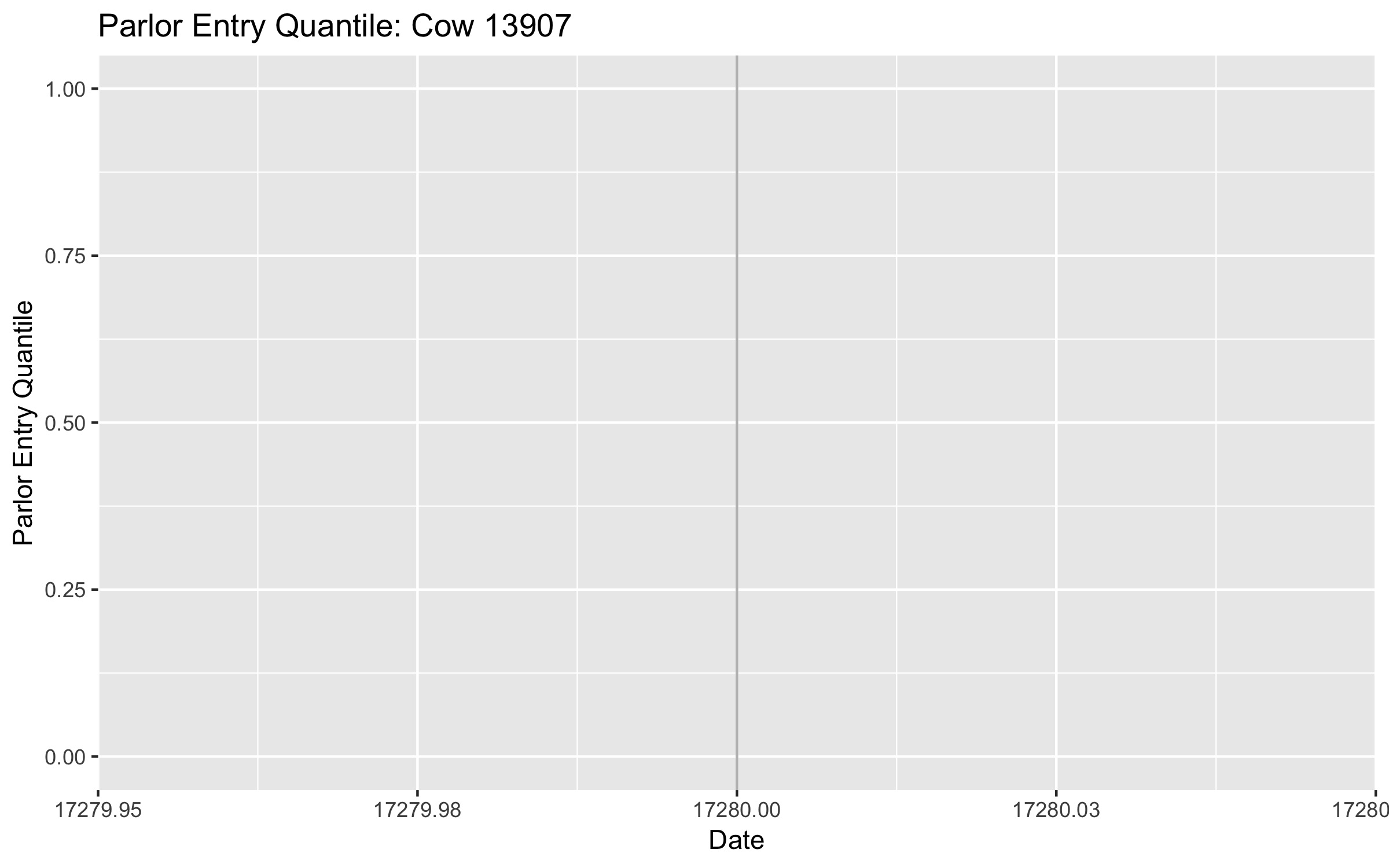

Supplement: Supplementary file 2 [file Data_Sheet_2.ZIP › Milking Yield/Cow_13907.jpg]

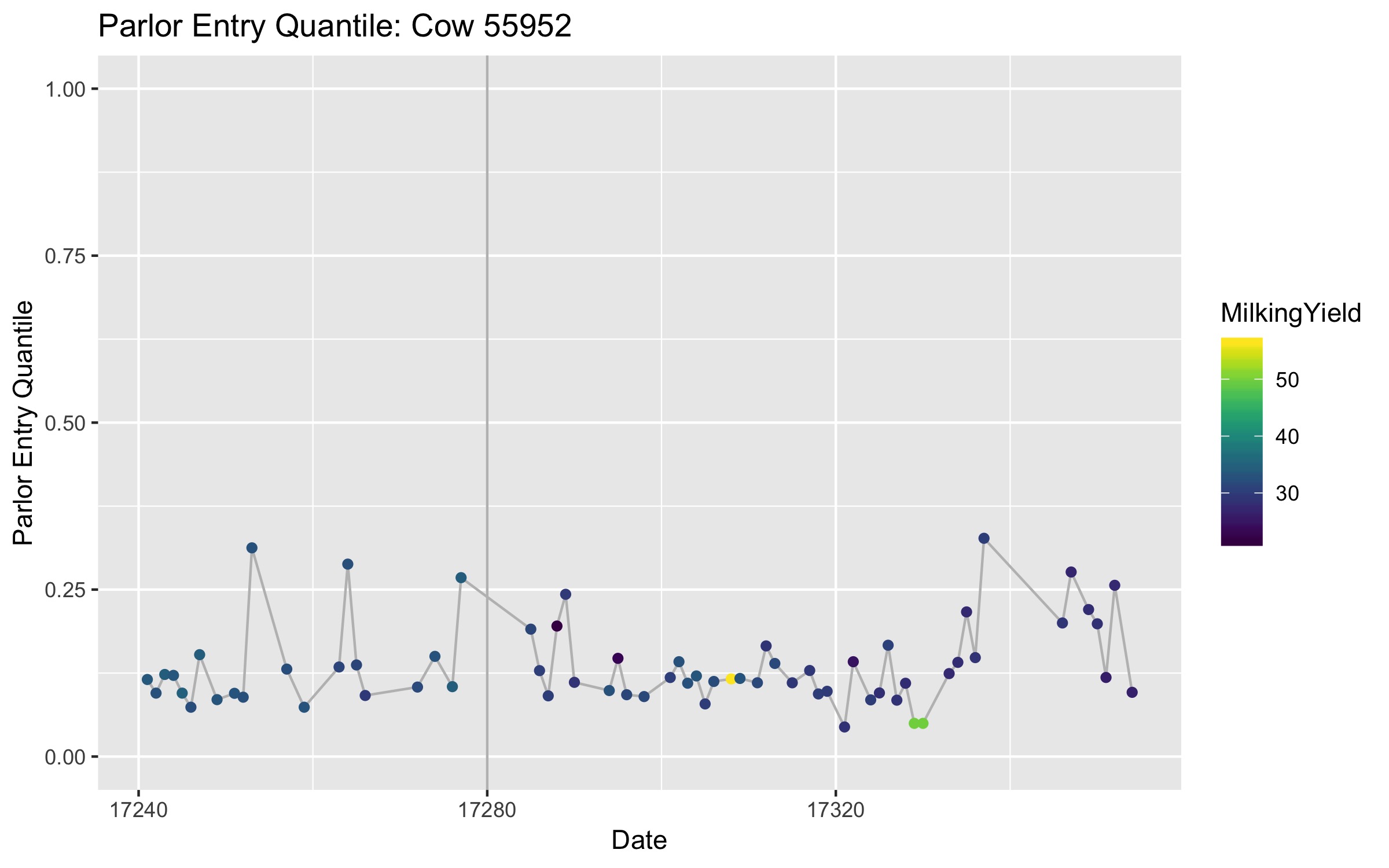

Supplement: Supplementary file 2 [file Data_Sheet_2.ZIP › Milking Yield/Cow_55952.jpg]

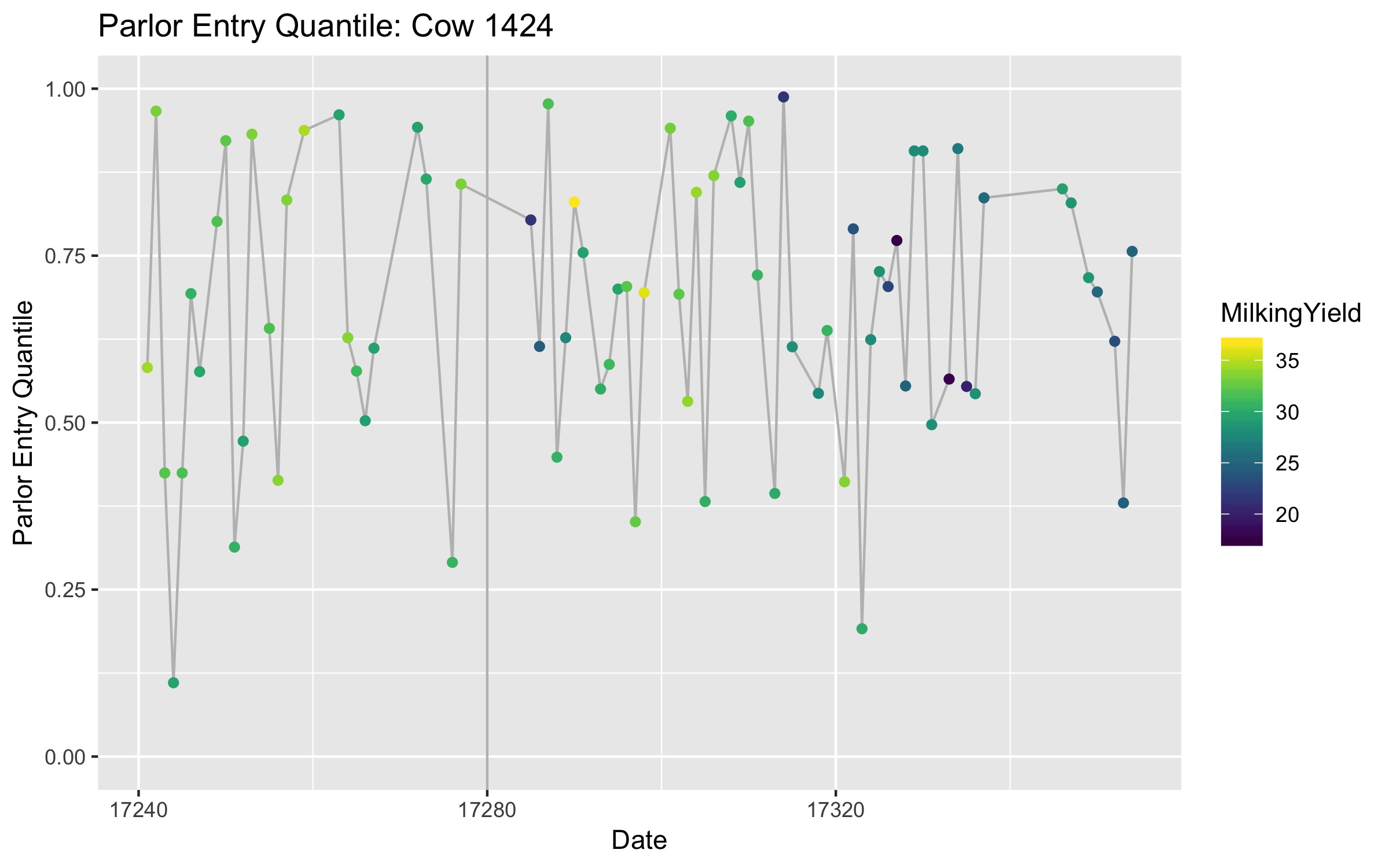

Supplement: Supplementary file 2 [file Data_Sheet_2.ZIP › Milking Yield/Cow_1424.jpg]

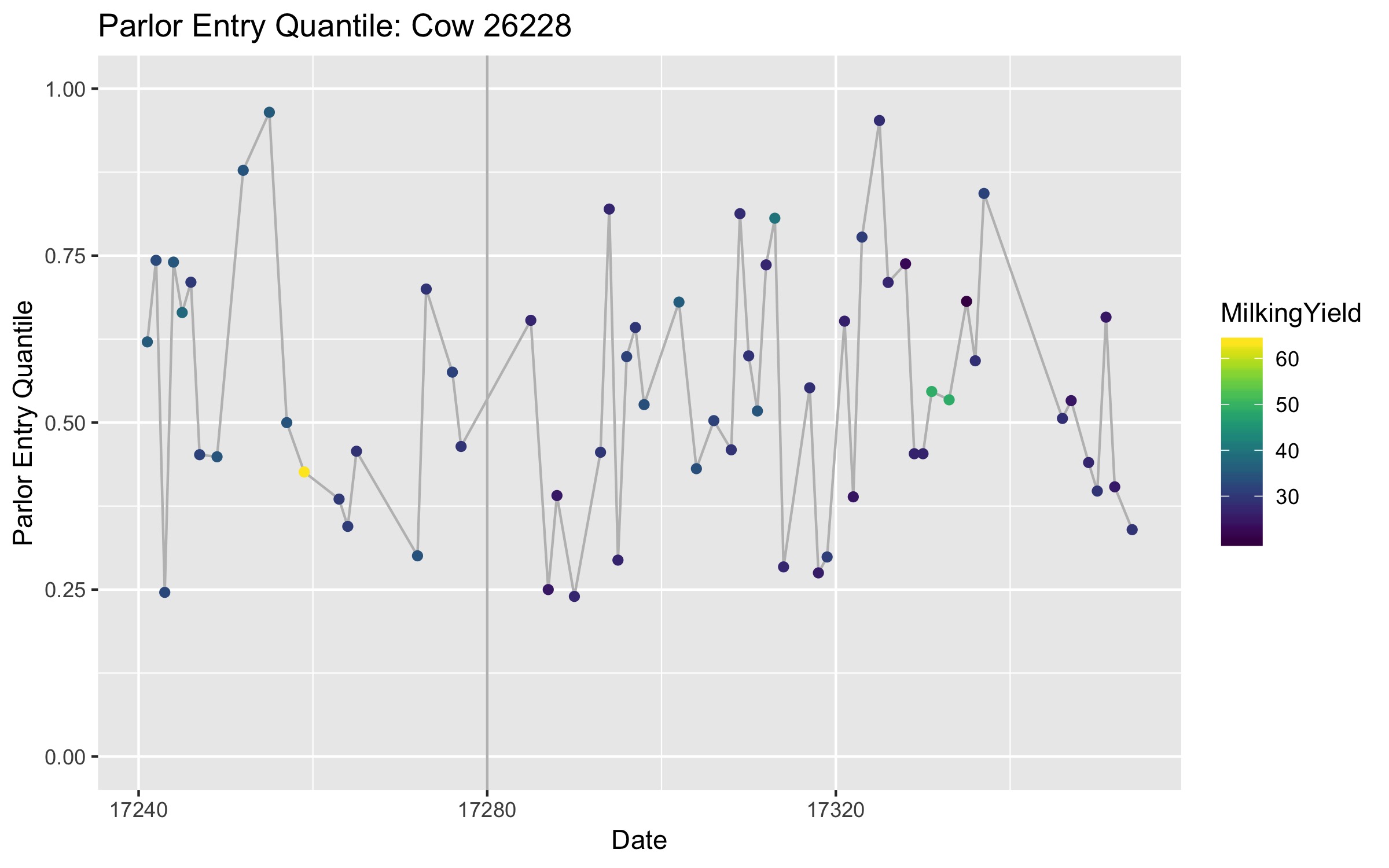

Supplement: Supplementary file 2 [file Data_Sheet_2.ZIP › Milking Yield/Cow_26228.jpg]

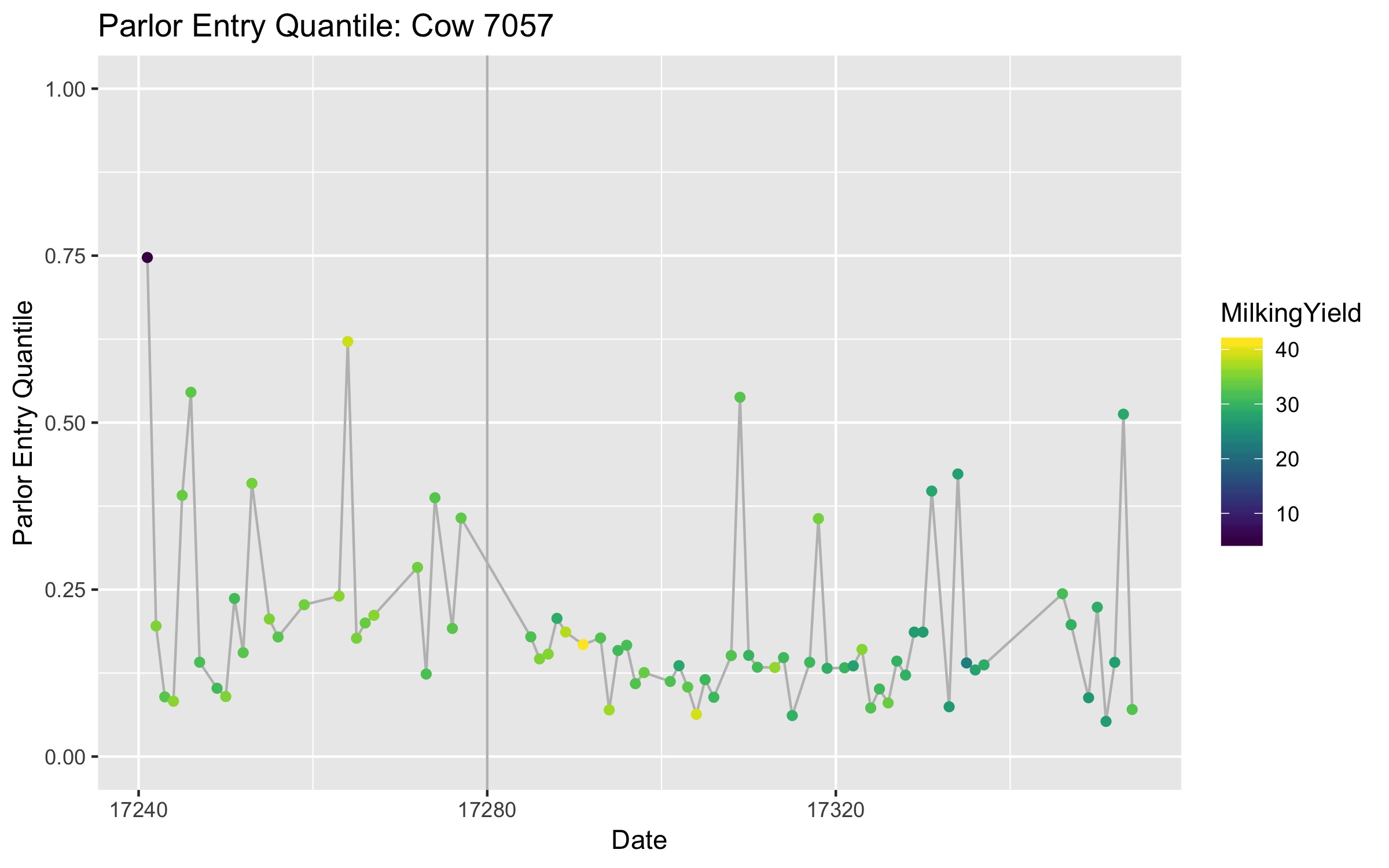

Supplement: Supplementary file 2 [file Data_Sheet_2.ZIP › Milking Yield/Cow_7057.jpg]

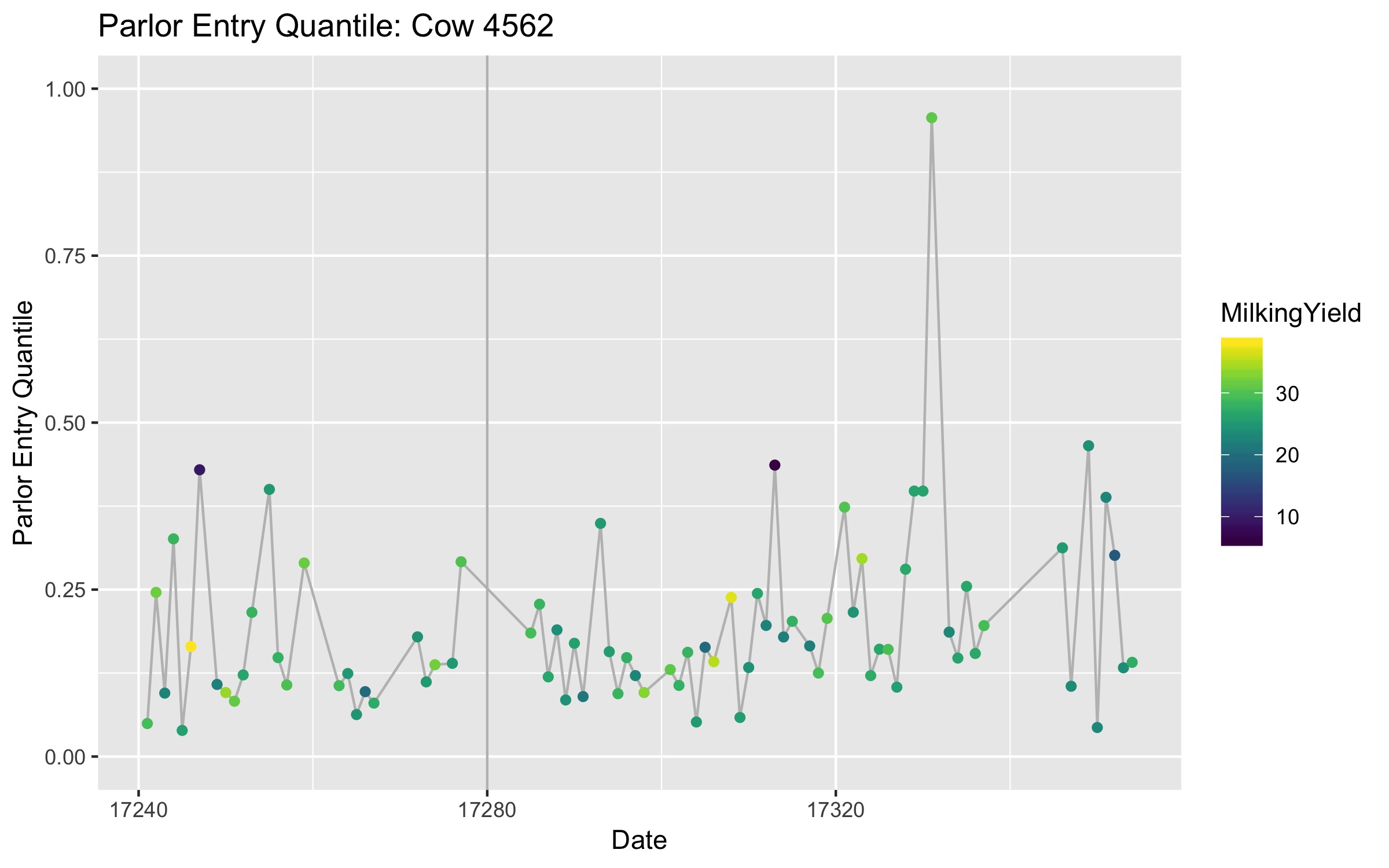

Supplement: Supplementary file 2 [file Data_Sheet_2.ZIP › Milking Yield/Cow_4562.jpg]

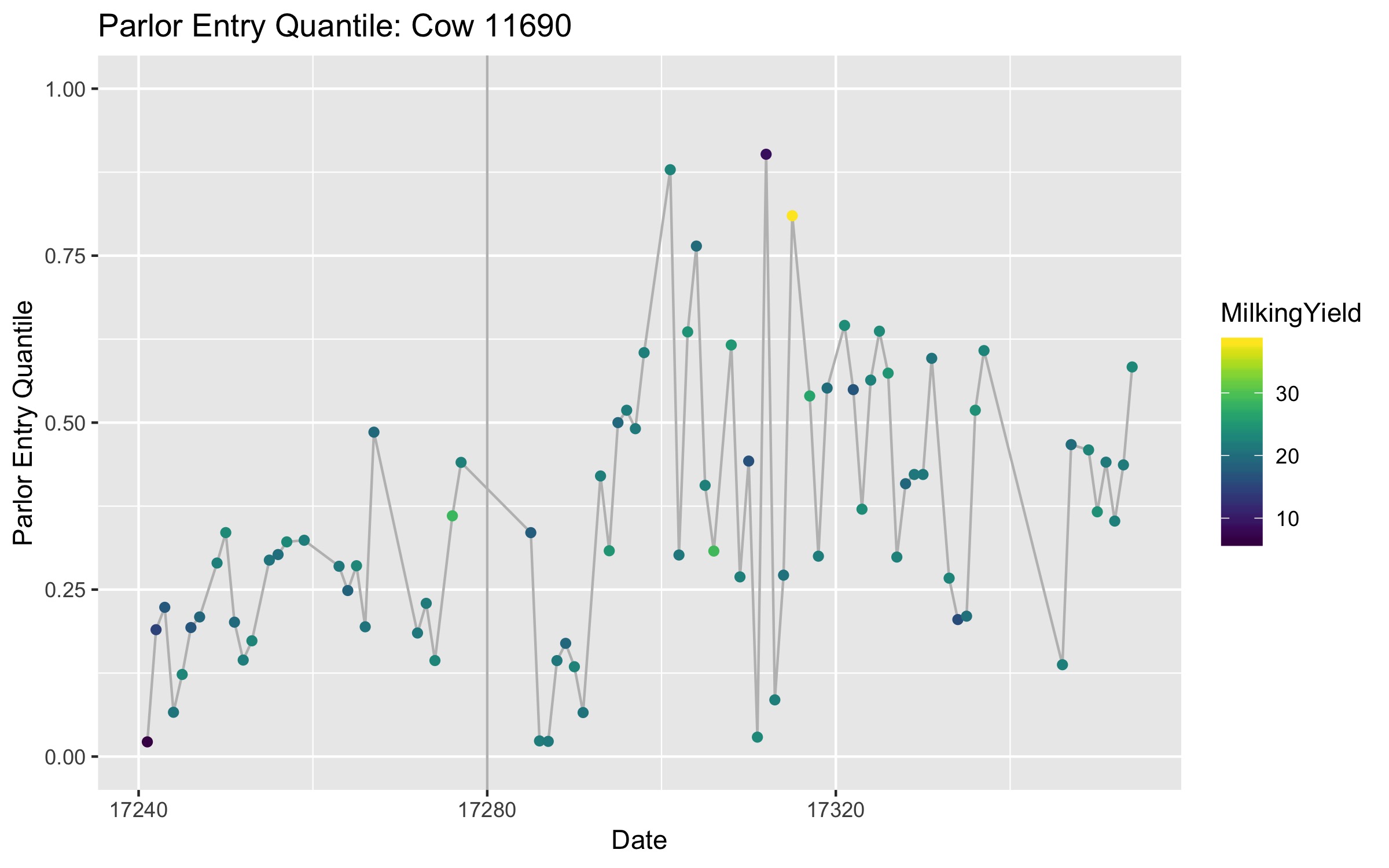

Supplement: Supplementary file 2 [file Data_Sheet_2.ZIP › Milking Yield/Cow_11690.jpg]

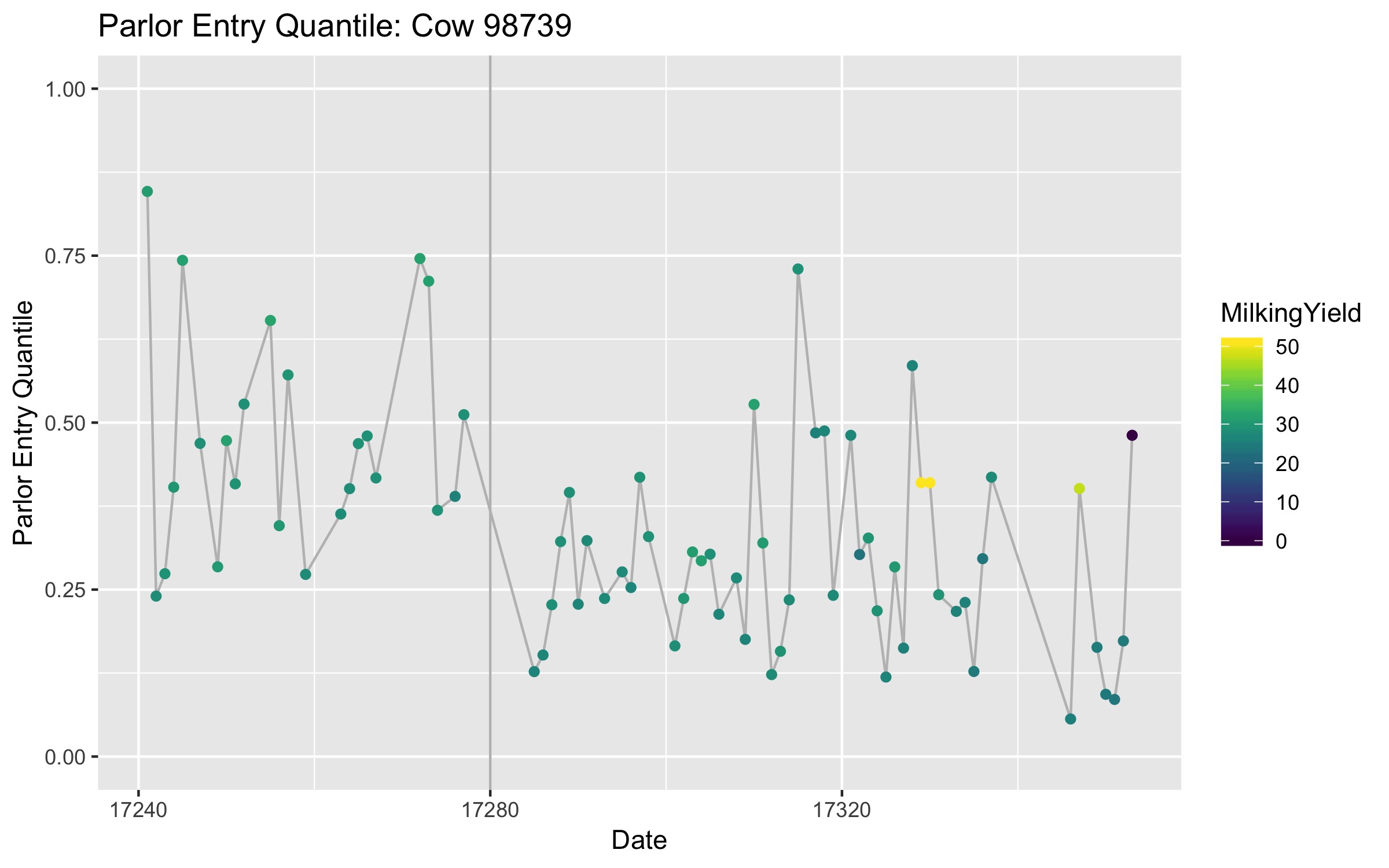

Supplement: Supplementary file 2 [file Data_Sheet_2.ZIP › Milking Yield/Cow_98739.jpg]

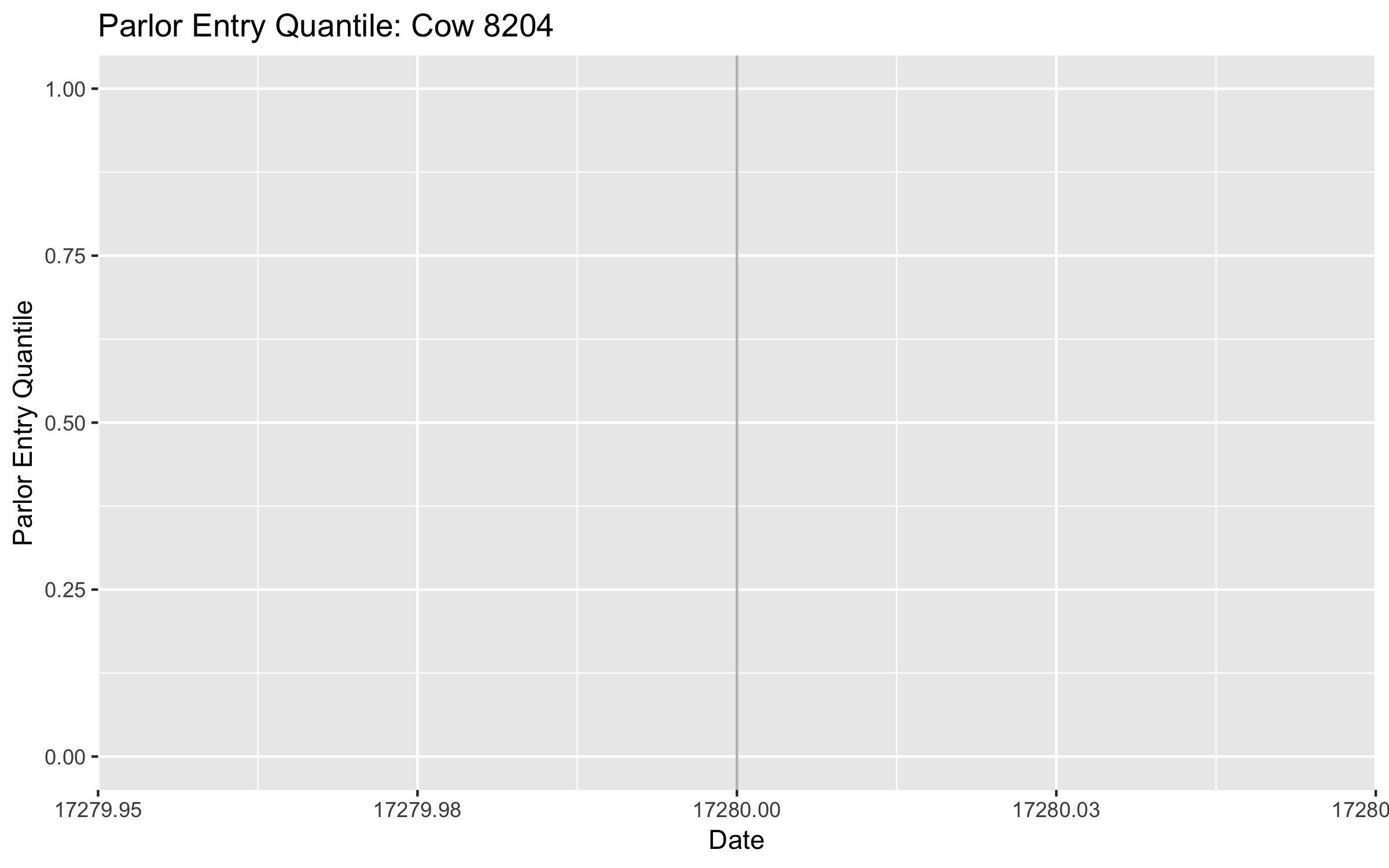

Supplement: Supplementary file 2 [file Data_Sheet_2.ZIP › Milking Yield/Cow_8204.jpg]

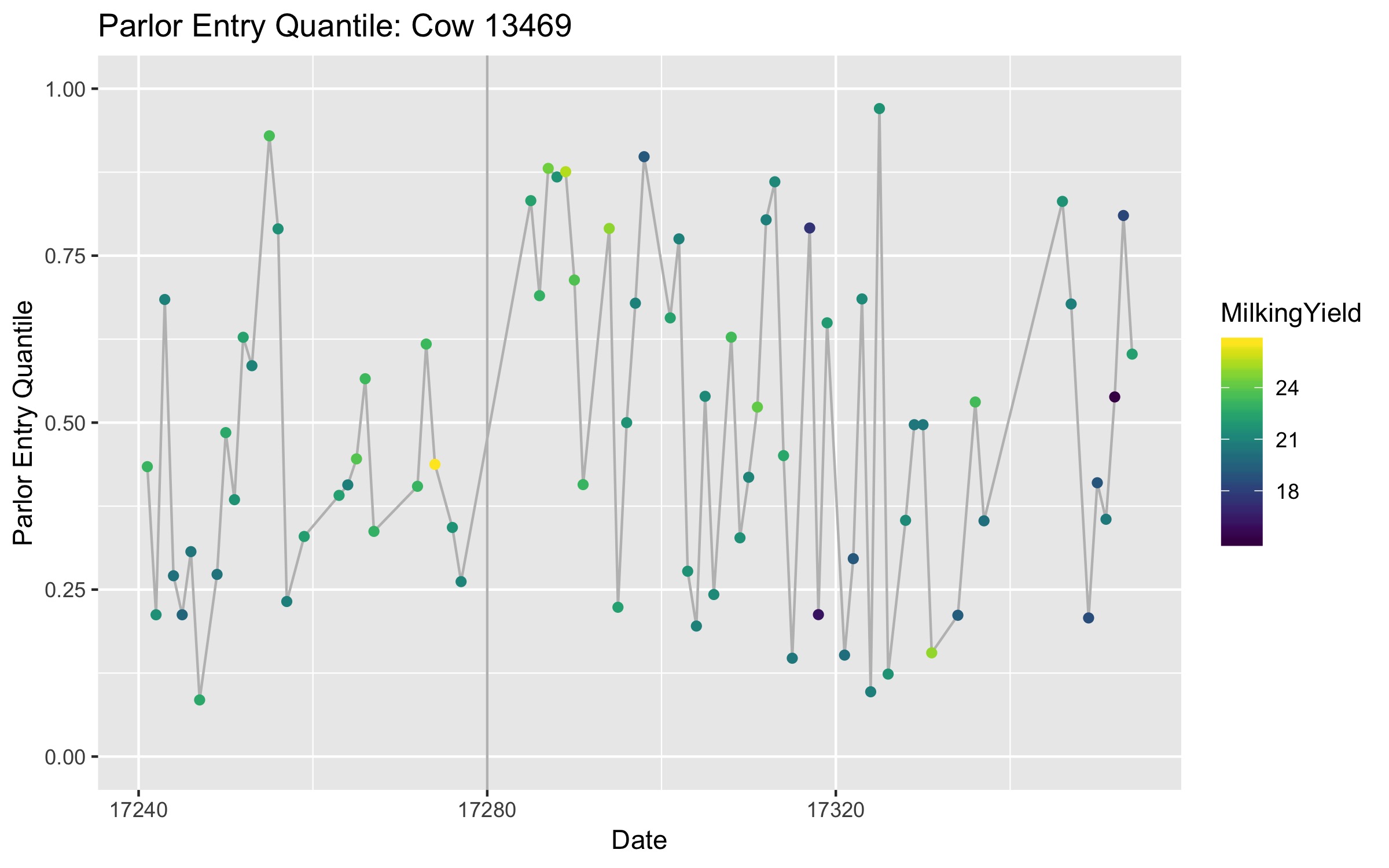

Supplement: Supplementary file 2 [file Data_Sheet_2.ZIP › Milking Yield/Cow_13469.jpg]

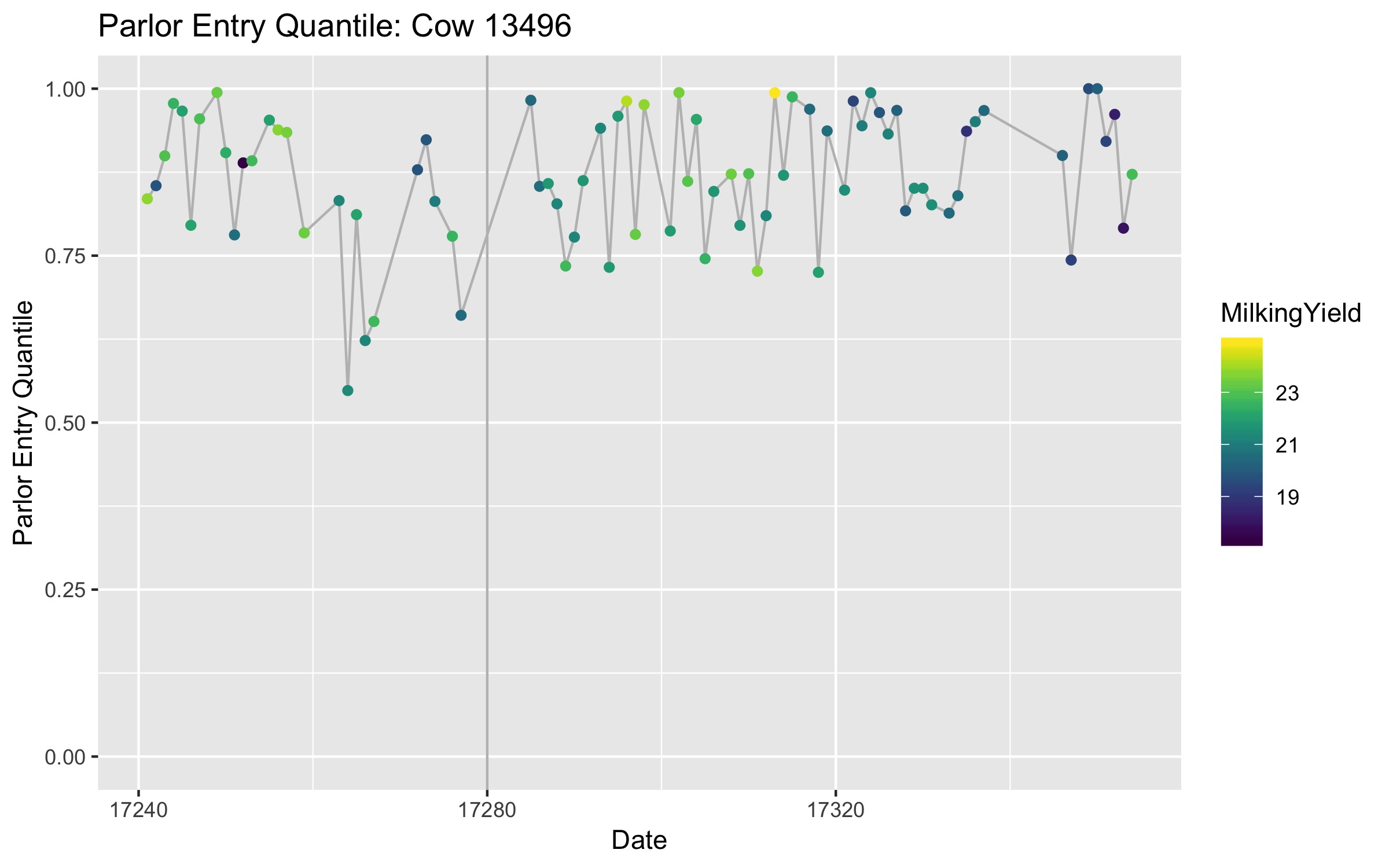

Supplement: Supplementary file 2 [file Data_Sheet_2.ZIP › Milking Yield/Cow_13496.jpg]

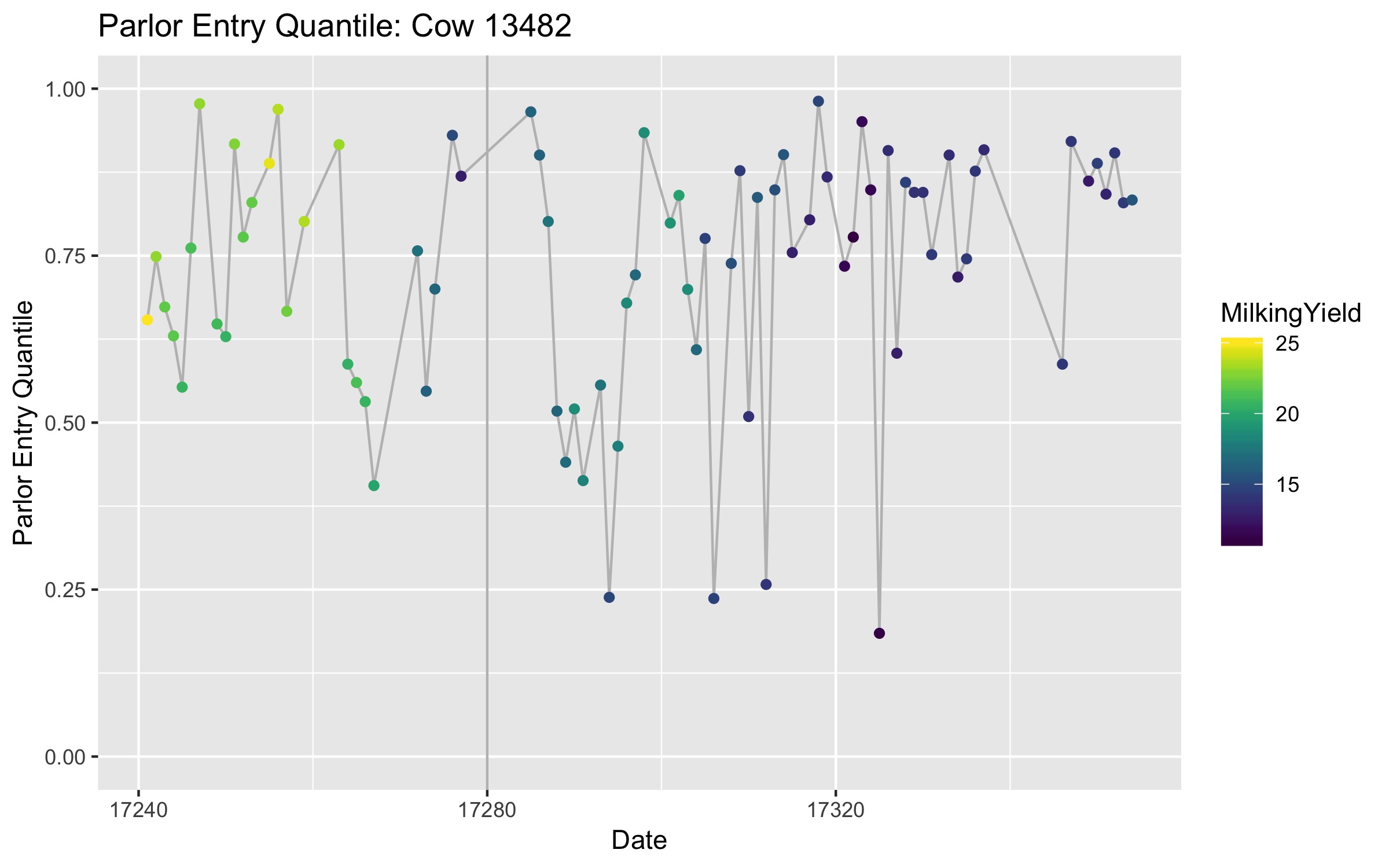

Supplement: Supplementary file 2 [file Data_Sheet_2.ZIP › Milking Yield/Cow_13482.jpg]

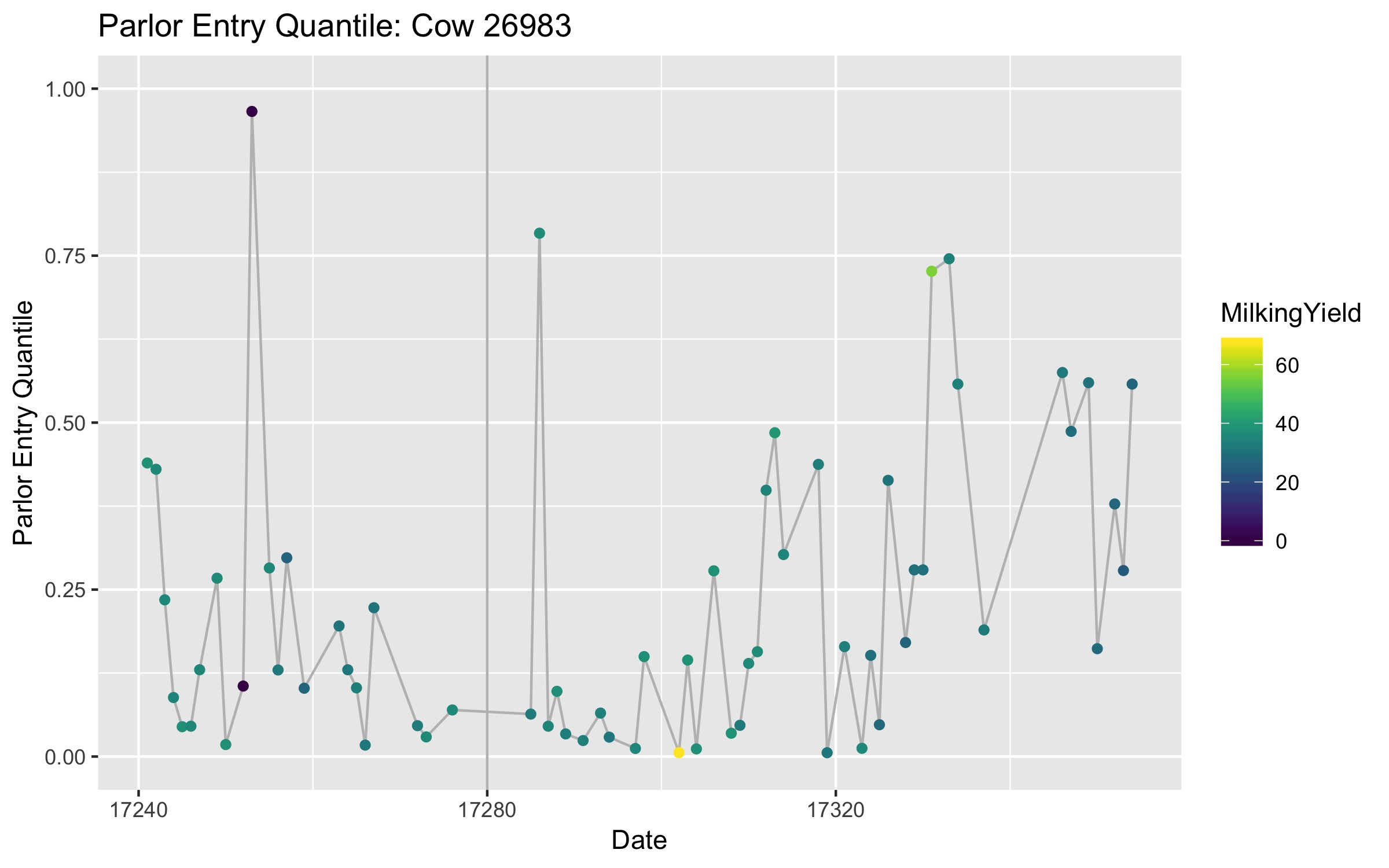

Supplement: Supplementary file 2 [file Data_Sheet_2.ZIP › Milking Yield/Cow_26983.jpg]

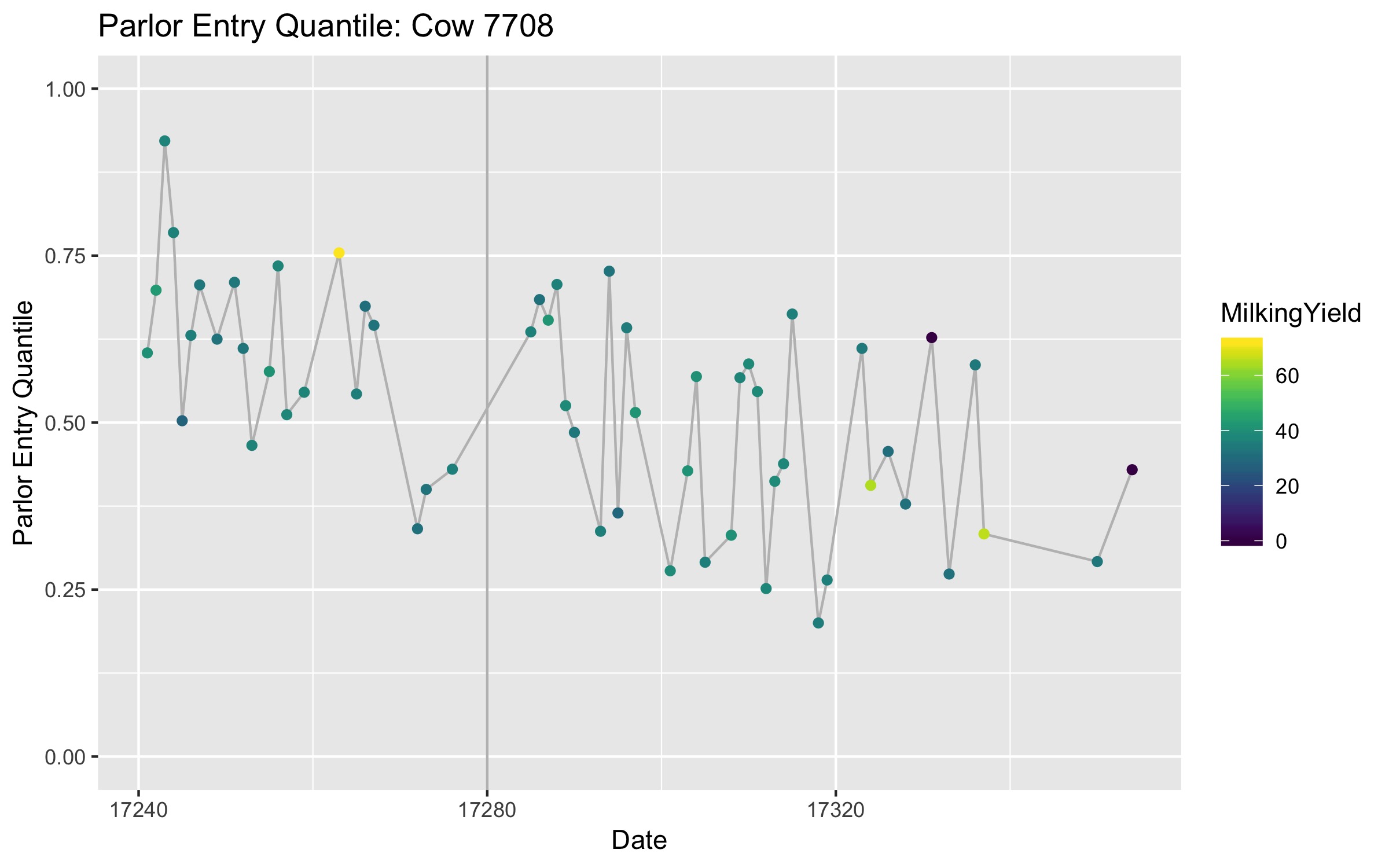

Supplement: Supplementary file 2 [file Data_Sheet_2.ZIP › Milking Yield/Cow_7708.jpg]

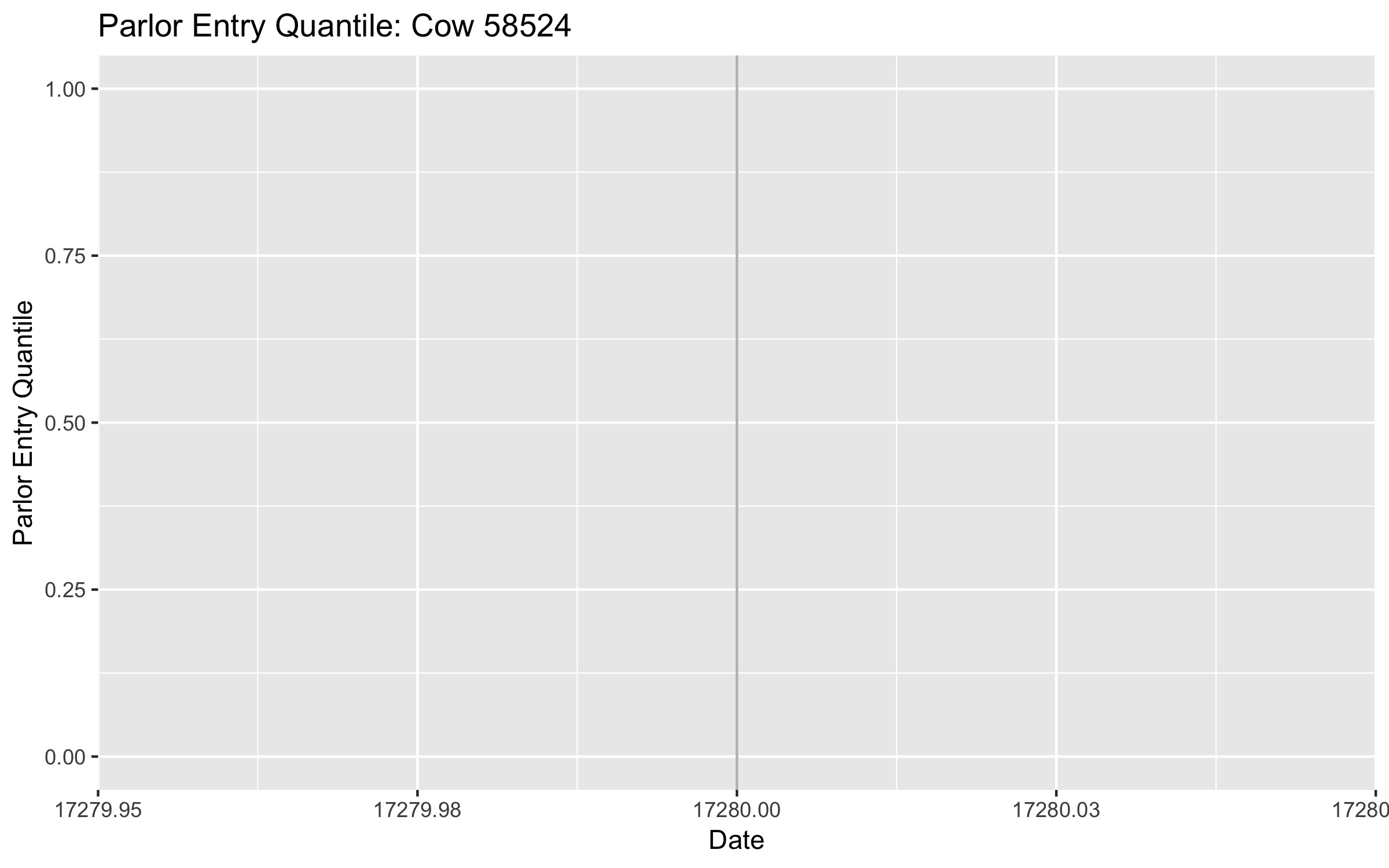

Supplement: Supplementary file 2 [file Data_Sheet_2.ZIP › Milking Yield/Cow_58524.jpg]

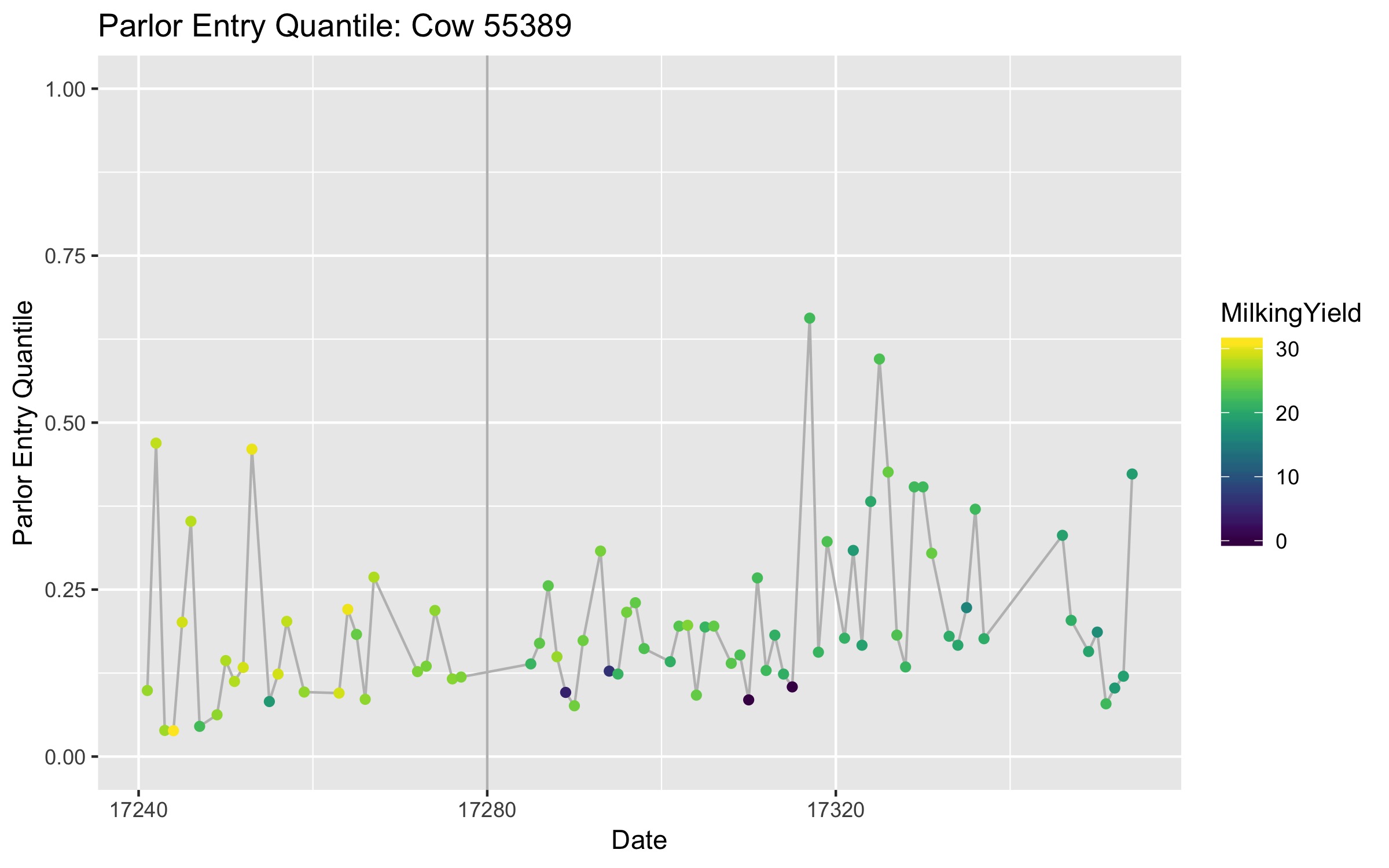

Supplement: Supplementary file 2 [file Data_Sheet_2.ZIP › Milking Yield/Cow_55389.jpg]

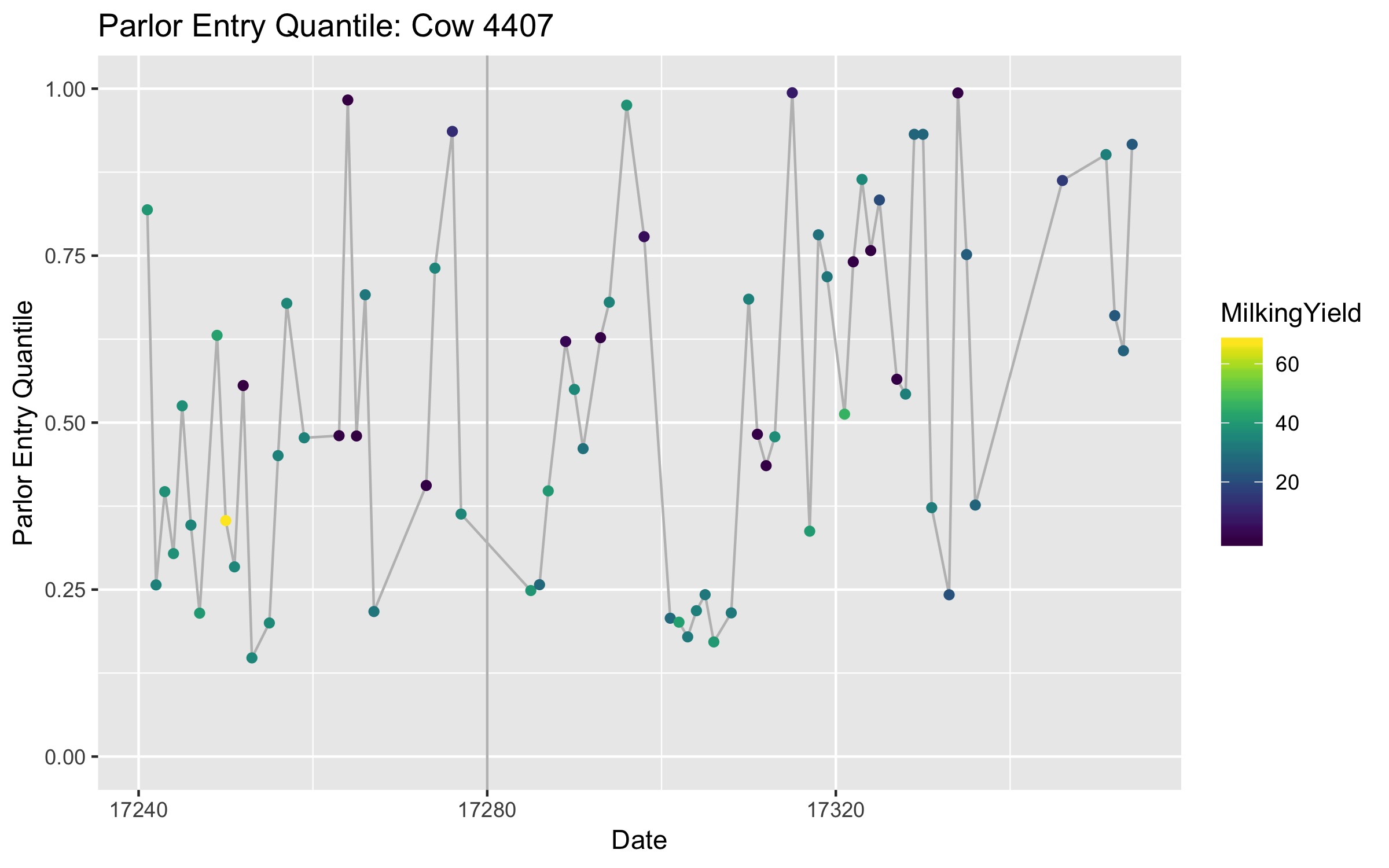

Supplement: Supplementary file 2 [file Data_Sheet_2.ZIP › Milking Yield/Cow_4407.jpg]

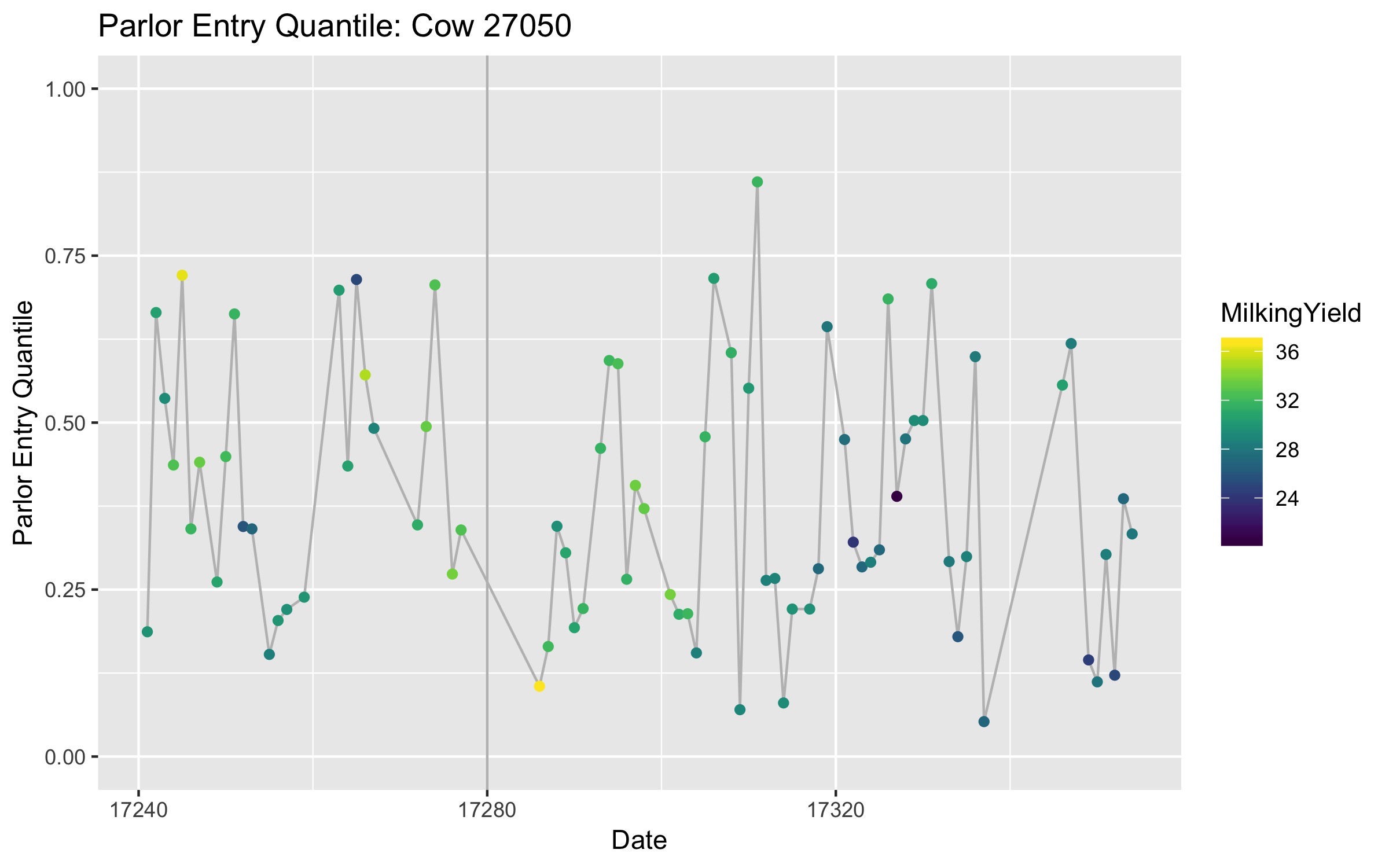

Supplement: Supplementary file 2 [file Data_Sheet_2.ZIP › Milking Yield/Cow_27050.jpg]

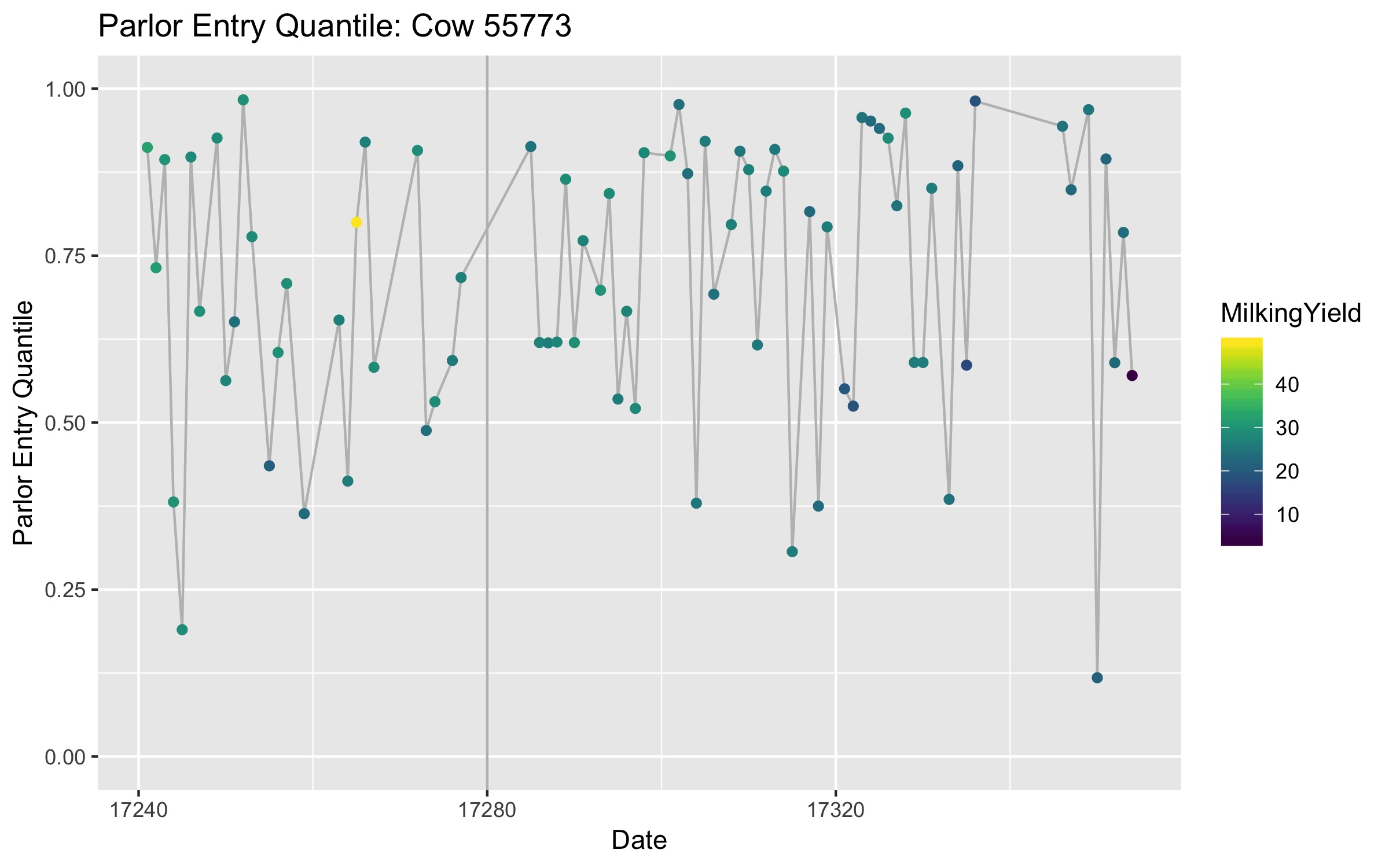

Supplement: Supplementary file 2 [file Data_Sheet_2.ZIP › Milking Yield/Cow_55773.jpg]

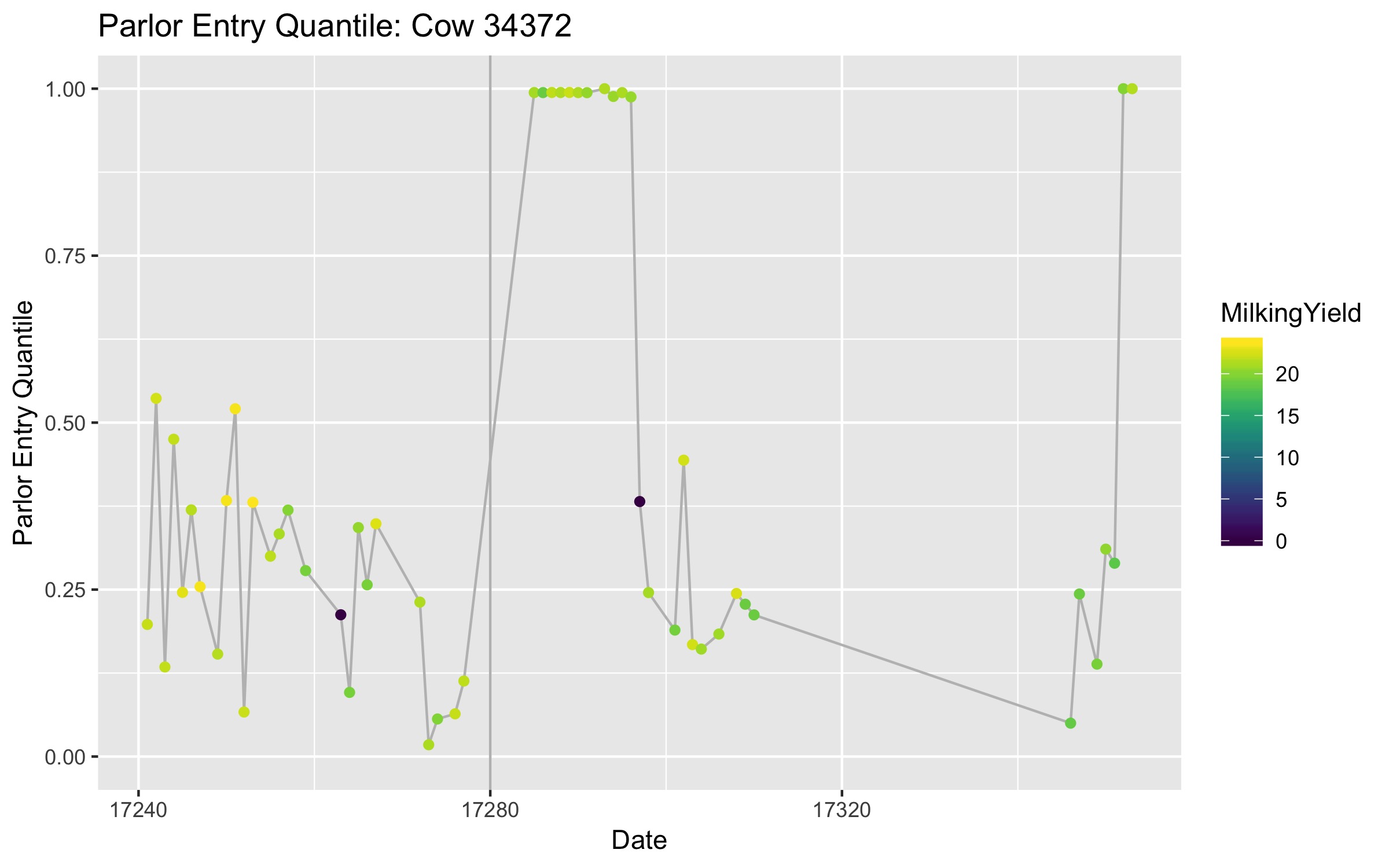

Supplement: Supplementary file 2 [file Data_Sheet_2.ZIP › Milking Yield/Cow_34372.jpg]

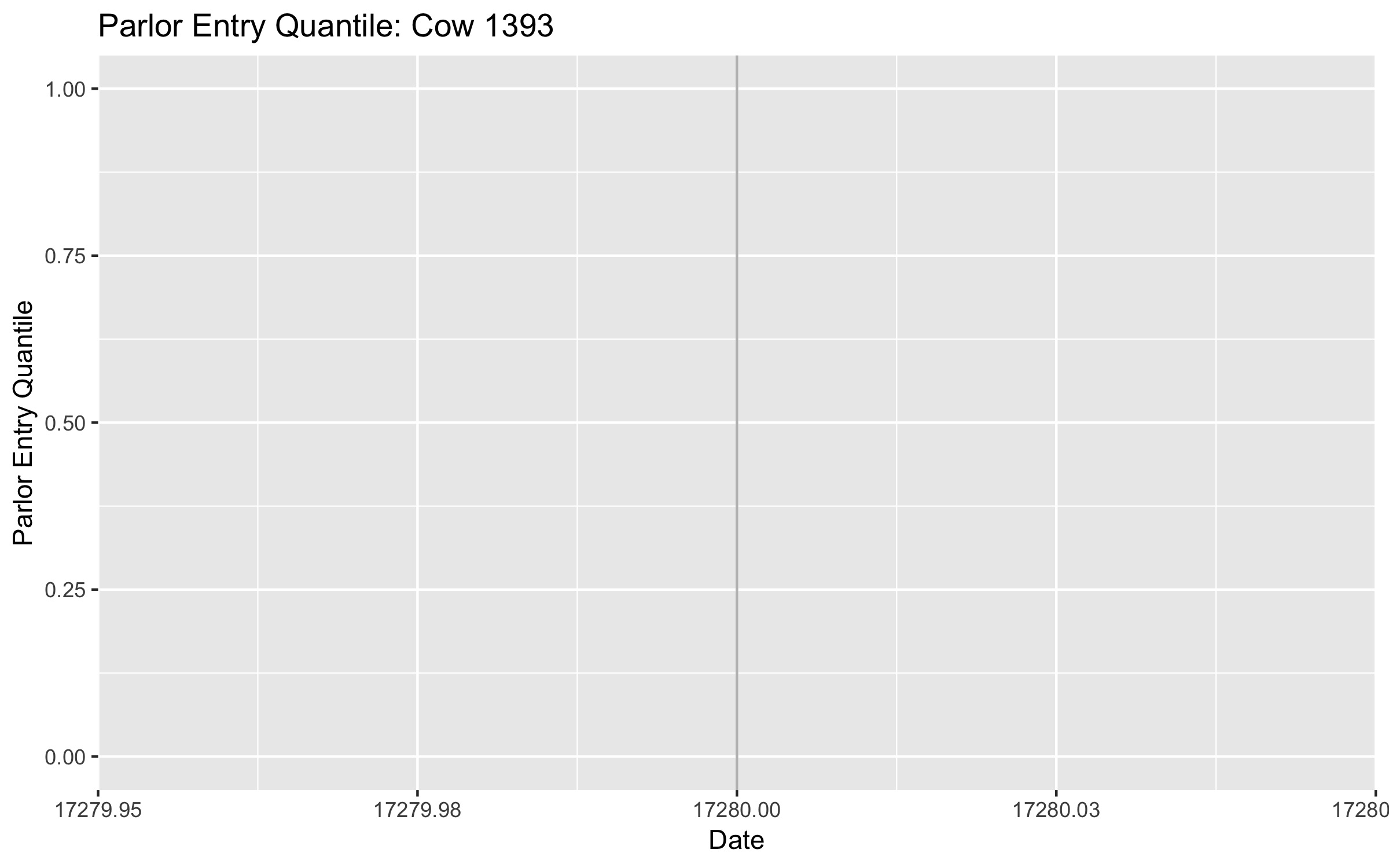

Supplement: Supplementary file 2 [file Data_Sheet_2.ZIP › Milking Yield/Cow_1393.jpg]

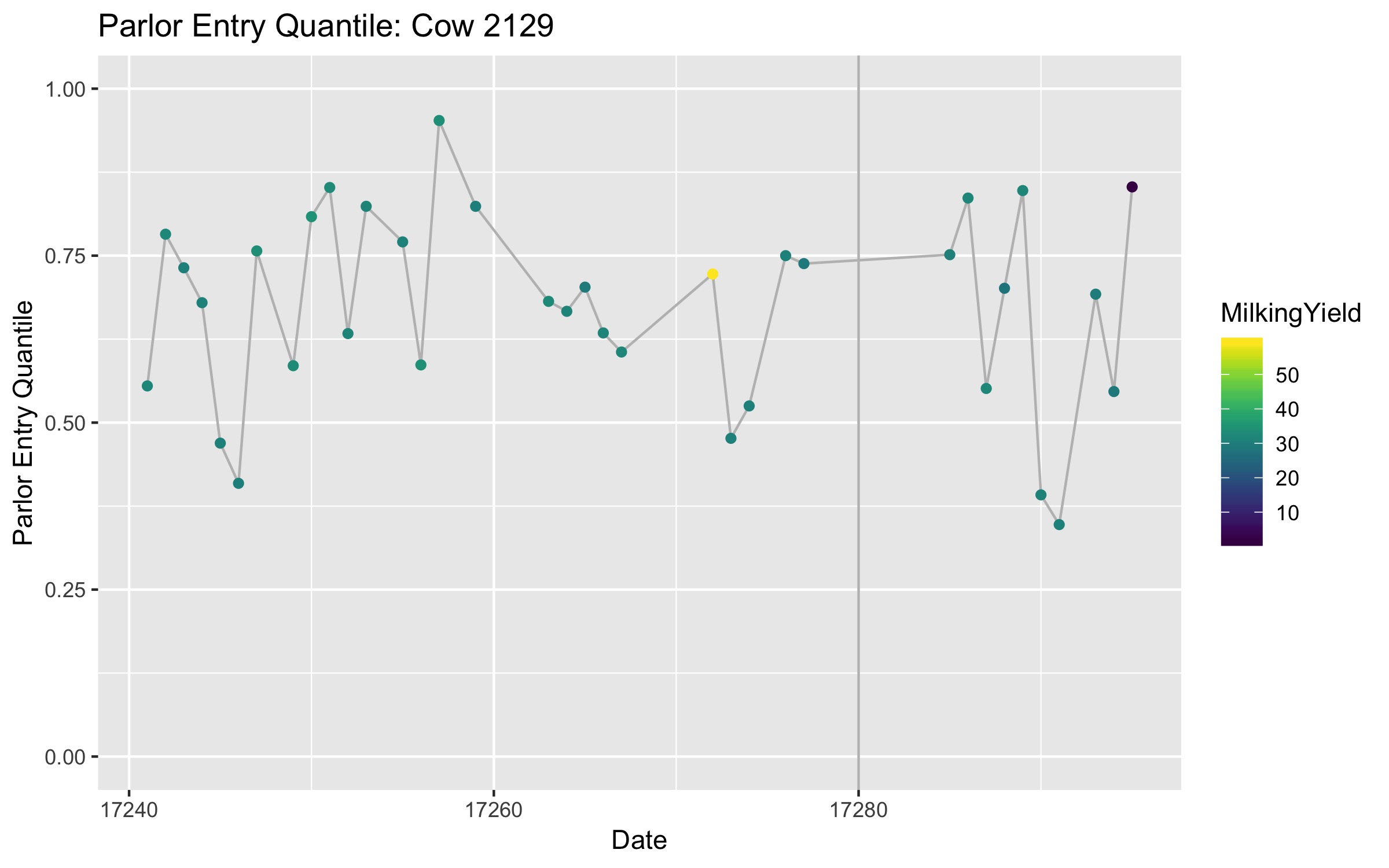

Supplement: Supplementary file 2 [file Data_Sheet_2.ZIP › Milking Yield/Cow_2129.jpg]

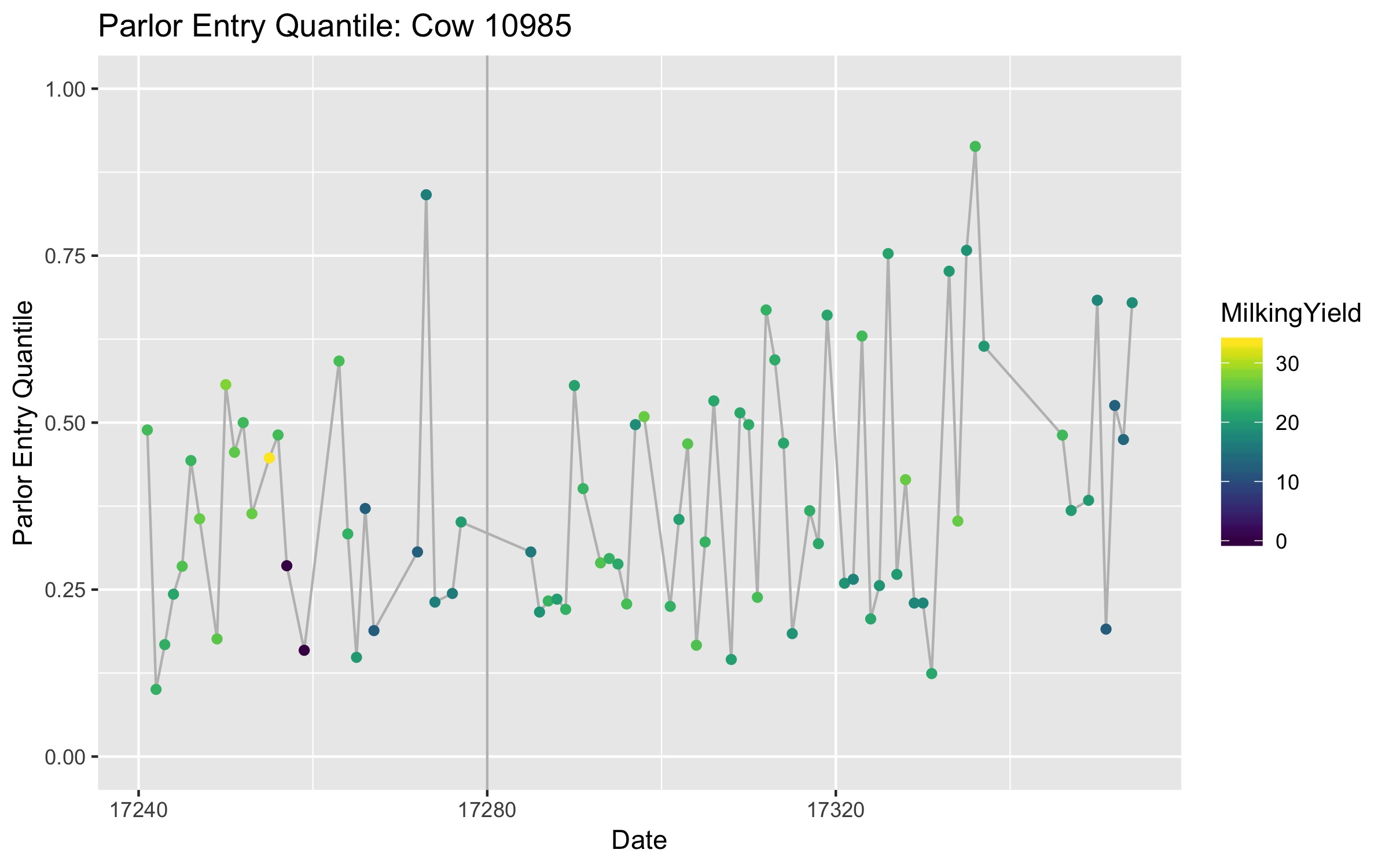

Supplement: Supplementary file 2 [file Data_Sheet_2.ZIP › Milking Yield/Cow_10985.jpg]

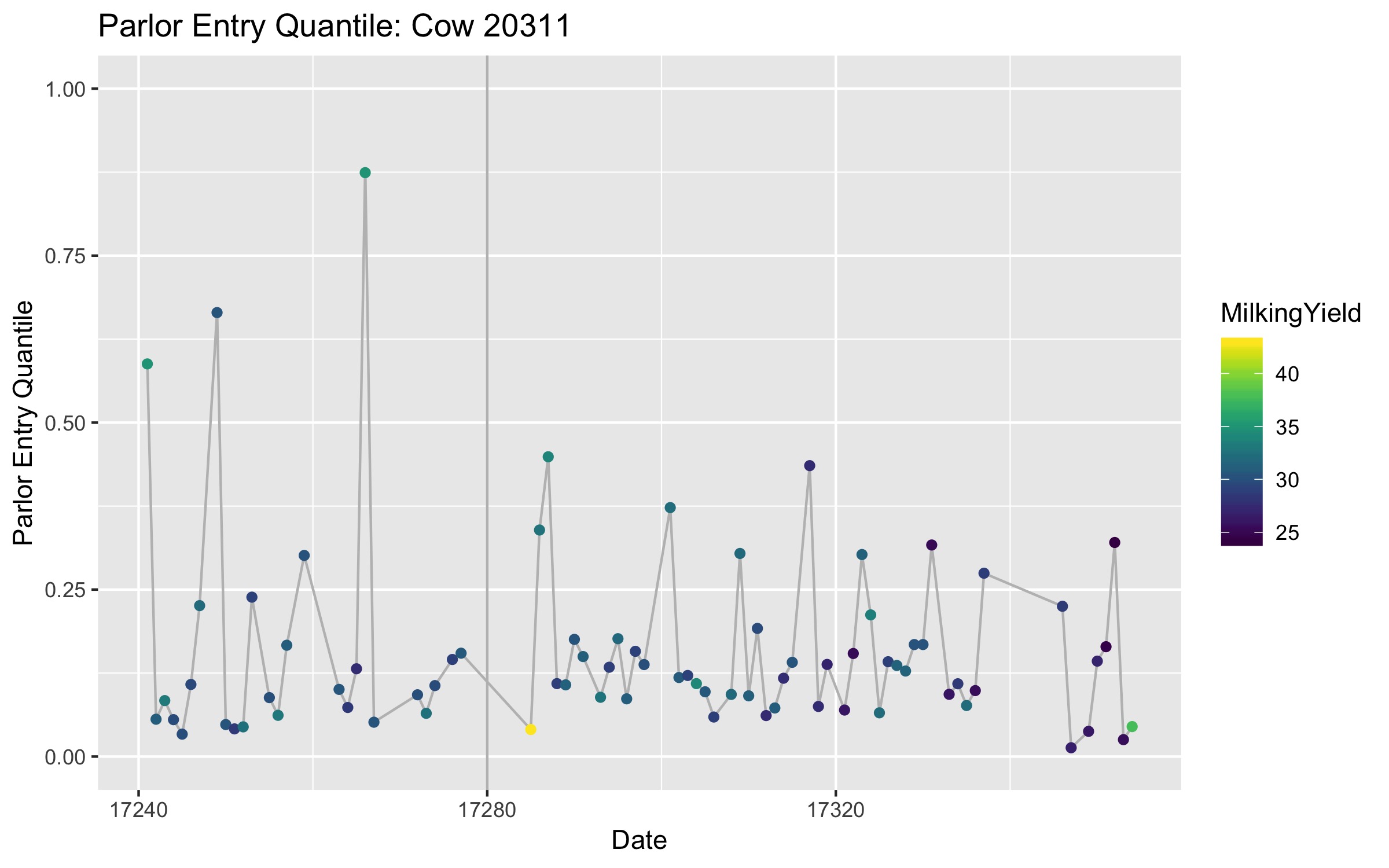

Supplement: Supplementary file 2 [file Data_Sheet_2.ZIP › Milking Yield/Cow_20311.jpg]

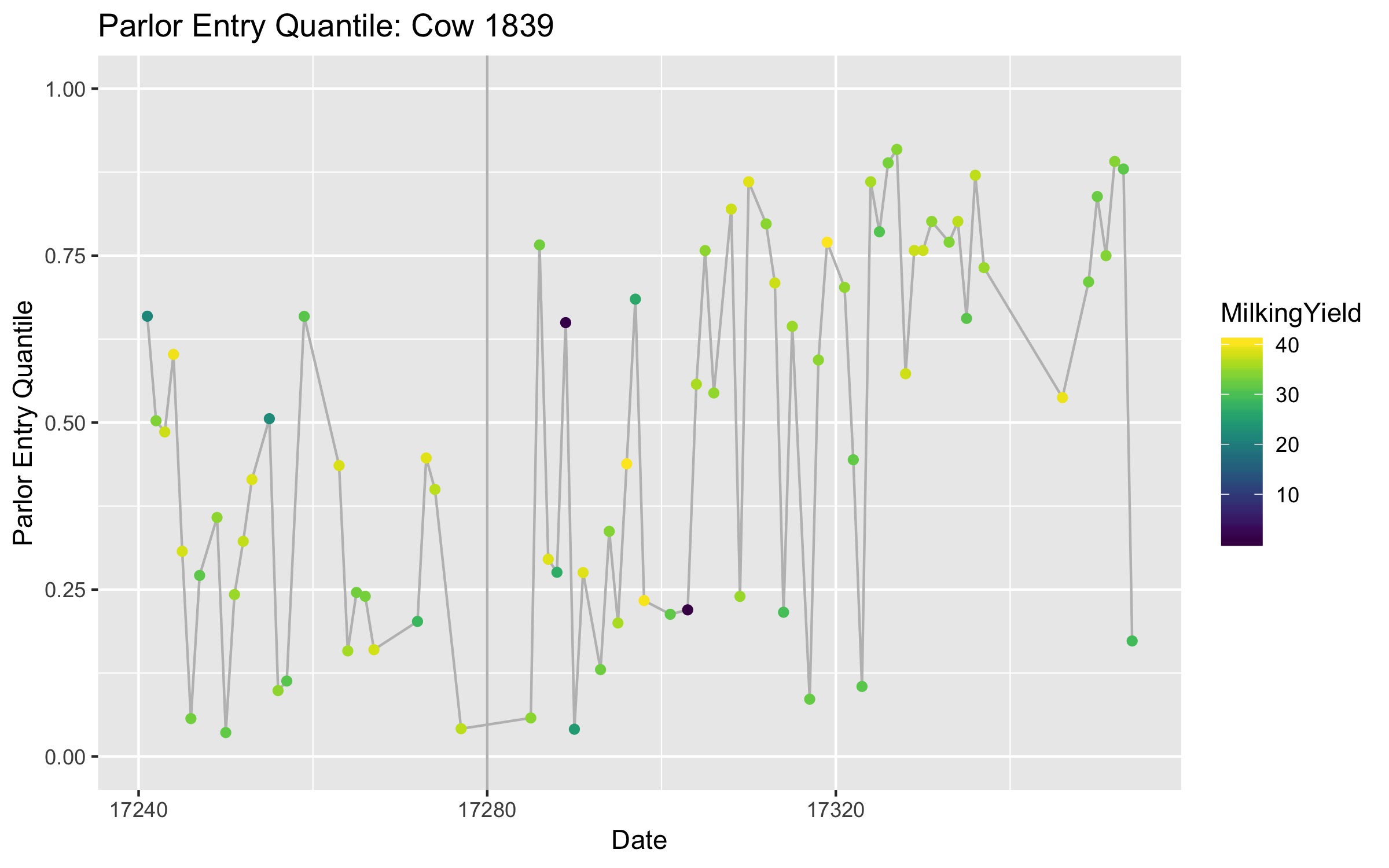

Supplement: Supplementary file 2 [file Data_Sheet_2.ZIP › Milking Yield/Cow_1839.jpg]

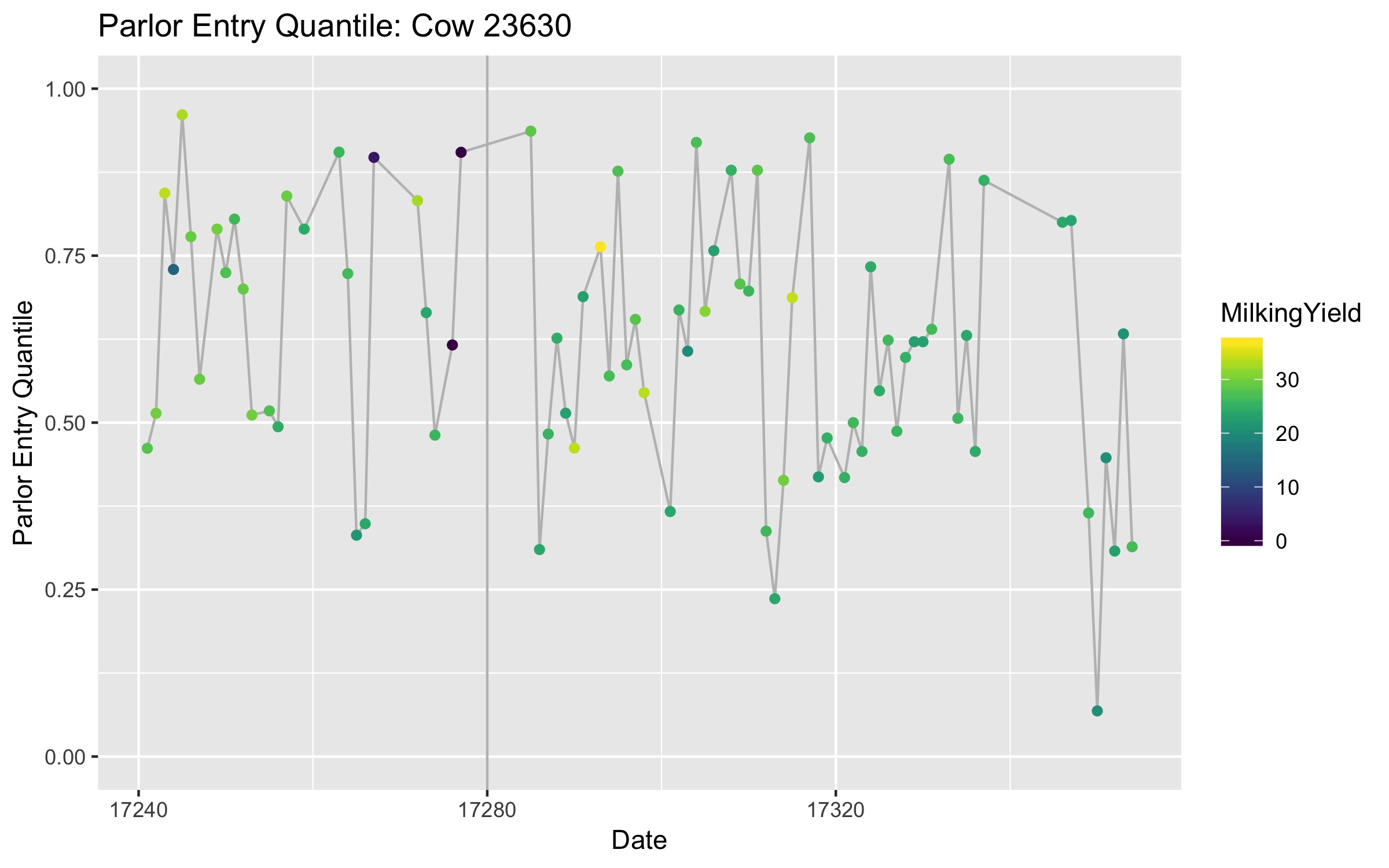

Supplement: Supplementary file 2 [file Data_Sheet_2.ZIP › Milking Yield/Cow_23630.jpg]

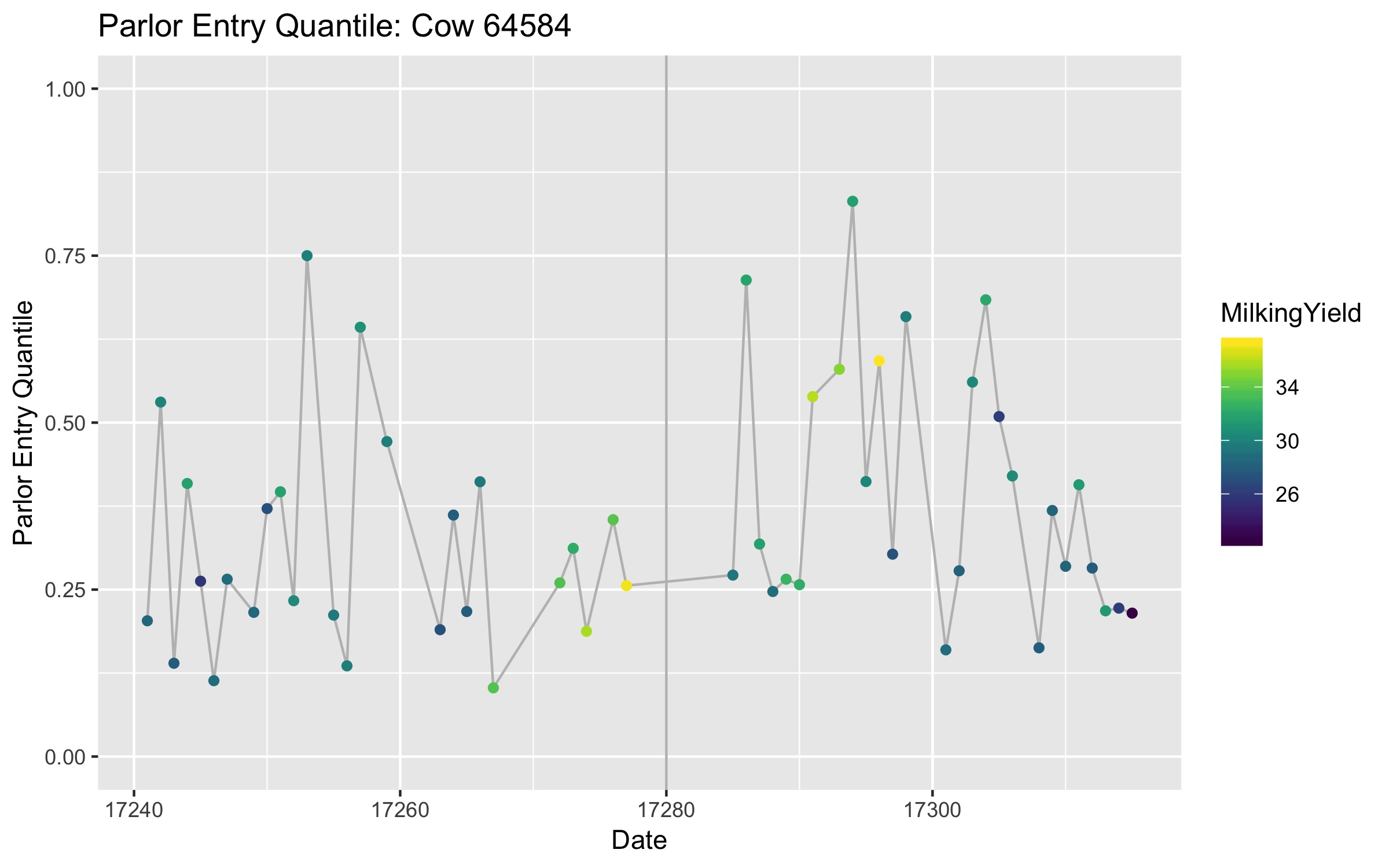

Supplement: Supplementary file 2 [file Data_Sheet_2.ZIP › Milking Yield/Cow_64584.jpg]

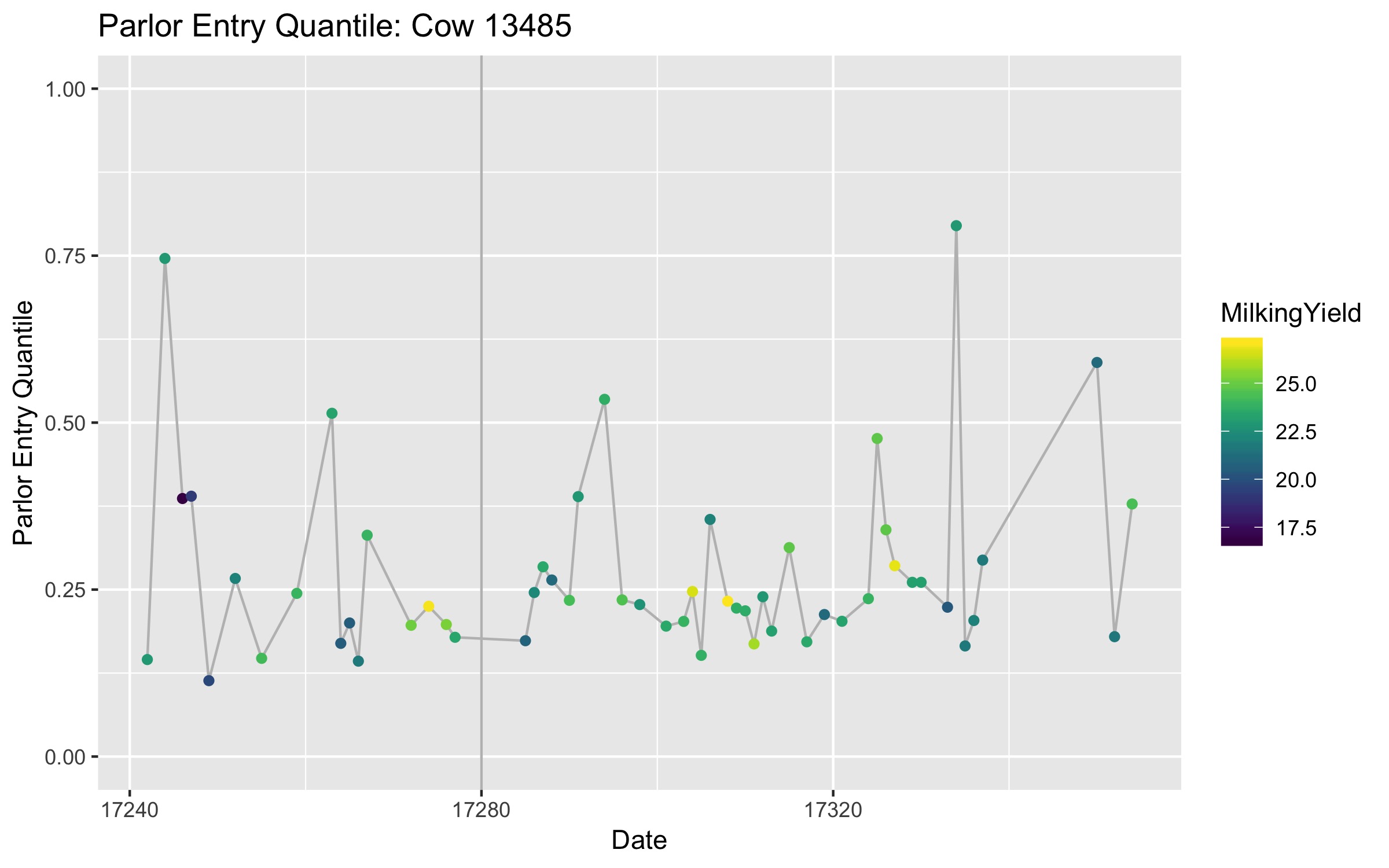

Supplement: Supplementary file 2 [file Data_Sheet_2.ZIP › Milking Yield/Cow_13485.jpg]

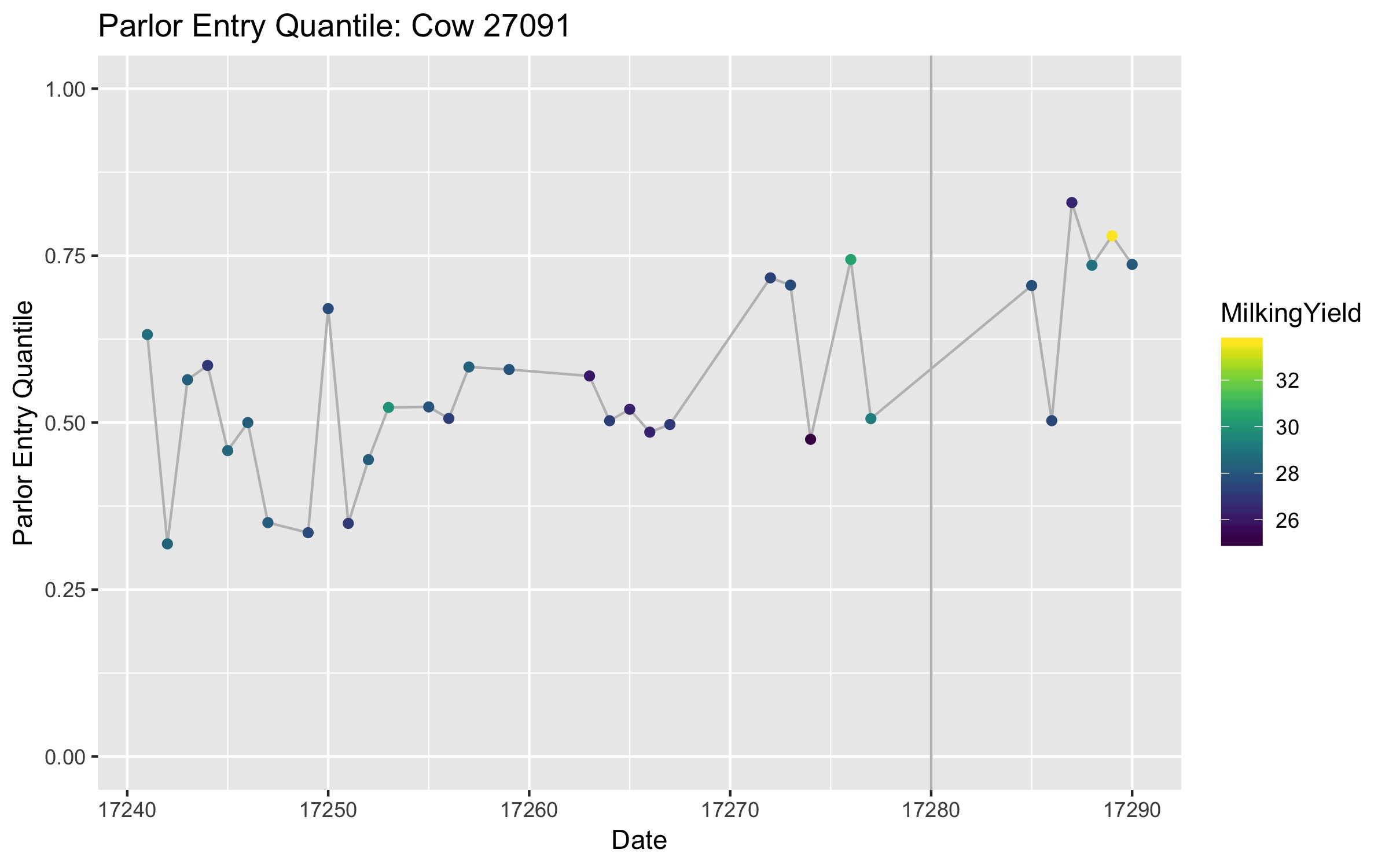

Supplement: Supplementary file 2 [file Data_Sheet_2.ZIP › Milking Yield/Cow_27091.jpg]

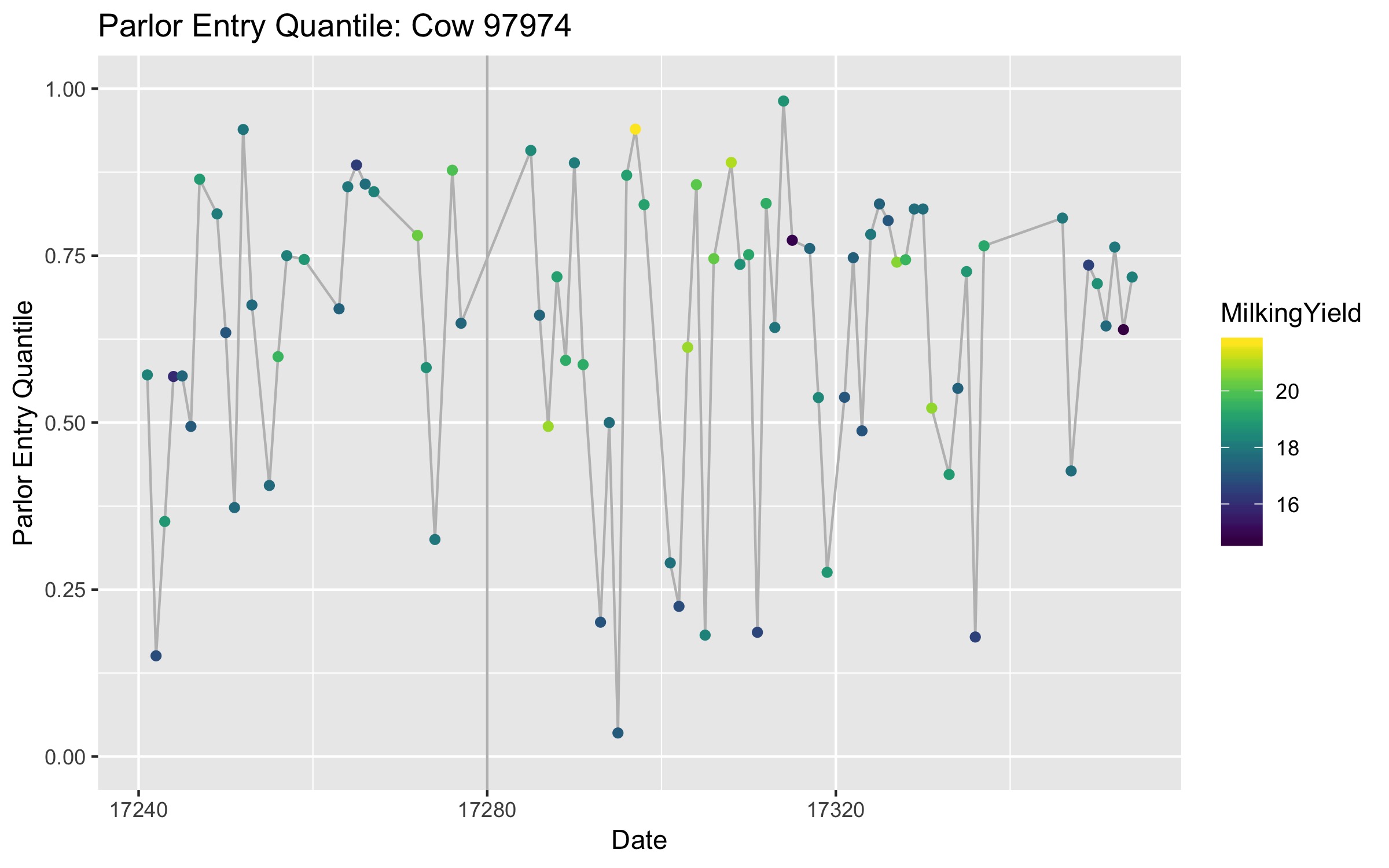

Supplement: Supplementary file 2 [file Data_Sheet_2.ZIP › Milking Yield/Cow_97974.jpg]

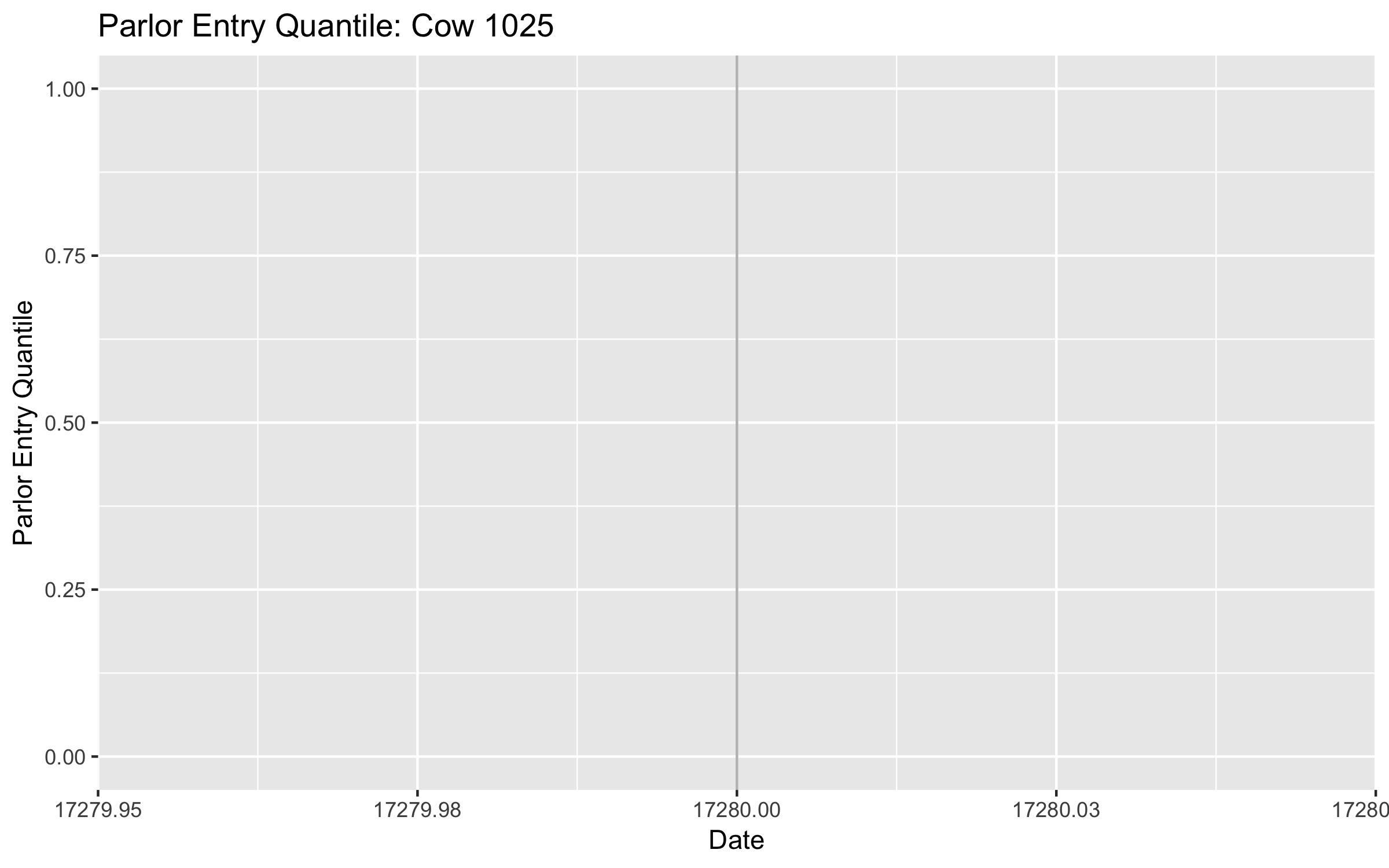

Supplement: Supplementary file 2 [file Data_Sheet_2.ZIP › Milking Yield/Cow_1025.jpg]

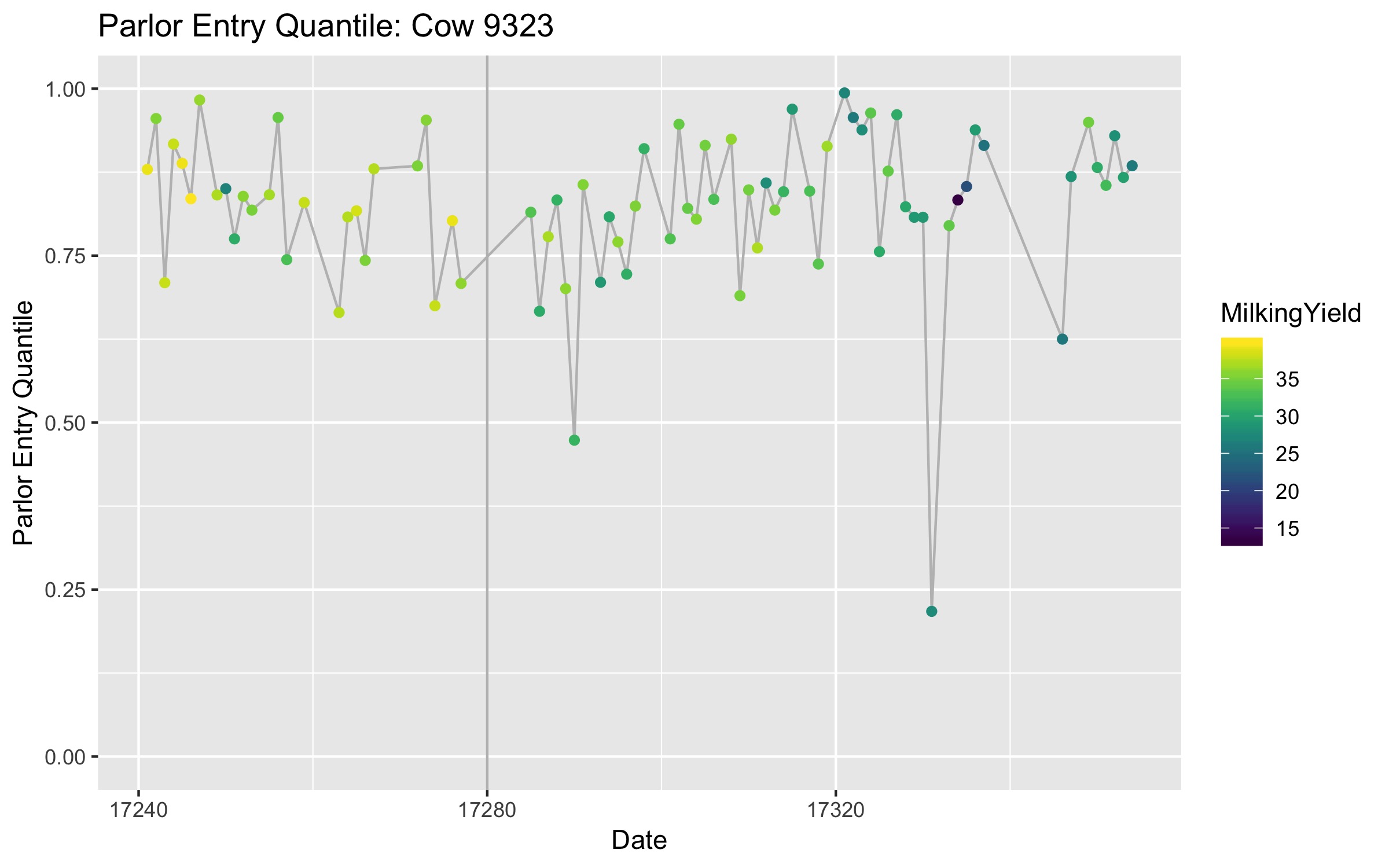

Supplement: Supplementary file 2 [file Data_Sheet_2.ZIP › Milking Yield/Cow_9323.jpg]

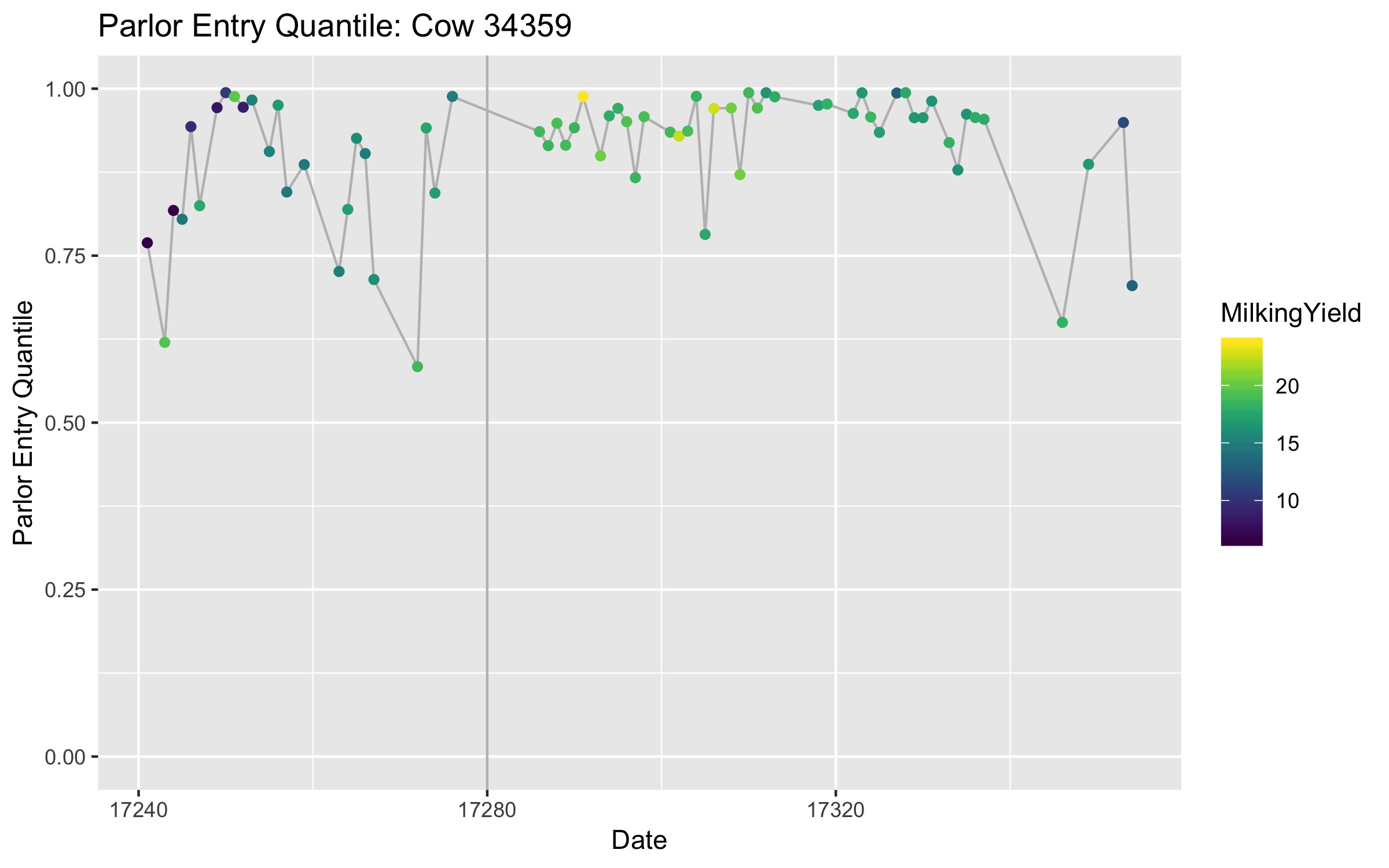

Supplement: Supplementary file 2 [file Data_Sheet_2.ZIP › Milking Yield/Cow_34359.jpg]

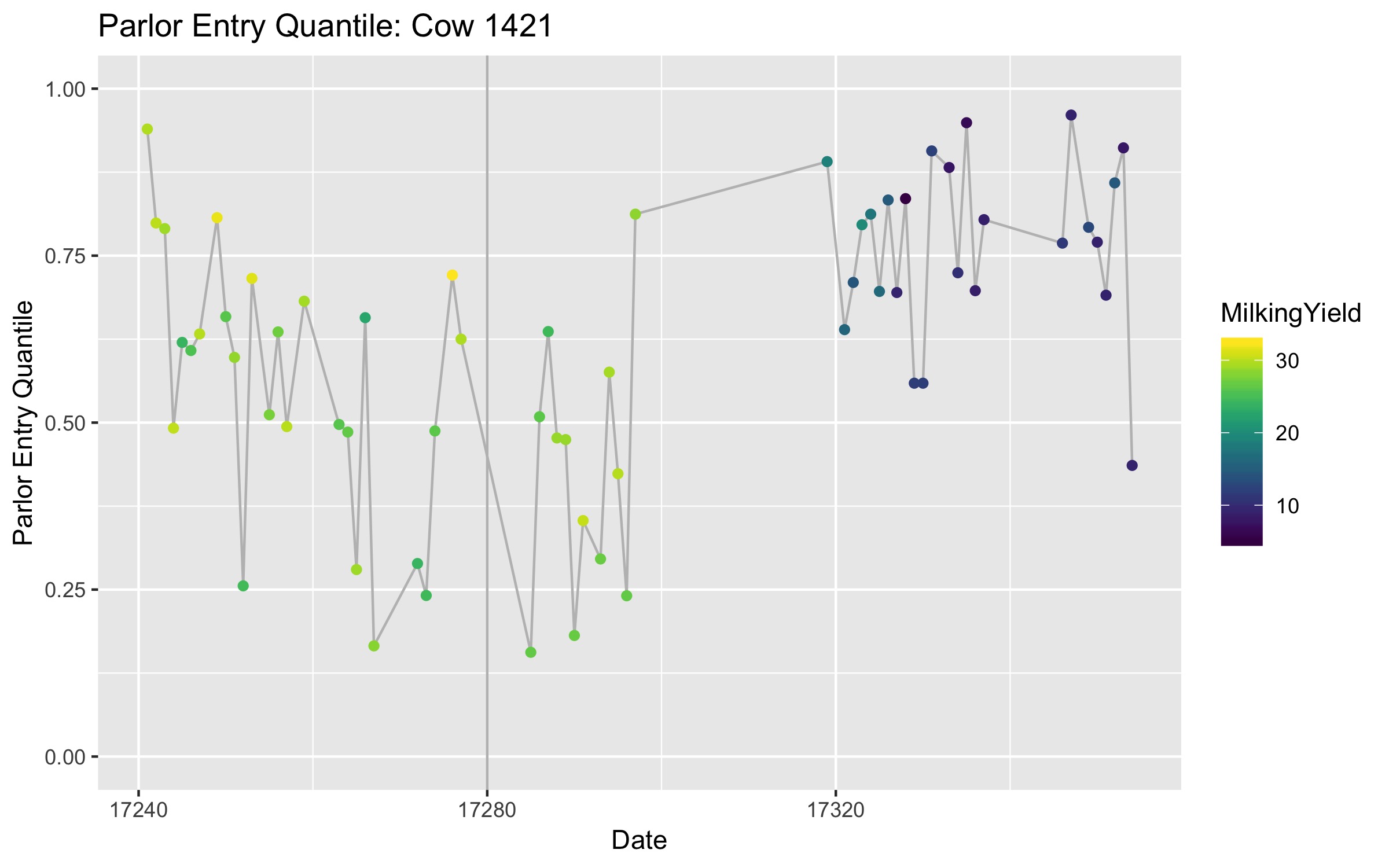

Supplement: Supplementary file 2 [file Data_Sheet_2.ZIP › Milking Yield/Cow_1421.jpg]

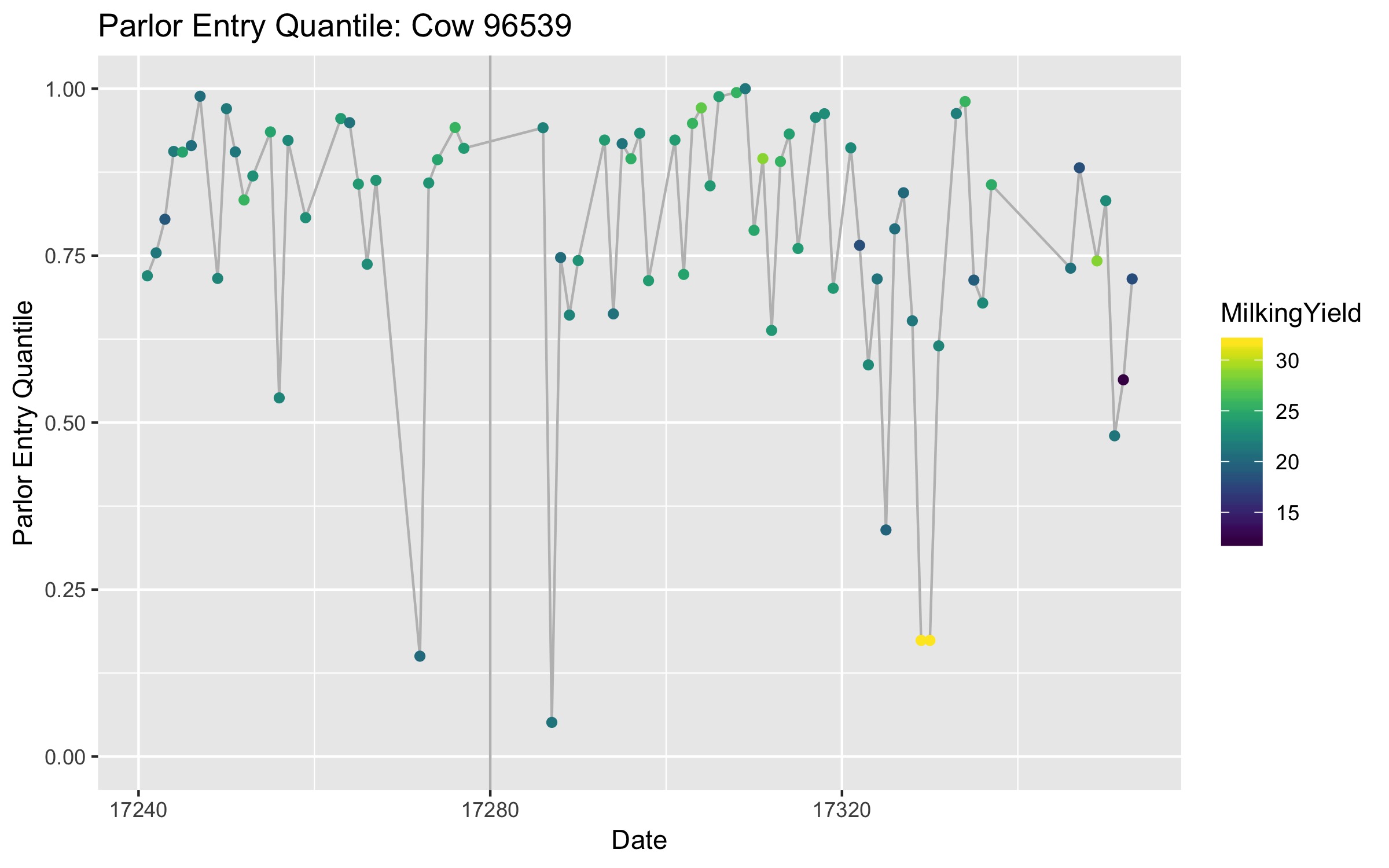

Supplement: Supplementary file 2 [file Data_Sheet_2.ZIP › Milking Yield/Cow_96539.jpg]

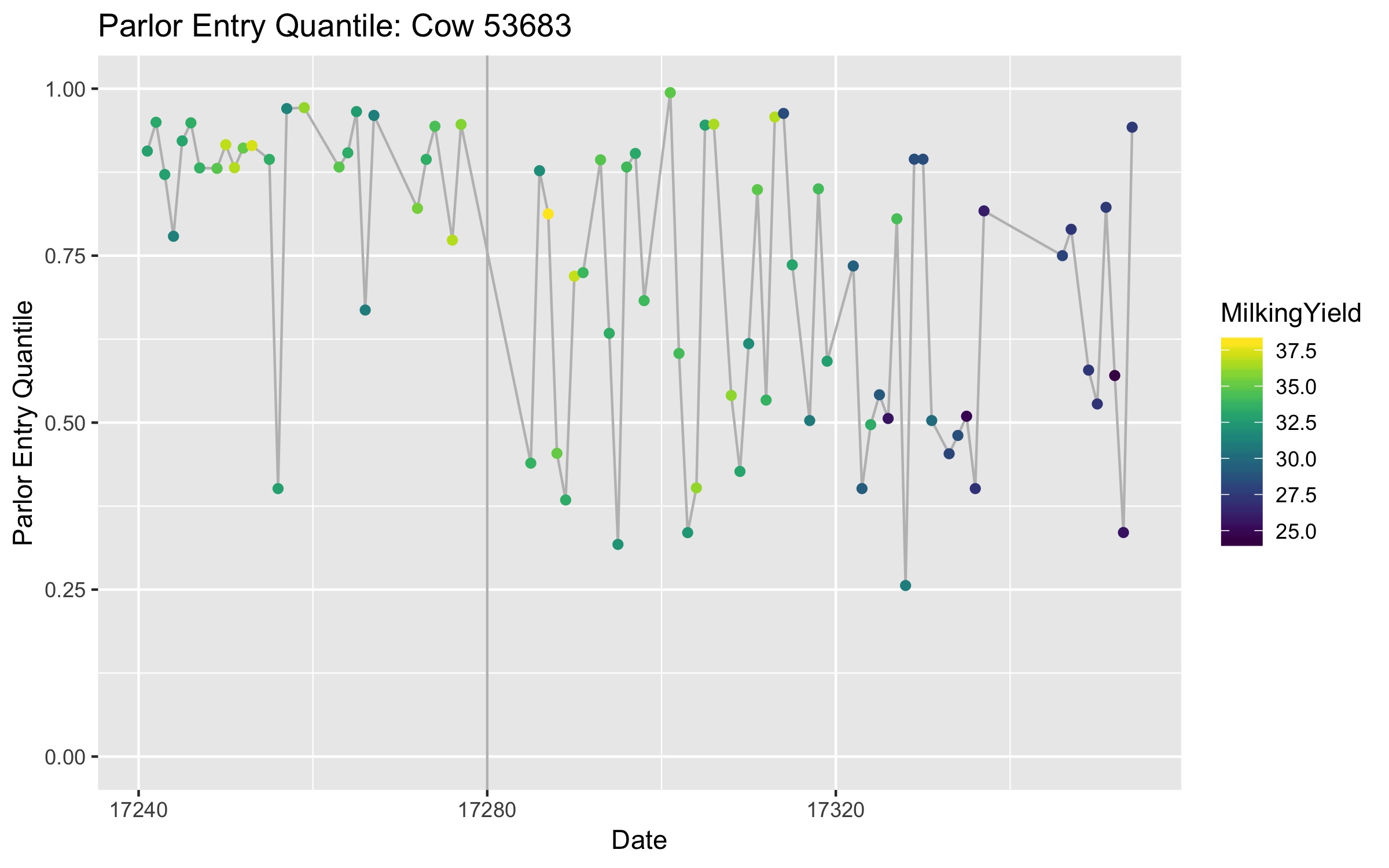

Supplement: Supplementary file 2 [file Data_Sheet_2.ZIP › Milking Yield/Cow_53683.jpg]
