# Supplementary figures and images for: Mind the Queue: A Case Study in Visualizing Heterogeneous Behavioral Patterns in Livestock Sensor Data Using Unsupervised Machine Learning Techniques (part 3 of 4)
Source: Front Vet Sci. 2020 Aug 13;7:523. doi: 10.3389/fvets.2020.00523 (PMC7518149; doi:10.3389/fvets.2020.00523)

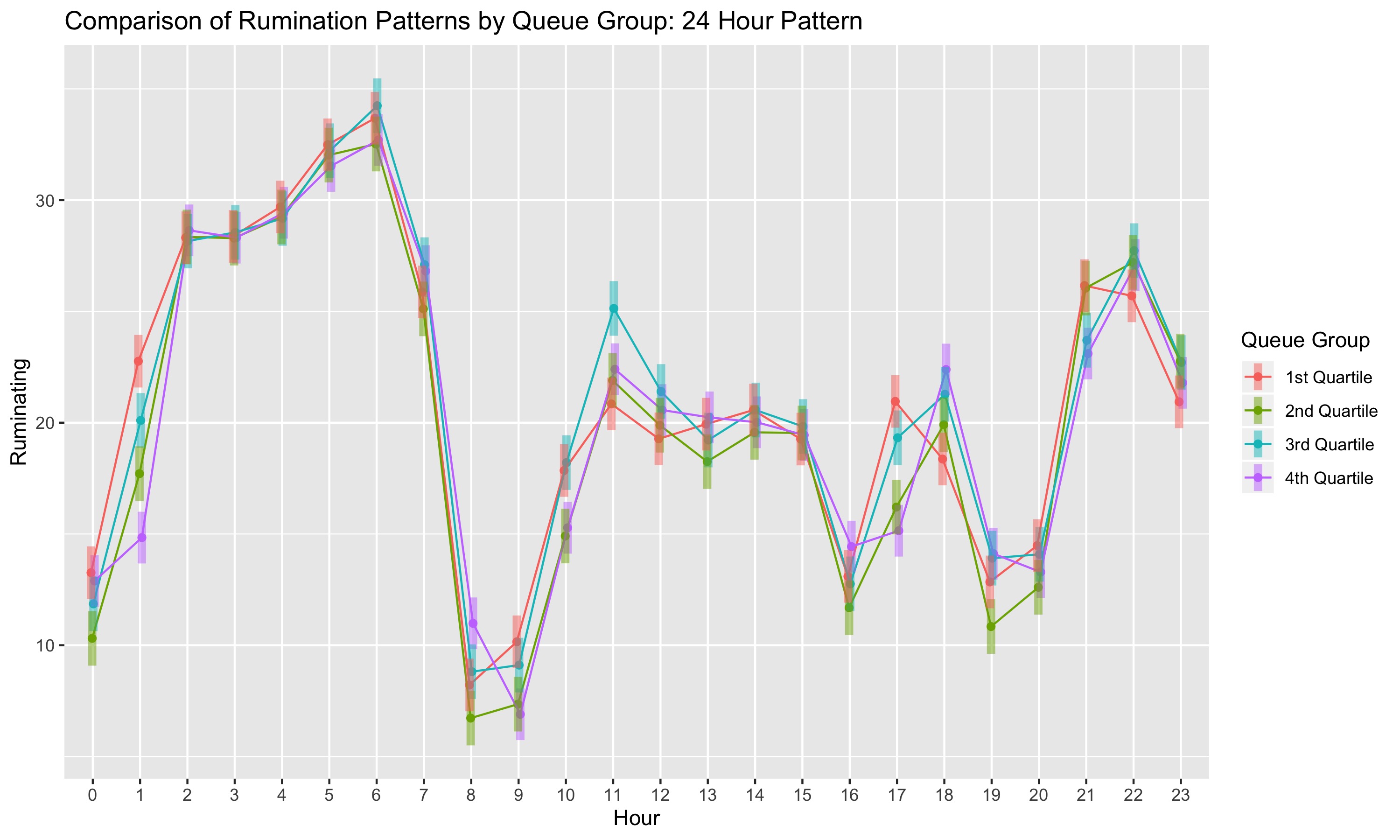

Supplement: Supplementary file 3 [file Data_Sheet_3.ZIP › MeanPlots/Ruminating_Cyc.jpg]

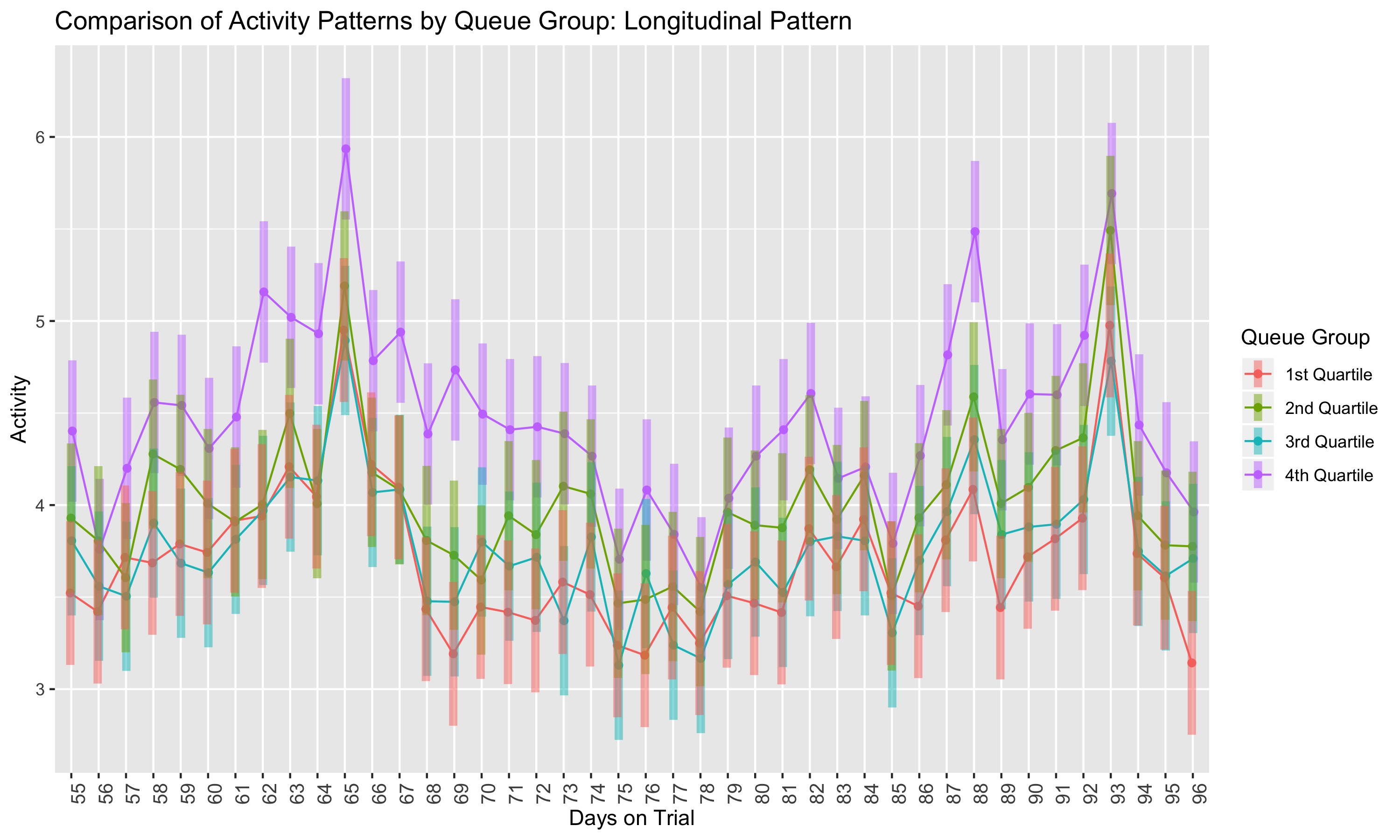

Supplement: Supplementary file 3 [file Data_Sheet_3.ZIP › MeanPlots/Activity_Long.jpg]

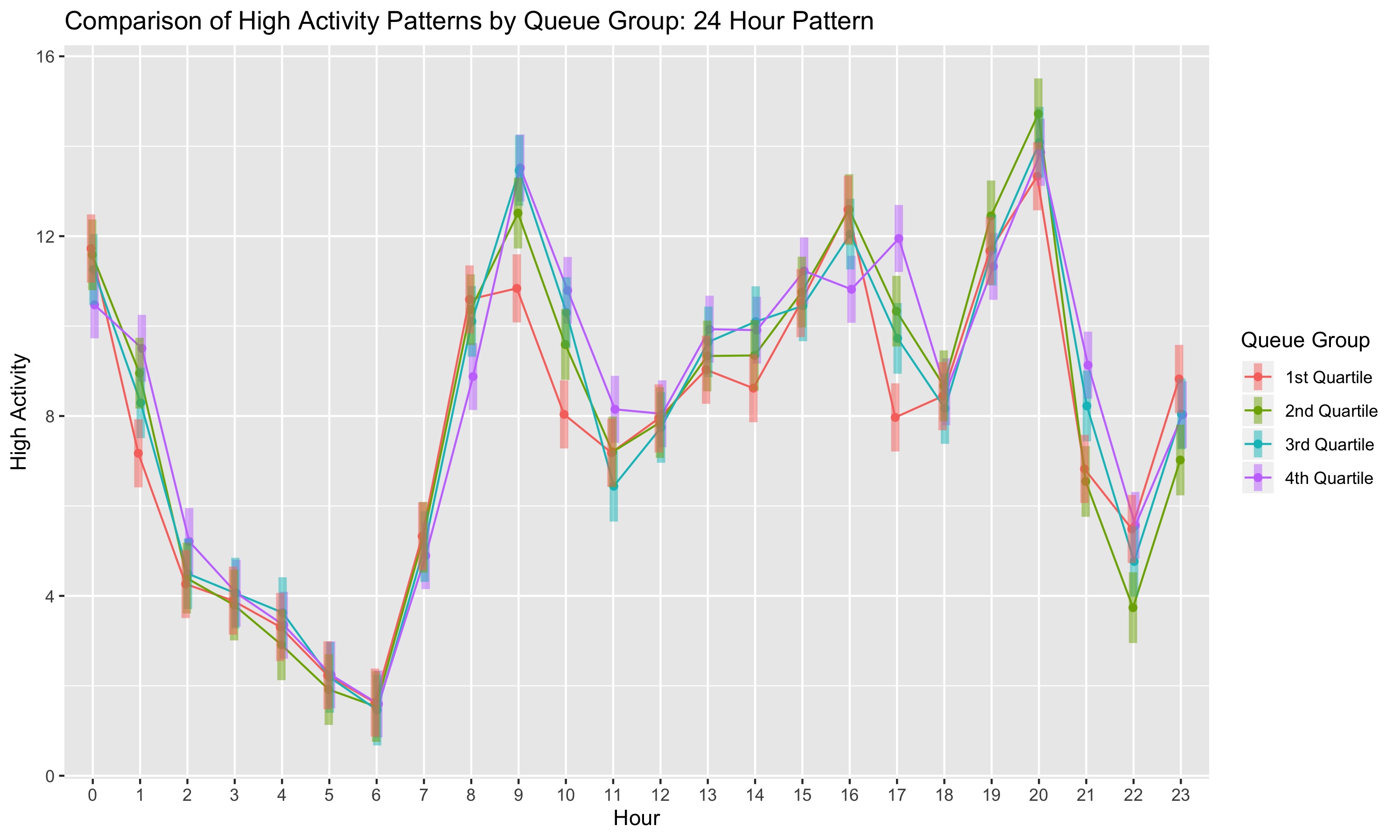

Supplement: Supplementary file 3 [file Data_Sheet_3.ZIP › MeanPlots/HighActivity_Cyc.jpg]

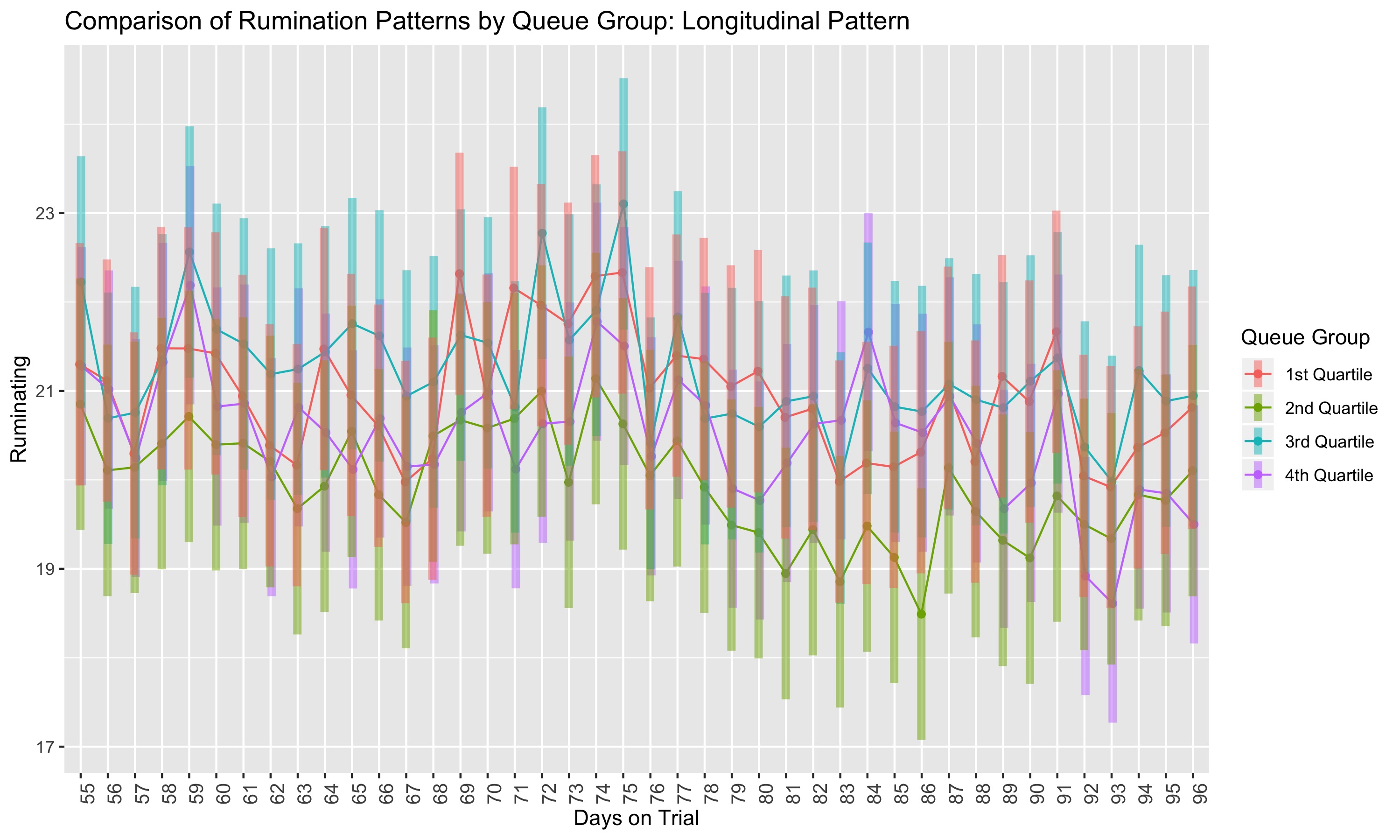

Supplement: Supplementary file 3 [file Data_Sheet_3.ZIP › MeanPlots/Ruminating_Long.jpg]

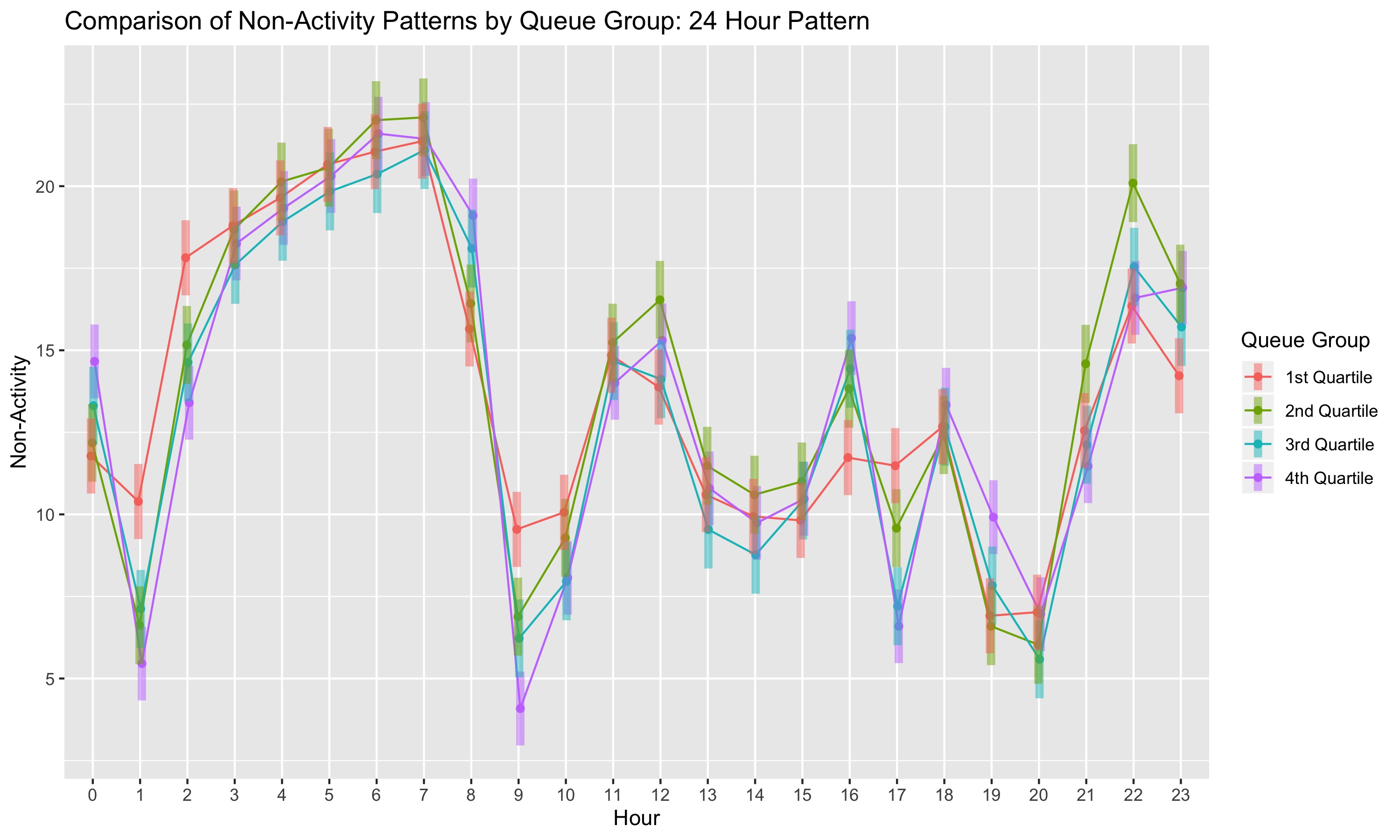

Supplement: Supplementary file 3 [file Data_Sheet_3.ZIP › MeanPlots/NonActivity_Cyc.jpg]

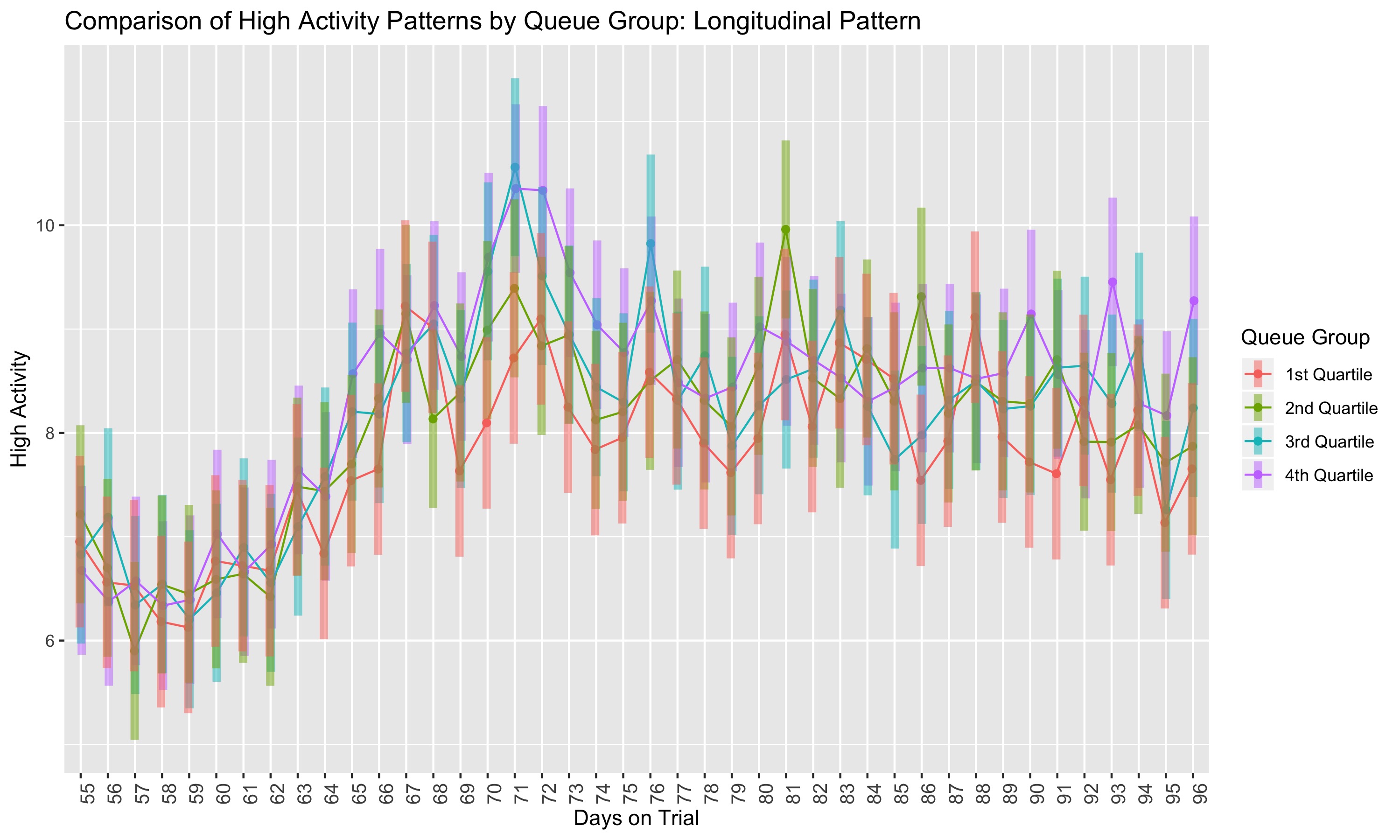

Supplement: Supplementary file 3 [file Data_Sheet_3.ZIP › MeanPlots/HighActivity_Long.jpg]

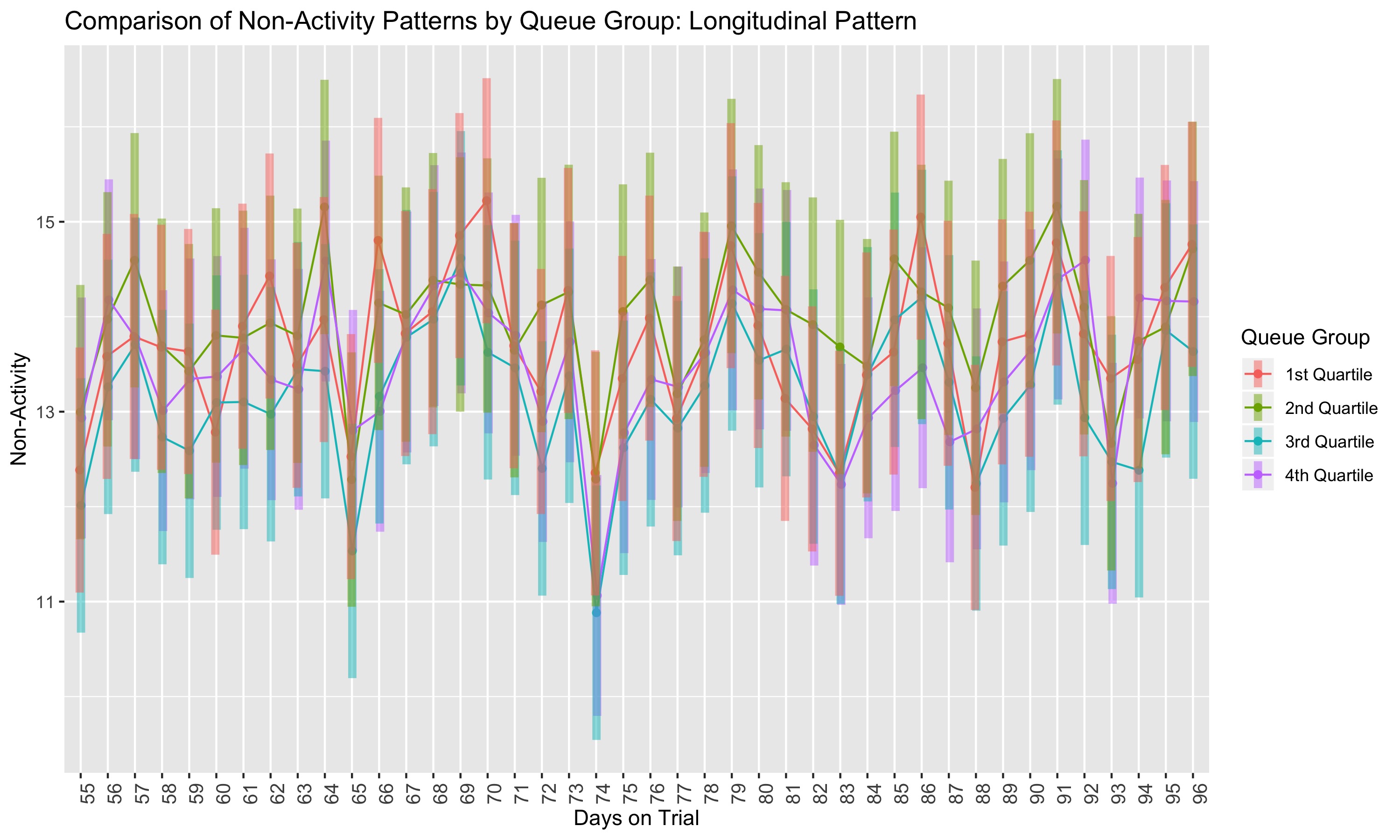

Supplement: Supplementary file 3 [file Data_Sheet_3.ZIP › MeanPlots/NonActivity_Long.jpg]

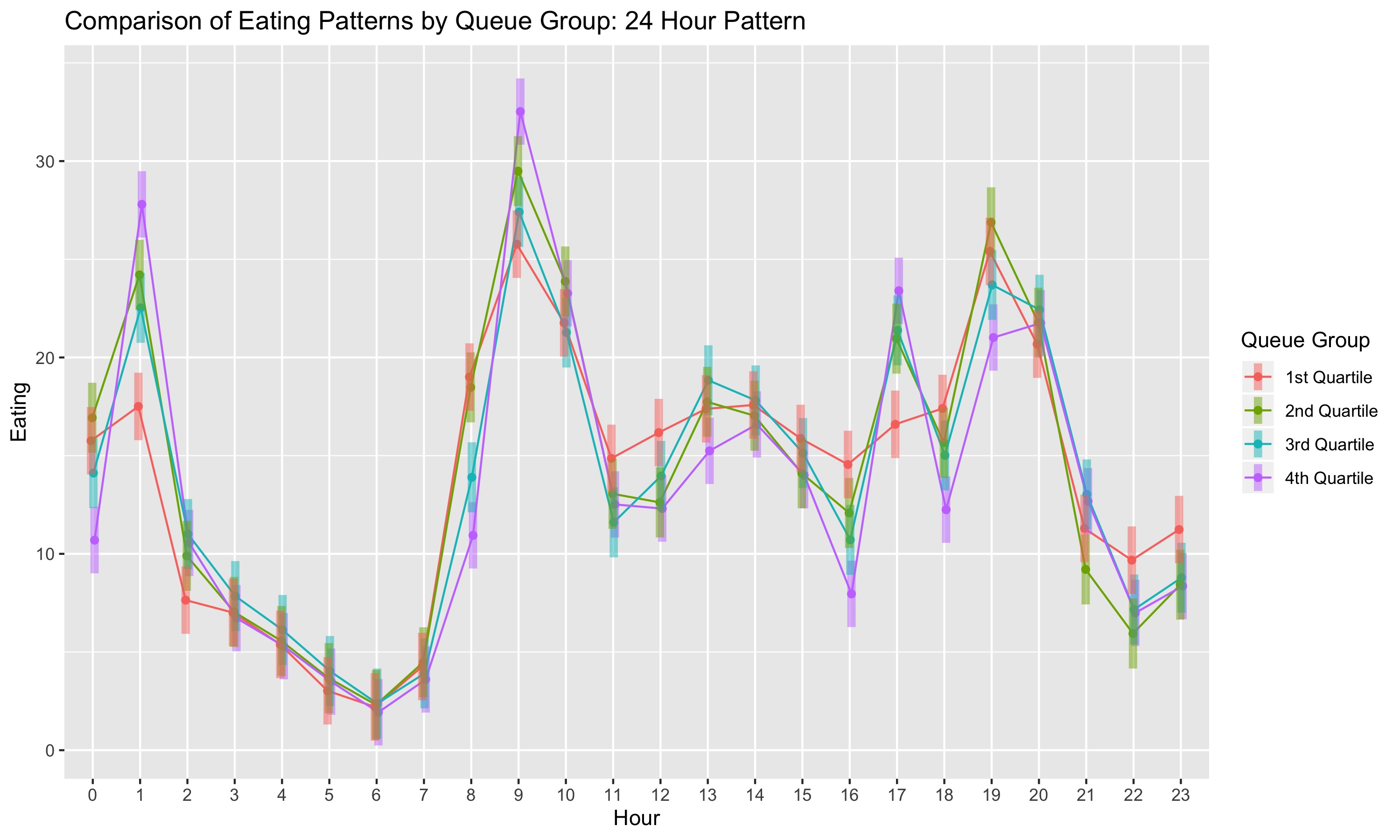

Supplement: Supplementary file 3 [file Data_Sheet_3.ZIP › MeanPlots/Eating_Cyc.jpg]

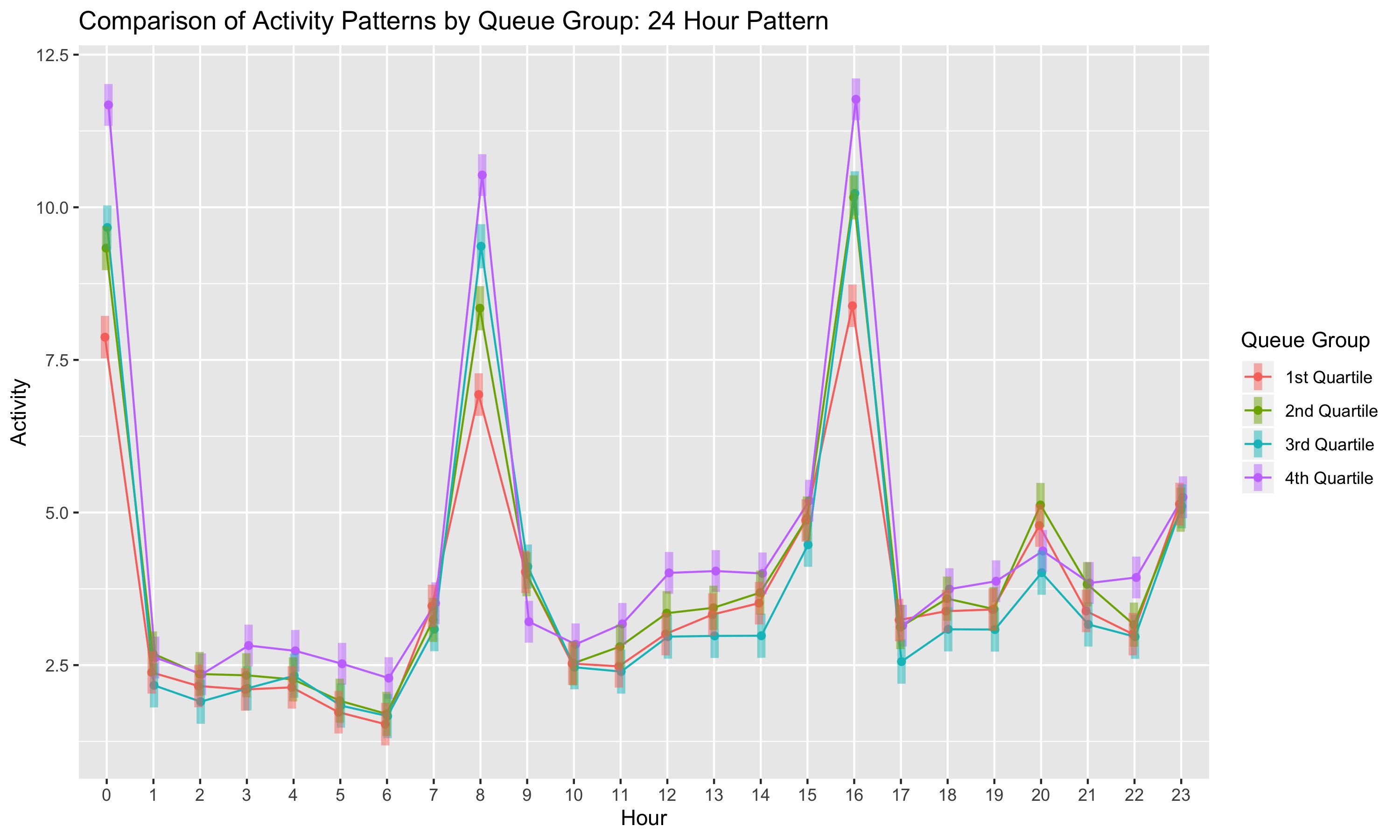

Supplement: Supplementary file 3 [file Data_Sheet_3.ZIP › MeanPlots/Activity_Cyc.jpg]

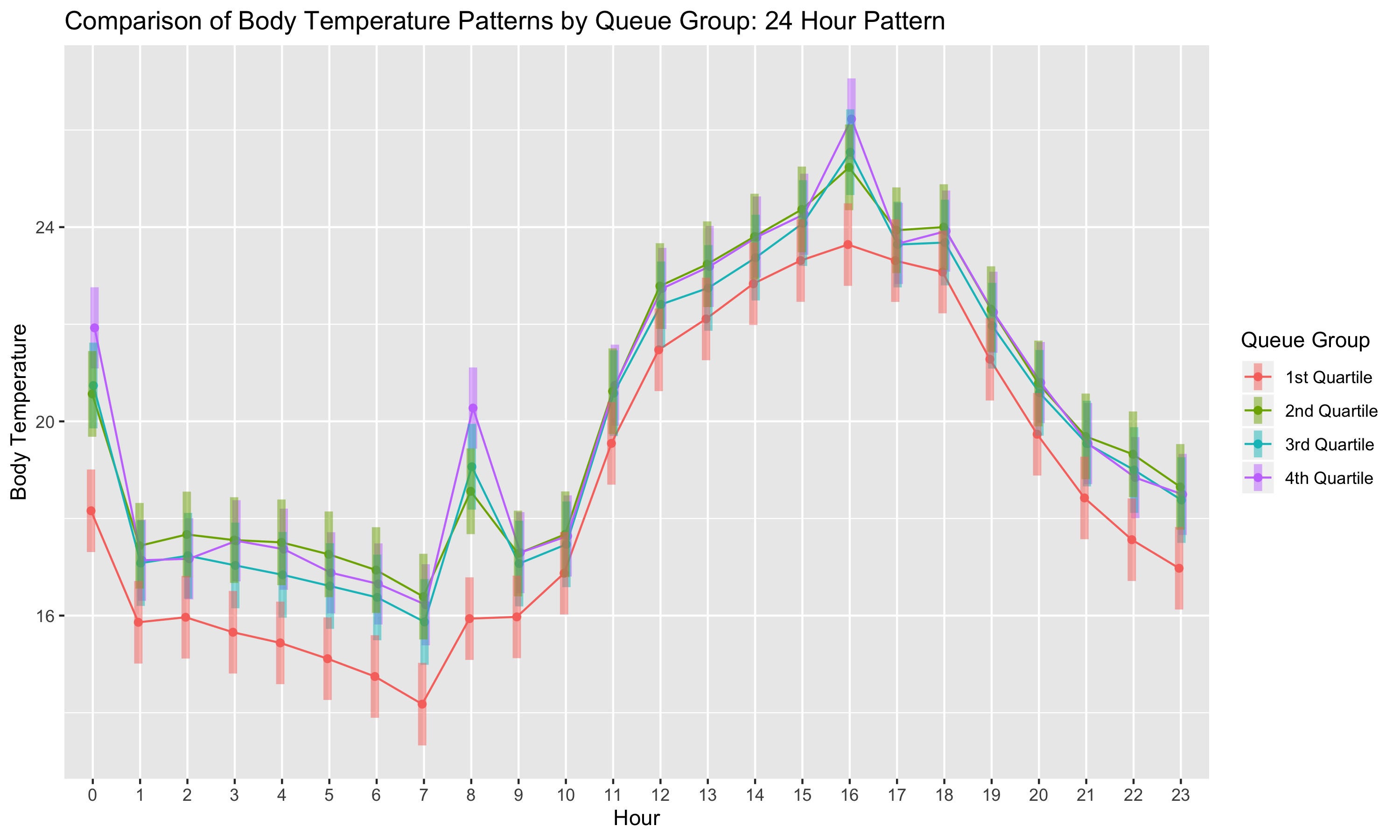

Supplement: Supplementary file 3 [file Data_Sheet_3.ZIP › MeanPlots/Temperature_Cyc.jpg]

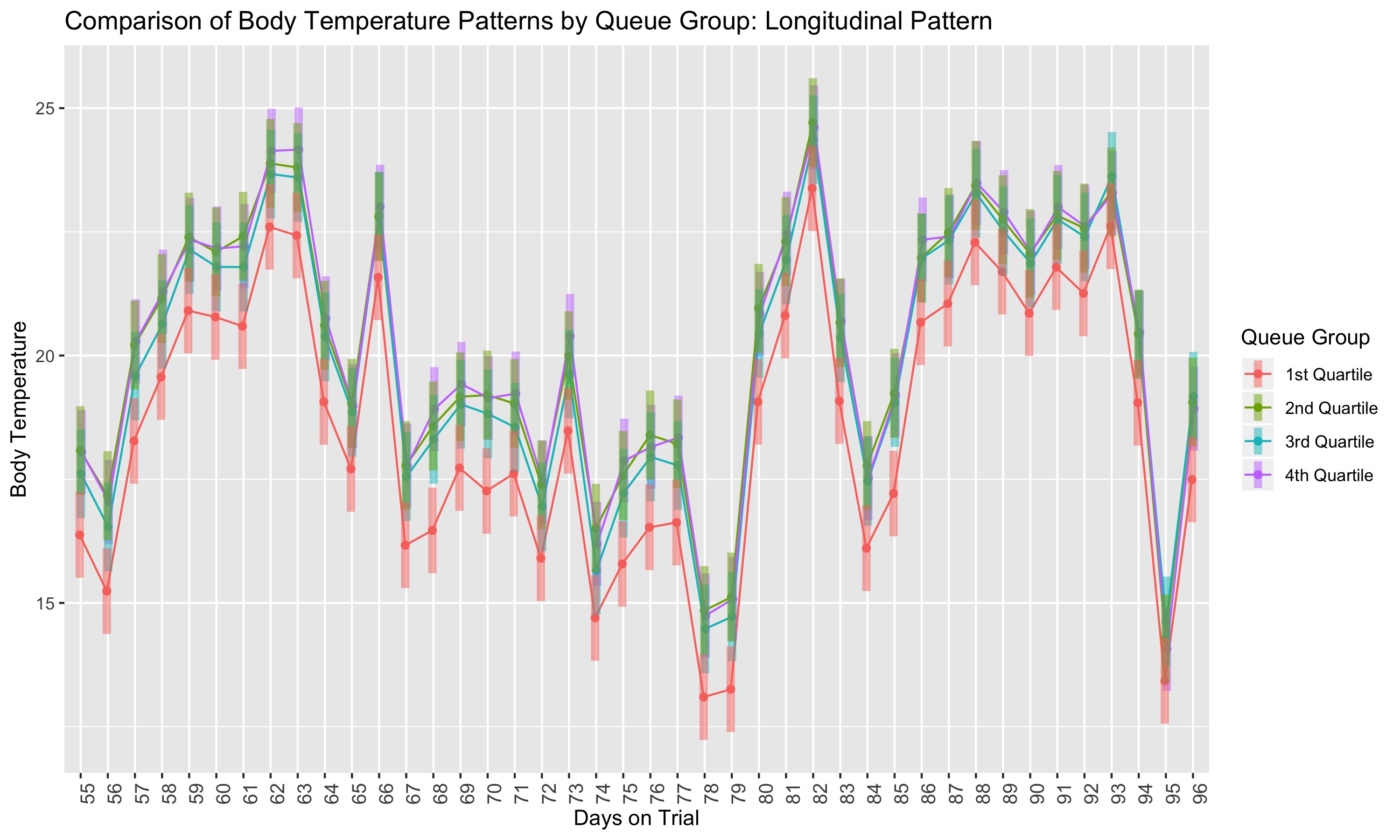

Supplement: Supplementary file 3 [file Data_Sheet_3.ZIP › MeanPlots/Temperature_Long.jpg]

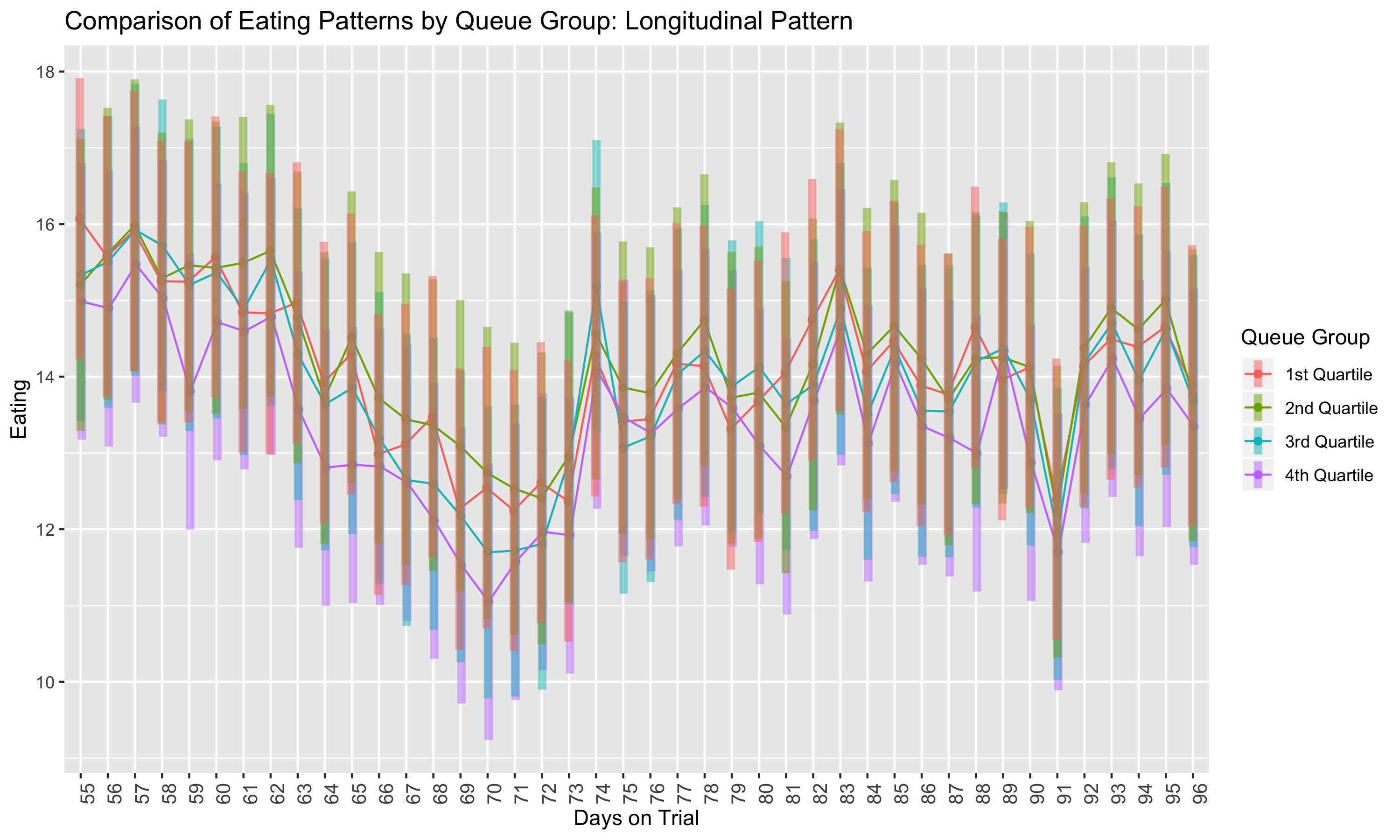

Supplement: Supplementary file 3 [file Data_Sheet_3.ZIP › MeanPlots/Eating_Long.jpg]

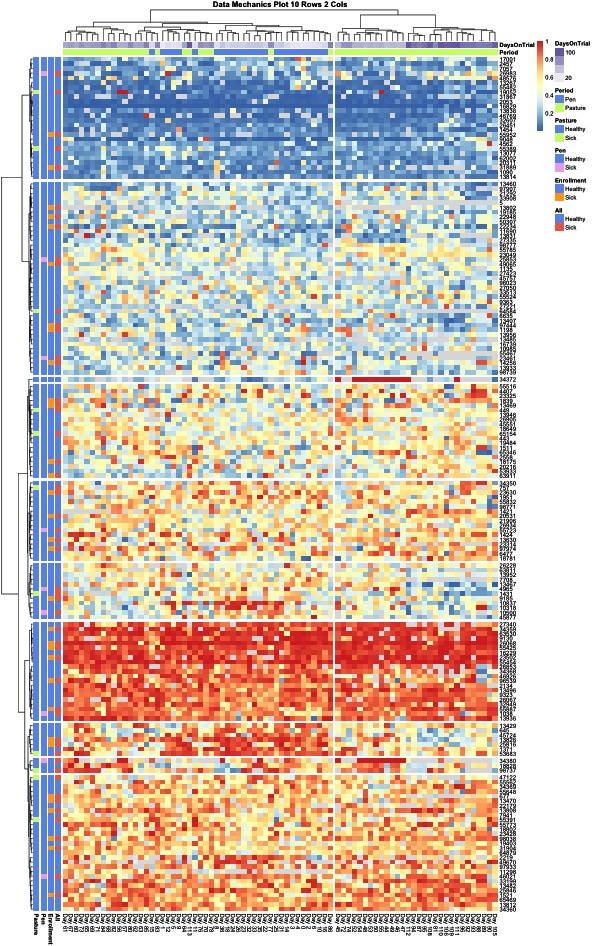

Supplement: Supplementary file 5 [file Data_Sheet_5.ZIP › Grid_LR/DatMechPlotOutR10C2 _Final.jpeg]

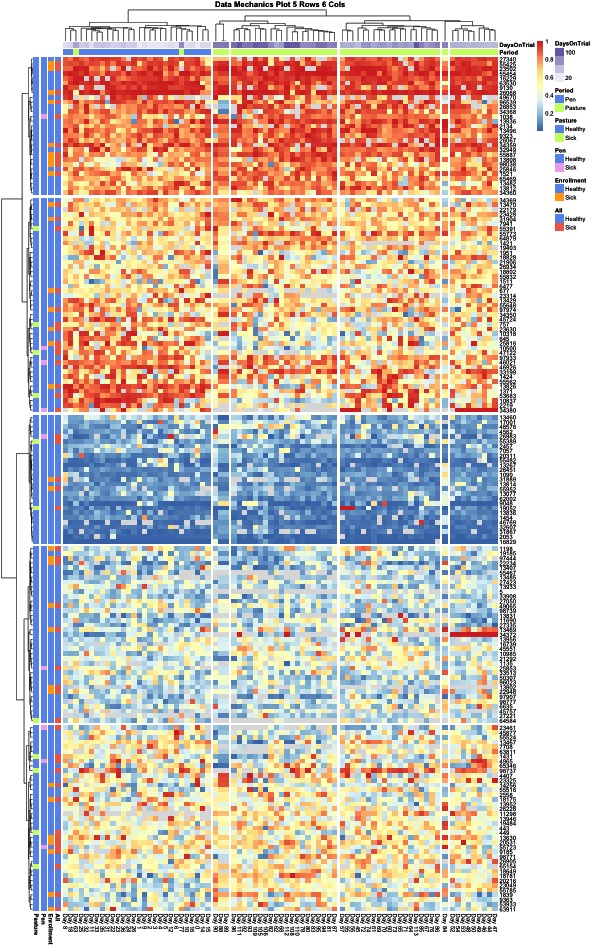

Supplement: Supplementary file 5 [file Data_Sheet_5.ZIP › Grid_LR/DatMechPlotOutR5C6 _Final.jpeg]

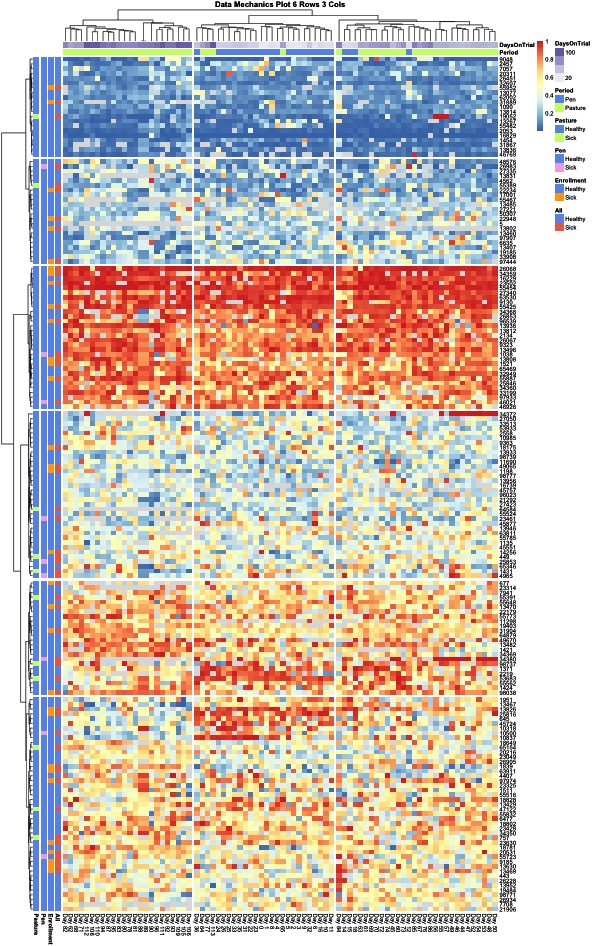

Supplement: Supplementary file 5 [file Data_Sheet_5.ZIP › Grid_LR/DatMechPlotOutR6C3 _Final.jpeg]

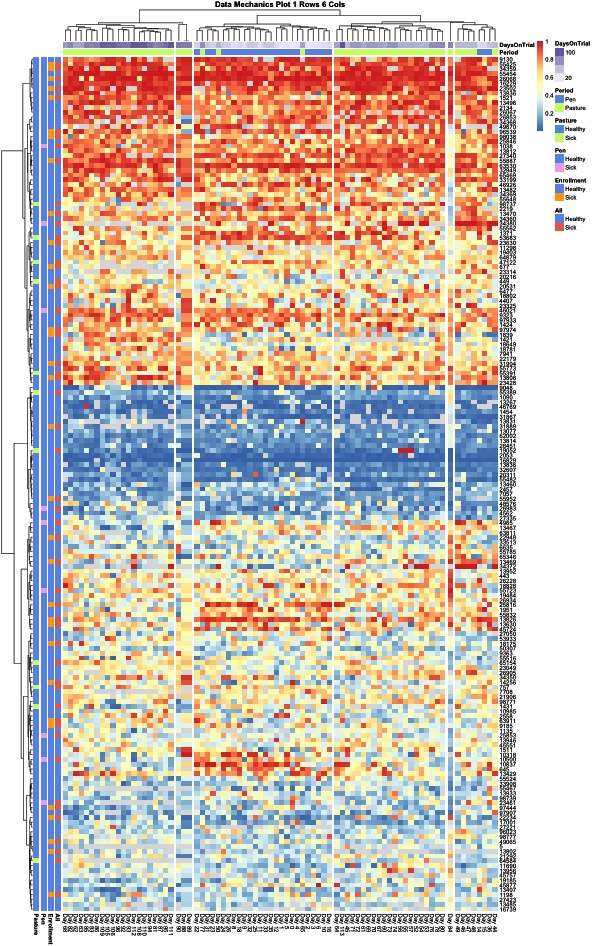

Supplement: Supplementary file 5 [file Data_Sheet_5.ZIP › Grid_LR/DatMechPlotOutR1C6 _Final.jpeg]

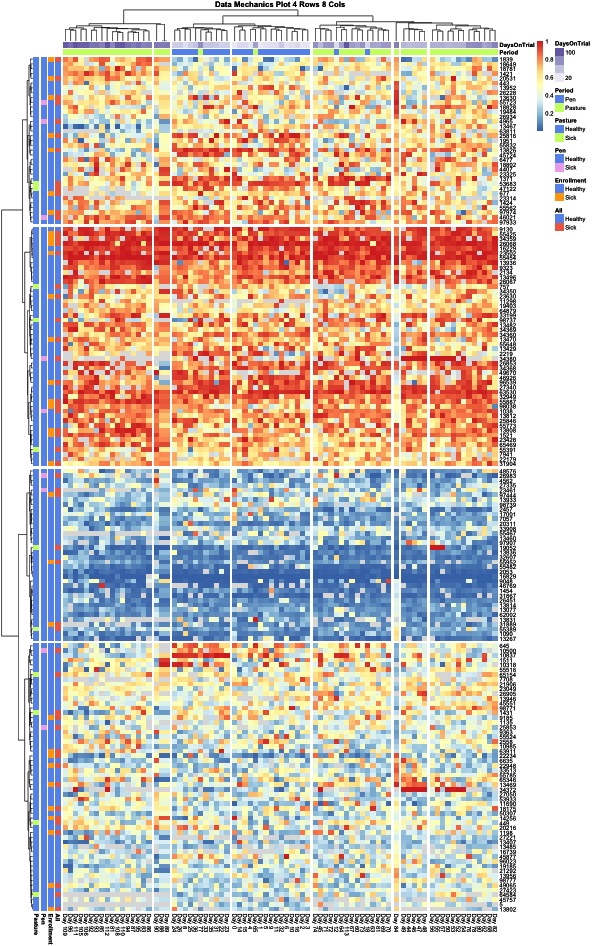

Supplement: Supplementary file 5 [file Data_Sheet_5.ZIP › Grid_LR/DatMechPlotOutR4C8 _Final.jpeg]

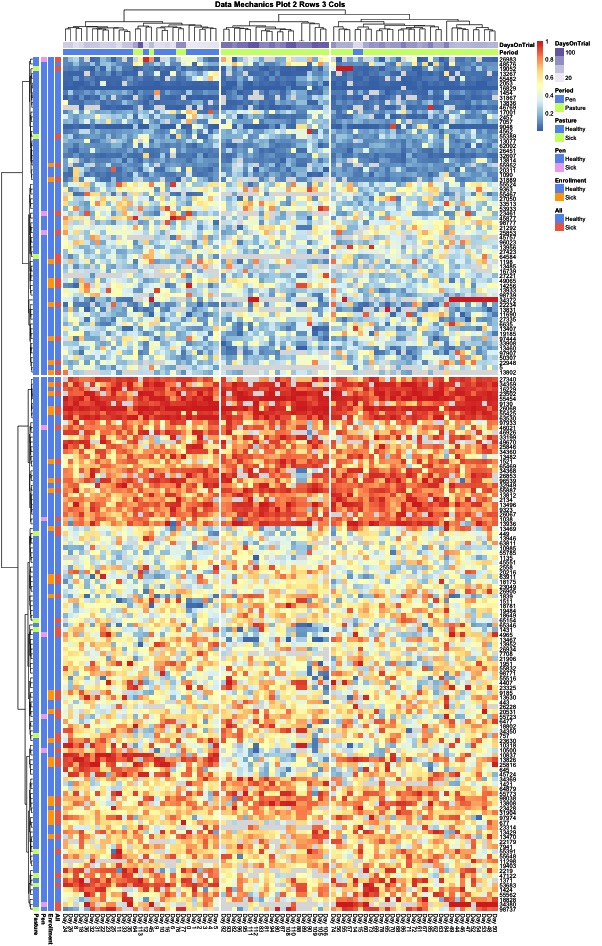

Supplement: Supplementary file 5 [file Data_Sheet_5.ZIP › Grid_LR/DatMechPlotOutR2C3 _Final.jpeg]

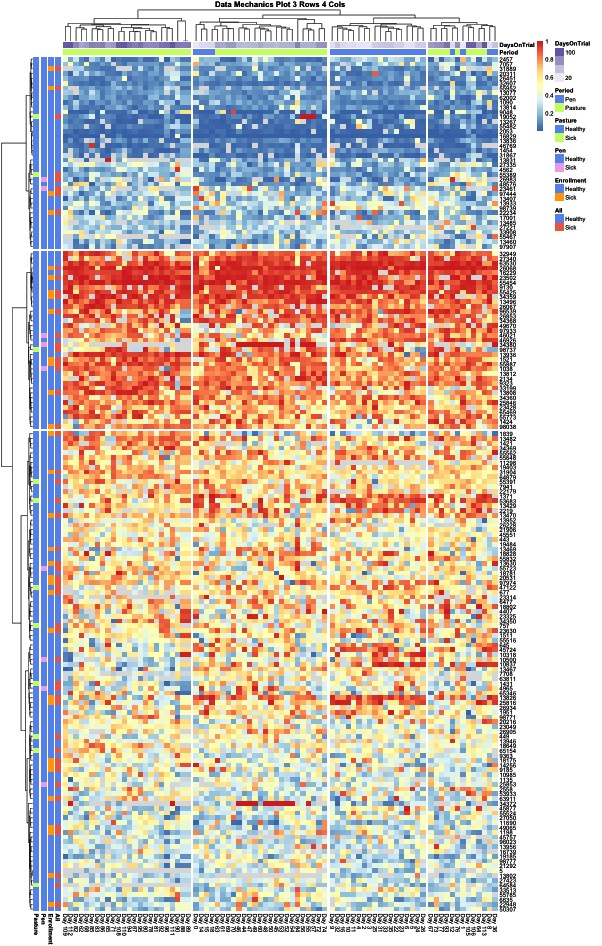

Supplement: Supplementary file 5 [file Data_Sheet_5.ZIP › Grid_LR/DatMechPlotOutR3C4 _Final.jpeg]

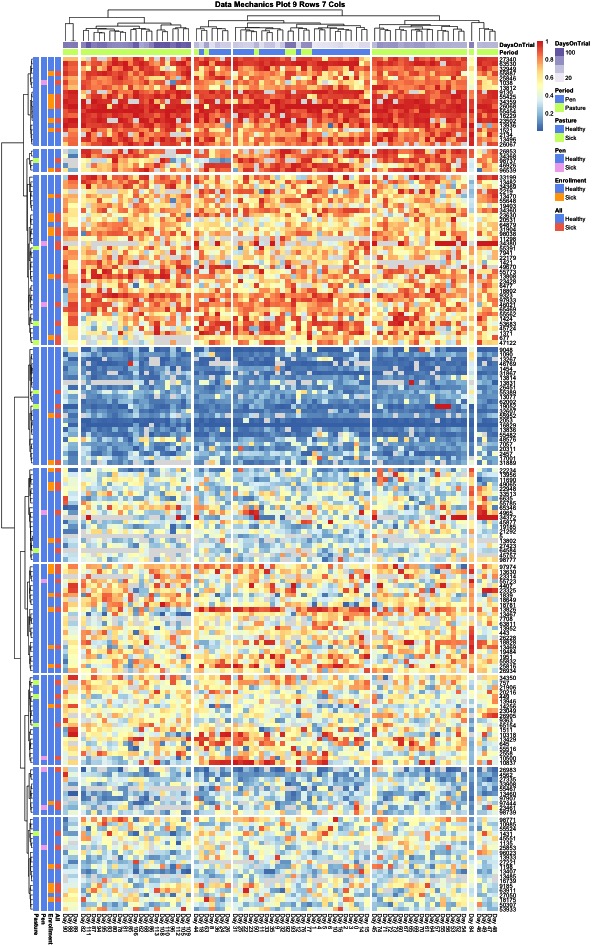

Supplement: Supplementary file 5 [file Data_Sheet_5.ZIP › Grid_LR/DatMechPlotOutR9C7 _Final.jpeg]

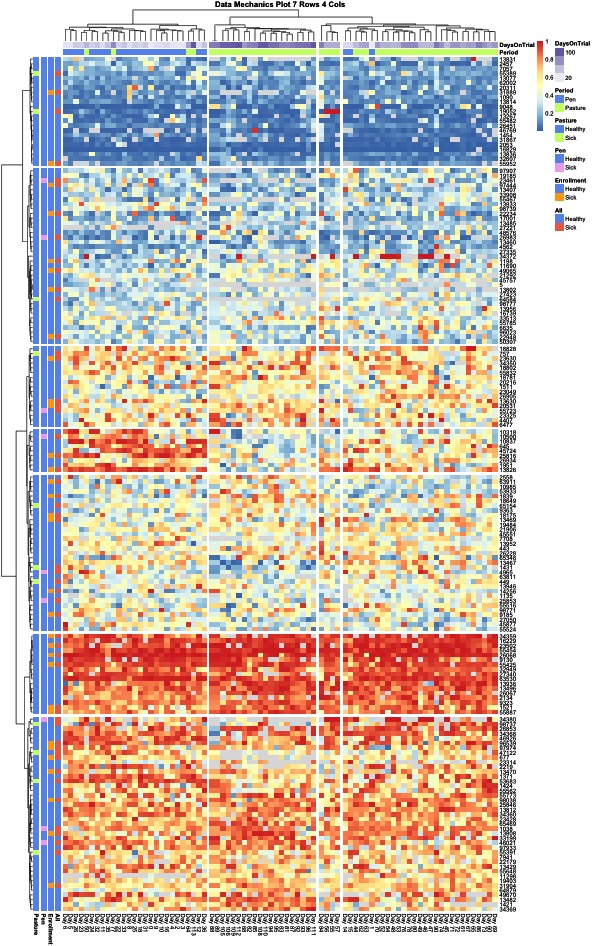

Supplement: Supplementary file 5 [file Data_Sheet_5.ZIP › Grid_LR/DatMechPlotOutR7C4 _Final.jpeg]

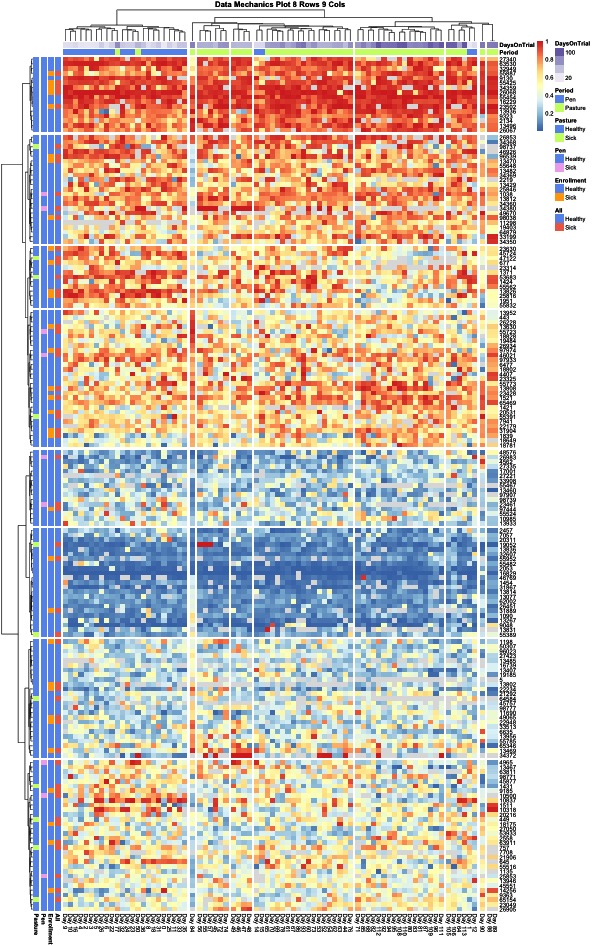

Supplement: Supplementary file 5 [file Data_Sheet_5.ZIP › Grid_LR/DatMechPlotOutR8C9 _Final.jpeg]

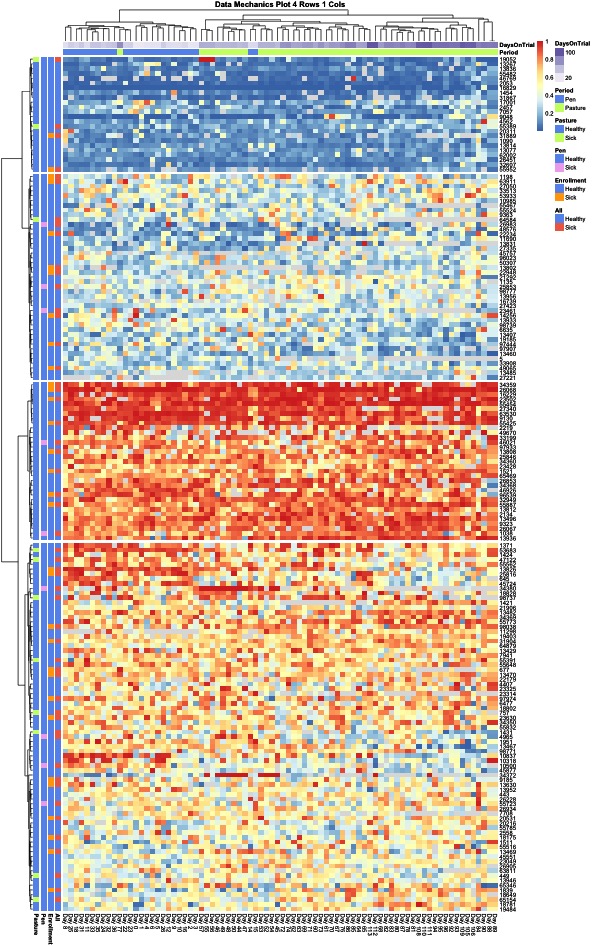

Supplement: Supplementary file 5 [file Data_Sheet_5.ZIP › Grid_LR/DatMechPlotOutR4C1 _Final.jpeg]

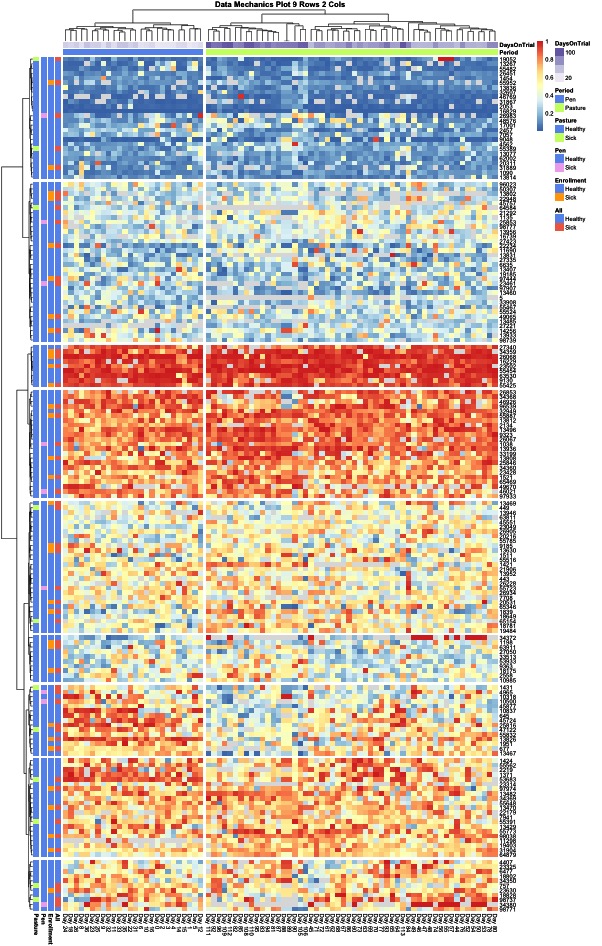

Supplement: Supplementary file 5 [file Data_Sheet_5.ZIP › Grid_LR/DatMechPlotOutR9C2 _Final.jpeg]

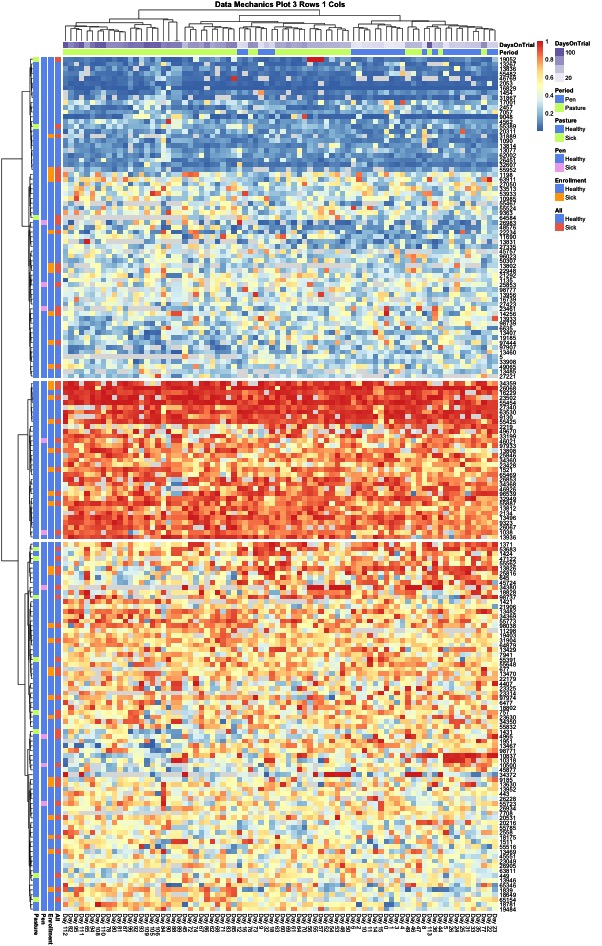

Supplement: Supplementary file 5 [file Data_Sheet_5.ZIP › Grid_LR/DatMechPlotOutR3C1 _Final.jpeg]

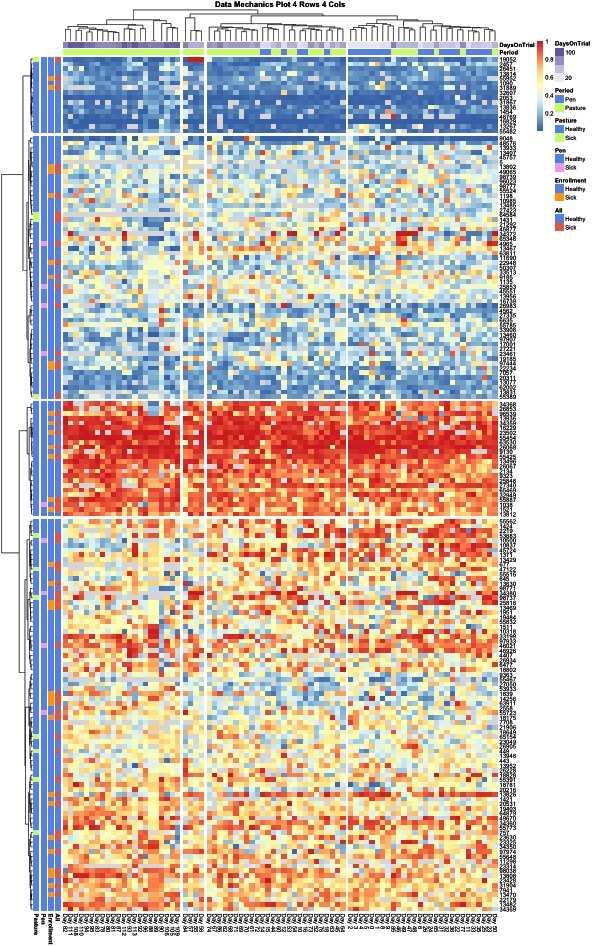

Supplement: Supplementary file 5 [file Data_Sheet_5.ZIP › Grid_LR/DatMechPlotOutR4C4 _Final.jpeg]

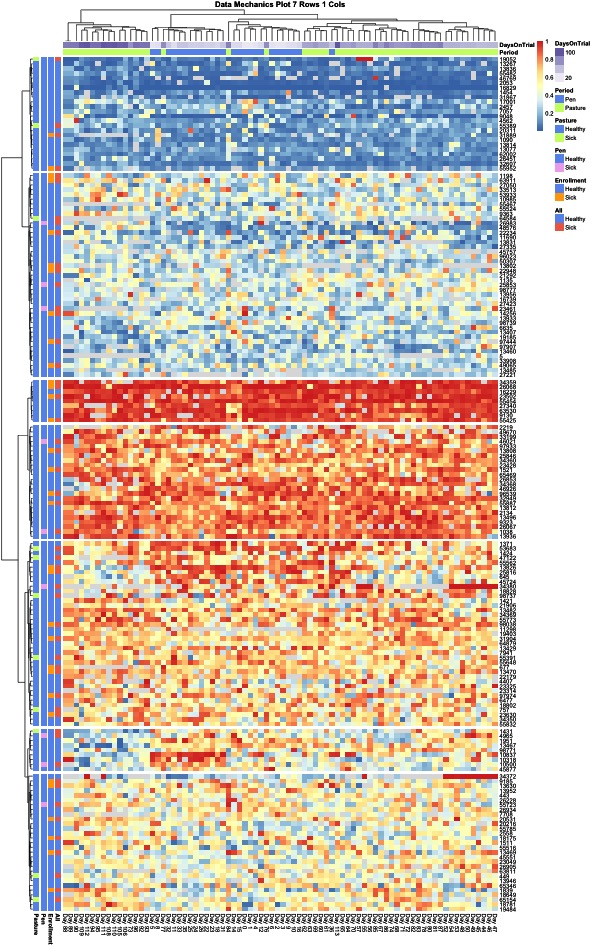

Supplement: Supplementary file 5 [file Data_Sheet_5.ZIP › Grid_LR/DatMechPlotOutR7C1 _Final.jpeg]

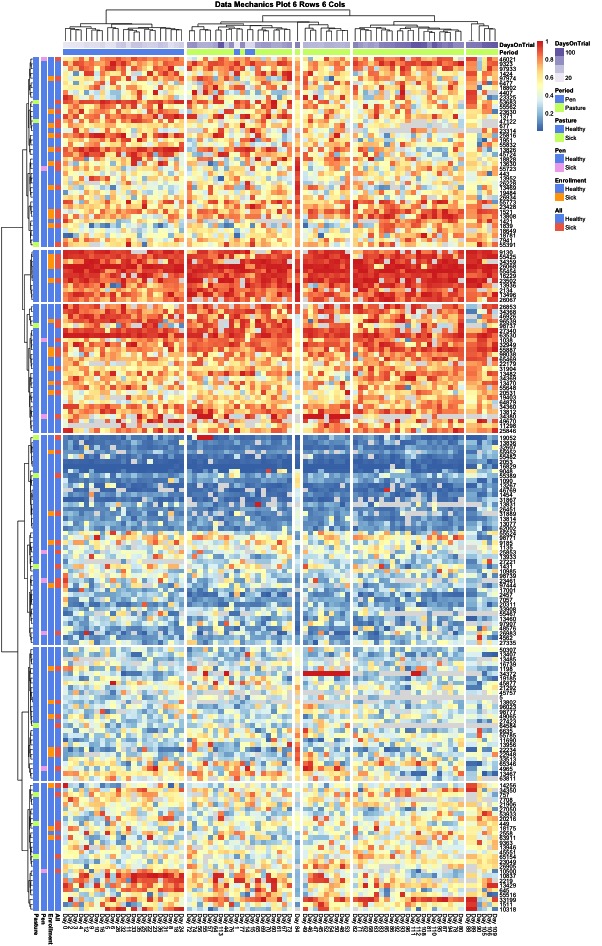

Supplement: Supplementary file 5 [file Data_Sheet_5.ZIP › Grid_LR/DatMechPlotOutR6C6 _Final.jpeg]

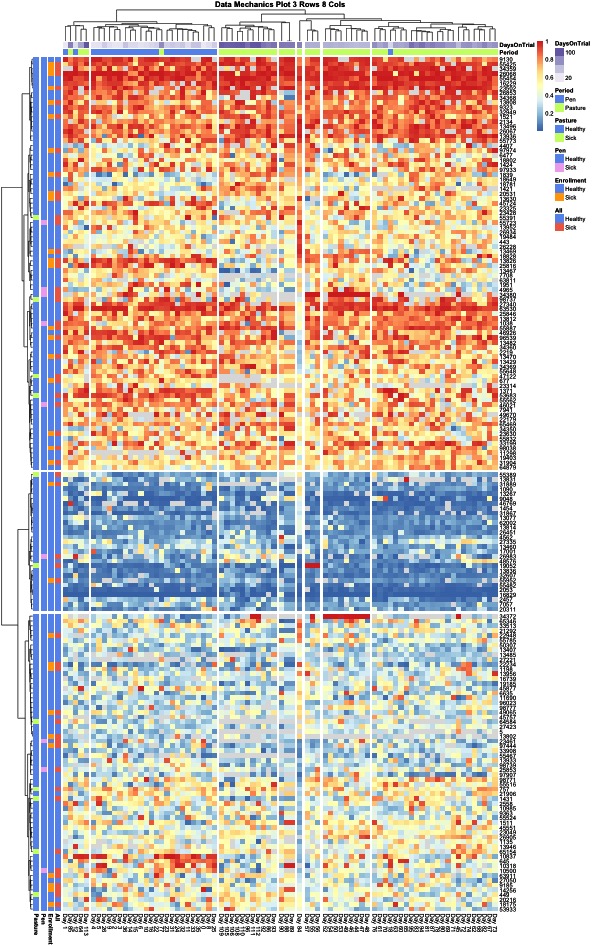

Supplement: Supplementary file 5 [file Data_Sheet_5.ZIP › Grid_LR/DatMechPlotOutR3C8 _Final.jpeg]

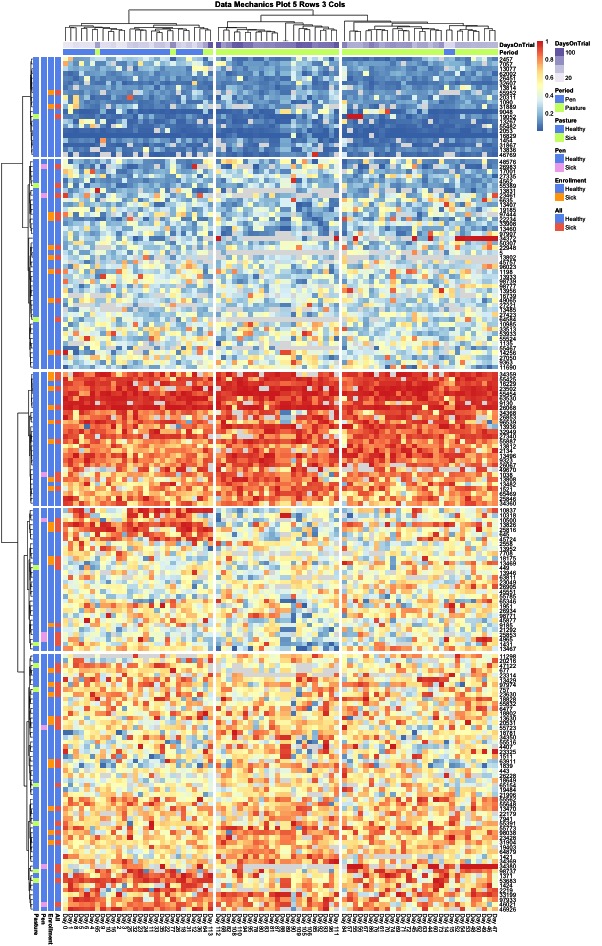

Supplement: Supplementary file 5 [file Data_Sheet_5.ZIP › Grid_LR/DatMechPlotOutR5C3 _Final.jpeg]

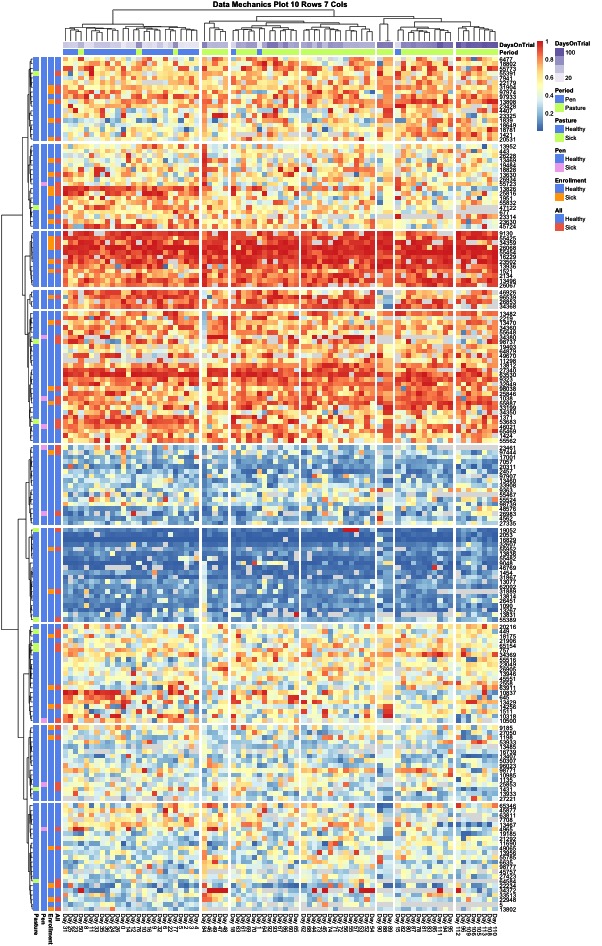

Supplement: Supplementary file 5 [file Data_Sheet_5.ZIP › Grid_LR/DatMechPlotOutR10C7 _Final.jpeg]

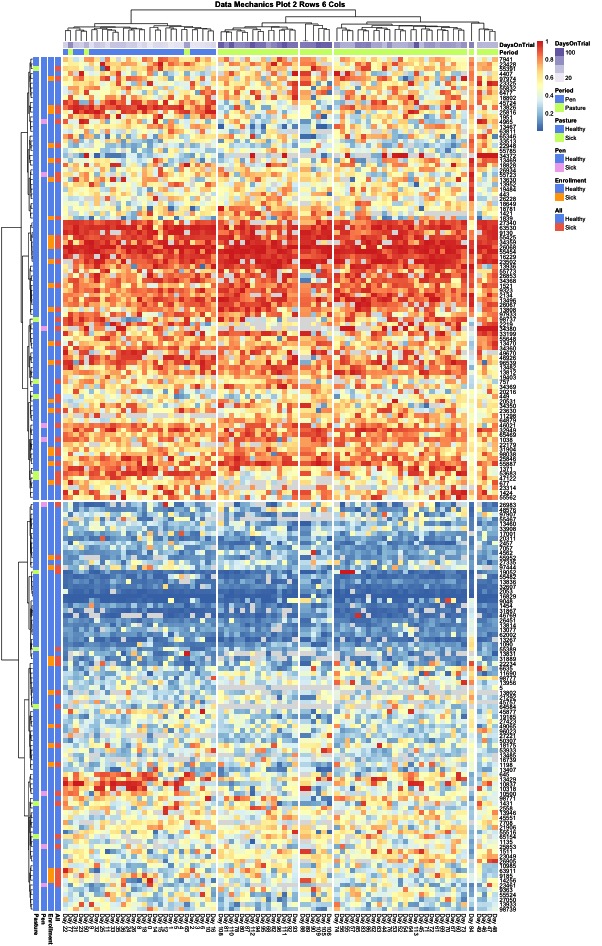

Supplement: Supplementary file 5 [file Data_Sheet_5.ZIP › Grid_LR/DatMechPlotOutR2C6 _Final.jpeg]

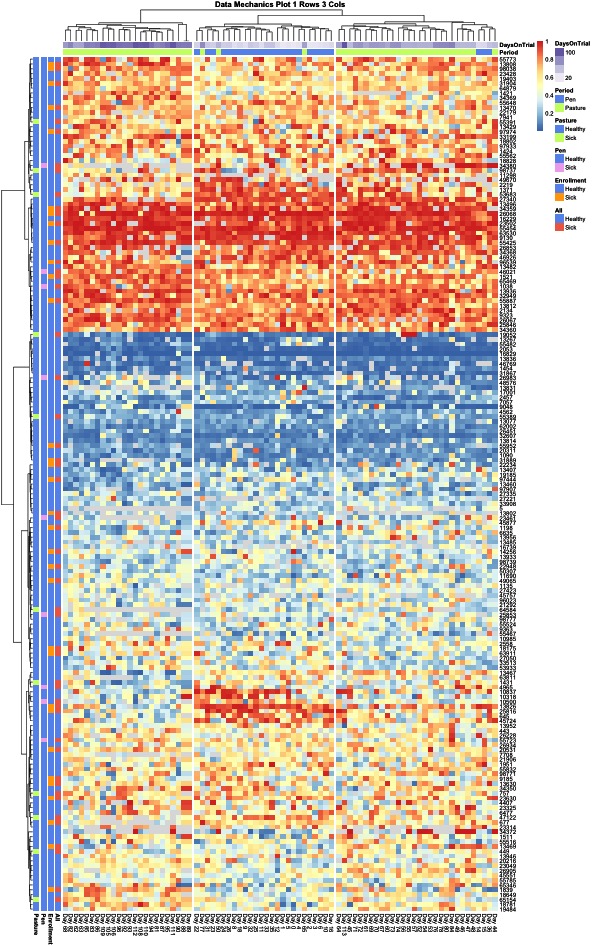

Supplement: Supplementary file 5 [file Data_Sheet_5.ZIP › Grid_LR/DatMechPlotOutR1C3 _Final.jpeg]

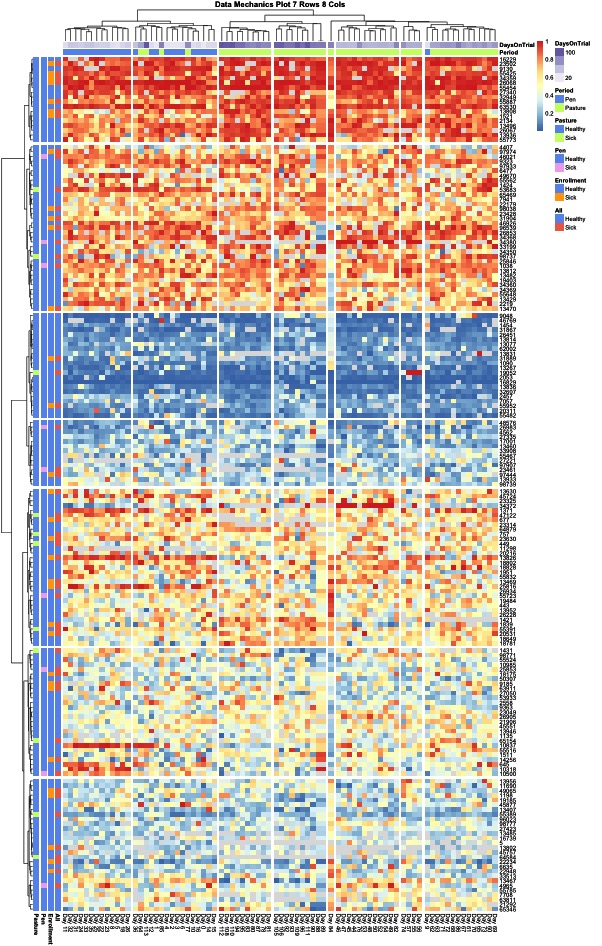

Supplement: Supplementary file 5 [file Data_Sheet_5.ZIP › Grid_LR/DatMechPlotOutR7C8 _Final.jpeg]

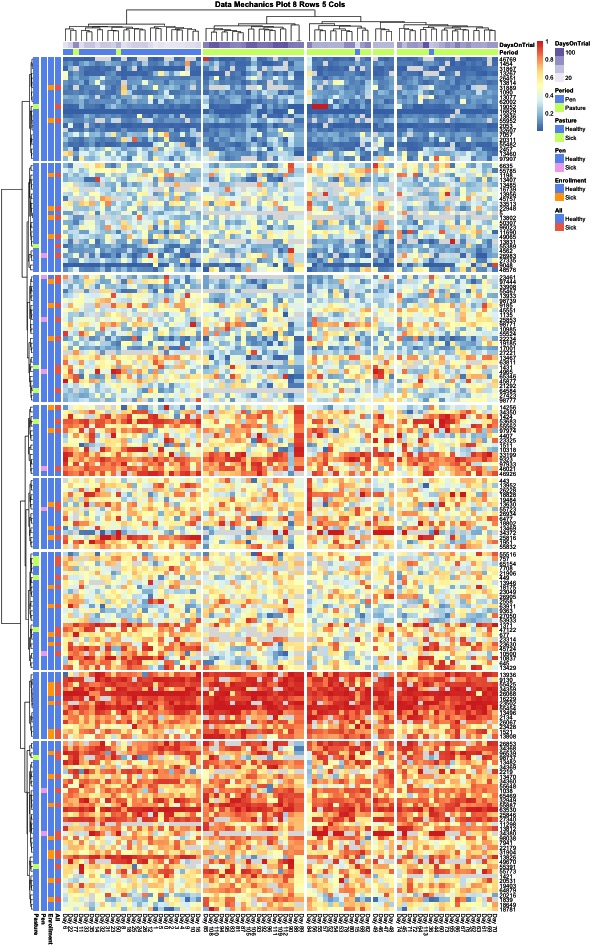

Supplement: Supplementary file 5 [file Data_Sheet_5.ZIP › Grid_LR/DatMechPlotOutR8C5 _Final.jpeg]

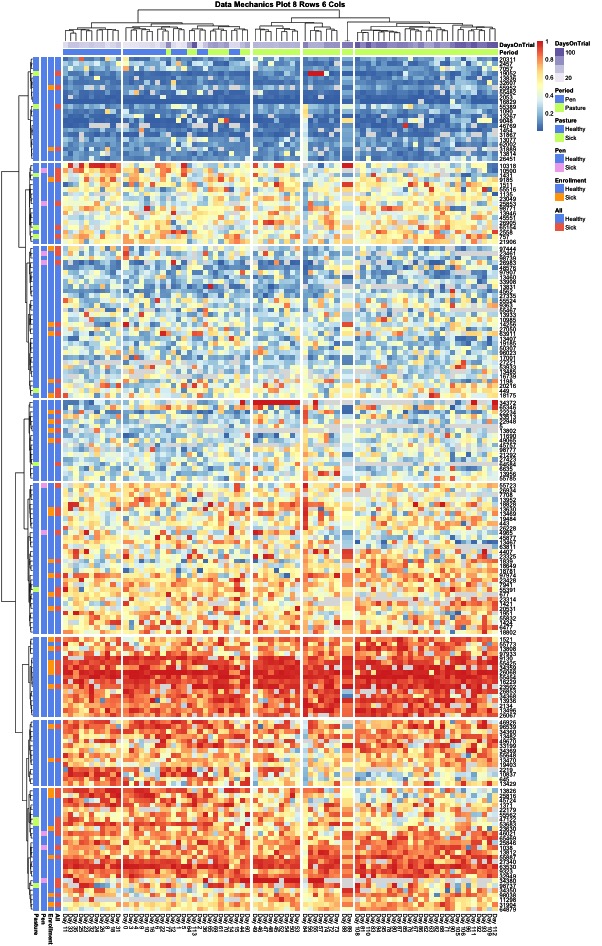

Supplement: Supplementary file 5 [file Data_Sheet_5.ZIP › Grid_LR/DatMechPlotOutR8C6 _Final.jpeg]

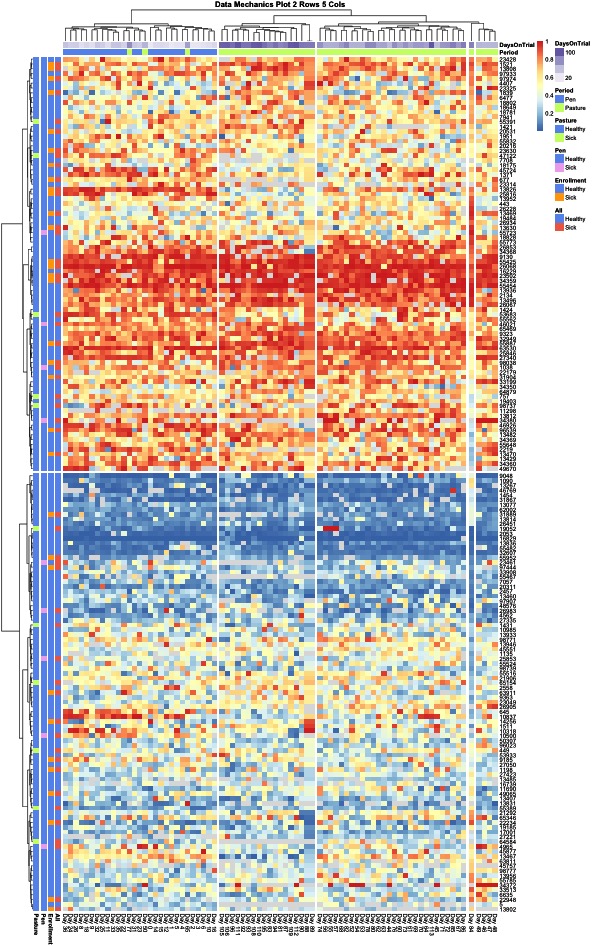

Supplement: Supplementary file 5 [file Data_Sheet_5.ZIP › Grid_LR/DatMechPlotOutR2C5 _Final.jpeg]

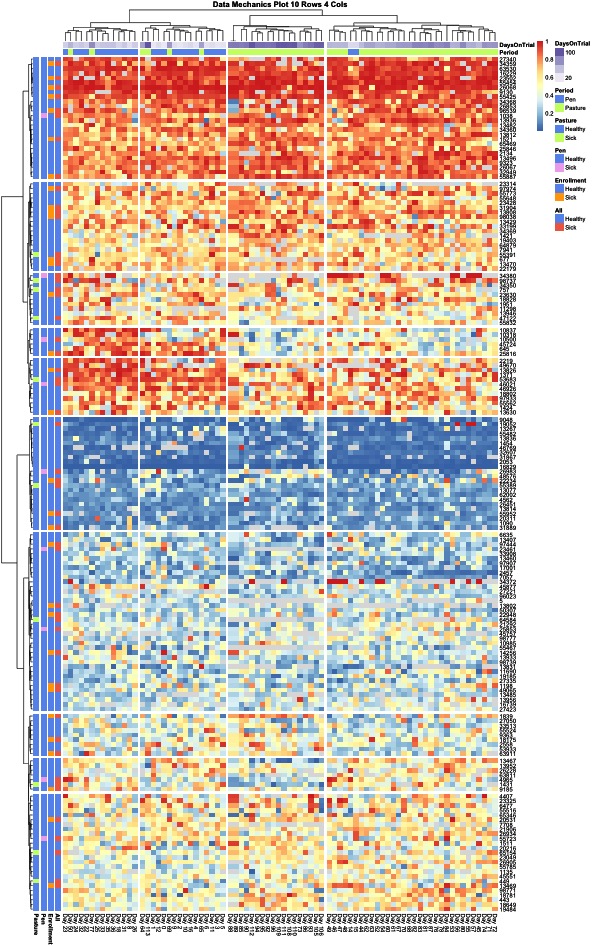

Supplement: Supplementary file 5 [file Data_Sheet_5.ZIP › Grid_LR/DatMechPlotOutR10C4 _Final.jpeg]

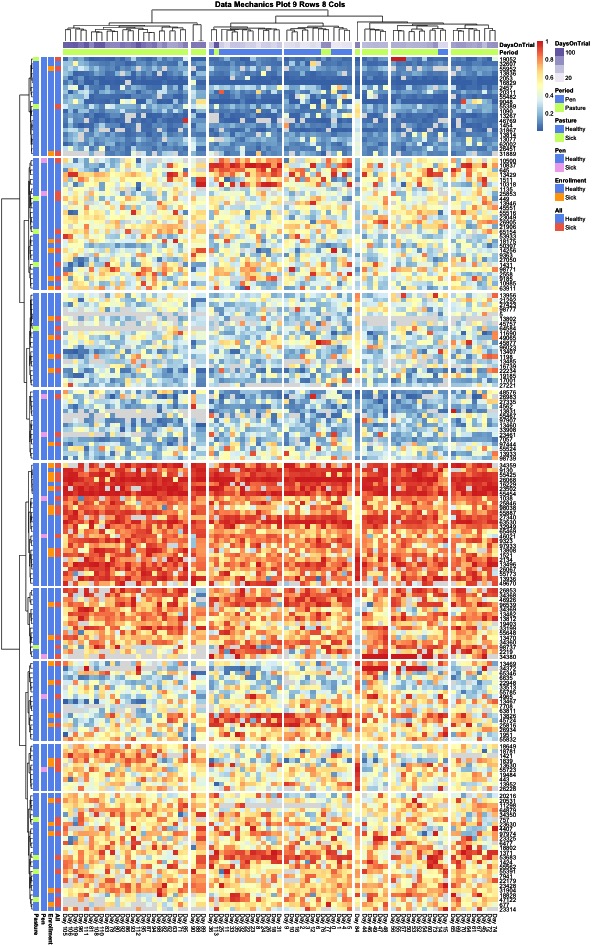

Supplement: Supplementary file 5 [file Data_Sheet_5.ZIP › Grid_LR/DatMechPlotOutR9C8 _Final.jpeg]

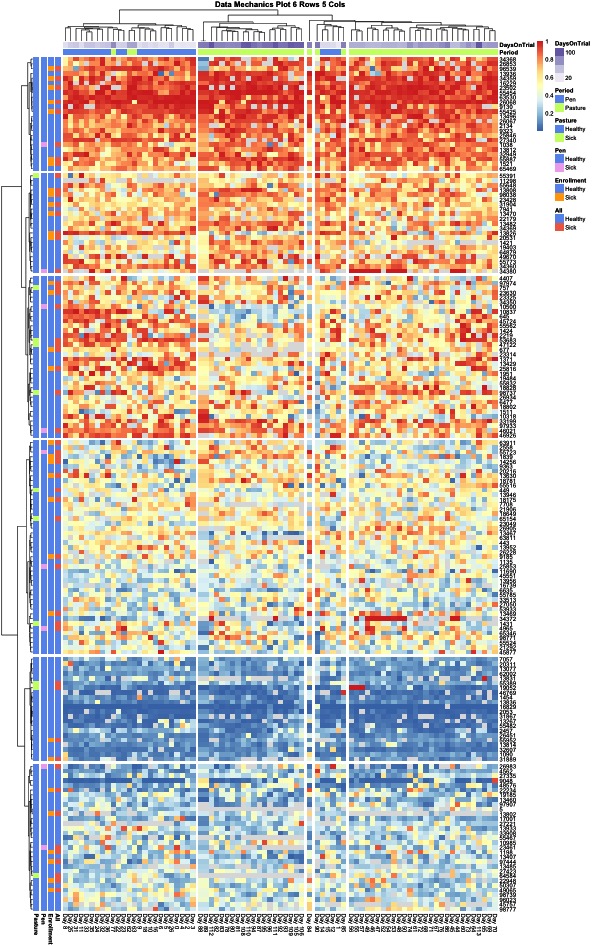

Supplement: Supplementary file 5 [file Data_Sheet_5.ZIP › Grid_LR/DatMechPlotOutR6C5 _Final.jpeg]

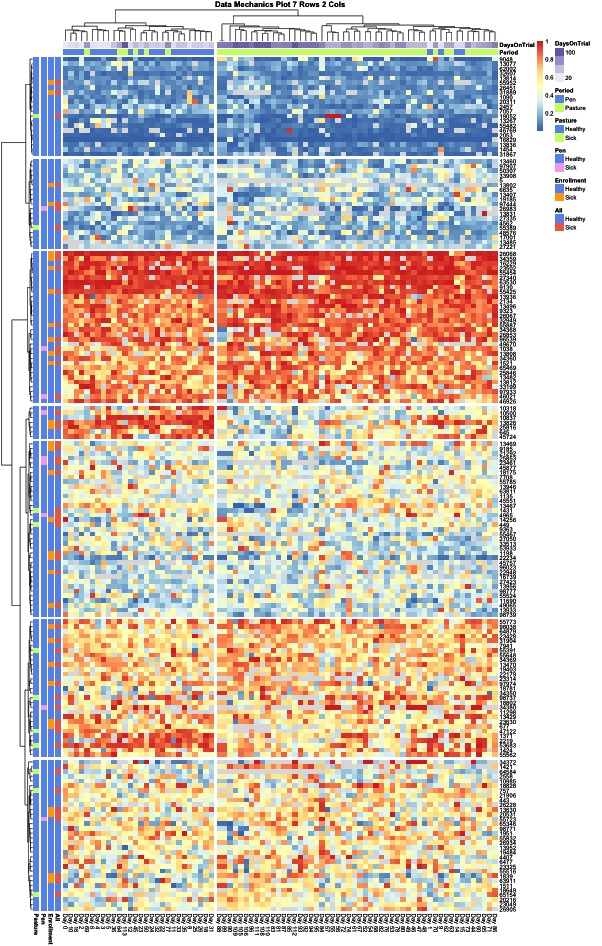

Supplement: Supplementary file 5 [file Data_Sheet_5.ZIP › Grid_LR/DatMechPlotOutR7C2 _Final.jpeg]

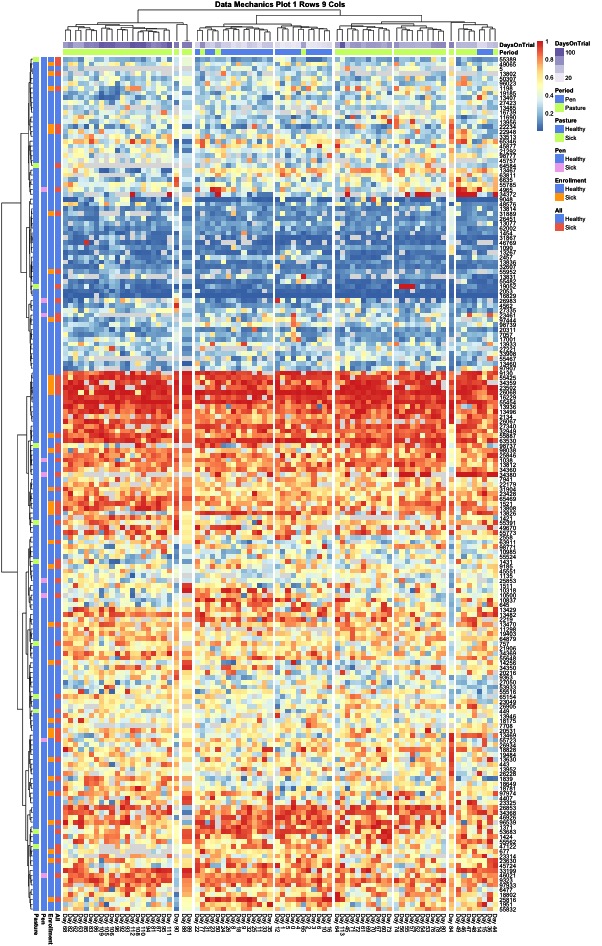

Supplement: Supplementary file 5 [file Data_Sheet_5.ZIP › Grid_LR/DatMechPlotOutR1C9 _Final.jpeg]

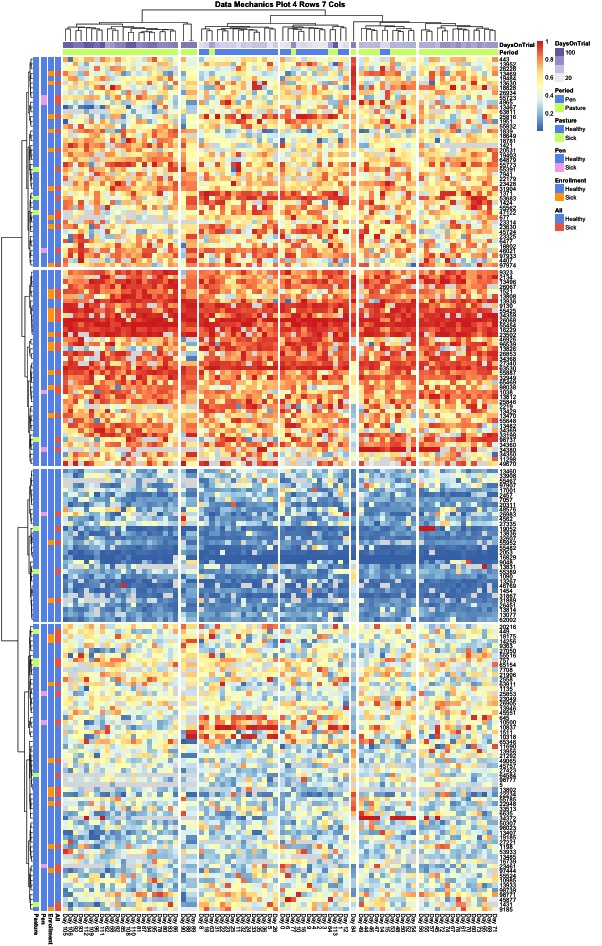

Supplement: Supplementary file 5 [file Data_Sheet_5.ZIP › Grid_LR/DatMechPlotOutR4C7 _Final.jpeg]

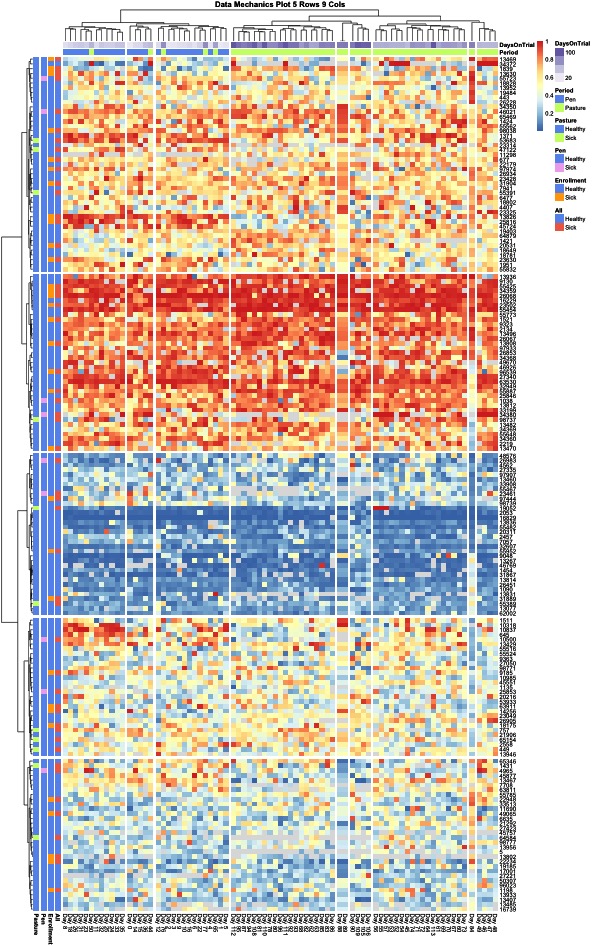

Supplement: Supplementary file 5 [file Data_Sheet_5.ZIP › Grid_LR/DatMechPlotOutR5C9 _Final.jpeg]

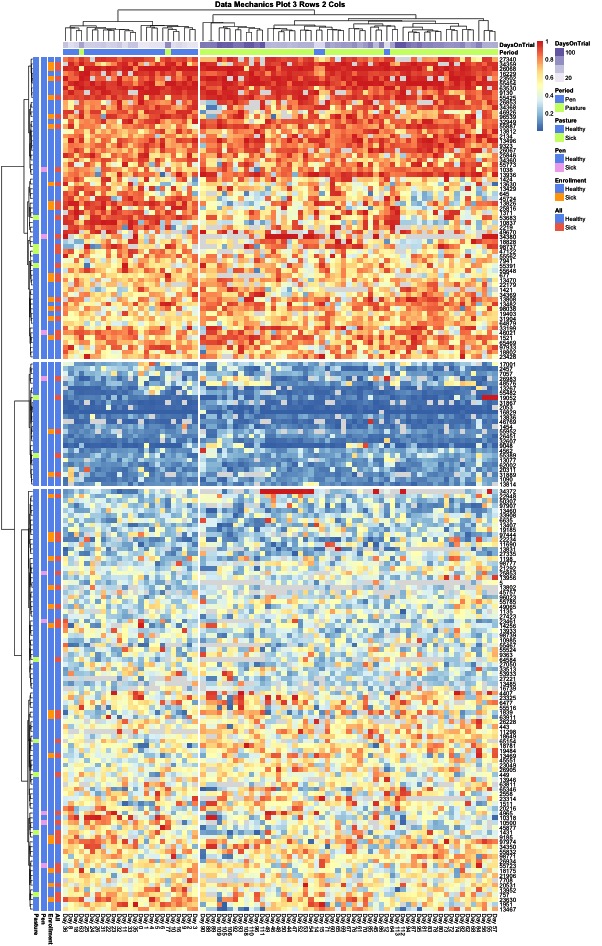

Supplement: Supplementary file 5 [file Data_Sheet_5.ZIP › Grid_LR/DatMechPlotOutR3C2 _Final.jpeg]

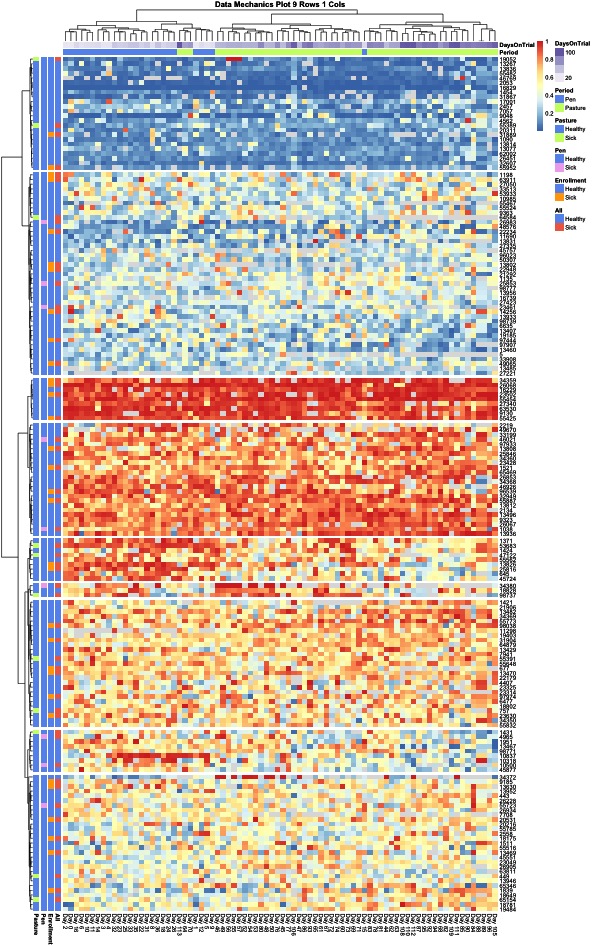

Supplement: Supplementary file 5 [file Data_Sheet_5.ZIP › Grid_LR/DatMechPlotOutR9C1 _Final.jpeg]

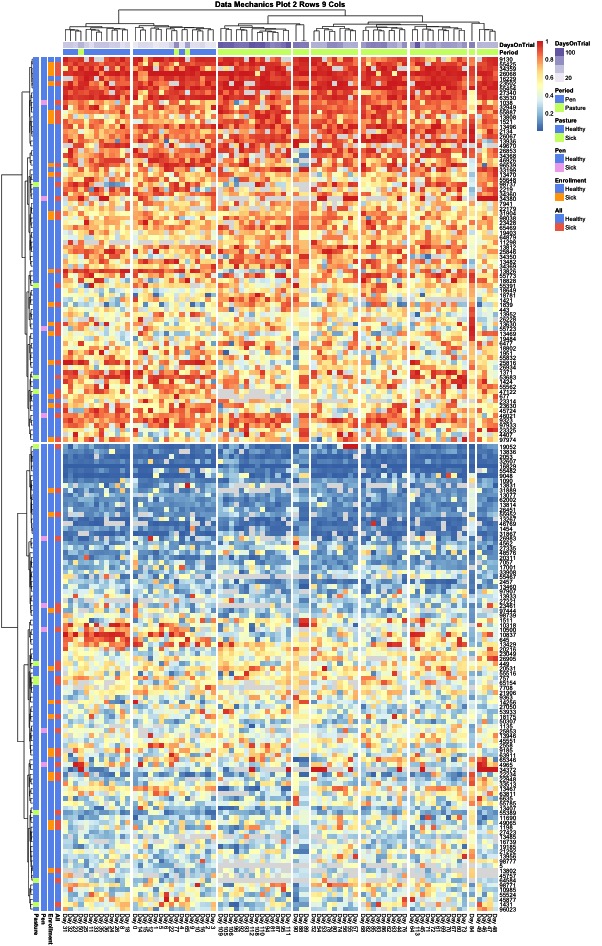

Supplement: Supplementary file 5 [file Data_Sheet_5.ZIP › Grid_LR/DatMechPlotOutR2C9 _Final.jpeg]

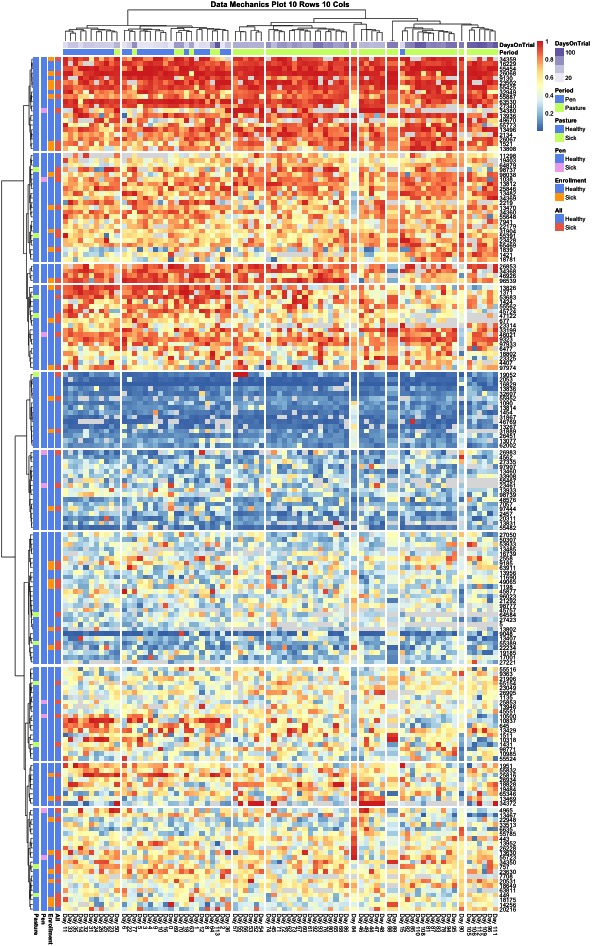

Supplement: Supplementary file 5 [file Data_Sheet_5.ZIP › Grid_LR/DatMechPlotOutR10C10 _Final.jpeg]

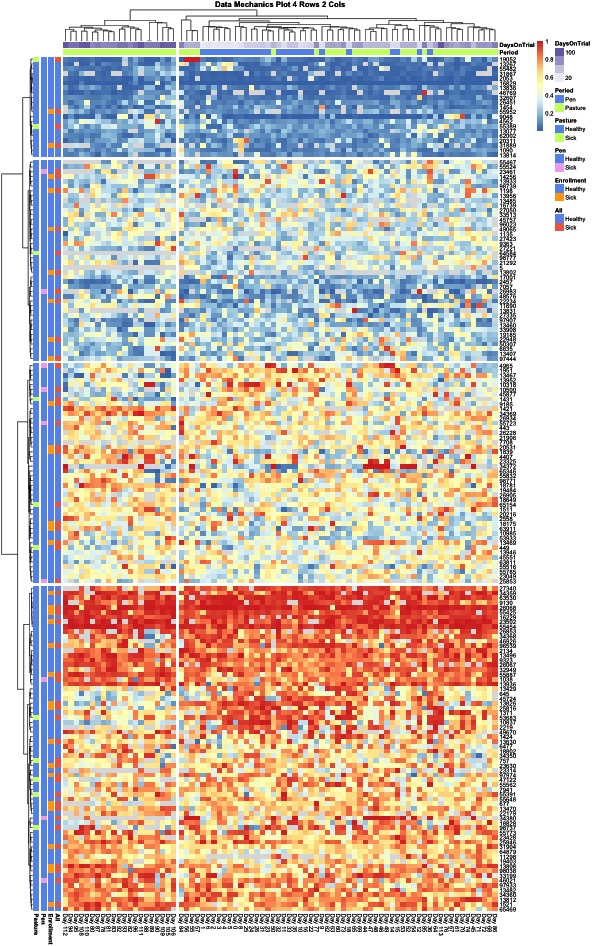

Supplement: Supplementary file 5 [file Data_Sheet_5.ZIP › Grid_LR/DatMechPlotOutR4C2 _Final.jpeg]

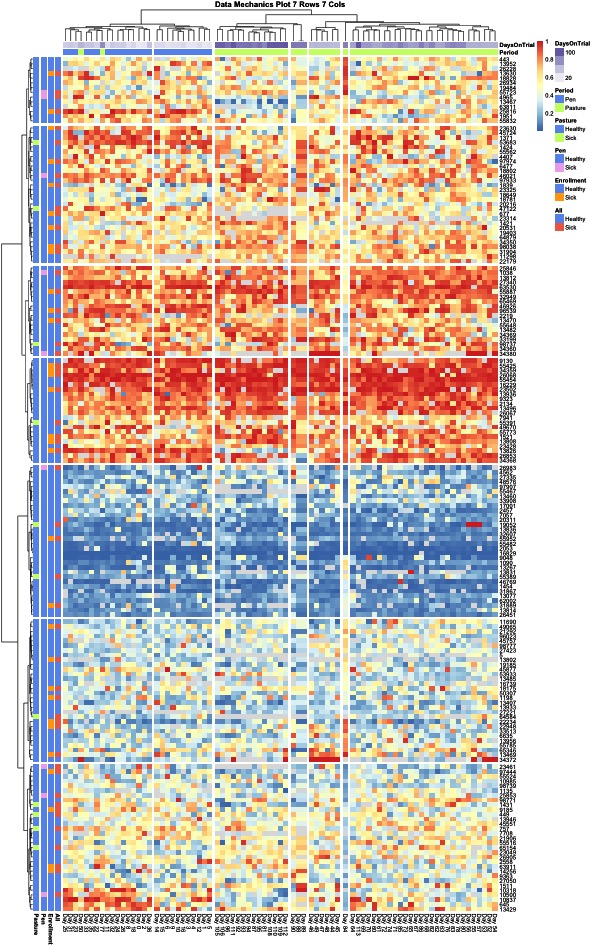

Supplement: Supplementary file 5 [file Data_Sheet_5.ZIP › Grid_LR/DatMechPlotOutR7C7 _Final.jpeg]

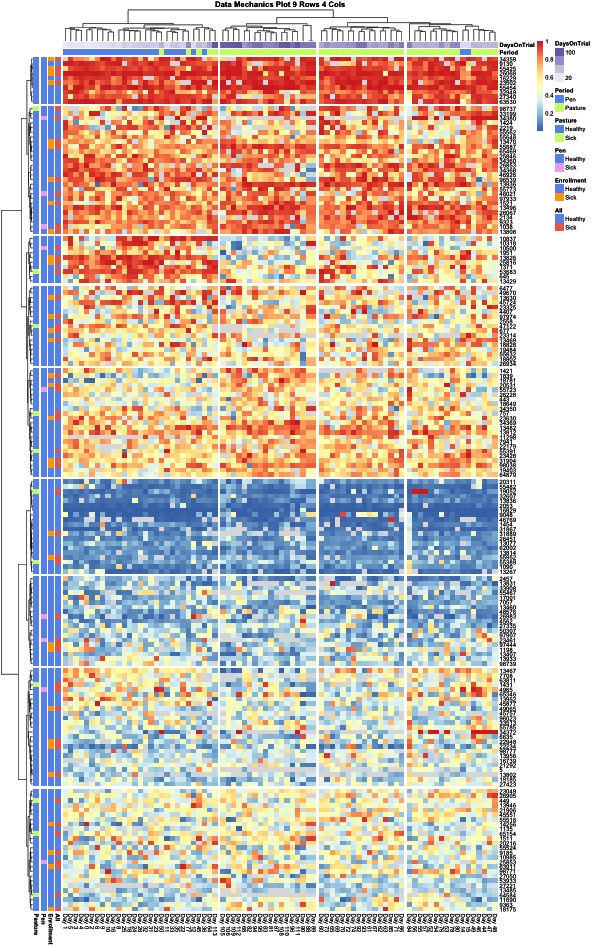

Supplement: Supplementary file 5 [file Data_Sheet_5.ZIP › Grid_LR/DatMechPlotOutR9C4 _Final.jpeg]

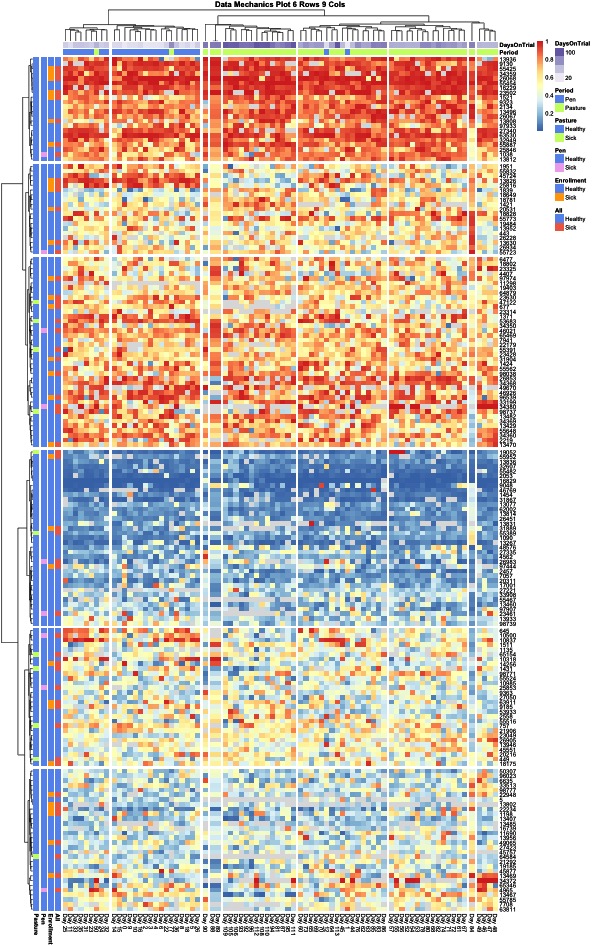

Supplement: Supplementary file 5 [file Data_Sheet_5.ZIP › Grid_LR/DatMechPlotOutR6C9 _Final.jpeg]

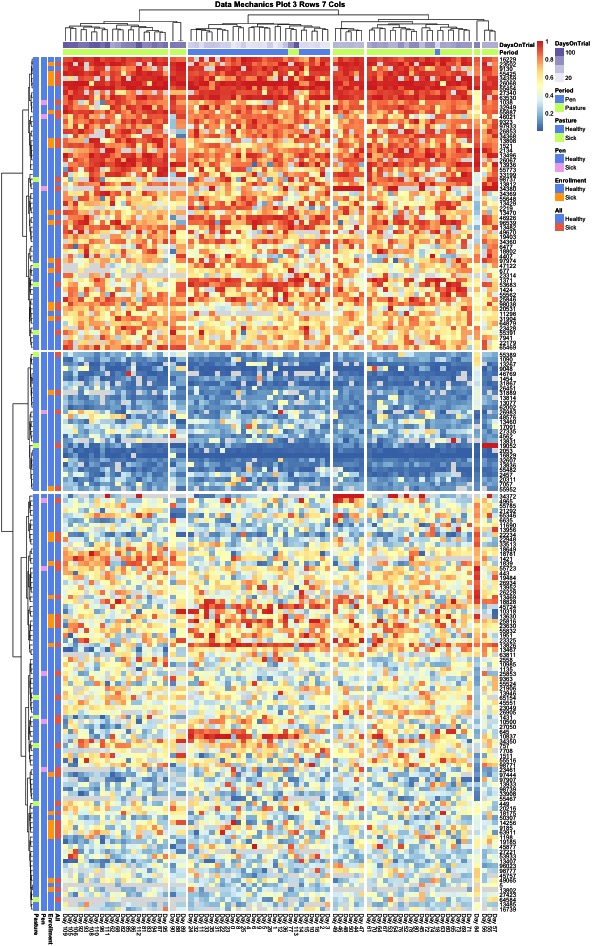

Supplement: Supplementary file 5 [file Data_Sheet_5.ZIP › Grid_LR/DatMechPlotOutR3C7 _Final.jpeg]

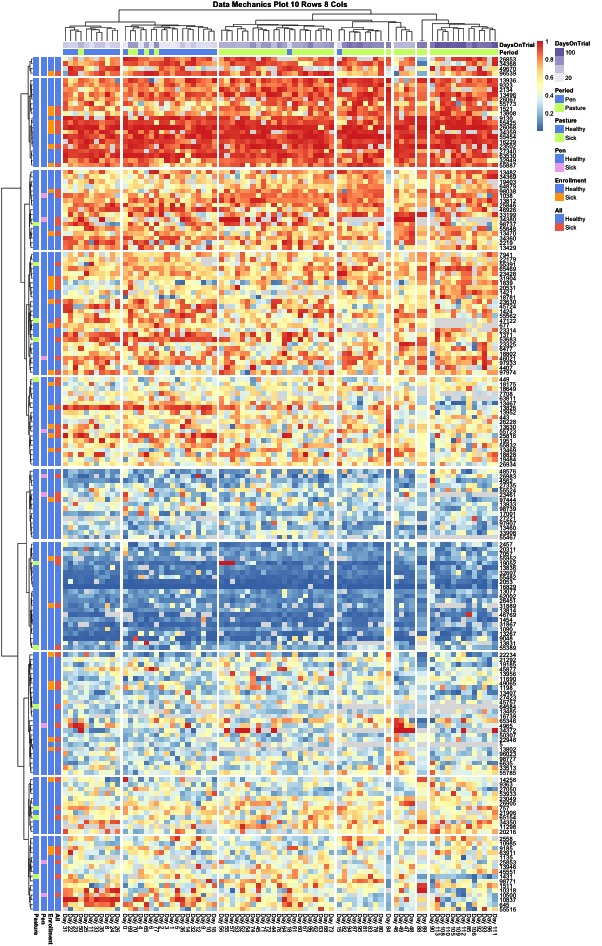

Supplement: Supplementary file 5 [file Data_Sheet_5.ZIP › Grid_LR/DatMechPlotOutR10C8 _Final.jpeg]

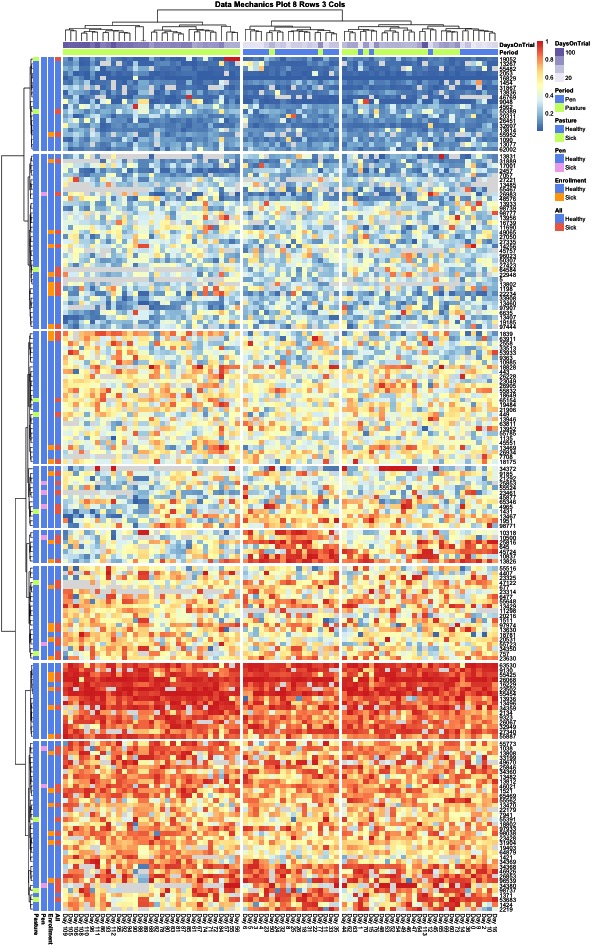

Supplement: Supplementary file 5 [file Data_Sheet_5.ZIP › Grid_LR/DatMechPlotOutR8C3 _Final.jpeg]

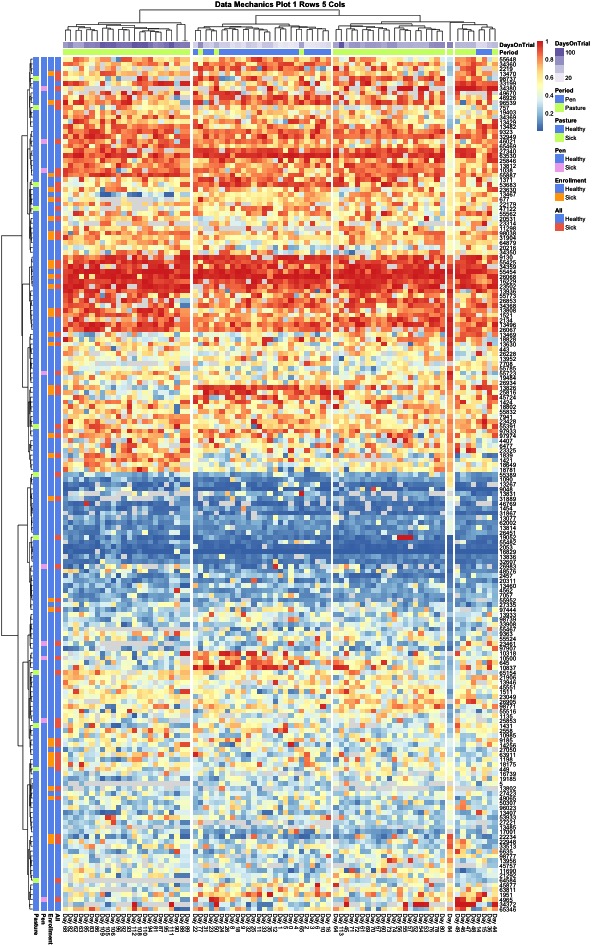

Supplement: Supplementary file 5 [file Data_Sheet_5.ZIP › Grid_LR/DatMechPlotOutR1C5 _Final.jpeg]

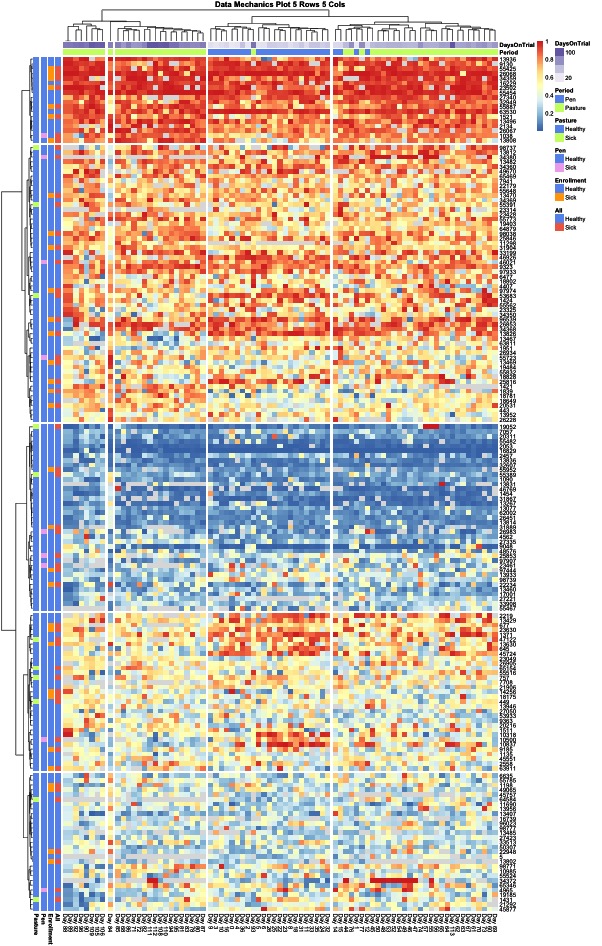

Supplement: Supplementary file 5 [file Data_Sheet_5.ZIP › Grid_LR/DatMechPlotOutR5C5 _Final.jpeg]

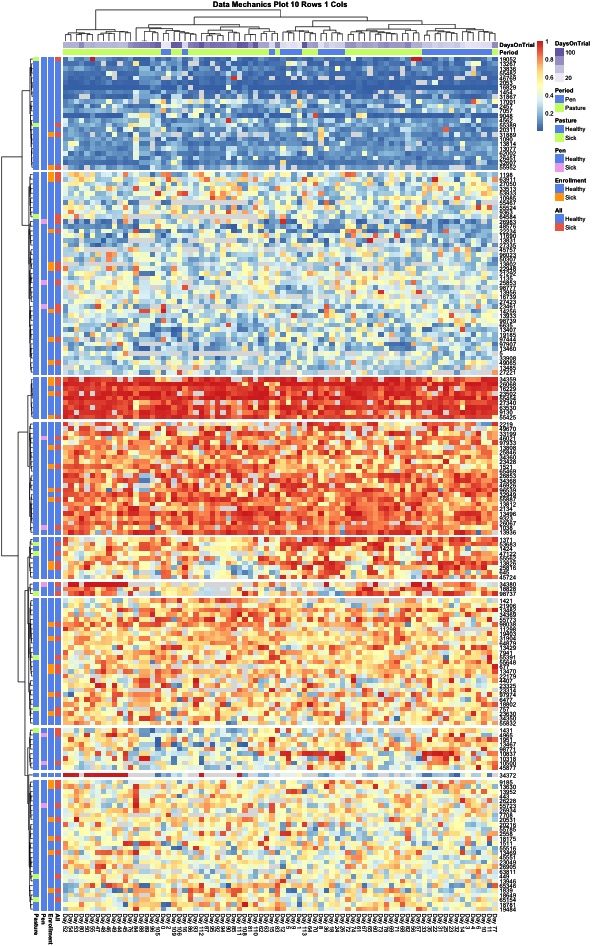

Supplement: Supplementary file 5 [file Data_Sheet_5.ZIP › Grid_LR/DatMechPlotOutR10C1 _Final.jpeg]

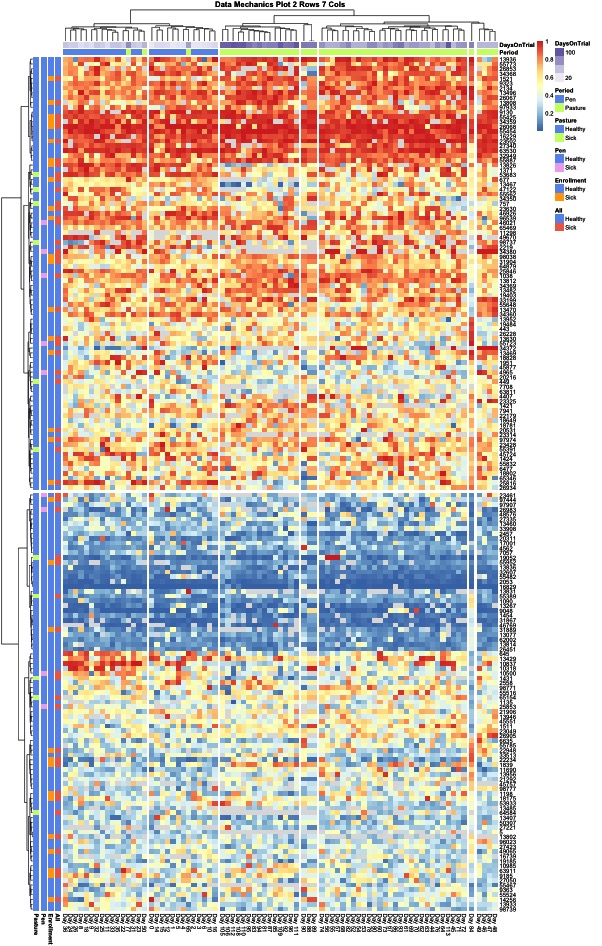

Supplement: Supplementary file 5 [file Data_Sheet_5.ZIP › Grid_LR/DatMechPlotOutR2C7 _Final.jpeg]

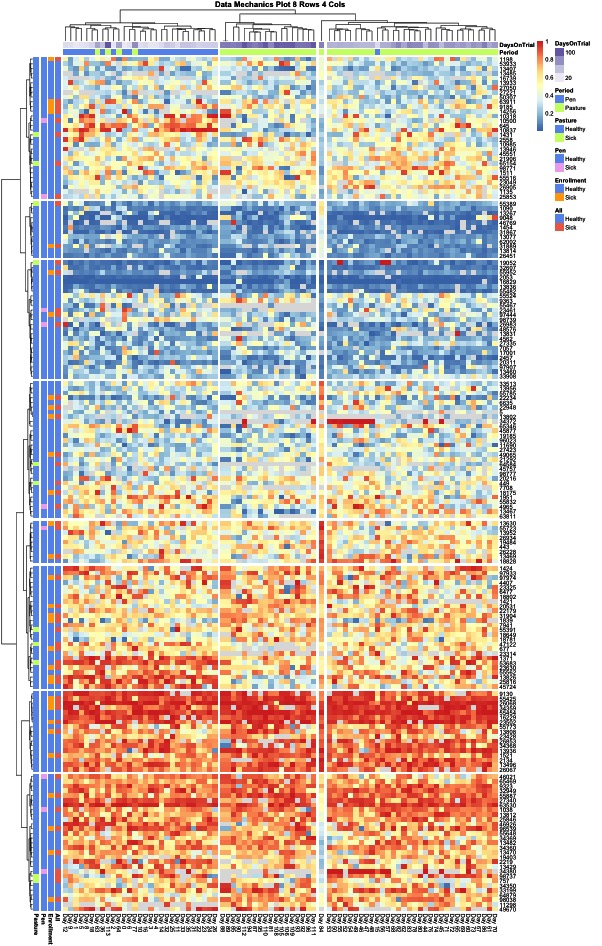

Supplement: Supplementary file 5 [file Data_Sheet_5.ZIP › Grid_LR/DatMechPlotOutR8C4 _Final.jpeg]

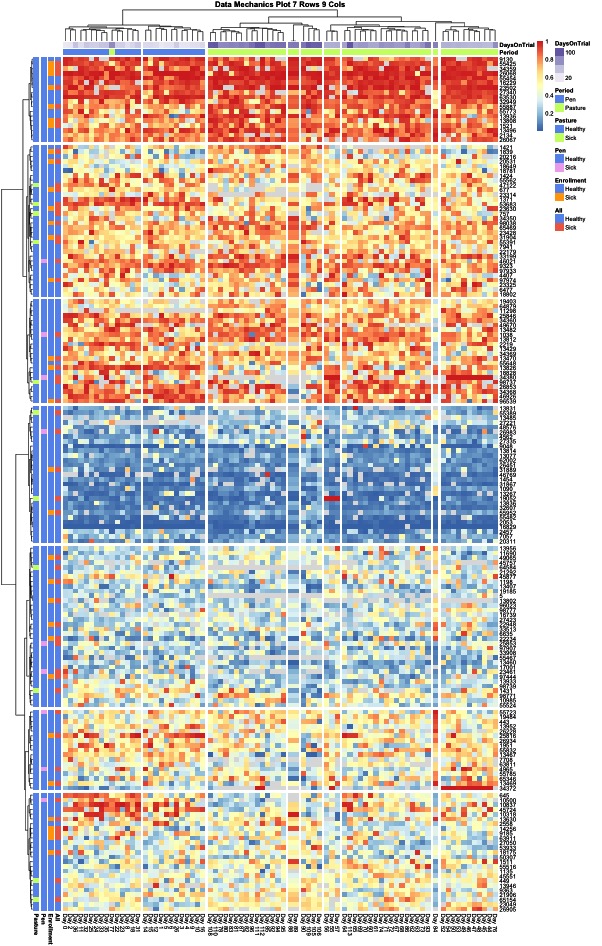

Supplement: Supplementary file 5 [file Data_Sheet_5.ZIP › Grid_LR/DatMechPlotOutR7C9 _Final.jpeg]

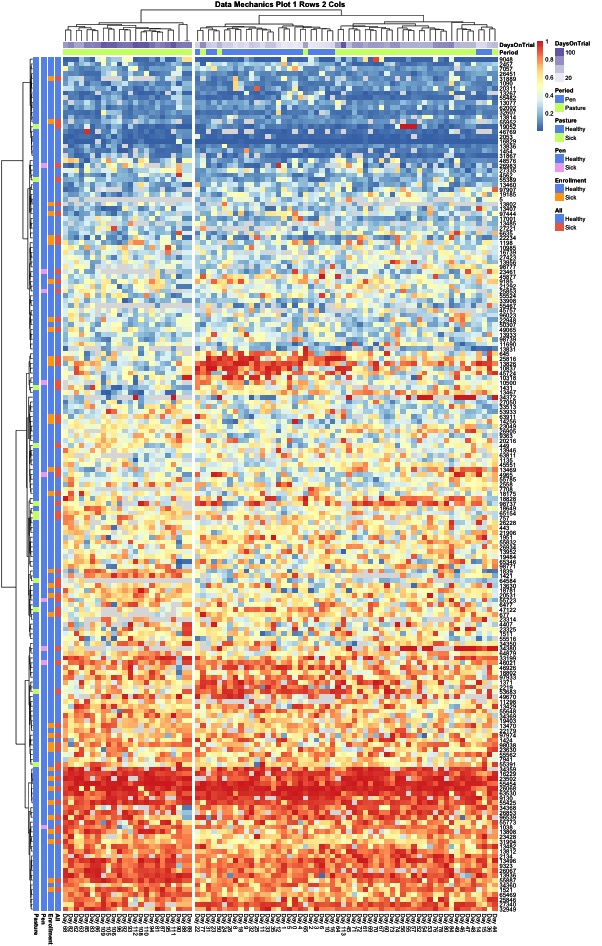

Supplement: Supplementary file 5 [file Data_Sheet_5.ZIP › Grid_LR/DatMechPlotOutR1C2 _Final.jpeg]

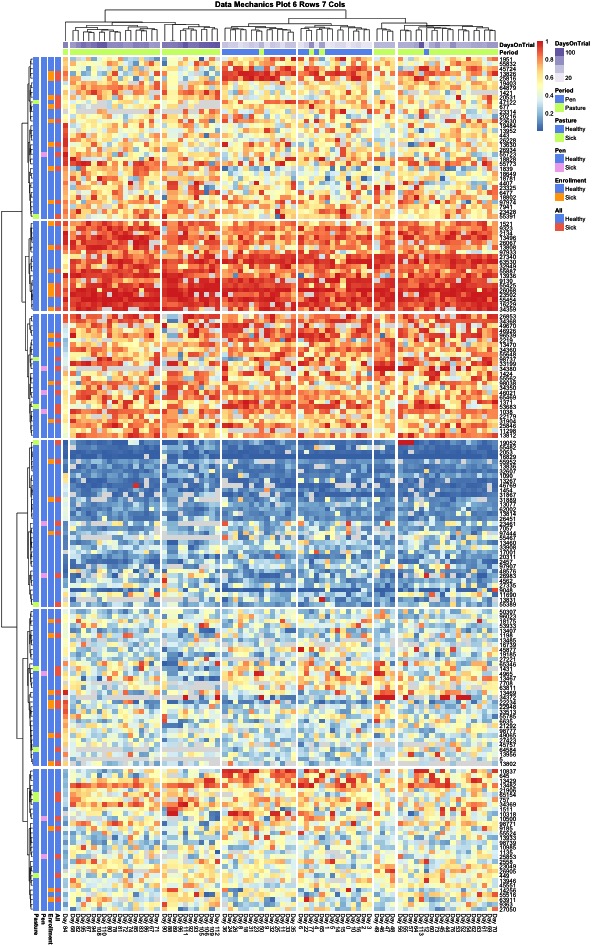

Supplement: Supplementary file 5 [file Data_Sheet_5.ZIP › Grid_LR/DatMechPlotOutR6C7 _Final.jpeg]

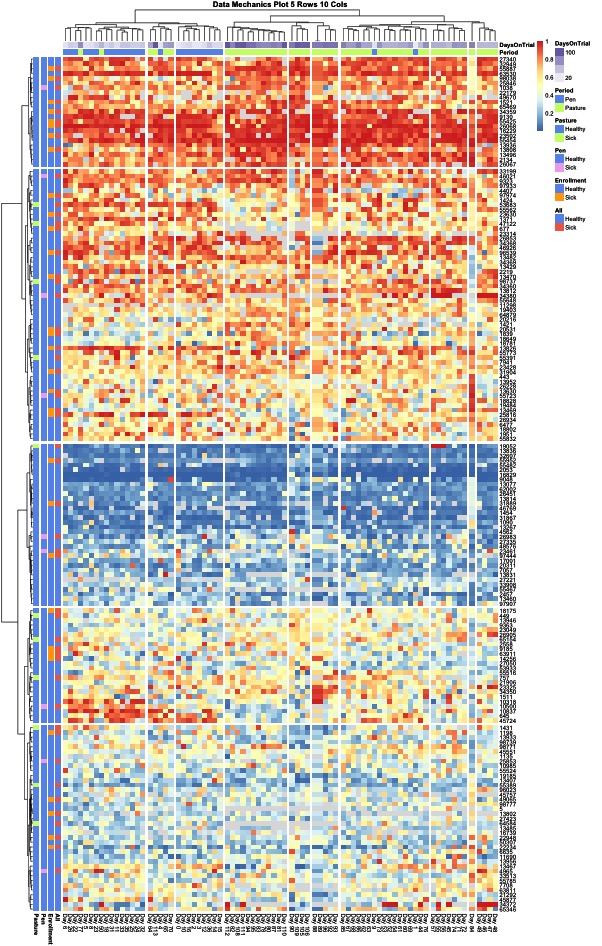

Supplement: Supplementary file 5 [file Data_Sheet_5.ZIP › Grid_LR/DatMechPlotOutR5C10 _Final.jpeg]

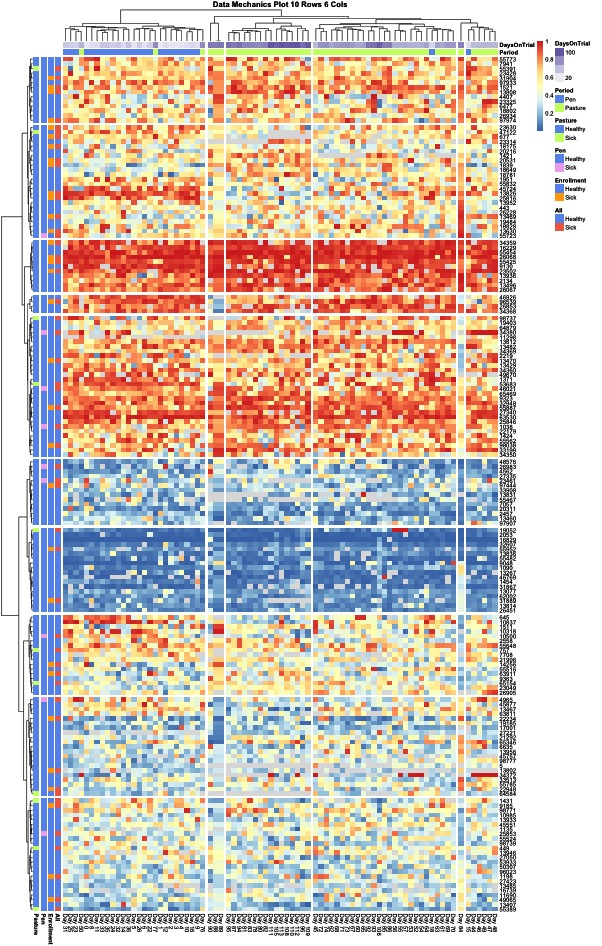

Supplement: Supplementary file 5 [file Data_Sheet_5.ZIP › Grid_LR/DatMechPlotOutR10C6 _Final.jpeg]

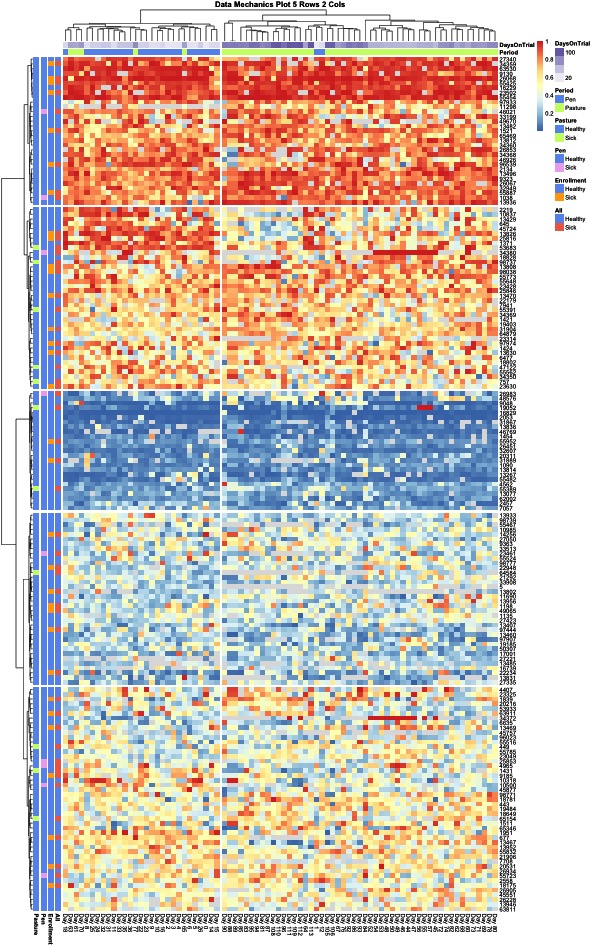

Supplement: Supplementary file 5 [file Data_Sheet_5.ZIP › Grid_LR/DatMechPlotOutR5C2 _Final.jpeg]

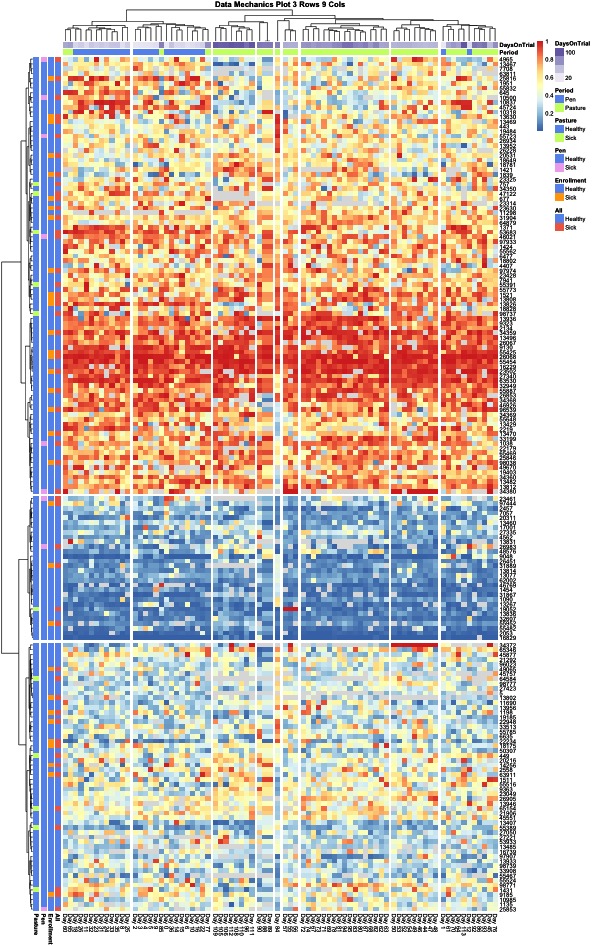

Supplement: Supplementary file 5 [file Data_Sheet_5.ZIP › Grid_LR/DatMechPlotOutR3C9 _Final.jpeg]

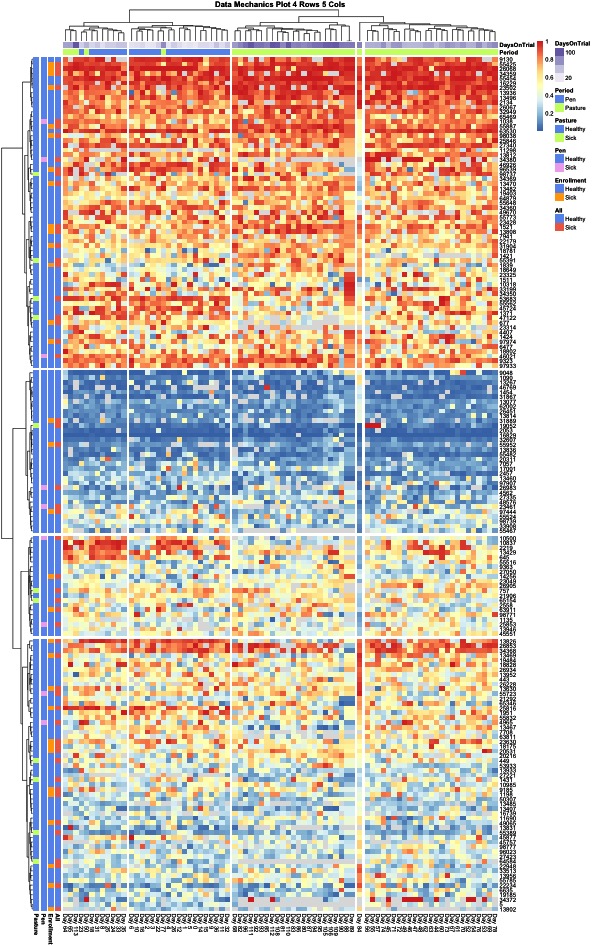

Supplement: Supplementary file 5 [file Data_Sheet_5.ZIP › Grid_LR/DatMechPlotOutR4C5 _Final.jpeg]

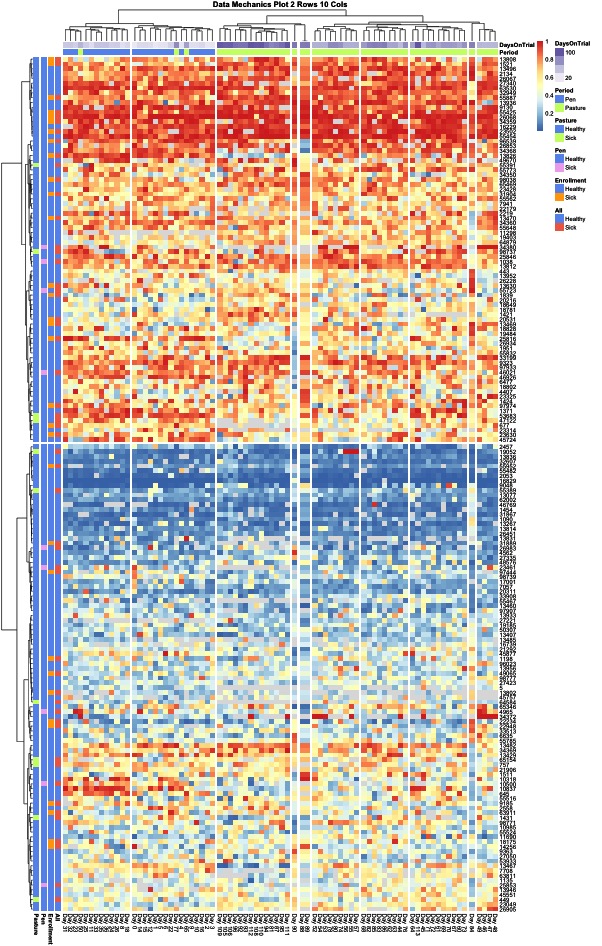

Supplement: Supplementary file 5 [file Data_Sheet_5.ZIP › Grid_LR/DatMechPlotOutR2C10 _Final.jpeg]

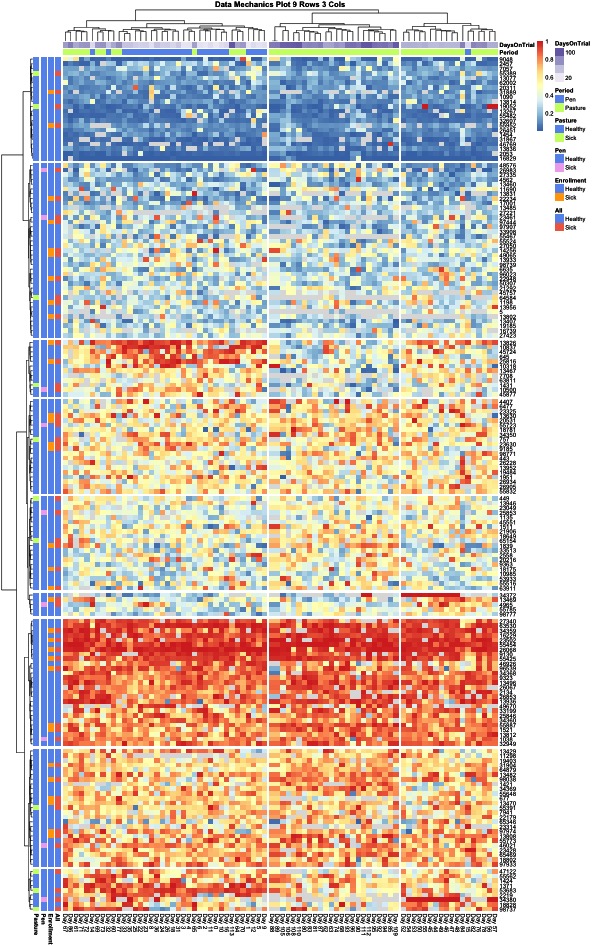

Supplement: Supplementary file 5 [file Data_Sheet_5.ZIP › Grid_LR/DatMechPlotOutR9C3 _Final.jpeg]

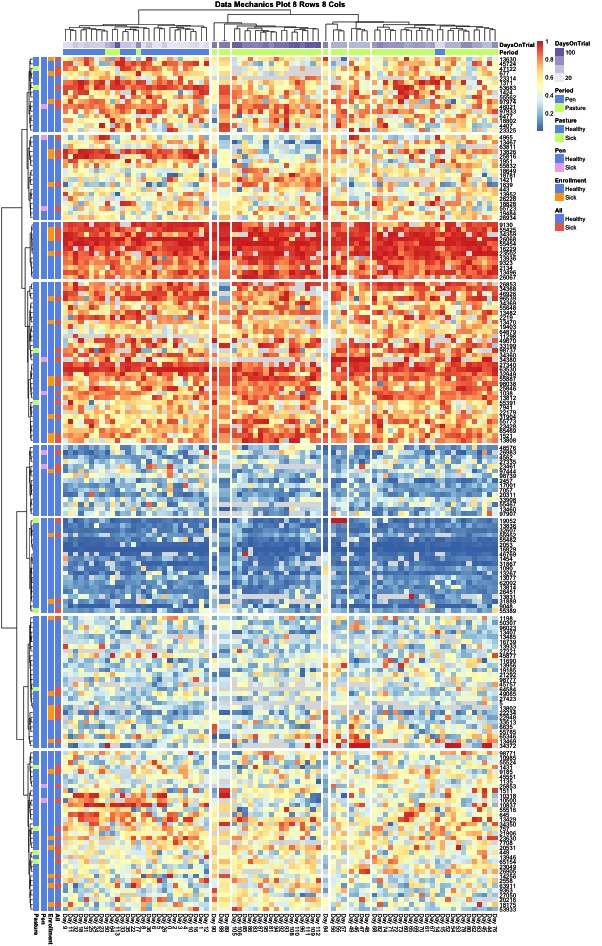

Supplement: Supplementary file 5 [file Data_Sheet_5.ZIP › Grid_LR/DatMechPlotOutR8C8 _Final.jpeg]

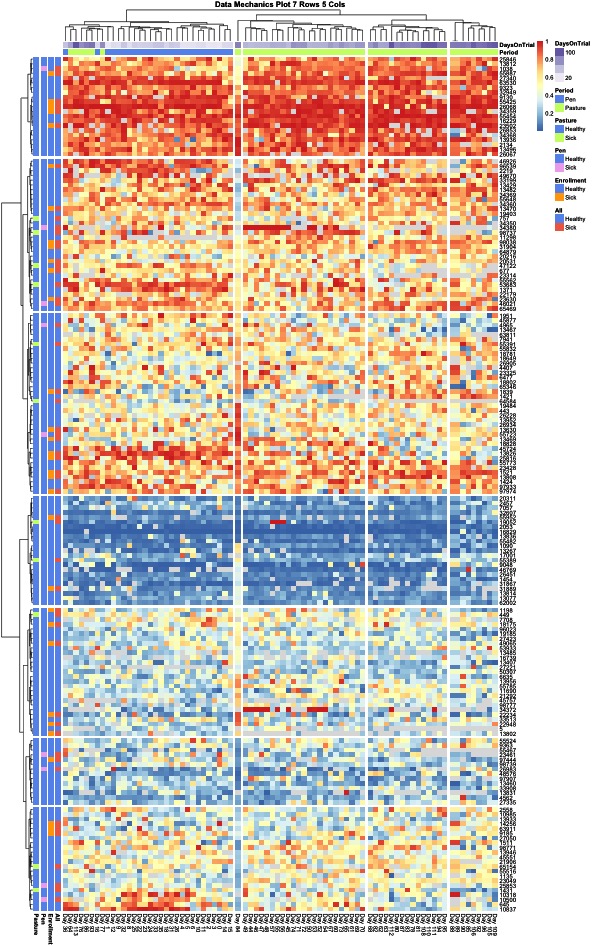

Supplement: Supplementary file 5 [file Data_Sheet_5.ZIP › Grid_LR/DatMechPlotOutR7C5 _Final.jpeg]

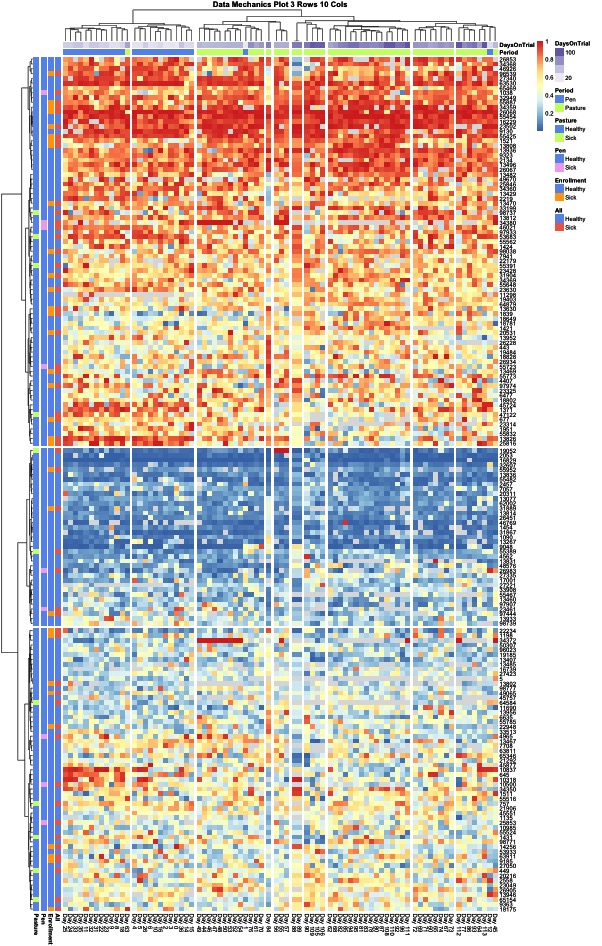

Supplement: Supplementary file 5 [file Data_Sheet_5.ZIP › Grid_LR/DatMechPlotOutR3C10 _Final.jpeg]

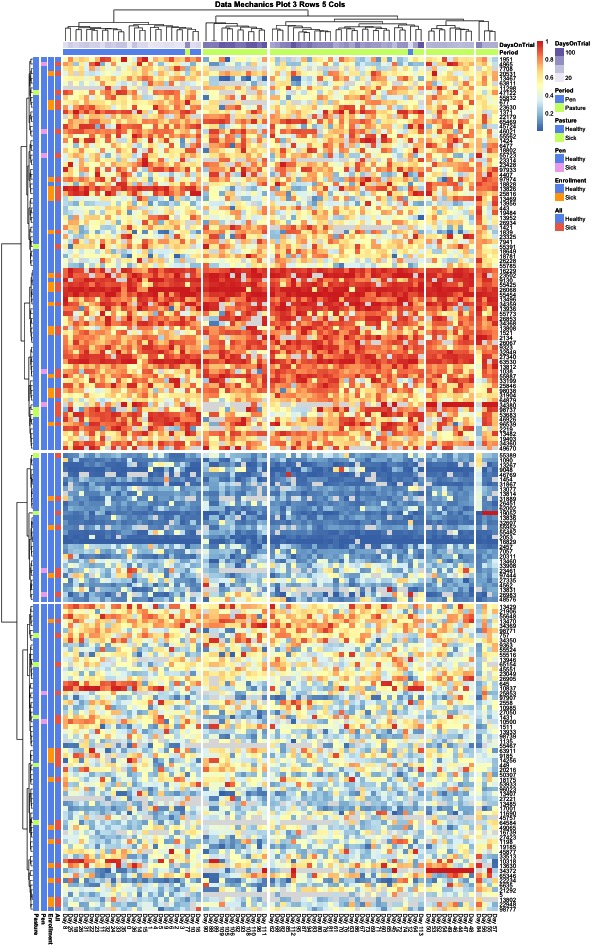

Supplement: Supplementary file 5 [file Data_Sheet_5.ZIP › Grid_LR/DatMechPlotOutR3C5 _Final.jpeg]

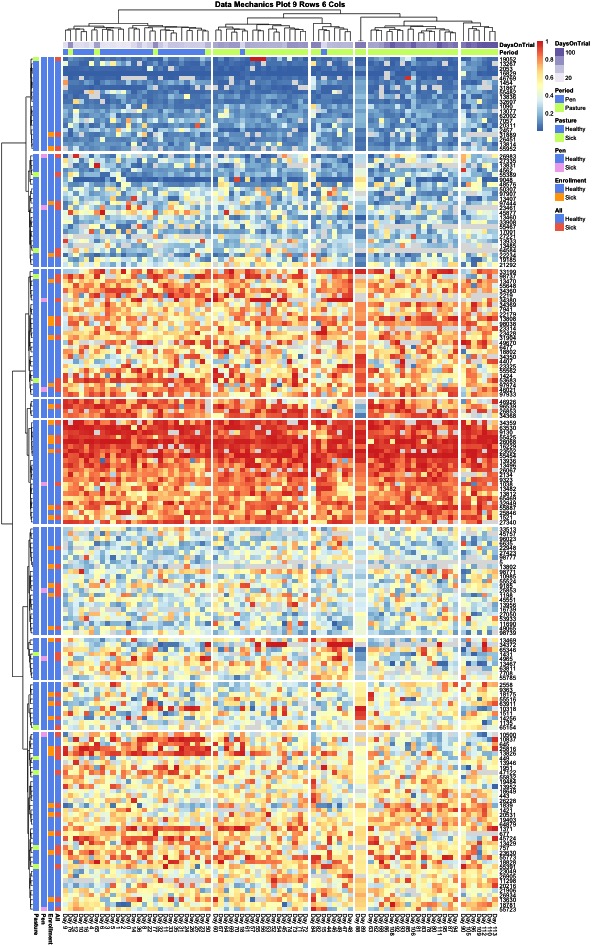

Supplement: Supplementary file 5 [file Data_Sheet_5.ZIP › Grid_LR/DatMechPlotOutR9C6 _Final.jpeg]

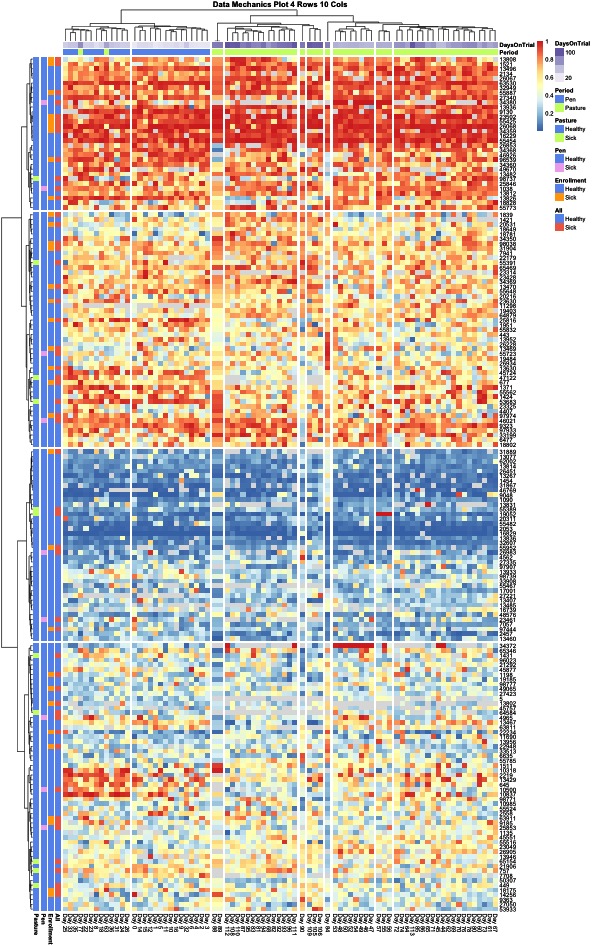

Supplement: Supplementary file 5 [file Data_Sheet_5.ZIP › Grid_LR/DatMechPlotOutR4C10 _Final.jpeg]

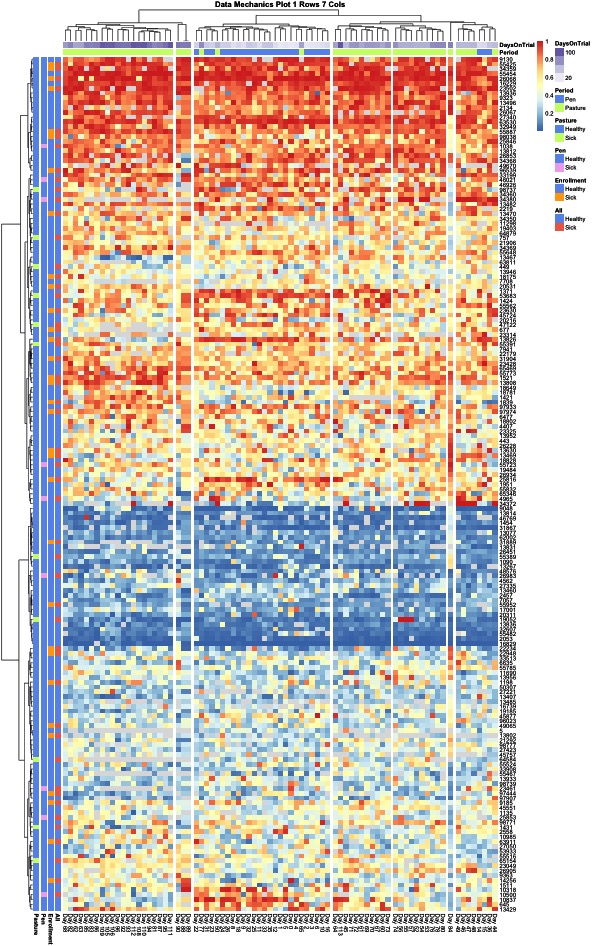

Supplement: Supplementary file 5 [file Data_Sheet_5.ZIP › Grid_LR/DatMechPlotOutR1C7 _Final.jpeg]

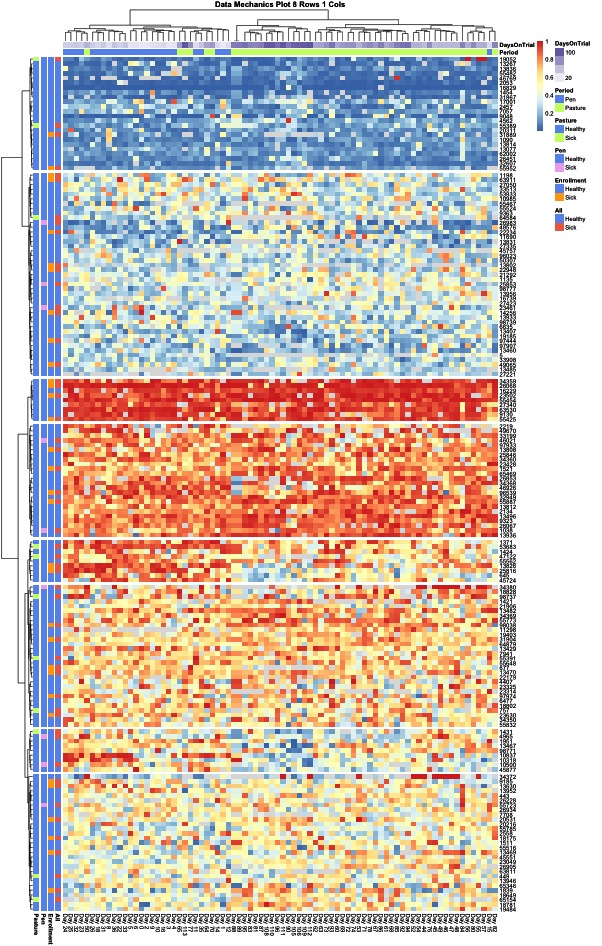

Supplement: Supplementary file 5 [file Data_Sheet_5.ZIP › Grid_LR/DatMechPlotOutR8C1 _Final.jpeg]

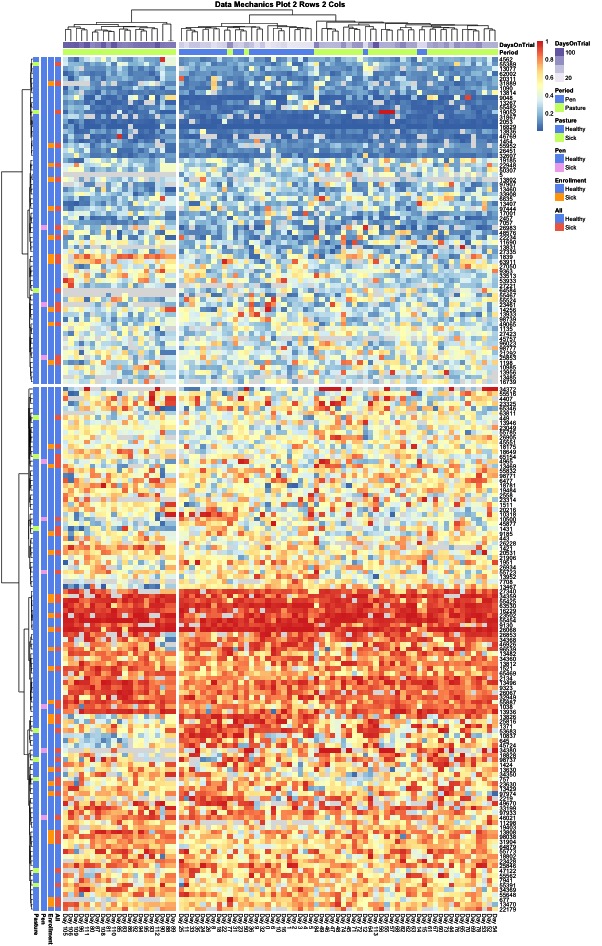

Supplement: Supplementary file 5 [file Data_Sheet_5.ZIP › Grid_LR/DatMechPlotOutR2C2 _Final.jpeg]

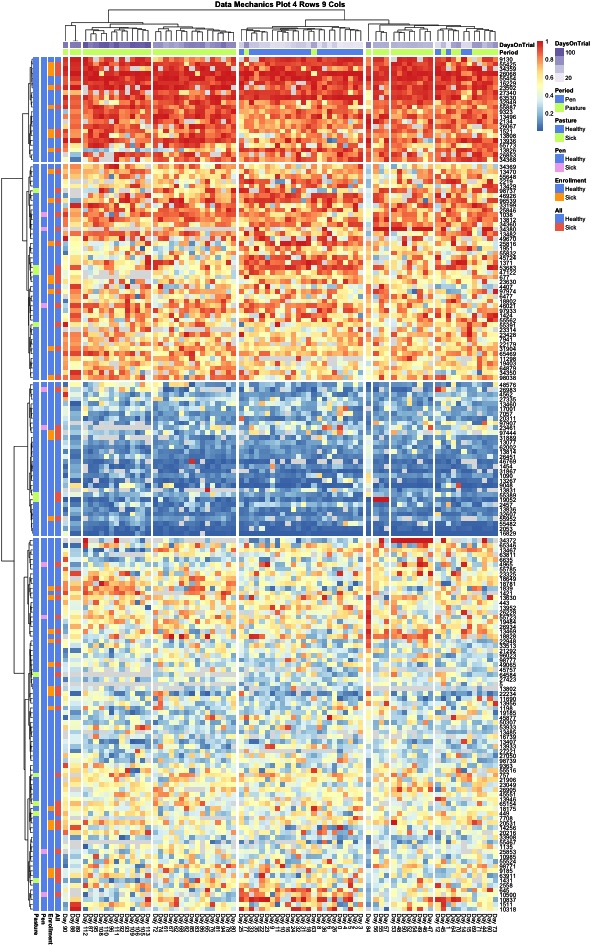

Supplement: Supplementary file 5 [file Data_Sheet_5.ZIP › Grid_LR/DatMechPlotOutR4C9 _Final.jpeg]

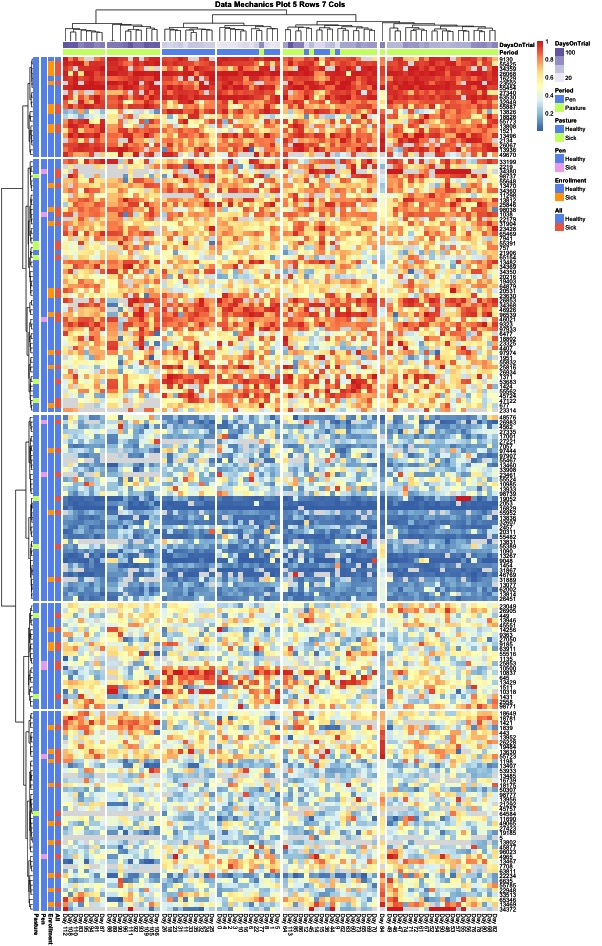

Supplement: Supplementary file 5 [file Data_Sheet_5.ZIP › Grid_LR/DatMechPlotOutR5C7 _Final.jpeg]

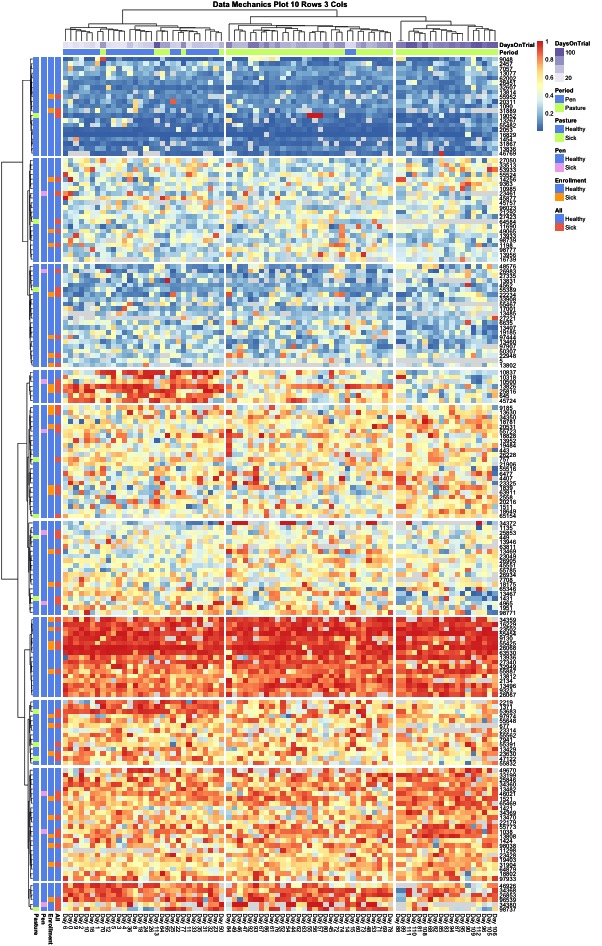

Supplement: Supplementary file 5 [file Data_Sheet_5.ZIP › Grid_LR/DatMechPlotOutR10C3 _Final.jpeg]

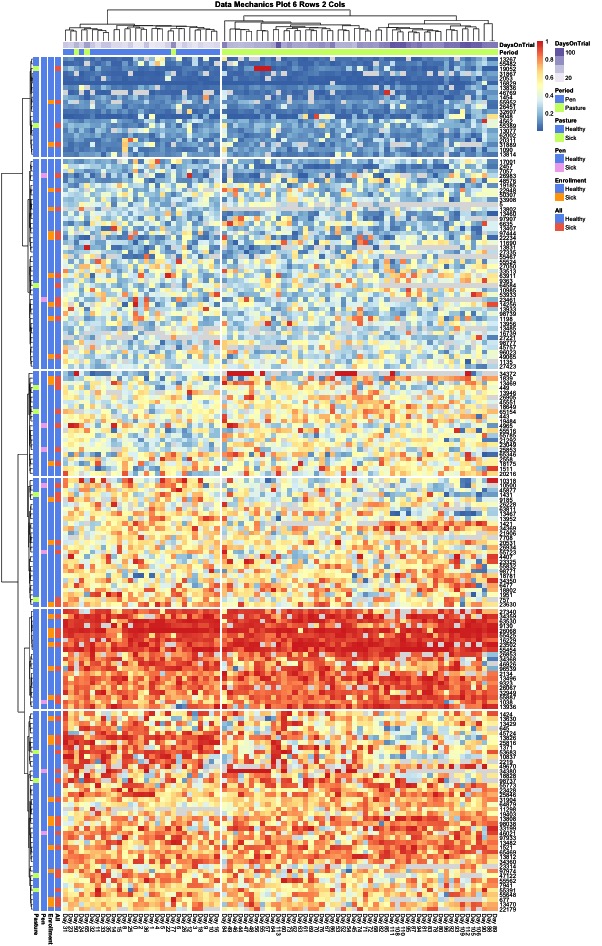

Supplement: Supplementary file 5 [file Data_Sheet_5.ZIP › Grid_LR/DatMechPlotOutR6C2 _Final.jpeg]

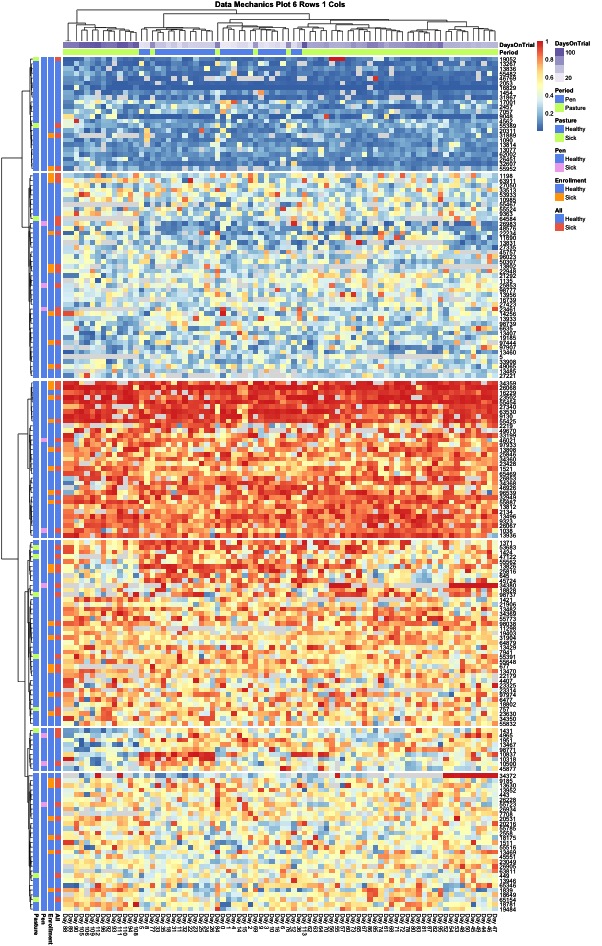

Supplement: Supplementary file 5 [file Data_Sheet_5.ZIP › Grid_LR/DatMechPlotOutR6C1 _Final.jpeg]

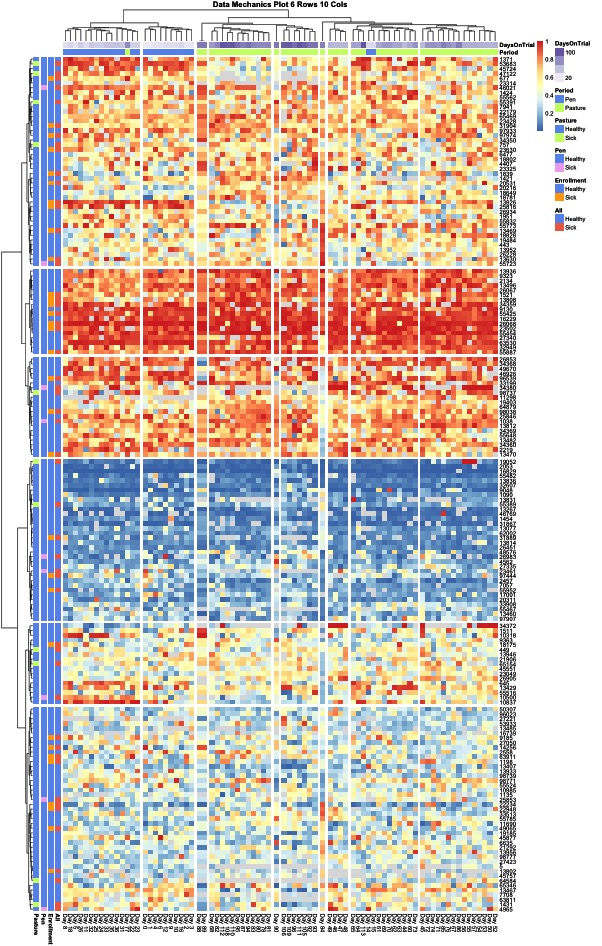

Supplement: Supplementary file 5 [file Data_Sheet_5.ZIP › Grid_LR/DatMechPlotOutR6C10 _Final.jpeg]

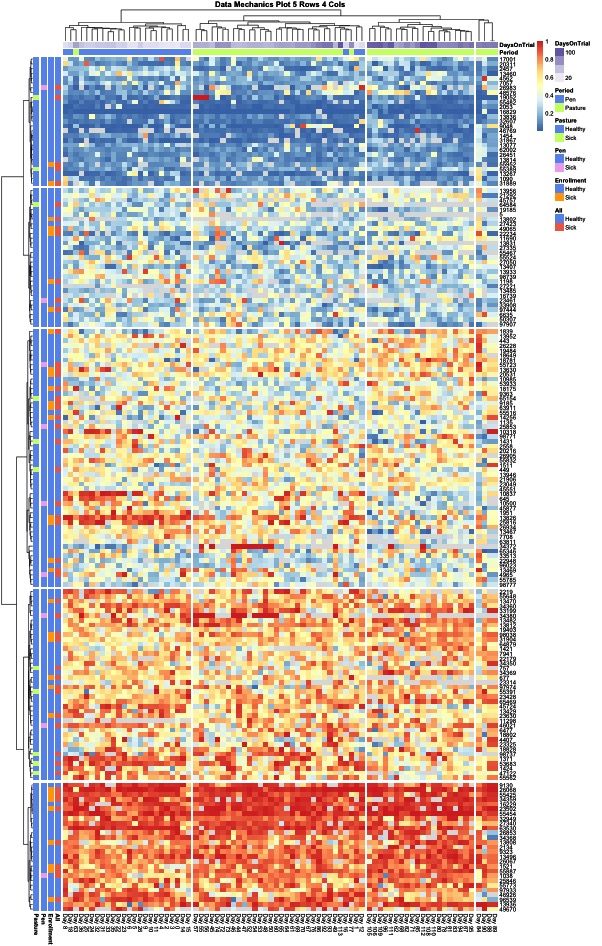

Supplement: Supplementary file 5 [file Data_Sheet_5.ZIP › Grid_LR/DatMechPlotOutR5C4 _Final.jpeg]

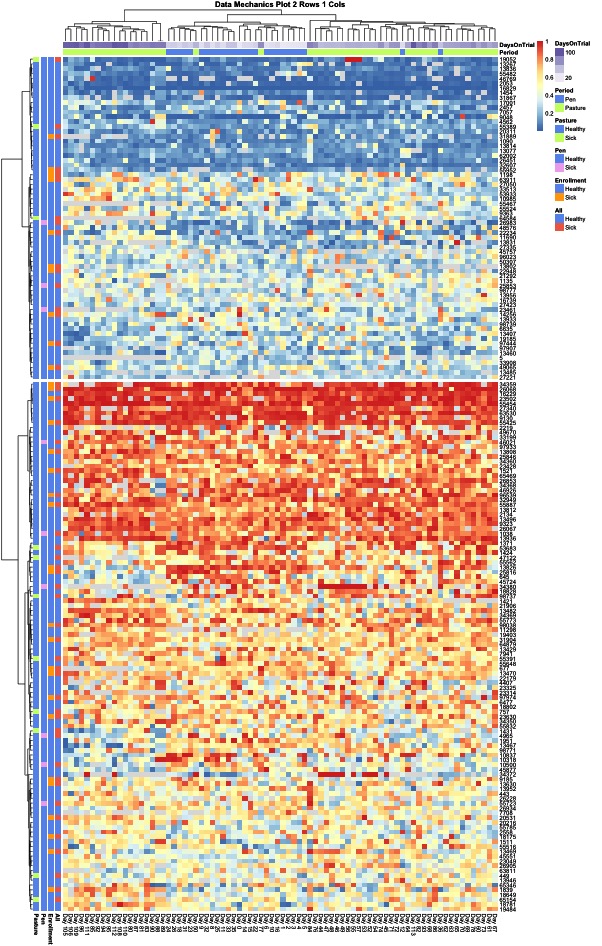

Supplement: Supplementary file 5 [file Data_Sheet_5.ZIP › Grid_LR/DatMechPlotOutR2C1 _Final.jpeg]

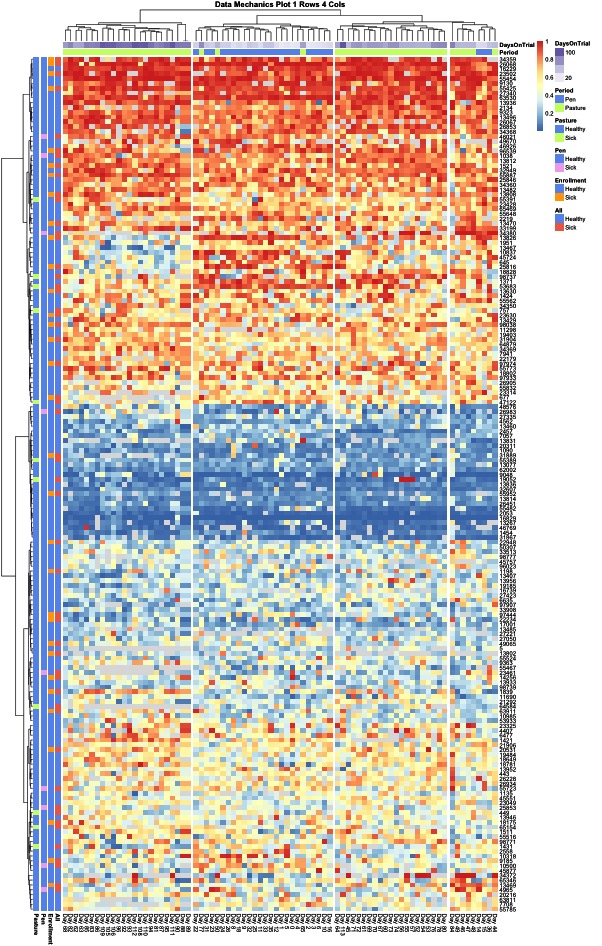

Supplement: Supplementary file 5 [file Data_Sheet_5.ZIP › Grid_LR/DatMechPlotOutR1C4 _Final.jpeg]

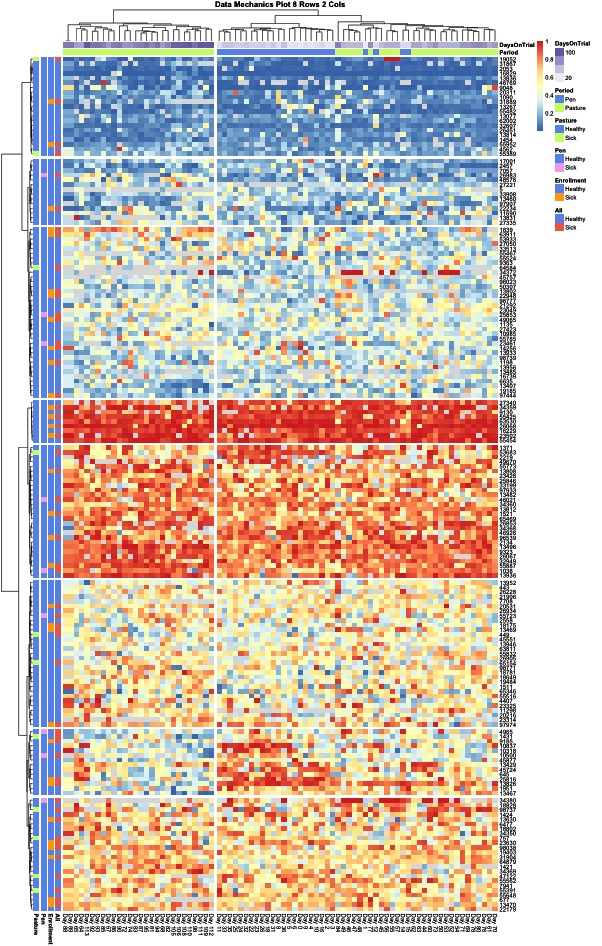

Supplement: Supplementary file 5 [file Data_Sheet_5.ZIP › Grid_LR/DatMechPlotOutR8C2 _Final.jpeg]

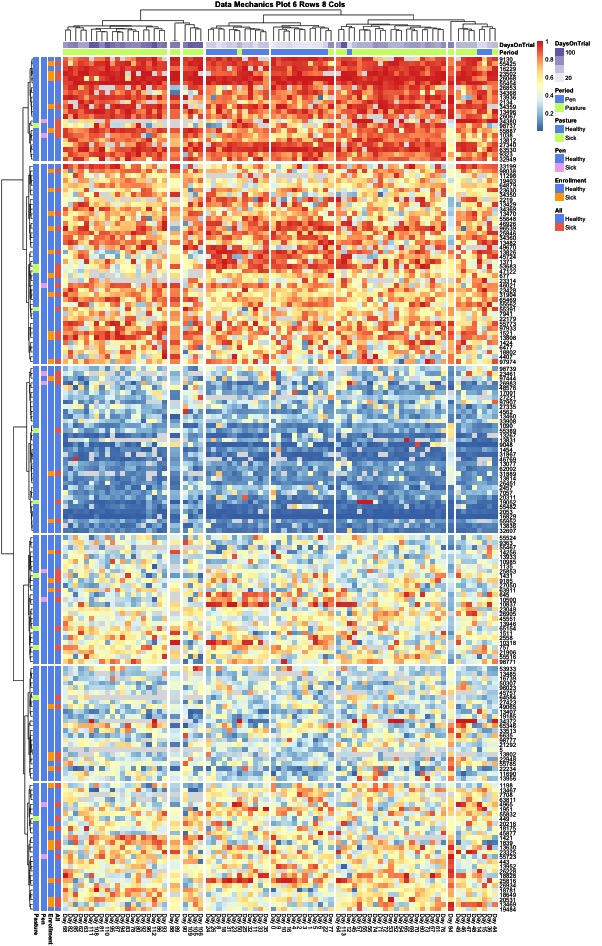

Supplement: Supplementary file 5 [file Data_Sheet_5.ZIP › Grid_LR/DatMechPlotOutR6C8 _Final.jpeg]

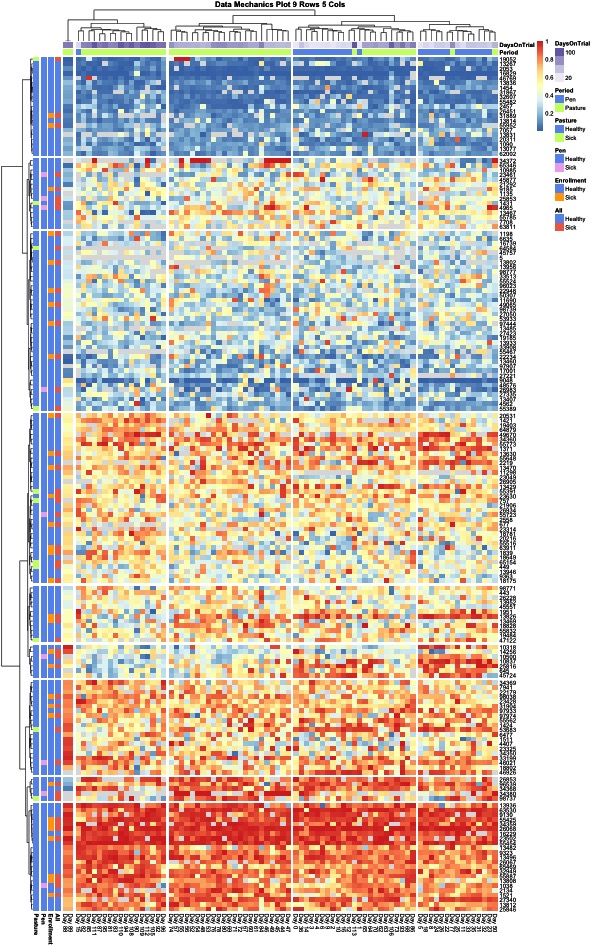

Supplement: Supplementary file 5 [file Data_Sheet_5.ZIP › Grid_LR/DatMechPlotOutR9C5 _Final.jpeg]

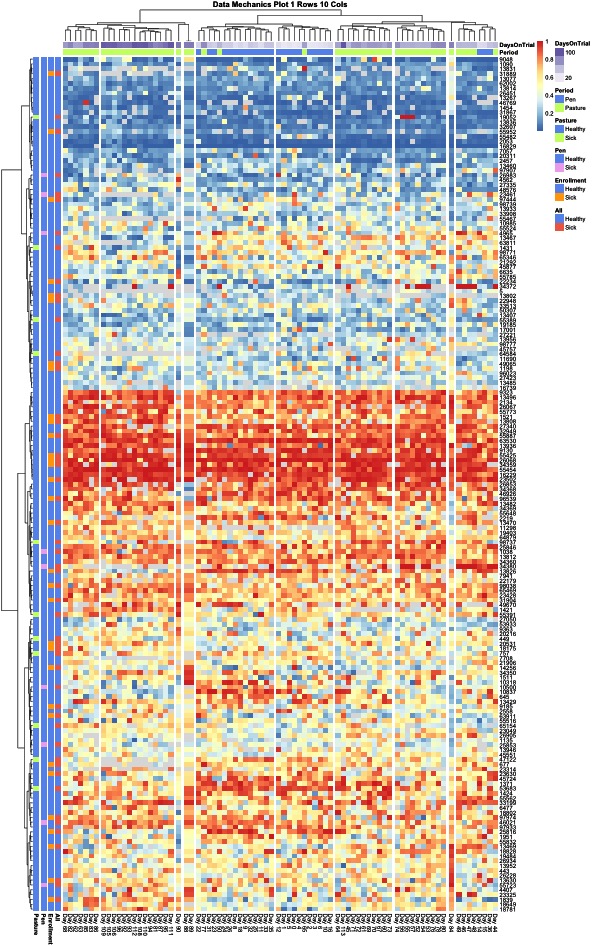

Supplement: Supplementary file 5 [file Data_Sheet_5.ZIP › Grid_LR/DatMechPlotOutR1C10 _Final.jpeg]

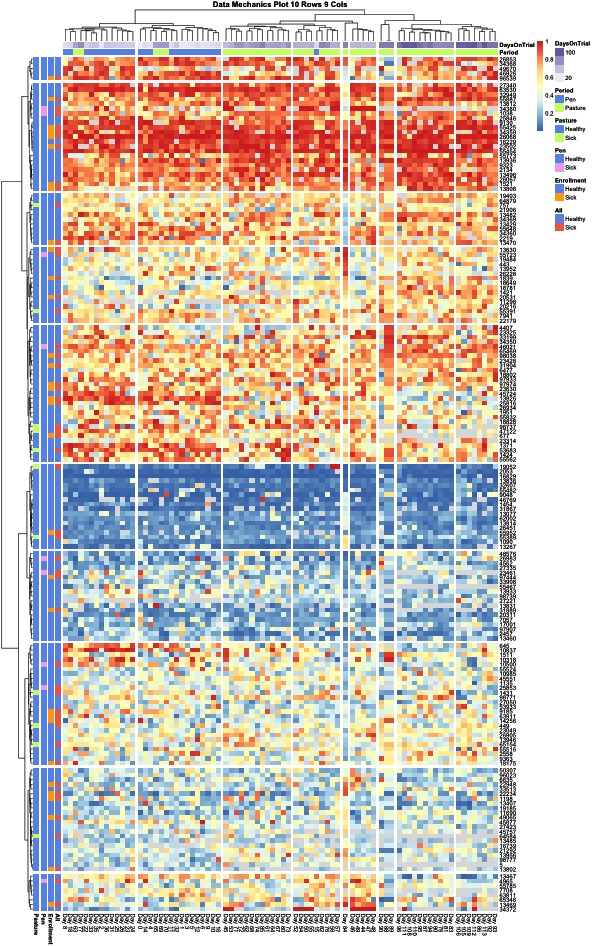

Supplement: Supplementary file 5 [file Data_Sheet_5.ZIP › Grid_LR/DatMechPlotOutR10C9 _Final.jpeg]

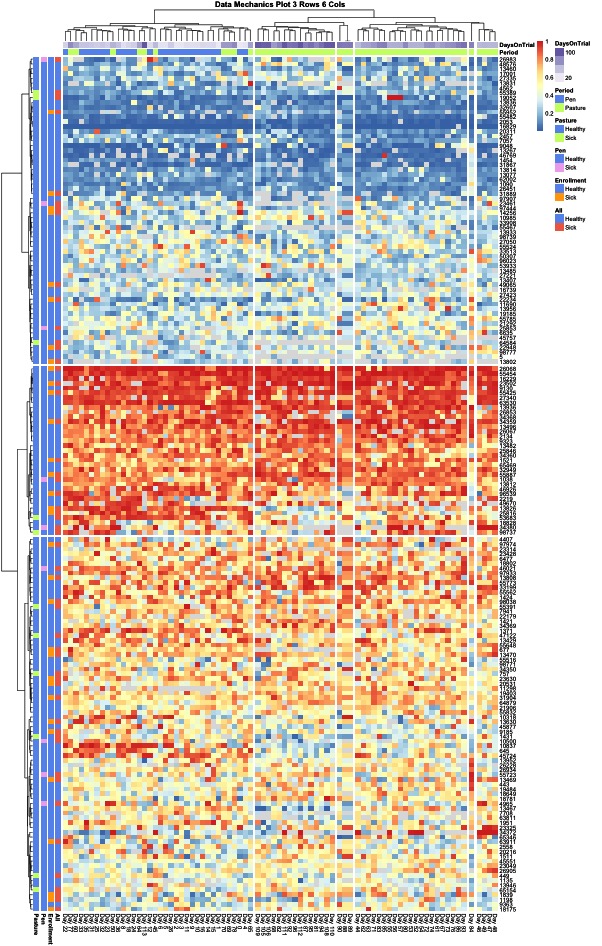

Supplement: Supplementary file 5 [file Data_Sheet_5.ZIP › Grid_LR/DatMechPlotOutR3C6 _Final.jpeg]

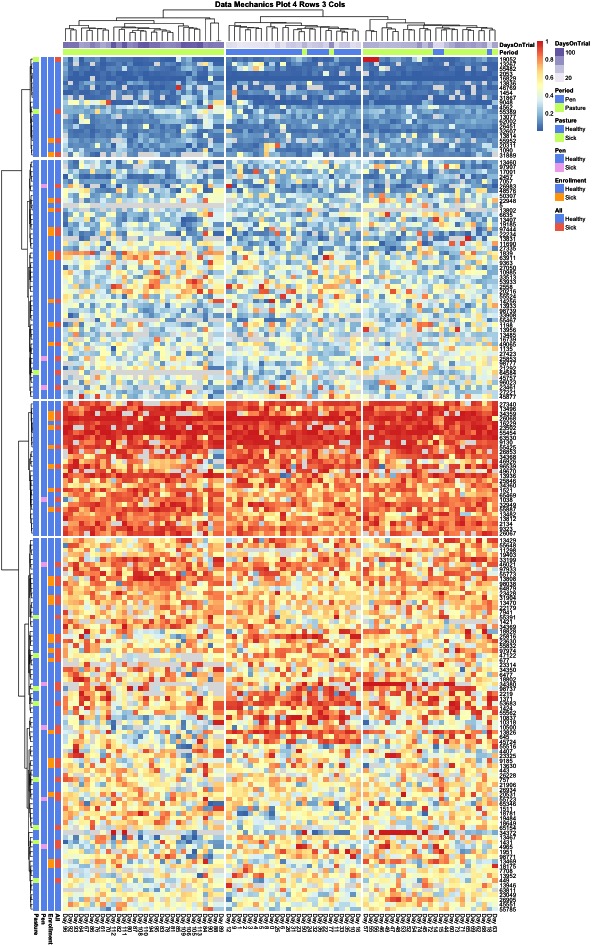

Supplement: Supplementary file 5 [file Data_Sheet_5.ZIP › Grid_LR/DatMechPlotOutR4C3 _Final.jpeg]

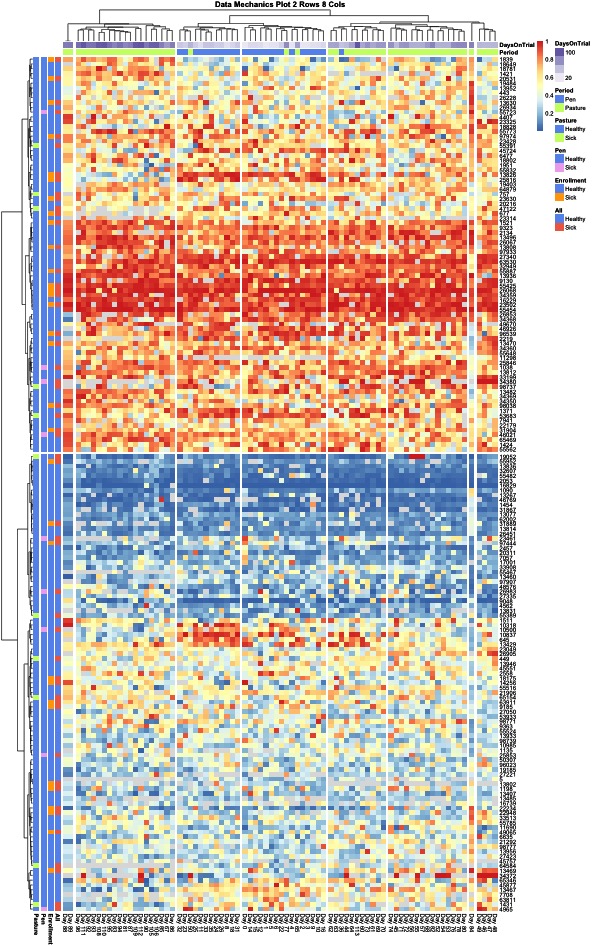

Supplement: Supplementary file 5 [file Data_Sheet_5.ZIP › Grid_LR/DatMechPlotOutR2C8 _Final.jpeg]

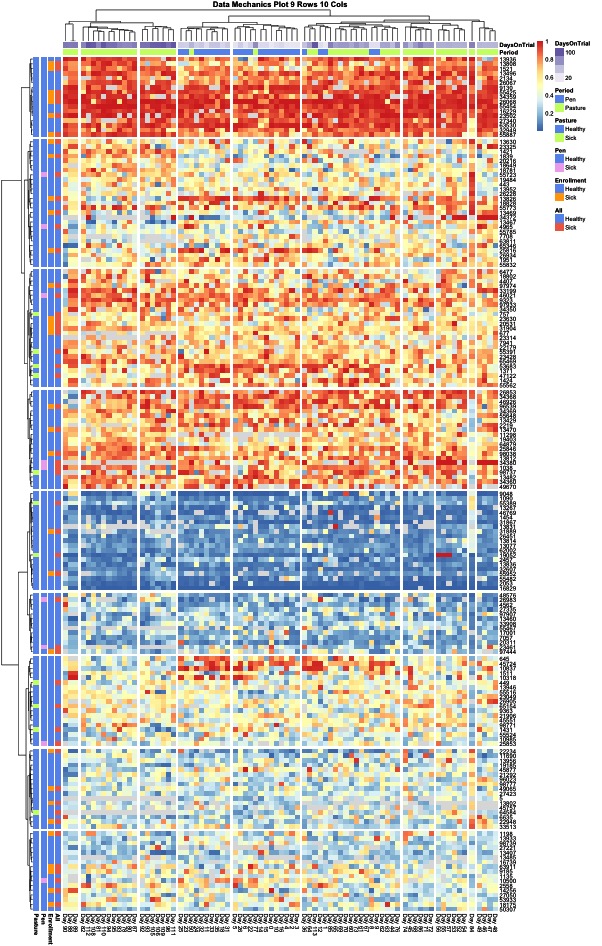

Supplement: Supplementary file 5 [file Data_Sheet_5.ZIP › Grid_LR/DatMechPlotOutR9C10 _Final.jpeg]

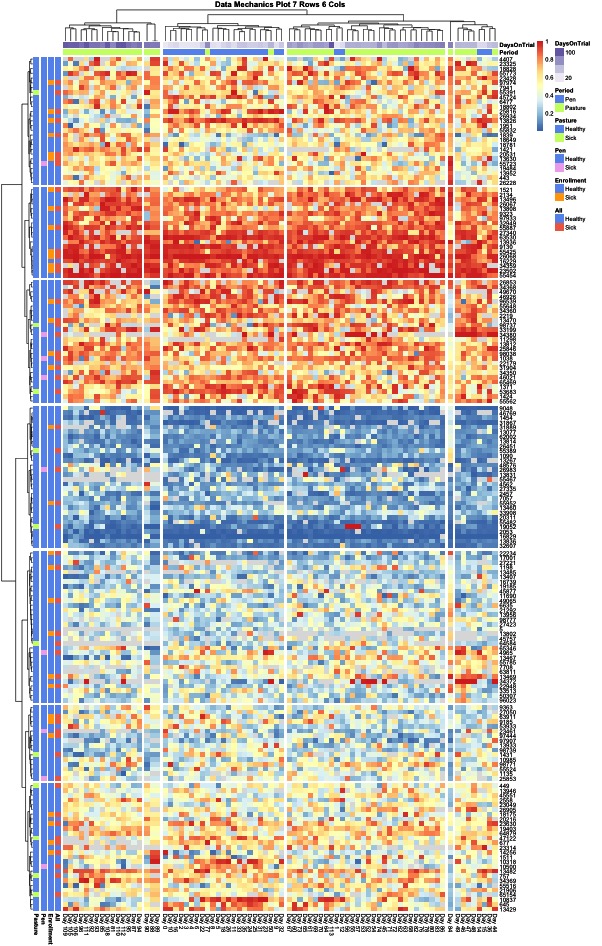

Supplement: Supplementary file 5 [file Data_Sheet_5.ZIP › Grid_LR/DatMechPlotOutR7C6 _Final.jpeg]

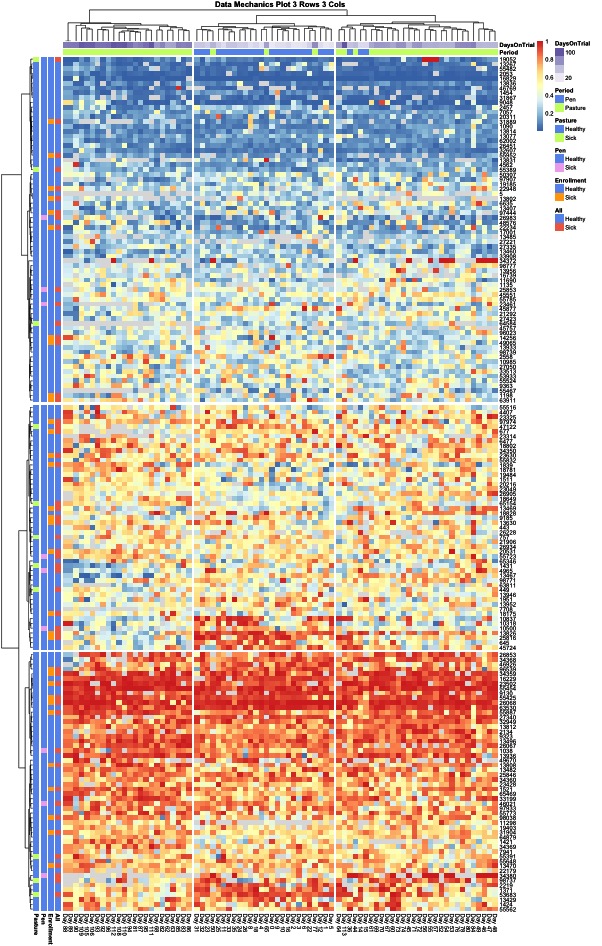

Supplement: Supplementary file 5 [file Data_Sheet_5.ZIP › Grid_LR/DatMechPlotOutR3C3 _Final.jpeg]

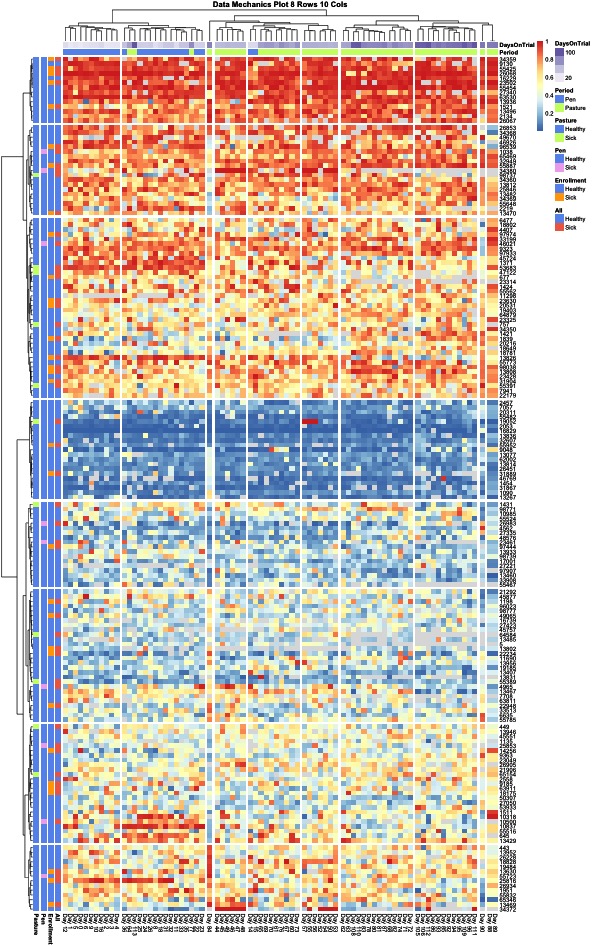

Supplement: Supplementary file 5 [file Data_Sheet_5.ZIP › Grid_LR/DatMechPlotOutR8C10 _Final.jpeg]
