# Supplementary figures and images for: Mind the Queue: A Case Study in Visualizing Heterogeneous Behavioral Patterns in Livestock Sensor Data Using Unsupervised Machine Learning Techniques (part 4 of 4)
Source: Front Vet Sci. 2020 Aug 13;7:523. doi: 10.3389/fvets.2020.00523 (PMC7518149; doi:10.3389/fvets.2020.00523)

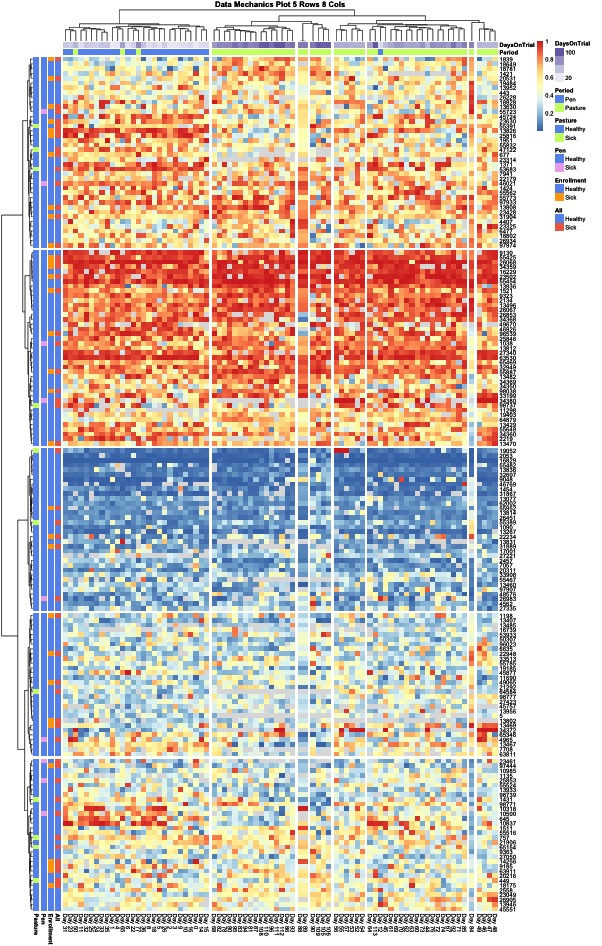

Supplement: Supplementary file 5 [file Data_Sheet_5.ZIP › Grid_LR/DatMechPlotOutR5C8 _Final.jpeg]

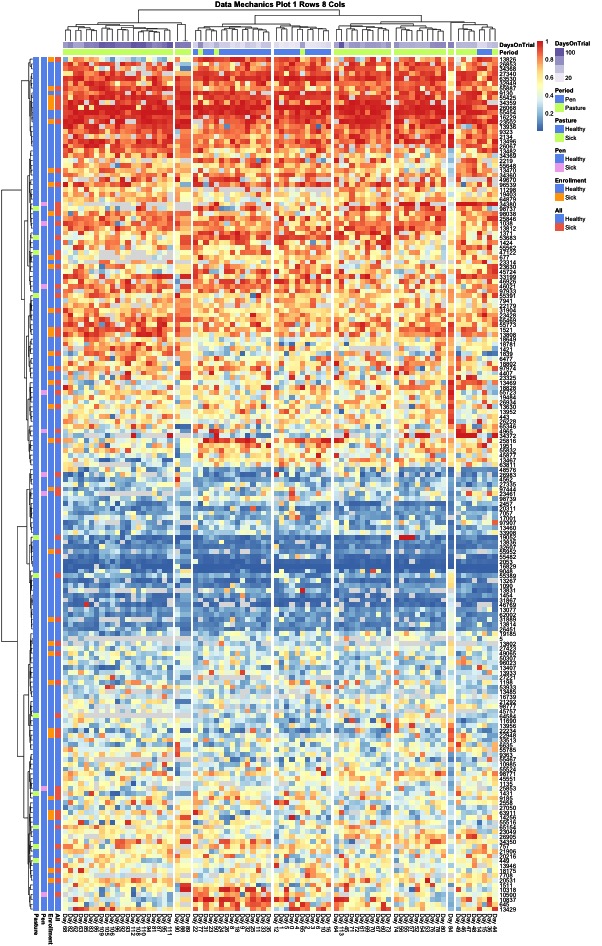

Supplement: Supplementary file 5 [file Data_Sheet_5.ZIP › Grid_LR/DatMechPlotOutR1C8 _Final.jpeg]

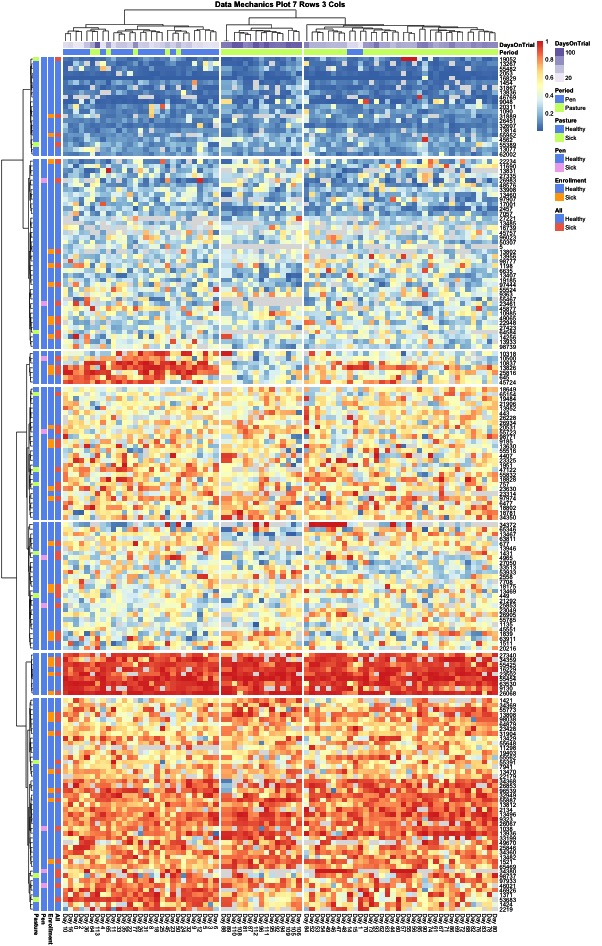

Supplement: Supplementary file 5 [file Data_Sheet_5.ZIP › Grid_LR/DatMechPlotOutR7C3 _Final.jpeg]

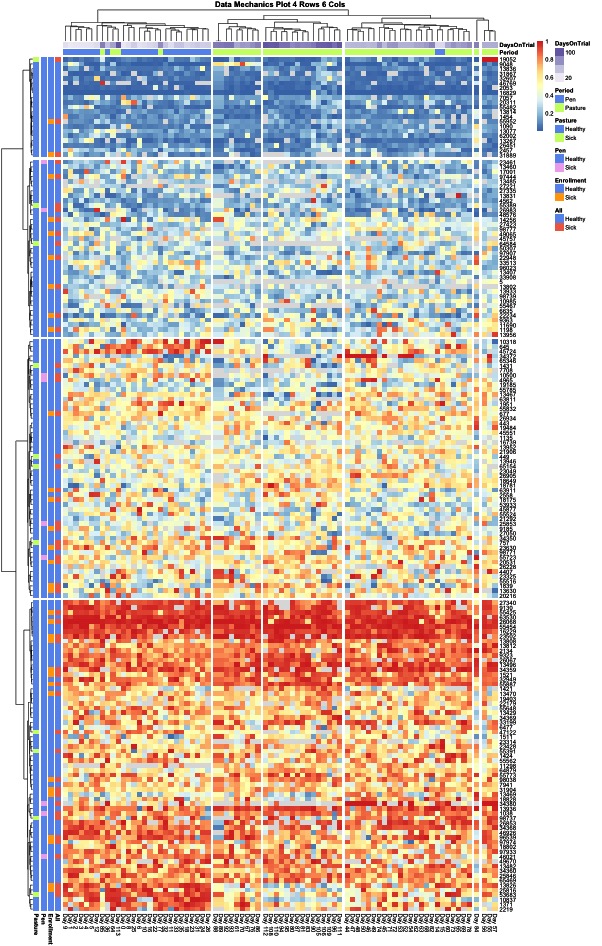

Supplement: Supplementary file 5 [file Data_Sheet_5.ZIP › Grid_LR/DatMechPlotOutR4C6 _Final.jpeg]

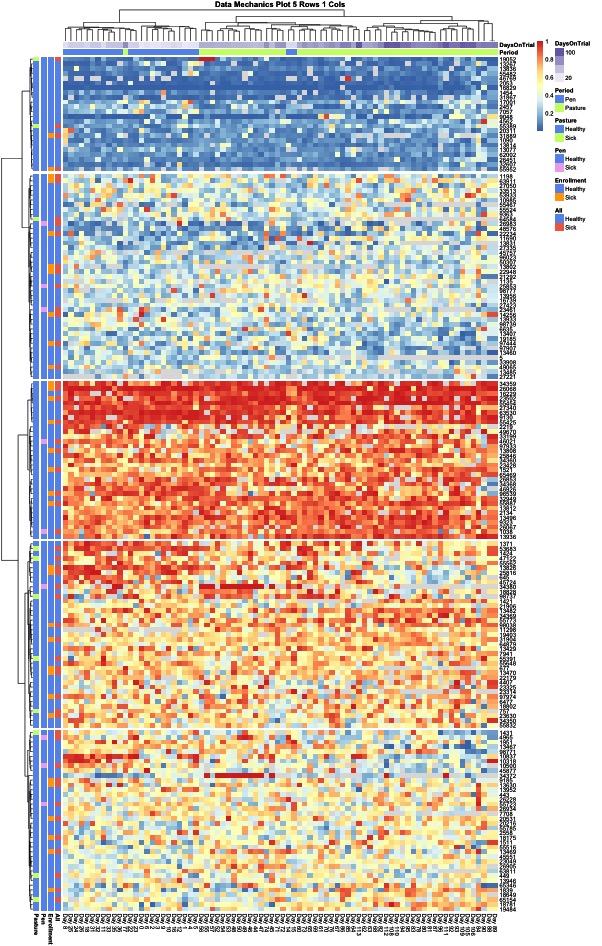

Supplement: Supplementary file 5 [file Data_Sheet_5.ZIP › Grid_LR/DatMechPlotOutR5C1 _Final.jpeg]

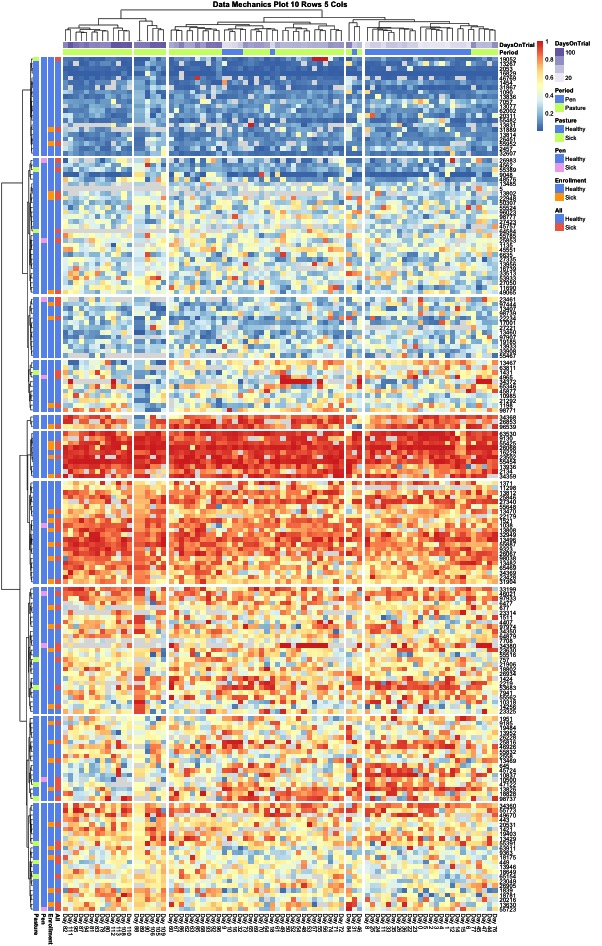

Supplement: Supplementary file 5 [file Data_Sheet_5.ZIP › Grid_LR/DatMechPlotOutR10C5 _Final.jpeg]

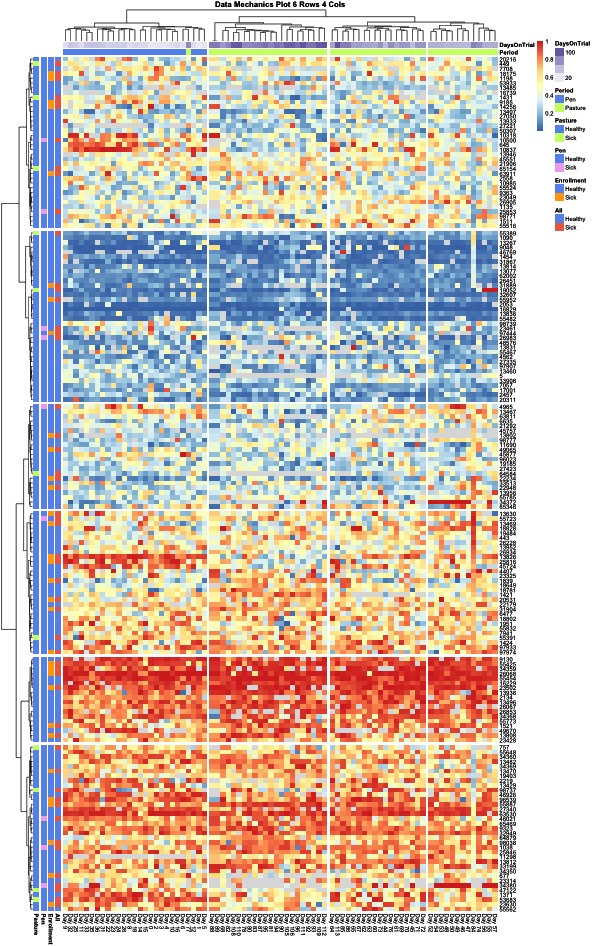

Supplement: Supplementary file 5 [file Data_Sheet_5.ZIP › Grid_LR/DatMechPlotOutR6C4 _Final.jpeg]

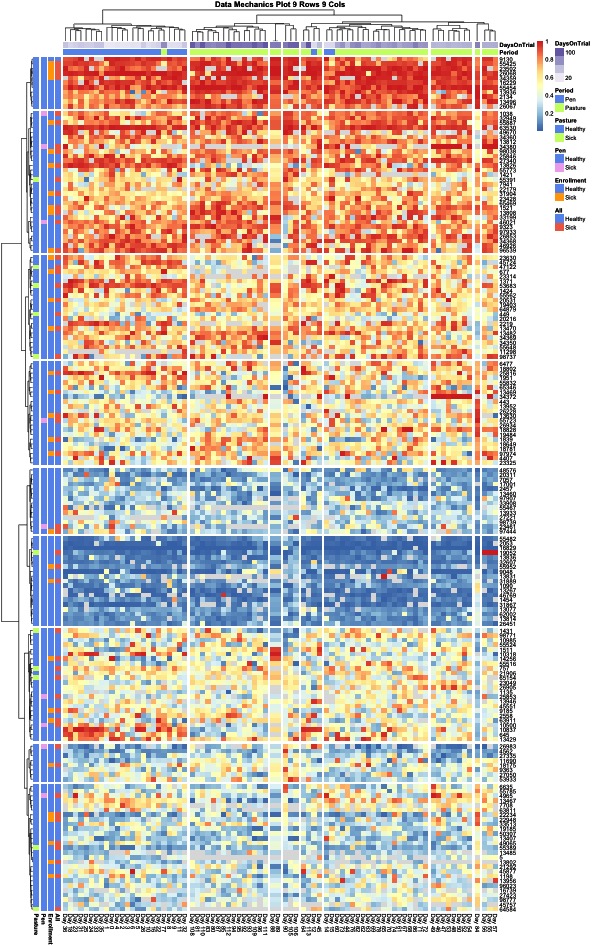

Supplement: Supplementary file 5 [file Data_Sheet_5.ZIP › Grid_LR/DatMechPlotOutR9C9 _Final.jpeg]

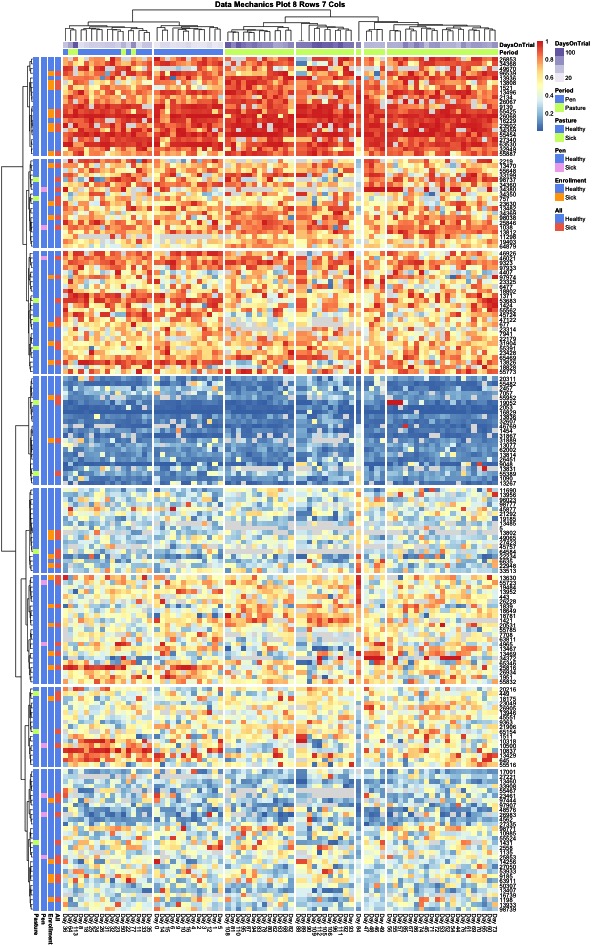

Supplement: Supplementary file 5 [file Data_Sheet_5.ZIP › Grid_LR/DatMechPlotOutR8C7 _Final.jpeg]

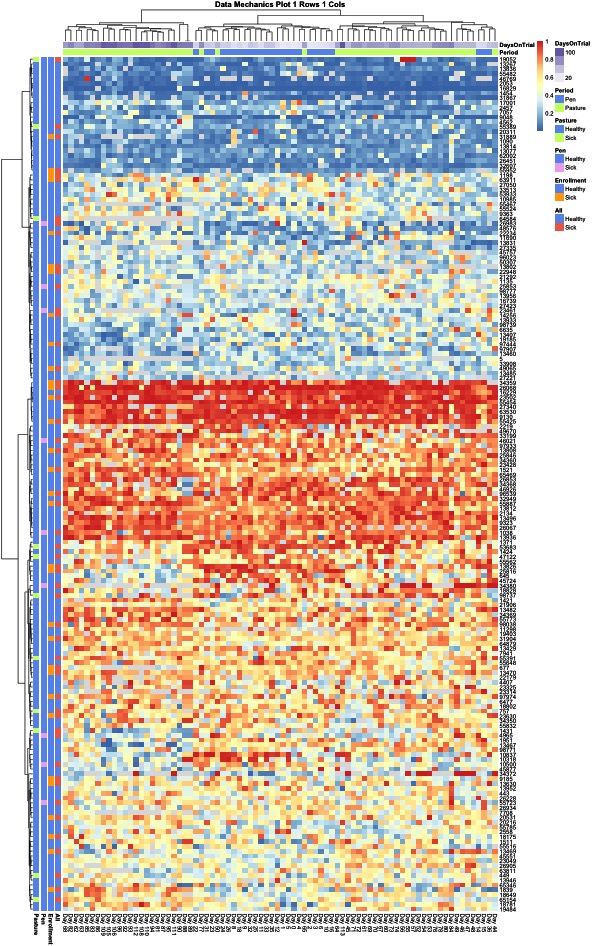

Supplement: Supplementary file 5 [file Data_Sheet_5.ZIP › Grid_LR/DatMechPlotOutR1C1 _Final.jpeg]

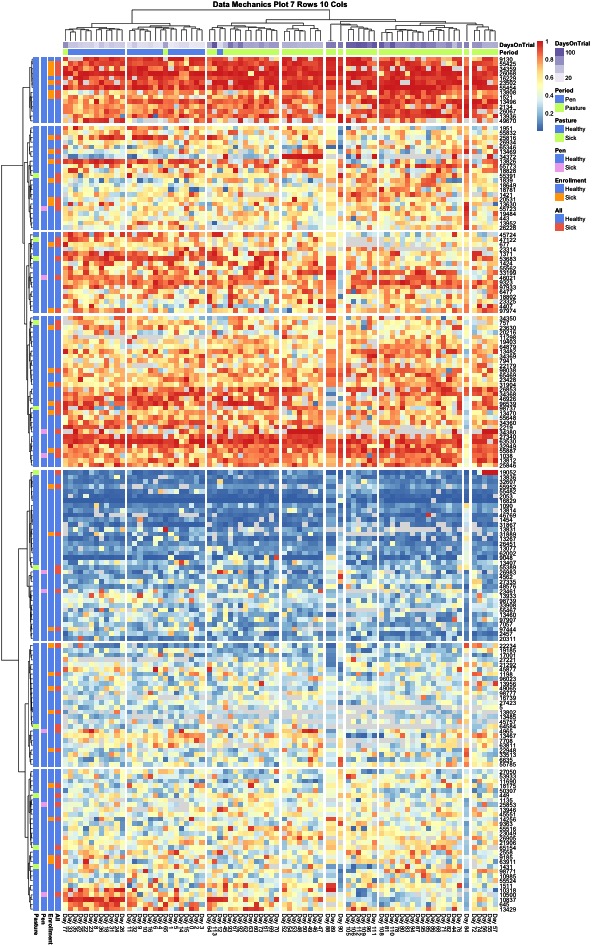

Supplement: Supplementary file 5 [file Data_Sheet_5.ZIP › Grid_LR/DatMechPlotOutR7C10 _Final.jpeg]

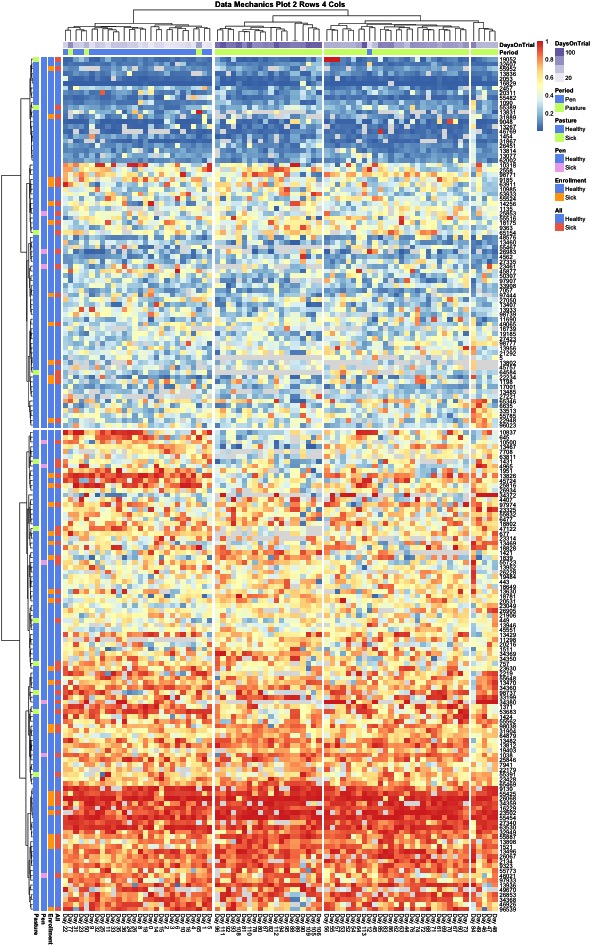

Supplement: Supplementary file 5 [file Data_Sheet_5.ZIP › Grid_LR/DatMechPlotOutR2C4 _Final.jpeg]
