# Supplementary material for: Mind the Queue: A Case Study in Visualizing Heterogeneous Behavioral Patterns in Livestock Sensor Data Using Unsupervised Machine Learning Techniques
Source: Front Vet Sci. 2020 Aug 13;7:523. doi: 10.3389/fvets.2020.00523 (PMC7518149; doi:10.3389/fvets.2020.00523)
Supplement: Supplementary file 6 [file Data_Sheet_6.ZIP › Supplemental_Materials.html]

Supplemental Materials Statistical Analyses


# Supplemental Materials Statistical Analyses

```
isconda = F
isexporting = T

library('plotly')
```

```
## Loading required package: ggplot2
```

```
## 
## Attaching package: 'plotly'
```

```
## The following object is masked from 'package:ggplot2':
## 
##     last_plot
```

```
## The following object is masked from 'package:stats':
## 
##     filter
```

```
## The following object is masked from 'package:graphics':
## 
##     layout
```

```
library('processx')
#library('doBy')
library('plyr')
```

```
## 
## Attaching package: 'plyr'
```

```
## The following objects are masked from 'package:plotly':
## 
##     arrange, mutate, rename, summarise
```

```
library('nlme')
library('emmeans')
```

```
## Warning: package 'emmeans' was built under R version 3.5.2
```

```
library('ggplot2')
library('knitr')
```

```
## Warning: package 'knitr' was built under R version 3.5.2
```

```
library('kableExtra')
library('magrittr')
library('hms')
library('viridis')
```

```
## Loading required package: viridisLite
```

# Data Wrangling - Milk Order Records

## Make Complete Cow List

I want a list of all enrolled cows, which I’ll get from the 150 lactation record data dump.

```
milkyeild150 <- read.csv('Data/Milk Yeild 150 DIM.csv', header = T, stringsAsFactors = F)
cowlist_complete <- unique(milkyeild150$ID)
length(cowlist_complete)
```

```
## [1] 202
```

## Read in CSV’s with identified milking number identified in header

Read in the data:

```
datlist <- read.csv('Data/Milking_Indexed_Data/CSVList.csv', stringsAsFactors = F, header = F)
if(nchar(datlist[1,1])>10){  # I'm only doing this to get around a stupid pc ead in error in first entry
  datlist[1,1] <- '0206_1.csv'
}

orderdata_indexed <- list()

for (i in 1:nrow(datlist)){
  milking <- paste('Data/Milking_Indexed_Data/', datlist[i,1], sep = '')
  temp <- read.csv(milking, stringsAsFactors = F, header = F, na.strings = ' -')
  
  #seperating header and data
  temp2 = temp[1:20,1]
  start <- which("Cow No." == temp2 | "Cow No. " == temp2)
  header <- temp[1:(start-1),]
  dat <- temp[(start+1):nrow(temp), ]
  names(dat) <- temp[start,]
  #dat <-  read.csv(milking, stringsAsFactors = F, header = T, skip = (start-1))
  
  orderdata_indexed[[i]] <-  list(data = dat, HeaderInfo = header)
}
```

Pulling out Sheet Attributes:

```
GroupID <- 34

for (i in 1:length(orderdata_indexed)){
  
  dataf <-  orderdata_indexed[[i]]$data
  headtemp <-  orderdata_indexed[[i]]$HeaderInfo
  

  if(" Milk Time Today 1" %in% names(dataf) | "Milk Time Today 1" %in% names(dataf)){
     
     orderdata_indexed[[i]][['Edited']] <- TRUE
     
     
     # date info
     orderdata_indexed[[i]][['DateC']] <- gsub(' ', '', headtemp[1,7])
     orderdata_indexed[[i]][['Date']] <- as.Date(gsub(' ', '', headtemp[1,7]), format = "%m/%d/%y", tz = 'America/Denver')
     orderdata_indexed[[i]][['DateN']] <- as.numeric(orderdata_indexed[[i]][['Date']])
     
    
     # milking info
     
     orderdata_indexed[[i]][['Milking']] <- 1
     
     # time info
     
     coltemp <- which(names(dataf) ==" Milk Time Today 1" | names(dataf) =="Milk Time Today 1" )
     dataf$MilkingTime <- as.POSIXct(paste(orderdata_indexed[[i]][['DateC']], dataf[,coltemp], sep = ''), format = '%m/%d/%y %H:%M:%S', tz = 'MST')
     
      
     # check if majority of times fall before 9am (and thus during the morning milking)

     temp <- dataf$MilkingTime[dataf[,1] %in% cowlist_complete]
     
     if(sum(as.hms(temp) > as.hms('9:00:00'), na.rm = T)/length(temp) > 1/3){
       orderdata_indexed[[i]][['Edited']] <- FALSE
       next # if more than 1/3 of cows were milk after 9am, then skip this data set b/c it isn't normal morning milking
     }
     
     dataf$MilkingTime[which(as.hms(dataf$MilkingTime) > as.hms('9:00:00'))]<- NA # any cows milked after 9am set as a missing milking record
     dataf$OverallMilkingOrder <- rank(dataf$MilkingTime, na.last = 'keep')
     orderdata_indexed[[i]][['data_formatted']] <- dataf
     
     # order info
     
     datasub <- subset(dataf, dataf$` Group No.` == '34')
     datasub$MilkingOrder <- rank(datasub$MilkingTime, na.last = 'keep')
     datasub <- datasub[order(datasub$MilkingTime), ]
     
     # yeild info
     datasub$MilkingYield <- as.numeric(datasub$` Milk Today 1`)
     datasub$MilkingYieldRank <- rank(datasub$MilkingYield, na.last = 'keep') # yeild in morning milk relative the group
     
     datasub$TotYield <- NA
     datasub$TotYieldRank <- NA
     
     # milking duration & DIM
     
      coltemp <- which(names(dataf) ==" Milk Dur. Today 1" | names(dataf) =="Milk Dur. Today 1" )
      datasub$MilkingDurationC <- paste('0:',gsub(' ', '', datasub[,coltemp]), sep = '' )
      datasub$MilkingDuration <- as.hms(datasub$MilkingDurationC) - as.hms('0:00:00') # milking time in seconds
     
      coltemp <- which(names(dataf) ==" Days In Milk" | names(dataf) =="Days In Milk" )
      datasub$DIM <- as.integer(datasub[,coltemp])

     
     orderdata_indexed[[i]][['data_subset']] <- datasub
     orderdata_indexed[[i]][['CowCount']] <- sum(!is.na(datasub$MilkingTime) & datasub[,1] %in% cowlist_complete, na.rm=T)

    
  }else if(" Milk Time Yest 1" %in% names(dataf) | "Milk Time Yest 1" %in% names(dataf) ){
    
     orderdata_indexed[[i]][['Edited']] <- TRUE
     
     # date info
     
     orderdata_indexed[[i]][['DateC']] <- gsub(' ', '', headtemp[1,7])
     orderdata_indexed[[i]][['Date']] <- as.Date(gsub(' ', '', headtemp[1,7]), format = "%m/%d/%y", tz = 'America/Denver') - 1
     orderdata_indexed[[i]][['DateN']] <- as.numeric(orderdata_indexed[[i]][['Date']])
     
     
     # milking info
     
     orderdata_indexed[[i]][['Milking']] <- 1
     
     # time info
     
     coltemp <- which(names(dataf) ==" Milk Time Yest 1" | names(dataf) =="Milk Yest Today 1" )
     dataf$MilkingTime <- NA
     dataf$MilkingTime <- as.POSIXct(dataf$MilkingTime)
     
     dataf$MilkingTime <- as.POSIXct(paste(as.character(orderdata_indexed[[i]][['Date']]), dataf[,coltemp], sep = ''), tryFormats = c('%Y-%m-%d %H:%M:%S'), tz = 'MST', optional = T)

     # check if majority of times fall before 9am (and thus during the morning milking)

     temp <- dataf$MilkingTime[dataf[,1] %in% cowlist_complete]
     
     if(sum(as.hms(temp) > as.hms('9:00:00'), na.rm = T)/length(temp) > 1/3){
       orderdata_indexed[[i]][['Edited']] <- FALSE
       next # if more than 1/3 of cows were milk after 9am, then skip this data set b/c it isn't normal morning milking
     }
     
     dataf$MilkingTime[which(as.hms(dataf$MilkingTime) > as.hms('9:00:00'))]<- NA # any cows milked after 9am set as a missing milking record
     dataf$OverallMilkingOrder <- rank(dataf$MilkingTime, na.last = 'keep')
     orderdata_indexed[[i]][['data_formatted']] <- dataf
     
     # order info
     
     datasub <- subset(dataf, dataf$` Group No.` == '34')
     datasub$MilkingOrder <- rank(datasub$MilkingTime, na.last = 'keep')
     datasub <- datasub[order(datasub$MilkingTime), ]
     
     # yeild info
     datasub$MilkingYield <- as.numeric( datasub$` Milk  Yest 1`)
     datasub$MilkingYieldRank <- rank(datasub$MilkingYield, na.last = 'keep') # yeild in morning milk relative the group
     
     datasub$TotYield <- as.numeric( datasub$` Tot. Milk Yest.`)
     datasub$TotYieldRank <- rank(datasub$TotYield, na.last = 'keep') # total yeild relative the group
     
     # milking duration & DIM
     
      coltemp <- which(names(dataf) ==" Milk Dur. Yest 1" | names(dataf) =="Milk Dur. Yest 1" )
      datasub$MilkingDurationC <- paste('0:',gsub(' ', '', datasub[,coltemp]), sep = '' )
      datasub$MilkingDuration <- as.hms(datasub$MilkingDurationC) - as.hms('0:00:00') # milking time in seconds
      
    
      coltemp <- which(names(dataf) ==" Days In Milk" | names(dataf) =="Days In Milk" )
      datasub$DIM <- as.integer(datasub[,coltemp])
     
     
     
     orderdata_indexed[[i]][['data_subset']] <- datasub
     orderdata_indexed[[i]][['CowCount']] <- sum(!is.na(datasub$MilkingTime) & datasub[,1] %in% cowlist_complete, na.rm=T)
     
     
     
     
    
  }else{
    
    orderdata_indexed[[i]][['Edited']] <- FALSE
    
  }
  
  
}
```

Check on duplicate entries

```
dates_indexed <- data.frame(Date = NA, DateN = NA)

for(i in 1:length(orderdata_indexed)){
  
  if(orderdata_indexed[[i]]$Edited){
    dates_indexed[i,1] <- as.character(orderdata_indexed[[i]][['Date']])
    dates_indexed[i,2] <- orderdata_indexed[[i]][['DateN']]
  }
  
}

# View(dates_indexed[order(dates_indexed$DateN),])
# 
# temp <- data.frame(Day1 = orderdata_indexed[[60]]$data_subset$MilkingTime, Day2 = orderdata_indexed[[63]]$data_subset$MilkingTime)
# View(temp)

# Drop Duplicate Days

orderdata_indexed[[4]]$Edited <- FALSE
```

## Read in CSV’s with unidentified milking number

Read in the data:

```
datlist <- read.csv('Data/Milking_Infered_Data/CSVList.csv', stringsAsFactors = F, header = F)
orderdata_infered <- list()

for (i in 1:nrow(datlist)){
  
  milking <- paste('Data/Milking_Infered_Data/', datlist[i,1], sep = '')
  temp <- read.csv(milking, stringsAsFactors = F, header = F, na.strings = ' -')
  
  #seperating header 
  temp2 = temp[1:20,1]
  start1 <- which("Cow No." == temp2 | "Cow No. " == temp2)
  #start <- which(grepl('Count', temp[,1]))
  header <- temp[1:(start1-1),]
  
  # read in just the data (and avoid the column formatting issues)
  
  temp <- read.csv(milking, stringsAsFactors = F, na.strings = ' -', header = F, skip = start1-1)
  temp2 = temp[1:20,1]
  start2 <- which("Cow No." == temp2 | "Cow No. " == temp2)

  dat <- temp[(start2+1):nrow(temp),]
  names(dat) <- temp[start2,]
  
  orderdata_infered[[i]] <-  list(data = dat, HeaderInfo = header)
}

# check that data was read in correctly 

# for(i in 1:length(orderdata_infered) ){
#   
#   print(names(orderdata_infered[[i]]$data)[1])
#   
# }
```

Pulling out Sheet Attributes:

A number of these sheets don’t have entries for milking time. I think they are in fact sorted by milking order, but since I can’t validate this, I’m not going to use these records.

```
for (i in 1:length(orderdata_infered)){
 
   dataf <- orderdata_infered[[i]]$data
   
   if('Milk.Time.Yest.1' %in% names(dataf) | " Milk Time Yest 1" %in% names(dataf)){
     
     
     # get date
     
     headtemp <- orderdata_infered[[i]]$HeaderInfo
     timetemp <- gsub(' ', '', headtemp[1,3])
     datestart <- regexpr('Date:', timetemp)[1] + 5
     datestop <- regexpr('Time', timetemp)[1] - 1
     
     orderdata_infered[[i]][['Date']] <- as.Date(substr(timetemp, datestart, datestop), tryFormats = c("%m/%d/%y", "%d.%m.%y"), tz = 'America/Denver') - 1
     orderdata_infered[[i]][['DateC']] <- as.character(orderdata_infered[[i]][['Date']])
     orderdata_infered[[i]][['DateN']] <- as.numeric(orderdata_infered[[i]][['Date']])
     
     # format time data
     
     coltemp <- which(names(dataf) =="Milk.Time.Yest.1" | names(dataf) ==" Milk Time Yest 1" )
     
     dataf$MilkingTime <- as.POSIXct(paste(orderdata_infered[[i]][['DateC']], dataf[,coltemp], sep = ' '), tryFormats = c('%Y-%m-%d  %H:%M:%S'), tz = 'MST', optional = T)
     dataf$MilkingTime[!is.na(dataf[,coltemp])] <- as.POSIXct( paste( orderdata_infered[[i]][['DateC']], dataf[!is.na(dataf[,coltemp]),coltemp], sep = ' '), tryFormats = c('%Y-%m-%d  %H:%M:%S'), tz = 'MST', optional = T)
     
     
     # check if majority of times fall before 9am (and thus during the morning milking)

     temp <- dataf$MilkingTime[dataf[,1] %in% cowlist_complete]
     
     if(sum(as.hms(temp) > as.hms('9:00:00'), na.rm = T)/length(temp) > 1/3){
       orderdata_infered[[i]][['Edited']] <- FALSE
       next # if more than 1/3 of cows were milk after 9am, then skip this data set b/c it isn't normal morning milking
     }
     
     dataf$MilkingTime[which(as.hms(dataf$MilkingTime) > as.hms('9:00:00'))]<- NA # any cows milked after 9am set as a missing milking record
     
     
     orderdata_infered[[i]][['dataf']] <- dataf
     
     datasub <- dataf[order(dataf$MilkingTime),]
     datasub$MilkingOrder <- rank(datasub$MilkingTime, na.last = 'keep')
     
     # format milk data
     
     coltemp <- which(names(dataf) =="Milk..Yest.1" | names(dataf) ==" Milk  Yest 1" )
     datasub$MilkingYield <- as.numeric(datasub[,coltemp])
     datasub$MilkingYieldRank <- rank(datasub$MilkingYield, na.last = 'keep')
     
     coltemp <- which(names(dataf) =="Tot..Milk.Yest." | names(dataf) ==" Tot. Milk Yest." )
     datasub$TotYield <- as.numeric( datasub[,coltemp])
     datasub$TotYieldRank <- rank(datasub$TotYield, na.last = 'keep')
     
      # milking duration & DIM
     
      coltemp <- which(names(dataf) =="Days.In.Milk" | names(dataf) ==" Days In Milk" )
      datasub$DIM <- as.integer(datasub[,coltemp])
     
     
       # Low Yield Flag
      
      lyftemp <- which(names(dataf) == 'Low Yield Yest. 3' | names(dataf) == ' Low Yield Yest. 3' )
      if(length(lyftemp)>0){
        datasub$LowYield3 <- datasub[,lyftemp]
      }
     
      lyftemp <- which(names(dataf) == 'Low Yield Yest. 2' | names(dataf) == ' Low Yield Yest. 2' )
      if(length(lyftemp)>0){
        datasub$LowYield2 <- datasub[,lyftemp]
      }
     
      lyftemp <- which(names(dataf) == 'Low Yield Yest. 1' | names(dataf) == ' Low Yield Yest. 1' )
      if(length(lyftemp)>0){
        datasub$LowYield1 <- datasub[,lyftemp]
      }
      
     # double check that milking times are in the morning
     
      orderdata_infered[[i]][['data_subset']] <- datasub
      orderdata_infered[[i]][['CowCount']] <- sum(!is.na(datasub$MilkingTime) & datasub[,1] %in% cowlist_complete, na.rm=T)
     
      orderdata_infered[[i]][['Edited']] <- TRUE
     
   }else{
     orderdata_infered[[i]][['Edited']] <- FALSE
   }
   
  
}
```

Ok, now I need to go in and check a couple duplicate days, which look like duplicated dumps

```
dates_infered <- data.frame(Date = NA, DateN = NA)

for(i in 1:length(orderdata_infered)){
  
  if(orderdata_infered[[i]]$Edited){
    dates_infered[i,1] <- as.character(orderdata_infered[[i]][['Date']])
    dates_infered[i,2] <- orderdata_infered[[i]][['DateN']]
  }
  
}

# View(dates_infered[order(dates_infered$DateN),])
# 
# temp <- data.frame(Day1 = orderdata_infered[[60]]$data_subset$MilkingTime, Day2 = orderdata_infered[[63]]$data_subset$MilkingTime)
# View(temp)

# Drop Duplicate Days

orderdata_infered[[37]]$Edited <- FALSE
orderdata_infered[[45]]$Edited <- FALSE
orderdata_infered[[50]]$Edited <- FALSE
orderdata_infered[[60]]$Edited <- FALSE
orderdata_infered[[61]]$Edited <- FALSE
orderdata_infered[[62]]$Edited <- FALSE
```

## Aggregate Milk Order Outcomes

```
milkorderdat <- data.frame(CowID = cowlist_complete)

for(i in 1:length(orderdata_infered)){
  if(orderdata_infered[[i]][['Edited']]){
    
    dat <- orderdata_infered[[i]][['data_subset']]
    dat2 <- data.frame(CowID = dat[,1])
    dat2$MilkingOrder <- dat$MilkingOrder
    
    milkorderdat <- merge(milkorderdat, dat2, by = 'CowID', all.x = T)
    names(milkorderdat)[ncol(milkorderdat)] <- orderdata_infered[[i]][['DateN']]
    
    if(is.na(orderdata_infered[[i]][['DateN']])){
      print(i)
    }
    
  }
}

#table(names(milkorderdat))

for(i in 1:length(orderdata_indexed)){
  if(orderdata_indexed[[i]][['Edited']]){
    
    dat <- orderdata_indexed[[i]][['data_subset']]
    dat2 <- data.frame(CowID = dat[,1])
    dat2$MilkingOrder <- dat$MilkingOrder
    
    milkorderdat <- merge(milkorderdat, dat2, by = 'CowID', all.x = T)
    names(milkorderdat)[ncol(milkorderdat)] <- orderdata_indexed[[i]][['DateN']]
    
  }
  
  
}

# reorder columns by date

temp <- milkorderdat[,-1]
temp2 <- as.numeric(names(temp))
milkorderdat <- cbind(milkorderdat$CowID, temp[,order(temp2)])
names(milkorderdat)[1] <- 'CowID'
```

And now, I want to normalize these results to account for the fluctuating number of animals in the pen. I’ll do this by dividing the entry position by the total number of cows in a column with non-na values. The first cows in the pen will still have the lowest quantuile values, but now they will consistently range from 0-1.

While I’m at it, I also want to track attendance of cows in each milking observation.

```
milkquantdat <- data.frame(CowID = milkorderdat$CowID)
cowattendance <- c()

for(i in 2:ncol(milkorderdat)){
  
  milkquantdat[,i] <- milkorderdat[,i]/sum(!is.na(milkorderdat[,i]))
  names(milkquantdat)[i] <- names(milkorderdat)[i]
  
  cowattendance <- c(cowattendance, sum(!is.na(milkorderdat[,i])))
  
}

names(cowattendance) <- names(milkorderdat)[-1]
#cowattendance

qplot(cowattendance, geom="histogram", xlab = 'Number of Cows in Attendance', ylab = '', main = 'Distribution of Cow Attendance Rates Across Days', col=I("black"), fill=I("blue"), alpha=I(.5), bins = 20)  + geom_vline(xintercept = 150)
```

```
qplot(as.numeric(names(cowattendance)), cowattendance, xlab = 'Date', ylab = 'Number of Cows in Attendance', main = 'Cow Attendance Rates Across Days',  alpha=I(.5)) + geom_hline(yintercept = 150) + geom_vline(xintercept = 17182 + 55) + geom_vline(xintercept = 17363 - 7)
```

```
sum(cowattendance>=150)
```

```
## [1] 103
```

Finally I need to cull these records down to remove any mucky days o.o

Firstly, Ill remove any of the trial days where at least 3/4 of the cows (150) are recorded at a given milking.

The start of the trial was Jan 16 (17182). I want to drop any columns that are less than 55 days into the trial (before all cows were enrolled, so before the final herd heirarchy could be fully established).

Also, the last day of the trial was July 16 (17363), and I’ll want to remove the last 7 days of the trial.

Finally, I want to avoid any muckiness that might have come in with the transition to pasture, which occured on April 24 (17280). To that end, I’ll drop the two days before and 4 days following pasture

```
cowattendance_final <- cowattendance[cowattendance >150]
cowattendance_final <- cowattendance_final[as.numeric(names(cowattendance_final)) >= (17182 + 55)]
cowattendance_final <- cowattendance_final[as.numeric(names(cowattendance_final)) < (17363 - 7)]
cowattendance_final <- cowattendance_final[! as.numeric(names(cowattendance_final)) %in% (17280-2):(17280+4)]
length(cowattendance_final)
```

```
## [1] 80
```

```
cowattendance_pen <- cowattendance_final[as.numeric(names(cowattendance_final)) <17280]
length(cowattendance_pen)
```

```
## [1] 26
```

```
cowattendance_pasture <- cowattendance_final[as.numeric(names(cowattendance_final)) > 17280]
length(cowattendance_pasture)
```

```
## [1] 54
```

```
# subsetting data

milkorderdat_all <- milkorderdat[,names(milkorderdat) %in% c('CowID', names(cowattendance_final))]
milkorderdat_pen <- milkorderdat[,names(milkorderdat) %in% c('CowID', names(cowattendance_pen))]
milkorderdat_pasture <- milkorderdat[,names(milkorderdat) %in% c('CowID', names(cowattendance_pasture))]

milkquantdat_all <- milkquantdat[,names(milkquantdat) %in% c('CowID', names(cowattendance_final))]
milkquantdat_pen <- milkquantdat[,names(milkquantdat) %in% c('CowID', names(cowattendance_pen))]
milkquantdat_pasture <- milkquantdat[,names(milkquantdat) %in% c('CowID', names(cowattendance_pasture))]
```

That leaves us with 80 days of quality milk order observations. Of these 26 come from the pen period and 54 from the pasture period.

```
# retain the pre-cull records
milkorderdat_all_unculled <- milkorderdat_all
milkquantdat_all_unculled <- milkquantdat_all


cowattendance_bycow <- apply(milkorderdat_all, 1, function(x) sum(is.na(x)))
names(cowattendance_bycow)<- milkorderdat_all$CowID

qplot(cowattendance_bycow, geom="histogram", xlab = 'Number of Milkings Recorded as Missing', 
      ylab = '', main = 'Distribution of Milking Attendance Rates Across Cows', 
      col=I("black"), fill=I("blue"), alpha=I(.5), bins = 20)  + 
  geom_vline(xintercept = 15)
```

## Aggregate Milking Yield Outcomes

First, I’ll pulling milking 1 yeild information

```
milkingyielddat <- data.frame(CowID = cowlist_complete)

for(i in 1:length(orderdata_infered)){
  if(orderdata_infered[[i]][['Edited']]){
    
    dat <- orderdata_infered[[i]][['data_subset']]
    dat2 <- data.frame(CowID = dat[,1])
    dat2$MilkingOrder <- dat$MilkingYield
    
    milkingyielddat <- merge(milkingyielddat, dat2, by = 'CowID', all.x = T)
    names(milkingyielddat)[ncol(milkingyielddat)] <- orderdata_infered[[i]][['DateN']]
    
    if(is.na(orderdata_infered[[i]][['DateN']])){
      print(i)
    }
    
  }
}

#table(names(milkingyielddat))

for(i in 1:length(orderdata_indexed)){
  if(orderdata_indexed[[i]][['Edited']]){
    
    dat <- orderdata_indexed[[i]][['data_subset']]
    dat2 <- data.frame(CowID = dat[,1])
    dat2$MilkingOrder <- dat$MilkingYield
    
    milkingyielddat <- merge(milkingyielddat, dat2, by = 'CowID', all.x = T)
    names(milkingyielddat)[ncol(milkingyielddat)] <- orderdata_indexed[[i]][['DateN']]
    
  }
  
  
}

# reorder columns by date

temp <- milkingyielddat[,-1]
temp2 <- as.numeric(names(temp))
milkingyielddat <- cbind(milkingyielddat$CowID, temp[,order(temp2)])
names(milkingyielddat)[1] <- 'CowID' 

# cull cow and days

milkingyielddat_all <- milkingyielddat[ , names(milkingyielddat) %in% names(milkorderdat_all)]

milkingyielddat_all_unculled <- milkingyielddat_all
milkingyielddat_all <- milkingyielddat_all[milkingyielddat_all$CowID %in% milkorderdat_all$CowID , ]

milkingyielddat_pen <- milkingyielddat_all[ , names(milkingyielddat_all) %in% names(milkorderdat_pen)]
milkingyielddat_pasture <- milkingyielddat_all[ , names(milkingyielddat_all) %in% names(milkorderdat_pasture)]
```

# Data Wrangling - Cow Attributes

First, I need to pull together a bunch of the cow attribute fields from various data sets.

## Health Status

I’ll begin by looking at the sick lists.

```
sickdat <- read.csv('Data/sick_cows_organilac.csv', stringsAsFactors = F)
sickdat$DIGNOSIS[sickdat$DIGNOSIS == ''] <- NA
sickdat$DATE.SICK[sickdat$DATE.SICK == ''] <- NA 

sickdat$Date <- as.Date(sickdat$DATE.SICK, format = '%m/%d/%y')
sickdat$DateN <- as.numeric(sickdat$Date)  

startdate <- min(as.numeric(names(milkquantdat_all)[-1]))
pasturedate <- 17280
sickdat$Sick_Pasture <- sickdat$Sick  &  sickdat$DateN > 17280
sickdat$Sick_Pen <- sickdat$Sick  &  sickdat$DateN < pasturedate  &  sickdat$DateN > startdate
sickdat$Sick_BurnIn <-  sickdat$Sick  &  sickdat$DateN < startdate

cowlist_healthy_all <- sickdat$ID[sickdat$Sick == 0]
length(cowlist_healthy_all)
```

```
## [1] 136
```

ok, now I want to make a new culled milkquant dataframe that contains only the cows that are verified healthy. So, I’ll be keeping only cows that never got sick, and who were logged in at least 75% of observed milkings.

```
milkquantdat_all2 <- milkquantdat[milkquantdat$CowID %in% cowlist_healthy_all, ] # remove sicks
milkquantdat_all2 <- milkquantdat_all2[ , names(milkquantdat_all2) %in% names(milkquantdat_all)] # drop wonky days

rownames(milkquantdat_all2) <- milkquantdat_all2$CowID
milkquantdat_all2 <- milkquantdat_all2[,-which(names(milkquantdat_all2) == 'CowID')]

temp <- apply(!is.na(milkquantdat_all2), 1, function(x) sum(x, na.rm = T))/ncol(milkquantdat_all2)
milkquantdat_all2 <- milkquantdat_all2[temp >= 0.75, ] # cull cows with excessive missing records
dim(milkquantdat_all2)
```

```
## [1] 114  80
```

```
milkquantdat_pen2 <- milkquantdat_all2[ , as.numeric(names(milkquantdat_all2)) < pasturedate]
dim(milkquantdat_pen2)
```

```
## [1] 114  26
```

```
milkquantdat_pasture2 <- milkquantdat_all2[ , as.numeric(names(milkquantdat_all2)) > pasturedate]
dim(milkquantdat_pasture2)
```

```
## [1] 114  54
```

I also want to make a dataset with both healthy and sick cows present, but I still need cows that stuck around to get a reasonable sense of their position in the herd, so here I’ll retain all cows that showed up for at least 50% of the milkings

```
milkquantdat_sickhealthy <- milkquantdat[,names(milkquantdat) %in% names(milkquantdat_all2)]
rownames(milkquantdat_sickhealthy) <- milkquantdat$CowID

temp <- apply(milkquantdat_sickhealthy, 1, function(x) sum(!is.na(x))/ncol(milkquantdat_sickhealthy))
milkquantdat_sickhealthy <- milkquantdat_sickhealthy[temp>=0.5,]
dim(milkquantdat_sickhealthy)
```

```
## [1] 177  80
```

## Birth Date

```
birthdatedat_master <- read.csv('Data/Birthdates-aurora.CSV', stringsAsFactors = F)
birthdatedat_master$Date <- as.Date(birthdatedat_master$BrthDate, format = '%m/%d/%y')
names(birthdatedat_master)[1] <- 'CowID' 
birthdatedat_master$CowID <- gsub(' ', '', birthdatedat_master$CowID)

birthdatedat <- merge(data.frame(CowID = sickdat$ID), birthdatedat_master, by = 'CowID', all.x = T)

for(i in 1:nrow(birthdatedat)){
  
  if(is.na(birthdatedat$Date[i])){
    temp <- which(paste(birthdatedat$CowID[i],'*', sep='') == birthdatedat_master$CowID)
    if(length(temp) > 0){
      birthdatedat$BrthDate[i] <- birthdatedat_master$BrthDate[temp]
      birthdatedat$Date[i] <- birthdatedat_master$Date[temp]
    }
  }
  
}
 
birthdatedat$DateN <- min(as.numeric(names(milkquantdat[,-1]))) - as.numeric(birthdatedat$Date) # age of cow on the first day of cow enrollment
```

## Calving Date

Now I want to get the date that cows entered the herd using their calving date records. I’ll also pull their experimental treatment group.

```
milk150 <- read.csv('Data/Milk Yeild 150 DIM.csv', stringsAsFactors = F, na.strings = '.')
experdat <- unique(milk150[,-c(2,3,4, 6, 8)])
names(experdat) <- c('CowID','Group','CalvingDate')

experdat$CalvingDateF <- as.Date(experdat$CalvingDate, format = '%m/%d/%y', tz = 'MST')
experdat$CalvingDateN <- as.numeric(experdat$CalvingDateF) - min(as.numeric(experdat$CalvingDateF), na.rm = T)
```

## Milk Yield

And now, finally, I want to look at their milk yield. This data set is challenging to work with, as it is not validated test day records but noisy parlor records. There are a lot of missing entries from failed readers, and also a lot of low-ball yields when cows knock their claws off one or more times on a given day. That will make fitting the actual curve to the data dubious at best. So I think I am just simply going to pull off the 95% quantile milking record and use that as a robust estimate of peak yield.

```
milk150dat <- data.frame(CowID = sickdat$ID, MilkYield95 = NA)

for(i in 1:nrow(milk150dat)){
  
  temp <- subset(milk150$MILKDAY, milk150$ID == milk150dat$CowID[i])
  milk150dat$MilkYield95[i] <- quantile(temp, probs = 0.95, na.rm = T)
  
}
```

## Compiling Cow Attributes

```
cowattribdat <- birthdatedat[ ,c('CowID', 'BrthDate', 'DateN')]
names(cowattribdat)[3] <- 'AgeDaysOld'

cowattribdat <- merge(cowattribdat, milk150dat, by = 'CowID', all.x = T)

temp <- experdat[ ,c('CowID', 'CalvingDate', 'CalvingDateN')]
cowattribdat <- merge(cowattribdat, temp, by = 'CowID', all.x = T)

temp <- sickdat[ , c('ID' ,'group', "Sick", "Sick_BurnIn", "Sick_Pen", "Sick_Pasture", "DIGNOSIS")]
names(temp)[1] <- 'CowID' 
cowattribdat <- merge(cowattribdat, temp, by = 'CowID', all.x = T)
cowattribdat$Sick <- ifelse(cowattribdat$Sick == 1, TRUE, FALSE)
```

# Data Wrangling - Sensor Data

The first sensor data set I want to bring in is the herd manager data set, and get the columns formatted.

```
HerdMan_master <- read.csv('Data/Sensor Data/HerdManager_Masterlist.csv', header = T, stringsAsFactors = F)
HerdMan_master$TimeStampF <- strptime(HerdMan_master$TimeStamp, format = '%Y%m%d %H:%M:%S', tz = 'MST')
HerdMan_master$Date <- as.Date(HerdMan_master$TimeStampF)
head(HerdMan_master)
```

```
##   Id         TimeStamp MeasurementCount NotActive Ruminating Eating Active
## 1  5 20170112 22:00:00               60        14          0     31      6
## 2  5 20170112 23:00:00               60        20         13     17      3
## 3  5 20170113 00:00:00               60        26         33      0      1
## 4  5 20170113 01:00:00               60        18         36      1      2
## 5  5 20170113 02:00:00               60         0          3     49      0
## 6  5 20170113 03:00:00               60        15         31      0     10
##   HighActive Temperature          TimeStampF       Date
## 1          9        9.50 2017-01-12 22:00:00 2017-01-12
## 2          7        9.61 2017-01-12 23:00:00 2017-01-12
## 3          0       11.13 2017-01-13 00:00:00 2017-01-13
## 4          3       10.25 2017-01-13 01:00:00 2017-01-13
## 5          8        7.78 2017-01-13 02:00:00 2017-01-13
## 6          4       10.03 2017-01-13 03:00:00 2017-01-13
```

Next, I want to cull down the datelist so that I only look at dates after the herd has been fully established and prior to the herd leaving the supervised trial. As with the social networks, I’ll only start looking at data from 55 days into the start of the trial on Jan 16th (17182+55) and since I really don’t have that many pasture observations, I’ll go ahead and cull any days after the cows were let out to pasture on April 24 (17280)

```
peddat <- subset(HerdMan_master, as.numeric(HerdMan_master$Date) >= (17182+55) & as.numeric(HerdMan_master$Date) <  17280)

#table(peddat$Id)
length(unique(peddat$Date))
```

```
## [1] 43
```

```
table(as.numeric(peddat$Date))
```

```
## 
## 17237 17238 17239 17240 17241 17242 17243 17244 17245 17246 17247 17248 
##  4440  4440  4440  4424  4438  4440  4440  4440  4440  4440  4432  4416 
## 17249 17250 17251 17252 17253 17254 17255 17256 17257 17258 17259 17260 
##  4416  4416  4416  4416  4414  4401  4416  4416  4416  4416  4416  4416 
## 17261 17262 17263 17264 17265 17266 17267 17268 17269 17270 17271 17272 
##  4405  4392  4389  4368  4368  4368  4368  4368  4368  4367  4344  4344 
## 17273 17274 17275 17276 17277 17278 17279 
##  4344  4363  4368  4368  4368  4368  3822
```

That leaves us with 43 days of observations. But it looks like we’ve got one day where several hundred observations are missing, which coincidentally is the day before pasture access, so we’ll drop that one to be safe as well. Leaving 42 observation days

```
peddat <- subset(peddat, as.numeric(peddat$Date) != 17279 )
length(unique(peddat$Date))
```

```
## [1] 42
```

```
table(peddat$Date)
```

```
## 
## 2017-03-12 2017-03-13 2017-03-14 2017-03-15 2017-03-16 2017-03-17 
##       4440       4440       4440       4424       4438       4440 
## 2017-03-18 2017-03-19 2017-03-20 2017-03-21 2017-03-22 2017-03-23 
##       4440       4440       4440       4440       4432       4416 
## 2017-03-24 2017-03-25 2017-03-26 2017-03-27 2017-03-28 2017-03-29 
##       4416       4416       4416       4416       4414       4401 
## 2017-03-30 2017-03-31 2017-04-01 2017-04-02 2017-04-03 2017-04-04 
##       4416       4416       4416       4416       4416       4416 
## 2017-04-05 2017-04-06 2017-04-07 2017-04-08 2017-04-09 2017-04-10 
##       4405       4392       4389       4368       4368       4368 
## 2017-04-11 2017-04-12 2017-04-13 2017-04-14 2017-04-15 2017-04-16 
##       4368       4368       4368       4367       4344       4344 
## 2017-04-17 2017-04-18 2017-04-19 2017-04-20 2017-04-21 2017-04-22 
##       4344       4363       4368       4368       4368       4368
```

I now need to cull any cows without sufficient records. Several cows have fewer than 1000 sensor logs, whereas the majority of cows have 1008, so I’m going to drop these animals for insufficient records. Also, I’ll cull 62002, as it looks like she lost her sensor after the pasture.

```
table(peddat$Id)[table(peddat$Id)<max(table(peddat$Id))]
```

```
## 
##  1839 10500 13920 19484 21906 31889 
##   990   991   256   589   930   645
```

```
peddat <- subset(peddat, !(peddat$Id %in% c(1839, 10500, 13920, 19484, 21906, 31889)))

length(unique(peddat$Id))
```

```
## [1] 179
```

Now I need to format the hour field, and correct for the time zone

```
#peddat$adjtemperdat_night$TimeStampC <- <- as.character(peddat$TimeStampF)
peddat$TimeDiff <- peddat$TimeStampF -  peddat$TimeStampF[1]

peddat$TimeStampC <- as.character(peddat$TimeStampF)
peddat$Hour  <- as.numeric(substring(peddat$TimeStampC, 12,13))

peddat$HourCorrected <- NA
peddat$HourCorrected[peddat$Hour == 0] <- 19
peddat$HourCorrected[peddat$Hour == 1] <- 20
peddat$HourCorrected[peddat$Hour == 2] <- 21
peddat$HourCorrected[peddat$Hour == 3] <- 22
peddat$HourCorrected[peddat$Hour == 4] <- 23
peddat$HourCorrected[peddat$Hour == 5] <- 0
peddat$HourCorrected[peddat$Hour == 6] <- 1
peddat$HourCorrected[peddat$Hour == 7] <- 2
peddat$HourCorrected[peddat$Hour == 8] <- 3
peddat$HourCorrected[peddat$Hour == 9] <- 4
peddat$HourCorrected[peddat$Hour == 10] <- 5
peddat$HourCorrected[peddat$Hour == 11] <- 6
peddat$HourCorrected[peddat$Hour == 12] <- 7
peddat$HourCorrected[peddat$Hour == 13] <- 8
peddat$HourCorrected[peddat$Hour == 14] <- 9
peddat$HourCorrected[peddat$Hour == 15] <- 10
peddat$HourCorrected[peddat$Hour == 16] <- 11
peddat$HourCorrected[peddat$Hour == 17] <- 12
peddat$HourCorrected[peddat$Hour == 18] <- 13
peddat$HourCorrected[peddat$Hour == 19] <- 14
peddat$HourCorrected[peddat$Hour == 20] <- 15
peddat$HourCorrected[peddat$Hour == 21] <- 16
peddat$HourCorrected[peddat$Hour == 22] <- 17
peddat$HourCorrected[peddat$Hour == 23] <- 18

peddat$Hourc <- as.factor(peddat$HourCorrected)

peddat$DateN <- as.numeric(peddat$Date)
peddat$DayOnTrial <- peddat$DateN - 17182
peddat$Datec <- as.factor(peddat$DayOnTrial)
```

Ok, now I want to isolate the cows that I know had no recorded health events from the larger herd, so that I can focus on cows who’s behavior likely wasn’t altered by illness

```
peddat_sickhealthy <- peddat

peddat <- subset(peddat, peddat$Id %in% cowlist_healthy_all)

cowlist_peddat <- unique(peddat$Id)
length(cowlist_peddat)
```

```
## [1] 124
```

Next, in order to caluculate a distance matrix, I need to align this data by the time stamps assigned to each observation across cows. In order to encourage the merges to run faster, I’m going to create an interger column that represent the difference in seconds from the earliest time stamp in this dataset (3-12-2017 00:00:00)

```
uniquedatelist <- unique(peddat[,c(which(names(peddat)=='TimeStampF'),
                                   which(names(peddat)=='TimeDiff'),
                                   which(names(peddat)=='Hour'),
                                   which(names(peddat)=='HourCorrected'))])


# initialize data frames

nonactivedat <- uniquedatelist
nonactivedat <- cbind(nonactivedat, matrix(NA, ncol = length(cowlist_peddat), nrow = length(unique(peddat$TimeDiff))))
names(nonactivedat)[-c(1,2,3,4)] <- cowlist_peddat

activedat <- uniquedatelist
activedat <- cbind(activedat, matrix(NA, ncol = length(cowlist_peddat), nrow = length(unique(peddat$TimeDiff))))
names(activedat)[-c(1,2,3,4)] <- cowlist_peddat

hiactivedat <- uniquedatelist
hiactivedat <- cbind(hiactivedat, matrix(NA, ncol = length(cowlist_peddat), nrow = length(unique(peddat$TimeDiff))))
names(hiactivedat)[-c(1,2,3,4)] <- cowlist_peddat

totactivedat <- uniquedatelist
totactivedat <- cbind(totactivedat, matrix(NA, ncol = length(cowlist_peddat), nrow = length(unique(peddat$TimeDiff))))
names(totactivedat)[-c(1,2,3,4)] <- cowlist_peddat

rumdat <- uniquedatelist
rumdat <- cbind(rumdat, matrix(NA, ncol = length(cowlist_peddat), nrow = length(unique(peddat$TimeDiff))))
names(rumdat)[-c(1,2,3,4)] <- cowlist_peddat

eatdat <- uniquedatelist
eatdat <- cbind(eatdat, matrix(NA, ncol = length(cowlist_peddat), nrow = length(unique(peddat$TimeDiff))))
names(eatdat)[-c(1,2,3,4)] <- cowlist_peddat

temperdat <- uniquedatelist
temperdat <- cbind(temperdat, matrix(NA, ncol = length(cowlist_peddat), nrow = length(unique(peddat$TimeDiff))))
names(temperdat)[-c(1,2,3,4)] <- cowlist_peddat

# fill in data to structured time dataset


for(i in 1:length(cowlist_peddat)){
  
  # join cow's data with structured timestamps data
  
  cowdat <- subset(peddat, peddat$Id == cowlist_peddat[i])
  tempdat <- merge(cowdat, nonactivedat[,c(1,2)], by = 'TimeDiff', all.y = T)
  
  # add cow data to its respective rows in each response set
  
  nonactivedat[ , which(names(nonactivedat)==cowlist_peddat[i])] <- tempdat$NotActive
  activedat[ , which(names(activedat)==cowlist_peddat[i])] <- tempdat$Active
  hiactivedat[ , which(names(hiactivedat)==cowlist_peddat[i])] <- tempdat$HighActive
  rumdat[ , which(names(rumdat)==cowlist_peddat[i])] <- tempdat$Ruminating
  eatdat[ , which(names(eatdat)==cowlist_peddat[i])] <- tempdat$Eating
  temperdat[ , which(names(temperdat)==cowlist_peddat[i])] <- tempdat$Temperature
  totactivedat[ , which(names(totactivedat)==cowlist_peddat[i])]<- tempdat$TotalActivity
  
}
```

And now to break these datasets down into temporal subsets

```
# nonactivedat

nonactivedat_night <- subset(nonactivedat, nonactivedat$Hour %in% c(8,9,10,11,12))
nonactivedat_morning <- subset(nonactivedat, nonactivedat$Hour %in% c(15,16,17,18,19,20))
nonactivedat_af <- subset(nonactivedat, nonactivedat$Hour %in% c(23,0,1,2,3,4))


# activedat

activedat_night <- subset(activedat, activedat$Hour %in% c(8,9,10,11,12))
activedat_morning <- subset(activedat, activedat$Hour %in% c(15,16,17,18,19,20))
activedat_af <- subset(activedat, activedat$Hour %in% c(23,0,1,2,3,4))

# highactivedat

hiactivedat_night <- subset(hiactivedat, hiactivedat$Hour %in% c(8,9,10,11,12))
hiactivedat_morning <- subset(hiactivedat, hiactivedat$Hour %in% c(15,16,17,18,19,20))
hiactivedat_af <- subset(hiactivedat, hiactivedat$Hour %in% c(23,0,1,2,3,4))

# ruminationdat

rumdat_night <- subset(rumdat, rumdat$Hour %in% c(8,9,10,11,12))
rumdat_morning <- subset(rumdat, rumdat$Hour %in% c(15,16,17,18,19,20))
rumdat_af <- subset(rumdat, rumdat$Hour %in% c(23,0,1,2,3,4))

# eatdat

eatdat_night <- subset(eatdat, eatdat$Hour %in% c(8,9,10,11,12))
eatdat_morning <- subset(eatdat, eatdat$Hour %in% c(15,16,17,18,19,20))
eatdat_af <- subset(eatdat, eatdat$Hour %in% c(23,0,1,2,3,4))

# temperature

temperdat_night <- subset(temperdat, temperdat$Hour %in% c(8,9,10,11,12))
temperdat_morning <- subset(temperdat, temperdat$Hour %in% c(15,16,17,18,19,20))
temperdat_af <- subset(temperdat, temperdat$Hour %in% c(23,0,1,2,3,4))
```

# Entropy Analysis

## Entropy by Position

My first goal here is to get a general idea about the shape of the queue structure. I want to know if certain positions within the queue are occupied by a more diverse set of cows than other positions.

Because the rank quantiles were not generated with the same number of cows and each milking, I cannot track entropy by the ordinal entry order. Instead I will bin the quantiles by width 5%.

Within each bin, I’ll idendify the all cows that fall within that quantile range. If this were a completely random process, any one cow would have as much chance as another to fall in that range. So the probability (p) used to generate entroppy will be number of observations for a given cow over the total number of observations in that quantile.

```
dat <- milkquantdat_all2

entropybyquantile <- function(dat, binwidth = 0.05, base = 2){
  
  temp <- seq(0,1,binwidth)
  entout <- data.frame(Quantile_Low = temp[-length(temp)], Quantile_High = temp[-1], Entropy = NA)
  
  for(i in 1:nrow(entout)){
    
    # remove quantile obs not in current bin
    dattemp <- dat
    dattemp[!(dattemp > entout$Quantile_Low[i] & dattemp <= entout$Quantile_High[i])]  <- NA
      
    # calculate entropy
  
    N = sum(!is.na(dattemp))
    cowcounts <- apply(dattemp, 1, function(x) sum(!is.na(x)))
    cowcounts <- cowcounts[cowcounts != 0]
    entout$Entropy[i] <- sum(-(cowcounts/N)  * log(cowcounts/N, base = base))
    
  }
  
  return(entout)
  
}


# Observed Data
entropy_byquantile <- entropybyquantile(milkquantdat_all2)

qplot(entropy_byquantile$Quantile_Low+0.025, entropy_byquantile$Entropy, xlab = 'Queue Quantile', ylab = 'Entropy', main = 'Relationship Between Queue Position and Entropy: All Data', col=I("black"), alpha=I(.5))
```

As expected, the beginning and end of the queue demonstrate the greatest consistency in cows present, particularly the front of the queue.

And now I’d like to compare this to a completely randomized milking order, which I will do here by simple resampling observed milking quantiles amongst cows within each given observation day

```
milkquantdat_all_rand <- milkquantdat_all2

set.seed(61916)
for(i in 1:ncol(milkquantdat_all_rand)){
  datvec <- milkquantdat_all_rand[,i] 
  milkquantdat_all_rand[,i] <- datvec[sample(1:length(datvec))]
}

rownames(milkquantdat_all_rand) <- rownames(milkquantdat_all2)

entropy_byquantile_rand <- entropybyquantile(milkquantdat_all_rand)

qplot(entropy_byquantile_rand$Quantile_Low+0.025, entropy_byquantile_rand$Entropy, xlab = 'Queue Quantile', ylab = 'Entropy', main = 'Relationship Between Queue Position and Entropy: Randomized Data', col=I("black"), alpha=I(.5))
```

```
temp <- rbind(entropy_byquantile, entropy_byquantile_rand)
temp$Data <- as.factor(c(rep('Observed', nrow(entropy_byquantile)), rep('Randomized', nrow(entropy_byquantile_rand))))
temp$Quantile_Low <- temp$Quantile_Low+0.025
               
qplot(Quantile_Low, Entropy, data = temp, colour = Data, geom = 'point', xlab = 'Queue Quantile', ylab = 'Entropy', main = 'Relationship Between Queue Position and Entropy')
```

```
if(isexporting){
  jpeg('Viz/EntropyQueue_Position.jpeg' , width = 7, height = 4, units = 'in', res = 300)
  qplot(Quantile_Low, Entropy, data = temp, colour = Data, geom = 'point', xlab = 'Queue Quantile', ylab = 'Entropy In Cow Attendance', main = 'Relationship Between Queue Position and Cow Entropy')                
  dev.off()
}
```

```
## quartz_off_screen 
##                 2
```

## Entropy By Cow

Next I want to look at entropy at the individual cow level. I want to get an idea of what cows are showing greater consistency in their queue positions. Again, because the number of cows in each milking varries, I’ll bin the quantiles by width 5%. I also want to explore whether cows are more consistent in their queue position would be observed with a completely randomized queueing process. I’ll do this by comparing the observed entropy to entropy values generated by random permutation of the observed data columns

```
#dat <- milkquantdat_all[, -1]
#cowrow <- 1
entropybycow <- function(dat, cowrow, binwidth = 0.05, base = 2, Nboot = 5000){
  # data = data frame of milking quantiles, with obs day in columns and obs cows in rows
  # cowcol = row in data frame in which target cow's data is found
  # binwidth = percentiles at which to generate categorical bins in data
  # base = log base (default 2 for shannon entropy)
  # Nboot = nummber of permutations to run
  
  
  # bin data
  
  datvec <- unlist(dat[cowrow,])  # vector of observed entry quantiles
  
  vec_c <- cut(datvec, breaks = seq(0,1,binwidth))
  bincounts <- table(vec_c)
  bincounts <- bincounts[bincounts!= 0]
  
  ent_obs <- sum(-(bincounts/sum(bincounts))  * log(bincounts/sum(bincounts), base = base))
  
  bootentropy <- rep(NA, Nboot)
  for(i in 1:Nboot){
    
    datr <- apply(dat, 2, sample) # randomize all observed data
    datvecr <- unlist(datr[cowrow,]) # vector of permuted data
    bootsamp <- cut(datvecr, breaks = seq(0,1,binwidth))
    #bootsamp <- sample(levels(vec_c), sum(bincounts), replace = T)
    bootbin <- table(bootsamp)
    bootbin <- bootbin[bootbin != 0]
    
    bootentropy[i] <- sum(-(bootbin/sum(bootbin))  * log(bootbin/sum(bootbin), base = base))
    
  } 
  
  pval <- sum(bootentropy <= ent_obs)/ Nboot
  return(list(ObsEntropy = ent_obs, pval = pval))
  
}

entropy_byquantile <- entropybycow(milkquantdat_all2, 1)
entropy_byquantile
```

```
## $ObsEntropy
## [1] 3.596098
## 
## $pval
## [1] 0
```

Alright, now lets apply this to each cow. I also want to compare the observed entropy values against their median entry position.

```
entropy_bycow <- data.frame(CowID = rownames(milkquantdat_all2), MedianQuantile = NA, ObsEntropy = NA, pval = NA)

set.seed(61916)
for (i in 1:nrow(milkquantdat_all2)){
  
  temp <- entropybycow(milkquantdat_all2, i)
  entropy_bycow$ObsEntropy[i] <- temp$ObsEntropy
  entropy_bycow$pval[i] <- temp$pval
  entropy_bycow$MedianQuantile[i] <- median(unlist(milkquantdat_all2[i,]), na.rm = T)
  
}

entropy_bycow <- entropy_bycow[order(entropy_bycow$MedianQuantile),] # order by median entry position
entropy_bycow
```

```
##     CowID MedianQuantile ObsEntropy   pval
## 47  16829    0.005988024  0.4271423 0.0000
## 22   9048    0.018990385  2.0716437 0.0000
## 11   2053    0.029243766  0.9087705 0.0000
## 89  46769    0.037037037  1.4345388 0.0000
## 76  31867    0.039548023  1.6458697 0.0000
## 31  13267    0.048484848  1.9681388 0.0000
## 95  55482    0.054545455  2.1604018 0.0000
## 77  32607    0.062500000  1.9649723 0.0000
## 8    1454    0.075368018  2.1960630 0.0000
## 40  13836    0.076585060  2.1674823 0.0000
## 67  26451    0.095238095  2.3179143 0.0000
## 39  13814    0.096153846  2.2171848 0.0000
## 103 62002    0.106512605  2.4342001 0.0000
## 3    1090    0.106713557  2.5069271 0.0000
## 57  20311    0.117813573  2.7776909 0.0000
## 30  13077    0.143678161  2.5407810 0.0000
## 20   7057    0.151515152  2.6145792 0.0000
## 14   2457    0.151898734  3.1230529 0.0000
## 17   4562    0.156523054  2.9089826 0.0000
## 91  48576    0.165227273  3.4063184 0.0000
## 48  17001    0.176527677  3.2288309 0.0000
## 34  13460    0.208942017  3.3755696 0.0000
## 32  13407    0.238434983  3.4602815 0.0000
## 110 97907    0.259777757  3.4996757 0.0000
## 81  33908    0.263315474  3.3928514 0.0000
## 92  50307    0.264088476  3.3973006 0.0000
## 73  27335    0.264144621  3.5642737 0.0000
## 72  27221    0.275449102  3.2900183 0.0000
## 53  19185    0.288311688  3.6464546 0.0000
## 109 96023    0.312138728  3.5481601 0.0000
## 112 98739    0.313062912  3.5141277 0.0000
## 41  13933    0.314814815  3.5660070 0.0000
## 29  11690    0.322646104  3.8317577 0.0004
## 19   6635    0.333333333  3.7032981 0.0000
## 45  13956    0.334280303  3.5550529 0.0000
## 71  27050    0.340909091  3.7142806 0.0000
## 75  27423    0.345214896  3.4316089 0.0000
## 114 98777    0.345454545  3.5649155 0.0000
## 28  10985    0.353796844  3.6513684 0.0000
## 93  53933    0.363128492  3.6693632 0.0000
## 80  33513    0.369696970  3.7799074 0.0000
## 97  55524    0.374669266  3.8558151 0.0006
## 58  21292    0.382857143  3.6895455 0.0000
## 25   9363    0.404344512  3.6200048 0.0000
## 88  45877    0.405063291  3.9027599 0.0024
## 4    1135    0.407185629  3.5221335 0.0000
## 101 55785    0.437125749  3.6480509 0.0000
## 26  10318    0.449704142  3.9764115 0.0208
## 86  45551    0.461731602  3.5373625 0.0000
## 56  20216    0.469492471  3.9172519 0.0020
## 43  13946    0.484662577  3.4889688 0.0000
## 105 63811    0.488095238  3.7729847 0.0000
## 44  13952    0.494097222  3.7795663 0.0000
## 15   2558    0.497005988  3.9169282 0.0042
## 61  23049    0.502994012  3.6018197 0.0000
## 96  55516    0.509202454  3.9307296 0.0072
## 2     645    0.511627907  4.0478488 0.1270
## 16   4407    0.512658228  3.9936054 0.0372
## 9    1511    0.527389706  3.9968157 0.0384
## 27  10837    0.529273650  3.8412993 0.0000
## 66  26228    0.529920422  3.7035221 0.0000
## 49  18649    0.532323617  3.7148144 0.0000
## 55  19484    0.534161491  3.7649455 0.0000
## 59  21906    0.547486034  3.6133915 0.0000
## 69  26905    0.547619048  3.7216653 0.0000
## 113 98771    0.550483830  3.9506820 0.0112
## 62  23325    0.564705882  3.9561222 0.0128
## 107 65346    0.565732759  4.0484732 0.1260
## 1     443    0.566146574  3.5960978 0.0000
## 35  13467    0.567424242  3.9417169 0.0102
## 50  18781    0.573084815  3.7088520 0.0000
## 70  26934    0.576470588  3.7544067 0.0000
## 102 55832    0.596310028  3.7625023 0.0000
## 52  18828    0.604347826  3.8664038 0.0006
## 10   1951    0.604503350  4.0662558 0.1730
## 18   6477    0.621301775  3.8279893 0.0000
## 87  45724    0.633540373  3.9059194 0.0026
## 82  34350    0.635593220  3.9453279 0.0102
## 6    1421    0.639240506  3.7949977 0.0000
## 33  13429    0.646276323  3.8719988 0.0010
## 51  18802    0.646350071  3.6380534 0.0000
## 106 64879    0.653631285  3.4836932 0.0000
## 85  34369    0.662721893  3.7433006 0.0000
## 21   7941    0.662843804  3.4776687 0.0000
## 54  19403    0.675346288  3.3446118 0.0000
## 99  55648    0.689842586  3.6989984 0.0000
## 98  55562    0.691116034  3.7719384 0.0000
## 7    1424    0.693896298  3.8154535 0.0000
## 63  23428    0.699386503  3.4761007 0.0000
## 60  22179    0.708321006  3.3044136 0.0000
## 5    1371    0.753086420  3.6470658 0.0000
## 13   2219    0.754282527  3.7699072 0.0000
## 108 65469    0.761006289  3.3288034 0.0000
## 79  33199    0.772166105  3.7021451 0.0000
## 100 55773    0.772455090  3.6591841 0.0000
## 111 97933    0.775641026  3.4991549 0.0000
## 36  13482    0.776767677  3.5922723 0.0000
## 83  34360    0.784090909  3.3103727 0.0000
## 64  25846    0.787795808  3.4197519 0.0000
## 38  13812    0.802432173  3.1579871 0.0000
## 90  46926    0.806629834  3.5005693 0.0000
## 12   2134    0.827609005  2.9571895 0.0000
## 24   9323    0.837058081  2.8022203 0.0000
## 84  34368    0.852272727  3.2298312 0.0000
## 78  32949    0.857142857  3.0030557 0.0000
## 65  26067    0.863354037  2.7890778 0.0000
## 37  13496    0.871943948  2.6929299 0.0000
## 68  26853    0.873101358  2.8500721 0.0000
## 42  13936    0.881837552  2.8480039 0.0000
## 74  27340    0.904609775  2.6042790 0.0000
## 46  16229    0.932098765  1.8788198 0.0000
## 23   9130    0.934640523  2.2315030 0.0000
## 104 63530    0.946107784  2.0191120 0.0000
## 94  55454    0.971582653  1.5759321 0.0000
```

```
entropy_bycow$PValue <- factor(ifelse(entropy_bycow$pval < 0.05, '<0.05', '>0.05'), levels = c('>0.05','<0.05'))
qplot(MedianQuantile, ObsEntropy, data =entropy_bycow, colour = PValue,  xlab = 'Median Parlor Entry Quantile', ylab = 'Cow Entropy', main = 'Relationship Between Queue Position and Cow Entropy')
```

```
if(isexporting){
  jpeg('Viz/EntropyQueue_Cow.jpeg' , width = 8, height = 6, units = 'in', res = 300)
  qplot(MedianQuantile, ObsEntropy, data =entropy_bycow, colour = PValue,  xlab = 'Median Parlor Entry Quantile', ylab = 'Entropy In Entry Position', main = 'Relationship Between Cow Entropy and Median Parlor Entry Position') 
  dev.off()
}
```

```
## quartz_off_screen 
##                 2
```

Nearly all of these cows have a lower observed entropy than if this were a purely random process, and those pvals that weren’t significant were still quite low. So I feel confident saying that there this isn’t a purely random behavior for the overwhelming majority of animals in this herd.

And now I’m curious what this looks like using variance, which is scale dependent and sensitive to outliers.

```
varbycow <- function(dat, cowrow, Nboot = 5000){
  # data = data frame of milking quantiles, with obs day in columns and obs cows in rows
  # cowcol = row in data frame in which target cow's data is found
  # Nboot = nummber of permutations to run
  
  
  # bin data
  
  datvec <- unlist(dat[cowrow,])  # vector of observed entry quantiles
  var_obs <- var(datvec, na.rm = T)
  
  bootentropy <- rep(NA, Nboot)
  for(i in 1:Nboot){
    
    datr <- apply(dat, 2, sample) # randomize all observed data
    datvecr <- unlist(datr[cowrow,]) # vector of permuted data
    bootentropy[i] <- var(datvecr, na.rm = T)
    
  } 
  
  pval <- sum(bootentropy <= var_obs)/ Nboot
  return(list(ObsVar= var_obs, pval = pval))
  
}

#varbycow(milkquantdat_all2, 1)

var_bycow <- data.frame(CowID = rownames(milkquantdat_all2), MedianQuantile = NA, ObsVar = NA, pval = NA)


set.seed(61916)
dat <- milkquantdat_all2
for (i in 1:nrow(dat)){
  
  temp <- varbycow(dat, i)
  var_bycow$ObsVar[i] <- temp$ObsVar
  var_bycow$pval[i] <- temp$pval
  var_bycow$MedianQuantile[i] <- median(unlist(dat[i, ]), na.rm = T)
  
}


var_bycow <- var_bycow[order(var_bycow$MedianQuantile),] # order by median entry position
var_bycow
```

```
##     CowID MedianQuantile      ObsVar   pval
## 47  16829    0.005988024 0.002799930 0.0000
## 22   9048    0.018990385 0.038908586 0.0000
## 11   2053    0.029243766 0.002545463 0.0000
## 89  46769    0.037037037 0.015915019 0.0000
## 76  31867    0.039548023 0.004599390 0.0000
## 31  13267    0.048484848 0.016890939 0.0000
## 95  55482    0.054545455 0.014294397 0.0000
## 77  32607    0.062500000 0.009463747 0.0000
## 8    1454    0.075368018 0.010004869 0.0000
## 40  13836    0.076585060 0.005276917 0.0000
## 67  26451    0.095238095 0.010269918 0.0000
## 39  13814    0.096153846 0.009666852 0.0000
## 103 62002    0.106512605 0.007964321 0.0000
## 3    1090    0.106713557 0.012289253 0.0000
## 57  20311    0.117813573 0.021049609 0.0000
## 30  13077    0.143678161 0.010158280 0.0000
## 20   7057    0.151515152 0.018694346 0.0000
## 14   2457    0.151898734 0.019684020 0.0000
## 17   4562    0.156523054 0.018040636 0.0000
## 91  48576    0.165227273 0.041492770 0.0000
## 48  17001    0.176527677 0.029689148 0.0000
## 34  13460    0.208942017 0.023058411 0.0000
## 32  13407    0.238434983 0.031564618 0.0000
## 110 97907    0.259777757 0.034869941 0.0000
## 81  33908    0.263315474 0.018719203 0.0000
## 92  50307    0.264088476 0.027389843 0.0000
## 73  27335    0.264144621 0.029636296 0.0000
## 72  27221    0.275449102 0.024511769 0.0000
## 53  19185    0.288311688 0.029357494 0.0000
## 109 96023    0.312138728 0.032332403 0.0000
## 112 98739    0.313062912 0.029105208 0.0000
## 41  13933    0.314814815 0.033727634 0.0000
## 29  11690    0.322646104 0.040588765 0.0000
## 19   6635    0.333333333 0.041801349 0.0000
## 45  13956    0.334280303 0.035187049 0.0000
## 71  27050    0.340909091 0.036766905 0.0000
## 75  27423    0.345214896 0.018331134 0.0000
## 114 98777    0.345454545 0.030816454 0.0000
## 28  10985    0.353796844 0.033727390 0.0000
## 93  53933    0.363128492 0.040418600 0.0000
## 80  33513    0.369696970 0.038927037 0.0000
## 97  55524    0.374669266 0.042764739 0.0000
## 58  21292    0.382857143 0.031288092 0.0000
## 25   9363    0.404344512 0.033840826 0.0000
## 88  45877    0.405063291 0.044604639 0.0000
## 4    1135    0.407185629 0.023369117 0.0000
## 101 55785    0.437125749 0.029907939 0.0000
## 26  10318    0.449704142 0.070526896 0.0404
## 86  45551    0.461731602 0.026015425 0.0000
## 56  20216    0.469492471 0.044918838 0.0000
## 43  13946    0.484662577 0.025415271 0.0000
## 105 63811    0.488095238 0.035202680 0.0000
## 44  13952    0.494097222 0.035601501 0.0000
## 15   2558    0.497005988 0.046000337 0.0000
## 61  23049    0.502994012 0.026883318 0.0000
## 96  55516    0.509202454 0.046318245 0.0000
## 2     645    0.511627907 0.061398949 0.0038
## 16   4407    0.512658228 0.064313986 0.0074
## 9    1511    0.527389706 0.050301494 0.0000
## 27  10837    0.529273650 0.083299853 0.3978
## 66  26228    0.529920422 0.033639526 0.0000
## 49  18649    0.532323617 0.031493288 0.0000
## 55  19484    0.534161491 0.033164555 0.0000
## 59  21906    0.547486034 0.030833561 0.0000
## 69  26905    0.547619048 0.032811343 0.0000
## 113 98771    0.550483830 0.046402943 0.0000
## 62  23325    0.564705882 0.050754453 0.0000
## 107 65346    0.565732759 0.067524293 0.0214
## 1     443    0.566146574 0.028097534 0.0000
## 35  13467    0.567424242 0.056246305 0.0002
## 50  18781    0.573084815 0.044351090 0.0000
## 70  26934    0.576470588 0.030613669 0.0000
## 102 55832    0.596310028 0.035549912 0.0000
## 52  18828    0.604347826 0.056076652 0.0004
## 10   1951    0.604503350 0.053301652 0.0000
## 18   6477    0.621301775 0.047535369 0.0000
## 87  45724    0.633540373 0.062249924 0.0028
## 82  34350    0.635593220 0.051338677 0.0000
## 6    1421    0.639240506 0.045627945 0.0000
## 33  13429    0.646276323 0.043906565 0.0000
## 51  18802    0.646350071 0.034568321 0.0000
## 106 64879    0.653631285 0.025008778 0.0000
## 85  34369    0.662721893 0.038268307 0.0000
## 21   7941    0.662843804 0.027491451 0.0000
## 54  19403    0.675346288 0.017049539 0.0000
## 99  55648    0.689842586 0.034079140 0.0000
## 98  55562    0.691116034 0.043545645 0.0000
## 7    1424    0.693896298 0.042240657 0.0000
## 63  23428    0.699386503 0.022710874 0.0000
## 60  22179    0.708321006 0.018965836 0.0000
## 5    1371    0.753086420 0.039742115 0.0000
## 13   2219    0.754282527 0.052762801 0.0000
## 108 65469    0.761006289 0.019618077 0.0000
## 79  33199    0.772166105 0.052985848 0.0000
## 100 55773    0.772455090 0.042425240 0.0000
## 111 97933    0.775641026 0.036457662 0.0000
## 36  13482    0.776767677 0.034859975 0.0000
## 83  34360    0.784090909 0.022994649 0.0000
## 64  25846    0.787795808 0.025305751 0.0000
## 38  13812    0.802432173 0.016452607 0.0000
## 90  46926    0.806629834 0.037511659 0.0000
## 12   2134    0.827609005 0.016805741 0.0000
## 24   9323    0.837058081 0.013464463 0.0000
## 84  34368    0.852272727 0.036927980 0.0000
## 78  32949    0.857142857 0.015510115 0.0000
## 65  26067    0.863354037 0.010643408 0.0000
## 37  13496    0.871943948 0.009584145 0.0000
## 68  26853    0.873101358 0.023567509 0.0000
## 42  13936    0.881837552 0.035565606 0.0000
## 74  27340    0.904609775 0.015565718 0.0000
## 46  16229    0.932098765 0.002946551 0.0000
## 23   9130    0.934640523 0.007410824 0.0000
## 104 63530    0.946107784 0.009061145 0.0000
## 94  55454    0.971582653 0.003108474 0.0000
```

```
var_bycow$PValue <- factor(ifelse(var_bycow$pval < 0.05, '<0.05', '>0.05'), levels = c('>0.05','<0.05'))
qplot(MedianQuantile, ObsVar, data =var_bycow, colour = PValue,  xlab = 'Median Parlor Entry Quantile', ylab = 'Variance In Entry Quantile', main = 'Relationship Between Queue Position and Cow Variance')
```

```
if(isexporting){
  jpeg('Viz/VarQueue_Cow.jpeg' , width = 8, height = 6, units = 'in', res = 300)
  qplot(MedianQuantile, ObsVar, data =var_bycow, colour = PValue,  xlab = 'Median Parlor Entry Quantile', ylab = 'Variance In Entry Quantile', main = 'Relationship Between Cow Variance and Median Parlor Entry Position') 
  dev.off()
}
```

```
## quartz_off_screen 
##                 2
```

And now, lets compare both these results to parametric tests for differences in queue position. First, I’ll fit cow means as fixed effects, and use beta tests to identify significantly non-random queueing positions.

# Dimension Reduction

### PCA

```
corout <- cor(milkquantdat_all2, use = "pairwise.complete.obs")
pcaout <- eigen(corout)

plot(1:length(pcaout$values), pcaout$values, xlab = 'Dimension', ylab = 'Eigen Value', main = 'Scree Plot')
```

```
loadingmat <- matrix(NA, nrow = nrow(milkquantdat_all2), ncol= 3)
for(i in 1:nrow(loadingmat)){
  temp1 <- sum(milkquantdat_all2[i,] * pcaout$vectors[,1], na.rm = T)
  temp2 <- sum(milkquantdat_all2[i,] * pcaout$vectors[,2], na.rm = T)
  temp3 <- sum(milkquantdat_all2[i,] * pcaout$vectors[,3], na.rm = T)
  loadingmat[i,] <- c(temp1, temp2, temp3)
}

dat <- milkquantdat_all2
col_by_med <- apply(dat, 1, function(x) median(x, na.rm = T))
col_by_IQR <- apply(dat, 1, function(x) IQR(x, na.rm = T))

legendtitle1 <- list(yref='paper',xref="paper",y=1.05,x=1.1, text="Median",showarrow=F)
legendtitle2 <- list(yref='paper',xref="paper",y=1.05,x=1.1, text="IQR",showarrow=F)


plot_ly(x=loadingmat[,1], y=loadingmat[,2], z=loadingmat[,3], type="scatter3d", mode="markers", marker = list(size = 5), color = col_by_med) %>% layout(title ="PCA Embedding of All Data: Dimensions 1,2,3", annotations=legendtitle1)
```

```
plot_ly(x=loadingmat[,1], y=loadingmat[,2], type="scatter", mode="markers", marker = list(size = 5), color = col_by_med) %>% layout(title ="PCA Embedding of All Data: Dimensions 1,2", annotations=legendtitle1)
```

```
plot_ly(x=loadingmat[,1], y=loadingmat[,2], type="scatter", mode="markers", marker = list(size = 5), color = col_by_IQR) %>% layout(title ="PCA Embedding of All Data: Dimensions 1,2", annotations=legendtitle2)
```

```
# checking this result with base package 

# pcaout <- princomp(milkquantdat_all2, cor = T,  na.action = na.exclude)
# plot(1:length(pcaout$sdev), pcaout$sdev, xlab = 'Dimension', ylab = 'Eigen Value', main = 'Scree Plot')
# 
# plot_ly(x=pcaout$scores[,1], y=pcaout$scores[,2], z=pcaout$scores[,3], type="scatter3d", mode="markers", marker = list(size = 5), color = col_by_med) %>% layout(title ="PCA Embedding of All Data: Dimensions 1,2,3", annotations=legendtitle1)
# 
# plot_ly(x=pcaout$scores[,1], y=pcaout$scores[,2], type="scatter", mode="markers", marker = list(size = 5), color = col_by_med) %>% layout(title ="PCA Embedding of All Data: Dimensions 1,2", annotations=legendtitle1)


p1 <- plot_ly(x=loadingmat[,1], y=loadingmat[,2], type="scatter", mode="markers", marker = list(size = 7), color = col_by_med) %>% layout(title ="PCA Embedding of Milking Order Records", annotations=legendtitle1, margin = 1)


#htmlwidgets::saveWidget(as_widget(p1), file = 'PCA.html', selfcontained = T)


if(isexporting & isconda){
  orca(p1, file = 'Viz/PCA.jpeg', scale = 10)
}
```

Some linearity, and some evidence that the there is greater uncertainty in the middle ranks, but its not a super clear geomtry.

## Defining Diffusion Map Function

```
DiffMap <- function(S, k=10, plots = T){
  # returns the eigen values and vectors for a diffusion map computed using similarity matrix S with threshold value k for size of the local network (default is k = 10)
  
  if(nrow(S) != ncol(S)){stop("Error: Similarity matrix should be square")}
  N = nrow(S) 
  
  # Threshold S - retain only the largest k values in each data points row (ie - their local network)
  
  W = matrix(NA, nrow = N, ncol = N) # initialize weight matrix
  
  for(i in 1:N){  # retain top k weights in each row
    rowdat <- S[i,]
    rowrank <- rank(rowdat)
    rowdat[rowrank <= (N-k)] <- 0
    W[i,] <- rowdat
    
  }
  
  for(i in 1:N){ # make weight matrix symmetric
    for(j in 1:N){
      
      if(W[i,j] != 0){
        W[j,i] = W[i,j]
      }
      
    }
  }
  
  # Create the Laplacian
  
  L = matrix(NA, nrow = N, ncol = N) # initialize Laplacian matrix
  
  for(i in 1:N){
    for(j in 1:N){
      if(i == j){
        L[i,j] = sum(W[i,])
      }else{
        L[i,j] = -W[i,j]
      }
    }
  }
  
  # Normalize the weighted Laplacian
  
  D = L + W
  Ln = diag(diag(D)^(-1/2)) %*% L %*% diag(diag(D)^(-1/2))
  
  # Eigen Decompose the Laplacian
  
  eig.out <- eigen(Ln)
  
  # Visualize results of eigen values
  
  if(plots){
    plot(seq(length(eig.out$values),1), eig.out$values, xlab = 'Dimension', ylab = 'Eigen Value', main = 'Eigen Values')
    plot(1:(length(eig.out$values)-1), diff(eig.out$values, lag = 1), xlab = 'Dimension', ylab = 'Eigen Gap', main = 'Gaps Between Eigen Values', pch = as.character(seq((length(eig.out$values)-1),1)) )
  }
  
  return(list(EigenValue = eig.out$values[seq(length(eig.out$values)-1,1)], EigenVectors = eig.out$vectors[,seq(length(eig.out$values)-1,1)])) # return results in order from smallest to largest, dropping the first eigen value/vector (0, and vector of ones)
  
}
```

## Diffusion Map - Signal Distance

Ok, first I want to get a sense of this data set with just a straight up distance matrix calculation. This will take into account both patterns in queue position and relative proximity in the queue. Thus, cows that consistently walk into the parlor near to each other will be embedded next to each other.

```
dat <- milkquantdat_all2
S = as.matrix(1/dist(dat, diag = T, upper = T, method = "euclidean"), nrow = nrow(dat))

diffmat.sig.all <- DiffMap(S)
```

```
# plot by median entry

col_by_med <- apply(dat, 1, function(x) median(x, na.rm = T))
legendtitle1 <- list(yref='paper',xref="paper",y=1.05,x=1.1, text="Median",showarrow=F)

plot_ly(x=diffmat.sig.all$EigenVectors[,1], y=diffmat.sig.all$EigenVectors[,2], z=diffmat.sig.all$EigenVectors[,3], type="scatter3d", mode="markers", marker = list(size = 5), color = col_by_med) %>% layout(title ="Diffusion Map of Observed Qeueu Data: Dimensions 1,2,3", annotations=legendtitle1)
```

```
scene = list(camera = list(eye = list(x = 0, y = -1.3, z = 0.2)))
ay <- list(showticklabels = FALSE, title = '')

p1 <- plot_ly(x=diffmat.sig.all$EigenVectors[,1], y=diffmat.sig.all$EigenVectors[,2], z=diffmat.sig.all$EigenVectors[,3], type="scatter3d", mode="markers", marker = list(size = 5), color = col_by_med) %>% layout(title ="Diffusion Map of Observed Milk Order Records: Dimensions 1,2,3", annotations=legendtitle1, scene = scene, margin = 1)

if(isexporting & isconda){
  orca(p1, file = 'Viz/DiffMap.jpeg', scale = 10)
}
if(isexporting & !isconda){
  suppressWarnings(htmlwidgets::saveWidget(as_widget(p1), file = '/Users/catie/Documents/Research/*PhD/Projects/Milk Order Networks/Round_2/Viz/DiffMap/All_Median_D123.html', selfcontained = T))
}

plot_ly(x=diffmat.sig.all$EigenVectors[,4], y=diffmat.sig.all$EigenVectors[,2], z=diffmat.sig.all$EigenVectors[,3], type="scatter3d", mode="markers", marker = list(size = 5), color = col_by_med) %>% layout(title ="Diffusion Map of All Data: Dimensions 2,3,4", annotations=legendtitle1)
```

```
p2 <- plot_ly(x=diffmat.sig.all$EigenVectors[,4], y=diffmat.sig.all$EigenVectors[,2], z=diffmat.sig.all$EigenVectors[,3], type="scatter3d", mode="markers", marker = list(size = 5), color = col_by_med) %>% layout(title ="Diffusion Map of All Data: Dimensions 2,3,4", annotations=legendtitle1)

if(isexporting & !isconda){
  suppressWarnings(htmlwidgets::saveWidget(as_widget(p2), file = '/Users/catie/Documents/Research/*PhD/Projects/Milk Order Networks/Round_2/Viz/DiffMap/All_Median_D234.html', selfcontained = T))
}

plot_ly(x=diffmat.sig.all$EigenVectors[,4], y=diffmat.sig.all$EigenVectors[,5], z=diffmat.sig.all$EigenVectors[,3], type="scatter3d", mode="markers", marker = list(size = 5), color = col_by_med) %>% layout(title ="Diffusion Map of All Data: Dimensions 3,4,5", annotations=legendtitle1)
```

```
p3 <- plot_ly(x=diffmat.sig.all$EigenVectors[,4], y=diffmat.sig.all$EigenVectors[,5], z=diffmat.sig.all$EigenVectors[,3], type="scatter3d", mode="markers", marker = list(size = 5), color = col_by_med) %>% layout(title ="Diffusion Map of All Data: Dimensions 3,4,5", annotations=legendtitle1)

if(isexporting & !isconda){
  suppressWarnings(htmlwidgets::saveWidget(as_widget(p3), file = '/Users/catie/Documents/Research/*PhD/Projects/Milk Order Networks/Round_2/Viz/DiffMap/All_Median_D345.html', selfcontained = T))
}
```

```
# plot by entry variance

col_by_var<- apply(dat, 1, function(x) IQR(x, na.rm = T))
legendtitle1 <- list(yref='paper',xref="paper",y=1.05,x=1.1, text="IQR",showarrow=F)

plot_ly(x=diffmat.sig.all$EigenVectors[,1], y=diffmat.sig.all$EigenVectors[,2], z=diffmat.sig.all$EigenVectors[,3], type="scatter3d", mode="markers", marker = list(size = 5), color = col_by_var) %>% layout(title ="Diffusion Map of All Data: Dimensions 1,2,3", annotations=legendtitle1)
```

```
p1 <- plot_ly(x=diffmat.sig.all$EigenVectors[,1], y=diffmat.sig.all$EigenVectors[,2], z=diffmat.sig.all$EigenVectors[,3], type="scatter3d", mode="markers", marker = list(size = 5), color = col_by_var) %>% layout(title ="Diffusion Map of All Data: Dimensions 1,2,3", annotations=legendtitle1)

if(isexporting & !isconda){
  suppressWarnings(htmlwidgets::saveWidget(as_widget(p1), file = '/Users/catie/Documents/Research/*PhD/Projects/Milk Order Networks/Round_2/Viz/DiffMap/All_IQR_D123.html', selfcontained = T))
}


plot_ly(x=diffmat.sig.all$EigenVectors[,4], y=diffmat.sig.all$EigenVectors[,2], z=diffmat.sig.all$EigenVectors[,3], type="scatter3d", mode="markers", marker = list(size = 5), color = col_by_var) %>% layout(title ="Diffusion Map of All Data: Dimensions 2,3,4", annotations=legendtitle1)
```

```
p2 <- plot_ly(x=diffmat.sig.all$EigenVectors[,4], y=diffmat.sig.all$EigenVectors[,2], z=diffmat.sig.all$EigenVectors[,3], type="scatter3d", mode="markers", marker = list(size = 5), color = col_by_var) %>% layout(title ="Diffusion Map of All Data: Dimensions 2,3,4", annotations=legendtitle1)

if(isexporting & !isconda){
  suppressWarnings(htmlwidgets::saveWidget(as_widget(p2), file = '/Users/catie/Documents/Research/*PhD/Projects/Milk Order Networks/Round_2/Viz/DiffMap/All_IQR_D234.html', selfcontained = T))
}


plot_ly(x=diffmat.sig.all$EigenVectors[,4], y=diffmat.sig.all$EigenVectors[,5], z=diffmat.sig.all$EigenVectors[,3], type="scatter3d", mode="markers", marker = list(size = 5), color = col_by_var) %>% layout(title ="Diffusion Map of All Data: Dimensions 3,4,5", annotations=legendtitle1)
```

```
p3 <- plot_ly(x=diffmat.sig.all$EigenVectors[,4], y=diffmat.sig.all$EigenVectors[,5], z=diffmat.sig.all$EigenVectors[,3], type="scatter3d", mode="markers", marker = list(size = 5), color = col_by_var) %>% layout(title ="Diffusion Map of All Data: Dimensions 3,4,5", annotations=legendtitle1)

if(isexporting & !isconda){
  suppressWarnings(htmlwidgets::saveWidget(as_widget(p3), file = '/Users/catie/Documents/Research/*PhD/Projects/Milk Order Networks/Round_2/Viz/DiffMap/All_IQR_D345.html', selfcontained = T))
}
```

```
# plot by median milking yield

milkingyielddat_all2 <- milkingyielddat[milkingyielddat$CowID %in% as.numeric(rownames(milkquantdat_all2)),
                                        names(milkingyielddat) %in% names(milkquantdat_all2)]

col_by_yield<- apply(milkingyielddat_all2, 1, function(x) median(x, na.rm = T))
legendtitle1 <- list(yref='paper',xref="paper",y=1.05,x=1.1, text="Milking Yeild",showarrow=F)

plot_ly(x=diffmat.sig.all$EigenVectors[,1], y=diffmat.sig.all$EigenVectors[,2], z=diffmat.sig.all$EigenVectors[,3], type="scatter3d", mode="markers", marker = list(size = 5), color = col_by_yield) %>% layout(title ="Diffusion Map of All Queue Data: Dimensions 1,2,3", annotations=legendtitle1)
```

```
plot_ly(x=diffmat.sig.all$EigenVectors[,4], y=diffmat.sig.all$EigenVectors[,2], z=diffmat.sig.all$EigenVectors[,3], type="scatter3d", mode="markers", marker = list(size = 5), color = col_by_yield) %>% layout(title ="Diffusion Map of All Data: Dimensions 2,3,4", annotations=legendtitle1)
```

```
plot_ly(x=diffmat.sig.all$EigenVectors[,4], y=diffmat.sig.all$EigenVectors[,5], z=diffmat.sig.all$EigenVectors[,3], type="scatter3d", mode="markers", marker = list(size = 5), color = col_by_yield) %>% layout(title ="Diffusion Map of All Data: Dimensions 3,4,5", annotations=legendtitle1)
```

```
# plot by milking yield variance


col_by_yield<- apply(milkingyielddat_all2, 1, function(x) IQR(x, na.rm = T))
legendtitle1 <- list(yref='paper',xref="paper",y=1.05,x=1.1, text="Yield IQR",showarrow=F)

plot_ly(x=diffmat.sig.all$EigenVectors[,1], y=diffmat.sig.all$EigenVectors[,2], z=diffmat.sig.all$EigenVectors[,3], type="scatter3d", mode="markers", marker = list(size = 5), color = col_by_yield) %>% layout(title ="Diffusion Map of All Data: Dimensions 1,2,3", annotations=legendtitle1)
```

```
plot_ly(x=diffmat.sig.all$EigenVectors[,4], y=diffmat.sig.all$EigenVectors[,2], z=diffmat.sig.all$EigenVectors[,3], type="scatter3d", mode="markers", marker = list(size = 5), color = col_by_yield) %>% layout(title ="Diffusion Map of All Data: Dimensions 2,3,4", annotations=legendtitle1)
```

```
plot_ly(x=diffmat.sig.all$EigenVectors[,4], y=diffmat.sig.all$EigenVectors[,5], z=diffmat.sig.all$EigenVectors[,3], type="scatter3d", mode="markers", marker = list(size = 5), color = col_by_yield) %>% layout(title ="Diffusion Map of All Data: Dimensions 3,4,5", annotations=legendtitle1)
```

```
# plot by cow age

temp <- data.frame(CowID = as.numeric(rownames(milkquantdat_all2)))
temp2 <- cowattribdat[,c("CowID", "AgeDaysOld", "CalvingDateN", "group" , "Sick")]
temp3 <- merge(temp, temp2, by = 'CowID', all.x = T)

col_by_age <- temp3$AgeDaysOld
legendtitle1 <- list(yref='paper',xref="paper",y=1.05,x=1.1, text="Cow Age",showarrow=F)

plot_ly(x=diffmat.sig.all$EigenVectors[,1], y=diffmat.sig.all$EigenVectors[,2], z=diffmat.sig.all$EigenVectors[,3], type="scatter3d", mode="markers", marker = list(size = 5), color = col_by_age) %>% layout(title ="Diffusion Map of All Data: Dimensions 1,2,3", annotations=legendtitle1)
```

```
# plot by calving date

col_by_enroll <- temp3$CalvingDateN
legendtitle1 <- list(yref='paper',xref="paper",y=1.05,x=1.1, text="Calving Date",showarrow=F)


plot_ly(x=diffmat.sig.all$EigenVectors[,1], y=diffmat.sig.all$EigenVectors[,2], z=diffmat.sig.all$EigenVectors[,3], type="scatter3d", mode="markers", marker = list(size = 5), color = col_by_enroll ) %>% layout(title ="Diffusion Map of All Data: Dimensions 1,2,3", annotations=legendtitle1, scene = scene)
```

This mapping reveals a highly linear underlying geometry. It seems to have identified for each cow the center around which their entry obs are scattered (~ median) and ordered them. Flaring along the middle of the line suggests that there is greater uncertainty in position in the middle of the queue. Cows with greater rank uncertainty seem to be pulled towards the certer away from the line.

Its impossible to say wether this might reflect linearity in the underying hierarchy, or is simply an artifact of the linearization process.

There doesn’t seem to be any clear relationship to median milking yield, but cows with more variable milking yields (inter-quartile range) might be pulled slightly towards the center of the geometry.

Surprisingly, I don’t see any super clear trends related to id rank, which should roughly reflect cow age. I do see that the front and end of the queue are occupied by middle to older-aged cows, with the lighter shades not showing up to you move into the more medial ranks. But there are still plenty of older cows with low ear tag numbers filling in the middle ranks. I am curious how well age correlates to rank in domestic settings, where generational structures are not preserved. I’d expect size and fitness to be stronger determinates, but it seems quite possible that a older cow that has picked up any permenant physical handicaps (expl - feet issues, poor body condition, etc) could end up on par with respect to dominance to a hiefer. And of course, there is personality to consider.

Now, I’m curious to see how well this geometry replicates when broken down into pen and pasture sub-sets.

```
# pen data

dat <- milkquantdat_pen2
S = as.matrix(1/dist(dat, diag = T, upper = T, method = "euclidean"), nrow = nrow(dat))

diffmat.sig.pen <- DiffMap(S)
```

```
col_by_med <- apply(dat, 1, function(x) median(x, na.rm = T))
legendtitle1 <- list(yref='paper',xref="paper",y=1.05,x=1.1, text="Median",showarrow=F)

plot_ly(x=diffmat.sig.pen$EigenVectors[,1], y=diffmat.sig.pen$EigenVectors[,2], z=diffmat.sig.pen$EigenVectors[,3], type="scatter3d", mode="markers", marker = list(size = 5), color = col_by_med) %>% layout(title ="Diffusion Map of Pen Data: Dimensions 1,2,3", annotations=legendtitle1)
```

```
plot_ly(x=diffmat.sig.pen$EigenVectors[,4], y=diffmat.sig.pen$EigenVectors[,2], z=diffmat.sig.pen$EigenVectors[,3], type="scatter3d", mode="markers", marker = list(size = 5), color = col_by_med) %>% layout(title ="Diffusion Map of Pen Data: Dimensions 2,3,4", annotations=legendtitle1)
```

```
col_by_med <- apply(milkquantdat_pasture2, 1, function(x) median(x, na.rm = T))
legendtitle1 <- list(yref='paper',xref="paper",y=1.05,x=1.1, text="Median Pasture",showarrow=F)

plot_ly(x=diffmat.sig.pen$EigenVectors[,1], y=diffmat.sig.pen$EigenVectors[,2], z=diffmat.sig.pen$EigenVectors[,3], type="scatter3d", mode="markers", marker = list(size = 5), color = col_by_med) %>% layout(title ="Diffusion Map of Pen Data: Dimensions 1,2,3", annotations=legendtitle1)
```

```
# pasture data

dat <- milkquantdat_pasture2
S = as.matrix(1/dist(dat, diag = T, upper = T, method = "euclidean"), nrow = nrow(dat))

diffmat.sig.pasture <- DiffMap(S)
```

```
col_by_med <- apply(dat, 1, function(x) median(x, na.rm = T))
legendtitle1 <- list(yref='paper',xref="paper",y=1.05,x=1.1, text="Median",showarrow=F)

plot_ly(x=diffmat.sig.pasture$EigenVectors[,1], y=diffmat.sig.pasture$EigenVectors[,2], z=diffmat.sig.pasture$EigenVectors[,3], type="scatter3d", mode="markers", marker = list(size = 5), color = col_by_med) %>% layout(title ="Diffusion Map of Pasture Data: Dimensions 1,2,3", annotations=legendtitle1)
```

```
plot_ly(x=diffmat.sig.pasture$EigenVectors[,4], y=diffmat.sig.pasture$EigenVectors[,2], z=diffmat.sig.pasture$EigenVectors[,3], type="scatter3d", mode="markers", marker = list(size = 5), color = col_by_med) %>% layout(title ="Diffusion Map of Pasture Data: Dimensions 2,3,4", annotations=legendtitle1)
```

```
col_by_med <- apply(milkquantdat_pen2, 1, function(x) median(x, na.rm = T))
legendtitle1 <- list(yref='paper',xref="paper",y=1.05,x=1.1, text="Median Pen",showarrow=F)

plot_ly(x=diffmat.sig.pasture$EigenVectors[,1], y=diffmat.sig.pasture$EigenVectors[,2], z=diffmat.sig.pasture$EigenVectors[,3], type="scatter3d", mode="markers", marker = list(size = 5), color = col_by_med) %>% layout(title ="Diffusion Map of Pasture Data: Dimensions 1,2,3", annotations=legendtitle1)
```

Yep, seems to be the same deal. The colorings also suggest the pen and pasture sequences are extremely similar.

Now, I want to compare this to a completely randomized milking order. To do this, I’m simply going to randomize observations within rows

```
if('CowID' %in% names(milkquantdat_all_rand)){
  dat <- milkquantdat_all_rand[,-which(names(milkquantdat_all_rand) == 'CowID')]
}else{
  dat <- milkquantdat_all_rand
}


S = as.matrix(1/dist(dat, diag = T, upper = T, method = "euclidean"), nrow = nrow(dat))

diffmat.sig.rand <- DiffMap(S)
```

```
# plot by median

col_by_med <- apply(dat, 1, function(x) median(x, na.rm = T))
legendtitle1 <- list(yref='paper',xref="paper",y=1.05,x=1.1, text="Median",showarrow=F)

plot_ly(x=diffmat.sig.rand$EigenVectors[,1], y=diffmat.sig.rand$EigenVectors[,2], z=diffmat.sig.rand$EigenVectors[,3], type="scatter3d", mode="markers", marker = list(size = 5), color = col_by_med) %>% layout(title ="Diffusion Map of Randomized Queue Data: Dimensions 1,2,3", annotations=legendtitle1)
```

```
plot_ly(x=diffmat.sig.rand$EigenVectors[,4], y=diffmat.sig.rand$EigenVectors[,2], z=diffmat.sig.rand$EigenVectors[,3], type="scatter3d", mode="markers", marker = list(size = 5), color = col_by_med) %>% layout(title ="Diffusion Map of Randomized Data: Dimensions 2,3,4", annotations=legendtitle1)
```

```
plot_ly(x=diffmat.sig.rand$EigenVectors[,4], y=diffmat.sig.rand$EigenVectors[,5], z=diffmat.sig.rand$EigenVectors[,3], type="scatter3d", mode="markers", marker = list(size = 5), color = col_by_med) %>% layout(title ="Diffusion Map of Randomized Data: Dimensions 3,4,5", annotations=legendtitle1)
```

```
scene = list(camera = list(eye = list(x = -0.1, y = 1.35, z = 0.2)))

p1 <- plot_ly(x=diffmat.sig.rand$EigenVectors[,1], y=diffmat.sig.rand$EigenVectors[,2], z=diffmat.sig.rand$EigenVectors[,3], type="scatter3d", mode="markers", marker = list(size = 5), color = col_by_med) %>% layout(title ="Diffusion Map of Randomized Milk Order Data: Dimensions 1,2,3", annotations=legendtitle1, scene = scene, margin = 1)

if(isexporting & isconda){  
  orca(p1, file = 'Viz/DiffMapRand.jpeg', scale = 10)
}
```

Yep, this geometry certainly isn’t pure artifact, but must be driven by consistent individual differences in queue position, which was shown by the entropy plots.

Finally, I want to see how this structure holds up when we also plot the sick cows..

```
dat <- milkquantdat_sickhealthy
S = as.matrix(1/dist(dat, diag = T, upper = T, method = "euclidean"), nrow = nrow(dat))

diffmat.sig.all.sicks <- DiffMap(S)
```

```
# plot by median entry

col_by_med <- apply(dat, 1, function(x) median(x, na.rm = T))
legendtitle1 <- list(yref='paper',xref="paper",y=1.05,x=1.1, text="Median",showarrow=F)

plot_ly(x=diffmat.sig.all.sicks$EigenVectors[,1], y=diffmat.sig.all.sicks$EigenVectors[,2], z=diffmat.sig.all.sicks$EigenVectors[,3], type="scatter3d", mode="markers", marker = list(size = 5), color = col_by_med) %>% layout(title ="Diffusion Map of Observed Qeueu Data: Dimensions 1,2,3", annotations=legendtitle1)
```

```
# plot by sick vs healthy

col_by_health <- ifelse(rownames(milkquantdat_sickhealthy) %in% rownames(milkquantdat_all2), 'Healthy', 'Sick')
legendtitle1 <- list(yref='paper',xref="paper",y=1.05,x=1.1, text="Health Status",showarrow=F)

plot_ly(x=diffmat.sig.all.sicks$EigenVectors[,1], y=diffmat.sig.all.sicks$EigenVectors[,2], z=diffmat.sig.all.sicks$EigenVectors[,3], type="scatter3d", mode="markers", marker = list(size = 5), color = col_by_health) %>% layout(title ="Diffusion Map of Observed Qeueu Data: Dimensions 1,2,3", annotations=legendtitle1)
```

```
## Warning in RColorBrewer::brewer.pal(N, "Set2"): minimal value for n is 3, returning requested palette with 3 different levels

## Warning in RColorBrewer::brewer.pal(N, "Set2"): minimal value for n is 3, returning requested palette with 3 different levels
```

These sicks look pretty evenly distributed about the queue to me.

## Defining Function to Estimate Harmonic Artifact

Equations for harmonics of eigen vectors of path graph

http://www.cs.yale.edu/homes/spielman/561/2009/lect02-09.pdf

```
makeRefLine <- function(N, dims){
  
  out <- matrix(NA, nrow=N, ncol = dims)
  
  u = seq(1, N, 1)
  for(k in 1:dims){
    v = -cos((pi*k*u)/N-(pi*k)/(2*N)) # virbrational moment 1 (ie - egien vector k)
    v1 = (1/sum(v^2)^0.5) * v # normalize eigen vector
    out[,k] <- v1
  }
  
  return(out)
}

# check bases

temp<- makeRefLine(135, 5)
plot_ly(x=temp[,1], y=temp[,2], z=temp[,3], type="scatter3d", mode="markers", marker = list(size = 5)) %>% layout(title ="Diffusion Map of Simulated Data: Dimensions 1,2,3", annotations=legendtitle1)
```

```
plot_ly(x=temp[,4], y=temp[,2], z=temp[,3], type="scatter3d", mode="markers", marker = list(size = 5)) %>% layout(title ="Diffusion Map of Simulated Data: Dimensions 2,3,4", annotations=legendtitle1)
```

To pull off some summary statistics, I first need to figure out which cow should be assigned to each reference position. To get a linear rank order would require some form of optimization, but I don’t want to impose linearity where it is not necessarily present. Therefore, I’ll simple assign each cow to its closest reference point with respect to euclidean distance. The identifity of that reference point will provide an estimate of their position along the line. I’ll also pull off the euclidean distance between cow point and reference point in each dimension as an estimate of variance.

```
# rawdata <- milkquantdat_all_imp[,-1]
# embedding_data <- embedding_data
# dims <- 3
# mycowlist <- milkquantdat_pasture_imp$CowID
# flip = T

DiffMapStats <- function(rawdata, embedding_data, dims, mycowlist, plottitle = '', ...){
  #Input
  #   rawdata = the data used to create the embedding (and only those colums) with corresponding rows
  #   embedding_data = eigen vectors from diffusion map embedding of real data (row is animal)
  #   dims = number of dimensions to consider in this 
  #   k = size of the local network used to create the embedding
  
  dat <- embedding_data[,1:dims]
  
  # create reference (simulated) data
  
  dat.comp <- makeRefLine(nrow(dat), dims)
  
  # curtail and scale actual data
  
  # dat <- matrix(NA, nrow = nrow(embedding_data), ncol = dims)
  # 
  # for(i in 1:dims){ # normalized eigen vectors from embedding
  #   v <- embedding_data[,i]
  #   s <- sum(v^2)^0.5
  #   dat[,i] <- 1/s * v
  # }
  # 
  # 
  
  # align dims by sign
  
  temp <- apply(rawdata, 1, function(x) median(x, na.rm = T)) 
  dattemp <- dat[order(temp),] # reorder rows embedding data to ascend by median queue position
  
  for(i in 1:dims){ # check if sign should be reversed in any dim
    
    vec1 <- sign(dattemp[,i])
    vec2 <- sign(dat.comp[,i])
    
    notranscount <- sum(vec1 * vec2 < 0) 
    transcount <- sum(-1 * vec1 * vec2 < 0)
    
    if(transcount < notranscount){ # if flipping axis results in fewer mis-aligned signs, then flip
      dat.comp[,i] <- -dat.comp[,i] 
    }
    
  }
  
  
  # check embedding
  
  coltemp <- c(rep(1,nrow(dat.comp)), rep(0,nrow(dat.comp)))
  legendtitle1 <- list(yref='paper',xref="paper",y=1.05,x=1.1, text="Obs/Ref",showarrow=F)

  temp <- rbind(dat, dat.comp)
  p1 <- plot_ly(x=temp[,1], y=temp[,2], z=temp[,3], type="scatter3d", mode="markers", marker = list(size = 5), color = coltemp) %>% layout(title = plottitle, annotations=legendtitle1, showlegend = FALSE, ...)
  
  
  
  # Assign cows to positions - Nonlinear
  
  rank_assign <- data.frame(CowID = mycowlist, Position = NA)
  #rownames(rank_assign) <- cowlist
  
  for(i in 1:nrow(rank_assign)){ # i is the cow
  
    distcomp <- c()
    for(j in 1:nrow(dat.comp)){
       distcomp[j] <- sum((dat.comp[j,] - dat[i,])^2)^0.5
    }
    
    rank_assign$Position[i] <- which.min(distcomp)
    
  }
  
  #plot_ly(x=dat[,1], y=dat[,2], z=dat[,3], type="scatter3d", mode="markers", marker = list(size = 5), color = rank_assign$Position) %>% layout(title ="Diffusion Map of Simulated Data: Dimensions 1,2,3", annotations=legendtitle1)
  #table(rank_assign$Position)
  
  # Extract Distance Measures from reference line
  
  RefDist <- cbind(rank_assign, matrix(NA, nrow = length(mycowlist), ncol = (dims+1)))
  start = which(names(RefDist)=='Position')+1
  names(RefDist)[start] <- 'EuclidDist'
  
  for(i in 1:dims){ # add names for dimesnsion diff variables
    names(RefDist)[start+i] <- paste('Dim',i,sep = '_')
  }
  
  
  for(i in 1:nrow(dat)){ # compute summary statistics
    refpoint <- dat.comp[RefDist$Position[i], ]
    RefDist[i,start] <- sum((refpoint - dat[i,])^2)^0.5 # absolute distance
    RefDist[i,(start+1):ncol(RefDist)] <- refpoint - dat[i,]
  }
  
  return(list(plotout = p1, statout = RefDist))
  
} 

scene = list(camera = list(eye = list(x = 0, y = -1.3, z = 0.35)))

artifact.out <- DiffMapStats(milkquantdat_all2, diffmat.sig.all$EigenVectors, 5, rownames(milkquantdat_all2), plottitle ='Diffusion Map of Observed Milk Order Records: Dimensions 1,2,3', scene = scene, margin = 1)
artifact.out$plotout
```

```
if(isexporting & isconda){
  orca(artifact.out$plotout, file = 'Viz/DiffMapArtif.jpeg', scale = 10)
}
```

# Temporal Dynamics w/ Standard EDA

## Quantile Plots by Cow

First I want to run milk quantile plots colored by the cows milking yield (relative to herself).

```
dat <- milkquantdat_all_unculled
dat2 <- milkingyielddat_all_unculled

if(isexporting){
  for(i in 1:nrow(dat)){
    
    dattemp <- data.frame(Date = as.numeric(names(dat)[-1]), MilkOrder = unlist(dat[i,-1]), MilkingYield = unlist(dat2[i, -1]))
    dattemp <- dattemp[complete.cases(dattemp),]
    
    jpeg(paste('Viz/Milk Order/Quantile Plots/Milking Yield/Cow_', dat$CowID[i], '.jpg' , sep = ''), width = 8, height = 5, units = 'in', res = 300)
    print(ggplot(dattemp, aes(x=Date, y=MilkOrder, colour = MilkingYield)) +
            geom_line(colour='grey') +
            geom_point() + 
            ggtitle(paste('Parlor Entry Quantile: Cow', dat$CowID[i], sep = ' ')) + 
            ylim(0,1) + ylab('Parlor Entry Quantile') + 
            scale_color_viridis() + 
            labs(fill = "Milking Yield") +
            geom_vline(xintercept = 17280, colour='grey') )
    dev.off()
    
  }
}
```

I don’t see many cows with clear trends here. Cows 13467 and cow 13826 are the only visuallly obvious exceptions, both of which move forward in the queue. There are also a few cows interesting runs of high consistency at the end of the pen period, but I don’t think they are a proper temporal shift. One example is 10837

## Comparison of Median Queue Position - Pen vs Pasture

```
median_pen <- apply(milkquantdat_pen2, 1, function(x) median(x, na.rm = T))
median_pasture <- apply(milkquantdat_pasture2, 1, function(x) median(x, na.rm = T))

cor.out <- cor.test(median_pen, median_pasture, method = 'pearson')
cor.out
```

```
## 
##  Pearson's product-moment correlation
## 
## data:  median_pen and median_pasture
## t = 22.97, df = 112, p-value < 2.2e-16
## alternative hypothesis: true correlation is not equal to 0
## 95 percent confidence interval:
##  0.8695757 0.9358327
## sample estimates:
##       cor 
## 0.9082379
```

```
kendal.out <- cor.test(median_pen, median_pasture, method = 'kendall')
kendal.out
```

```
## 
##  Kendall's rank correlation tau
## 
## data:  median_pen and median_pasture
## z = 11.659, p-value < 2.2e-16
## alternative hypothesis: true tau is not equal to 0
## sample estimates:
##       tau 
## 0.7392857
```

```
medcompdat <- data.frame(Pen = median_pen, Pasture = median_pasture, Difference = median_pasture - median_pen)

hist(medcompdat$Difference)
```

```
medcompdat[abs(medcompdat$Difference)>0.2,]
```

```
##             Pen   Pasture Difference
## 645   0.7738971 0.4506173 -0.3232798
## 1371  0.8625000 0.6459627 -0.2165373
## 6477  0.4791124 0.6816772  0.2025648
## 10837 0.8831006 0.4265757 -0.4565249
## 18649 0.3750000 0.5808383  0.2058383
## 23049 0.3296216 0.5605096  0.2308880
## 33199 0.6456312 0.8522116  0.2065803
## 45724 0.8687151 0.5498417 -0.3188733
## 45877 0.5618316 0.3491124 -0.2127192
## 46926 0.9159605 0.7065868 -0.2093736
```

```
ggplot(medcompdat, aes(x=Pen, y=Pasture, colour = Difference)) + #)) + #
    #geom_line(colour='grey') +
    geom_point() + 
    ggtitle('Comparison of Median Entry Quantile Values') + 
    ylim(0,1) + ylab('Pasture Observations') + 
    xlim(0,1) + xlab('Pen Observations') + 
    geom_label(x=0.12, y=0.93, label= paste("Correlation = ", round(cor.out$estimate,2), '\n Kendall Tau = ', round(kendal.out$estimate,2)) , colour = 'black')+ 
    scale_color_viridis()
```

```
  #labs(fill = "Difference") +
  #geom_vline(xintercept = 17280, colour='grey')


if(isexporting){
  jpeg('Viz/Comparison Median Entry Quantile.jpg', width = 8, height = 5, units = 'in', res = 300)
  ggplot(medcompdat, aes(x=Pen, y=Pasture, colour = Difference)) + #)) + #
    #geom_line(colour='grey') +
    geom_point() + 
    ggtitle('Comparison of Median Entry Quantile Values') + 
    ylim(0,1) + ylab('Pasture Observations') + 
    xlim(0,1) + xlab('Pen Observations') + 
    geom_label(x=0.12, y=0.93, label= paste("Correlation = ", round(cor.out$estimate,2), '\n Kendall Tau = ', round(kendal.out$estimate,2)) , colour = 'black')+ 
    scale_color_viridis() 
  #labs(fill = "Difference") +
  #geom_vline(xintercept = 17280, colour='grey')
  dev.off()
}
```

```
## quartz_off_screen 
##                 2
```

# Temporal Dynamics w/ Data Mechanics

## Preliminary Visualizations

First, I want to run independent cluster analyses of the queue records and cow attributes to get a sense of their structure

### Queue Position

```
# All Data

dat <- milkquantdat_all2

d <- dist(dat) # cluster by euclidean distance
h.dist.all <- hclust(d, method = 'ward.D2') 
plot(h.dist.all, xlab = 'Cow ID', main = 'Cluster Dendrogram of All Queueing Records: Euclidean Dist')
```

```
# Pen Data

dat <- milkquantdat_pen2

d <- dist(dat) # cluster by euclidean distance
h.dist.pen <- hclust(d, method = 'ward.D2') 
plot(h.dist.pen, xlab = 'Cow ID', main = 'Cluster Dendrogram of Pen Queueing Records: Euclidean Dist')
```

```
CowID_Pen <- rownames(dat)


# Pasture Data

dat <- milkquantdat_pasture2

d <- dist(dat) # cluster by euclidean distance
h.dist.pasture <- hclust(d, method = 'ward.D2') 
plot(h.dist.pasture, xlab = 'Cow ID', main = 'Cluster Dendrogram of Pasture Queueing Records: Euclidean Dist')
```

```
CowID_Pastures <- rownames(dat)

# Compare Clusterings for Pen vs Pasture

dist.out.pasture <- data.frame(CowID = CowID_Pastures, 
                               Pasture2 = as.numeric(cutree(h.dist.pasture,2)),
                               Pasture3 = as.numeric(cutree(h.dist.pasture,3)),
                               Pasture4 = as.numeric(cutree(h.dist.pasture,4)),
                               Pasture5 = as.numeric(cutree(h.dist.pasture,5)))

dist.out.pen <- data.frame(CowID = CowID_Pen, 
                               Pen2 = as.numeric(cutree(h.dist.pen,2)),
                               Pen3 = as.numeric(cutree(h.dist.pen,3)),
                               Pen4 = as.numeric(cutree(h.dist.pen,4)),
                               Pen5 = as.numeric(cutree(h.dist.pen,5)))

dist.out.all <- merge(dist.out.pen , dist.out.pasture, by = 'CowID')


table(dist.out.all$Pen2, dist.out.all$Pasture2) # contingency table k = 2
```

```
##    
##      1  2
##   1 18 57
##   2 32  7
```

```
table(dist.out.all$Pen3, dist.out.all$Pasture3) # contingency table k = 3
```

```
##    
##      1  2  3
##   1 18 34  1
##   2 32  7  0
##   3  0  5 17
```

```
table(dist.out.all$Pen4, dist.out.all$Pasture4) # contingency table k = 4
```

```
##    
##      1  2  3  4
##   1 15  6  0  0
##   2 19  7  0 13
##   3  0  5 17  0
##   4  3 28  1  0
```

```
table(dist.out.all$Pen5, dist.out.all$Pasture5) # contingency table k = 5
```

```
##    
##      1  2  3  4  5
##   1 15  5  0  0  1
##   2 15  6  0  2  1
##   3  0  1 17  0  4
##   4  3 15  1  0 13
##   5  4  0  0 11  0
```

k = 4 looks like quite a good cut

Overall there’s pretty good agreement between pen and pasture. And some good seperation between broader subgroups.

I also wants to cluster across observation day

```
# All Data

dat <- milkquantdat_all2

d <- dist(t(dat)) # cluster by euclidean distance
h.days.all <- hclust(d, method = 'ward.D2') 
plot(h.days.all, xlab = 'Cow ID', main = 'Cluster of Observation Days: Euclidean Dist')
```

Some seperation in the broader categories. Also it looks like there are some anomolous days - at least two on an extreme branch.

### Cow Attribute Data

First I want to cluster the cow age

```
tempvec <- cowattribdat$AgeDaysOld
names(tempvec) <- cowattribdat$CowID
tempvec <- tempvec[!is.na(tempvec)]
d <- dist(tempvec)

h.age <- hclust(d, method = 'ward.D2') 
plot(h.age, xlab = 'Cow ID', labels = tempvec, main = 'Dendrogram of Cow Age')
```

### Calving Date

```
tempvec <- cowattribdat$CalvingDateN
names(tempvec) <- cowattribdat$CowID
tempvec <- tempvec[!is.na(tempvec)]
d <- dist(tempvec)

h.cdate <- hclust(d, method = 'ward.D2') 
plot(h.cdate, xlab = 'Cow ID', labels = tempvec, main = 'Dendrogram of Calving Date')
```

### Milk Yield

```
tempvec <- cowattribdat$MilkYield95
names(tempvec) <- cowattribdat$CowID
tempvec <- tempvec[!is.na(tempvec)]
d <- dist(tempvec)

h.milkyield <- hclust(d, method = 'ward.D2') 
plot(h.milkyield, xlab = 'Cow ID', labels = round(tempvec,1), main = 'Dendrogram of Peak Milk Yield')
```

## Data Mechanics

### Data Wrangling - Discretize Cow Attributes

First, I need to discretize all my covariates using their dendrogram visualizations

```
DMdat <- data.frame(CowID = cowattribdat$CowID)
rownames(DMdat) <- DMdat$CowID

# Calving Date

temp <- cowattribdat[ , c('CowID','CalvingDate','CalvingDateN')]
rownames(temp) <- temp$CowID
temp <- temp[!is.na(temp$CalvingDateN),]

nclust = 4
temp$CalvingDateC <- cutree(h.cdate, nclust)
temp$CalvingDateT <- temp$CalvingDateC 

for(i in 1:nclust){
  
  temp2 <- subset(temp, temp$CalvingDateC == i)
  stringtemp <- paste(temp2$CalvingDate[which.min(temp2$CalvingDateN)], '-', 
                      temp2$CalvingDate[which.max(temp2$CalvingDateN)], sep = '')
  
  temp$CalvingDateC[temp$CalvingDateC == i] <- stringtemp
  
}

DMdat <- merge(DMdat, temp[,c('CowID','CalvingDateC')], by = 'CowID', all.x = T)

temp2 <- temp[order(temp$CalvingDateN),]
DMdat$CalvingDateC <- factor(DMdat$CalvingDateC, levels = unique(temp2$CalvingDateC), ordered = T)


# Cow Age

temp <- cowattribdat[ , c('CowID', 'BrthDate', 'AgeDaysOld')]
rownames(temp) <- temp$CowID
temp <- temp[!is.na(temp$AgeDaysOld),]

nclust = 5
temp$AgeC<- cutree(h.age, nclust)

for(i in unique(temp$AgeC)){
  
  temp2 <- subset(temp, temp$AgeC == i)
  temp2 <- temp2[!is.na(temp2$BrthDate),]
  stringtemp <- paste(temp2$BrthDate[which.min(temp2$AgeDaysOld)], '-', 
                      temp2$BrthDate[which.max(temp2$AgeDaysOld)], sep = '')
  
  temp$AgeC[temp$AgeC == i] <- stringtemp
  
}

DMdat <- merge(DMdat, temp[,c('CowID','AgeC')], by = 'CowID', all.x = T)

temp2 <- temp[order(temp$AgeDaysOld),] 
DMdat$AgeC <- factor(DMdat$AgeC, levels = unique(temp2$AgeC), ordered = T)


# Milking Yield

temp <- cowattribdat[ , c('CowID','MilkYield95')]
rownames(temp) <- temp$CowID
temp <- temp[!is.na(temp$MilkYield95),]

nclust = 5
temp$MilkYield95C <- cutree(h.milkyield, nclust)

for(i in 1:nclust){
  
  temp2 <- subset(temp, temp$MilkYield95C == i)
  stringtemp <- paste(round(min(temp2$MilkYield95),1), 'lb-', 
                      round(max(temp2$MilkYield95),1), 'lb', sep = '')
  
  temp$MilkYield95C[temp$MilkYield95C == i] <- stringtemp
  
}

DMdat <- merge(DMdat, temp[,c('CowID','MilkYield95C')], by = 'CowID', all.x = T)

temp2 <- temp[order(temp$MilkYield95),] 
DMdat$MilkYield95C <- factor(DMdat$MilkYield95C, levels = unique(temp2$MilkYield95C), ordered = T)


# health attribs

temp <- cowattribdat[,c('CowID','group','Sick','Sick_BurnIn','Sick_Pen','Sick_Pasture','DIGNOSIS')]
DMdat <- merge(DMdat, temp, by = 'CowID', all.x = T)
DMdat$group <- as.factor(DMdat$group)

rownames(DMdat) <- DMdat$CowID
```

## Data Mechanics Visualization - Healthy Cows

For the heatmap viz I’ll use the pheatmap package to get away from this GMD package. For more info, see

https://www.huber.embl.de/msmb/Chap-Graphics.html

http://sape.inf.usi.ch/quick-reference/ggplot2/colour

http://colorbrewer2.org/#type=sequential&scheme=OrRd&n=6

```
dmplot <- function(data.norm, n.row.clusters = 1, n.col.clusters = 1, hclust_method = "ward.D2", maxreps = 10, verbose = FALSE, display.plot = TRUE, export.plot = TRUE, imwidth = 10, imheight = 15, imres = 300, plot_title = 'Data Mechanics Plot', filename = 'DatMechPlotOut', ...){
  
  require("pheatmap")
  require("RColorBrewer")
  require('dplyr')
  
  # prepare data
  
  clusters.col.out <- list()
  clusters.row.out <- list()
  
  # initial cluster
  
  hclustr <- hclust(dist(data.norm), method = hclust_method)
  clusters.row.out[[1]] <- cutree(hclustr, n.row.clusters)
  hclustc <- hclust(dist(t(data.norm)), method = hclust_method)
  clusters.col.out[[1]] <- cutree(hclustc, n.col.clusters)

  if(verbose){
    jpeg(paste(filename,'_V0.jpeg'), width = imwidth, height = imheight, units = 'in', res = imres)
    print(pheatmap(mat = data.norm,
         cluster_cols = hclustc,
         cluster_rows = hclustr,
         #clustering_method = "ward.D2",
         cutree_rows = n.row.clusters , 
         cutree_cols = n.col.clusters , 
         #annotation_row = auxdata,
         main = paste(plot_title, ': Iteration 0', sep = ''),
         ...))
    dev.off()
  }
  
  
  # recalculate row distances using DM
  
  rowdist.new = matrix(0, nrow(data.norm), nrow(data.norm))
  rowdist.list = list()
  rowdim.list = numeric(n.col.clusters)
  for(i in 1:n.col.clusters){
    #i = 1
    #print(i)
    inds = which(cutree(hclustc, n.col.clusters) == i)
    temp = data.norm[,inds]
    tempmat <- as.matrix(dist(temp))/sqrt(length(inds))
    tempmat[is.na(tempmat)] <- 0 # if any NA distances due to missing values, set distance to 0
    rowdist.new = rowdist.new + tempmat  #This is the calculation for updating row distances using the original column hierarchical clustering tree.  The denominator normalizes the weigths so large clusters don't have disproportionate influence on the new distance matrix.
    #print(sum(sum(is.na(rowdist.new))))
    rowdist.list[[i]] = as.matrix(dist(temp))
    rowdim.list[i] = length(inds)
  }

  hclustr <- hclust(as.dist(rowdist.new), method = hclust_method)
  clusters.row.out[[length(clusters.row.out) + 1]] <- cutree(hclustr, n.row.clusters)
  
  
  # Recalculate column distances
  
  coldist.new = matrix(0, ncol(data.norm), ncol(data.norm))
  #coldist.new = as.matrix(dist(t(data.norm)))
  for(i in 1:n.row.clusters){
    # i = 1
    inds = which(cutree(hclustr, n.row.clusters) == i)
    temp = data.norm[inds,]
    tempmat <- as.matrix(dist(t(temp)))/sqrt(length(inds))
    tempmat[is.na(tempmat)] <- 0
    coldist.new = coldist.new + tempmat
  }
  
  hclustc <- hclust(as.dist(coldist.new), method = hclust_method)
  clusters.col.out[[length(clusters.col.out)+1]] <- cutree(hclustc, n.col.clusters)
  
  
  if(verbose){
    jpeg(paste(filename,'_V1.jpeg'), width = imwidth, height = imheight, units = 'in', res = imres)
    print(pheatmap(mat = data.norm,
         cluster_cols = hclustc,
         cluster_rows = hclustr,
         #clustering_method = "ward.D2",
         cutree_rows = n.row.clusters , 
         cutree_cols = n.col.clusters , 
         #annotation_row = auxdata,
         main = paste(plot_title, ': Iteration 1', sep = ''),
         ...))
    dev.off()
  }

  # Loop through this process for a while until
  # cluster membership doesn't change, or until counter
  # reaches a prespecified number.
  ctr = 2
  while(ctr <= maxreps){
    
    #print(ctr)
    
    # recalculate row distances us DM
    
    rowdist.new = matrix(0, nrow(data.norm), nrow(data.norm))
    rowdist.list = list()
    rowdim.list = numeric(n.col.clusters)
    for(i in 1:n.col.clusters){
      #i = 1
      inds = which(cutree(hclustc, n.col.clusters) == i)
      temp = data.norm[,inds]
      tempmat <- as.matrix(dist(temp))/sqrt(length(inds))
      tempmat[is.na(tempmat)] <- 0
      rowdist.new = rowdist.new + tempmat  #This is the calculation for updating row distances using the original column hierarchical clustering tree.  The denominator normalizes the weigths so large clusters don't have disproportionate influence on the new distance matrix.
      rowdist.list[[i]] = as.matrix(dist(temp))
      rowdim.list[i] = length(inds)
    }
    
    hclustr <- hclust(as.dist(rowdist.new), method = hclust_method)
    clusters.row.out[[length(clusters.row.out) + 1]] <- cutree(hclustr, n.row.clusters)
   
    
    # Recalculate column distances
    
    coldist.new = matrix(0, ncol(data.norm), ncol(data.norm))
    #coldist.new = as.matrix(dist(t(data.norm)))
    for(i in 1:n.row.clusters){
      # i = 1
      inds = which(cutree(hclustr, n.row.clusters) == i)
      temp = data.norm[inds,]
      tempmat <- as.matrix(dist(t(temp)))/sqrt(length(inds)) 
      tempmat[is.na(tempmat)] <- 0
      coldist.new = coldist.new + tempmat
    }

    hclustc <- hclust(as.dist(coldist.new), method = hclust_method)
    clusters.col.out[[length(clusters.col.out) + 1]] <- cutree(hclustc, n.col.clusters)
    
    if(verbose){
      jpeg(paste(filename, '_V',ctr,'.jpeg', sep = '') , width = imwidth, height = imheight, units = 'in', res = imres)
      print(pheatmap(mat = data.norm,
                     cluster_cols = hclustc,
                     cluster_rows = hclustr,
                     #clustering_method = "ward.D2",
                     cutree_rows = n.row.clusters , 
                     cutree_cols = n.col.clusters , 
                     #annotation_row = auxdata,
                     main = paste(plot_title, ': Iteration ', ctr, sep = ''),
                     ...))
      dev.off()
    }
    
    if(
      sum((clusters.col.out[[length(clusters.col.out)]] - clusters.col.out[[length(clusters.col.out) - 1]])^2) == 0 &
      sum((clusters.row.out[[length(clusters.row.out)]] - clusters.row.out[[length(clusters.row.out) - 1]])^2) == 0
    ){ ctr = 1000000 } # bottom out if the row and column clusters don't change
    
    ctr = ctr + 1   
    
    
  }
  
  if(export.plot){
    jpeg(paste(filename,'_Final.jpeg'), width = imwidth, height = imheight, units = 'in', res = imres)
    print(pheatmap(mat = data.norm,
                   cluster_cols = hclustc,
                   cluster_rows = hclustr,
                   #clustering_method = "ward.D2",
                   cutree_rows = n.row.clusters , 
                   cutree_cols = n.col.clusters , 
                   #annotation_row = auxdata,
                   main = plot_title,
                   ...))
    dev.off()
  }
  
  if(display.plot){
   pheatmap(mat = data.norm,
                 cluster_cols = hclustc,
                 cluster_rows = hclustr,
                 #clustering_method = "ward.D2",
                 cutree_rows = n.row.clusters , 
                 cutree_cols = n.col.clusters , 
                 #annotation_row = auxdata,
                 main = plot_title,
                 ...)
  }
  
  
  
  return(list(colclust = clusters.col.out[[length(clusters.col.out)]],
              rowclust = clusters.row.out[[length(clusters.row.out)]],
              iteration_results = list(clusters_col = clusters.col.out, 
                                       clusters_row = clusters.row.out)))
  
  
}


data.norm <- milkquantdat_all2
colnames(data.norm) <- paste('Day', as.numeric(names(data.norm)) - min(as.numeric(names(data.norm))) + 1)

auxdata <- DMdat[DMdat$CowID %in% cowlist_healthy_all, c('group', 'AgeC','CalvingDateC','MilkYield95C')]
names(auxdata) <- c('Treatment', 'Age', 'CalvingDate', 'MilkYield')

temp <- ifelse(as.numeric(names(milkquantdat_all2)) > 17280, 'Pasture', 'Pen')
colauxdat <- data.frame(Period = as.factor(temp))
colauxdat$DaysOnTrial <- as.numeric(names(milkquantdat_all2)) - min(as.numeric(names(milkquantdat_all2))) + 1
rownames(colauxdat) <- names(data.norm)

if(isexporting){
  dmploy.out <- dmplot(data.norm, n.row.clusters = 3, n.col.clusters = 2, 
                     maxreps = 15, verbose = T, display.plot = F, 
                     imwidth = 12, imheight = 15,
                     annotation_row = auxdata, 
                     annotation_col = colauxdat,
                     annotation_colors = list(
                       CalvingDate = setNames(brewer.pal(nlevels(auxdata$CalvingDate), "Blues"),
                                              levels(auxdata$CalvingDate)),
                       Age = setNames(brewer.pal(nlevels(auxdata$Age), "Purples"),
                                              levels(auxdata$Age)),
                       MilkYield = setNames(brewer.pal(nlevels(auxdata$MilkYield), "Reds"),
                                              levels(auxdata$MilkYield)),
                       Treatment  = c('Control' = 'cornflowerblue', 'Organilac' = 'darkolivegreen1'),
                       Period = c('Pen' = 'cornflowerblue', 'Pasture' = 'darkolivegreen1'),
                       DaysOnTrial  = brewer.pal(colauxdat$DaysOnTrial, "Purples")
                       #DaysOnTrial  = setNames(brewer.pal(nlevels(colauxdat$DaysOnTrial), "Purples"),
                       #                       levels(colauxdat$DaysOnTrial))
                       ),
                     filename = 'Viz/DatMechViz/R3C2/DatMechPlotOut'
                     )
}
```

```
## Loading required package: pheatmap
```

```
## Warning: package 'pheatmap' was built under R version 3.5.2
```

```
## Loading required package: RColorBrewer
```

```
## Loading required package: dplyr
```

```
## 
## Attaching package: 'dplyr'
```

```
## The following object is masked from 'package:nlme':
## 
##     collapse
```

```
## The following objects are masked from 'package:plyr':
## 
##     arrange, count, desc, failwith, id, mutate, rename, summarise,
##     summarize
```

```
## The following objects are masked from 'package:stats':
## 
##     filter, lag
```

```
## The following objects are masked from 'package:base':
## 
##     intersect, setdiff, setequal, union
```

```
## Warning in if (n < 3) {: the condition has length > 1 and only the first
## element will be used
```

```
## Warning in brewer.pal(colauxdat$DaysOnTrial, "Purples"): minimal value for n is 3, returning requested palette with 3 different levels
```

```
if(isexporting){
  dmploy.out <- dmplot(data.norm, n.row.clusters = 4, n.col.clusters = 2, 
                     maxreps = 15, verbose = T, display.plot = F, 
                     imwidth = 12, imheight = 15,
                     annotation_row = auxdata, 
                     annotation_col = colauxdat,
                     annotation_colors = list(
                       CalvingDate = setNames(brewer.pal(nlevels(auxdata$CalvingDate), "Blues"),
                                              levels(auxdata$CalvingDate)),
                       Age = setNames(brewer.pal(nlevels(auxdata$Age), "Purples"),
                                              levels(auxdata$Age)),
                       MilkYield = setNames(brewer.pal(nlevels(auxdata$MilkYield), "Reds"),
                                              levels(auxdata$MilkYield)),
                       Treatment  = c('Control' = 'cornflowerblue', 'Organilac' = 'darkolivegreen1'),
                       Period = c('Pen' = 'cornflowerblue', 'Pasture' = 'darkolivegreen1'),
                       DaysOnTrial  = brewer.pal(colauxdat$DaysOnTrial, "Purples")
                       #DaysOnTrial  = setNames(brewer.pal(nlevels(colauxdat$DaysOnTrial), "Purples"),
                       #                       levels(colauxdat$DaysOnTrial))
                       ),
                     filename = 'Viz/DatMechViz/R4C2/DatMechPlotOut'
                     )
}
```

```
## Warning in if (n < 3) {: the condition has length > 1 and only the first element will be used

## Warning in if (n < 3) {: minimal value for n is 3, returning requested palette with 3 different levels
```

```
if(isexporting){
  dmploy.out <- dmplot(data.norm, n.row.clusters = 5, n.col.clusters = 3, 
                     maxreps = 15, verbose = T, display.plot = F, 
                     imwidth = 12, imheight = 15,
                     annotation_row = auxdata, 
                     annotation_col = colauxdat,
                     annotation_colors = list(
                       CalvingDate = setNames(brewer.pal(nlevels(auxdata$CalvingDate), "Blues"),
                                              levels(auxdata$CalvingDate)),
                       Age = setNames(brewer.pal(nlevels(auxdata$Age), "Purples"),
                                              levels(auxdata$Age)),
                       MilkYield = setNames(brewer.pal(nlevels(auxdata$MilkYield), "Reds"),
                                              levels(auxdata$MilkYield)),
                       Treatment  = c('Control' = 'cornflowerblue', 'Organilac' = 'darkolivegreen1'),
                       Period = c('Pen' = 'cornflowerblue', 'Pasture' = 'darkolivegreen1'),
                       DaysOnTrial  = brewer.pal(colauxdat$DaysOnTrial, "Purples")
                       #DaysOnTrial  = setNames(brewer.pal(nlevels(colauxdat$DaysOnTrial), "Purples"),
                       #                       levels(colauxdat$DaysOnTrial))
                       ),
                     filename = 'Viz/DatMechViz/R5C3/DatMechPlotOut'
                     )
}
```

```
## Warning in if (n < 3) {: the condition has length > 1 and only the first element will be used

## Warning in if (n < 3) {: minimal value for n is 3, returning requested palette with 3 different levels
```

```
dmploy.out <- dmplot(data.norm, n.row.clusters = 1, n.col.clusters = 8, 
                     maxreps = 15, 
                     verbose = F, display.plot = T, export.plot = F,
                     imwidth = 12, imheight = 15,
                     annotation_row = auxdata, 
                     annotation_col = colauxdat,
                     annotation_colors = list(
                       CalvingDate = setNames(brewer.pal(nlevels(auxdata$CalvingDate), "Blues"),
                                              levels(auxdata$CalvingDate)),
                       Age = setNames(brewer.pal(nlevels(auxdata$Age), "Purples"),
                                              levels(auxdata$Age)),
                       MilkYield = setNames(brewer.pal(nlevels(auxdata$MilkYield), "Reds"),
                                              levels(auxdata$MilkYield)),
                       Treatment  = c('Control' = 'cornflowerblue', 'Organilac' = 'darkolivegreen1'),
                       Period = c('Pen' = 'cornflowerblue', 'Pasture' = 'darkolivegreen1'),
                       DaysOnTrial  = brewer.pal(colauxdat$DaysOnTrial, "Purples")
                       #DaysOnTrial  = setNames(brewer.pal(nlevels(colauxdat$DaysOnTrial), "Purples"),
                       #                       levels(colauxdat$DaysOnTrial))
                       ),
                     filename = 'Viz/DatMechViz/R5C3/DatMechPlotOut'
                     )
```

```
## Warning in if (n < 3) {: the condition has length > 1 and only the first element will be used

## Warning in if (n < 3) {: minimal value for n is 3, returning requested palette with 3 different levels
```

And now I want to run this on a grid of cluster values

Alright, and now I want to write a function that will run DM on a grid of col and row cluster numbers

```
dmplot_grid <- function(data.norm, n.row.clusters = 1, n.col.clusters = 1, hclust_method = "ward.D2", maxreps = 10, verbose = FALSE, display.plot = FALSE, imwidth = 10, imheight = 15, imres = 300, plot_title = 'Data Mechanics Plot', filename = 'DatMechPlotOut', ...){
  
  
  cluster.results <- list()
  
  for(r in n.row.clusters){
    for(c in n.col.clusters){
      
      cout <- dmplot(data.norm, n.row.clusters = r, n.col.clusters = c, hclust_method = hclust_method, maxreps = maxreps, verbose = verbose, display.plot = display.plot, imwidth = imwidth , imheight = imheight, imres = imres , plot_title = paste(plot_title, r, 'Rows', c ,'Cols', sep = ' '), filename = paste(filename,'R', r, 'C', c, sep = ''), ...)

      
      cluster.results[[length(cluster.results) + 1]] <- list(nrow = r, ncol = c, cluster.out = cout)
      
    }
  }
  
  return(cluster.results)
  
}

if(isexporting){
  dmout <- dmplot_grid(data.norm, n.row.clusters = 1:10, n.col.clusters = 1:10, 
                     maxreps = 15, verbose = F,
                     imwidth = 14, imheight = 15,
                     #imres = 50,
                     annotation_row = auxdata, 
                     annotation_col = colauxdat,
                     annotation_colors = list(
                       CalvingDate = setNames(brewer.pal(nlevels(auxdata$CalvingDate), "Blues"),
                                              levels(auxdata$CalvingDate)),
                       Age = setNames(brewer.pal(nlevels(auxdata$Age), "Purples"),
                                              levels(auxdata$Age)),
                       MilkYield = setNames(brewer.pal(nlevels(auxdata$MilkYield), "Reds"),
                                              levels(auxdata$MilkYield)),
                       Treatment  = c('Control' = 'cornflowerblue', 'Organilac' = 'darkolivegreen1'),
                       Period = c('Pen' = 'cornflowerblue', 'Pasture' = 'darkolivegreen1'),
                       DaysOnTrial  = brewer.pal(colauxdat$DaysOnTrial, "Purples")
                       #DaysOnTrial  = setNames(brewer.pal(nlevels(colauxdat$DaysOnTrial), "Purples"),
                       #                       levels(colauxdat$DaysOnTrial))
                       ),
                     filename = 'Viz/DatMechViz/Grid/DatMechPlotOut'
                     )
}
```

```
## Warning in if (n < 3) {: the condition has length > 1 and only the first
## element will be used
```

```
## Warning in brewer.pal(colauxdat$DaysOnTrial, "Purples"): minimal value for n is 3, returning requested palette with 3 different levels
```

Some thoughts from these plots.

The next observation is that, looking at ACF of individual cows, there doesn’t seem to be a lot of temporal correlation, nor do you see any very clear shifts between the pen and pasture period. But DM provides a means to assess this across the entire herd, and show that there are clear differences between these two period, as they are not randomly assorting.

The first is that this is an extremely powerful way to asess outliers. Perhaps that is selling this technique short, but that is no mean issue to address on a problem of this scale. Looking at the individual cow plots, very few outliers are apparent. This approach allows use to identify days that are irrigular assessed against both the entire herd and against subgroups. That is incredibly useful for data scrubbing.

It is also succeeding in picking up outliers at the individual cow level that are so significant they are shifting assessment of the broader herd. See R2C3, which show a division in the pasture days that appears to be driven largely by a single cow, who normally leads the herd, but on this subset of days fell back near the rear. Such patterns are both behaviorally interesting, but would also be maddening to a linear regression model.

At R6 you find a subset of cows who clearly are not homogenous in entry pattern across the pen and pasture subsets, having moved up quite a ways in the pasture period.

Another interesting trend is that, as you get into the higher division of rows, the two highly consistent groups and front and back of the herd remain quite consistent in size and edentify. It is the middle groups that are getting broken up into smaller groups that show some correlation in their flucturations in entry position during subsets of the time period. R8C3 shows this quite nicely.

## Data Mechanics Visualization - All Cows

Now, I want to look at the dynamics of all the cows, those with and without recorded health challenges. I’ll look at all cows the attended at least 50% of milkings, which leave 177 animals

```
milkquantdat_sickhealthy <- milkquantdat[,names(milkquantdat) %in% names(milkquantdat_all2)]
rownames(milkquantdat_sickhealthy) <- milkquantdat$CowID

temp <- apply(milkquantdat_sickhealthy, 1, function(x) sum(!is.na(x))/ncol(milkquantdat_sickhealthy))
milkquantdat_sickhealthy <- milkquantdat_sickhealthy[temp>=0.5,]
dim(milkquantdat_sickhealthy)
```

```
## [1] 177  80
```

```
auxdata <- DMdat[DMdat$CowID %in% rownames(milkquantdat_sickhealthy), c("Sick", "Sick_BurnIn", "Sick_Pen", "Sick_Pasture", 'DIGNOSIS')]
auxdata <- DMdat[DMdat$CowID %in% rownames(milkquantdat_sickhealthy), c("Sick", "Sick_BurnIn", "Sick_Pen", "Sick_Pasture")]
auxdata$Sick <- ifelse(auxdata$Sick, 'Sick', 'Healthy')
auxdata$Sick_BurnIn <- ifelse(auxdata$Sick_BurnIn, 'Sick', 'Healthy')
auxdata$Sick_Pen <- ifelse(auxdata$Sick_Pen, 'Sick', 'Healthy')
auxdata$Sick_Pasture <- ifelse(auxdata$Sick_Pasture, 'Sick', 'Healthy')
#auxdata$DIGNOSIS[is.na(auxdata$DIGNOSIS)] <- 'Healthy'
names(auxdata) <- c('All', 'Enrollment', 'Pen', 'Pasture')
#names(auxdata) <- c('All', 'Enrollment', 'Pen', 'Pasture', 'Diagnosis')


data.norm <- milkquantdat_sickhealthy
names(data.norm) <- paste('Day', as.numeric(names(data.norm)) - min(as.numeric(names(data.norm)[-1])) + 1)

temp <- ifelse(as.numeric(names(milkquantdat_sickhealthy)) > 17280, 'Pasture',
               ifelse(as.numeric(names(milkquantdat_sickhealthy)) >=
                        min(as.numeric(names(milkquantdat_all)[-1])), 'Pen', 'Enrollment')) 
colauxdat <- data.frame(Period = as.factor(temp))
colauxdat$DaysOnTrial <- as.numeric(names(milkquantdat_sickhealthy)) - min(as.numeric(names(milkquantdat_all)[-1])) + 1
rownames(colauxdat) <- names(data.norm)

if(isexporting){
  dmout.sick <- dmplot_grid(data.norm, n.row.clusters = 1:10, n.col.clusters = 1:10, 
                     maxreps = 15, verbose = F, display.plot = F,
                     imwidth = 14, imheight = 22,
                     #imres = 43,
                     annotation_row = auxdata, 
                     annotation_col = colauxdat,
                     annotation_colors = list(
                       All = c('Healthy' = 'cornflowerblue', 'Sick' = 'coral2'),
                       Enrollment = c('Healthy' = 'cornflowerblue', 'Sick' = 'orange1'),
                       Pen = c('Healthy' = 'cornflowerblue', 'Sick' = 'plum2'),
                       Pasture = c('Healthy' = 'cornflowerblue', 'Sick' = 'darkolivegreen1'),
                       Period = c('Pen' = 'cornflowerblue', 'Pasture' = 'darkolivegreen1'),             
                       DaysOnTrial  = brewer.pal(colauxdat$DaysOnTrial, "Purples")
                       #DaysOnTrial  = setNames(brewer.pal(nlevels(colauxdat$DaysOnTrial), "Purples"),
                       #                       levels(colauxdat$DaysOnTrial))
                       ),
                     filename = 'Viz/DatMechViz/Grid_LR/DatMechPlotOut'
                     #filename = 'Viz/DatMechViz/Grid_Sicks/DatMechPlotOut'
                     )
}
```

```
## Warning in if (n < 3) {: the condition has length > 1 and only the first
## element will be used
```

```
## Warning in brewer.pal(colauxdat$DaysOnTrial, "Purples"): minimal value for n is 3, returning requested palette with 3 different levels
```

```
dmploy.out <- dmplot(data.norm, n.row.clusters = 9, n.col.clusters = 2, 
                     maxreps = 15, 
                     verbose = F, display.plot = T, export.plot = F,
                     imwidth = 14, imheight = 22,
                     annotation_row = auxdata, 
                     annotation_col = colauxdat,
                     annotation_colors = list(
                       All = c('Healthy' = 'cornflowerblue', 'Sick' = 'coral2'),
                       Enrollment = c('Healthy' = 'cornflowerblue', 'Sick' = 'orange1'),
                       Pen = c('Healthy' = 'cornflowerblue', 'Sick' = 'plum2'),
                       Pasture = c('Healthy' = 'cornflowerblue', 'Sick' = 'darkolivegreen1'),
                       Period = c('Pen' = 'cornflowerblue', 'Pasture' = 'darkolivegreen1'),             
                       DaysOnTrial  = brewer.pal(colauxdat$DaysOnTrial, "Purples")
                       #DaysOnTrial  = setNames(brewer.pal(nlevels(colauxdat$DaysOnTrial), "Purples"),
                       #                       levels(colauxdat$DaysOnTrial))
                       ),
                     
                     filename = 'Viz/DatMechViz/Grid_Sicks/DatMechPlotOut'
                     
                     )
```

```
## Warning in if (n < 3) {: the condition has length > 1 and only the first element will be used

## Warning in if (n < 3) {: minimal value for n is 3, returning requested palette with 3 different levels
```

Good resolution at:

R6C2 R7C3 R9C2 - perfect seperation of periods

I see pretty much the same patterns as before. What I don’t see is any clear differentiations in sick status. Maybe the cows consistently in the very front have a slightly lower rate of ilness, but if its there it’s only subtly so.

# Linear Analysis - Cow Attributes

## Defining Functions - Mutual Conditional Entropy Test

First, I’m going to write a function to calculate the multual entropy of a contingency table. I’ll default to base 2 for the log (shannon’s entropy) and I’ll weight the row and column entorpy sums by size of each cluster (so tiny clusters don’t unduly influence the calculation).

```
#mat <- table(clust.out.queue$Pen6, clust.out.queue$Pasture4) # contingency table k = 2

mutcondEntropy <- function(mat, logbase = 2){
  
  rowsum <- apply(mat, 1, sum)
  colsum <- apply(mat, 2, sum)
  
  # calculate row entropy 
  
  E_row = 0
  for(i in 1:nrow(mat)){
    p = mat[i,]/rowsum[i] + 10e-20  # I'm adding a tiny number to avoid getting -Inf from the log
    E_row <- E_row + (rowsum[i]/sum(rowsum))* (-sum(p*log(p, base = logbase))) 
  }
  
  
  # calcultate column entropy
  
  E_col = 0
  for(j in 1:ncol(mat)){
    p = mat[,j]/colsum[j] + 10e-20  # I'm adding a tiny number to avoid getting -Inf from the log
    E_col <- E_col + (colsum[j]/sum(colsum))*(-sum(p*log(p, base = logbase))) 
  }
  
  return(list(MutualEntropy = (E_row + E_col)/2, RowEntropy = E_row, ColumnEntropy = E_col))
  
  
}


# Check calculation

temp <- matrix(0, nrow = 4, ncol = 4)
temp[1,1] <- 1
temp[1,2] <- 1
temp[2,3] <- 1
temp[2,4] <- 1
temp[3,1] <- 1
temp[3,3] <- 1
temp[4,2] <- 1
temp[4,4] <- 1
temp
```

```
##      [,1] [,2] [,3] [,4]
## [1,]    1    1    0    0
## [2,]    0    0    1    1
## [3,]    1    0    1    0
## [4,]    0    1    0    1
```

```
mutcondEntropy(temp)
```

```
## $MutualEntropy
## [1] 1
## 
## $RowEntropy
## [1] 1
## 
## $ColumnEntropy
## [1] 1
```

Alright, now that we’ve got a summary statistic, I want to create a permutation test to assess if the categorizaitons generated in the observed data have a lower mutual conditional entropy (ie - share more information) that would be expected by randomly assigning cows to observed data vectors. By simply reassigning cows to data vectors, the marginal distributions will not be changes, only the bivariate associations will be eliminated by the randomization. I’ll randomize both the row and the column clusters to be thorough.

```
MCEtest <- function(x, y, plotresults = F, B = 2000, logbase = 2){
  #x = data vector of row categorical variable
  #y = dat vector of column categorical variable
  #NOTE: x and y should by aligned by observed individuals
  
  entobs <- mutcondEntropy(table(x,y), logbase = logbase)$MutualEntropy
  
  randent <- rep(NA, B)
  for(i in 1:B){
    xr <- sample(x, replace = F)
    yr <- sample(y, replace = F)
    randent[i] <- mutcondEntropy(table(xr, yr), logbase = logbase)$MutualEntropy
  }
  
  if(plotresults){
    hist(randent, 
         xlim = c( min(c(randent,entobs)), max(c(randent,entobs))),
         main = 'Histogram of Randomized MCE Values')
    abline(v=entobs, col = 'red')
  }
  
  return(list(ObservedMCE = entobs, PVal = sum(randent < entobs)/B))
  
}

#MCEtest(clust.out.queue$Pasture6, clust.out.queue$Pen6, plotresults = T)
```

Righto, now the final challenge with implementing this code is that different features in the data set may come into resolution at different levels of categorical resolution. I can use the plots of the hclust trees to get a rough idea of where strucutre ends and the stochastic weeds begin, but I don’t know where in between the bivariate relationships may come into rolution. So I want to set up functionality to test for significant MCE on a grid of the two variables under consideration.

```
# xtree <- h.dist.pen
# xrange <- 2:5
# ytree <- h.dist.pasture
# yrange <- 2:5

bivartree <- function(xtree, ytree, xrange = c(2), yrange=c(2), logbase = 2, nperm = 2000){
  #xtree = hclust object for the row variable
  #ytree = hclustobject for the column variable
  #NOTE: labels for the two tree need to match
  
  try(if(1 %in% xrange | 1 %in% yrange ) stop("Error: Cluster size must be >1"))
  
  p_out <- matrix(NA, nrow = length(xrange) , ncol = length(yrange) )
  rownames(p_out) <- paste('k=',xrange, sep='')
  colnames(p_out) <- paste('k=',yrange, sep='')
  
  contabout <- list()
  
  for(i in 1:length(xrange)){
    rowout <- list()
    for(j in 1:length(yrange)){
      
      cx <- data.frame(ObsID = names(cutree(xtree, xrange[i])),  cx =  cutree(xtree, xrange[i]))
      cy <- data.frame(ObsID = names(cutree(ytree, yrange[j])),  cy =  cutree(ytree, yrange[j]))
      temp <- merge(cx,cy, by = 'ObsID')
      
      temptest <- MCEtest(temp$cx, temp$cy , B = nperm, logbase = logbase)

      
      rowout[[yrange[j]]] <- list(ContingencyTable = table(temp$cx, temp$cy),
                                  ObserveMCE = temptest$ObservedMCE,
                                  PVal = temptest$PVal)
      p_out[i,j] <- temptest$PVal
      
    }
    contabout[[xrange[i]]] <- rowout
  }
  
  return(list(SignificanceMatrix = p_out, Results = contabout))
  
}

# clustest_queue_penpast <- bivartree(h.dist.pen, h.dist.pasture, xrange = 2:10, yrange = 2:10)
# clustest_queue_penpast$SignificanceMatrix
# 
# clustest_queue_penpast$Results[[3]][[3]]
```

Finally, I want a function to pull out the optimal p-value from the grid result. I’m going to do this by first ranking p-values across each row and column, then marginally finding the lowest average rank in order to identify the optimal row and column values for cluster sizes.

```
gridout <- as.data.frame(matrix(c(0.1,0.03,0.2,0.002,0.4,0.6),2,3))
getBVTPval <- function(gridout){
  
  rankinrow <- t(apply(gridout, 1, rank))
  avgcolrank <- apply(rankinrow, 2, mean)
  mincol <- which.min(avgcolrank)
  
  rankincol <- apply(gridout, 2, rank)
  avgrowrank <- apply(rankincol, 1, mean)
  minrow <- which.min(avgrowrank)
  
  return(list(pval = gridout[minrow, mincol], row = minrow, col = mincol))
  
}

#getBVTPval(gridout)
```

Righto, game on :D

## Healthy Cows

### Data Wrangling

First, I need to get this data back in long format (with the correct cow attributes attached).

```
dat.linmod <- data.frame(CowID = NA, 
                         Age = NA,
                         CalvingDate = NA, 
                         MilkYield = NA,
                         Treatment = NA,
                         Date = NA,
                         EntryQuantile = NA)

ntemp <- ncol(milkquantdat_all2)
for(i in 1:nrow(milkquantdat_all2)){

  cowtemp <- as.numeric(rownames(milkquantdat_all2)[i])
  rtemp <- which(cowattribdat$CowID == cowtemp)
  
  if(!length(rtemp) > 0){
    print(cowtemp)
    next
  }
  
  dattemp <- data.frame(CowID = rep(cowtemp, ntemp), 
                         Age = rep(cowattribdat$AgeDaysOld[rtemp], ntemp),
                         CalvingDate = rep(cowattribdat$CalvingDateN[rtemp], ntemp), 
                         MilkYield = rep(cowattribdat$MilkYield95[rtemp], ntemp),
                         Treatment = rep(cowattribdat$group[rtemp], ntemp),
                         Date = names(milkquantdat_all2),
                         EntryQuantile = unlist(milkquantdat_all2[i, ]) 
                        )
  
  dat.linmod <- rbind(dat.linmod, dattemp)
  
}

dat.linmod <- dat.linmod[-1, ]
dat.linmod <- dat.linmod[complete.cases(dat.linmod),]
length(unique(dat.linmod$CowID)) == nrow(milkquantdat_all2)
```

```
## [1] FALSE
```

```
dat.linmod$Agec <- scale(dat.linmod$Age)
dat.linmod$CalvingDatec <- scale(dat.linmod$CalvingDate)
dat.linmod$MilkYieldc <- scale(dat.linmod$MilkYield)
```

Now I need to remove the outlier days that I identified in the data mechanics code

```
dat.linmod.uml <- dat.linmod[!dat.linmod$Date == '17325',]
dat.linmod.uml <- dat.linmod.uml[!dat.linmod.uml$Date == '17329',]
dat.linmod.uml <- dat.linmod.uml[!dat.linmod.uml$Date == '17330',]
dat.linmod.uml <- dat.linmod.uml[!dat.linmod.uml$Date == '17331',]
```

### Linear Models

Model with homogenous variance

```
lme.out.culled <- lme(EntryQuantile ~  Agec + CalvingDatec + MilkYieldc  + Treatment + Agec*MilkYieldc + Agec*CalvingDatec + MilkYieldc*CalvingDatec, 
                      random = ~ 1 | CowID, 
                      data = dat.linmod.uml , 
                      method = 'ML') 

summary(lme.out.culled )
```

```
## Linear mixed-effects model fit by maximum likelihood
##  Data: dat.linmod.uml 
##         AIC       BIC   logLik
##   -4774.206 -4704.909 2397.103
## 
## Random effects:
##  Formula: ~1 | CowID
##         (Intercept)  Residual
## StdDev:   0.2241254 0.1703968
## 
## Fixed effects: EntryQuantile ~ Agec + CalvingDatec + MilkYieldc + Treatment +      Agec * MilkYieldc + Agec * CalvingDatec + MilkYieldc * CalvingDatec 
##                              Value  Std.Error   DF   t-value p-value
## (Intercept)              0.4258750 0.03964558 7449 10.742055  0.0000
## Agec                    -0.1189473 0.03478939   96 -3.419068  0.0009
## CalvingDatec             0.0084785 0.02312847   96  0.366582  0.7147
## MilkYieldc               0.1420707 0.03588897   96  3.958616  0.0001
## TreatmentOrganilac       0.0145924 0.04528792   96  0.322214  0.7480
## Agec:MilkYieldc          0.0729462 0.03095511   96  2.356516  0.0205
## Agec:CalvingDatec       -0.0276077 0.02883152   96 -0.957552  0.3407
## CalvingDatec:MilkYieldc  0.0365557 0.03561271   96  1.026478  0.3072
##  Correlation: 
##                         (Intr) Agec   ClvngD MlkYld TrtmnO Agc:MY Agc:CD
## Agec                     0.302                                          
## CalvingDatec            -0.002 -0.187                                   
## MilkYieldc              -0.314 -0.729  0.045                            
## TreatmentOrganilac      -0.638 -0.177 -0.006  0.077                     
## Agec:MilkYieldc         -0.541 -0.319  0.032  0.475  0.046              
## Agec:CalvingDatec       -0.129 -0.133 -0.182 -0.007  0.116 -0.075       
## CalvingDatec:MilkYieldc  0.113  0.056  0.097  0.045 -0.098 -0.114 -0.686
## 
## Standardized Within-Group Residuals:
##         Min          Q1         Med          Q3         Max 
## -4.62763146 -0.56816265 -0.05522952  0.58632865  5.24932858 
## 
## Number of Observations: 7553
## Number of Groups: 104
```

```
anova(lme.out.culled)
```

```
##                         numDF denDF  F-value p-value
## (Intercept)                 1  7449 479.2068  <.0001
## Agec                        1    96   1.0433  0.3096
## CalvingDatec                1    96   0.0016  0.9686
## MilkYieldc                  1    96   9.5755  0.0026
## Treatment                   1    96   0.1052  0.7463
## Agec:MilkYieldc             1    96   6.1107  0.0152
## Agec:CalvingDatec           1    96   0.1215  0.7281
## CalvingDatec:MilkYieldc     1    96   1.0537  0.3072
```

```
qqnorm(as.vector(lme.out.culled$residuals))
```

Model with heterogenous variance

```
ctrl <- lmeControl(opt='optim')
lme.out.uml <- lme(EntryQuantile ~  Agec + CalvingDatec + MilkYieldc  + Treatment + Agec*MilkYieldc + Agec*CalvingDatec + MilkYieldc*CalvingDatec , 
                   random = ~ 1 | CowID, 
                   weights = varIdent(form = ~ 1 | CowID),
                   data = dat.linmod.uml,
                   control=ctrl,
                   method = 'ML') 

summary(lme.out.uml)
```

```
## Linear mixed-effects model fit by maximum likelihood
##  Data: dat.linmod.uml 
##        AIC       BIC  logLik
##   -6246.92 -5463.864 3236.46
## 
## Random effects:
##  Formula: ~1 | CowID
##         (Intercept)  Residual
## StdDev:   0.2248149 0.1631351
## 
## Variance function:
##  Structure: Different standard deviations per stratum
##  Formula: ~1 | CowID 
##  Parameter estimates:
##       443       645      1090      1135      1371      1424      1454 
## 1.0000000 1.5147906 0.5776144 0.9280497 1.2358281 1.2650996 0.5987701 
##      1511      1951      2053      2219      2457      2558      4562 
## 1.2871306 1.4178596 0.3022210 1.3664740 0.8699835 1.3285760 0.6099606 
##      6635      7057      7941      9048      9130      9323      9363 
## 1.1945156 0.8459359 1.0223492 1.1089129 0.5328361 0.5841644 1.0688751 
##     10318     10837     10985     11690     13077     13267     13407 
## 1.5676595 1.7893385 1.1241017 1.2378288 0.6121660 0.7034666 1.1076821 
##     13429     13460     13467     13482     13496     13812     13814 
## 1.2739111 0.9208607 1.4643130 1.1026731 0.6112383 0.7615542 0.5854280 
##     13836     13933     13936     13946     13952     13956     16229 
## 0.4398051 1.1438268 1.1676035 0.9853307 1.1477824 1.1372004 0.3334067 
##     16829     17001     18649     18781     18802     18828     19185 
## 0.1382038 1.0602575 1.0878593 1.2958707 1.0871238 1.4648131 1.0437754 
##     19403     19484     20216     20311     21292     21906     22179 
## 0.7579903 1.0915691 1.2955554 0.9036581 0.9959510 1.0666129 0.8585925 
##     23049     23325     23428     25846     26067     26228     26451 
## 0.9899105 1.3622285 0.9246320 0.9763509 0.6287841 1.1113646 0.5857795 
##     26853     26905     26934     27050     27335     27340     27423 
## 0.6630528 1.1166903 1.0161899 1.1763643 1.0306149 0.7237317 0.8049281 
##     31867     32607     32949     33199     33513     33908     34350 
## 0.3722835 0.6095203 0.7685845 1.3609491 1.1855729 0.8313736 1.3732705 
##     34360     34368     34369     45551     45724     46769     46926 
## 0.8743339 0.9189423 1.1876100 0.9908305 1.5674864 0.7652973 1.0530197 
##     48576     50307     55454     55482     55516     55524     55562 
## 1.2312880 1.0261865 0.3463751 0.7350738 1.3226279 1.2668122 1.2968052 
##     55773     55785     55832     62002     63530     63811     65469 
## 1.2763870 0.9866831 1.1176121 0.5010154 0.5156575 1.1407543 0.8514937 
##     96023     97907     97933     98739     98771     98777 
## 1.1088205 1.1476491 1.1450689 1.0573517 1.2390903 1.0708199 
## Fixed effects: EntryQuantile ~ Agec + CalvingDatec + MilkYieldc + Treatment +      Agec * MilkYieldc + Agec * CalvingDatec + MilkYieldc * CalvingDatec 
##                              Value  Std.Error   DF   t-value p-value
## (Intercept)              0.4256233 0.03975239 7449 10.706861  0.0000
## Agec                    -0.1190762 0.03485318   96 -3.416508  0.0009
## CalvingDatec             0.0083584 0.02320098   96  0.360261  0.7194
## MilkYieldc               0.1423976 0.03597686   96  3.958032  0.0001
## TreatmentOrganilac       0.0146765 0.04541948   96  0.323133  0.7473
## Agec:MilkYieldc          0.0730195 0.03103534   96  2.352786  0.0207
## Agec:CalvingDatec       -0.0276968 0.02888393   96 -0.958901  0.3400
## CalvingDatec:MilkYieldc  0.0368135 0.03571627   96  1.030722  0.3053
##  Correlation: 
##                         (Intr) Agec   ClvngD MlkYld TrtmnO Agc:MY Agc:CD
## Agec                     0.301                                          
## CalvingDatec            -0.001 -0.186                                   
## MilkYieldc              -0.313 -0.728  0.045                            
## TreatmentOrganilac      -0.638 -0.177 -0.005  0.077                     
## Agec:MilkYieldc         -0.541 -0.318  0.032  0.475  0.046              
## Agec:CalvingDatec       -0.129 -0.132 -0.184 -0.007  0.116 -0.075       
## CalvingDatec:MilkYieldc  0.113  0.055  0.097  0.045 -0.097 -0.114 -0.685
## 
## Standardized Within-Group Residuals:
##         Min          Q1         Med          Q3         Max 
## -4.69072698 -0.62743304 -0.06605475  0.66473878  7.28512941 
## 
## Number of Observations: 7553
## Number of Groups: 104
```

```
anova(lme.out.uml)
```

```
##                         numDF denDF  F-value p-value
## (Intercept)                 1  7449 475.9494  <.0001
## Agec                        1    96   1.0411  0.3101
## CalvingDatec                1    96   0.0011  0.9737
## MilkYieldc                  1    96   9.5782  0.0026
## Treatment                   1    96   0.1059  0.7456
## Agec:MilkYieldc             1    96   6.0984  0.0153
## Agec:CalvingDatec           1    96   0.1202  0.7296
## CalvingDatec:MilkYieldc     1    96   1.0624  0.3053
```

```
qqnorm(as.vector(lme.out.uml$residuals))
```

Test significance of variance model

```
anova(lme.out.culled, lme.out.uml)
```

```
##                Model  df       AIC       BIC   logLik   Test  L.Ratio
## lme.out.culled     1  10 -4774.206 -4704.909 2397.103                
## lme.out.uml        2 113 -6246.920 -5463.864 3236.460 1 vs 2 1678.714
##                p-value
## lme.out.culled        
## lme.out.uml     <.0001
```

### Visualize Trend

```
temp <- unique(data.frame(CowID = dat.linmod.uml$CowID, Age = dat.linmod.uml$Age))
temp2 <- unique(data.frame(CowID = names(predict(lme.out.uml)), Predicted = predict(lme.out.uml)))
predict.uml.age <- merge(temp, temp2, by = 'CowID')
dim(predict.uml.age)
```

```
## [1] 104   3
```

```
temp <- unique(data.frame(CowID = dat.linmod.uml$CowID, MilkYield = dat.linmod.uml$MilkYield))
predict.uml.ageyield <- merge(predict.uml.age, temp, by = 'CowID')

temp <- c(1, coef(lme.out.uml$modelStruct$varStruct, unconstrained=F))
names(temp)[1] <- names(predict(lme.out.uml))[1]
temp <- (temp *lme.out.uml$sigma)^2
temp <- temp/min(temp)
temp2 <- data.frame(CowID = names(temp), Variance = temp)
predict.uml.ageyield <- merge(predict.uml.ageyield, temp2, by = 'CowID')

ggplot(predict.uml.ageyield, aes(x=Age, y=Predicted, colour = MilkYield, size = 1/Variance)) +
          geom_point() + 
          ylim(0,1) +
          ggtitle('Association Between Queue Position and Cow Age: Healthy Cows') + 
          xlab('Age in Days') +
          ylab('Predicted Queue Position') + 
          scale_color_viridis()
```

```
if(isexporting){
  jpeg('Viz/QueueVsAge_Healthy.jpg', width = 8, height = 5, units = 'in', res = 300)
  ggplot(predict.uml.ageyield, aes(x=Age, y=Predicted, colour = MilkYield, size = 1/Variance)) +
    geom_point() + 
    ylim(0,1) +
    ggtitle('Association Between Queue Position and Cow Age: Healthy Cows') + 
    xlab('Age in Days') +
    ylab('Predicted Queue Position') + 
    scale_color_viridis() 
  dev.off()
}
```

```
## quartz_off_screen 
##                 2
```

```
ggplot(predict.uml.ageyield, aes(x=MilkYield, y=Predicted, colour = Age, size = 1/Variance)) +
          geom_point() + 
          ylim(0,1) +
          ggtitle('Association Between Queue Position and Milk Yield: Healthy Cows') + 
          xlab('Peak Milk Yield') +
          ylab('Predicted Queue Position') + 
          scale_color_viridis()
```

```
if(isexporting){
  jpeg('Viz/QueueVsYield_Healthy.jpg', width = 8, height = 5, units = 'in', res = 300)
  ggplot(predict.uml.ageyield, aes(x=MilkYield, y=Predicted, colour = Age, size = 1/Variance)) +
    geom_point() + 
    ylim(0,1) +
    ggtitle('Association Between Queue Position and Milk Yield: Healthy Cows') + 
    xlab('Peak Milk Yield') +
    ylab('Predicted Queue Position') + 
    scale_color_viridis() 
  #labs(fill = "Milking Yield") 
  #geom_vline(xintercept = 17280, colour='grey')
  dev.off()
}
```

```
## quartz_off_screen 
##                 2
```

```
# plot(dat.linmod.uml$Age, dat.linmod.uml$EntryQuantile, col = as.factor(dat.linmod.uml$CowID), type = 'p', pch = 16)
# plot(dat.linmod.uml$MilkYield, dat.linmod.uml$EntryQuantile,  col = as.factor(dat.linmod.uml$CowID), type = 'p', pch = 16) 
# 
# tempx <- tapply(dat.linmod.uml$Age, dat.linmod.uml$CowID, function(x) mean(x, na.rm = T))
# tempy<- tapply(dat.linmod.uml$EntryQuantile, dat.linmod.uml$CowID, function(x) mean(x, na.rm = T))
# 
# plot(tempx, tempy, xlab = 'Age', ylab = 'Average Entry Quantile', pch = 16)
# 
# tempx <- tapply(dat.linmod.uml$MilkYield, dat.linmod.uml$CowID, function(x) mean(x, na.rm = T))
# plot(tempx, tempy, xlab = 'Milk Yield', ylab = 'Average Entry Quantile', pch = 16)

cor(predict.uml.ageyield$Age, predict.uml.ageyield$MilkYield)
```

```
## [1] 0.6983928
```

### Mutual Conditional Entropy Test

```
set.seed(1616)

# initialize hclust objects

milkquantdat_all2_dropdays <- milkquantdat_all2[rownames(milkquantdat_all2) %in% predict.uml.ageyield$CowID,] 
milkquantdat_all2_dropdays <- milkquantdat_all2_dropdays[ , !names(milkquantdat_all2_dropdays) %in% c('17325', '17329', '17330', '17331')]
dim(milkquantdat_all2_dropdays)
```

```
## [1] 104  76
```

```
d <- dist(milkquantdat_all2_dropdays)
h.all.reg <- hclust(d, method ='ward.D2') 
plot(h.all.reg)
```

```
temp <- predict.uml.ageyield$Age
names(temp) <- predict.uml.ageyield$CowID
d <- dist(temp)
h.age.reg <- hclust(d, method ='ward.D2')
plot(h.age.reg)
```

```
temp <- predict.uml.ageyield$MilkYield
names(temp) <- predict.uml.ageyield$CowID
d <- dist(temp)
h.yield.reg <- hclust(d, method ='ward.D2') 
plot(h.yield.reg)
```

```
# Association With Age
print('Age')
```

```
## [1] "Age"
```

```
btreeout_h_queue_age <- bivartree(h.all.reg, h.age.reg, xrange = 2:7, yrange = 2:6)
btreeout_h_queue_age$SignificanceMatrix
```

```
##        k=2   k=3    k=4    k=5    k=6
## k=2 0.1030 0.129 0.2400 0.2190 0.2980
## k=3 0.3240 0.350 0.6010 0.5065 0.6760
## k=4 0.4825 0.486 0.7345 0.4220 0.6040
## k=5 0.6270 0.701 0.9120 0.6225 0.6440
## k=6 0.6635 0.803 0.9335 0.7640 0.8045
## k=7 0.7985 0.815 0.9480 0.8600 0.6815
```

```
pout_h_queue_age <- getBVTPval(btreeout_h_queue_age$SignificanceMatrix)
pout_h_queue_age
```

```
## $pval
## [1] 0.103
## 
## $row
## k=2 
##   1 
## 
## $col
## k=2 
##   1
```

```
temp <- cutree(h.all.reg, 2)
temp2 <- data.frame(CowID = names(temp), Queue2 = temp)
predict.uml.ageyield <- merge(predict.uml.ageyield, temp2, by = 'CowID')

temp <- cutree(h.age.reg, 2)
temp2 <- data.frame(CowID = names(temp), Age2 = temp)
predict.uml.ageyield <- merge(predict.uml.ageyield, temp2, by = 'CowID')

tempage <- tapply(predict.uml.ageyield$Age, predict.uml.ageyield$Age2, median)
tempqueue <- tapply(predict.uml.ageyield$Predicted, predict.uml.ageyield$Queue2, median)

discreg_age_h <- data.frame(Queue = NA, Age = NA, Size = NA)
for(i in 1:length(tempage)){
  for(j in 1:length(tempqueue)){
    
    tempsum <- sum(predict.uml.ageyield$Age2 ==i & predict.uml.ageyield$Queue2 == j)
    if(!tempsum == 0){
      temp <- data.frame(Queue = tempqueue[j], Age = tempage[i], Size = tempsum)
      discreg_age_h <- rbind(discreg_age_h, temp)
    }
    
  }
}

ggplot(discreg_age_h, aes(y=Queue, x=Age, size = Size)) +
          geom_point() + 
          ylim(0,1) +
          ggtitle('Association Between Queue Position and Cow Age: Healthy Cows') + 
          xlab('Age in Days Cluster Median') +
          ylab('Predicted Queue Position Cluster Median') + 
          scale_color_viridis()
```

```
## Warning: Removed 1 rows containing missing values (geom_point).
```

```
if(isexporting){
  jpeg('Viz/MCE_Age_Healthy.jpg', width = 8, height = 5, units = 'in', res = 300)
  ggplot(discreg_age_h, aes(y=Queue, x=Age, size = Size)) +
    geom_point() + 
    ylim(0,1) +
    ggtitle('Association Between Queue Position and Cow Age: Healthy Cows') + 
    xlab('Age in Days Cluster Median') +
    ylab('Predicted Queue Position Cluster Median') + 
    scale_color_viridis()
  dev.off()
}
```

```
## quartz_off_screen 
##                 2
```

```
# Association With Yield
print('Yield')
```

```
## [1] "Yield"
```

```
btreeout_h_queue_yield <- bivartree(h.all.reg, h.yield.reg, xrange = 2:7, yrange = 2:7)
btreeout_h_queue_yield$SignificanceMatrix
```

```
##        k=2    k=3    k=4    k=5    k=6    k=7
## k=2 0.1790 0.0220 0.0465 0.0905 0.1335 0.1880
## k=3 0.3910 0.0455 0.0320 0.0360 0.0690 0.1445
## k=4 0.6125 0.1025 0.0460 0.0225 0.0785 0.1455
## k=5 0.7690 0.1230 0.0805 0.0445 0.0615 0.1400
## k=6 0.8690 0.1995 0.1915 0.1410 0.2050 0.2320
## k=7 0.9210 0.3795 0.3140 0.2320 0.2130 0.2610
```

```
pout_h_queue_yield <- getBVTPval(btreeout_h_queue_yield$SignificanceMatrix)
pout_h_queue_yield
```

```
## $pval
## [1] 0.036
## 
## $row
## k=3 
##   2 
## 
## $col
## k=5 
##   4
```

```
temp <- cutree(h.all.reg, 3)
temp2 <- data.frame(CowID = names(temp), Queue3 = temp)
predict.uml.ageyield <- merge(predict.uml.ageyield, temp2, by = 'CowID')

temp <- cutree(h.yield.reg, 5)
temp2 <- data.frame(CowID = names(temp), Yield5 = temp)
predict.uml.ageyield <- merge(predict.uml.ageyield, temp2, by = 'CowID')

tempyield <- tapply(predict.uml.ageyield$MilkYield, predict.uml.ageyield$Yield5, median)
tempqueue <- tapply(predict.uml.ageyield$Predicted, predict.uml.ageyield$Queue3, median)

discreg_yield_h <- data.frame(Queue = NA, MilkYield = NA, Size = NA)
for(i in 1:length(tempyield)){
  for(j in 1:length(tempqueue)){
    
    tempsum <- sum(predict.uml.ageyield$Yield5 ==i & predict.uml.ageyield$Queue3 == j)
    if(!tempsum == 0){
      temp <- data.frame(Queue = tempqueue[j], MilkYield = tempyield[i], Size = tempsum)
      discreg_yield_h <- rbind(discreg_yield_h, temp)
    }
    
  }
}

ggplot(discreg_yield_h, aes(y=Queue, x=MilkYield, size = Size)) +
          geom_point() + 
          ylim(0,1) +
          ggtitle('Association Between Queue Position and Peak Yield: Healthy Cows') + 
          xlab('Peak Milk Yield Cluster Median') +
          ylab('Predicted Queue Position Cluster Median') + 
          scale_color_viridis()
```

```
## Warning: Removed 1 rows containing missing values (geom_point).
```

```
if(isexporting){
  jpeg('Viz/MCE_Yield_Healthy.jpg', width = 8, height = 5, units = 'in', res = 300)
  ggplot(discreg_yield_h, aes(y=Queue, x=MilkYield, size = Size)) +
    geom_point() + 
    ylim(0,1) +
    ggtitle('Association Between Queue Position and Peak Yield: Healthy Cows') + 
    xlab('Peak Milk Yield Cluster Median') +
    ylab('Predicted Queue Position Cluster Median') + 
    scale_color_viridis()
  dev.off()
}
```

```
## quartz_off_screen 
##                 2
```

## Full Dataset

### Data Wrangling

First lets get this in long format and cull the outlier days

```
dat.linmod.sh <- data.frame(CowID = NA, 
                         Age = NA,
                         CalvingDate = NA, 
                         MilkYield = NA,
                         Treatment = NA,
                         Date = NA,
                         EntryQuantile = NA)

ntemp <- ncol(milkquantdat_sickhealthy)
for(i in 1:nrow(milkquantdat_sickhealthy)){

  cowtemp <- as.numeric(rownames(milkquantdat_sickhealthy)[i])
  rtemp <- which(cowattribdat$CowID == cowtemp)
  
  if(!length(rtemp) > 0){
    print(cowtemp)
    next
  }
  
  dattemp <- data.frame(CowID = rep(cowtemp, ntemp), 
                         Age = rep(cowattribdat$AgeDaysOld[rtemp], ntemp),
                         CalvingDate = rep(cowattribdat$CalvingDateN[rtemp], ntemp), 
                         MilkYield = rep(cowattribdat$MilkYield95[rtemp], ntemp),
                         Treatment = rep(cowattribdat$group[rtemp], ntemp),
                         Date = names(milkquantdat_sickhealthy),
                         EntryQuantile = unlist(milkquantdat_sickhealthy[i, ]) 
                        )
  
  dat.linmod.sh <- rbind(dat.linmod.sh, dattemp)
  
}

dat.linmod.sh <- dat.linmod.sh[complete.cases(dat.linmod.sh),]
length(unique(dat.linmod.sh$CowID))
```

```
## [1] 160
```

```
nrow(milkquantdat_sickhealthy)
```

```
## [1] 177
```

```
dat.linmod.sh <- dat.linmod.sh[!dat.linmod.sh$Date == '17325', ]
dat.linmod.sh <- dat.linmod.sh[!dat.linmod.sh$Date == '17329', ]
dat.linmod.sh <- dat.linmod.sh[!dat.linmod.sh$Date == '17330', ]
dat.linmod.sh <- dat.linmod.sh[!dat.linmod.sh$Date == '17331', ]

dat.linmod.sh$Agec <- scale(dat.linmod.sh$Age)
dat.linmod.sh$CalvingDatec <- scale(dat.linmod.sh$CalvingDate)
dat.linmod.sh$MilkYieldc <- scale(dat.linmod.sh$MilkYield)
```

### Linear Models

Model with homogenous variance

```
lme.out.sh.null <- lme(EntryQuantile ~  Agec + CalvingDatec + MilkYieldc  + Treatment + Agec*MilkYieldc + Agec*CalvingDatec + MilkYieldc*CalvingDatec , 
                   random = ~ 1 | CowID, 
                   data = dat.linmod.sh,
                   control=ctrl,
                   method = 'ML')

summary(lme.out.sh.null)
```

```
## Linear mixed-effects model fit by maximum likelihood
##  Data: dat.linmod.sh 
##         AIC       BIC   logLik
##   -6294.924 -6221.576 3157.462
## 
## Random effects:
##  Formula: ~1 | CowID
##         (Intercept) Residual
## StdDev:   0.2252032 0.177063
## 
## Fixed effects: EntryQuantile ~ Agec + CalvingDatec + MilkYieldc + Treatment +      Agec * MilkYieldc + Agec * CalvingDatec + MilkYieldc * CalvingDatec 
##                              Value  Std.Error    DF   t-value p-value
## (Intercept)              0.4624406 0.02981745 11165 15.509062  0.0000
## Agec                    -0.0681541 0.02555611   152 -2.666841  0.0085
## CalvingDatec            -0.0088646 0.01882919   152 -0.470788  0.6385
## MilkYieldc               0.0571248 0.02570924   152  2.221956  0.0278
## TreatmentOrganilac       0.0179149 0.03595735   152  0.498228  0.6190
## Agec:MilkYieldc          0.0359506 0.02293526   152  1.567479  0.1191
## Agec:CalvingDatec       -0.0221320 0.02366395   152 -0.935260  0.3511
## CalvingDatec:MilkYieldc  0.0229342 0.02813267   152  0.815217  0.4162
##  Correlation: 
##                         (Intr) Agec   ClvngD MlkYld TrtmnO Agc:MY Agc:CD
## Agec                     0.190                                          
## CalvingDatec            -0.003 -0.181                                   
## MilkYieldc              -0.212 -0.675 -0.008                            
## TreatmentOrganilac      -0.614 -0.049 -0.007  0.010                     
## Agec:MilkYieldc         -0.462 -0.279  0.063  0.377 -0.042              
## Agec:CalvingDatec       -0.143 -0.134 -0.151  0.049  0.034  0.051       
## CalvingDatec:MilkYieldc  0.093  0.103 -0.025  0.003 -0.031 -0.192 -0.691
## 
## Standardized Within-Group Residuals:
##         Min          Q1         Med          Q3         Max 
## -4.45461845 -0.58916296 -0.04352043  0.60231231  5.05080749 
## 
## Number of Observations: 11325
## Number of Groups: 160
```

```
anova(lme.out.sh.null)
```

```
##                         numDF denDF  F-value p-value
## (Intercept)                 1 11165 762.5329  <.0001
## Agec                        1   152   3.8388  0.0519
## CalvingDatec                1   152   0.4008  0.5276
## MilkYieldc                  1   152   2.9154  0.0898
## Treatment                   1   152   0.3766  0.5404
## Agec:MilkYieldc             1   152   2.9197  0.0895
## Agec:CalvingDatec           1   152   0.2646  0.6077
## CalvingDatec:MilkYieldc     1   152   0.6646  0.4162
```

```
qqnorm(lme.out.sh.null)
```

Model with heterogenous variance

```
ctrl <- lmeControl(opt='optim')
lme.out.sh <- lme(EntryQuantile ~  Agec + CalvingDatec + MilkYieldc  + Treatment + Agec*MilkYieldc + Agec*CalvingDatec + MilkYieldc*CalvingDatec , 
                   random = ~ 1 | CowID, 
                   weights = varIdent(form = ~ 1 | CowID),
                   data = dat.linmod.sh,
                   control=ctrl,
                   method = 'ML') 

summary(lme.out.sh)
```

```
## Linear mixed-effects model fit by maximum likelihood
##  Data: dat.linmod.sh 
##         AIC       BIC   logLik
##   -8354.217 -7114.641 4346.108
## 
## Random effects:
##  Formula: ~1 | CowID
##         (Intercept)  Residual
## StdDev:    0.226096 0.1425789
## 
## Variance function:
##  Structure: Different standard deviations per stratum
##  Formula: ~1 | CowID 
##  Parameter estimates:
##         5       443       449       645       757      1038      1090 
## 1.0000000 1.1444604 1.2330414 1.7325906 1.2762424 1.1524400 0.6608124 
##      1135      1198      1371      1424      1431      1454      1511 
## 1.0617712 1.5544843 1.4138993 1.4472365 1.5727957 0.6850007 1.4727781 
##      1521      1839      1951      2053      2219      2457      2558 
## 1.0694193 1.9113664 1.6223466 0.3457185 1.5631500 0.9954283 1.5202757 
##      4562      4965      6635      7057      7708      7941      9048 
## 0.6978449 1.6282627 1.3667409 0.9679164 1.1321630 1.1694092 1.2689369 
##      9130      9323      9363     10318     10500     10837     10985 
## 0.6097393 0.6683264 1.2228587 1.7941253 1.4820876 2.0475916 1.2863398 
##     11298     11690     13077     13267     13407     13429     13460 
## 1.1721166 1.4162718 0.7006522 0.8051014 1.2675492 1.4575581 1.0534444 
##     13467     13469     13470     13482     13485     13496     13630 
## 1.6750751 1.6529703 1.1004012 1.2617705 0.9926903 0.6992963 1.4884456 
##     13802     13808     13812     13814     13826     13831     13836 
## 0.9237134 1.3596875 0.8711338 0.6695810 1.9352926 1.3021573 0.5029567 
##     13933     13936     13946     13952     13956     14256     16229 
## 1.3086410 1.3358248 1.1270479 1.3131668 1.3011681 1.2118406 0.3814831 
##     16739     16829     17001     18175     18649     18781     18802 
## 1.0819368 0.1581124 1.2128995 1.2940459 1.2445451 1.4828483 1.2437669 
##     18828     19052     19185     19403     19484     20216     20311 
## 1.6750964 1.3560541 1.1943201 0.8676025 1.2490202 1.4829767 1.0337403 
##     20531     21292     21906     22179     22234     22948     23049 
## 1.2023779 1.1396207 1.2204168 0.9825441 1.4681089 1.2532335 1.1327038 
##     23314     23325     23428     23461     23502     23630     25816 
## 1.4913533 1.5588543 1.0580138 1.5191468 0.4684759 1.3931983 1.7432813 
##     25846     25853     26067     26068     26228     26451     26853 
## 1.1170521 1.1126453 0.7194228 0.6134605 1.2703802 0.6701673 0.7580011 
##     26905     26934     26983     27050     27335     27340     27423 
## 1.2778245 1.1624097 1.5314036 1.3459397 1.1792218 0.8282584 0.9206991 
##     31867     31889     31904     32607     32949     33199     33513 
## 0.4257291 0.8940412 0.8153070 0.6973695 0.8795532 1.5576793 1.3564620 
##     33908     34350     34359     34360     34368     34369     34372 
## 0.9509447 1.5722345 0.6831885 1.0003966 1.0514712 1.3588277 2.3875652 
##     34380     45551     45724     45757     46769     46926     48576 
## 1.6046060 1.1336208 1.7939599 1.2463988 0.8770708 1.2048629 1.4087933 
##     49065     50307     53683     55389     55425     55454     55467 
## 1.1714572 1.1741574 1.4915282 0.7836187 0.6526383 0.3963570 1.4755552 
##     55482     55516     55524     55562     55723     55773     55785 
## 0.8409338 1.5132152 1.4494436 1.4838855 1.4039269 1.4605278 1.1287356 
##     55832     55887     55952     62002     63530     63811     63911 
## 1.2788523 0.9194161 0.4339061 0.5730972 0.5898349 1.3068841 1.5667613 
##     65154     65469     96023     96539     97444     97907     97933 
## 1.2361250 0.9743276 1.2685111 1.2002527 1.2761292 1.3134554 1.3100151 
##     97974     98038     98737     98739     98771     98777 
## 1.5507050 1.3301323 1.8375228 1.2097567 1.4176780 1.2252279 
## Fixed effects: EntryQuantile ~ Agec + CalvingDatec + MilkYieldc + Treatment +      Agec * MilkYieldc + Agec * CalvingDatec + MilkYieldc * CalvingDatec 
##                              Value  Std.Error    DF   t-value p-value
## (Intercept)              0.4622907 0.02993494 11165 15.443178  0.0000
## Agec                    -0.0682598 0.02564299   152 -2.661927  0.0086
## CalvingDatec            -0.0090151 0.01890553   152 -0.476850  0.6342
## MilkYieldc               0.0573756 0.02580529   152  2.223407  0.0277
## TreatmentOrganilac       0.0179504 0.03609804   152  0.497269  0.6197
## Agec:MilkYieldc          0.0360159 0.02302229   152  1.564393  0.1198
## Agec:CalvingDatec       -0.0221738 0.02373562   152 -0.934199  0.3517
## CalvingDatec:MilkYieldc  0.0230717 0.02824550   152  0.816827  0.4153
##  Correlation: 
##                         (Intr) Agec   ClvngD MlkYld TrtmnO Agc:MY Agc:CD
## Agec                     0.190                                          
## CalvingDatec            -0.002 -0.180                                   
## MilkYieldc              -0.211 -0.675 -0.009                            
## TreatmentOrganilac      -0.614 -0.048 -0.007  0.009                     
## Agec:MilkYieldc         -0.462 -0.279  0.062  0.377 -0.042              
## Agec:CalvingDatec       -0.143 -0.134 -0.152  0.048  0.034  0.051       
## CalvingDatec:MilkYieldc  0.093  0.103 -0.025  0.003 -0.031 -0.191 -0.691
## 
## Standardized Within-Group Residuals:
##        Min         Q1        Med         Q3        Max 
## -4.7069155 -0.6513516 -0.0548821  0.6760470  7.2853775 
## 
## Number of Observations: 11325
## Number of Groups: 160
```

```
anova(lme.out.sh)
```

```
##                         numDF denDF  F-value p-value
## (Intercept)                 1 11165 756.2274  <.0001
## Agec                        1   152   3.8131  0.0527
## CalvingDatec                1   152   0.4081  0.5239
## MilkYieldc                  1   152   2.9261  0.0892
## Treatment                   1   152   0.3751  0.5411
## Agec:MilkYieldc             1   152   2.9094  0.0901
## Agec:CalvingDatec           1   152   0.2616  0.6098
## CalvingDatec:MilkYieldc     1   152   0.6672  0.4153
```

```
qqnorm(as.vector(lme.out.sh$residuals))
```

Test significance of variance model

```
anova(lme.out.sh.null, lme.out.sh)
```

```
##                 Model  df       AIC       BIC   logLik   Test  L.Ratio
## lme.out.sh.null     1  10 -6294.924 -6221.576 3157.462                
## lme.out.sh          2 169 -8354.217 -7114.641 4346.108 1 vs 2 2377.293
##                 p-value
## lme.out.sh.null        
## lme.out.sh       <.0001
```

### Visualization

```
temp <- unique(data.frame(CowID = dat.linmod.sh$CowID, Age = dat.linmod.sh$Age))
temp2 <- unique(data.frame(CowID = names(predict(lme.out.sh)), Predicted = predict(lme.out.sh)))
predict.sh.age <- merge(temp, temp2, by = 'CowID')
dim(predict.sh.age)
```

```
## [1] 160   3
```

```
temp <- unique(data.frame(CowID = dat.linmod.sh$CowID, MilkYield = dat.linmod.sh$MilkYield))
predict.sh.ageyield <- merge(predict.sh.age, temp, by = 'CowID')

temp <- c(1, coef(lme.out.sh$modelStruct$varStruct, unconstrained=F))
names(temp)[1] <- names(predict(lme.out.sh))[1]
temp <- (temp *lme.out.sh$sigma)^2
temp <- temp/min(temp)
temp2 <- data.frame(CowID = names(temp), Variance = temp)
predict.sh.ageyield <- merge(predict.sh.ageyield, temp2, by = 'CowID')


ggplot(predict.sh.ageyield, aes(x=Age, y=Predicted, colour = MilkYield, size = 1/Variance)) +
          geom_point() + 
          ylim(0,1) +
          ggtitle('Association Between Queue Position and Cow Age: All Cows') + 
          xlab('Age in Days') +
          ylab('Predicted Queue Position') + 
          scale_color_viridis() +
          labs(fill = "Peak Yield")
```

```
if(isexporting){
  jpeg('Viz/QueueVsAge_All.jpg', width = 8, height = 5, units = 'in', res = 300)
  ggplot(predict.sh.ageyield, aes(x=Age, y=Predicted, colour = MilkYield, size = 1/Variance)) +
    geom_point() + 
    ylim(0,1) +
    ggtitle('Association Between Queue Position and Cow Age: All Cows') + 
    xlab('Age in Days') +
    ylab('Predicted Queue Position') + 
    scale_color_viridis() +
    labs(fill = "Peak Yield") 
  dev.off()
}
```

```
## quartz_off_screen 
##                 2
```

```
ggplot(predict.sh.ageyield, aes(x=MilkYield, y=Predicted, colour = Age, size = 1/Variance)) +
          geom_point() + 
          ylim(0,1) +
          ggtitle('Association Between Queue Position and Milk Yield: All Cows') + 
          xlab('Peak Milk Yield') +
          ylab('Predicted Queue Position') + 
          scale_color_viridis()
```

```
          #labs(fill = "Milking Yield") 
          #geom_vline(xintercept = 17280, colour='grey')

if(isexporting){
  jpeg('Viz/QueueVsYield_All.jpg', width = 8, height = 5, units = 'in', res = 300)
  ggplot(predict.sh.ageyield, aes(x=MilkYield, y=Predicted, colour = Age, size = 1/Variance)) +
    geom_point() + 
    ylim(0,1) +
    ggtitle('Association Between Queue Position and Milk Yield: All Cows') + 
    xlab('Peak Milk Yield') +
    ylab('Predicted Queue Position') + 
    scale_color_viridis() 
  #labs(fill = "Milking Yield") 
  #geom_vline(xintercept = 17280, colour='grey')
  dev.off()
}
```

```
## quartz_off_screen 
##                 2
```

```
cor(predict.sh.ageyield$Age, predict.sh.ageyield$MilkYield)
```

```
## [1] 0.6574068
```

### Mutual Conditional Entropy Test

```
set.seed(1616)


milkquantdat_sickhealthy_dropdays <- milkquantdat_sickhealthy[rownames(milkquantdat_sickhealthy) %in% predict.sh.ageyield$CowID,] 
milkquantdat_sickhealthy_dropdays <- milkquantdat_sickhealthy_dropdays[ , !names(milkquantdat_sickhealthy_dropdays) %in% c('17325', '17329', '17330', '17331')]
dim(milkquantdat_sickhealthy_dropdays)
```

```
## [1] 160  76
```

```
d <- dist(milkquantdat_sickhealthy_dropdays)
h.sh.reg <- hclust(d, method ='ward.D2') 
plot(h.sh.reg)
```

```
temp <- predict.sh.ageyield$Age
names(temp) <- predict.sh.ageyield$CowID
d <- dist(temp)
h.age.shreg <- hclust(d, method ='ward.D2')
plot(h.age.shreg)
```

```
temp <- predict.sh.ageyield$MilkYield
names(temp) <- predict.sh.ageyield$CowID
d <- dist(temp)
h.yield.shreg <- hclust(d, method ='ward.D2') 
plot(h.yield.shreg)
```

```
#Association with Age
print('age')
```

```
## [1] "age"
```

```
btreeout_sh_queue_age <- bivartree(h.sh.reg, h.age.shreg, xrange = 2:8, yrange = 2:6)
btreeout_sh_queue_age$SignificanceMatrix
```

```
##        k=2    k=3    k=4    k=5    k=6
## k=2 0.0940 0.2075 0.3400 0.4715 0.6550
## k=3 0.0625 0.1260 0.2830 0.4620 0.6810
## k=4 0.1390 0.2890 0.4300 0.6570 0.7905
## k=5 0.2540 0.2550 0.3775 0.6250 0.7480
## k=6 0.3145 0.3365 0.3260 0.5350 0.6855
## k=7 0.3085 0.1235 0.0195 0.0855 0.1850
## k=8 0.3880 0.2185 0.0465 0.2105 0.3785
```

```
pout_sh_queue_age <- getBVTPval(btreeout_sh_queue_age$SignificanceMatrix)
pout_sh_queue_age
```

```
## $pval
## [1] 0.3085
## 
## $row
## k=7 
##   6 
## 
## $col
## k=2 
##   1
```

```
temp <- cutree(h.sh.reg, 7)
temp2 <- data.frame(CowID = names(temp), Queue7 = temp)
predict.sh.ageyield <- merge(predict.sh.ageyield, temp2, by = 'CowID')

temp <- cutree(h.age.shreg, 2)
temp2 <- data.frame(CowID = names(temp), Age2 = temp)
predict.sh.ageyield <- merge(predict.sh.ageyield, temp2, by = 'CowID')

tempage <- tapply(predict.sh.ageyield$Age, predict.sh.ageyield$Age2, median)
tempqueue <- tapply(predict.sh.ageyield$Predicted, predict.sh.ageyield$Queue7, median)

discreg_age_sh <- data.frame(Queue = NA, Age = NA, Size = NA)
for(i in 1:length(tempage)){
  for(j in 1:length(tempqueue)){
    
    tempsum <- sum(predict.sh.ageyield$Age2 ==i & predict.sh.ageyield$Queue7 == j)
    if(!tempsum == 0){
      temp <- data.frame(Queue = tempqueue[j], Age = tempage[i], Size = tempsum)
      discreg_age_sh <- rbind(discreg_age_sh, temp)
    }
    
  }
}

ggplot(discreg_age_sh, aes(y=Queue, x=Age, size = Size)) +
          geom_point() + 
          ylim(0,1) +
          ggtitle('Association Between Queue Position and Cow Age: All Cows') + 
          xlab('Age in Days Cluster Median') +
          ylab('Predicted Queue Position Cluster Median') + 
          scale_color_viridis()
```

```
## Warning: Removed 1 rows containing missing values (geom_point).
```

```
if(isexporting){
  jpeg('Viz/MCE_Age_all.jpg', width = 8, height = 5, units = 'in', res = 300)
  ggplot(discreg_age_sh, aes(y=Queue, x=Age, size = Size)) +
    geom_point() + 
    ylim(0,1) +
    ggtitle('Association Between Queue Position and Cow Age: All Cows') + 
    xlab('Age in Days Cluster Median') +
    ylab('Predicted Queue Position Cluster Median') + 
    scale_color_viridis()
  dev.off()
}
```

```
## quartz_off_screen 
##                 2
```

```
# Association with Yield
print('Yield')
```

```
## [1] "Yield"
```

```
btreeout_sh_queue_yield <- bivartree(h.sh.reg, h.yield.shreg, xrange = 2:8, yrange = 2:7)
btreeout_sh_queue_yield$SignificanceMatrix
```

```
##        k=2    k=3    k=4    k=5    k=6    k=7
## k=2 0.2210 0.0120 0.0180 0.0365 0.0585 0.0760
## k=3 0.3975 0.0145 0.0500 0.0765 0.1000 0.0920
## k=4 0.6025 0.0435 0.1200 0.2305 0.2845 0.3375
## k=5 0.5170 0.0540 0.1955 0.2840 0.3810 0.4550
## k=6 0.5875 0.0600 0.1990 0.3290 0.4580 0.5255
## k=7 0.7160 0.1265 0.3175 0.0860 0.1085 0.1445
## k=8 0.7455 0.1600 0.2745 0.0755 0.0885 0.1125
```

```
pout_sh_queue_yield <- getBVTPval(btreeout_sh_queue_yield$SignificanceMatrix)
pout_sh_queue_yield
```

```
## $pval
## [1] 0.012
## 
## $row
## k=2 
##   1 
## 
## $col
## k=3 
##   2
```

```
temp <- cutree(h.sh.reg, 2)
temp2 <- data.frame(CowID = names(temp), Queue2 = temp)
predict.sh.ageyield <- merge(predict.sh.ageyield, temp2, by = 'CowID')


temp <- cutree(h.yield.shreg, 3)
temp2 <- data.frame(CowID = names(temp), Yield5 = temp)
predict.sh.ageyield <- merge(predict.sh.ageyield, temp2, by = 'CowID')

tempyield <- tapply(predict.sh.ageyield$MilkYield, predict.sh.ageyield$Yield5, median)
tempqueue <- tapply(predict.sh.ageyield$Predicted, predict.sh.ageyield$Queue2, median)

discreg_yield_sh <- data.frame(Queue = NA, MilkYield = NA, Size = NA)
for(i in 1:length(tempyield)){
  for(j in 1:length(tempqueue)){
    
    tempsum <- sum(predict.sh.ageyield$Yield5 ==i & predict.sh.ageyield$Queue2 == j)
    if(!tempsum == 0){
      temp <- data.frame(Queue = tempqueue[j], MilkYield = tempyield[i], Size = tempsum)
      discreg_yield_sh <- rbind(discreg_yield_sh, temp)
    }
    
  }
}

ggplot(discreg_yield_sh, aes(y=Queue, x=MilkYield, size = Size)) +
          geom_point() + 
          ylim(0,1) +
          ggtitle('Association Between Queue Position and Peak Yield: All Cows') + 
          xlab('Peak Milk Yield Cluster Median') +
          ylab('Predicted Queue Position Cluster Median ') + 
          scale_color_viridis()
```

```
## Warning: Removed 1 rows containing missing values (geom_point).
```

```
if(isexporting){
  jpeg('Viz/MCE_Yield_all.jpg', width = 8, height = 5, units = 'in', res = 300)
  ggplot(discreg_yield_sh, aes(y=Queue, x=MilkYield, size = Size)) +
    geom_point() + 
    ylim(0,1) +
    ggtitle('Association Between Queue Position and Peak Yield: All Cows') + 
    xlab('Peak Milk Yield Cluster Median') +
    ylab('Predicted Queue Position Cluster Median ') + 
    scale_color_viridis()
  dev.off()
}
```

```
## quartz_off_screen 
##                 2
```

# Exploratory Analysis of Sensor Records

## Mixed Model Analysis

First, I need to get this data into the right format. Peddat is already in long format, so I just need to add my group attributes from the queue data. I will do this by arbitrarily dividing the herd into four groups based on their median entry position. Cows consistently entering the queue first will be the first quantile, cows entering last will be the fourth quantile

```
# get groups

medgroupdat <- data.frame(Id = rownames(milkquantdat_all2), 
                          med = apply(milkquantdat_all2,1, 
                                      function(x) median(x, na.rm = T)))

medgroupdat$medrank <- rank(medgroupdat$med) 
medgroupdat$medrankq <- medgroupdat$medrank/nrow(milkquantdat_all2)

medgroupdat$group <- ifelse(medgroupdat$medrankq<0.25,'1st Quartile',NA)
medgroupdat$group <- ifelse(medgroupdat$medrankq>=0.25 &
                              medgroupdat$medrankq<0.5, '2nd Quartile', medgroupdat$group)
medgroupdat$group <- ifelse(medgroupdat$medrankq>=0.5 &
                              medgroupdat$medrankq<0.75, '3rd Quartile', medgroupdat$group)
medgroupdat$group <- ifelse(is.na(medgroupdat$group), '4th Quartile', medgroupdat$group)

# add to peddat

peddat2 <- merge(peddat, medgroupdat[,c('Id','group')], by = 'Id', all.x = T)
peddat2$group <- as.factor(peddat2$group)

peddat2 <- peddat2[!is.na(peddat2$group), ]
```

Alright now lets fit a linear model to get the mean values and error bars of these groups.

### Activity

```
ctrl <- lmeControl(opt='optim')
lme.out.act <- lme(Active ~  group*Hourc + group*Datec , 
                   random = ~ 1 | Id, 
                   data = peddat2,
                   control=ctrl,
                   method = 'REML')

#summary(lme.out.act)
anova(lme.out.act)
```

```
##             numDF  denDF   F-value p-value
## (Intercept)     1 109507 3011.3366  <.0001
## group           3    105    5.9837   8e-04
## Hourc          23 109507 1948.9262  <.0001
## Datec          41 109507   32.8284  <.0001
## group:Hourc    69 109507   25.5035  <.0001
## group:Datec   123 109507    1.4488   9e-04
```

```
#peddat2$fitted_rum <- fitted(lme.out.rum,0)

#unique(peddat2[,c('Id', 'group')])  
# 3 = 645, 1421
# 1 = 1090, 1454
# 2 = 1135, 2558 
# 4 = 1371, 1424

emmip(lme.out.act, group~Hourc,CIs = T, ylab = 'Activity', xlab = 'Hour') + 
  ggtitle('Comparison of Activity Patterns by Queue Group: 24 Hour Pattern') + 
  labs(color="Queue Group")
```

```
emmip(lme.out.act, group~Datec,CIs = T, ylab = 'Activity', xlab = 'Days on Trial') + 
  ggtitle('Comparison of Activity Patterns by Queue Group: Longitudinal Pattern') + 
  labs(color="Queue Group")  +
  theme(axis.text.x = element_text(size = 10, angle = 90))
```

```
if(isexporting){
  jpeg('Viz/BivariateAnalyses/MeanPlots/Activity_Cyc.jpg', width = 10, height = 6, units = 'in', res = 300)
  emmip(lme.out.act, group~Hourc,CIs = T, ylab = 'Activity', xlab = 'Hour') + 
    ggtitle('Comparison of Activity Patterns by Queue Group: 24 Hour Pattern') + 
    labs(color="Queue Group") 
  dev.off()
  
  jpeg('Viz/BivariateAnalyses/MeanPlots/Activity_Long.jpg', width = 10, height = 6, units = 'in', res = 300)
  emmip(lme.out.act, group~Datec,CIs = T, ylab = 'Activity', xlab = 'Days on Trial') + 
    ggtitle('Comparison of Activity Patterns by Queue Group: Longitudinal Pattern') + 
    labs(color="Queue Group")  +
    theme(axis.text.x = element_text(size = 10, angle = 90))
  dev.off()
}
```

```
## quartz_off_screen 
##                 2
```

### High Activity

```
ctrl <- lmeControl(opt='optim')
lme.out.hiact <- lme(HighActive ~  group*Hourc + group*Datec , 
                   random = ~ 1 | Id, 
                   data = peddat2,
                   control=ctrl,
                   method = 'REML')

#summary(lme.out.hiact)
anova(lme.out.hiact)
```

```
##             numDF  denDF   F-value p-value
## (Intercept)     1 109507 2307.1331  <.0001
## group           3    105    0.4987   0.684
## Hourc          23 109507 1214.1824  <.0001
## Datec          41 109507   46.0400  <.0001
## group:Hourc    69 109507   14.0064  <.0001
## group:Datec   123 109507    2.3966  <.0001
```

```
emmip(lme.out.hiact, group~Hourc,CIs = T, ylab = 'High Activity', xlab = 'Hour') + 
    ggtitle('Comparison of High Activity Patterns by Queue Group: 24 Hour Pattern') + 
    labs(color="Queue Group")
```

```
emmip(lme.out.hiact, group~Datec,CIs = T, ylab = 'High Activity', xlab = 'Days on Trial') + 
    ggtitle('Comparison of High Activity Patterns by Queue Group: Longitudinal Pattern') + 
    labs(color="Queue Group")  +
    theme(axis.text.x = element_text(size = 10, angle = 90))
```

```
if(isexporting){
  jpeg('Viz/BivariateAnalyses/MeanPlots/HighActivity_Cyc.jpg', width = 10, height = 6, units = 'in', res = 300)
  emmip(lme.out.hiact, group~Hourc,CIs = T, ylab = 'High Activity', xlab = 'Hour') + 
    ggtitle('Comparison of High Activity Patterns by Queue Group: 24 Hour Pattern') + 
    labs(color="Queue Group") 
  dev.off()
  
  jpeg('Viz/BivariateAnalyses/MeanPlots/HighActivity_Long.jpg', width = 10, height = 6, units = 'in', res = 300)
  emmip(lme.out.hiact, group~Datec,CIs = T, ylab = 'High Activity', xlab = 'Days on Trial') + 
    ggtitle('Comparison of High Activity Patterns by Queue Group: Longitudinal Pattern') + 
    labs(color="Queue Group")  +
    theme(axis.text.x = element_text(size = 10, angle = 90))
  dev.off()
}
```

```
## quartz_off_screen 
##                 2
```

### Nonactivity

```
ctrl <- lmeControl(opt='optim')
lme.out.nonact <- lme(NotActive ~  group*Hourc + group*Datec , 
                   random = ~ 1 | Id, 
                   data = peddat2,
                   control=ctrl,
                   method = 'REML')

#summary(lme.out.nonact)
anova(lme.out.nonact)
```

```
##             numDF  denDF  F-value p-value
## (Intercept)     1 109507 3302.682  <.0001
## group           3    105    0.503  0.6813
## Hourc          23 109507  725.426  <.0001
## Datec          41 109507    7.549  <.0001
## group:Hourc    69 109507   13.142  <.0001
## group:Datec   123 109507    0.722  0.9913
```

```
emmip(lme.out.nonact, group~Hourc,CIs = T, ylab = 'Non-Activity', xlab = 'Hour') + 
    ggtitle('Comparison of Non-Activity Patterns by Queue Group: 24 Hour Pattern') + 
    labs(color="Queue Group")
```

```
emmip(lme.out.nonact, group~Datec,CIs = T, ylab = 'Non-Activity', xlab = 'Days on Trial') + 
    ggtitle('Comparison of Non-Activity Patterns by Queue Group: Longitudinal Pattern') + 
    labs(color="Queue Group")  +
    theme(axis.text.x = element_text(size = 10, angle = 90))
```

```
if(isexporting){
  jpeg('Viz/BivariateAnalyses/MeanPlots/NonActivity_Cyc.jpg', width = 10, height = 6, units = 'in', res = 300)
  emmip(lme.out.nonact, group~Hourc,CIs = T, ylab = 'Non-Activity', xlab = 'Hour') + 
    ggtitle('Comparison of Non-Activity Patterns by Queue Group: 24 Hour Pattern') + 
    labs(color="Queue Group") 
  dev.off()
  
  jpeg('Viz/BivariateAnalyses/MeanPlots/NonActivity_Long.jpg', width = 10, height = 6, units = 'in', res = 300)
  emmip(lme.out.nonact, group~Datec,CIs = T, ylab = 'Non-Activity', xlab = 'Days on Trial') + 
    ggtitle('Comparison of Non-Activity Patterns by Queue Group: Longitudinal Pattern') + 
    labs(color="Queue Group")  +
    theme(axis.text.x = element_text(size = 10, angle = 90))
  dev.off()
}
```

```
## quartz_off_screen 
##                 2
```

### Eating

```
ctrl <- lmeControl(opt='optim')
lme.out.eat <- lme(Eating ~  group*Hourc + group*Datec , 
                   random = ~ 1 | Id, 
                   data = peddat2,
                   control=ctrl,
                   method = 'REML')

#summary(lme.out.eat)
anova(lme.out.eat)
```

```
##             numDF  denDF   F-value p-value
## (Intercept)     1 109507 1245.4640  <.0001
## group           3    105    0.2096  0.8896
## Hourc          23 109507 1293.6366  <.0001
## Datec          41 109507   14.5622  <.0001
## group:Hourc    69 109507   26.5571  <.0001
## group:Datec   123 109507    0.3215  1.0000
```

```
emmip(lme.out.eat, group~Hourc,CIs = T, ylab = 'Eating', xlab = 'Hour') + 
    ggtitle('Comparison of Eating Patterns by Queue Group: 24 Hour Pattern') + 
    labs(color="Queue Group")
```

```
emmip(lme.out.eat, group~Datec,CIs = T, ylab = 'Eating', xlab = 'Days on Trial') + 
    ggtitle('Comparison of Eating Patterns by Queue Group: Longitudinal Pattern') + 
    labs(color="Queue Group")  +
    theme(axis.text.x = element_text(size = 10, angle = 90))
```

```
if(isexporting){
  jpeg('Viz/BivariateAnalyses/MeanPlots/Eating_Cyc.jpg', width = 10, height = 6, units = 'in', res = 300)
  emmip(lme.out.eat, group~Hourc,CIs = T, ylab = 'Eating', xlab = 'Hour') + 
    ggtitle('Comparison of Eating Patterns by Queue Group: 24 Hour Pattern') + 
    labs(color="Queue Group") 
  dev.off()
  
  jpeg('Viz/BivariateAnalyses/MeanPlots/Eating_Long.jpg', width = 10, height = 6, units = 'in', res = 300)
  emmip(lme.out.eat, group~Datec,CIs = T, ylab = 'Eating', xlab = 'Days on Trial') + 
    ggtitle('Comparison of Eating Patterns by Queue Group: Longitudinal Pattern') + 
    labs(color="Queue Group")  +
    theme(axis.text.x = element_text(size = 10, angle = 90))
  dev.off()
}
```

```
## quartz_off_screen 
##                 2
```

### Rumination

```
ctrl <- lmeControl(opt='optim')
lme.out.rum <- lme(Ruminating ~  group*Hourc + group*Datec , 
                   random = ~ 1 | Id, 
                   data = peddat2,
                   control=ctrl,
                   method = 'REML')

#summary(lme.out.rum)
anova(lme.out.rum)
```

```
##             numDF  denDF  F-value p-value
## (Intercept)     1 109507 8048.993  <.0001
## group           3    105    1.389  0.2501
## Hourc          23 109507 1240.799  <.0001
## Datec          41 109507    4.396  <.0001
## group:Hourc    69 109507   11.032  <.0001
## group:Datec   123 109507    0.688  0.9967
```

```
emmip(lme.out.rum, group~Hourc,CIs = T, ylab = 'Ruminating', xlab = 'Hour') + 
    ggtitle('Comparison of Rumination Patterns by Queue Group: 24 Hour Pattern') + 
    labs(color="Queue Group")
```

```
emmip(lme.out.rum, group~Datec,CIs = T, ylab = 'Ruminating', xlab = 'Days on Trial') + 
    ggtitle('Comparison of Rumination Patterns by Queue Group: Longitudinal Pattern') + 
    labs(color="Queue Group")  +
    theme(axis.text.x = element_text(size = 10, angle = 90))
```

```
if(isexporting){
  jpeg('Viz/BivariateAnalyses/MeanPlots/Ruminating_Cyc.jpg', width = 10, height = 6, units = 'in', res = 300)
  emmip(lme.out.rum, group~Hourc,CIs = T, ylab = 'Ruminating', xlab = 'Hour') + 
    ggtitle('Comparison of Rumination Patterns by Queue Group: 24 Hour Pattern') + 
    labs(color="Queue Group") 
  dev.off()
  
  jpeg('Viz/BivariateAnalyses/MeanPlots/Ruminating_Long.jpg', width = 10, height = 6, units = 'in', res = 300)
  emmip(lme.out.rum, group~Datec,CIs = T, ylab = 'Ruminating', xlab = 'Days on Trial') + 
    ggtitle('Comparison of Rumination Patterns by Queue Group: Longitudinal Pattern') + 
    labs(color="Queue Group")  +
    theme(axis.text.x = element_text(size = 10, angle = 90))
  dev.off()
}
```

```
## quartz_off_screen 
##                 2
```

### Temperature

```
ctrl <- lmeControl(opt='optim')
lme.out.temp <- lme(Temperature ~  group*Hourc + group*Datec , 
                   random = ~ 1 | Id, 
                   data = peddat2,
                   control=ctrl,
                   method = 'REML')

#summary(lme.out.temp)
anova(lme.out.temp)
```

```
##             numDF  denDF  F-value p-value
## (Intercept)     1 109507 8615.589  <.0001
## group           3    105    3.047   0.032
## Hourc          23 109507 3949.001  <.0001
## Datec          41 109507 1839.102  <.0001
## group:Hourc    69 109507   19.364  <.0001
## group:Datec   123 109507    2.252  <.0001
```

```
emmip(lme.out.temp, group~Hourc,CIs = T, ylab = 'Body Temperature', xlab = 'Hour') + 
    ggtitle('Comparison of Body Temperature Patterns by Queue Group: 24 Hour Pattern') + 
    labs(color="Queue Group")
```

```
emmip(lme.out.temp, group~Datec,CIs = T,ylab = 'Body Temperature', xlab = 'Days on Trial') +
   ggtitle('Comparison of Body Temperature Patterns by Queue Group: Longitudinal Pattern') +
   labs(color="Queue Group")  +
   theme(axis.text.x = element_text(size = 10, angle = 90))
```

```
if(isexporting){
  jpeg('Viz/BivariateAnalyses/MeanPlots/Temperature_Cyc.jpg', width = 10, height = 6, units = 'in', res = 300)
  emmip(lme.out.temp, group~Hourc,CIs = T, ylab = 'Body Temperature', xlab = 'Hour') + 
    ggtitle('Comparison of Body Temperature Patterns by Queue Group: 24 Hour Pattern') + 
    labs(color="Queue Group") 
  dev.off()
  
  jpeg('Viz/BivariateAnalyses/MeanPlots/Temperature_Long.jpg', width = 10, height = 6, units = 'in', res = 300)
  emmip(lme.out.temp, group~Datec,CIs = T, ylab = 'Body Temperature', xlab = 'Days on Trial') + 
    ggtitle('Comparison of Body Temperature Patterns by Queue Group: Longitudinal Pattern') + 
    labs(color="Queue Group")  +
    theme(axis.text.x = element_text(size = 10, angle = 90))
  dev.off()
}
```

```
## quartz_off_screen 
##                 2
```

## Analysis by Mutual Conditional Entorpy

First, I want to make sure that the cows I’m using in the linear models are the same ones that I’m using in the MCE tests, so that I’m not comparing the results of apple and oranges

```
cowlist_peddat2 <- unique(peddat2$Id)
cowlist_healthy_sensor <- cowlist_complete[cowlist_complete %in% cowlist_peddat2] # & cowlist_complete %in% rownames(milkquantdat_all2)]
length(cowlist_healthy_sensor)
```

```
## [1] 109
```

### Non-Activity

```
set.seed(161916)


# nonactivedat$TimeStampC <- as.character(nonactivedat$TimeStampF)
# nonactivedat$Hour  <- as.numeric(substring(nonactivedat$TimeStampC, 12,13))


# all data

dat <- t(nonactivedat[,names(nonactivedat) %in% cowlist_healthy_sensor])
d <- dist(dat)
h.nonact <- hclust(d, method = 'ward.D2') 
plot(h.nonact, xlab = 'Cow ID',  main = 'Dendrogram of Nonactivity Data')
```

```
# night time data

dat <- t(nonactivedat_night[,names(nonactivedat_night) %in% cowlist_healthy_sensor])
d <- dist(dat)
h.nonactn <- hclust(d, method ='ward.D2') 
plot(h.nonactn, xlab = 'Cow ID',  main = 'Dendrogram of Night Time Nonactivity Data')
```

```
# evening time data

dat <- t(nonactivedat_af[,names(nonactivedat_af) %in% cowlist_healthy_sensor])
d <- dist(dat)
h.nactaf <- hclust(d,  method ='ward.D2') 
plot(h.nactaf, xlab = 'Cow ID', main = 'Dendrogram of Afternoon Time Non-Activity Data')
```

```
# morning data

dat <- t(nonactivedat_morning[,names(nonactivedat_morning) %in% cowlist_healthy_sensor])

d <- dist(dat)
h.nactam <- hclust(d, method ='ward.D2') 
plot(h.nactam, xlab = 'Cow ID', main = 'Dendrogram of Morning Time Non-Activity Data')
```

```
# all data w/out milking

dat <- t(nonactivedat_morning[,names(nonactivedat_morning) %in% cowlist_healthy_sensor])
dat <- cbind(dat, 
             t(nonactivedat_af[,names(nonactivedat_af) %in% cowlist_healthy_sensor]))
dat <- cbind(dat, 
             t(nonactivedat_night[,names(nonactivedat_night) %in% cowlist_healthy_sensor]))

d <- dist(dat)
h.nactl<- hclust(d, method ='ward.D2') 
plot(h.nactl, xlab = 'Cow ID', main = 'Dendrogram of Lounging Period Non-Activity Data')
```

```
# Comparison to Qeueu to all nonactivity data

btreeout_queueall_nonact <- bivartree(h.dist.all, h.nonact, xrange = 2:10, yrange = 2:10)
btreeout_queueall_nonact$SignificanceMatrix
```

```
##         k=2    k=3    k=4   k=5    k=6    k=7    k=8    k=9   k=10
## k=2  0.6555 0.8680 0.0000 0e+00 0.0000 0.0000 0.0000 0.0000 0.0015
## k=3  0.8410 0.8965 0.0015 1e-03 0.0035 0.0005 0.0045 0.0060 0.0170
## k=4  0.5045 0.6430 0.0025 2e-03 0.0045 0.0070 0.0115 0.0380 0.1005
## k=5  0.6865 0.6135 0.0025 2e-03 0.0090 0.0030 0.0035 0.0220 0.0630
## k=6  0.4810 0.5355 0.0035 2e-03 0.0055 0.0030 0.0025 0.0250 0.0835
## k=7  0.5455 0.2370 0.0000 5e-04 0.0020 0.0000 0.0000 0.0075 0.0245
## k=8  0.6555 0.3525 0.0005 1e-03 0.0035 0.0015 0.0020 0.0225 0.0930
## k=9  0.7405 0.5415 0.0075 5e-03 0.0095 0.0030 0.0080 0.0735 0.1510
## k=10 0.8320 0.5240 0.0090 7e-03 0.0095 0.0050 0.0055 0.0915 0.2150
```

```
kable(round(btreeout_queueall_nonact$SignificanceMatrix,3), format = "html", caption = 'NonActivity Overall') %>% cat(., file = "Viz/BivariateAnalyses/MCETest/Nonactivity/Overall.html")
btreeout_queueall_nonact$Results[[3]][[6]]
```

```
## $ContingencyTable
##    
##      1  2  3  4  5  6
##   1 23 19  5  1  7  1
##   2  9  4  3  0  1  2
##   3  2 22  4  2  4  0
## 
## $ObserveMCE
##        1 
## 1.558447 
## 
## $PVal
## [1] 0.0035
```

```
# Comparison of Queue to night time nonactivity data

btreeout_queueall_nonactn <- bivartree(h.dist.all, h.nonactn, xrange = 2:10, yrange = 2:10)
kable(round(btreeout_queueall_nonactn$SignificanceMatrix,3), format = "html", caption = 'NonActivity Overnight') %>% cat(., file = "Viz/BivariateAnalyses/MCETest/Nonactivity/Overnight.html")
btreeout_queueall_nonactn$SignificanceMatrix
```

```
##         k=2    k=3    k=4    k=5    k=6    k=7    k=8    k=9   k=10
## k=2  0.6455 0.9335 0.1395 0.0630 0.1325 0.2245 0.2780 0.3660 0.3380
## k=3  0.6230 0.9055 0.3920 0.3080 0.5155 0.6570 0.7885 0.8550 0.8220
## k=4  0.7790 0.8950 0.5765 0.5740 0.3890 0.6165 0.7615 0.8325 0.8485
## k=5  0.8470 0.9635 0.6340 0.5995 0.3745 0.5865 0.7555 0.8505 0.8930
## k=6  0.8725 0.7555 0.2530 0.2485 0.1800 0.3050 0.4660 0.6535 0.7250
## k=7  0.9425 0.6785 0.2960 0.3110 0.2340 0.4025 0.4035 0.5170 0.5285
## k=8  0.9440 0.4545 0.2020 0.2420 0.2040 0.3975 0.3995 0.5330 0.5785
## k=9  0.8770 0.3310 0.1800 0.2320 0.2135 0.4860 0.5075 0.6250 0.6030
## k=10 0.8755 0.4230 0.3015 0.3230 0.2635 0.3615 0.2800 0.4075 0.4320
```

```
# Comparison of Queue to afternoon time nonactivity data

btreeout_queueall_nonactaf <- bivartree(h.dist.all, h.nactaf, xrange = 2:10, yrange = 2:10)
kable(round(btreeout_queueall_nonactaf$SignificanceMatrix,3), format = "html", caption = 'NonActivity Afternoon') %>% cat(., file = "Viz/BivariateAnalyses/MCETest/Nonactivity/Afternoon.html")
btreeout_queueall_nonactaf$SignificanceMatrix
```

```
##         k=2    k=3    k=4    k=5    k=6    k=7    k=8    k=9   k=10
## k=2  0.3605 0.0105 0.0015 0.0020 0.0050 0.0110 0.0140 0.0060 0.0130
## k=3  0.6650 0.0615 0.0125 0.0315 0.0680 0.1465 0.1935 0.1010 0.1690
## k=4  0.6890 0.1390 0.0370 0.0360 0.0955 0.2175 0.3165 0.1160 0.1850
## k=5  0.8625 0.2575 0.0795 0.0605 0.0895 0.2240 0.3060 0.1740 0.1595
## k=6  0.5675 0.2290 0.0780 0.0565 0.0890 0.2545 0.3460 0.1980 0.1500
## k=7  0.6275 0.3105 0.1540 0.0910 0.1480 0.2115 0.3125 0.1870 0.1630
## k=8  0.7115 0.4935 0.2375 0.0615 0.1015 0.1335 0.1945 0.1420 0.1215
## k=9  0.5130 0.4050 0.2470 0.0850 0.1290 0.1875 0.2800 0.1815 0.1630
## k=10 0.6260 0.2815 0.1390 0.0410 0.0685 0.0890 0.1370 0.0920 0.0845
```

```
# Comparison of Queue to morning time nonactivity data

btreeout_queueall_nonactam <- bivartree(h.dist.all, h.nactam, xrange = 2:10, yrange = 2:10)
kable(round(btreeout_queueall_nonactam$SignificanceMatrix,3), format = "html", caption = 'NonActivity Morning') %>% cat(., file = "Viz/BivariateAnalyses/MCETest/Nonactivity/Morning.html")
btreeout_queueall_nonactam$SignificanceMatrix
```

```
##         k=2    k=3    k=4    k=5    k=6    k=7    k=8    k=9   k=10
## k=2  0.5405 0.8295 0.7760 0.8595 0.7245 0.7875 0.7185 0.8645 0.9325
## k=3  0.4585 0.8165 0.8940 0.9460 0.8545 0.7765 0.8020 0.7130 0.6810
## k=4  0.6565 0.9285 0.9190 0.9825 0.9475 0.8350 0.8880 0.8685 0.9025
## k=5  0.7730 0.8925 0.9100 0.9340 0.9095 0.8105 0.8675 0.8310 0.7820
## k=6  0.8010 0.4025 0.6160 0.7895 0.7665 0.6975 0.7625 0.7260 0.6885
## k=7  0.5390 0.3180 0.6440 0.7280 0.5490 0.4515 0.5470 0.5185 0.4775
## k=8  0.5985 0.4545 0.6000 0.6145 0.4695 0.4315 0.5020 0.4865 0.4945
## k=9  0.6905 0.6150 0.7890 0.8315 0.7375 0.7010 0.7955 0.7830 0.7880
## k=10 0.7525 0.6410 0.6055 0.7315 0.6500 0.6300 0.7055 0.6985 0.7230
```

```
# Comparison of Queue to lounging nonactivity data

btreeout_queueall_nonactl <- bivartree(h.dist.all, h.nactl, xrange = 2:10, yrange = 2:10)
kable(round(btreeout_queueall_nonactl$SignificanceMatrix,3), format = "html", caption = 'NonActivity Lounging') %>% cat(., file = "Viz/BivariateAnalyses/MCETest/Nonactivity/Lounging.html")
btreeout_queueall_nonactl$SignificanceMatrix
```

```
##         k=2    k=3    k=4    k=5    k=6    k=7    k=8    k=9   k=10
## k=2  0.8140 0.9570 0.0280 0.0485 0.0765 0.1165 0.0860 0.1235 0.1745
## k=3  0.9650 0.9970 0.1510 0.2810 0.4285 0.5820 0.4360 0.5825 0.7390
## k=4  0.9040 0.9825 0.3360 0.5850 0.7340 0.7870 0.7215 0.8715 0.8840
## k=5  0.9715 0.9865 0.5370 0.4170 0.6215 0.6035 0.6220 0.7615 0.8375
## k=6  0.9835 0.9945 0.6850 0.5920 0.7950 0.8080 0.8410 0.9280 0.9495
## k=7  0.9935 0.9920 0.8020 0.7280 0.8135 0.8450 0.8120 0.8985 0.9405
## k=8  0.9970 0.9955 0.8760 0.8395 0.9060 0.9310 0.9350 0.9760 0.9780
## k=9  0.9330 0.9840 0.8870 0.8475 0.9305 0.9635 0.9510 0.9820 0.9850
## k=10 0.8775 0.9635 0.8445 0.8350 0.9150 0.9775 0.9755 0.9895 0.9935
```

Interesting, there is a significant relationship here between the queue clusterings and nonactivity, which appears to be driven predominantly by afternoon lying patterns.

```
#p.out.nonact.all <- btreeout_queueall_nonact$SignificanceMatrix[3,4]
p.out.nonact.all <- getBVTPval(btreeout_queueall_nonact$SignificanceMatrix)
p.out.nonact.all
```

```
## $pval
## [1] 5e-04
## 
## $row
## k=7 
##   6 
## 
## $col
## k=5 
##   4
```

```
#p.out.nonact.l <- btreeout_queueall_nonactl$SignificanceMatrix[3,3]
p.out.nonact.l <- getBVTPval(btreeout_queueall_nonactl$SignificanceMatrix)
p.out.nonact.l
```

```
## $pval
## [1] 0.0485
## 
## $row
## k=2 
##   1 
## 
## $col
## k=5 
##   4
```

```
#p.out.nonact.am <- btreeout_queueall_nonactam$SignificanceMatrix[3,4]
p.out.nonact.am <- getBVTPval(btreeout_queueall_nonactam$SignificanceMatrix)
p.out.nonact.am
```

```
## $pval
## [1] 0.4315
## 
## $row
## k=8 
##   7 
## 
## $col
## k=7 
##   6
```

```
#p.out.nonact.af <- btreeout_queueall_nonactaf$SignificanceMatrix[3,4]
p.out.nonact.af <- getBVTPval(btreeout_queueall_nonactaf$SignificanceMatrix)
p.out.nonact.af
```

```
## $pval
## [1] 0.002
## 
## $row
## k=2 
##   1 
## 
## $col
## k=5 
##   4
```

```
#p.out.nonact.n <- btreeout_queueall_nonactn$SignificanceMatrix[3,3]
p.out.nonact.n <- getBVTPval(btreeout_queueall_nonactn$SignificanceMatrix)
p.out.nonact.n
```

```
## $pval
## [1] 0.1325
## 
## $row
## k=2 
##   1 
## 
## $col
## k=6 
##   5
```

### Activity

```
set.seed(161916)


# all data

dat <- t(activedat[,names(activedat) %in% cowlist_healthy_sensor])
d <- dist(dat)

h.act <- hclust(d, method ='ward.D2') 
plot(h.act, xlab = 'Cow ID',  main = 'Dendrogram of Activity Data')
```

```
# night time data

dat <- t(activedat_night[,names(activedat_night) %in% cowlist_healthy_sensor])
d <- dist(dat)

h.actn <- hclust(d, method ='ward.D2') 
plot(h.actn, xlab = 'Cow ID',  main = 'Dendrogram of Night Time Activity Data')
```

```
# evening data

dat <- t(activedat_af[,names(activedat_af) %in% cowlist_healthy_sensor])

d <- dist(dat)
h.actaf <- hclust(d, method ='ward.D2') 
plot(h.actaf, xlab = 'Cow ID', main = 'Dendrogram of Afternoon Time Activity Data')
```

```
# morning data

dat <- t(activedat_morning[,names(activedat_morning) %in% cowlist_healthy_sensor])

d <- dist(dat)
h.actam <- hclust(d, method ='ward.D2') 
plot(h.actam, xlab = 'Cow ID', main = 'Dendrogram of Morning Time Activity Data')
```

```
# all data w/out milking

dat <- t(activedat_morning[,names(activedat_morning) %in% cowlist_healthy_sensor])
dat <- cbind(dat, 
             t(activedat_af[,names(activedat_af) %in% cowlist_healthy_sensor]))
dat <- cbind(dat, 
             t(activedat_night[,names(activedat_night) %in% cowlist_healthy_sensor]))

d <- dist(dat)
h.actl<- hclust(d, method ='ward.D2') 
plot(h.actl, xlab = 'Cow ID', main = 'Dendrogram of Lounging Period Activity Data')
```

```
# Comparison to Qeueu to all activity data

btreeout_queueall_act <- bivartree(h.dist.all, h.act, xrange = 2:10, yrange = 2:10)
btreeout_queueall_act$SignificanceMatrix
```

```
##         k=2    k=3    k=4    k=5    k=6    k=7    k=8    k=9   k=10
## k=2  0.0005 0.0005 0.0000 0.0000 0.0000 0.0000 0.0000 0.0000 0.0000
## k=3  0.0000 0.0000 0.0000 0.0000 0.0000 0.0000 0.0000 0.0000 0.0000
## k=4  0.0000 0.0000 0.0000 0.0000 0.0000 0.0000 0.0000 0.0000 0.0005
## k=5  0.0000 0.0005 0.0000 0.0015 0.0015 0.0000 0.0000 0.0010 0.0010
## k=6  0.0000 0.0000 0.0000 0.0015 0.0000 0.0000 0.0000 0.0020 0.0030
## k=7  0.0010 0.0005 0.0005 0.0005 0.0010 0.0000 0.0000 0.0005 0.0025
## k=8  0.0005 0.0025 0.0035 0.0155 0.0055 0.0030 0.0015 0.0040 0.0180
## k=9  0.0035 0.0040 0.0090 0.0265 0.0255 0.0120 0.0135 0.0170 0.0415
## k=10 0.0040 0.0040 0.0105 0.0300 0.0195 0.0125 0.0085 0.0205 0.0425
```

```
btreeout_queueall_act$Results[[2]][[7]]
```

```
## $ContingencyTable
##    
##      1  2  3  4  5  6  7
##   1 35  2 15 13 10  0  0
##   2  6  9  2  1 14  1  1
## 
## $ObserveMCE
##        1 
## 1.326086 
## 
## $PVal
## [1] 0
```

```
btreeout_queueall_act$Results[[6]][[7]]
```

```
## $ContingencyTable
##    
##      1  2  3  4  5  6  7
##   1 12  0  3  4  6  0  0
##   2  1  1  0  1  1  0  0
##   3  8  0  8  2  1  0  0
##   4 14  1  4  6  2  0  0
##   5  5  4  1  1  8  0  1
##   6  1  5  1  0  6  1  0
## 
## $ObserveMCE
##        1 
## 1.934958 
## 
## $PVal
## [1] 0
```

```
kable(round(btreeout_queueall_act$SignificanceMatrix,3), format = "html", caption = 'Activity Overall') %>% cat(., file = "Viz/BivariateAnalyses/MCETest/Activity/Overall.html")


# Comparison to Qeueu to night activity data

btreeout_queueall_actn <- bivartree(h.dist.all, h.actn, xrange = 2:10, yrange = 2:10)
btreeout_queueall_actn$SignificanceMatrix
```

```
##         k=2    k=3    k=4    k=5    k=6    k=7    k=8    k=9   k=10
## k=2  0.0000 0.0000 0.0205 0.0470 0.0250 0.0455 0.0610 0.0330 0.0360
## k=3  0.1585 0.3435 0.1215 0.2425 0.0990 0.1490 0.1855 0.1215 0.1905
## k=4  0.7090 0.9000 0.2555 0.4330 0.3065 0.3910 0.2270 0.1890 0.3165
## k=5  0.0000 0.0580 0.1660 0.2790 0.1375 0.2230 0.1295 0.0990 0.1950
## k=6  0.0430 0.1230 0.3360 0.3230 0.1285 0.1600 0.1040 0.0960 0.0965
## k=7  0.1000 0.2565 0.2885 0.2475 0.0605 0.0790 0.0515 0.0345 0.0430
## k=8  0.1410 0.3185 0.4405 0.3105 0.0780 0.1150 0.0660 0.0645 0.0655
## k=9  0.3320 0.6170 0.5025 0.3680 0.0815 0.1340 0.0995 0.0810 0.1145
## k=10 0.3965 0.5680 0.5730 0.3425 0.1030 0.0940 0.0685 0.0560 0.0810
```

```
kable(round(btreeout_queueall_actn$SignificanceMatrix,3), format = "html", caption = 'Activity Night') %>% cat(., file = "Viz/BivariateAnalyses/MCETest/Activity/Night.html")


# Comparison to Qeueu to afternoon activity data

btreeout_queueall_actaf <- bivartree(h.dist.all, h.actaf, xrange = 2:10, yrange = 2:10)
btreeout_queueall_actaf$SignificanceMatrix
```

```
##         k=2   k=3    k=4    k=5    k=6    k=7    k=8    k=9   k=10
## k=2  0.0120 0e+00 0.0000 0.0000 0.0000 0.0000 0.0005 0.0000 0.0000
## k=3  0.0575 0e+00 0.0000 0.0005 0.0005 0.0005 0.0025 0.0020 0.0020
## k=4  0.0950 0e+00 0.0000 0.0035 0.0035 0.0060 0.0185 0.0130 0.0130
## k=5  0.1600 0e+00 0.0000 0.0000 0.0000 0.0015 0.0005 0.0000 0.0010
## k=6  0.2245 5e-04 0.0000 0.0010 0.0000 0.0010 0.0010 0.0000 0.0005
## k=7  0.1280 5e-04 0.0010 0.0000 0.0010 0.0015 0.0030 0.0000 0.0000
## k=8  0.2325 1e-03 0.0010 0.0010 0.0010 0.0045 0.0090 0.0005 0.0005
## k=9  0.3040 1e-03 0.0035 0.0035 0.0050 0.0070 0.0150 0.0010 0.0040
## k=10 0.2385 3e-03 0.0030 0.0030 0.0050 0.0040 0.0140 0.0020 0.0045
```

```
kable(round(btreeout_queueall_actaf$SignificanceMatrix,3), format = "html", caption = 'Activity Afternoon') %>% cat(., file = "Viz/BivariateAnalyses/MCETest/Activity/Afternoon.html")

# Comparison to Qeueu to morning activity data

btreeout_queueall_actam <- bivartree(h.dist.all, h.actam, xrange = 2:10, yrange = 2:15)
btreeout_queueall_actam$SignificanceMatrix
```

```
##         k=2    k=3    k=4    k=5    k=6    k=7    k=8    k=9   k=10   k=11
## k=2  0.2130 0.1370 0.0835 0.1715 0.0610 0.0610 0.1385 0.1890 0.1550 0.0385
## k=3  0.3335 0.3620 0.3715 0.4835 0.2815 0.3620 0.2875 0.3000 0.2715 0.1485
## k=4  0.5135 0.6845 0.8250 0.7535 0.5775 0.7665 0.5835 0.4510 0.1830 0.1075
## k=5  0.5275 0.4710 0.4665 0.4870 0.5440 0.6190 0.6015 0.5540 0.2285 0.1775
## k=6  0.4615 0.4260 0.4580 0.4925 0.5135 0.6115 0.4600 0.4480 0.2110 0.1580
## k=7  0.5435 0.5550 0.5975 0.5935 0.6925 0.7820 0.6705 0.4810 0.2330 0.2040
## k=8  0.6530 0.7015 0.7655 0.7660 0.8080 0.8850 0.8340 0.6335 0.4125 0.3595
## k=9  0.4340 0.4770 0.5735 0.6110 0.2190 0.3035 0.3805 0.2960 0.1835 0.1510
## k=10 0.5035 0.5825 0.5780 0.6600 0.2745 0.3595 0.3880 0.1250 0.0720 0.0710
##        k=12   k=13   k=14   k=15
## k=2  0.0790 0.1285 0.1470 0.1630
## k=3  0.2345 0.2050 0.2695 0.2775
## k=4  0.2010 0.2055 0.2905 0.2595
## k=5  0.2395 0.2795 0.3345 0.3205
## k=6  0.2095 0.2685 0.3480 0.3345
## k=7  0.2860 0.3955 0.4710 0.5040
## k=8  0.4610 0.6095 0.7120 0.7420
## k=9  0.2350 0.4065 0.5110 0.5820
## k=10 0.1215 0.2750 0.1975 0.2625
```

```
kable(round(btreeout_queueall_actam$SignificanceMatrix,3), format = "html", caption = 'Activity Morning') %>% cat(., file = "Viz/BivariateAnalyses/MCETest/Activity/Morning.html")

# Comparison of Queue to lounging activity data

btreeout_queueall_actl <- bivartree(h.dist.all, h.actl, xrange = 2:10, yrange = 2:10)
kable(round(btreeout_queueall_actl$SignificanceMatrix,3), format = "html", caption = 'Activity Lounging') %>% cat(., file = "Viz/BivariateAnalyses/MCETest/Activity/Lounging.html")
btreeout_queueall_actl$SignificanceMatrix
```

```
##         k=2    k=3    k=4    k=5    k=6    k=7    k=8    k=9   k=10
## k=2  0.0000 0.0000 0.0135 0.0100 0.0075 0.0135 0.0065 0.0090 0.0125
## k=3  0.1690 0.3435 0.1240 0.0935 0.1070 0.0710 0.0500 0.0790 0.0905
## k=4  0.6835 0.8935 0.3445 0.3235 0.3015 0.2310 0.1620 0.1870 0.1850
## k=5  0.0000 0.0650 0.0620 0.0450 0.0470 0.0645 0.0465 0.0755 0.1040
## k=6  0.0345 0.1380 0.1365 0.1175 0.1295 0.0850 0.0735 0.1485 0.1620
## k=7  0.1085 0.2560 0.1725 0.1780 0.1870 0.1405 0.0800 0.1385 0.1785
## k=8  0.1550 0.3385 0.3065 0.3350 0.3215 0.2815 0.1690 0.3170 0.3460
## k=9  0.3195 0.6190 0.4375 0.2770 0.2835 0.2950 0.1910 0.4040 0.4290
## k=10 0.3860 0.5785 0.4825 0.2895 0.3075 0.3440 0.2490 0.3880 0.4290
```

```
#p.out.act.all <- btreeout_queueall_act$SignificanceMatrix[3,6]
p.out.act.all <- getBVTPval(btreeout_queueall_act$SignificanceMatrix)
p.out.act.all
```

```
## $pval
## [1] 0
## 
## $row
## k=3 
##   2 
## 
## $col
## k=8 
##   7
```

```
#p.out.act.l <- btreeout_queueall_actl$SignificanceMatrix[3,6]
p.out.act.l <- getBVTPval(btreeout_queueall_actl$SignificanceMatrix)
p.out.act.l
```

```
## $pval
## [1] 0.0065
## 
## $row
## k=2 
##   1 
## 
## $col
## k=8 
##   7
```

```
#p.out.act.am <- btreeout_queueall_actam$SignificanceMatrix[3,6]
p.out.act.am <- getBVTPval(btreeout_queueall_actam$SignificanceMatrix)
p.out.act.am
```

```
## $pval
## [1] 0.0385
## 
## $row
## k=2 
##   1 
## 
## $col
## k=11 
##   10
```

```
#p.out.act.af <- btreeout_queueall_actaf$SignificanceMatrix[3,5]
p.out.act.af <- getBVTPval(btreeout_queueall_actaf$SignificanceMatrix)
p.out.act.af
```

```
## $pval
## [1] 0
## 
## $row
## k=2 
##   1 
## 
## $col
## k=3 
##   2
```

```
#p.out.act.n <- btreeout_queueall_actn$SignificanceMatrix[3,5]
p.out.act.n <- getBVTPval(btreeout_queueall_actn$SignificanceMatrix)
p.out.act.n
```

```
## $pval
## [1] 0.033
## 
## $row
## k=2 
##   1 
## 
## $col
## k=9 
##   8
```

### High Activity

```
set.seed(161916)


# all data

dat <- t(hiactivedat[,names(hiactivedat) %in% cowlist_healthy_sensor])
d <- dist(dat)

h.hiact <- hclust(d,  method ='ward.D2') 
plot(h.hiact, xlab = 'Cow ID',  main = 'Dendrogram of High Activity Data')
```

```
# night time data

dat <- t(hiactivedat_night[,names(hiactivedat_night) %in% cowlist_healthy_sensor])
d <- dist(dat)

h.hiactn <- hclust(d, method ='ward.D2') 
plot(h.hiactn, xlab = 'Cow ID',  main = 'Dendrogram of Night Time High Activity Data')
```

```
# evening data


dat <- t(hiactivedat_af[,names(hiactivedat_af) %in% cowlist_healthy_sensor])
d <- dist(dat)

h.hiactaf <- hclust(d,  method ='ward.D2') 
plot(h.hiactaf, xlab = 'Cow ID', main = 'Dendrogram of Afternoon Time High Activity Data')
```

```
# morning data

dat <- t(hiactivedat_morning[,names(hiactivedat_morning) %in% cowlist_healthy_sensor])
d <- dist(dat)

h.hiactam <- hclust(d,  method ='ward.D2') 
plot(h.hiactam, xlab = 'Cow ID',  main = 'Dendrogram of Morning Time High Activity Data')
```

```
# all data w/out milking

dat <- t(hiactivedat_morning[,names(hiactivedat_morning) %in% cowlist_healthy_sensor])
dat <- cbind(dat, 
             t(hiactivedat_af[,names(hiactivedat_af) %in% cowlist_healthy_sensor]))
dat <- cbind(dat, 
             t(hiactivedat_night[,names(hiactivedat_night) %in% cowlist_healthy_sensor]))

d <- dist(dat)
h.hiactl<- hclust(d, method ='ward.D2') 
plot(h.hiactl, xlab = 'Cow ID', main = 'Dendrogram of Lounging Period High Activity Data')
```

```
# Comparison to Qeueu to all high activity data

btreeout_queueall_hiact <- bivartree(h.dist.all, h.hiact, xrange = 2:10, yrange = 2:10)
btreeout_queueall_hiact$SignificanceMatrix
```

```
##         k=2    k=3    k=4    k=5    k=6    k=7    k=8    k=9   k=10
## k=2  0.0310 0.0810 0.1000 0.0995 0.1150 0.0870 0.1175 0.0520 0.0260
## k=3  0.0765 0.1350 0.2275 0.2685 0.3675 0.1245 0.1730 0.1070 0.0915
## k=4  0.0685 0.0870 0.1490 0.2555 0.3780 0.1780 0.2095 0.1150 0.1130
## k=5  0.0555 0.1005 0.1620 0.2410 0.3305 0.1935 0.2400 0.1565 0.2045
## k=6  0.0765 0.0560 0.1280 0.1415 0.2120 0.1315 0.1450 0.1530 0.1645
## k=7  0.1325 0.1360 0.2430 0.1300 0.2410 0.1560 0.2045 0.2165 0.2630
## k=8  0.1360 0.0930 0.2020 0.0565 0.0990 0.0530 0.0875 0.0450 0.0370
## k=9  0.1180 0.1075 0.2250 0.0610 0.1065 0.0805 0.0810 0.0605 0.0380
## k=10 0.1835 0.1080 0.1390 0.0365 0.0190 0.0100 0.0180 0.0165 0.0105
```

```
kable(round(btreeout_queueall_hiact$SignificanceMatrix,3), format = "html", caption = 'High Activity Overall') %>% cat(., file = "Viz/BivariateAnalyses/MCETest/HighActivity/Overall.html")

# Comparison to Qeueu to night high activity data

btreeout_queueall_hiactn <- bivartree(h.dist.all, h.hiactn, xrange = 2:10, yrange = 2:10)
btreeout_queueall_hiactn$SignificanceMatrix
```

```
##         k=2    k=3    k=4    k=5    k=6    k=7    k=8    k=9   k=10
## k=2  0.0000 0.0270 0.0140 0.0330 0.0780 0.1355 0.2145 0.2235 0.1630
## k=3  0.1745 0.3365 0.1400 0.1935 0.3740 0.5735 0.7665 0.2710 0.2720
## k=4  0.6920 0.2070 0.1520 0.2550 0.4550 0.3460 0.6295 0.2575 0.3050
## k=5  0.3155 0.2445 0.0555 0.2680 0.3880 0.2940 0.4850 0.2105 0.1995
## k=6  0.3430 0.4530 0.1495 0.3165 0.4330 0.2855 0.3410 0.1680 0.1485
## k=7  0.4035 0.5240 0.2325 0.4600 0.6495 0.5135 0.5485 0.3640 0.3640
## k=8  0.4610 0.6740 0.4120 0.4695 0.6815 0.5930 0.6895 0.5050 0.5225
## k=9  0.6430 0.7910 0.6035 0.4560 0.5870 0.5685 0.7135 0.5680 0.6040
## k=10 0.0820 0.5080 0.3280 0.3845 0.5810 0.5540 0.7095 0.5850 0.6570
```

```
btreeout_queueall_hiactn$Results[[2]][[2]]
```

```
## $ContingencyTable
##    
##      1  2
##   1 75  0
##   2 33  1
## 
## $ObserveMCE
##         1 
## 0.4697714 
## 
## $PVal
## [1] 0
```

```
btreeout_queueall_hiactn$Results[[2]][[4]]
```

```
## $ContingencyTable
##    
##      1  2  3  4
##   1 72  3  0  0
##   2 31  0  2  1
## 
## $ObserveMCE
##         1 
## 0.5800937 
## 
## $PVal
## [1] 0.014
```

```
kable(round(btreeout_queueall_hiactn$SignificanceMatrix,3), format = "html", caption = 'High Activity Night') %>% cat(., file = "Viz/BivariateAnalyses/MCETest/HighActivity/Night.html")


# Comparison to Qeueu to afternoon high activity data

btreeout_queueall_hiactaf <- bivartree(h.dist.all, h.hiactaf, xrange = 2:10, yrange = 2:10)
btreeout_queueall_hiactaf$SignificanceMatrix
```

```
##         k=2    k=3    k=4    k=5    k=6    k=7    k=8    k=9   k=10
## k=2  0.5325 0.8505 0.6995 0.6150 0.7125 0.7685 0.6450 0.2020 0.1280
## k=3  0.8030 0.4230 0.3060 0.3610 0.5550 0.6825 0.4790 0.2455 0.1890
## k=4  0.9095 0.5040 0.3640 0.4710 0.7095 0.7610 0.6375 0.4505 0.4040
## k=5  0.9710 0.4825 0.2175 0.3365 0.5735 0.7050 0.6740 0.5635 0.5180
## k=6  0.9550 0.6720 0.3665 0.5020 0.7100 0.7175 0.7250 0.6320 0.5815
## k=7  0.9035 0.7035 0.5080 0.6415 0.8425 0.8630 0.6680 0.6155 0.5740
## k=8  0.9445 0.8220 0.6560 0.7890 0.9125 0.9280 0.7700 0.7145 0.6945
## k=9  0.9150 0.8645 0.7015 0.8295 0.8290 0.9090 0.7735 0.7775 0.7505
## k=10 0.9420 0.3680 0.3240 0.2650 0.2660 0.5370 0.3875 0.3960 0.3980
```

```
kable(round(btreeout_queueall_hiactaf$SignificanceMatrix,3), format = "html", caption = 'High Activity Afternoon') %>% cat(., file = "Viz/BivariateAnalyses/MCETest/HighActivity/Afternoon.html")


# Comparison to Qeueu to morning high activity data

btreeout_queueall_hiactam <- bivartree(h.dist.all, h.hiactam, xrange = 2:10, yrange = 2:10)
btreeout_queueall_hiactam$SignificanceMatrix
```

```
##         k=2    k=3    k=4    k=5    k=6    k=7    k=8    k=9   k=10
## k=2  0.2305 0.5050 0.6200 0.8105 0.7520 0.3105 0.1700 0.0600 0.1050
## k=3  0.4465 0.6015 0.7840 0.7565 0.7860 0.5360 0.4320 0.2920 0.3940
## k=4  0.5230 0.2745 0.5570 0.6380 0.7410 0.2485 0.1900 0.1365 0.1750
## k=5  0.3220 0.1920 0.4365 0.5185 0.6490 0.2730 0.2570 0.1775 0.2620
## k=6  0.3090 0.2405 0.3895 0.5275 0.6095 0.3260 0.2965 0.2350 0.3140
## k=7  0.4605 0.3860 0.3505 0.4875 0.5955 0.3345 0.2940 0.2360 0.3210
## k=8  0.3660 0.3690 0.4035 0.5610 0.6750 0.4530 0.4200 0.3850 0.3075
## k=9  0.2470 0.3145 0.3330 0.5080 0.6240 0.4265 0.4330 0.3775 0.3125
## k=10 0.1795 0.1175 0.2150 0.3180 0.4425 0.3040 0.2945 0.2720 0.2425
```

```
kable(round(btreeout_queueall_hiactam$SignificanceMatrix,3), format = "html", caption = 'High Activity Morning') %>% cat(., file = "Viz/BivariateAnalyses/MCETest/HighActivity/Morning.html")

# Comparison of Queue to lounging high activity data

btreeout_queueall_hiactl <- bivartree(h.dist.all, h.hiactl, xrange = 2:10, yrange = 2:10)
kable(round(btreeout_queueall_hiactl$SignificanceMatrix,3), format = "html", caption = 'High Activity Lounging') %>% cat(., file = "Viz/BivariateAnalyses/MCETest/HighActivity/Lounging.html")
btreeout_queueall_hiactl$SignificanceMatrix
```

```
##         k=2    k=3    k=4    k=5    k=6    k=7    k=8    k=9   k=10
## k=2  0.0285 0.1340 0.1640 0.1785 0.2090 0.1885 0.1655 0.2055 0.3075
## k=3  0.2275 0.3835 0.5050 0.6265 0.6250 0.6305 0.2665 0.3170 0.4780
## k=4  0.0765 0.2185 0.3680 0.5390 0.6360 0.6810 0.4205 0.5610 0.8040
## k=5  0.0550 0.2705 0.4195 0.5675 0.7410 0.8190 0.6225 0.7820 0.9235
## k=6  0.0725 0.3380 0.5115 0.6505 0.6040 0.6960 0.5130 0.7260 0.8885
## k=7  0.1145 0.5060 0.6665 0.8140 0.7985 0.8745 0.7700 0.8150 0.9375
## k=8  0.1825 0.6015 0.7755 0.8815 0.9010 0.9025 0.8425 0.8710 0.8880
## k=9  0.0920 0.4370 0.5940 0.7510 0.8170 0.8615 0.7640 0.8325 0.8770
## k=10 0.1350 0.5110 0.5565 0.4285 0.3325 0.3725 0.3075 0.4470 0.5330
```

```
#p.out.hiact.all <- btreeout_queueall_hiact$SignificanceMatrix[3,2]
p.out.hiact.all <- getBVTPval(btreeout_queueall_hiact$SignificanceMatrix)
p.out.hiact.all
```

```
## $pval
## [1] 0.052
## 
## $row
## k=2 
##   1 
## 
## $col
## k=9 
##   8
```

```
#p.out.hiact.l <- btreeout_queueall_hiactl$SignificanceMatrix[3,2]
p.out.hiact.l <- getBVTPval(btreeout_queueall_hiactl$SignificanceMatrix)
p.out.hiact.l
```

```
## $pval
## [1] 0.0285
## 
## $row
## k=2 
##   1 
## 
## $col
## k=2 
##   1
```

```
#p.out.hiact.am <- btreeout_queueall_hiactam$SignificanceMatrix[3,3]
p.out.hiact.am <- getBVTPval(btreeout_queueall_hiactam$SignificanceMatrix)
p.out.hiact.am
```

```
## $pval
## [1] 0.272
## 
## $row
## k=10 
##    9 
## 
## $col
## k=9 
##   8
```

```
#p.out.hiact.af <- btreeout_queueall_hiactaf$SignificanceMatrix[3,3]
p.out.hiact.af <- getBVTPval(btreeout_queueall_hiactaf$SignificanceMatrix)
p.out.hiact.af
```

```
## $pval
## [1] 0.306
## 
## $row
## k=3 
##   2 
## 
## $col
## k=4 
##   3
```

```
#p.out.hiact.n <- btreeout_queueall_hiactn$SignificanceMatrix[3,5]
p.out.hiact.n <- getBVTPval(btreeout_queueall_hiactn$SignificanceMatrix)
p.out.hiact.n
```

```
## $pval
## [1] 0.014
## 
## $row
## k=2 
##   1 
## 
## $col
## k=4 
##   3
```

### Rumenation

```
set.seed(161916)

# all data

dat <- t(rumdat[,names(rumdat) %in% cowlist_healthy_sensor])
d <- dist(dat)

h.rum <- hclust(d,  method ='ward.D2') 
plot(h.rum, xlab = 'Cow ID',  main = 'Dendrogram of Rumination Data')
```

```
# night time data

dat <- t(rumdat_night[,names(rumdat_night) %in% cowlist_healthy_sensor])
d <- dist(dat)

h.rumn <- hclust(d,  method ='ward.D2') 
plot(h.rumn, xlab = 'Cow ID', main = 'Dendrogram of Night Time Rumination Data')
```

```
# evening data

dat <- t(rumdat_af[,names(rumdat_af) %in% cowlist_healthy_sensor])
d <- dist(dat)

h.rumaf <- hclust(d,  method ='ward.D2') 
plot(h.rumaf, xlab = 'Cow ID',  main = 'Dendrogram of Afternoon Time Rumination Data')
```

```
# morning data


dat <- t(rumdat_morning[,names(rumdat_morning) %in% cowlist_healthy_sensor])
d <- dist(dat)

h.rumam <- hclust(d,  method ='ward.D2') 
plot(h.rumam, xlab = 'Cow ID', main = 'Dendrogram of Morning Time Rumination Data')
```

```
# all data w/out milking

dat <- t(rumdat_morning[,names(rumdat_morning) %in% cowlist_healthy_sensor])
dat <- cbind(dat, 
             t(rumdat_af[,names(rumdat_af) %in% cowlist_healthy_sensor]))
dat <- cbind(dat, 
             t(rumdat_night[,names(rumdat_night) %in% cowlist_healthy_sensor]))

d <- dist(dat)
h.ruml<- hclust(d, method ='ward.D2') 
plot(h.ruml, xlab = 'Cow ID', main = 'Dendrogram of Lounging Period Rumination Data')
```

```
# Comparison to Qeueu to all high activity data

btreeout_queueall_rum <- bivartree(h.dist.all, h.rum, xrange = 2:10, yrange = 2:10)
btreeout_queueall_rum$SignificanceMatrix
```

```
##         k=2    k=3    k=4    k=5    k=6    k=7    k=8    k=9   k=10
## k=2  0.8220 0.6260 0.0210 0.0300 0.0430 0.0830 0.0550 0.0945 0.0955
## k=3  0.8550 0.6740 0.0650 0.1000 0.1365 0.0740 0.1005 0.1600 0.1810
## k=4  0.9155 0.8550 0.1760 0.2055 0.2930 0.2595 0.1155 0.1420 0.1210
## k=5  0.9655 0.8310 0.1730 0.2715 0.3855 0.2495 0.1545 0.1520 0.1645
## k=6  0.9170 0.7980 0.2055 0.3545 0.4920 0.3485 0.2760 0.2250 0.2495
## k=7  0.9360 0.9125 0.2710 0.4525 0.6710 0.5085 0.3265 0.2945 0.3270
## k=8  0.6600 0.7000 0.1380 0.3400 0.4250 0.3710 0.2415 0.1675 0.1915
## k=9  0.7185 0.8110 0.2550 0.3600 0.3580 0.2210 0.1560 0.1025 0.1290
## k=10 0.7935 0.8725 0.1905 0.3115 0.3580 0.2410 0.1975 0.1625 0.2010
```

```
kable(round(btreeout_queueall_rum$SignificanceMatrix,3), format = "html", caption = 'Rumination Overall') %>% cat(., file = "Viz/BivariateAnalyses/MCETest/Rumination/Overall.html")

# Comparison to Qeueu to night high activity data

btreeout_queueall_rumn <- bivartree(h.dist.all, h.rumn, xrange = 2:10, yrange = 2:10)
btreeout_queueall_rumn$SignificanceMatrix
```

```
##         k=2    k=3    k=4    k=5    k=6    k=7    k=8    k=9   k=10
## k=2  0.4595 0.5690 0.7260 0.8570 0.7465 0.7335 0.6405 0.5440 0.5405
## k=3  0.8290 0.7515 0.8995 0.9290 0.8400 0.8875 0.8860 0.8755 0.8355
## k=4  0.9445 0.9180 0.6735 0.8305 0.5210 0.7090 0.7625 0.7895 0.6920
## k=5  0.9895 0.9775 0.8265 0.8860 0.5550 0.6755 0.6865 0.6605 0.4805
## k=6  0.9945 0.9535 0.8150 0.4525 0.2165 0.3475 0.4220 0.3980 0.2690
## k=7  0.9810 0.8665 0.6960 0.3775 0.2035 0.3725 0.4750 0.4445 0.3505
## k=8  0.9725 0.7115 0.5570 0.2965 0.1970 0.3575 0.4915 0.5370 0.1520
## k=9  0.9900 0.8370 0.7500 0.5245 0.3135 0.5285 0.6995 0.7140 0.3400
## k=10 0.9915 0.9015 0.8415 0.7095 0.4190 0.6740 0.7905 0.7530 0.3485
```

```
kable(round(btreeout_queueall_rumn$SignificanceMatrix,3), format = "html", caption = 'Rumination Night') %>% cat(., file = "Viz/BivariateAnalyses/MCETest/Rumination/Night.html")


# Comparison to Qeueu to afternoon high activity data

btreeout_queueall_rumaf <- bivartree(h.dist.all, h.rumaf, xrange = 2:10, yrange = 2:10)
btreeout_queueall_rumaf$SignificanceMatrix
```

```
##         k=2    k=3    k=4    k=5    k=6    k=7    k=8    k=9   k=10
## k=2  0.5005 0.4610 0.3305 0.4725 0.6185 0.6680 0.7755 0.8935 0.8970
## k=3  0.8425 0.1090 0.1630 0.2675 0.4500 0.5940 0.7330 0.8535 0.8720
## k=4  0.8460 0.0660 0.1425 0.1950 0.2110 0.2280 0.3615 0.4315 0.5235
## k=5  0.9230 0.1440 0.2865 0.3110 0.2265 0.2665 0.3595 0.4220 0.5090
## k=6  0.3000 0.0520 0.1365 0.1615 0.1305 0.1735 0.2565 0.3135 0.3820
## k=7  0.4015 0.0710 0.2220 0.2845 0.1140 0.1100 0.1960 0.1330 0.2055
## k=8  0.3235 0.0925 0.2105 0.3280 0.1190 0.1305 0.2025 0.1680 0.1950
## k=9  0.2075 0.0690 0.1900 0.3405 0.0665 0.0895 0.1470 0.1160 0.1945
## k=10 0.2830 0.0830 0.1460 0.2680 0.0750 0.0820 0.1450 0.1280 0.1975
```

```
kable(round(btreeout_queueall_rumaf$SignificanceMatrix,3), format = "html", caption = 'Rumination Afternoon') %>% cat(., file = "Viz/BivariateAnalyses/MCETest/Rumination/Afternoon.html")

# Comparison to Qeueu to afternoon high activity data

btreeout_queueall_rumam <- bivartree(h.dist.all, h.rumam, xrange = 2:10, yrange = 2:10)
btreeout_queueall_rumam$SignificanceMatrix
```

```
##         k=2    k=3    k=4    k=5    k=6    k=7    k=8    k=9   k=10
## k=2  0.3715 0.7415 0.9000 0.8065 0.8935 0.8970 0.9485 0.9250 0.9155
## k=3  0.0890 0.2490 0.5045 0.6115 0.7875 0.5095 0.6700 0.4405 0.4230
## k=4  0.1625 0.3895 0.6370 0.7805 0.9080 0.7920 0.8170 0.7215 0.6500
## k=5  0.2365 0.5695 0.8215 0.8580 0.9600 0.9315 0.8610 0.8125 0.8060
## k=6  0.2290 0.3270 0.5360 0.2360 0.5115 0.5335 0.5205 0.4520 0.4735
## k=7  0.3250 0.2505 0.4980 0.1665 0.2740 0.2915 0.3425 0.3090 0.2905
## k=8  0.4340 0.4295 0.5825 0.2795 0.4665 0.5140 0.5295 0.5210 0.5095
## k=9  0.4175 0.4285 0.5660 0.3270 0.4815 0.3570 0.3410 0.3760 0.3920
## k=10 0.3595 0.4450 0.5785 0.3900 0.4265 0.2010 0.2210 0.2140 0.2325
```

```
kable(round(btreeout_queueall_rumam$SignificanceMatrix,3), format = "html", caption = 'Rumination Morning') %>% cat(., file = "Viz/BivariateAnalyses/MCETest/Rumination/Morning.html")

# Comparison of Queue to lounging rumination data

btreeout_queueall_ruml <- bivartree(h.dist.all, h.ruml, xrange = 2:10, yrange = 2:10)
kable(round(btreeout_queueall_ruml$SignificanceMatrix,3), format = "html", caption = 'Rumination Lounging') %>% cat(., file = "Viz/BivariateAnalyses/MCETest/Rumination/Lounging.html")
btreeout_queueall_ruml$SignificanceMatrix
```

```
##         k=2    k=3    k=4    k=5    k=6    k=7    k=8    k=9   k=10
## k=2  0.8240 0.3615 0.3885 0.3655 0.5130 0.5140 0.6200 0.7235 0.7580
## k=3  0.4410 0.1270 0.1565 0.1310 0.2165 0.3010 0.4760 0.6085 0.5765
## k=4  0.6110 0.1725 0.2495 0.1360 0.1880 0.2600 0.4650 0.6380 0.6885
## k=5  0.7690 0.3185 0.4840 0.2365 0.3585 0.4690 0.5100 0.5805 0.5795
## k=6  0.2175 0.1200 0.2310 0.0985 0.2330 0.3355 0.3595 0.3995 0.3965
## k=7  0.3250 0.0640 0.1735 0.1035 0.1910 0.3515 0.3005 0.3515 0.4225
## k=8  0.2910 0.0700 0.1810 0.0925 0.1680 0.1745 0.1415 0.1860 0.2435
## k=9  0.1825 0.0500 0.1580 0.0920 0.1805 0.1405 0.1430 0.1490 0.2255
## k=10 0.2535 0.0310 0.1145 0.0860 0.2045 0.1485 0.1505 0.1940 0.2560
```

```
#p.out.rum.all <- btreeout_queueall_rum$SignificanceMatrix[3,5]
p.out.rum.all <- getBVTPval(btreeout_queueall_rum$SignificanceMatrix)
p.out.rum.all
```

```
## $pval
## [1] 0.021
## 
## $row
## k=2 
##   1 
## 
## $col
## k=4 
##   3
```

```
#p.out.rum.l <- btreeout_queueall_ruml$SignificanceMatrix[3,3]
p.out.rum.l <- getBVTPval(btreeout_queueall_ruml$SignificanceMatrix)
p.out.rum.l
```

```
## $pval
## [1] 0.05
## 
## $row
## k=9 
##   8 
## 
## $col
## k=3 
##   2
```

```
#p.out.rum.am <- btreeout_queueall_rumam$SignificanceMatrix[3,2]
p.out.rum.am <- getBVTPval(btreeout_queueall_rumam$SignificanceMatrix)
p.out.rum.am
```

```
## $pval
## [1] 0.325
## 
## $row
## k=7 
##   6 
## 
## $col
## k=2 
##   1
```

```
#p.out.rum.af <- btreeout_queueall_rumaf$SignificanceMatrix[3,3]
p.out.rum.af <- getBVTPval(btreeout_queueall_rumaf$SignificanceMatrix)
p.out.rum.af
```

```
## $pval
## [1] 0.083
## 
## $row
## k=10 
##    9 
## 
## $col
## k=3 
##   2
```

```
#p.out.rum.n <- btreeout_queueall_rumn$SignificanceMatrix[3,2]
p.out.rum.n <- getBVTPval(btreeout_queueall_rumn$SignificanceMatrix)
p.out.rum.n
```

```
## $pval
## [1] 0.152
## 
## $row
## k=8 
##   7 
## 
## $col
## k=10 
##    9
```

### Eating

```
(161916)
```

```
## [1] 161916
```

```
# all data

dat <- t(eatdat[,names(eatdat) %in% cowlist_healthy_sensor])
d <- dist(dat)

h.eat <- hclust(d,  method ='ward.D2') 
plot(h.eat, xlab = 'Cow ID',  main = 'Dendrogram of Eating Data')
```

```
# night time data

dat <- t(eatdat_night[,names(eatdat_night) %in% cowlist_healthy_sensor])
d <- dist(dat)

h.eatn <- hclust(d,  method ='ward.D2') 
plot(h.eatn, xlab = 'Cow ID',  main = 'Dendrogram of Night Time Eating Data')
```

```
# morning data

dat <- t(eatdat_morning[,names(eatdat_morning) %in% cowlist_healthy_sensor])
d <- dist(dat)

h.eatam <- hclust(d,  method ='ward.D2') 
plot(h.eatam, xlab = 'Cow ID',  main = 'Dendrogram of Morning Time Eating Data')
```

```
# evening data

dat <- t(eatdat_af[,names(eatdat_af) %in% cowlist_healthy_sensor])
d <- dist(dat)

h.eataf <- hclust(d,  method ='ward.D2') 
plot(h.eataf, xlab = 'Cow ID', main = 'Dendrogram of Afternoon Time Eating Data')
```

```
# all data w/out milking

dat <- t(eatdat_morning[,names(eatdat_morning) %in% cowlist_healthy_sensor])
dat <- cbind(dat, 
             t(eatdat_af[,names(eatdat_af) %in% cowlist_healthy_sensor]))
dat <- cbind(dat, 
             t(eatdat_night[,names(eatdat_night) %in% cowlist_healthy_sensor]))

d <- dist(dat)
h.eatl<- hclust(d, method ='ward.D2') 
plot(h.eatl, xlab = 'Cow ID', main = 'Dendrogram of Lounging Period Eating Data')
```

```
# Comparison to Qeueu to all eating data

btreeout_queueall_eat <- bivartree(h.dist.all, h.eat, xrange = 2:10, yrange = 2:10)
btreeout_queueall_eat$SignificanceMatrix
```

```
##         k=2    k=3    k=4    k=5    k=6    k=7    k=8    k=9   k=10
## k=2  0.8095 0.6020 0.0370 0.0225 0.0010 0.0025 0.0050 0.0125 0.0195
## k=3  0.5675 0.6670 0.0660 0.0670 0.0045 0.0055 0.0130 0.0285 0.0475
## k=4  0.6040 0.7355 0.1230 0.1745 0.0075 0.0120 0.0355 0.0510 0.0820
## k=5  0.2385 0.5045 0.0375 0.0965 0.0075 0.0115 0.0175 0.0450 0.0640
## k=6  0.3725 0.3595 0.0360 0.0140 0.0000 0.0045 0.0035 0.0030 0.0105
## k=7  0.3865 0.4425 0.0190 0.0135 0.0035 0.0040 0.0095 0.0015 0.0050
## k=8  0.4345 0.5140 0.0265 0.0310 0.0060 0.0125 0.0230 0.0055 0.0110
## k=9  0.5530 0.4725 0.0265 0.0330 0.0075 0.0070 0.0220 0.0080 0.0165
## k=10 0.5940 0.5770 0.0400 0.0505 0.0075 0.0145 0.0415 0.0190 0.0415
```

```
btreeout_queueall_eat$Results[[4]][[7]]
```

```
## $ContingencyTable
##    
##      1  2  3  4  5  6  7
##   1  8  7  6  2  2  2  2
##   2  7  6  0  2  2  2  0
##   3 10  4  1  1  4  2  5
##   4  3  8  8  6  2  7  0
## 
## $ObserveMCE
##        1 
## 2.060314 
## 
## $PVal
## [1] 0.012
```

```
kable(round(btreeout_queueall_eat$SignificanceMatrix,3), format = "html", caption = 'Eating Overall') %>% cat(., file = "Viz/BivariateAnalyses/MCETest/Eating/Overall.html")


# Comparison to Qeueu to night eating data

btreeout_queueall_eatn <- bivartree(h.dist.all, h.eatn, xrange = 2:10, yrange = 2:10)
btreeout_queueall_eatn$SignificanceMatrix
```

```
##         k=2    k=3    k=4    k=5    k=6    k=7    k=8    k=9   k=10
## k=2  0.3610 0.7075 0.8805 0.9400 0.9680 0.9790 0.9890 0.7865 0.7860
## k=3  0.2695 0.6000 0.7965 0.9070 0.8860 0.7575 0.8995 0.7555 0.7850
## k=4  0.3680 0.7545 0.9195 0.8370 0.8880 0.8500 0.9695 0.9335 0.9135
## k=5  0.5415 0.6975 0.7565 0.7095 0.7435 0.7260 0.8815 0.8270 0.8575
## k=6  0.4940 0.5985 0.5810 0.5265 0.5990 0.6145 0.8025 0.6985 0.7185
## k=7  0.5900 0.4855 0.5585 0.5300 0.6395 0.6810 0.7880 0.7360 0.6810
## k=8  0.7100 0.4755 0.6170 0.6325 0.7525 0.7765 0.8905 0.8575 0.8060
## k=9  0.2125 0.2420 0.4125 0.4395 0.6045 0.6370 0.8290 0.8015 0.8000
## k=10 0.2975 0.2280 0.4605 0.4980 0.6875 0.7340 0.9175 0.8760 0.8685
```

```
kable(round(btreeout_queueall_eatn$SignificanceMatrix,3), format = "html", caption = 'Eating Night') %>% cat(., file = "Viz/BivariateAnalyses/MCETest/Eating/Night.html")


# Comparison to Qeueu to afternoon eating data

btreeout_queueall_eataf <- bivartree(h.dist.all, h.eataf, xrange = 2:10, yrange = 2:10)
btreeout_queueall_eataf$SignificanceMatrix
```

```
##         k=2    k=3    k=4    k=5    k=6    k=7    k=8    k=9   k=10
## k=2  0.3880 0.7145 0.0655 0.0980 0.0680 0.1090 0.1435 0.1770 0.2445
## k=3  0.7075 0.9660 0.1370 0.2980 0.1980 0.1715 0.1650 0.1970 0.3005
## k=4  0.8270 0.9565 0.1135 0.2290 0.2295 0.2240 0.2730 0.2815 0.4225
## k=5  0.8875 0.9795 0.2545 0.2115 0.1720 0.2260 0.2655 0.3215 0.3820
## k=6  0.4790 0.6960 0.1170 0.1035 0.1145 0.1435 0.1985 0.2670 0.2400
## k=7  0.5545 0.8480 0.1520 0.1835 0.1605 0.1940 0.3040 0.4140 0.3860
## k=8  0.6200 0.7960 0.1795 0.1820 0.1930 0.2345 0.3905 0.4950 0.4315
## k=9  0.6950 0.9035 0.3270 0.2565 0.2100 0.3025 0.4805 0.5690 0.5680
## k=10 0.7610 0.9145 0.2085 0.1900 0.1565 0.2895 0.4965 0.6275 0.6430
```

```
kable(round(btreeout_queueall_eataf$SignificanceMatrix,3), format = "html", caption = 'Eating Afternoon') %>% cat(., file = "Viz/BivariateAnalyses/MCETest/Eating/Afternoon.html")


# Comparison to Qeueu to morning eating data

btreeout_queueall_eatam <- bivartree(h.dist.all, h.eatam, xrange = 2:10, yrange = 2:10)
btreeout_queueall_eatam$SignificanceMatrix
```

```
##         k=2    k=3    k=4    k=5    k=6    k=7    k=8    k=9   k=10
## k=2  0.5145 0.5340 0.7120 0.7985 0.8945 0.9245 0.9400 0.9565 0.2475
## k=3  0.7025 0.7775 0.5050 0.6080 0.4655 0.5200 0.6465 0.6755 0.2710
## k=4  0.6330 0.8430 0.4700 0.5240 0.3955 0.5365 0.6305 0.7100 0.3950
## k=5  0.6220 0.3930 0.3065 0.3570 0.3535 0.3750 0.5530 0.6270 0.3945
## k=6  0.3890 0.3095 0.1885 0.2255 0.2810 0.3615 0.5940 0.6815 0.4820
## k=7  0.4710 0.3155 0.2475 0.3135 0.3465 0.3970 0.6655 0.7560 0.6075
## k=8  0.5795 0.4675 0.3290 0.4210 0.5075 0.5300 0.7790 0.8345 0.7015
## k=9  0.5705 0.4795 0.3385 0.4030 0.5700 0.4455 0.7440 0.8340 0.7155
## k=10 0.6930 0.6080 0.4485 0.5370 0.6910 0.6320 0.8400 0.8945 0.8310
```

```
kable(round(btreeout_queueall_eatam$SignificanceMatrix,3), format = "html", caption = 'Eating Morning') %>% cat(., file = "Viz/BivariateAnalyses/MCETest/Eating/Morning.html")

# Comparison of Queue to lounging rumination data

btreeout_queueall_eatl <- bivartree(h.dist.all, h.eatl, xrange = 2:10, yrange = 2:10)
kable(round(btreeout_queueall_eatl$SignificanceMatrix,3), format = "html", caption = 'Eating Lounging') %>% cat(., file = "Viz/BivariateAnalyses/MCETest/Eating/Lounging.html")
btreeout_queueall_eatl$SignificanceMatrix
```

```
##         k=2    k=3    k=4    k=5    k=6    k=7    k=8    k=9   k=10
## k=2  0.5390 0.7690 0.7680 0.3065 0.4250 0.4850 0.6110 0.7455 0.4585
## k=3  0.8990 0.9685 0.9795 0.8060 0.8290 0.8510 0.9205 0.9720 0.7390
## k=4  0.5480 0.7475 0.8765 0.4225 0.5355 0.5700 0.6365 0.7885 0.5555
## k=5  0.5130 0.7800 0.7315 0.3465 0.5185 0.5690 0.6700 0.7245 0.5525
## k=6  0.1125 0.4485 0.5055 0.2760 0.4270 0.5295 0.6575 0.6925 0.5555
## k=7  0.0910 0.4405 0.4000 0.2340 0.4185 0.3365 0.4635 0.4890 0.3750
## k=8  0.1350 0.5360 0.5030 0.3640 0.6185 0.5580 0.6610 0.6920 0.6245
## k=9  0.0725 0.4000 0.4020 0.3280 0.5975 0.5145 0.6530 0.7010 0.6015
## k=10 0.1125 0.4850 0.5475 0.3280 0.6030 0.5890 0.7080 0.7620 0.6960
```

```
#p.out.eat.all <- btreeout_queueall_eat$SignificanceMatrix[3,7]
p.out.eat.all <- getBVTPval(btreeout_queueall_eat$SignificanceMatrix)
p.out.eat.all
```

```
## $pval
## [1] 0.0035
## 
## $row
## k=7 
##   6 
## 
## $col
## k=6 
##   5
```

```
#p.out.eat.l <- btreeout_queueall_eatl$SignificanceMatrix[3,5]
p.out.eat.l <- getBVTPval(btreeout_queueall_eatl$SignificanceMatrix)
p.out.eat.l
```

```
## $pval
## [1] 0.234
## 
## $row
## k=7 
##   6 
## 
## $col
## k=5 
##   4
```

```
#p.out.eat.am <- btreeout_queueall_eatam$SignificanceMatrix[3,3]
p.out.eat.am <- getBVTPval(btreeout_queueall_eatam$SignificanceMatrix)
p.out.eat.am
```

```
## $pval
## [1] 0.1885
## 
## $row
## k=6 
##   5 
## 
## $col
## k=4 
##   3
```

```
#p.out.eat.af <- btreeout_queueall_eataf$SignificanceMatrix[3,6]
p.out.eat.af <- getBVTPval(btreeout_queueall_eataf$SignificanceMatrix)
p.out.eat.af
```

```
## $pval
## [1] 0.0655
## 
## $row
## k=2 
##   1 
## 
## $col
## k=4 
##   3
```

```
#p.out.eat.n <- btreeout_queueall_eatn$SignificanceMatrix[3,3]
p.out.eat.n <- getBVTPval(btreeout_queueall_eatn$SignificanceMatrix)
p.out.eat.n
```

```
## $pval
## [1] 0.2125
## 
## $row
## k=9 
##   8 
## 
## $col
## k=2 
##   1
```

### Temperature

```
set.seed(161916)

# all data

dat <- t(temperdat[,names(temperdat) %in% cowlist_healthy_sensor])
d <- dist(dat)

h.temp <- hclust(d,  method ='ward.D2') 
plot(h.temp, xlab = 'Cow ID',  main = 'Dendrogram of Temperature Data')
```

```
# night time data

dat <- t(temperdat_night[,names(temperdat_night) %in% cowlist_healthy_sensor])
d <- dist(dat)

h.tempn <- hclust(d,  method ='ward.D2') 
plot(h.tempn, xlab = 'Cow ID', main = 'Dendrogram of Night Temperature Data')
```

```
# evening data

dat <- t(temperdat_af[,names(temperdat_af) %in% cowlist_healthy_sensor])
d <- dist(dat)

h.tempaf <- hclust(d,  method ='ward.D2') 
plot(h.tempaf, xlab = 'Cow ID',  main = 'Dendrogram of Evening Temperature Data')
```

```
# morning data

dat <- t(temperdat_morning[,names(temperdat_morning) %in% cowlist_healthy_sensor])
d <- dist(dat)

h.tempam <- hclust(d,  method ='ward.D2') 
plot(h.tempam, xlab = 'Cow ID',  main = 'Dendrogram of Morning Temperature Data')
```

```
# all data w/out milking

dat <- t(temperdat_morning[,names(temperdat_morning) %in% cowlist_healthy_sensor])
dat <- cbind(dat, 
             t(temperdat_af[,names(temperdat_af) %in% cowlist_healthy_sensor]))
dat <- cbind(dat, 
             t(temperdat_night[,names(temperdat_night) %in% cowlist_healthy_sensor]))

d <- dist(dat)
h.templ<- hclust(d, method ='ward.D2') 
plot(h.templ, xlab = 'Cow ID', main = 'Dendrogram of Lounging Period Temperature Data')
```

```
# Comparison to Qeueu to all temp data

btreeout_queueall_temp <- bivartree(h.dist.all, h.temp, xrange = 2:10, yrange = 2:10)
btreeout_queueall_temp$SignificanceMatrix
```

```
##         k=2    k=3    k=4    k=5    k=6    k=7    k=8    k=9   k=10
## k=2  0.0270 0.0560 0.1200 0.0250 0.0500 0.0925 0.0750 0.0385 0.0310
## k=3  0.0080 0.0115 0.0025 0.0020 0.0020 0.0050 0.0060 0.0010 0.0020
## k=4  0.0155 0.0265 0.0090 0.0040 0.0110 0.0160 0.0125 0.0105 0.0080
## k=5  0.0035 0.0010 0.0005 0.0015 0.0000 0.0025 0.0015 0.0015 0.0000
## k=6  0.0090 0.0040 0.0020 0.0030 0.0045 0.0055 0.0065 0.0045 0.0030
## k=7  0.0065 0.0005 0.0035 0.0065 0.0065 0.0090 0.0025 0.0025 0.0020
## k=8  0.0090 0.0105 0.0065 0.0050 0.0050 0.0070 0.0045 0.0065 0.0025
## k=9  0.0135 0.0100 0.0105 0.0080 0.0095 0.0175 0.0160 0.0175 0.0070
## k=10 0.0205 0.0325 0.0155 0.0125 0.0090 0.0115 0.0155 0.0260 0.0065
```

```
kable(round(btreeout_queueall_temp$SignificanceMatrix,3), format = "html", caption = 'Temperature Overall') %>% cat(., file = "Viz/BivariateAnalyses/MCETest/Temperature/Overall.html")


# Comparison to Qeueu to night temp data

btreeout_queueall_tempn <- bivartree(h.dist.all, h.tempn, xrange = 2:10, yrange = 2:10)
btreeout_queueall_tempn$SignificanceMatrix
```

```
##         k=2    k=3    k=4    k=5    k=6    k=7    k=8    k=9   k=10
## k=2  0.0545 0.1340 0.2705 0.3360 0.4765 0.6100 0.7115 0.8035 0.8580
## k=3  0.0610 0.0555 0.0465 0.0895 0.1740 0.2775 0.4075 0.4810 0.6205
## k=4  0.1360 0.1055 0.0865 0.0915 0.2390 0.3220 0.3910 0.3925 0.5370
## k=5  0.0225 0.0150 0.0185 0.0185 0.0650 0.0955 0.0910 0.0965 0.1425
## k=6  0.0365 0.0250 0.0365 0.0125 0.0640 0.1165 0.1175 0.1285 0.1975
## k=7  0.0345 0.0090 0.0250 0.0225 0.0870 0.1140 0.1345 0.1295 0.2180
## k=8  0.0430 0.0225 0.0425 0.0230 0.1055 0.1740 0.1650 0.1755 0.2870
## k=9  0.0530 0.0410 0.0705 0.0125 0.0470 0.0800 0.0770 0.0910 0.1560
## k=10 0.0505 0.0520 0.1015 0.0180 0.0895 0.1415 0.1590 0.1775 0.2235
```

```
btreeout_queueall_tempn$Results[[9]][[2]]
```

```
## $ContingencyTable
##    
##      1  2
##   1  6  4
##   2  2  2
##   3  7 12
##   4 10 10
##   5 11  9
##   6  7  3
##   7 13  1
##   8  6  1
##   9  2  3
## 
## $ObserveMCE
##        1 
## 1.870483 
## 
## $PVal
## [1] 0.053
```

```
kable(round(btreeout_queueall_tempn$SignificanceMatrix,3), format = "html", caption = 'Temperature Night') %>% cat(., file = "Viz/BivariateAnalyses/MCETest/Temperature/Night.html")


# Comparison to Qeueu to afternon temp data

btreeout_queueall_tempaf <- bivartree(h.dist.all, h.tempaf, xrange = 2:15, yrange = 2:10)
btreeout_queueall_tempaf$SignificanceMatrix
```

```
##         k=2    k=3    k=4    k=5    k=6    k=7    k=8    k=9   k=10
## k=2  0.1995 0.0990 0.0305 0.0635 0.1000 0.1995 0.1915 0.2780 0.3645
## k=3  0.3985 0.0660 0.0170 0.0055 0.0070 0.0200 0.0280 0.0425 0.0615
## k=4  0.5980 0.1315 0.0575 0.0225 0.0305 0.0450 0.0520 0.1090 0.1235
## k=5  0.3275 0.0325 0.0205 0.0085 0.0170 0.0315 0.0265 0.0805 0.0930
## k=6  0.5080 0.0810 0.0500 0.0245 0.0365 0.0665 0.0740 0.1340 0.1555
## k=7  0.3615 0.0960 0.0820 0.0600 0.0700 0.1120 0.0975 0.1855 0.1720
## k=8  0.4655 0.1785 0.0180 0.0190 0.0295 0.0470 0.0410 0.1130 0.1210
## k=9  0.5780 0.2855 0.0590 0.0420 0.0875 0.1425 0.1335 0.2610 0.2600
## k=10 0.6700 0.4205 0.1095 0.0575 0.1450 0.1840 0.2015 0.3065 0.3085
## k=11 0.7225 0.5495 0.2130 0.1480 0.3330 0.3220 0.2935 0.4520 0.3600
## k=12 0.7130 0.5645 0.2520 0.1660 0.3925 0.3900 0.3615 0.5245 0.4355
## k=13 0.6785 0.5810 0.2465 0.1895 0.4480 0.4490 0.4170 0.4470 0.3215
## k=14 0.7685 0.6785 0.3835 0.2995 0.4925 0.4745 0.4665 0.3590 0.2880
## k=15 0.7915 0.7235 0.4840 0.4125 0.6685 0.5740 0.5485 0.4000 0.3160
```

```
btreeout_queueall_tempaf$Results[[9]][[3]]
```

```
## $ContingencyTable
##    
##      1  2  3
##   1  5  1  4
##   2  1  1  2
##   3 10  0  9
##   4  9  2  9
##   5  5  7  8
##   6  4  2  4
##   7 10  2  2
##   8  5  1  1
##   9  2  1  2
## 
## $ObserveMCE
##        1 
## 2.082645 
## 
## $PVal
## [1] 0.2855
```

```
kable(round(btreeout_queueall_tempaf$SignificanceMatrix,3), format = "html", caption = 'Temperature Afternoon') %>% cat(., file = "Viz/BivariateAnalyses/MCETest/Temperature/Afternoon.html")

# Comparison to Qeueu to morning temp data

btreeout_queueall_tempam <- bivartree(h.dist.all, h.tempam, xrange = 2:10, yrange = 2:10)
btreeout_queueall_tempam$SignificanceMatrix
```

```
##         k=2    k=3    k=4    k=5    k=6    k=7    k=8    k=9   k=10
## k=2  0.1550 0.0395 0.0975 0.1600 0.2730 0.3220 0.4470 0.5555 0.4390
## k=3  0.0260 0.0065 0.0230 0.0275 0.0795 0.1430 0.2645 0.3210 0.2135
## k=4  0.0450 0.0340 0.0735 0.1310 0.2680 0.4235 0.5810 0.2585 0.2100
## k=5  0.0640 0.0250 0.0740 0.1175 0.1865 0.2650 0.3555 0.1315 0.1305
## k=6  0.0875 0.0370 0.1165 0.1720 0.1830 0.3060 0.2965 0.1075 0.0995
## k=7  0.0995 0.0220 0.1085 0.1165 0.1200 0.2550 0.2075 0.0925 0.0810
## k=8  0.1650 0.0485 0.1995 0.2330 0.2160 0.3725 0.3535 0.2015 0.2110
## k=9  0.1170 0.0695 0.1100 0.1240 0.1125 0.1430 0.1760 0.0855 0.0870
## k=10 0.1535 0.0930 0.2260 0.2550 0.2115 0.2845 0.3295 0.1415 0.1635
```

```
kable(round(btreeout_queueall_tempam$SignificanceMatrix,3), format = "html", caption = 'Temperature Morning') %>% cat(., file = "Viz/BivariateAnalyses/MCETest/Temperature/Morning.html")

# Comparison of Queue to lounging temperature data

btreeout_queueall_templ <- bivartree(h.dist.all, h.templ, xrange = 2:10, yrange = 2:10)
kable(round(btreeout_queueall_templ$SignificanceMatrix,3), format = "html", caption = 'Temperature Lounging') %>% cat(., file = "Viz/BivariateAnalyses/MCETest/Temperature/Lounging.html")
btreeout_queueall_templ$SignificanceMatrix
```

```
##         k=2    k=3    k=4    k=5    k=6    k=7    k=8    k=9   k=10
## k=2  0.1345 0.1005 0.2225 0.1245 0.1575 0.2460 0.2890 0.3220 0.2630
## k=3  0.2580 0.0555 0.0105 0.0040 0.0045 0.0175 0.0220 0.0370 0.0165
## k=4  0.4260 0.0710 0.0085 0.0125 0.0100 0.0110 0.0180 0.0325 0.0370
## k=5  0.0570 0.0020 0.0005 0.0040 0.0015 0.0025 0.0060 0.0055 0.0080
## k=6  0.1070 0.0105 0.0015 0.0035 0.0060 0.0120 0.0170 0.0270 0.0220
## k=7  0.0960 0.0060 0.0035 0.0070 0.0090 0.0170 0.0180 0.0230 0.0195
## k=8  0.1675 0.0205 0.0160 0.0085 0.0090 0.0130 0.0155 0.0180 0.0235
## k=9  0.2155 0.0240 0.0160 0.0085 0.0120 0.0125 0.0230 0.0410 0.0385
## k=10 0.2185 0.0265 0.0170 0.0110 0.0120 0.0220 0.0325 0.0250 0.0325
```

```
#p.out.temp.all <- btreeout_queueall_temp$SignificanceMatrix[3,4]
p.out.temp.all <- getBVTPval(btreeout_queueall_temp$SignificanceMatrix)
p.out.temp.all
```

```
## $pval
## [1] 0
## 
## $row
## k=5 
##   4 
## 
## $col
## k=10 
##    9
```

```
#p.out.temp.l <- btreeout_queueall_templ$SignificanceMatrix[3,4]
p.out.temp.l <- getBVTPval(btreeout_queueall_templ$SignificanceMatrix)
p.out.temp.l
```

```
## $pval
## [1] 0.004
## 
## $row
## k=5 
##   4 
## 
## $col
## k=5 
##   4
```

```
#p.out.temp.am <- btreeout_queueall_tempam$SignificanceMatrix[3,3]
p.out.temp.am <- getBVTPval(btreeout_queueall_tempam$SignificanceMatrix)
p.out.temp.am
```

```
## $pval
## [1] 0.022
## 
## $row
## k=7 
##   6 
## 
## $col
## k=3 
##   2
```

```
#p.out.temp.af <- btreeout_queueall_tempaf$SignificanceMatrix[3,4]
p.out.temp.af <- getBVTPval(btreeout_queueall_tempaf$SignificanceMatrix)
p.out.temp.af
```

```
## $pval
## [1] 0.0055
## 
## $row
## k=3 
##   2 
## 
## $col
## k=5 
##   4
```

```
#p.out.temp.n <- btreeout_queueall_tempn$SignificanceMatrix[3,3]
p.out.temp.n <- getBVTPval(btreeout_queueall_tempn$SignificanceMatrix)
p.out.temp.n
```

```
## $pval
## [1] 0.015
## 
## $row
## k=5 
##   4 
## 
## $col
## k=3 
##   2
```

### Summary of Results

```
tempmat <- matrix(NA, ncol = 5, nrow = 6)

# nonactivity


temp <- ifelse(round(p.out.nonact.all$pval,3) == 0,  paste('<0.001', ' ~', p.out.nonact.all$row+1, ',', p.out.nonact.all$col+1,'~', sep = ''), paste(round(p.out.nonact.all$pval,3), ' ~', p.out.nonact.all$row+1, ',', p.out.nonact.all$col+1,'~', sep = ''))
#temp <- paste(round(p.out.nonact.all$pval,3), ' ~', p.out.nonact.all$row+1, ',', p.out.nonact.all$col+1,'~', sep = '')
#temp <- paste('<0.001', ' ~', p.out.nonact.all$row+1, ',', p.out.nonact.all$col+1,'~', sep = '')
tempmat[1,1] <- temp

temp <- paste(round(p.out.nonact.l$pval,3), ' ~', p.out.nonact.l$row+1, ',', p.out.nonact.l$col+1,'~', sep = '')
tempmat[1,2] <- temp

temp <- paste(round(p.out.nonact.am$pval,3), ' ~', p.out.nonact.am$row+1, ',', p.out.nonact.am$col+1,'~', sep = '')
tempmat[1,3] <- temp

temp <- paste(round(p.out.nonact.af$pval,3), ' ~', p.out.nonact.af$row+1, ',', p.out.nonact.af$col+1,'~', sep = '')
tempmat[1,4] <- temp

temp <- paste(round(p.out.nonact.n$pval,3), ' ~', p.out.nonact.n$row+1, ',', p.out.nonact.n$col+1,'~', sep = '')
tempmat[1,5] <- temp

# Activity

#temp <- paste(round(p.out.act.all$pval,3), ' ~', p.out.act.all$row+1, ',', p.out.act.all$col+1,'~', sep = '')
#temp <- paste('<0.001', ' ~', p.out.act.all$row+1, ',', p.out.act.all$col+1,'~', sep = '')
temp <- ifelse(round(p.out.act.all$pval,3) == 0, paste('<0.001', ' ~', p.out.act.all$row+1, ',', p.out.act.all$col+1,'~', sep = ''), paste(round(p.out.act.all$pval,3), ' ~', p.out.act.all$row+1, ',', p.out.act.all$col+1,'~', sep = ''))
tempmat[2,1] <- temp

temp <- paste(round(p.out.act.l$pval,3), ' ~', p.out.act.l$row+1, ',', p.out.act.l$col+1,'~', sep = '')
tempmat[2,2] <- temp

temp <- paste(round(p.out.act.am$pval,3), ' ~', p.out.act.am$row+1, ',', p.out.act.am$col+1,'~', sep = '')
tempmat[2,3] <- temp

#temp <- paste(round(p.out.act.af$pval,3), ' ~', p.out.act.af$row+1, ',', p.out.act.af$col+1,'~', sep = '')
#temp <- paste('<0.001', ' ~', p.out.act.af$row+1, ',', p.out.act.af$col+1,'~', sep = '')
temp <- ifelse(round(p.out.act.af$pval,3) == 0, paste('<0.001', ' ~', p.out.act.af$row+1, ',', p.out.act.af$col+1,'~', sep = ''), paste(round(p.out.act.af$pval,3), ' ~', p.out.act.af$row+1, ',', p.out.act.af$col+1,'~', sep = ''))
tempmat[2,4] <- temp

#temp <- paste(round(p.out.act.n$pval,3), ' ~', p.out.act.n$row+1, ',', p.out.act.n$col+1,'~', sep = '')
#temp <- paste('<0.001', ' ~', p.out.act.n$row+1, ',', p.out.act.n$col+1,'~', sep = '')
temp <- ifelse(round(p.out.act.n$pval,3) == 0, paste('<0.001', ' ~', p.out.act.n$row+1, ',', p.out.act.n$col+1,'~', sep = ''), paste(round(p.out.act.n$pval,3), ' ~', p.out.act.n$row+1, ',', p.out.act.n$col+1,'~', sep = ''))
tempmat[2,5] <- temp

# hiactivity

temp <- paste(round(p.out.hiact.all$pval,3), ' ~', p.out.hiact.all$row+1, ',', p.out.hiact.all$col+1,'~', sep = '')
tempmat[3,1] <- temp

temp <- paste(round(p.out.hiact.l$pval,3), ' ~', p.out.hiact.l$row+1, ',', p.out.hiact.l$col+1,'~', sep = '')
tempmat[3,2] <- temp

temp <- paste(round(p.out.hiact.am$pval,3), ' ~', p.out.hiact.am$row+1, ',', p.out.hiact.am$col+1,'~', sep = '')
tempmat[3,3] <- temp

temp <- paste(round(p.out.hiact.af$pval,3), ' ~', p.out.hiact.af$row+1, ',', p.out.hiact.af$col+1,'~', sep = '')
tempmat[3,4] <- temp

temp <- paste(round(p.out.hiact.n$pval,3), ' ~', p.out.hiact.n$row+1, ',', p.out.hiact.n$col+1,'~', sep = '')
tempmat[3,5] <- temp


# Eating

#temp <- paste(round(p.out.eat.all$pval,3), ' ~', p.out.eat.all$row+1, ',', p.out.eat.all$col+1,'~', sep = '')
#temp <- paste('<0.001', ' ~', p.out.eat.all$row+1, ',', p.out.eat.all$col+1,'~', sep = '')
temp <- ifelse(round(p.out.eat.all$pval,3) == 0, paste('<0.001', ' ~', p.out.eat.all$row+1, ',', p.out.eat.all$col+1,'~', sep = ''), paste(round(p.out.eat.all$pval,3), ' ~', p.out.eat.all$row+1, ',', p.out.eat.all$col+1,'~', sep = ''))
tempmat[4,1] <- temp

temp <- paste(round(p.out.eat.l$pval,3), ' ~', p.out.eat.l$row+1, ',', p.out.eat.l$col+1,'~', sep = '')
tempmat[4,2] <- temp

temp <- paste(round(p.out.eat.am$pval,3), ' ~', p.out.eat.am$row+1, ',', p.out.eat.am$col+1,'~', sep = '')
tempmat[4,3] <- temp

temp <- paste(round(p.out.eat.af$pval,3), ' ~', p.out.eat.af$row+1, ',', p.out.eat.af$col+1,'~', sep = '')
tempmat[4,4] <- temp

temp <- paste(round(p.out.eat.n$pval,3), ' ~', p.out.eat.n$row+1, ',', p.out.eat.n$col+1,'~', sep = '')
tempmat[4,5] <- temp


# Rumenation

temp <- paste(round(p.out.rum.all$pval,3), ' ~', p.out.rum.all$row+1, ',', p.out.rum.all$col+1,'~', sep = '')
tempmat[5,1] <- temp

temp <- paste(round(p.out.rum.l$pval,3), ' ~', p.out.rum.l$row+1, ',', p.out.rum.l$col+1,'~', sep = '')
tempmat[5,2] <- temp

temp <- paste(round(p.out.rum.am$pval,3), ' ~', p.out.rum.am$row+1, ',', p.out.rum.am$col+1,'~', sep = '')
tempmat[5,3] <- temp

temp <- paste(round(p.out.rum.af$pval,3), ' ~', p.out.rum.af$row+1, ',', p.out.rum.af$col+1,'~', sep = '')
tempmat[5,4] <- temp

temp <- paste(round(p.out.rum.n$pval,3), ' ~', p.out.rum.n$row+1, ',', p.out.rum.n$col+1,'~', sep = '')
tempmat[5,5] <- temp


# Temperature

#temp <- paste(round(p.out.temp.all$pval,3), ' ~', p.out.temp.all$row+1, ',', p.out.temp.all$col+1,'~', sep = '')
#temp <- paste('<0.001', ' ~', p.out.temp.all$row+1, ',', p.out.temp.all$col+1,'~', sep = '')
temp <- ifelse(round(p.out.temp.all$pval,3) == 0, paste('<0.001', ' ~', p.out.temp.all$row+1, ',', p.out.temp.all$col+1,'~', sep = ''), paste(round(p.out.temp.all$pval,3), ' ~', p.out.temp.all$row+1, ',', p.out.temp.all$col+1,'~', sep = ''))
tempmat[6,1] <- temp

temp <- paste(round(p.out.temp.l$pval,3), ' ~', p.out.temp.l$row+1, ',', p.out.temp.l$col+1,'~', sep = '')
tempmat[6,2] <- temp
temp <- paste(round(p.out.temp.am$pval,3), ' ~', p.out.temp.am$row+1, ',', p.out.temp.am$col+1,'~', sep = '')
tempmat[6,3] <- temp
temp <- paste(round(p.out.temp.af$pval,3), ' ~', p.out.temp.af$row+1, ',', p.out.temp.af$col+1,'~', sep = '')
tempmat[6,4] <- temp
temp <- paste(round(p.out.temp.n$pval,3), ' ~', p.out.temp.n$row+1, ',', p.out.temp.n$col+1,'~', sep = '')
tempmat[6,5] <- temp

bvtresults <- tempmat
rownames(bvtresults) <- c('Nonactivity','Activity','High Activity','Eating','Rumination','Temperature')
colnames(bvtresults) <- c('All','Lounging','Morning','Afternoon','Night')

save(bvtresults, file = 'SensorMCETest.RData')
```

```
kable(bvtresults)
```

|  | All | Lounging | Morning | Afternoon | Night |
| --- | --- | --- | --- | --- | --- |
| Nonactivity | <0.001 7,5 | 0.048 2,5 | 0.432 8,7 | 0.002 2,5 | 0.132 2,6 |
| Activity | <0.001 3,8 | 0.006 2,8 | 0.038 2,11 | <0.001 2,3 | 0.033 2,9 |
| High Activity | 0.052 2,9 | 0.028 2,2 | 0.272 10,9 | 0.306 3,4 | 0.014 2,4 |
| Eating | 0.004 7,6 | 0.234 7,5 | 0.188 6,4 | 0.066 2,4 | 0.212 9,2 |
| Rumination | 0.021 2,4 | 0.05 9,3 | 0.325 7,2 | 0.083 10,3 | 0.152 8,10 |
| Temperature | <0.001 5,10 | 0.004 5,5 | 0.022 7,3 | 0.006 3,5 | 0.015 5,3 |

## Tube Plots

### Defining Plotting Functions

https://plot.ly/r/text-and-annotations/#styling-annotations

```
annotationkey <- data.frame(H = c(0, 1, 2, 3, 4, 5, 6, 7, 8, 9, 10, 11, 12, 13, 14, 15, 16, 17, 18, 19, 20, 21, 22, 23),
                            text = c("7pm",  "8pm",  "9pm",  "10pm", "11pm", "12pm", "1am",  "2am",  "3am",  "4am",  "5am",  "6am",  "7am",  "8am",  "9am",  "10am", "11am", "12am", "1pm",  "2pm",  "3pm",  "4pm",  "5pm",  "6pm"))

# setup tube plot function

tubeplot <- function(c, r, obs, radius = 1, filename = 'TubePlot', plot_title = '', annotationkey = data.frame(), mycamera = list(eye = list(x = 1, y = 1, z = 0)),  export_html = F, printtochunk = T, export_orca = F, orcascale = 10, ...){
  # c = data vector containing the variable for temporal position in cycle for each observation
  # r = data vector containing the variable for the replication of the cycle for each observation
  # obs = data used to color data point
  # radius = radius of circle being plotted
  # plotting options for plotly visualization
  
  require('plotly')
  
  h <- length(unique(c))
  d <- length(unique(r))
  
  # rescale h to have degrees of unit circle
  
  temp <- table(c)
  tempgap <- min(as.numeric(names(temp)[-1])-as.numeric(names(temp)[-length(temp)])) 
  
  deg <- c + (0-min(c))
  deg <- (2*pi)/(max(c) + tempgap) * c
  
  # calculate coordinates on circle
  
  x <- radius*cos(deg)
  y <- radius*sin(deg)
  
  # set up data frame & annotations
  
  datplot <- data.frame(X = x, Y = y, Z = r, deg = deg, Obs = obs)

  temp <- subset(datplot, datplot$Z == min(datplot$Z))
  temp$H <- c[r == min(r)]
  temp <- temp[order(temp$H),]
  temp$Xnew <- (radius*1.2)*cos(temp$deg)
  temp$Ynew <- (radius*1.2)*sin(temp$deg)
  
  if(nrow(annotationkey)>0){
    names(annotationkey) <- c('H','text')
    temp <- merge(temp, annotationkey)
  }else{
    temp$text <- temp$H
  }
  
  myannotations <- list()
  
  for(i in seq(1, nrow(temp), by = 1)){
    myannotations[[length(myannotations)+1]] <- list(x = temp$Xnew[i],
                                                     y = temp$Ynew[i],
                                                     z = temp$Z[i],
                                                     text = as.character(temp$text[i]), 
                                                     showarrow = F
                                                     #textangle = temp$deg[i] * 180/pi
                                                     )
  }
  

  # plot this monster o.O
  
    tempa <- paste(getwd(), filename ,'.html', sep = '') 
    
    p1 <-  plot_ly(x=datplot$X, y=datplot$Y, z = datplot$Z, color = datplot$Obs,
                   type="scatter3d", mode="markers") %>% 
                   layout(title = plot_title, scene = list(annotations = myannotations, camera = mycamera)  )
    
    if(export_html){
     suppressWarnings(htmlwidgets::saveWidget(as_widget(p1), file = tempa, selfcontained = T))
    }
    
    if(printtochunk){
      plot_ly(x=datplot$X, y=datplot$Y, z = datplot$Z, color = datplot$Obs,
                   type="scatter3d", mode="markers") %>% 
                   layout(title = plot_title, scene = list(annotations = myannotations,
                                                           camera = mycamera)  
                          )
    }
    
    if(export_orca){
      orca(p1, file = paste(filename, '.jpeg', sep = ''), scale = orcascale)
    }
    
  
  
    
}


dat <- activedat
temp = list(eye = list(x = 0, y = -1.3, z = 0.2))
tubeplot(dat$Hour, as.numeric(strftime(dat$TimeStampF, format = "%j")), dat[,5], filename = '/Viz/BivariateAnalyses/Test',  plot_title = "Tube Plot of Activity Data", annotationkey  = annotationkey , mycamera = temp, marker = list(size = 10))
```

Alright, now I want to make a function that will generate tube plots for each of the queue subgroups identified as having a significant association with sensor.

```
groupTubePlot <- function(dat, c, r, g, subsethour = c(), filename = 'TubePlot', plot_title = 'TubePlot', ...){
  # dat = cows arranged as columns, time observation as rows
  # c = data vector containing the variable for temporal position in cycle for each observation
  # r = data vector containing the variable for the replication of the cycle for each observation
  # g = group assignments made by hclust tree
  #... values to be fed into tube plot function
  
  require('plotly')
  
  tempobs <- matrix(NA, ncol = length(unique(g)), nrow = nrow(dat))
  
  for(tempg in 1:length(unique(g))){
    
    tempsubdat <- dat[,g==tempg]
    tempobs[ ,tempg] <- apply(tempsubdat, 1, function(x) median(x, na.rm = T))
    
  }
  
  if(length(subsethour)>0){
    tempobs[which(!c%in%subsethour), ] <- NA
  }
  
  mycolormin = min(tempobs, na.rm = T)
  mycolormax = max(tempobs, na.rm = T)
  
  # c <- c(c,13)
  # r <- c(r,max(r)+1)
  # tempobs <- rbind(tempobs, rep(mycolormax, ncol(tempobs)))
  #tempobs <- rbind(tempobs, rep(NA, ncol(tempobs)))
  #temp <- c(tempobs[,1], mycolormax, NA)
  
  # fix max
  tempobs[c==13 & r == min(r, na.rm = T),] <- rep(mycolormax, ncol(tempobs))
  c <- c(c,13)
  r <- c(r,min(r))
  tempobs <- rbind(tempobs, rep(NA, ncol(tempobs)))
  
  #fix min
  tempobs[c==13 & r == max(r, na.rm = T),] <- rep(mycolormin, ncol(tempobs))
  c <- c(c,13)
  r <- c(r,max(r))
  tempobs <- rbind(tempobs, rep(NA, ncol(tempobs)))
   
  for(tempg in 1:length(unique(g))){
    
    tubeplot(c, r, tempobs[,tempg], 
             filename = paste(filename, '_Group', tempg, sep=''),
             plot_title = paste(plot_title, ': Group ', tempg, sep=''), 
             ...)   
    
    if(tempg<length(unique(g))){
      for(contg in (tempg + 1):length(unique(g))){
        
        conts = tempobs[,tempg] - tempobs[,contg]
        
        tubeplot(c, r, conts, 
                 filename = paste(filename, '_ContrastG', tempg, 'vG', contg, sep=''),
                 plot_title = paste(plot_title, ': Contrast Group ', tempg, ' vs Group ', contg, sep=''),
                 #marker = list(colorscale='Spectral'),
                 ...)
        
      }
    }
    
  }
  
  
}
```

### Activity

```
dat <- activedat[,names(activedat) %in% cowlist_healthy_sensor]
dim(dat)
```

```
## [1] 1008  109
```

```
g <- cutree(h.dist.all,2)

table(g) # group 1 is the fron 2/3 of the herd, group 2 the back 1/3
```

```
## g
##  1  2 
## 80 34
```

```
# median group entry positions
temp <- apply(milkquantdat_all2,1,function(x) median(x, na.rm = T))
tapply(temp, g, median) # group 1 is in the front, group 2 in the back
```

```
##         1         2 
## 0.3664127 0.7804293
```

```
# cull to consensus data set
g <- g[names(g) %in% cowlist_healthy_sensor]
length(g)
```

```
## [1] 109
```

```
# check alignment
names(dat) == names(g)
```

```
##   [1] TRUE TRUE TRUE TRUE TRUE TRUE TRUE TRUE TRUE TRUE TRUE TRUE TRUE TRUE
##  [15] TRUE TRUE TRUE TRUE TRUE TRUE TRUE TRUE TRUE TRUE TRUE TRUE TRUE TRUE
##  [29] TRUE TRUE TRUE TRUE TRUE TRUE TRUE TRUE TRUE TRUE TRUE TRUE TRUE TRUE
##  [43] TRUE TRUE TRUE TRUE TRUE TRUE TRUE TRUE TRUE TRUE TRUE TRUE TRUE TRUE
##  [57] TRUE TRUE TRUE TRUE TRUE TRUE TRUE TRUE TRUE TRUE TRUE TRUE TRUE TRUE
##  [71] TRUE TRUE TRUE TRUE TRUE TRUE TRUE TRUE TRUE TRUE TRUE TRUE TRUE TRUE
##  [85] TRUE TRUE TRUE TRUE TRUE TRUE TRUE TRUE TRUE TRUE TRUE TRUE TRUE TRUE
##  [99] TRUE TRUE TRUE TRUE TRUE TRUE TRUE TRUE TRUE TRUE TRUE
```

```
# pull time variables

c <- activedat$Hour
r <- as.numeric(as.Date(activedat$TimeStampF, format = "%j")) - 17182

dat[c==5, ] <- NA
dat[c==21, ] <- NA
dat[c==13, ] <- NA

groupTubePlot(dat, c, r, g, 
              subsethour <- c(14,15,16,17,18,19,20),
              filename = '/Viz/BivariateAnalyses/Tubeplots/Active/MorningActive',
              plot_title = 'Morning Minutes Active',
              marker = list(size = 10),
              annotationkey  = annotationkey,
              mycamera = list(eye = list(x = -0.4, y = -1.6, z = 0)))

groupTubePlot(dat, c, r, g, 
              subsethour <- c(6,7,8,9,10,11,12),
              filename = '/Viz/BivariateAnalyses/Tubeplots/Active/NightActive',
              plot_title = 'Night Minutes Active',
              marker = list(size = 10),
              annotationkey  = annotationkey,
              mycamera = list(eye = list(x = -1.3, y = 1.2, z = 0)))  

groupTubePlot(dat, c, r, g, 
              subsethour <- c(23,0,1,2,3,4),
              filename = '/Viz/BivariateAnalyses/Tubeplots/Active/AfterActive',
              plot_title = 'Afternoon Minutes Active',
              marker = list(size = 10),
              annotationkey  = annotationkey,
              mycamera = list(eye = list(x = 1.7, y = 0.5, z = 0)))

groupTubePlot(dat, c, r, g,
              filename = '/Viz/BivariateAnalyses/Tubeplots/Active/Active',
              plot_title = 'Minutes Active',
              marker = list(size = 10),
              annotationkey  = annotationkey,
              mycamera = list(eye = list(x = 1.5, y = 1.5, z = 0)))
```

Export files

```
if(isexporting){

  # morning
  
  if(isconda){
    
    groupTubePlot(dat, c, r, g, 
                  subsethour <- c(14,15,16,17,18,19,20),
                  filename = '/Viz/BivariateAnalyses/Tubeplots/Active/MorningActive',
                  plot_title = 'Morning Minutes Active',
                  marker = list(size = 10),
                  printtochunk = F, export_orca = T,
                  annotationkey  = annotationkey,
                  mycamera = list(eye = list(x = -0.4, y = -1.6, z = 0)))
    
  }else{
    
    groupTubePlot(dat, c, r, g, 
                  subsethour <- c(14,15,16,17,18,19,20),
                  filename = '/Viz/BivariateAnalyses/Tubeplots/Active/MorningActive',
                  plot_title = 'Morning Minutes Active',
                  marker = list(size = 10),
                  printtochunk = F, export_html = T,
                  annotationkey  = annotationkey,
                  mycamera = list(eye = list(x = -0.4, y = -1.6, z = 0)))
    
  }
  
  # afternoon
  
  if(isconda){
    
    groupTubePlot(dat, c, r, g, 
                  subsethour <- c(23,0,1,2,3,4),
                  filename = '/Viz/BivariateAnalyses/Tubeplots/Active/AfterActive',
                  plot_title = 'Afternoon Minutes Active',
                  marker = list(size = 10),
                  printtochunk = F, export_orca = T,
                  annotationkey  = annotationkey,
                  mycamera = list(eye = list(x = 1.7, y = 0.5, z = 0)))
  }else{
    groupTubePlot(dat, c, r, g, 
                  subsethour <- c(23,0,1,2,3,4),
                  filename = '/Viz/BivariateAnalyses/Tubeplots/Active/AfterActive',
                  plot_title = 'Afternoon Minutes Active',
                  marker = list(size = 10),
                  printtochunk = F, export_html = T,
                  annotationkey  = annotationkey,
                  mycamera = list(eye = list(x = 1.7, y = 0.5, z = 0)))
  }
  
  # night
  
  if(isconda){
    
    groupTubePlot(dat, c, r, g, 
                  subsethour <- c(6,7,8,9,10,11,12),
                  filename = '/Viz/BivariateAnalyses/Tubeplots/Active/NightActive',
                  plot_title = 'Night Minutes Active',
                  printtochunk = F, export_orca = T,
                  marker = list(size = 10),
                  annotationkey  = annotationkey,
                  mycamera = list(eye = list(x = -1.3, y = 1.2, z = 0))) 
    
  }else{
    
    groupTubePlot(dat, c, r, g, 
                  subsethour <- c(6,7,8,9,10,11,12),
                  filename = '/Viz/BivariateAnalyses/Tubeplots/Active/NightActive',
                  plot_title = 'Night Minutes Active',
                  printtochunk = F, export_html = T,
                  marker = list(size = 10),
                  annotationkey  = annotationkey,
                  mycamera = list(eye = list(x = -1.3, y = 1.2, z = 0)))
    
  }
  
  # all
  
  if(!isconda){
    groupTubePlot(dat, c, r, g,
                  filename = '/Viz/BivariateAnalyses/Tubeplots/Active/Active',
                  plot_title = 'Minutes Active',
                  marker = list(size = 10),
                  export_html = T, printtochunk = F, 
                  annotationkey  = annotationkey,
                  mycamera = list(eye = list(x = 1.5, y = 1.5, z = 0)))
  }

}
```

### High Activity

```
dat <- hiactivedat[,names(hiactivedat) %in% cowlist_healthy_sensor]
dim(dat)
```

```
## [1] 1008  109
```

```
#g <- cutree(h.dist.all,2)
#g <- g[names(g) %in% cowlist_healthy_sensor]
#length(g)

# check alignment

names(dat) == names(g)
```

```
##   [1] TRUE TRUE TRUE TRUE TRUE TRUE TRUE TRUE TRUE TRUE TRUE TRUE TRUE TRUE
##  [15] TRUE TRUE TRUE TRUE TRUE TRUE TRUE TRUE TRUE TRUE TRUE TRUE TRUE TRUE
##  [29] TRUE TRUE TRUE TRUE TRUE TRUE TRUE TRUE TRUE TRUE TRUE TRUE TRUE TRUE
##  [43] TRUE TRUE TRUE TRUE TRUE TRUE TRUE TRUE TRUE TRUE TRUE TRUE TRUE TRUE
##  [57] TRUE TRUE TRUE TRUE TRUE TRUE TRUE TRUE TRUE TRUE TRUE TRUE TRUE TRUE
##  [71] TRUE TRUE TRUE TRUE TRUE TRUE TRUE TRUE TRUE TRUE TRUE TRUE TRUE TRUE
##  [85] TRUE TRUE TRUE TRUE TRUE TRUE TRUE TRUE TRUE TRUE TRUE TRUE TRUE TRUE
##  [99] TRUE TRUE TRUE TRUE TRUE TRUE TRUE TRUE TRUE TRUE TRUE
```

```
# pull time variables

c <- hiactivedat$Hour
r <- as.numeric(as.Date(hiactivedat$TimeStampF, format = "%j")) - 17182

dat[c==5, ] <- NA
dat[c==21, ] <- NA
dat[c==13, ] <- NA

groupTubePlot(dat, c, r, g, 
              subsethour <- c(14,15,16,17,18,19,20),
              filename = '/Viz/BivariateAnalyses/Tubeplots/HighActive/MorningHighActive',
              plot_title = 'Morning Minutes High Active',
              marker = list(size = 10),
              mycamera = list(eye = list(x = -0.4, y = -1.6, z = 0)),
              annotationkey  = annotationkey)

groupTubePlot(dat, c, r, g, 
              subsethour <- c(6,7,8,9,10,11,12),
              filename = '/Viz/BivariateAnalyses/Tubeplots/HighActive/NightHighActive',
              plot_title = 'Night Minutes High Active',
              marker = list(size = 10),
              mycamera = list(eye = list(x = -1.3, y = 1.2, z = 0)),
              annotationkey  = annotationkey)  

groupTubePlot(dat, c, r, g, 
              subsethour <- c(23,0,1,2,3,4),
              filename = '/Viz/BivariateAnalyses/Tubeplots/HighActive/AfterHighActive',
              plot_title = 'Afternoon Minutes High Active',
              marker = list(size = 10),
              mycamera = list(eye = list(x = 1.7, y = 0.5, z = 0)),
              annotationkey  = annotationkey)

groupTubePlot(dat, c, r, g, 
              filename = '/Viz/BivariateAnalyses/Tubeplots/HighActive/HighActive',
              plot_title = 'Minutes High Activity',
              annotationkey  = annotationkey, 
              marker = list(size = 10),
              mycamera = list(eye = list(x = 1.5, y = 1.5, z = 0))
                          )
```

Exporting files

```
if(isexporting){
  
  # morning
  
  if(isconda){
    
    groupTubePlot(dat, c, r, g, 
                  subsethour <- c(14,15,16,17,18,19,20),
                  filename = '/Viz/BivariateAnalyses/Tubeplots/HighActive/MorningHighActive',
                  plot_title = 'Morning Minutes Highly Active',
                  marker = list(size = 10),
                  printtochunk = F, export_orca = T,
                  annotationkey  = annotationkey,
                  mycamera = list(eye = list(x = -0.4, y = -1.6, z = 0))
                  )
    
  }else{
    
    groupTubePlot(dat, c, r, g, 
                  subsethour <- c(14,15,16,17,18,19,20),
                  filename = '/Viz/BivariateAnalyses/Tubeplots/HighActive/MorningHighActive',
                  plot_title = 'Morning Minutes Highly Active',
                  marker = list(size = 10),
                  printtochunk = F, export_html = T,
                  annotationkey  = annotationkey,
                  mycamera = list(eye = list(x = -0.4, y = -1.6, z = 0))
                  )
    
  }
  
  # afternoon
  
  if(isconda){
    
    groupTubePlot(dat, c, r, g, 
                  subsethour <- c(23,0,1,2,3,4),
                  filename = '/Viz/BivariateAnalyses/Tubeplots/HighActive/AfterHighActive',
                  plot_title = 'Afternoon Minutes Highly Active',
                  marker = list(size = 10),
                  printtochunk = F, export_orca = T,
                  annotationkey  = annotationkey,
                  mycamera = list(eye = list(x = 1.7, y = 0.5, z = 0)))
  }else{
    groupTubePlot(dat, c, r, g, 
                  subsethour <- c(23,0,1,2,3,4),
                  filename = '/Viz/BivariateAnalyses/Tubeplots/HighActive/AfterHighActive',
                  plot_title = 'Afternoon Minutes Highly Active',
                  marker = list(size = 10),
                  printtochunk = F, export_html = T,
                  annotationkey  = annotationkey,
                  mycamera = list(eye = list(x = 1.7, y = 0.5, z = 0))
                  )
  }
  
  # night
  
  if(isconda){
    
    groupTubePlot(dat, c, r, g, 
                  subsethour <- c(6,7,8,9,10,11,12),
                  filename = '/Viz/BivariateAnalyses/Tubeplots/HighActive/NightHighActive',
                  plot_title = 'Night Minutes Highly Active',
                  printtochunk = F, export_orca = T,
                  marker = list(size = 10),
                  annotationkey  = annotationkey,
                  mycamera = list(eye = list(x = -1.3, y = 1.2, z = 0))) 
    
  }else{
    
    groupTubePlot(dat, c, r, g, 
                  subsethour <- c(6,7,8,9,10,11,12),
                  filename = '/Viz/BivariateAnalyses/Tubeplots/HighActive/NightHighActive',
                  plot_title = 'Night Minutes Highly Active',
                  printtochunk = F, export_html = T,
                  marker = list(size = 10),
                  annotationkey  = annotationkey,
                  mycamera = list(eye = list(x = -1.3, y = 1.2, z = 0))
                  )
    
  }
  
  # all
  
  if(!isconda){
    groupTubePlot(dat, c, r, g,
                  filename = '/Viz/BivariateAnalyses/Tubeplots/HighActive/HighActive',
                  plot_title = 'Minutes Highly Active',
                  marker = list(size = 10),
                  export_html = T, printtochunk = F, 
                  annotationkey  = annotationkey,
                  mycamera = list(eye = list(x = 1.5, y = 1.5, z = 0)))
  }
  
}
```

### Nonactive

```
dat <- nonactivedat[,names(nonactivedat) %in% cowlist_healthy_sensor]
dim(dat)
```

```
## [1] 1008  109
```

```
# g <- cutree(h.dist.all,2)
# g <- g[names(g) %in% cowlist_healthy_sensor]
# length(g)

# check alignment

names(g) == names(dat)
```

```
##   [1] TRUE TRUE TRUE TRUE TRUE TRUE TRUE TRUE TRUE TRUE TRUE TRUE TRUE TRUE
##  [15] TRUE TRUE TRUE TRUE TRUE TRUE TRUE TRUE TRUE TRUE TRUE TRUE TRUE TRUE
##  [29] TRUE TRUE TRUE TRUE TRUE TRUE TRUE TRUE TRUE TRUE TRUE TRUE TRUE TRUE
##  [43] TRUE TRUE TRUE TRUE TRUE TRUE TRUE TRUE TRUE TRUE TRUE TRUE TRUE TRUE
##  [57] TRUE TRUE TRUE TRUE TRUE TRUE TRUE TRUE TRUE TRUE TRUE TRUE TRUE TRUE
##  [71] TRUE TRUE TRUE TRUE TRUE TRUE TRUE TRUE TRUE TRUE TRUE TRUE TRUE TRUE
##  [85] TRUE TRUE TRUE TRUE TRUE TRUE TRUE TRUE TRUE TRUE TRUE TRUE TRUE TRUE
##  [99] TRUE TRUE TRUE TRUE TRUE TRUE TRUE TRUE TRUE TRUE TRUE
```

```
# pulling temporal variables

c <- nonactivedat$Hour
r <- as.numeric(as.Date(nonactivedat$TimeStampF, format = "%j")) - 17182

dat[c==5, ] <- NA
dat[c==21, ] <- NA
dat[c==13, ] <- NA

groupTubePlot(dat, c, r, g, 
              subsethour <- c(14,15,16,17,18,19,20),
              filename = '/Viz/BivariateAnalyses/Tubeplots/NonActive/MorningNonActive',
              plot_title = 'Morning Minutes Not Active',
              marker = list(size = 10),
              mycamera = list(eye = list(x = -0.4, y = -1.6, z = 0)),
              annotationkey  = annotationkey)

groupTubePlot(dat, c, r, g, 
              subsethour <- c(6,7,8,9,10,11,12),
              filename = '/Viz/BivariateAnalyses/Tubeplots/NonActive/NightNonActive',
              plot_title = 'Night Minutes Not Active',
              marker = list(size = 10),
              mycamera = list(eye = list(x = -1.3, y = 1.2, z = 0)),
              annotationkey  = annotationkey)  

groupTubePlot(dat, c, r, g, 
              subsethour <- c(23,0,1,2,3,4),
              filename = '/Viz/BivariateAnalyses/Tubeplots/NonActive/AfterNonActive',
              plot_title = 'Afternoon Minutes Not Active',
              marker = list(size = 10),
              mycamera = list(eye = list(x = 1.7, y = 0.5, z = 0)),
              annotationkey  = annotationkey)

groupTubePlot(dat, c, r, g, 
              filename = '/Viz/BivariateAnalyses/Tubeplots/NonActive/NonActive',
              plot_title = 'Minutes Not Active',
              annotationkey  = annotationkey, 
              marker = list(size = 10),
              mycamera = list(eye = list(x = 1.5, y = 1.5, z = 0))
                          )
```

Exporting files

```
if(isexporting){
  
  # morning
  
  if(isconda){
    
    groupTubePlot(dat, c, r, g, 
                  subsethour <- c(14,15,16,17,18,19,20),
                  filename = '/Viz/BivariateAnalyses/Tubeplots/Nonactive/MorningNonactive',
                  plot_title = 'Morning Minutes Nonactive',
                  marker = list(size = 10),
                  printtochunk = F, export_orca = T,
                  annotationkey  = annotationkey,
                  mycamera = list(eye = list(x = -0.4, y = -1.6, z = 0)))
    
  }else{
    
    groupTubePlot(dat, c, r, g, 
                  subsethour <- c(14,15,16,17,18,19,20),
                  filename = '/Viz/BivariateAnalyses/Tubeplots/Nonactive/MorningNonactive',
                  plot_title = 'Morning Minutes Nonactive',
                  marker = list(size = 10),
                  printtochunk = F, export_html = T,
                  annotationkey  = annotationkey,
                  mycamera = list(eye = list(x = -0.4, y = -1.6, z = 0)))
    
  }
  
  # afternoon
  
  if(isconda){
    
    groupTubePlot(dat, c, r, g, 
                  subsethour <- c(23,0,1,2,3,4),
                  filename = '/Viz/BivariateAnalyses/Tubeplots/Nonactive/AfterNonactive',
                  plot_title = 'Afternoon Minutes Nonactive',
                  marker = list(size = 10),
                  printtochunk = F, export_orca = T,
                  annotationkey  = annotationkey,
                  mycamera = list(eye = list(x = 1.7, y = 0.5, z = 0)))
  }else{
    groupTubePlot(dat, c, r, g, 
                  subsethour <- c(23,0,1,2,3,4),
                  filename = '/Viz/BivariateAnalyses/Tubeplots/Nonactive/AfterNonactive',
                  plot_title = 'Afternoon Minutes Nonactive',
                  marker = list(size = 10),
                  printtochunk = F, export_html = T,
                  annotationkey  = annotationkey,
                  mycamera = list(eye = list(x = 1.7, y = 0.5, z = 0)))
  }
  
  # night
  
  if(isconda){
    
    groupTubePlot(dat, c, r, g, 
                  subsethour <- c(6,7,8,9,10,11,12),
                  filename = '/Viz/BivariateAnalyses/Tubeplots/Nonactive/NightNonactive',
                  plot_title = 'Night Minutes Nonactive',
                  printtochunk = F, export_orca = T,
                  marker = list(size = 10),
                  annotationkey  = annotationkey,
                  mycamera = list(eye = list(x = -1.3, y = 1.2, z = 0))) 
    
  }else{
    
    groupTubePlot(dat, c, r, g, 
                  subsethour <- c(6,7,8,9,10,11,12),
                  filename = '/Viz/BivariateAnalyses/Tubeplots/Nonactive/NightNonactive',
                  plot_title = 'Night Minutes Nonactive',
                  printtochunk = F, export_html = T,
                  marker = list(size = 10),
                  annotationkey  = annotationkey,
                  mycamera = list(eye = list(x = -1.3, y = 1.2, z = 0)))
    
  }
  
  # all
  
  if(!isconda){
    groupTubePlot(dat, c, r, g,
                  filename = '/Viz/BivariateAnalyses/Tubeplots/Nonactive/Nonactive',
                  plot_title = 'Minutes Nonactive',
                  marker = list(size = 10),
                  export_html = T, printtochunk = F, 
                  annotationkey  = annotationkey,
                  mycamera = list(eye = list(x = 1.5, y = 1.5, z = 0)))
  }
  
}
```

### Rumination

```
dat <- rumdat[,names(rumdat) %in% cowlist_healthy_sensor]
dim(dat)
```

```
## [1] 1008  109
```

```
# g <- cutree(h.dist.all, 3)
# g <- g[names(g) %in% cowlist_healthy_sensor]
# length(g)

#check alignment
names(g) == names(dat)
```

```
##   [1] TRUE TRUE TRUE TRUE TRUE TRUE TRUE TRUE TRUE TRUE TRUE TRUE TRUE TRUE
##  [15] TRUE TRUE TRUE TRUE TRUE TRUE TRUE TRUE TRUE TRUE TRUE TRUE TRUE TRUE
##  [29] TRUE TRUE TRUE TRUE TRUE TRUE TRUE TRUE TRUE TRUE TRUE TRUE TRUE TRUE
##  [43] TRUE TRUE TRUE TRUE TRUE TRUE TRUE TRUE TRUE TRUE TRUE TRUE TRUE TRUE
##  [57] TRUE TRUE TRUE TRUE TRUE TRUE TRUE TRUE TRUE TRUE TRUE TRUE TRUE TRUE
##  [71] TRUE TRUE TRUE TRUE TRUE TRUE TRUE TRUE TRUE TRUE TRUE TRUE TRUE TRUE
##  [85] TRUE TRUE TRUE TRUE TRUE TRUE TRUE TRUE TRUE TRUE TRUE TRUE TRUE TRUE
##  [99] TRUE TRUE TRUE TRUE TRUE TRUE TRUE TRUE TRUE TRUE TRUE
```

```
# pull temporal variables
c <- rumdat$Hour
r <- as.numeric(as.Date(rumdat$TimeStampF, format = "%j")) - 17182

dat[c==5, ] <- NA
dat[c==21, ] <- NA
dat[c==13, ] <- NA

groupTubePlot(dat, c, r, g, 
              subsethour <- c(14,15,16,17,18,19,20),
              filename = '/Viz/BivariateAnalyses/Tubeplots/Rumination/MorningRumination',
              plot_title = 'Morning Minutes Ruminating',
              marker = list(size = 10),
              mycamera = list(eye = list(x = -0.4, y = -1.6, z = 0)),
              annotationkey  = annotationkey)

groupTubePlot(dat, c, r, g, 
              subsethour <- c(6,7,8,9,10,11,12),
              filename = '/Viz/BivariateAnalyses/Tubeplots/Rumination/NightRumination',
              plot_title = 'Night Minutes Ruminating',
              marker = list(size = 10),
              mycamera = list(eye = list(x = -1.3, y = 1.2, z = 0)),
              annotationkey  = annotationkey)  

groupTubePlot(dat, c, r, g, 
              subsethour <- c(23,0,1,2,3,4),
              filename = '/Viz/BivariateAnalyses/Tubeplots/Rumination/AfterRumination',
              plot_title = 'Afternoon Minutes Ruminating',
              marker = list(size = 10),
              mycamera = list(eye = list(x = 1.7, y = 0.5, z = 0)),
              annotationkey  = annotationkey)


groupTubePlot(dat, c, r, g, 
              filename = '/Viz/BivariateAnalyses/Tubeplots/Rumination/Rumination',
              plot_title = 'Minutes Ruminating',
              annotationkey  = annotationkey, 
              mycamera = list(eye = list(x = 1.5, y = 1.5, z = 0)),
              marker = list(size = 10)
              )
```

Exporting files

```
if(isexporting){
  
  # morning
  
  if(isconda){
    
    groupTubePlot(dat, c, r, g, 
                  subsethour <- c(14,15,16,17,18,19,20),
                  filename = '/Viz/BivariateAnalyses/Tubeplots/Rumination/MorningRumination',
                  plot_title = 'Morning Minutes Rumination',
                  marker = list(size = 10),
                  printtochunk = F, export_orca = T,
                  annotationkey  = annotationkey,
                  mycamera = list(eye = list(x = -0.4, y = -1.6, z = 0))
                  )
    
  }else{
    
    groupTubePlot(dat, c, r, g, 
                  subsethour <- c(14,15,16,17,18,19,20),
                  filename = '/Viz/BivariateAnalyses/Tubeplots/Rumination/MorningRumination',
                  plot_title = 'Morning Minutes Rumination',
                  marker = list(size = 10),
                  printtochunk = F, export_html = T,
                  annotationkey  = annotationkey,
                  mycamera = list(eye = list(x = -0.4, y = -1.6, z = 0))
                  )
    
  }
  
  # afternoon
  
  if(isconda){
    
    groupTubePlot(dat, c, r, g, 
                  subsethour <- c(23,0,1,2,3,4),
                  filename = '/Viz/BivariateAnalyses/Tubeplots/Rumination/AfterRumination',
                  plot_title = 'Afternoon Minutes Rumination',
                  marker = list(size = 10),
                  printtochunk = F, export_orca = T,
                  annotationkey  = annotationkey,
                  mycamera = list(eye = list(x = 1.7, y = 0.5, z = 0)))
  }else{
    groupTubePlot(dat, c, r, g, 
                  subsethour <- c(23,0,1,2,3,4),
                  filename = '/Viz/BivariateAnalyses/Tubeplots/Rumination/AfterRumination',
                  plot_title = 'Afternoon Minutes Rumination',
                  marker = list(size = 10),
                  printtochunk = F, export_html = T,
                  annotationkey  = annotationkey,
                  mycamera = list(eye = list(x = 1.7, y = 0.5, z = 0)))
  }
  
  # night
  
  if(isconda){
    
    groupTubePlot(dat, c, r, g, 
                  subsethour <- c(6,7,8,9,10,11,12),
                  filename = '/Viz/BivariateAnalyses/Tubeplots/Rumination/NightRumination',
                  plot_title = 'Night Minutes Rumination',
                  printtochunk = F, export_orca = T,
                  marker = list(size = 10),
                  annotationkey  = annotationkey,
                  mycamera = list(eye = list(x = -1.3, y = 1.2, z = 0))) 
    
  }else{
    
    groupTubePlot(dat, c, r, g, 
                  subsethour <- c(6,7,8,9,10,11,12),
                  filename = '/Viz/BivariateAnalyses/Tubeplots/Rumination/NightRumination',
                  plot_title = 'Night Minutes Rumination',
                  printtochunk = F, export_html = T,
                  marker = list(size = 10),
                  annotationkey  = annotationkey,
                  mycamera = list(eye = list(x = -1.3, y = 1.2, z = 0)))
    
  }
  
  # all
  
  if(!isconda){
    groupTubePlot(dat, c, r, g,
                  filename = '/Viz/BivariateAnalyses/Tubeplots/Rumination/Rumination',
                  plot_title = 'Minutes Rumination',
                  marker = list(size = 10),
                  export_html = T, printtochunk = F, 
                  annotationkey  = annotationkey,
                  mycamera = list(eye = list(x = 1.5, y = 1.5, z = 0)))
  }
  
}
```

### Eating

```
dat <- eatdat[,names(eatdat) %in% cowlist_healthy_sensor]
dim(dat)
```

```
## [1] 1008  109
```

```
# g <- cutree(h.dist.all,3)
# g <- g[names(g) %in% cowlist_healthy_sensor]
# length(g)

# checking alignment

names(g) == names(dat)
```

```
##   [1] TRUE TRUE TRUE TRUE TRUE TRUE TRUE TRUE TRUE TRUE TRUE TRUE TRUE TRUE
##  [15] TRUE TRUE TRUE TRUE TRUE TRUE TRUE TRUE TRUE TRUE TRUE TRUE TRUE TRUE
##  [29] TRUE TRUE TRUE TRUE TRUE TRUE TRUE TRUE TRUE TRUE TRUE TRUE TRUE TRUE
##  [43] TRUE TRUE TRUE TRUE TRUE TRUE TRUE TRUE TRUE TRUE TRUE TRUE TRUE TRUE
##  [57] TRUE TRUE TRUE TRUE TRUE TRUE TRUE TRUE TRUE TRUE TRUE TRUE TRUE TRUE
##  [71] TRUE TRUE TRUE TRUE TRUE TRUE TRUE TRUE TRUE TRUE TRUE TRUE TRUE TRUE
##  [85] TRUE TRUE TRUE TRUE TRUE TRUE TRUE TRUE TRUE TRUE TRUE TRUE TRUE TRUE
##  [99] TRUE TRUE TRUE TRUE TRUE TRUE TRUE TRUE TRUE TRUE TRUE
```

```
# pulling time variables

c <- eatdat$Hour
r <- as.numeric(as.Date(eatdat$TimeStampF, format = "%j")) - 17182

dat[c==5, ] <- NA
dat[c==21, ] <- NA
dat[c==13, ] <- NA


groupTubePlot(dat, c, r, g, 
              subsethour <- c(14,15,16,17,18,19,20),
              filename = '/Viz/BivariateAnalyses/Tubeplots/Eating/MorningEating',
              plot_title = 'Morning Minutes Eating',
              marker = list(size = 10),
              mycamera = list(eye = list(x = -0.4, y = -1.6, z = 0)),
              annotationkey  = annotationkey)

groupTubePlot(dat, c, r, g, 
              subsethour <- c(6,7,8,9,10,11,12),
              filename = '/Viz/BivariateAnalyses/Tubeplots/Eating/NightEating',
              plot_title = 'Night Minutes Eating',
              marker = list(size = 10),
              mycamera = list(eye = list(x = -1.3, y = 1.2, z = 0)),
              annotationkey  = annotationkey)  

groupTubePlot(dat, c, r, g, 
              subsethour <- c(23,0,1,2,3,4),
              filename = '/Viz/BivariateAnalyses/Tubeplots/Eating/AfterEating',
              plot_title = 'Afternoon Minutes Eating',
              marker = list(size = 10),
              mycamera = list(eye = list(x = 1.7, y = 0.5, z = 0)),
              annotationkey  = annotationkey)


groupTubePlot(dat, c, r, g, 
              filename = '/Viz/BivariateAnalyses/Tubeplots/Eating/Eating',
              plot_title = 'Minutes Eating',
              annotationkey  = annotationkey, 
              mycamera = list(eye = list(x = 1.5, y = 1.5, z = 0)),
              marker = list(size = 10)
                          )
```

Exporting files

```
if(isexporting){
  
  # morning
  
  if(isconda){
    
    groupTubePlot(dat, c, r, g, 
                  subsethour <- c(14,15,16,17,18,19,20),
                  filename = '/Viz/BivariateAnalyses/Tubeplots/Eating/MorningEating',
                  plot_title = 'Morning Minutes Eating',
                  marker = list(size = 10),
                  printtochunk = F, export_orca = T,
                  annotationkey  = annotationkey,
                  mycamera = list(eye = list(x = -0.4, y = -1.6, z = 0))
    )
    
  }else{
    
    groupTubePlot(dat, c, r, g, 
                  subsethour <- c(14,15,16,17,18,19,20),
                  filename = '/Viz/BivariateAnalyses/Tubeplots/Eating/MorningEating',
                  plot_title = 'Morning Minutes Eating',
                  marker = list(size = 10),
                  printtochunk = F, export_html = T,
                  annotationkey  = annotationkey,
                  mycamera = list(eye = list(x = -0.4, y = -1.6, z = 0))
    )
    
  }
  
  # afternoon
  
  if(isconda){
    
    groupTubePlot(dat, c, r, g, 
                  subsethour <- c(23,0,1,2,3,4),
                  filename = '/Viz/BivariateAnalyses/Tubeplots/Eating/AfterEating',
                  plot_title = 'Afternoon Minutes Eating',
                  marker = list(size = 10),
                  printtochunk = F, export_orca = T,
                  annotationkey  = annotationkey,
                  mycamera = list(eye = list(x = 1.7, y = 0.5, z = 0)))
  }else{
    groupTubePlot(dat, c, r, g, 
                  subsethour <- c(23,0,1,2,3,4),
                  filename = '/Viz/BivariateAnalyses/Tubeplots/Eating/AfterEating',
                  plot_title = 'Afternoon Minutes Eating',
                  marker = list(size = 10),
                  printtochunk = F, export_html = T,
                  annotationkey  = annotationkey,
                  mycamera = list(eye = list(x = 1.7, y = 0.5, z = 0)))
  }
  
  # night
  
  if(isconda){
    
    groupTubePlot(dat, c, r, g, 
                  subsethour <- c(6,7,8,9,10,11,12),
                  filename = '/Viz/BivariateAnalyses/Tubeplots/Eating/NightEating',
                  plot_title = 'Night Minutes Eating',
                  printtochunk = F, export_orca = T,
                  marker = list(size = 10),
                  annotationkey  = annotationkey,
                  mycamera = list(eye = list(x = -1.3, y = 1.2, z = 0))) 
    
  }else{
    
    groupTubePlot(dat, c, r, g, 
                  subsethour <- c(6,7,8,9,10,11,12),
                  filename = '/Viz/BivariateAnalyses/Tubeplots/Eating/NightEating',
                  plot_title = 'Night Minutes Eating',
                  printtochunk = F, export_html = T,
                  marker = list(size = 10),
                  annotationkey  = annotationkey,
                  mycamera = list(eye = list(x = -1.3, y = 1.2, z = 0)))
    
  }
  
  # all
  
  if(!isconda){
    groupTubePlot(dat, c, r, g,
                  filename = '/Viz/BivariateAnalyses/Tubeplots/Eating/Eating',
                  plot_title = 'Minutes Eating',
                  marker = list(size = 10),
                  export_html = T, printtochunk = F, 
                  annotationkey  = annotationkey,
                  mycamera = list(eye = list(x = 1.5, y = 1.5, z = 0)))
  }
  
}
```

### Temperature

```
dat <- temperdat[,names(temperdat) %in% cowlist_healthy_sensor]
dim(dat)
```

```
## [1] 1008  109
```

```
g <- cutree(h.dist.all,5)

table(g) # groups are roughly equat size 15-30 cows
```

```
## g
##  1  2  3  4  5 
## 33 19 28 20 14
```

```
# median group entry positions
temp <- apply(milkquantdat_all2,1,function(x) median(x, na.rm = T))
tapply(temp, g, median) # group 1 is in the front, group 2 in the back
```

```
##          1          2          3          4          5 
## 0.54748603 0.07658506 0.32798972 0.73070371 0.87252265
```

```
#         1          2          3          4          5 
#0.54748603 0.07658506 0.32798972 0.73070371 0.87252265 


# cull to consensus data set

g <- g[names(g) %in% cowlist_healthy_sensor]
length(g)
```

```
## [1] 109
```

```
# check alignment

names(g) == names(dat)
```

```
##   [1] TRUE TRUE TRUE TRUE TRUE TRUE TRUE TRUE TRUE TRUE TRUE TRUE TRUE TRUE
##  [15] TRUE TRUE TRUE TRUE TRUE TRUE TRUE TRUE TRUE TRUE TRUE TRUE TRUE TRUE
##  [29] TRUE TRUE TRUE TRUE TRUE TRUE TRUE TRUE TRUE TRUE TRUE TRUE TRUE TRUE
##  [43] TRUE TRUE TRUE TRUE TRUE TRUE TRUE TRUE TRUE TRUE TRUE TRUE TRUE TRUE
##  [57] TRUE TRUE TRUE TRUE TRUE TRUE TRUE TRUE TRUE TRUE TRUE TRUE TRUE TRUE
##  [71] TRUE TRUE TRUE TRUE TRUE TRUE TRUE TRUE TRUE TRUE TRUE TRUE TRUE TRUE
##  [85] TRUE TRUE TRUE TRUE TRUE TRUE TRUE TRUE TRUE TRUE TRUE TRUE TRUE TRUE
##  [99] TRUE TRUE TRUE TRUE TRUE TRUE TRUE TRUE TRUE TRUE TRUE
```

```
# pull temporal variables

c <- temperdat$Hour
r <- as.numeric(as.Date(temperdat$TimeStampF, format = "%j")) - 17182

dat[c==5, ] <- NA
dat[c==21, ] <- NA
dat[c==13, ] <- NA

groupTubePlot(dat, c, r, g, 
              subsethour <- c(14,15,16,17,18,19,20),
              filename = '/Viz/BivariateAnalyses/Tubeplots/Temperature/MorningTemperature',
              plot_title = 'Morning Average Temperature',
              marker = list(size = 10),
              mycamera = list(eye = list(x = -0.4, y = -1.6, z = 0)),
              annotationkey  = annotationkey)

groupTubePlot(dat, c, r, g, 
              subsethour <- c(6,7,8,9,10,11,12),
              filename = '/Viz/BivariateAnalyses/Tubeplots/Temperature/NightTemperature',
              plot_title = 'Night Average Temperature',
              marker = list(size = 10),
              mycamera = list(eye = list(x = -1.3, y = 1.2, z = 0)),
              annotationkey  = annotationkey)  

groupTubePlot(dat, c, r, g, 
              subsethour <- c(23,0,1,2,3,4),
              filename = '/Viz/BivariateAnalyses/Tubeplots/Temperature/AfterTemperature',
              plot_title = 'Afternoon Average Temperature',
              marker = list(size = 10),
              mycamera = list(eye = list(x = 1.7, y = 0.5, z = 0)),
              annotationkey  = annotationkey)

groupTubePlot(dat, c, r, g, 
              filename = '/Viz/BivariateAnalyses/Tubeplots/Temperature/Temperature',
              plot_title = 'Average Body Temperature',
              annotationkey  = annotationkey, 
              marker = list(size = 10),
              mycamera = list(eye = list(x = 1.5, y = 1.5, z = 0))
              )  


# checking milk yields

temp <- merge(unique(dat.linmod.uml[,c('CowID','MilkYield')]), data.frame(CowID = names(g), Group = g), by = 'CowID', all.y = T)
temp[temp$Group == 2,]
```

```
##     CowID MilkYield Group
## 2    1090   101.000     2
## 6   13077    72.335     2
## 7   13267    71.100     2
## 16  13814    77.800     2
## 17  13836    71.000     2
## 25   1454    78.810     2
## 28  16829   100.540     2
## 36  20311    92.600     2
## 37   2053    97.080     2
## 45   2457    99.835     2
## 50  26451    91.610     2
## 59  31867    99.290     2
## 60  32607   112.065     2
## 72   4562    90.315     2
## 75  46769    99.700     2
## 81  55482    82.870     2
## 89  62002    99.100     2
## 98   7057   102.580     2
## 100  9048    99.000     2
```

```
tapply(temp$MilkYield, temp$Group, function(x) median(x, na.rm = T))
```

```
##       1       2       3       4       5 
##  95.675  97.080  93.520  98.070 104.745
```

Exporting files

```
if(isexporting){
  
  # morning
  
  if(isconda){
    
    groupTubePlot(dat, c, r, g, 
                  subsethour <- c(14,15,16,17,18,19,20),
                  filename = '/Viz/BivariateAnalyses/Tubeplots/Temperature/MorningTemperature',
                  plot_title = 'Morning Minutes Temperature',
                  marker = list(size = 10),
                  printtochunk = F, export_orca = T,
                  annotationkey  = annotationkey,
                  mycamera = list(eye = list(x = -0.4, y = -1.6, z = 0)))
    
  }else{
    
    groupTubePlot(dat, c, r, g, 
                  subsethour <- c(14,15,16,17,18,19,20),
                  filename = '/Viz/BivariateAnalyses/Tubeplots/Temperature/MorningTemperature',
                  plot_title = 'Morning Minutes Temperature',
                  marker = list(size = 10),
                  printtochunk = F, export_html = T,
                  annotationkey  = annotationkey,
                  mycamera = list(eye = list(x = -0.4, y = -1.6, z = 0)))
    
  }
  
  # afternoon
  
  if(isconda){
    
    groupTubePlot(dat, c, r, g, 
                  subsethour <- c(23,0,1,2,3,4),
                  filename = '/Viz/BivariateAnalyses/Tubeplots/Temperature/AfterTemperature',
                  plot_title = 'Afternoon Minutes Temperature',
                  marker = list(size = 10),
                  printtochunk = F, export_orca = T,
                  annotationkey  = annotationkey,
                  mycamera = list(eye = list(x = 1.7, y = 0.5, z = 0)))
  }else{
    groupTubePlot(dat, c, r, g, 
                  subsethour <- c(23,0,1,2,3,4),
                  filename = '/Viz/BivariateAnalyses/Tubeplots/Temperature/AfterTemperature',
                  plot_title = 'Afternoon Minutes Temperature',
                  marker = list(size = 10),
                  printtochunk = F, export_html = T,
                  annotationkey  = annotationkey,
                  mycamera = list(eye = list(x = 1.7, y = 0.5, z = 0)))
  }
  
  # night
  
  if(isconda){
    
    groupTubePlot(dat, c, r, g, 
                  subsethour <- c(6,7,8,9,10,11,12),
                  filename = '/Viz/BivariateAnalyses/Tubeplots/Temperature/NightTemperature',
                  plot_title = 'Night Minutes Temperature',
                  printtochunk = F, export_orca = T,
                  marker = list(size = 10),
                  annotationkey  = annotationkey,
                  mycamera = list(eye = list(x = -1.3, y = 1.2, z = 0))) 
    
  }else{
    
    groupTubePlot(dat, c, r, g, 
                  subsethour <- c(6,7,8,9,10,11,12),
                  filename = '/Viz/BivariateAnalyses/Tubeplots/Temperature/NightTemperature',
                  plot_title = 'Night Minutes Temperature',
                  printtochunk = F, export_html = T,
                  marker = list(size = 10),
                  annotationkey  = annotationkey,
                  mycamera = list(eye = list(x = -1.3, y = 1.2, z = 0)))
    
  }
  
  # all
  
  if(!isconda){
    groupTubePlot(dat, c, r, g,
                  filename = '/Viz/BivariateAnalyses/Tubeplots/Temperature/Temperature',
                  plot_title = 'Minutes Temperature',
                  marker = list(size = 10),
                  export_html = T, printtochunk = F, 
                  annotationkey  = annotationkey,
                  mycamera = list(eye = list(x = 1.5, y = 1.5, z = 0)))
  }
  
}
```
